# Coordinated outcome-wide analytic methodology for multi-wave analyses of the global flourishing study

**DOI:** 10.1186/s44263-026-00287-6

**Published:** 2026-06-02

**Authors:** R. Noah Padgett, Chris Felton, Matt Bradshaw, Ying Chen, Richard G. Cowden, Eric S. Kim, Renae Wilkinson, Byron R. Johnson, Tyler J. VanderWeele

**Affiliations:** 1 Harvard T.H. Chan School of Public Health, Department of Epidemiology, Boston, MA USA; 2 Human Flourishing Program, Harvard University, Cambridge, MA USA; 3 Institute for Religious Studies, Baylor University, Waco, TX USA; 4 Institute for Studies of Religion, Baylor University, Waco, TX USA; 5 Institute for Global Human Flourishing, Baylor University, Waco, TX USA; 6 The University of British Columbia, Department of Psychology, Vancouver, BC Canada

**Keywords:** Global Flourishing Study, Methodology, Outcome-wide, Meta-analysis, Mastery

## Abstract

In this article, we describe the analytic design of the coordinated set of outcome-wide analyses used for examining longitudinal associations using data from the Global Flourishing Study (GFS), involving a multinational and multidisciplinary group of scholars. We discuss the benefits of outcome-wide analyses and provide details on controlling for high-dimensional confounders using principal components, accounting for complex sampling designs, imputing missing data, conducting sensitivity analyses for unmeasured confounding, meta-analyzing estimates of associations from across countries, and reporting results. We provide a brief illustrative example of the outcome-wide approach by estimating the association of Wave 1 sense of mastery with a wide range of Wave 2 outcomes. The example illustrates how results can be sensitive to analytic decisions, such as different coding strategies for the predictor and the number of principal components included. We conclude by outlining the major strengths and limitations of the employed methodology.

## Background

The Global Flourishing Study (GFS) is a comprehensive, multinational panel research project designed to investigate the patterns, influencing factors, and connections among various dimensions of human flourishing and well-being [1, 2]. It encompasses over 200,000 participants from a geographically and culturally diverse set of 22 countries and one territory [3–5]. In recent years, the concept of flourishing has garnered increasing attention across disciplines including psychology, economics, and public health [6–12]. Flourishing itself might be defined as “the relative attainment of a state in which all aspects of a person’s life are good, including the contexts in which that person lives” [13], and thus understood, flourishing is highly multi-dimensional [2]. Despite this growing interest, numerous facets of well-being remain insufficiently studied on a global scale, as much of the existing literature reflects predominantly Western viewpoints and uses data from these contexts [5, 14]. The multinational design of the GFS offers a unique opportunity to examine flourishing through a multicultural lens, thereby taking a step toward addressing this critical gap. This article provides details on the quantitative methods used to perform a coordinated set of longitudinal analyses with Waves 1 and 2 of the GFS, involving a multinational and multidisciplinary group of scholars, and building on similar coordinated sets of analyses conducted for Wave 1 data of the GFS [15].

The two waves of GFS data that are now available open a wide range of possible analytic pathways. One analytic approach that leverages this longitudinal structure is an outcome-wide design [16]. With two waves of data, an outcome-wide design examines the association of a single focal exposure assessed at Wave 1 with a wide range of outcomes assessed at a subsequent wave. This is particularly valuable because many of the constructs included in the GFS data are seldom included in large-scale cross-cultural cohort studies, see survey development report by Lomas et al. [17], providing a unique opportunity to strengthen existing knowledge about flourishing from a multinational perspective.

The present paper describes a coordinated outcome-wide approach we used in many different papers, with different focal exposures, employing the data from Waves 1 and 2 of the GFS. The paper lays out a common methodology for these papers. The author team of each specific paper preregisters their analyses with the same methodology. In each case, for each paper and exposure, all analyses are conducted separately by country using nationally representative samples. This preserves heterogeneity in the interpretation of survey items across countries and facilitates the contextualization of results. Then, country-specific results are pooled using meta-analytic techniques to summarize the cross-national associations between the focal exposure and different outcomes at Wave 2. These analyses provide a template for evaluating evidence concerning the potential determinants of flourishing across countries, and the use of a consistent methodological approach across manuscripts enhances the comparability of results across various exposures and flourishing outcomes.

This article is comprised of four core components. First, we provide a high-level description of the data. Second, we elaborate on the outcome-wide design and discuss the measures used as covariates and outcomes. Third, we discuss technical aspects of the methodology, namely the regression analyses, accounting for the complex sampling design, missing data imputation, meta-analysis, and sensitivity analyses. Then, we use the sense of mastery exposure as an example to illustrate the outcome-wide analytic approach and presentation of results. Lastly, we provide some general comments on the strengths and limitations of the employed analytic approaches.

### Global flourishing study data

Wave 1 of the GFS included nationally representative samples from 22 countries and one territory: Argentina, Australia, Brazil, China, Egypt, Germany, Hong Kong (Special Administrative Region of China), India, Indonesia, Israel, Japan, Kenya, Mexico, Nigeria, the Philippines, Poland, South Africa, Spain, Sweden, Tanzania, Turkey, the United Kingdom, and the United States (*N* = 207,919). The countries were selected to (1) maximize coverage of the world’s population, (2) ensure geographic, cultural, and religious diversity, and (3) prioritize feasibility in Gallup’s existing data collection infrastructure. Data for Wave 1 were collected from March 2022 to January 2024, except in China (March/April of 2024) [18]. Data for Wave 2 were collected from January 2024 to December 2024 in most cases with about a year or more between Wave 1 and Wave 2, but in China with Wave 2 data collected at least six months after Wave 1. In Table 1, we summarize the dates of data collection and the intervals between successive annual surveys that were administered, also see Supplemental material 1: Figs. S1–2. The GFS survey assesses aspects of well-being, including happiness and life satisfaction, physical and mental health, meaning and purpose, character and virtue, close social relationships, and financial and material stability [19], alongside a wide range of other demographic, social, economic, political, religious, personality, childhood, community, health, and well-being variables. Gallup translated the GFS survey into multiple languages following the translation, review, adjudication, pretesting, and documentation (TRAPD) model for cross-cultural survey research [18, 20, 21]. Details about the translation, cognitive interviewing, and pilot testing phases of the GFS can be found elsewhere [4, 17, 22]. More information about the sampling design and weighting procedures is available in Ritter et al. [18] and Padgett et al. [23].

**Table 1:** Differences in days between Wave 2 and Wave 1 GFS annual survey administration date by country

Country	Wave 1 Start	Wave 2 Start	Difference between Waves 1 and 2 administrations (in days)				
			Mean (SD)	Minimum	Q1	Q3	Maximum
Argentina	2023-01-31	2024-07-15	402.6 (94.9)	243	315	472	673
Australia	2022-03-29	2024-05-08	565.9 (146.0)	243	450	704	868
Brazil	2023-01-31	2024-07-23	405.4 (78.6)	240	359	438	687
China	2024-03-04	2024-10-22	239.8 (12.3)	197	233	250	270
Egypt	2023-03-29	2024-07-30	420.5 (30.2)	320	405	440	576
Germany	2022-10-17	2024-05-16	363.2 (57.5)	216	331	363	684
Hong Kong	2023-10-10	2024-07-15	301.9 (19.8)	274	287	311	386
India	2023-05-06	2024-07-23	406.2 (70.0)	229	355	462	580
Indonesia	2022-11-21	2024-06-19	550.0 (88.6)	265	529	608	717
Israel	2022-11-17	2024-06-17	394.0 (102.0)	210	298	472	713
Japan	2022-12-13	2024-03-01	350.1 (30.5)	277	331	376	459
Kenya	2023-05-25	2024-07-23	450.7 (65.6)	248	419	500	580
Mexico	2022-11-17	2024-07-23	394.0 (89.4)	250	323	451	733
Nigeria	2023-05-25	2024-07-25	414.3 (50.3)	261	383	443	564
Philippines	2023-04-16	2024-07-20	369.5 (70.8)	229	317	423	581
Poland	2023-01-27	2024-06-25	431.6 (50.6)	295	392	458	688
South Africa	2023-04-12	2024-07-22	401.2 (69.4)	229	356	446	595
Spain	2022-11-07	2024-05-16	348.6 (104.0)	227	274	376	733
Sweden	2023-01-16	2024-02-12	367.9 (18.9)	329	353	386	422
Tanzania	2023-03-16	2024-07-22	451.3 (63.4)	248	404	500	618
Turkey	2023-05-10	2024-07-17	369.9 (63.8)	233	326	416	531
United Kingdom	2022-06-10	2024-05-07	409.2 (111.0)	201	332	498	876
United States	2022-08-04	2024-01-22	483.2 (93.3)	295	360	538	661
Overall	2022-03-29	2024-01-22	417.5 (98.2)	197	344	520	876

Q1, 1st quartile of distribution of days; Q3, 3rd quartile of distribution of days

Development of the GFS survey occurred over eight distinct phases: (1) selection of core well-being and demographic items; (2) solicitation of additional social, political, psychological and demographic items from domain experts worldwide; (3) revision of the initial survey draft using multidisciplinary feedback from scholars around the world; (4) modification of survey items following input from experts in multinational, multiregional and multicultural survey research; (5) survey draft refinement based on compiled input from an open invitation to comment, posted publicly and sent to numerous listservs; (6) questionnaire optimization with support from Gallup survey design specialists; (7) adaptation of items from an interviewer-administered to a self-administered survey instrument using best practices for web survey design to minimize item non response, illogical responses, and incomplete responses; and (8) confirmation by scholars in several participating countries that translations appropriately captured each items’ intended meaning [5]. The data are publicly available through the Center for Open Science [24]. Additional information about methodology and survey development can be found in the GFS Questionnaire Development Report [3, 4, 17] as well as the GFS Methodology [18], GFS Codebook (https://osf.io/cg76b), and GFS Translations documents [20].

## Outcome-wide analytic design

An outcome-wide analytic approach [16, 25] was used to examine the associations of a single focal exposure at Wave 1 with a range of subsequent outcomes at Wave 2. Compared to traditional analytic strategies that tend to focus on a single, narrow set of outcomes, this approach provides a more holistic assessment of an exposure’s association with various outcomes related to flourishing. The outcome-wide analytic design (1) reduces researcher degrees of freedom [26] in analyses by ensuring a consistent analytic strategy and the same set of covariates across models for all outcomes; (2) mitigates outcome reporting bias because results for all examined outcomes are reported simultaneously, even null results; and (3) provides insights into a fuller range of beneficial, harmful, and null associations with the exposure. Further details about the outcome-wide approach can be found elsewhere [25]. Nevertheless, the outcome-wide design, as with any analytic approach, has limitations worth noting. Because it addresses many outcomes within a single study, the approach inevitably sacrifices some depth in exchange for breadth. In addition, while applying consistent analytic strategies to assessing all outcomes facilitates comparison of effect sizes across outcomes, each of the outcomes may warrant more tailored statistical models to minimize bias or variance.

Similar to the coordinated Wave 1 GFS childhood predictors analyses [27], all analyses are initially conducted separately for each country and then pooled using random-effects meta-analyses. The random-effects meta-analytic approach was chosen because it does not assume that the country-specific regression coefficients being pooled are necessarily representative of associations estimated from repeated samples within the same population of respondents; rather, it assumes that the associations vary across countries, forming distinct populations with their own effects. A meta-analytic approach was also considered preferable over direct estimation via multilevel models because it does not presume cross-national measurement equivalence. Instead, this approach merely assumes that the survey item measures the same underlying construct, not that item reliability and measurement error are necessarily equal across countries. Cognitive testing during the survey development process showed some variation in the interpretation of some items across countries [22, 28], suggesting that it may be preferable to treat the measures as closely related, but not identical, assessments of each construct across the countries. As a result, the summary statistics by country are only partially comparable across countries, and direct comparison of any pair of countries should be interpreted with caution because different results across countries may reflect heterogeneity in measurement error rather than heterogeneity in the associations of the focal exposure with various aspects of well-being [29]. For these reasons, we conducted separate analyses for each country. A country-wise approach preserves potential heterogeneity in the interpretation of survey items across countries, allowing results to be contextualized within each country’s sociocultural particularities, while also enabling meta-analytic estimates to represent the average of these country-specific associations.

### Variables in analyses

#### Covariates

Country-specific analyses controlled for 17 covariates (9 demographic and 8 childhood variables) unless data were not available (described below). Additional details on all variables can be found in the GFS Codebook (https://osf.io/cg76b) or Crabtree et al. [3, 4].

*Demographic covariates:*

1. Gender was assessed as male, female, or other.
2. Year of birth (age) was classified into 1998–2005 (18–24 years), 1993–1998 (25–29 years), 1983–1993 (30–39 years), 1973–1983 (40–49 years), 1963–1973 (50–59 years), 1953–1963 (60–69 years), 1943–1953 (70–79 years), or 1943 or earlier (80 years or older).
3. Marital status was assessed as single/never married, married, separated, divorced, widowed, or domestic partner.
4. Employment was assessed as employed, self-employed, retired, student, homemaker, unemployed and looking for a job, and none of these/other.
5. Education was assessed as up to 8 years, 9–15 years, or 16 or more years.
6. Religious service attendance was assessed as more than once a week, once a week, one to three times a month, a few times a year, or never.
7. Immigration status was assessed with yes/no responses to: *Were you born in this country, or not?*
8. Religious affiliation was also assessed in all countries. Religious affiliation response category options included Christianity, Islam, Hinduism, Buddhism, Judaism, Sikhism, Baha’i, Jainism, Shinto, Taoism, Confucianism, Primal/animist/folk religion, Spiritism, Umbanda, Candomblé, and other African-derived religions, Chinese folk/traditional religion, some other religion, or no religion/atheist/agnostic. There were considerable cross-country differences in the response categories endorsed by participants, as some religious affiliations are only applicable in certain countries and not in others. When more than 5% of a within-country sample endorsed the no religion/atheist/agnostic category, this was used as the reference category in the country-specific analyses; otherwise, the most prominent religious group was used. Additionally, all religious affiliation categories endorsed by less than 3% of a within-country sample were collapsed into a single religious affiliation category.
9. Racial/ethnic identity was assessed in most countries but was not collected in China, Germany, Japan, Spain, or Sweden. Response categories varied across countries as appropriate. Country-specific analyses that controlled for racial/ethnic identity used a binary variable based on whether an individual was in the most prominent racial/ethnic group in the sample versus a minority racial/ethnic group. Additional details about the measurement of the sociodemographic variables can be found in the GFS Codebook (https://osf.io/cg76b).
*Retrospective childhood covariates:*
10. Relationship quality with mother during childhood was assessed with the question: *Please think about your relationship with your mother when you were growing up. In general, would you say that relationship was very good, somewhat good, somewhat bad, or very bad?* Responses were dichotomized as very/somewhat good versus very/somewhat bad. *Does not apply* was treated as a dichotomous control variable for respondents who did not have a mother due to death or absence.
11. An analogous variable was used for relationship quality with father.
12. Parental marital status during childhood was assessed with responses of married, divorced, never married, or one or both had died.
13. Subjective financial status was measured with: *Which one of these phrases comes closest to your own feelings about your family’s household income when you were growing up, such as when YOU were around 12 years old?* Responses were lived comfortably, got by, found it difficult, or found it very difficult.
14. Abuse was assessed with yes/no responses to *Were you ever physically or sexually abused when you were growing up?*
15. Subjective family non-belonging was assessed with the question: *When you were growing up, did you feel like an outsider in your family?* Response options were yes/no.
16. Childhood health was assessed by asking: *In general, how was your health when you were growing up? Was it excellent, very good, good, fair, or poor?*
17. Religious service attendance during childhood was assessed with: *How often did YOU attend religious services or worship at a temple, mosque, shrine, church, or other religious building when YOU were around 12 years old?* with responses of at least once/week, one-to-three times/month, less than once/month, or never.

#### Outcome variables

Fifty-six main Wave 2 outcomes were examined across domains of psychological well-being, psychological distress, social well-being, social distress, social participation, character and prosocial behavior, physical health and health behaviors, and socioeconomic outcomes. The list of Wave 2 outcomes is provided in Table A1 and is also available on the Center for Open Science (COS) website (https://osf.io/9kpd8). Further details on item wording can be found in Crabtree et al. [3, 4].

The outcomes also included a composite flourishing index that was constructed using two items from each of six domains: happiness & life satisfaction, mental & physical health, meaning & purpose, character & virtue, close social relationships, and financial & material stability [15, 19]. We also constructed another version of this index that excludes financial & material stability, as this particular domain is sometimes considered a means of flourishing rather than an end in itself. In this sample, the estimated coefficient alpha for the twelve items is 0.88 based on the complete-case Wave 2 sample. At the country level, coefficient alpha ranged from 0.75 in Nigeria to 0.94 in Japan.

Additional outcomes reported in the supplement include the six specific domains of the composite flourishing index, specific symptoms of depression and anxiety (two each), and 12 indicators related to religion/spirituality.

#### Focal exposures

Different research teams focus their efforts on a separate focal exposure from Wave 1, each predicting the wide range of Wave 2 outcomes described in the *Outcome variables* section. The range of exposures includes all the outcomes. The exposures are generally coded in the same way as were used in Wave 1 analyses to allow for comparability of results across papers. Each team preregisters how they will analyze the exposure as well as any additional sensitivity analyses to how the exposure is coded. In our example analysis with sense of mastery, we provide an example of the scope such sensitivity analyses can take.

#### Measurement invariance testing

Measurement invariance of the outcomes at Wave 2 is difficult to assess in these data because nearly all outcomes are measured using single-item assessments of various constructs. The only outcomes assessed by more than one item are the 12-item Secure Flourishing Index, the 10-item Flourishing Index, the 2-item depression composite, and the 2-item anxiety composite. We have assessed measurement invariance of the Secure Flourishing Index and the depression and anxiety composites.

For the Secure Flourishing Index, measurement invariance was assessed using moderated nonlinear factor analysis and traditional multiple-group confirmatory factor analysis, yielding evidence of partial metric and scalar invariance with respect to country, age, and gender. The interested reader is referred to Padgett, Johnson, and VanderWeele [30] for an in-depth discussion of the invariance of the Secure Flourishing Index in these data. To summarize, age had a significant moderating effect on several factor loadings, item intercepts, and a few item residual variances. For example, age had a positive moderating effect on the factor loading for the Physical Health item, while it had a negative moderating effect on the factor loading for the Mental Health item, suggesting that the two indicators of health are more or less informative about different aspects of health depending on age. Gender (being female vs male) had a relatively small moderating effect on the intercept for the Financial Security item, but no effects on factor loadings or item residual variances. Respondent country moderated at least one item parameter (factor loading, item intercept, or residual variances) for each of the country indicator variables. Country differences in the measurement model parameters varied with no clear pattern. In total, we found evidence of partial invariance of all measurement model parameters to some extent, though the non-invariance occurred most frequently in the item residual variances.

The 2-item depression and 2-item anxiety composites were created from the two facets of the 4-item Patient Health Questionnaire (PHQ-4) [31]. The measurement invariance of the 2 dimensions was assessed using separate-by-country confirmatory factor analysis (CFA) to help assess configural invariance and multi-group confirmatory factor analysis for higher levels of constraints in the measurement model (using ordinal factor analysis identification constraints). The analyses are reported on in our Supplementary material 2 file, and here we briefly summarize the results. The two-factor model fits well within each country, with standardized root mean residual (SRMR) being less than 0.02 in all countries, and the root mean square error of approximation (RMSEA) was below 0.06 in all countries except Israel, which had an RMSEA of 0.07 (90% CI: 0.04, 0.11). These results provide support for the two-factor structure being invariant across countries within the GFS. Based on the multi-group CFA analyses (with covariance matrix and person-level data input), we conclude that there is partial invariance with respect to the thresholds (see measurement invariance supplement for technical details). Once the subset of thresholds was freely estimated, we found evidence of approximate metric invariance across all countries. This means that factor loadings are approximately equal across all countries, implying that these items load on the depression and anxiety factors approximately the same across all countries. The model fit statistics did not show a meaningful discrepancy between the configural model, partial-threshold invariance model, and the metric invariance model. While the RMSEA is relatively high (0.06) in the metric invariance model, the other fit indices, comparative fit index (CFI) was 0.997, Tucker-Lewis Index (TLI) was 0.997, and SRMR was 0.009, which are quite good for such a large sample and large number of countries. The SRMR is especially excellent where the model-implied correlations were, on average, less than 0.01 correlation units off. The largest (though still small) discrepancy tended to occur in the correlation between items *loss of interest* and *worry about control*, with the largest discrepancy of −0.06 in Egypt. Based on these results, we conclude that the PHQ-4 two-factor model has evidence of metric invariance in Wave 2 of the GFS.

In addition to the tests for the multi-item scales above, we developed an approach to testing approximate invariance of single-item measures using a penalized heteroskedastic ordered probit model (HETOP) [32]. The test provides a preliminary evaluation of the equality of item location (e.g., item intercepts) and the strength of the association between the latent variable and the observed item (factor loading or discrimination parameter). The penalized HETOP model uses the discrete nature of (nearly) all outcome variables in the GFS, along with an item response theory-like parameterization for ordered categorical variables, to test for differential item functioning (DIF). More technical details are provided in Supplementary material 3 and in Padgett [32]. The goal of those analyses is to explore how the degree of between-group differences in the core measurement model parameters varies across groups and how these differences depend on the level of penalization used in estimation. We apply this approach to the focal predictor variable used in our illustrative analysis, *Sense of Mastery*, and to a few of the outcomes here.

This form of DIF analysis for a single-item assessment does not provide a clear decision about measurement invariance, and probably no analysis of single-item measures can. However, the analyses can provide a sense of the degree of separation between groups as a function of the measurement model parameters. The results of these analyses are summarized in Supplemental material 1: Table S4, which identifies countries that may exhibit uniform or nonuniform DIF for each single-item assessment. The analyses suggest potential measurement issues in India, Kenya, and Tanzania, with low item discrimination (nonuniform DIF) identified across many of the items that use an 11-point response scale. Respondents in Japan and Poland also showed significantly lower or higher responses across many outcomes, suggesting a potential response tendency in these countries. While these preliminary DIF results are not definitive, they provide some indication that, on average, the items are functioning similarly across most of the countries included in the GFS.

#### Measurement limitations and reliability

Almost all the variables used in these studies are measured using single-item assessments. Although single items can effectively capture the essence of a construct, they can limit the conceptual coverage and reliability of insights derived from these analyses. Several childhood characteristics were dichotomized to mitigate multicollinearity (e.g., relationship with mother/father), and the cut-points were chosen in consultation with researchers at Gallup to select categories that were substantively meaningful. These choices involve trade-offs: potential increase in variance of estimated coefficients due to multicollinearity among the exposures, versus the insight gained from using substantively meaningful categories. These decisions are documented in a methods article describing the prior coordinated childhood predictor analyses using Wave 1 data from the GFS [27].

Variables with Likert-type response scales were also dichotomized based on substantive grounds to provide a meaningful and simplified response scale for use as outcomes in the modified Poisson regression analyses. As part of the coordinated analyses and accompanying papers, all outcomes were either analyzed as approximately continuous or as binary, allowing for easier comparison across outcomes in alignment with analytic decisions by primary author teams applied to the Wave 1 coordinated analyses. A major limitation of this decision is that variation captured by responses on items with more than two response options is reduced, potentially masking some important aspects of variation. Ordinal regression models for Likert-type response scales were deemed less suitable because their coefficients cannot be concisely summarized and interpreted when the exposure effects vary across categories, which is especially challenging in the context of an outcome-wide study.

Another potential limitation of single-item assessments is reduced reliability. Low reliability attenuates the estimated association between variables [33]. Low reliability also increases imprecision in estimating variances [34]. Imprecise variance estimates within each country can have a notable impact on our results because we pool standardized estimates of associations across countries using meta-analytic techniques. When the estimated standard deviations of the predictor and outcome are themselves measured with error, the target parameter of interest—the standardized regression coefficient—is likewise measured with error, increasing the uncertainty in our results. To empirically investigate the influence of potential unreliability in single-item assessments, we use an assumed reliability-corrected estimate of association (see section *Sensitivity to unreliability* for more details).

### Statistical analysis

**Fig. 1:**
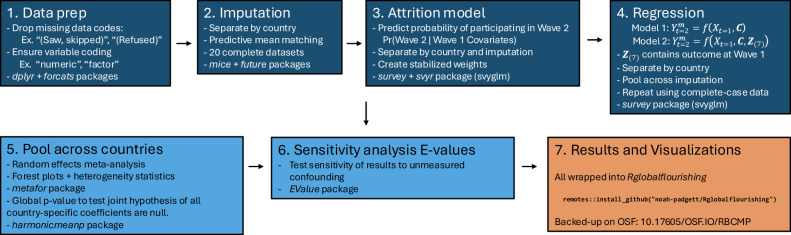
Flowchart and high-level summary of procedures and statistical analyses

This section describes the statistical analyses performed as part of the outcome-wide design. The analyses conducted include: (1) sample descriptive statistics; (2) handling of missing data; (3) country-specific outcome-wide regression analyses; (4) aggregating results across countries using meta-analytic techniques; and (5) sensitivity analyses to evaluate potential unmeasured confounding. Individual studies in this coordinated series follow the same analytic approach and have the analyses preregistered with COS. To facilitate analyses and reporting, we developed the open-source *Rglobalflourishing* package [35] for use with R 4.5 [36]. The code, which is openly available through GitHub (https://github.com/noah-padgett/Rglobalflourishing) and an Open Science Framework project (10.17605/osf.io/rbcmp), can be freely installed using the R programming language. The current paper helps provide technical documentation for the analyses conducted, which primarily used the *mice* package [37] for imputation and the *survey* package [38] for handling complex survey design adjustments. A high-level summary flow-chart of the analyses is provided in Fig. 1 to aid in transparency and reproducibility.

#### Descriptive statistics

Descriptive statistics were estimated for the focal exposure, control variables, and outcome variables at both waves using the *survey* and *gtsummary* packages [39]. All reported counts, proportions, means, and standard deviations are based on the weighted data. For all summary statistics of Wave 1 data, we used the Gallup-provided sampling weight for the full panel (e.g., the weight variable ANNUAL_WEIGHT_R2). For the summary statistics of Wave 2 data, we first restricted the sample to only those respondents with any responses to a Wave 2 variable (i.e., the “retained” sample). Then, the statistics were obtained using attrition-weight-adjusted sampling weights. We describe the attrition weights in more detail in the section on missing data.

#### Missing data, imputation, & attrition weights

The primary source of missing data was attrition between Waves 1 and 2. The retention rate varied from 23% in Hong Kong to 90% in China, and the overall retention rate was 62%. The sample sizes (by wave) and retention rates are shown in Table 2. The high rate of missingness introduces considerable uncertainty in longitudinal analyses for some countries/territories, such as Hong Kong, where the drop in sample size is particularly concerning. This leads to the need to account for missingness in more than one way to try to evaluate the sensitivity of our results to how missingness was handled.

**Table 2:** Summary of sample size and retention rates across countries

Country/Territory	N Wave 1	N Wave 2	N Dropped	% Retained
China	5022	4544	478	90.5
United States	38,312	32,245	6067	84.2
Sweden	15,068	11,609	3459	77.0
Japan	20,543	13,972	6571	68.0
Israel	3669	2490	1179	67.9
Kenya	11,389	7698	3691	67.6
United Kingdom	5368	3619	1749	67.4
Australia	3844	2582	1262	67.2
Egypt	4729	3040	1689	64.3
Poland	10,389	6478	3911	62.4
Tanzania	9075	5583	3492	61.5
Germany	9506	5529	3977	58.2
Philippines	5292	2682	2610	50.7
India	12,765	6374	6391	49.9
Spain	6290	2924	3366	46.5
Nigeria	6827	3146	3681	46.1
Argentina	6724	2932	3792	43.6
Mexico	5776	2278	3498	39.4
Indonesia	6992	2684	4308	38.4
South Africa	2651	978	1673	36.9
Turkey	1473	500	973	33.9
Brazil	13,203	4274	8929	32.4
Hong Kong	3012	707	2305	23.5
Overall	207,919	128,868	79,051	62.0

The primary analyses include all participants from Wave 1 or Wave 2, including those not observed at Wave 2—imputing 100% of the missing data for the attritors [40]. This all-cases approach mirrors our Wave 1 analyses. Additionally, using fully imputed data for our primary analysis facilitates planned future analyses, as some participants may return to complete Wave 3 after not responding in Wave 2. Aligning the analytic sample across years via multiple imputation will facilitate the comparison of results.

An alternative to multiple imputation is to estimate weights for the conditional probability of retention given covariates and then re-weight non-attritors by these inverse probability weights. This approach will be unbiased—that is, produce unbiased association estimates—when the attrition model is correctly specified. Similarly, multiple imputation will be unbiased when the imputation model is correctly specified. In the presence of unmeasured confounding between attrition and the outcome of interest, however, both methods will be biased [41, 42]. Nonetheless, the analysis using retention weights can help us assess the sensitivity of results to misspecification in the imputation model and to the specific assumptions made about missing data [43].

##### Multiple imputation

Imputation of data for the primary analyses is conducted by country. Imputation is conducted using multivariate imputation by chained equations with predictive mean matching [37]. The within-country imputation approach ensures that the imputation model accurately reflects country-specific contexts and assessment methods. To approximate the minimum number of necessary imputed datasets, we approximated the fraction of missing information (FMI) in the observed dataset [44]. Excluding variables not measured in some countries (e.g., “ever abused” in Israel, “say in government” in China), the FMI for the full dataset is 0.61, which ranges from 0.18 in China to 0.84 in Hong Kong. The maximum number of imputed datasets needed for the efficiency (FMI/m) to be less than 0.05 would be 0.84/0.05 ≈ 17. This supports the use of our preregistered 20 imputed datasets for stable variance estimation.

The set of predictors for Wave 1 missing observations includes the sampling weights, mode of survey administration, age, gender, education, income, employment status, marital status, race/ethnicity (when possible), urban/rural status, and retrospective childhood characteristics. See Padgett, Bradshaw, et al. [27] for a similar imputation setup for the coordinated analyses using Wave 1 GFS data.

For imputing missing data at Wave 2, the same set of variables is used for imputation, plus all Wave 1 variables. For each imputation model, a predictor will be omitted if there is no variation in the variable within a country or if the variable was not measured in a country (e.g., race/ethnicity in Japan).

A major assumption when using multiple imputation to account for missingness is that the imputation model is correctly specified. The imputation model would be misspecified if missingness is dependent on variables not included in the model. Given the robust set of demographic, childhood, and exposure variables used, we believe that the imputation model approximates the true missingness model reasonably well. However, we also used an alternative approach to handling missingness in longitudinal studies by conducting a semi-complete-case analysis that combined attrition weights and multiple imputation of the item-level missingness for those observed in both waves [45, 46]. We describe the methods for the attrition-weighted semi-complete-case analyses next.

##### Attrition weights

Attrition weights are created by first using a logistic regression model to predict the probability of retention (that is, the probability that the participant did not drop out). The attrition weights are then the stabilized inverse probability of retention [42]. Attrition predictors include the following: sampling weight, strata (when possible), mode of survey in Wave 1 (when it varies within country), age, gender, education, income, employment status, marital status, race/ethnicity (when possible), religious service attendance, urban/rural status, Big Five personality traits, days of exercise, depression, loneliness, and all domains of the Flourishing Index (happiness & life satisfaction, mental & physical health, meaning & purpose, character & virtue, close social relationships, financial & material stability). These characteristics were chosen to align with the common set of confounder controls reported for published outcome-wide studies [25], and in recognition of evidence that some aspects of wellbeing [46] and personal values are related to attrition [47, 48]. In total, these variables provide a reasonably comprehensive set of predictors of attrition while being concise enough to avoid severe multicollinearity.

To account for the small amount of missingness in the predictors of attrition, we estimated the logistic regression models after multiple imputation. The attrition predictor with the largest amount of missingness, at 1.5% of the full dataset, was the number of days of exercise per week. The attrition model was fit to each of the 20 imputed datasets. A stabilized weight was then created and appended to each imputed dataset. This attrition weight was multiplied by the provided sampling weight to construct an analysis weight for use in a supplemental semi-complete-case analysis [49]. For summary statistics of the semi-complete-case data, the analysis weight was averaged across imputed datasets to create a single weight per case, which was then appended to the raw data with missingness.

#### Regression analyses

Within each country, we conducted a series of weighted linear (for approximately continuous outcomes) and weighted modified Poisson (for binary outcomes) multivariate regression analyses, employing the sampling weights and survey design factors described in the *Sampling design* section. Similar to the Wave 1 coordinated childhood predictor analyses [27], we assume a generalized linear model for outcome ***Y***

$${\boldsymbol{E}}\left[{\boldsymbol{Y}}\right]=f\left({\bf{X}}{\boldsymbol{\beta }}\right),$$

where *f*(.) is the link function, which is either the identity function for continuous outcomes or the exponential function for modified Poisson regression; **X** is the design matrix of focal exposure and covariates; and ***β*** is the vector of regression coefficients

When the outcome is binary, we conducted modified Poisson regressions. Modified Poisson is a popular approach to estimating risk ratios (RR) [50]. The exponentiated coefficients from the results of the modified Poisson are interpreted as risk ratios: a risk ratio is the ratio of the probability of an outcome in an exposed group to the probability of an outcome in an unexposed group. Logistic regression was avoided for binary outcomes because the resulting odds ratio estimates are often misinterpreted as risk ratios. This is especially problematic and inaccurate when binary outcomes are common (prevalence between 0.10 and 0.90), as is the case for many binary outcomes in the GFS. To enhance comparability of results across outcomes and to facilitate interpretation, we relied on only modified-Poisson risk ratios for binary outcomes. However, risk ratios are not invariant to re-labelling of outcome categories, meaning that the risk-ratio for responding “yes” over “no” as the reference category is not simply the inverse of the risk-ratio when the model is fit with “yes” as the reference category instead. Readers should be cautious not to misinterpret these risk ratios as odds ratios.

An alternative to the modified Poisson approach to estimating risk ratios is the log-binomial model [51]. While using the log-binomial approach may be worth exploring, the estimation tends not to converge when cells of the design matrix are empty [52]. The analyses we are conducting use a relatively large number of controls in countries with varying sample sizes, sometimes relatively small, such as in Turkey for the semi-complete-case analyses, leading to a high probability of encountering convergence issues for at least some outcome-predictor-country combinations. In contrast, using the modified Poisson approach did not lead to convergence issues in our prior retrospective childhood predictor analyses [27], which similarly involved multiple binary outcomes, extensive controls, country-specific models, and varying sample sizes. Therefore, it is used again here as our approach to estimating risk ratio.

Two primary models were estimated. Model 1 specifies each outcome as a function of the focal exposure and demographic and retrospective childhood variables. Model 2 specifies each outcome as a function of the focal exposure, demographic variables, retrospective childhood variables, and principal components based on all prior values of the outcomes assessed at Wave 1 (i.e., baseline values of all other relevant indicators of flourishing available in the GFS), excluding the focal exposure.

**Fig. 2:**
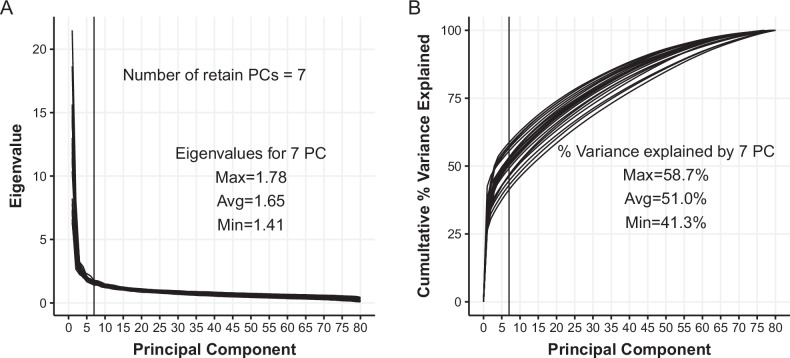
Principal component eigenvalue and percent of variance explained of the full set of potential contemporaneous covariates within each country shows how the proportion of variance explained in the first seven components varies by country (each line in the graph represents a separate country). **A** The value of each eigenvalue for all extracted principal components across all countries. **B** The cumulative percent of variable explained in the full set of Wave 1 variables used to construct the PCs across each country. The complete case sample size for each principal component analysis varied from 1115 in Turkey to 31,607 in the United States. Each line/point represents the eigenvalue/proportion of variance explained for each country. Country-specific values are provided in our Supplemental material 1: Tables S1–2

Model 2 has a large number of predictors (17 demographic and childhood variables, and 79 contemporaneously measured potential confounders), which increases the likelihood of issues with multicollinearity. To address this issue, principal components were used to reduce the dimensionality of the design matrix of contemporaneous covariates while maintaining an estimable and interpretable regression model to mitigate the influence of multicollinearity [53]. Using the GFS Wave 1 complete-case data, Fig. 2A displays a scree plot of the eigenvalues from the correlation among the contemporaneous potential confounders, and Fig. 2B displays the corresponding cumulative proportion of variance explained by each principal component. In these data, seven principal components accounted for an average of 51.0% of the variability in all the contemporaneous confounders. Additional principal components explained only between 1–2% of the variability each. We therefore selected seven principal components to balance the number of additional predictors in the regression model and the amount of variability explained. In the *Sensitivity of Results to Analytic Decisions* section, we provide some empirical support that results do not change substantially regardless of the number of retained principal components when the total percent of variance accounted for by the retained principal components is above 40–50%. Regardless of the focal exposure, the same number of principal components are extracted for consistency across studies.

#### Meta-analysis

The 22 countries and one territory were selected to provide broad geographical, cultural, and religious representation; they encompass all six populated continents and account for approximately two-thirds of the world’s population. Random effects meta-analyses were conducted and may be interpreted as estimating the pooled associations of each focal exposure and the standard deviation of the distribution of associations across countries from a hypothetical underlying population of which our sample of countries would be representative. While such an underlying population is hypothetical, this was viewed as a reasonable target of interest given the broad geographic coverage of the GFS. However, the results for each of the samples are also provided, which are of interest in their own right, and may also be useful for readers who would prefer not to consider this underlying hypothetical population.

A general random effects model was used assuming the distribution of effect sizes in the population is normally distributed [54–57], that is:

$${b}_{i} \sim {Normal}({\theta }_{i},{v}_{i})$$

$${\theta }_{i} \sim {Normal}\left(\mu ,{\tau }^{2}\right),$$

where *b*_*i*_ is the estimated (un)standardized effect of the focal exposure within country *i*, *v*_*i*_ is the variance/uncertainty of *b*_*i*_ within country (i.e., the squared standard error of the regression coefficient), *θ*_*i*_ is the unobserved true effect for the focal exposure without error in country *i*, *μ* is the population average effect, and *τ*^2^ is the estimated variance/heterogeneity of *θ*_*i*_ across countries. The model was estimated using the Paule and Mandel estimator [58–60].

The effect sizes being meta-analyzed effects—*b*_*i*_ —are obtained from the country-specific regression analyses. The focal effect sizes will be standardized using the within-country standard deviations of the focal exposure and outcome. The standardized versions of *b*_*i*_ and *v*_*i*_ are obtained by multiplying the unstandardized effect size by the ratio of the standard deviations for the predictor divided by the outcome. When modified Poisson regression is employed for binary outcomes, the effect sizes and variances are only multiplied by the predictor standard deviation. While the main results of each outcome-wide analysis are the standardized estimates for associations with the focal exposure of interest, unstandardized estimates will also be reported in the supplemental material of the respective manuscripts.

When a focal exposure was highly collinear with a covariate included in the within-country analysis, the resulting standard error of the effect can be large relative to the magnitude of the effect. Meta-analyzing several relatively imprecise effect estimates can result in the heterogeneity of effects being severely underestimated (${\hat{\tau }}^{2} < 0.01$). A low estimate aligns neither with the observed differences in country-specific effects nor with prior theoretical considerations. It is likely driven by the large standard errors arising from multicollinearity. As mentioned in the *Regression Analyses* section, multicollinearity was mitigated to some degree using principal components of the covariates, but it was not fully eliminated. When conducting the meta-analyses, the use of the Paule and Mandel estimator reduced the frequency of underestimated heterogeneity occurring relative to restricted maximum likelihood in preliminary testing; however, even the Paule and Mandel estimator did not completely eliminate the occurrence of this issue. In our results, we have noted when the estimates of heterogeneity based on the random effects meta-analyses are potentially smaller than one would expect and refer readers to the accompanying online supplement where the forest plots provide additional information about the heterogeneity (e.g., Q-statistics and Q-profile confidence intervals). Additional sensitivity and influence metrics are also reported, including leave-one-country-out pooled estimates, studentized residuals, and a Cook’s D-like statistic [61].

The meta-analytic estimate of the association between the focal exposure from Wave 1 and each outcome was reported, along with the 95% confidence interval, estimate of heterogeneity (tau), and a global *p*-value. The global *p*-value is an omnibus test of whether there is evidence of a significant association in at least one country in this sample [62, 63]; that is, testing the joint hypothesis that all country-specific associations are null. The Bonferroni corrected significance threshold for multiple testing is applied to the significance cutoff determined by the number of outcomes assessed. Although less conservative multiplicity adjustments are available, we chose the Bonferroni correction because it provides an interpretable benchmark with well-understood properties. Global *p*-values are treated as providing a high-level indication of whether there is little evidence of cross-country variation in the association of the focal exposure and outcome, or whether the pooled estimate of association is near the null, but may be masking cross-country heterogeneity.

All meta-analyses were conducted using the *metafor* package [54].

#### Sensitivity analyses

Two types of sensitivity analyses were conducted. First, we describe analyses evaluating the potential effects of using single-item measures on the estimates of association. Second, we describe the analyses conducted to evaluate the sensitivity of the results to potential unmeasured confounding. Together, these two sensitivity analyses provide evidence for (or against) the robustness of the results to threats to the validity of our analyses’ interpretation.

#### Sensitivity to unreliability

Spearman [64] developed an approach to correcting correlation coefficients for the unreliability of each variable being assessed, or “attenuation by errors.” That is, the observed correlation r_obs_ between variables X and Y is corrected using the reliability of each (*λ*_*x*_, *λ*_*y*_), resulting in a corrected correlation of ${r}_{{corrected}}=\frac{{r}_{{obs}}}{\sqrt{{\lambda }_{x}{\lambda }_{y}}}$. See [65] for a broader discussion of the correction, which has a long history outside the scope of the current paper to summarize, see also [66]. We apply the correction to the estimated standardized regression coefficients. The correction can be used by first correcting the country-level results, then meta-analyzing the corrected estimate for each set of corrected results, or by correcting the meta-analytic estimates themselves.

For outcome-wide studies using the GFS, obtaining estimates of reliability from the data is generally not possible because nearly all constructs are measured with single-item assessments. However, we can use an assumed level of reliability to evaluate how changes in reliability may affect our conclusions about the magnitude of effects. Instead of using separate reliabilities for the predictor and outcome, we can simplify the reporting of the sensitivity analysis by using a single factor (i.e., let $\lambda =\sqrt{{\lambda }_{x}{\lambda }_{y}}$) that represents the geometric mean of the reliability of each variable. We can interpret the effects of the average reliability of the predictor and outcome on the estimates of association after correcting for unreliability using the following:

$${\hat{b}}_{{corrected}}=\frac{\hat{b}}{\lambda }$$

$${SE}\left({\hat{b}}_{{corrected}}\right)=\frac{{SE}\left(\hat{b}\right)}{\lambda }$$

The corrected estimate and standard error are then used to construct confidence intervals to explore the effects of unreliability on inferences. The sensitivity analysis for unreliability tests can be summarized by examining what level of hypothesized reliability leads the confidence interval to contain the null. We propose three levels of reliability to test, limiting the breadth of results while still assessing a range of plausible levels for constructs: a conservative level (λ = 0.40), a moderate level (λ = 0.55), and an optimistic level (λ = 0.70). Single-item assessments have limited conceptual coverage of each construct and are commonly less reliable than multi-item scales, which typically have internal consistency estimates of reliability above 0.70 for psychological constructs. This makes a reliability upper limit of 0.70 seem optimistic for sensitivity testing, while a lower limit of 0.40 for reliability would indicate that less than half of the variance in observed scores can be attributable to actual differences in the construct of interest. While lower limits are certainly plausible for single-item measures, using 0.40 means all effect sizes are multiplied by 2.5. Such a strong multiplier is quite extreme disattenuation in our view, and any more would provide an overly optimistic view of the possible magnitude of associations. We apply this sensitivity analysis to our example analysis with *Sense of Mastery*.

#### Sensitivity to unmeasured confounding

For each reported association between the focal exposure from Wave 1 and outcomes at Wave 2, we calculated E-values to evaluate the sensitivity of results to potential unmeasured confounding. An E-value is the minimum strength of the association on the risk ratio scale that an unmeasured confounder must have with both the outcome and the exposure, above and beyond all measured covariates, for an unmeasured confounder to explain away an association [67, 68]. E-values range from 1.0 to infinity. A high E-value signifies that an unmeasured confounder would need to have strong associations with the focal exposure and the outcome to fully explain away the observed association, suggesting that the results are more likely to reflect a true causal relationship. An E-value closer to 1 signifies the opposite, where the observed association may be explained away by an unmeasured confounder that has a weak relationship with the outcome and the exposure. Approximate E-values can be obtained for continuous outcomes through scale conversions [67]. E-values for effect sizes were estimated using the *EValue* package [69] in R.

In addition to the E-value for the estimated regression coefficient for the focal exposure, another E-value can be estimated for the confidence interval of the regression coefficient. E-values for the confidence limit closest to the null denote the minimum strength of association on the risk ratio scale that an unmeasured confounder would need to have with both the exposure and the outcome to shift the CI to include the null value, conditional on the measured covariates [67].

One of the difficulties of interpreting E-values is determining whether the estimated E-value is large enough to suggest the results are relatively robust to unmeasured confounding. To help with this, we developed an approach to calibrate the E-value based on an observed confounder, such as gender, or employment, or other covariates. Because such confounders are observed, we can estimate their associations with the exposure and outcome to compute a benchmark E-value. We then compare the estimated E-value with the benchmark. If the estimated E-value is smaller than important benchmarks, and we suspect that an unmeasured confounder may have stronger associations with the exposure and outcome than these other covariates (e.g., gender, employment, etc.) do, we would conclude that the estimated association is not especially robust to plausible amounts of unmeasured confounding. If the estimated E-value is larger than the benchmarks, and we doubt the existence of an unmeasured confounder with stronger associations, then we would conclude that the estimated association is relatively robust.

More technically, the E-value describes the association between an exposure, outcome, and confounder on the scale of risk ratios. The interpretation of the E-value requires navigating a somewhat complex set of nested min-max operations on pairs of risk ratios [67]. Let *D* be a binary exposure, and let *Y* be a binary outcome. Let *U* represent a binary unmeasured confounder with levels *k* = 0, 1. For expository purposes, consider calculating the E-value for an unconditional association between the exposure and outcome. We begin by defining two core sensitivity analysis parameters: one relating the exposure to the unmeasured confounder (*RR*_*DU*_) and one relating the outcome to the unmeasured confounder (*RR*_*UY*_). The risk ratio of the exposure on the unmeasured confounder is

$${{RR}}_{{DU}}\equiv \max \left(\frac{\Pr \left(U=0{\rm{| }}D=1\right)}{\Pr (U=0{\rm{| }}D=0)},\frac{\Pr \left(U=1{\rm{| }}D=1\right)}{\Pr (U=1{\rm{| }}D=0)}\right),$$

where *RR*_*DU*_ is the maximum relative prevalence of the exposure across levels of the unmeasured confounder. Next, define three risk ratios capturing the relationship between the unmeasured confounder *U* and the outcome *Y* within levels of the exposure:

$${{RR}}_{{UY}|D=0}\equiv \frac{\max \left(\Pr \left(Y=1,|,D=0,U=1\right),\Pr \left(Y=1,|,D=0,U=0\right)\right)}{\min \left(\Pr \left(Y=1,|,D=0,U=1\right),\Pr \left(Y=1,|,D=0,U=0\right)\right)},$$

$${{RR}}_{{UY}|D=1}\equiv \frac{\max \left(\Pr \left(Y=1,|,D=1,U=1\right),\Pr \left(Y=1,|,D=1,U=0\right)\right)}{\min \left(\Pr \left(Y=1,|,D=1,U=1\right),\Pr \left(Y=1,|,D=1,U=0\right)\right)},$$

and

$${{RR}}_{{UY}}\equiv \max \left({{RR}}_{{UY}|D=1},{{RR}}_{{UY}|D=0}\right).$$

*RR*_*UY*_ thus represents the maximum relative prevalence of the outcome across levels of the unmeasured confounder within levels of the exposure. E-value can then be interpreted as the minimum value that *RR*_*UY*_ and *RR*_*DU*_ would have to both equal in order to explain away the association between *Y* and *D*. In the implementation of this approach with covariates, all of the parameters and definitions above are conditional on the measured set of covariates used in the analysis.

VanderWeele and Ding [67] describe the E-value as “the minimum strength of association, on the risk ratio scale, that an unmeasured confounder would need to have with both the treatment and outcome to fully explain away a specific treatment--outcome association.” The formal definition of the E-value highlights two subtleties. First, the outcome–confounder risk ratio quantifies the “effect” of the confounder on the outcome, which accords with how we conceptualize confounding. In contrast, the exposure–confounder risk ratio quantifies the “association between the exposure to the confounder, which departs from the usual understanding of confounding and may be more challenging to reason about what magnitude of risk ratio is reasonable when this value is larger. Second, the outcome–confounder risk ratio is defined slightly differently from how risk ratios are typically defined. In particular, *RR*_*UY*_ is the maximum ratio of outcome prevalences we can obtain when comparing two levels of *U* within levels of the exposure(e.g. for a binary exposure, the higher of the risk ratio for *U* when the exposure is present or absecnt). The confounder-outcome relationship is conditional on the exposure—which may take on many values in our setting—leading to even greater complexity with regard to what magnitude of E-value is “large” in any given context.

To facilitate interpreting the magnitude of E-values, we have developed an approach to calibrating—or benchmarking—the E-value against a known, observed confounder, using the definition of effects above. The calibrated values provide a sense of what magnitude of risk ratios is plausible for a given exposure–outcome combination. A natural first choice for such an observed confounder is gender, as it is related to many aspects of well-being. For simplicity, and because 99.6% of participants choose either “male” or “female,” we treat gender as binary for this analysis. The following steps can then be used to establish benchmark values for *RR*_*UY*_ and *RR*_*DU*_. We will use *RR*_*GY*_ and *RR*_*DG*_ to denote the E-value risk ratios for gender.

To calculate *RR*_*GY*_, we do the following:

1. Estimate the prevalence of the outcome (Y = 1) among male (G = male) among those with the exposure (D = 1).
2. Estimate the prevalence of outcome (Y = 1) among female (G = female) among those with the exposure (D = 1).
3. Compute $R{R}_{{GY|D}=1}$ as the larger of (1) and (2) divided by the smaller.
4. Estimate the prevalence of the outcome (Y = 1) among male (G = male) among those without the exposure (D = 0).
5. Estimate the prevalence of outcome (Y = 1) among female (G = female) among those without the exposure (D = 0).
6. Compute $R{R}_{{GY|D}=0}$ as the larger of (4) and (5) divided by the smaller.
7. Take the larger of $R{R}_{{GY|D}=1}$ and $R{R}_{{GY|D}=0}$ as *RR*_*GY*_.

To calculate *RR*_*DG*_, we do the following:

1. Calculate the proportion of the treated who are male, $\Pr (D=1{|G}={male})$, and calculate the proportion of the untreated who are male, $\Pr (D=0{|G}={male})$. The first proportion divided by the second gives us $R{R}_{{DG|male}}$.
2. Calculate the proportion of those with the exposure who are female, $\Pr (D=1{|G}={female})$, and calculate the proportion of those without the exposure who are female, $\Pr (D=0{|G}={female})$. The first proportion divided by the second gives us $R{R}_{{DG|female}}$.
3. Define *RR*_*DG*_ as the larger of $R{R}_{{DG|male}}$ and $R{R}_{{DG|female}}$.

The two risk-ratios, *RR*_*DG*_ and *RR*_*GY*_ provide a benchmark from for interpretating E-values. Specifically, the minimum strength of the association between gender and the exposure is *RR*_*DG*_, and between gender and the outcome is *RR*_*GY*_. If *RR*_*DG*_ or *RR*_*GY*_ exceed the E-value, and we suspect unmeasured confounders are even more strongly associated with the exposure and outcome than gender is, we should conclude the exposure–outcome association is not especially robust to unmeasured confounding. If, on the other hand, if the E-value is smaller than the two benchmarks, and we suspect the associations with gender are larger than the associations with any plausible unmeasured confounder, we should conclude that the exposure–outcome association is fairly robust to unmeasured confounding. In practice, we recommend using multiple observed covariates as benchmarks to help identify the range of plausible E-values.

In the example analysis section, we illustrate this calibration approach using the relationship between mastery and one of the composite flourishing indices. Because the outcome is a continuous variable, the above definitions are not directly applicable. Our initial suggestion is to dichotomize all variables at the top and bottom quintiles, which reduces the number of observations but provides reasonable separation between those in the top and bottom of the continuous variable distribution. Alternative dichotomization methods are possible and described in our online supplement.

We also use the Cinelli and Hazlett [70] approach, which parameterizes sensitivity in terms of *R*^2^—how much variance in the outcome and the exposure must an unmeasured confounder need to explain away the observed effect. This method was developed specifically to examine sensitivity to unmeasured confounding when the outcome is continuous and a linear outcome model is plausible.

#### Accounting for complex sampling design

Similar to the coordinated childhood predictor analyses based on Wave 1 of the GFS [27], accounting for the complex sampling design was accomplished by utilizing the information provided by Gallup on the primary sampling unit (PSU) IDs, strata IDs, and sampling weights. The weighting variable and PSU/strata IDs were included in all country-specific analyses. A complexity arises when respondents are recruited via face-to-face, because sometimes this can result in groups (strata) with a single PSU. When a stratum has only a single case, this is known as a lonely PSU and makes variance and standard error estimation more complex because traditional methods assume multiple PSUs within each stratum [71, 72]. We elected to use the ‘certainty’ specification where single-PSU stratum does not contribute to the variance. Complete details concerning the implementation of these methods to account for the complex sampling design of each country can be found in the *Rglobalflourishing* package [35].

## Example analysis – sense of mastery

We illustrate the aforementioned analytic methodology and corresponding results with an example based on Wave 1 sense of mastery predicting the 56 main outcomes of interest approximately one year later at Wave 2; see Kim et al. [73] for further details.

### Construct overview and importance

Sense of mastery—the perception that one has the ability to influence one’s environment and achieve desired outcomes [74, 75]—is important in its own right, and also because it shapes people’s trajectories of psychological, social, spiritual, behavioral, and physical health [76–80]. Sense of mastery was assessed in the GFS with the question, “*How often do you feel very capable in most things you do in life?”*; response options include: always, often, rarely, and never, which we dichotomized by collapsing always/often (coded 1) and rarely/never (coded 0).

**Fig. 3:**
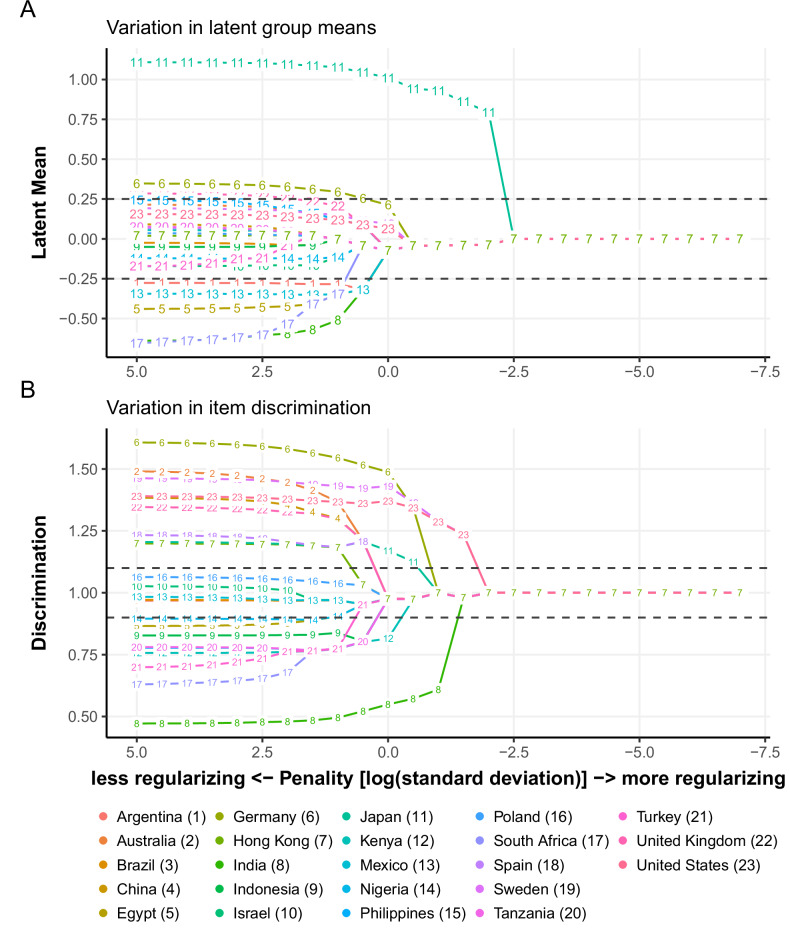
Sense of mastery differential item functioning results highlight India and Japan as potentially deviating. **A** Uniform DIF with respect to latent response variable location. **B** Non-uniform DIF with respect to latent response characteristics curve slope (discrimination parameter)

**Fig. 4:**
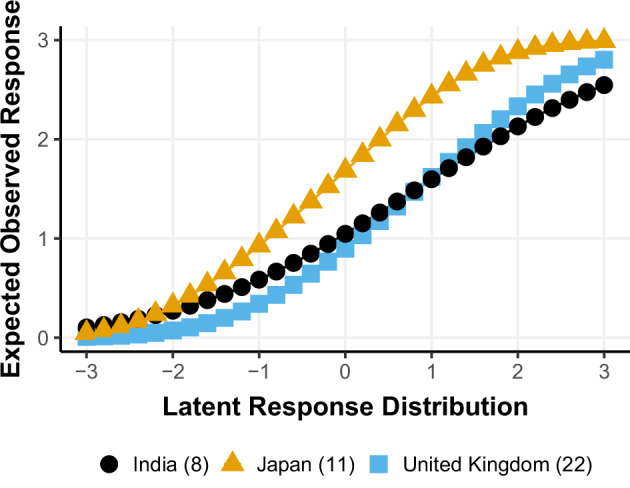
Item characteristic curves for India with non-uniform DIF, Japan with uniform DIF, and UK with no DIF

Differential item functioning is the degree to which individuals with the same level of the construct being assessed differ in their responses due to differences in the measurement model parameters. For the *Sense of mastery* predictor assessed at Wave 1, the penalized HETOP differential item functioning results can be summarized in three main plots. First, Fig. 3A shows how the differences among countries’ latent group intercepts vary as the estimation of the model increases in penalty for group differences. The major standout is Japan (group 11) in location, also known as uniform DIF. Second, Fig. 3B illustrates the differences in the item discrimination parameter. India (group 8) stands out as a country with relatively low discrimination, indicating the item does not differentiate between low and high mastery relative to the other countries, also known as non-uniform DIF. When these results are combined, we can plot the expected item response for each country using the group-specific measurement model parameters, as shown in Fig. 4 for India, Japan, and the United Kingdom, with the latter included to illustrate a country with little DIF. For the Japan sample, we found evidence of uniform DIF. This means that for individuals with the same level of the latent construct of *Sense of mastery*, respondents in Japan are more likely to have a higher response than participants from other countries. These results suggest that the analyses from the India sample need to be carefully interpreted, as differences in responses to *Sense of mastery* may be less indicative of differences in the underlying construct of *Sense of mastery* compared to other countries included in the GFS. A potential downstream effect of the lower discrimination of *Sense of mastery* in India is that effect sizes may be attenuated towards zero in this sample and, when combined with the other countries, could downwardly bias estimates of association.

### Illustrative results

Table 3 provides the outcome-wide meta-analytic results of the associations of mastery on subsequent well-being and other outcomes. A similar table is presented in other manuscripts that make use of this coordinated analytic framework to examine associations for other focal Wave 1 variables. The results provide some evidence that the pooled associations of mastery with subsequent outcomes tended to be stronger for the composite flourishing indices and other psychological well‑being outcomes. Character/prosocial traits and social well‑being/distress indicators showed more modest associations. Physical health and health behavior outcomes, and most socioeconomic indicators, showed smaller but generally favorable associations. Otherwise, associations with social participation factors, some health behaviors, and other relatively slowly changing structural statuses (e.g., educational attainment, current employment) were essentially null. When only controlling for demographic variables and childhood characteristic variables (Model 1), the associations were generally stronger than those from Model 2, which additionally adjusts for all contemporaneously assessed confounders (Model 2) using seven principal components. These patterns suggest that Model 2 might be over-controlling for potential mediators, thereby attenuating the estimated associations. However, we caution against overinterpreting the results from Model 1 because the set of controls may not be sufficient. The evidence provided by Models 1 and 2 of the potential associations between the focal exposure and outcomes at Wave 2 should be balanced according to what association is being interpreted, as the set of controls may be more or less sufficient to control confounding.

**Table 3:** Meta-analyzed associations of mastery at Wave 1 with well-being and other outcomes at Wave 2

Outcome	Model 1: Demographic and Childhood Variables as Covariates					Model 2: Demographic, Childhood, and Other Wave 1 Confounding Variables (Via Principal Components) as Covariates				
	RR	ES	95% CI	τ	Global *p*-value	RR	ES	95% CI	τ	Global *p*-value
*Human Flourishing*										
Secure flourishing index		0.20	(0.16,0.24)	0.09	3.78e–16***		0.04	(0.03,0.05)	0.02	5.11e–15***
Flourishing index		0.20	(0.17,0.24)	0.09	3.88e–16***		0.04	(0.03,0.05)	0.02	4.49e–13***
*Psychological Well-Being*										
Happiness		0.13	(0.10,0.17)	0.08	8.51e–16***		0.01	(0.01,0.02)	<0.01†	0.337
Life satisfaction		0.13	(0.10,0.17)	0.09	8.47e–16***		0.02	(0.01,0.03)	0.01	9.13e–04**
Current life evaluation		0.12	(0.09,0.15)	0.07	1.28e–15***		0.03	(0.02,0.03)	<0.01†	6.64e–04***
Future life evaluation		0.12	(0.09,0.15)	0.07	1.28e–15***		0.04	(0.03,0.04)	0.01	3.93e–07***
Optimism		0.13	(0.10,0.17)	0.08	1.01e–15***		0.04	(0.03,0.04)	<0.01†	7.82e–07***
Freedom to pursue what’s important		0.14	(0.11,0.17)	0.07	1.02e–15***		0.04	(0.03,0.05)	0.01	1.7e–06***
Inner peace	1.10		(1.07,1.14)	0.07	1.02e–15***	1.05		(1.03,1.06)	0.03	5.37e–12***
Life balance	1.13		(1.09,1.16)	0.08	1.13e–15***	1.06		(1.04,1.07)	0.03	6.54e–12***
Sense of mastery	1.16		(1.11,1.22)	0.14	6.38e–16***	1.13		(1.08,1.17)	0.10	1.02e–15***
Meaningful activities		0.16	(0.12,0.20)	0.09	9.81e–16***		0.05	(0.04,0.06)	0.02	5.35e–12***
Understanding purpose		0.15	(0.12,0.19)	0.08	8.37e–16***		0.04	(0.03,0.06)	0.02	1.28e–11***
Self-rated mental health		0.14	(0.11,0.18)	0.08	7.29e–16***		0.03	(0.02,0.04)	0.02	8.38e–04***
*Psychological Distress*										
Traumatic distress	0.95		(0.93,0.97)	0.04	1.75e–12***	1.01		(1.00,1.01)	<0.01†	0.450
Depression symptoms composite	0.88		(0.84,0.92)	0.09	1.02e–15***	0.99		(0.98,1.00)	0.01	3.81e–03**
Anxiety symptoms composite	0.88		(0.84,0.92)	0.09	1.2e–15***	1.00		(0.98,1.01)	0.02	0.114
Suffering	0.94		(0.91,0.96)	0.05	1.7e–15***	1.01		(1.00,1.02)	0.01	4.06e–03**
*Social Well-Being*										
Relationship contentment		0.13	(0.10,0.16)	0.07	1.09e–15***		0.01	(0.00,0.02)	<0.01†	0.133
Relationship satisfaction		0.13	(0.10,0.16)	0.06	1.02e–15***		0.01	(0.00,0.02)	<0.01†	0.451
Social support		0.09	(0.07,0.12)	0.05	1.68e–15***		0.01	(−0.00,0.02)	<0.01†	0.144
Intimate/close friend	1.03		(1.02,1.04)	0.02	5.11e–15***	1.00		(1.00,1.01)	<0.01†	0.250
Government approval	1.04		(1.02,1.06)	0.04	3.56e–10***	0.99		(0.99,1.00)	<0.01†	0.742
Say in government	1.05		(1.03,1.08)	0.05	4.88e–15***	1.00		(0.99,1.02)	0.02	0.017*
Belonging in country		0.10	(0.08,0.12)	0.04	1.62e–15***		0.02	(0.01,0.03)	<0.01†	0.038*
City/place satisfaction	1.04		(1.03,1.06)	0.03	2.5e–15***	1.01		(1.00,1.02)	<0.01†	0.030*
Trust within country	1.04		(1.02,1.05)	0.02	5.11e–15***	1.01		(1.00,1.01)	<0.01†	0.627
*Social Participation*										
Ever been married	1.00		(1.00,1.01)	<0.01†	0.521	1.00		(1.00,1.00)	<0.01†	0.944
Currently divorced	1.00		(0.98,1.02)	<0.01†	0.962	1.01		(0.99,1.03)	<0.01†	0.962
Number of children		−0.00	(−0.01,0.01)	0.01	0.151		−0.00	(−0.01,0.01)	0.01	0.140
Weekly + community participation	1.06		(1.03,1.10)	0.06	3.09e–11***	1.02		(1.00,1.04)	<0.01†	0.095
Weekly + religious attendance	1.01		(1.00,1.02)	<0.01†	0.145	1.00		(0.99,1.01)	<0.01†	0.898
*Social Distress*										
Loneliness		−0.11	(−0.13,−0.08)	0.06	1.27e–15***		0.00	(−0.01,0.01)	<0.01†	0.430
Perceived discrimination	0.98		(0.95,1.00)	0.05	2.99e–05***	1.03		(1.01,1.04)	<0.01†	0.082
*Character & Prosocial Behavior*										
Orientation to promote good		0.13	(0.10,0.15)	0.06	1.22e–15***		0.04	(0.04,0.05)	<0.01†	3.37e–13***
Delayed gratification		0.10	(0.07,0.12)	0.05	1.28e–15***		0.03	(0.02,0.04)	0.02	1.56e–05***
Hope		0.16	(0.12,0.19)	0.08	1.01e–15***		0.05	(0.04,0.05)	<0.01†	1.17e–06***
Gratitude		0.12	(0.09,0.15)	0.07	1.02e–15***		0.01	(0.01,0.02)	<0.01†	0.287
Showing love/care		0.10	(0.08,0.13)	0.05	1.7e–15***		0.02	(0.01,0.03)	0.02	0.010*
Forgivingness	1.03		(1.02,1.04)	0.02	5.11e–15***	1.01		(1.00,1.02)	<0.01†	0.015*
Charitable giving	1.03		(1.02,1.04)	0.02	3.61e–04***	1.00		(0.98,1.01)	0.02	0.220
Helping strangers	1.04		(1.03,1.06)	0.03	4.01e–07***	1.02		(1.01,1.02)	<0.01†	0.051
Volunteering	1.06		(1.03,1.09)	0.05	3.26e–06***	1.02		(1.01,1.03)	<0.01†	0.500
*Physical Health & Health Behavior*										
Self-rated physical health		0.12	(0.09,0.15)	0.07	1.02e–15***		0.03	(0.02,0.04)	0.02	7.2e–09***
Health problems	0.92		(0.90,0.94)	0.05	2.55e–15***	0.99		(0.98,1.01)	0.02	0.088
Pain in past 4 weeks	0.95		(0.94,0.97)	0.03	4.54e–15***	1.01		(1.00,1.02)	0.01	0.012*
Daily smoker	0.99		(0.97,1.01)	0.03	0.020*	1.02		(1.01,1.04)	<0.01†	0.019*
Number of drinks per week		0.01	(−0.00,0.01)	<0.01†	0.198		0.01	(0.00,0.02)	<0.01†	0.827
Days exercise per week		0.06	(0.04,0.07)	0.02	4.71e–15***		0.02	(0.01,0.03)	0.01	4.05e–04***
*Socioeconomic Outcomes*										
Financial security		0.09	(0.07,0.11)	0.04	1.7e–15***		0.02	(0.01,0.03)	<0.01†	1.12e–06***
Material security		0.09	(0.07,0.11)	0.05	1.7e–15***		0.02	(0.01,0.03)	0.01	1.71e–03**
Educational attainment (16 + years)	1.00		(1.00,1.00)	<0.01†	0.911	1.00		(1.00,1.00)	<0.01†	0.943
Currently employed	1.01		(1.00,1.01)	<0.01†	0.210	1.01		(1.00,1.01)	<0.01†	0.707
Financially comfortable/getting by	1.05		(1.04,1.06)	0.01	1.7e–15***	1.01		(1.01,1.02)	<0.01†	4.43e–03**
Own home	1.01		(1.01,1.02)	<0.01†	1.92e–03**	1.00		(1.00,1.01)	0.01	0.036*
Income -- top quintile	1.01		(1.01,1.01)	<0.01†	0.010*	1.00		(1.00,1.01)	<0.01†	0.662

*N* = 207,919; Reference for focal exposure: responded rarely/never; *RR* risk-ratio, null effect is 1.00, *ES* effect size measure for standardized regression coefficient, null effect is 0.00, *CI* confidence interval, *τ* (tau, heterogeneity), estimated standard deviation of the distribution of effects; Global *p*-value, joint test of the null hypothesis that the country-specific Wald tests are null in all countries

Multiple imputation was performed to impute missing data on the covariates, exposure, and outcomes. All models controlled for sociodemographic and childhood factors assessed at Wave 1: relationship with mother growing up; relationship with father growing up; parent marital status around age 12; experienced abuse growing up (except for Israel); felt like an outsider in family growing up; self-rated health growing up; subjective financial status growing up; religious affiliation at age 12; frequency of religious service attendance around age 12; year of birth; gender; education, employment status, marital status, immigration status; and racial/ethnic identity when available. For Model 2 with PC (principal components), the first seven principal components of the entire set of contemporaneous confounders assessed at Wave 1 were included as additional covariates of the outcomes at Wave 2

An outcome-wide analytic approach was used, and a separate model was run for each outcome. A different type of model was run depending on the nature of the outcome: (1) for each binary outcome, a weighted generalized linear model (with a log link and Poisson distribution) was used to estimate a RR; and (2) for each continuous outcome, a weighted linear regression model was used to estimate an ES. All effect sizes were standardized. For continuous outcomes, the ES represents the change in SD on the outcome between the lower and upper categories of the binary focal exposure. For binary outcomes, the RR represents the change in risk of being in the upper category compared to the lower category between the lower and upper categories of the binary focal exposure

*p*-value significance thresholds: *p* < 0.05*, *p* < 0.005**, (Bonferroni) *p* < 0.00089***, correction for multiple testing to significant threshold; † Estimate of τ (tau, heterogeneity) is likely unstable. Line-printer style abbreviations for small *p*-values (e.g., “2.22e–16”) are used to help conserve space, given the table and font size, to aid in readability

### Sensitivity to unreliability

The sensitivity to unreliability in the single-item measures is assessed using the assumed-known average reliability of each variable to correct for attenuation of association estimates. For ease of presentation, we only report on a subset of outcomes here (Table 4) with the full set of results provided in our Supplemental material 1: Table S5. The standardized effect of mastery on subsequent Secure Flourishing Index scores could be as high as 0.502 (95% CI: 0.466, 0.539) if the average reliability of Sense of Mastery and Flourishing is 0.40 or lower, which is substantially higher than if we assumed perfect reliability (est = 0.201, 95% CI: 0.164, 0.237). The associations between Sense of Mastery and being a Daily Smoker and Number of drinks per week are null on average according to our primary results, but if the average reliability is less than 0.55 for smoking and 0.70 or less for drinking, then the associations will be non-zero on average.

**Table 4:** Reliability corrected meta-analyzed estimates of association, Estimate (95% CI)

Outcome	Primary Result	Reliability corrected estimates		
		0.40	0.55	0.7
*Human Flourishing*				
Secure flourishing index	0.201 (0.164, 0.237)	0.502 (0.466, 0.539)	0.365 (0.329, 0.402)	0.287 (0.250, 0.323)
Flourishing index	0.204 (0.165, 0.243)	0.510 (0.471, 0.548)	0.371 (0.332, 0.409)	0.291 (0.253, 0.330)
*Physical Health & Health Behavior*				
Daily smoker	−0.010 (−0.030, 0.010)	−0.025 (−0.045, −0.006)	−0.018 (−0.038, 0.001)	−0.014 (−0.034, 0.005)
Number of drink per week	0.006 (−0.002, 0.013)	0.015 (0.007, 0.022)	0.011 (0.003, 0.018)	0.008 (0.001, 0.016)

All estimates are reported without transforming. So for binary outcomes, this means the effect sizes are on the log-risk-ratio scale. The reliability corrected point estimate and standard errors are computed by dividing the primary result value by reliability (0.40, 0.55, and 0.70)

### Sensitivity to unmeasured confounding

The sensitivity to unmeasured confounding is assessed using the E-value [67]. Table 5 provides the results of this primary sensitivity analysis, and details the sensitivity of the association between sense of mastery and each subsequent outcome to unmeasured confounding. The E-values generally range from 1.0 to 1.70 for the effect estimate and range from 1.0 to 1.60 for the confidence limit. Based on Model 1, the effect of mastery at Wave 1 on the secure flourishing index at Wave 2 has an E-value estimate of 1.69, meaning that an unmeasured confounder would need a risk ratio of 1.69 or greater with both mastery and this outcome to explain away the observed association. Model 1 includes a variety of demographic characteristics and childhood experiences that encompass a wide range of possible confounders, but covariate control is more limited so there may be many potential unmeasured confounders. One such confounder that might be strongly associated with mastery and the secure flourishing index is the personality trait of conscientiousness, which could reasonably have a strong association (risk ratio) of 1.70 or greater, and thus could explain away the association observed in Model 1.

**Table 5:** E-value sensitivity analysis for unmeasured confounding for the association between mastery and subsequent well-being and other outcomes

Outcome	Model 1: Demographic and Childhood Variables as Covariates		Model 2: Demographic, Childhood, and Other Wave 1 Confounding Variables (Via Principal Components) as Covariates		Calibrated E-value (RR: Gender - Sense of mastery = 1.11)
	E-value	E-value for CI	E-value	E-value for CI	RR: Gender - Outcome
*Human Flourishing*					
Secure flourishing index	1.69	1.59	1.24	1.21	1.20
Flourishing index	1.70	1.60	1.25	1.21	1.30
*Psychological Well-Being*					
Happiness	1.51	1.41	1.12	1.08	1.32
Life satisfaction	1.51	1.41	1.17	1.12	1.29
Current life evaluation	1.48	1.40	1.18	1.14	1.27
Future life evaluation	1.47	1.39	1.22	1.18	1.32
Optimism	1.51	1.42	1.22	1.18	1.23
Freedom to pursue what’s important	1.52	1.44	1.22	1.18	1.20
Inner peace	1.44	1.35	1.27	1.22	1.03
Life balance	1.50	1.41	1.30	1.26	1.04
Sense of mastery	1.60	1.45	1.50	1.39	1.02
Meaningful activities	1.58	1.48	1.25	1.22	1.28
Understanding purpose	1.56	1.48	1.25	1.21	1.14
Self-rated mental health	1.54	1.44	1.18	1.13	1.09
*Psychological Distress*					
Traumatic distress	1.29	1.22	1.08	1.00	1.21
Depression symptoms composite	1.54	1.41	1.10	1.00	1.16
Anxiety symptoms composite	1.53	1.41	1.06	1.00	1.29
Suffering	1.34	1.26	1.10	1.00	1.14
*Social Well-Being*					
Relationship contentment	1.50	1.42	1.12	1.06	1.26
Relationship satisfaction	1.49	1.42	1.11	1.06	1.23
Social support	1.40	1.34	1.09	1.00	1.37
Intimate/close friend	1.20	1.16	1.05	1.00	1.12
Government approval	1.24	1.16	1.08	1.00	1.03
Say in government	1.29	1.20	1.05	1.00	1.02
Belonging in country	1.42	1.36	1.15	1.11	1.11
City/place satisfaction	1.26	1.21	1.10	1.05	1.04
Trust within country	1.23	1.18	1.08	1.01	1.04
*Social Participation*					
Ever been married	1.06	1.03	1.05	1.00	1.07
Currently divorced	1.03	1.00	1.10	1.00	1.54
Number of children	1.03	1.00	1.01	1.00	1.23
Weekly + community participation	1.32	1.21	1.17	1.07	1.18
Weekly + religious attendance	1.10	1.03	1.04	1.00	1.18
*Social Distress*					
Loneliness	1.44	1.37	1.03	1.00	1.06
Perceived discrimination	1.18	1.00	1.19	1.13	1.05
*Character & Prosocial Behavior*					
Orientation to promote good	1.49	1.42	1.25	1.22	1.21
Delayed gratification	1.41	1.34	1.19	1.15	1.21
Hope	1.57	1.48	1.25	1.23	1.26
Gratitude	1.47	1.39	1.13	1.08	1.39
Showing love/care	1.43	1.36	1.16	1.09	1.41
Forgivingness	1.21	1.18	1.10	1.05	1.06
Charitable giving	1.21	1.15	1.03	1.00	1.01
Helping strangers	1.25	1.19	1.15	1.12	1.07
Volunteering	1.31	1.21	1.16	1.08	1.06
*Physical Health & Health Behavior*					
Self-rated physical health	1.48	1.40	1.20	1.15	1.07
Health problems	1.39	1.31	1.09	1.00	1.14
Pain in past 4 weeks	1.28	1.23	1.09	1.00	1.15
Daily smoker	1.11	1.00	1.17	1.09	1.00
Number of drinks per week	1.08	1.00	1.11	1.05	2.10
Days exercise per week	1.28	1.25	1.16	1.12	1.33
*Socioeconomic Outcomes*					
Financial security	1.38	1.32	1.15	1.11	1.12
Material security	1.38	1.32	1.15	1.11	1.12
Educational attainment (16 + years)	1.00	1.00	1.00	1.00	1.05
Currently employed	1.10	1.06	1.08	1.03	1.42
Financially comfortable/getting by	1.28	1.25	1.13	1.10	1.07
Own home	1.14	1.10	1.08	1.00	1.05

*N* = 207,919; The formula for calculating E-values can be found in VanderWeele and Ding (2017) [54]. E-values for estimate are the minimum strength of association on the risk ratio scale that an unmeasured confounder would need to have with both the exposure and the outcome to fully explain away the observed association between the exposure and outcome, conditional on the measured covariates. E-values for the 95% CI closest to the null denote the minimum strength of association on the risk ratio scale that an unmeasured confounder would need to have with both the exposure and the outcome to shift the CI to include the null value, conditional on the measured covariates

A complication with the above results for the sensitivity of results to unmeasured confounding is the somewhat arbitrary nature of the magnitude of the strength of effect the hypothesized unmeasured confounder (e.g., conscientiousness) must have to explain away the association. Using the calibration approach outlined in the *Sensitivity to unmeasured confounded* section, we find that the measured confounder of gender has a risk ratio of 1.1 with mastery and 1.20 with the secure flourishing index. To obtain the risk ratio with flourishing, we created a binary secure flourishing index variable using the top and bottom quintiles of the distribution of scores on the secure flourishing index. For the result of Model 1 to be reduced to the null, we would have to believe that an unmeasured confounder, such as conscientiousness, exists with a stronger association than gender (conditional on the measured confounders), with a risk ratio of 1.20. The calibrated values of the E-value are reported for every outcome reported on in Table 5. Additional calibrated values are shown in Supplemental material 1: Table S3 using age, race, and other observed confounders.

**Fig. 5:**
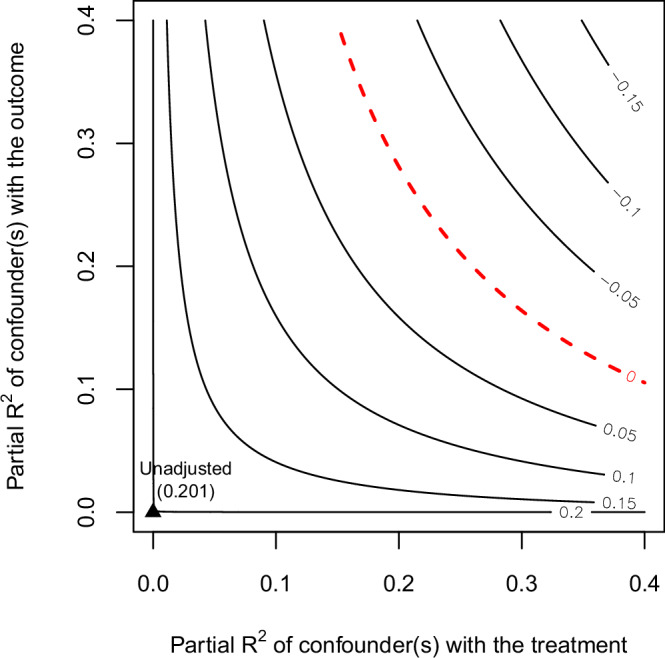
Sensitivity of effect of mastery (Wave 1) on the secure flourishing index (Wave 2) using Cincelli and Hazlett approach. Plot made using the sensemakr package [70] package in R using the meta-analyzed effect estimates reported in Table 3 for the effect of mastery on the secure flourishing index

Another complication with the sensitivity analyses is that thinking of confounders solely in terms of risk ratios is not always intuitive, especially for continuous variables; hence, using calibration to benchmark the magnitude of the E-value can be helpful. However, an alternative approach to sensitivity analysis for continuous outcomes is based on an R^2^-like approach so that unmeasured confounding can be conceptualized on a proportion of variance explained metric [70]. The contour plot of the sensitivity analysis is shown in Fig. 5.

### Sensitivity of results to analytic decisions

The analyses described as part of this coordinated effort involve many different analytic choices, each of which could lead to varying results. We explore how a few key analytic decisions might influence the results. We use a continuous outcome (secure flourishing index) and a binary outcome (depression symptoms) to probe the sensitivity of the results to analytic decisions.

**Fig. 6:**
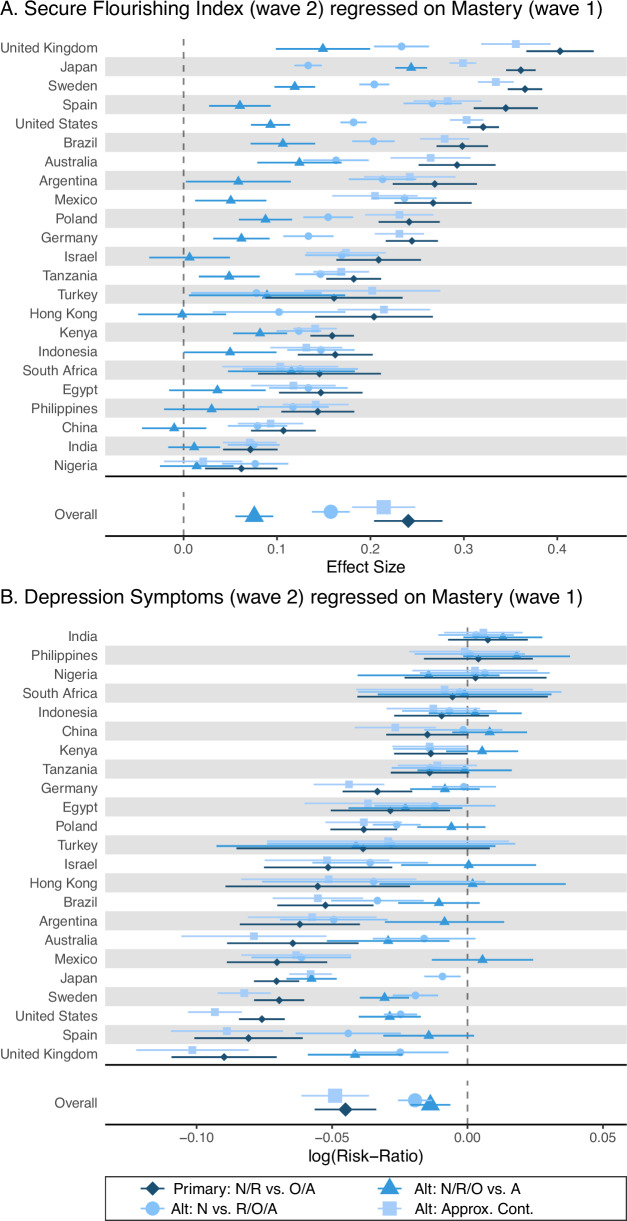
Sensitivity of regression analyses to choice of response coding of mastery. **A** Forest plot of association estimates between master (wave 1) and the secure flourishing index (wave 2). **B** Forest plot of the association estimates between mastery (wave 1) and depression symptoms (wave 2). Response options: N Never, R Rarely, O Often, A Always, Approx. approximately

One potential source of variation in results is how the focal exposure is coded for the analysis. Analysts using the GFS data have several coding choices due to the discrete nature of some variables (such as mastery). The default approach is for each team to dichotomize Likert-type responses into a binary variable whose operationalization is informed by subject-area expertise, which aligns with how analyses were conducted for Wave 1 and facilitates comparability of results across waves. The preregistered coding specified that mastery would be dichotomized such that never/rarely would be collapsed to represent the reference category (0) with often/always as the comparison category (1). This cut-off point was chosen before examining any data, because it reflects a conceptual distinction where individuals who consistently feel capable (“always/often”) are separated from those who have a pronounced deficit (“rarely/never”). Alternatively, one could (a) dichotomize as never verses any positive frequency (rarely/often/always); (b) dichotomize using a “not-always” approach where never, rarely, and often are treated as reference relative to always; and (c) treat the four ordered response categories as approximately continuous. The sensitivity of the results of regressing the secure flourishing index and depressive symptoms on sense of mastery based on these alternative mastery coding is shown in Fig. 6A and B. To evaluate if the main findings hinge on the exact cutoff point chosen, many research teams in the coordinated effort conducted such a sensitivity analysis, where the focal exposure is analyzed with an alternate dichotomization point.

**Fig. 7:**
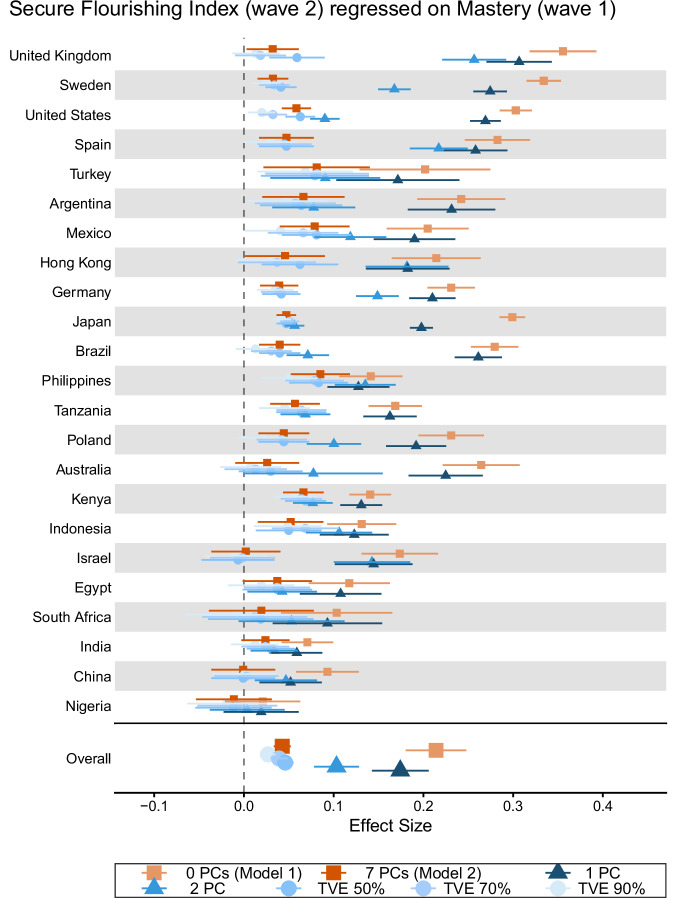
Sensitivity of results to the number of principal components included in the analysis. Countries are ordered by the average effect size across models tested. PC, principal components; TVE, total variance explained; the square points represent the results for the two focal analyses (Model 1 and Model 2); the triangular points (models with 1PC or 2 PC) use a fixed number of principal components across all countries; the circular points represent models estimated with a fixed minimum proportion of variability explained in the set of confounders ranging from 50, 70, and 90% so that the number of components included within any particular country meets this requirement

Another potential source of variation in results is how we select the number of principal components to include. For the coordinated analyses, we have preregistered using the first seven principal components, which account for between 41–58% of the variability in the set of contemporaneously assessed potential confounders across countries. However, it should be considered how this decision may influence the results. One might reasonably say that only one or two principal components are needed to sufficiently “control” for the information contained in the set of contemporaneously assessed confounders. Or, another reasonable alternative is to say that the number of principal components should vary across countries so that a minimum proportion of variation is explained. We provide the results for each of these possibilities for the association between mastery and the Secure Flourishing Index in Fig. 7. The result highlights how using a single principal component yields estimates nearly identical to Model 1, though slightly attenuated. The choice of seven principal components is nearly identical to varying the number of components included by country to achieve a minimum proportion of variance explained. Similar conclusions were drawn when depression symptoms were examined as the outcome (see Supplemental material 1: Figure S3).

## Discussions

The analytic methodology employed for this coordinated set of studies has several major strengths. First, the GFS includes approximately nationally representative samples from 22 diverse countries and one territory, collectively representing approximately two-thirds of the world’s population [5]. This broad population coverage, combined with the breadth of constructs assessed, provides a unique opportunity to investigate patterns and determinants of well-being across diverse cultural, economic, and social contexts. All analyses were conducted at the country-level, and the results were then pooled using meta-analytic techniques to account for uncertainty in the estimates and quantify heterogeneity across countries. This approach facilitates an understanding of both cross-national and country-specific patterns, which helps inform cross-national and localized policies or initiatives to enhance population well-being.

Second, these studies use an outcome-wide analytic approach. This approach provides a holistic view of the focal exposure’s association with multiple subsequent outcomes simultaneously, which facilitates a deeper understanding of the broad and multifaceted impacts of a focal exposure. The standardized analytic and reporting plan also helps reduce researcher subjectivity in covariate selection and mitigates concerns about publication bias [81].

Third, the GFS data are openly available through the Center for Open Science, and all code needed to reproduce this coordinated set of analyses are openly available through the *Rglobalflourishing* package [35]. All substantive papers following this coordinate set of analyses are each preregistered with the COS. This approach enhances transparency and supports the broader research community to explore these data and reproduce results.

Our approach also has several limitations. First, with observational data we cannot entirely rule out the presence of unmeasured confounding, although sensitivity analyses can help us assess how severe the resulting bias might need to be to alter conclusions.

Second, while the use of a wide range of potential confounders in Wave 1 provides some confidence in the resulting estimates of association, using only two waves of data cannot provide sufficient control for confounding to yield causal estimates. Two waves of data cannot distinguish between stable within-person changes in scores and between-person differences, thus making causal inference difficult or impossible. Future waves of data collection will provide the opportunity to explore potential causal relationships.

Third, while our choice to estimate two primary sets of models that included a smaller (Model 1) and larger (Model 2) set of covariates helps with gauging how effect estimates might shift under more or less conservative confounding control conditions, neither offers an optimal solution. Results for Model 1 could be biased away from the null because the set of covariates may not provide adequate confounding control, whereas results from Model 2 could be biased toward the null because if one or more contemporaneously measured covariates are causally downstream of the focal exposure then conditioning on these covariates may block causal pathways between the focal exposure and outcome and may potentially induce “collider bias” in the presence of unmeasured confounding between the post-exposure variable and the outcome [82, 83]. These limitations can make it difficult to reason about the overall direction of bias.

Fourth, virtually all variables are measured with some degree of error. Without knowledge about the nature of this measurement error, it is again difficult to reason about the direction of bias in these results due to possible measurement bias. However, non-differential measurement error tends to attenuate estimates of association [84]. Part of this error stems from the inability to rule out DIF in the single-item assessments of each construct across countries. While the invariance analyses of the Secure Flourishing Index [30] and the PHQ-4 (see section *Measurement invariance testing*) in the GFS provide some evidence of invariance for these composite measures, we are not aware of a suitable approach to statistically rule out measurement non-invariance of the single-item assessments. The novel single-item measure DIF test that was employed provides a rough evaluation of uniform and non-uniform DIF for single-item assessments, but it is not a traditional DIF evaluation, and the statistical properties of the model for testing DIF are currently unknown. Future work may find alternatives that can more rigorously evaluate DIF in the context of single-item assessments, but this remains a significant limitation of the present outcome-wide analyses. A consequence of DIF is that cross-national comparisons of associations are not precise and may be potentially biased. Our approach to mitigating such issues is to focus on the heterogeneity of associations across countries, providing a sense of which associations might be more stable. The added sensitivity analyses of the effects of reliability on estimates provide an additional layer for exploring how measurement may be impacting the results. In our Sense of Mastery example, several associations would be non-null if the reliability of the single-item measures were moderate (~0.55), which may indicate that our results are influenced by their unreliability. Other studies using the coordinated methodology may encounter similar cases.

Fifth, in multiwave analyses, longitudinal measurement invariance becomes a concern. If there is drift in the measurement properties of these items over time, especially if that drift is not equal across countries, it may lead to biased estimates of association and heterogeneity. The bias would conflate measure drift with actual change in the construct(s) of interest.

Sixth, our outcome-wide analysis ignores correlations between outcomes, which could be used to slightly reduce the variance of estimated associations (potentially at the cost of increased bias) [25].

Seventh, attrition is relatively high in some countries, potentially inducing bias. We attempt to address these with both multiple imputation and attrition weights. However, both multiple-imputation and attrition weighting may produce biased association estimates in the presence of unmeasured confounding between missingness and the outcome. Nevertheless, these methods themselves rely on somewhat different assumptions and thus constitute partial sensitivity analyses to each other [43]. The differential attrition across countries (almost 80% in Hong Kong to only 10% in China) may also be related to unmeasured country-level factors. Estimates from countries with higher missingness are more prone to potential bias introduced by imputation if the observed set of variables is insufficient to approximate the missing at random assumption. While the semi-complete-case attrition-weighted analyses are limited to participants who participate in all waves, the validity of the analyses still relies on the assumption that the observed variables are sufficient to approximate the non-participation mechanism. Both methods have their limitations, but together they help determine whether attrition significantly affects the interpretation of the results.

### Extending to a wave 3 outcome-wide design

**Fig. 8:**
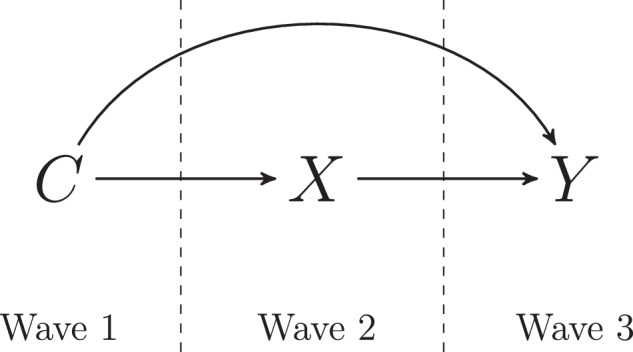
Analysis diagram for 3 Waves of data in the GFS longitudinal analyses, providing temporal separation between controls (C), focal exposure (X), and outcomes (Y)

The outcome-wide design for analyzing the combined Wave 1 and Wave 2 data of the GFS has several strengths and weaknesses as discussed above. One of the major limitations (weakness #2 above) is that we likely have either insufficient confounding control or over-control because the potential confounders were contemporaneously measured with the focal exposure variables in Wave 1 of the GFS. Once Wave 3 of the GFS data is released, this limitation can be significantly overcome using the expanded longitudinal design available. In these analyses, the structure changes so that we can employ a focal exposure measured at Wave 2 with the outcome at Wave 3 using the potential confounders measured at Wave 1, see Fig. 8. The analyses are functionally identical to those described for the Wave 2 outcome-wide analyses discussed throughout this paper, but with the major change that outcomes are assessed at Wave 3, with the focal exposure measured as Wave 2. This change ensures the pre-treatment confounding variables are temporally precedent to the exposure, thus avoiding controlling for potential mediators and avoiding potential collider bias when analyzing only two waves of data. This three-way design allows for control for the extensive set of covariates in a more straightforward way. Controlling for Wave 1 values of the outcomes assessed at Wave 3 also helps mitigate the possibility of reverse causation in evaluating the relationship between the focal exposure and the outcomes [85].

The expanded design, while functionally similar to the example analysis with Sense of Mastery, will have the added value of providing a more rigorous evaluation of the potential causal effects of each focal exposure at Wave 2 on outcomes assessed at Wave 3. The extended analyses have significant substantive advantages due to the design, but the analyses still have limitations stemming from the reliance on self-report data. The other potential limiting factor to these new analyses is the length of time between waves of data collection. For time-varying confounders, using the measured value at Wave 1 may not be sufficient for confounding control if the value has changed meaningfully in, say, the six to nine months following Wave 1 prior to Wave 2 data collection. This potentially biases the estimated effects due to inaccurate control for time-varying confounding. However, this still arguably constitutes a substantial advance over having to control for potential confounders contemporaneously. Additionally, for analyses with subsequent waves of data that use a single exposure from Wave 2 and covariates from Wave 1 but employ multiple waves of subsequent outcome data (e.g., repeated measures of outcomes from Wave 3, 4, etc.), a similar approach to confounding control could be used as described here, but with the outcome model altered [25.

Missing data and attrition will still be problematic, especially in countries with higher attrition rates, but data collection has emphasized trying to obtain participation from all respondents from Wave 1. This leads to a non-monotonic missingness pattern across waves with, for example, some respondents participating in Wave 1, not in Wave 2, and again in Wave 3. The multiple imputation approach described in this paper (see section on *Multiple imputation*) can handle such missingness patterns and gives consistent and unbiased estimates when the imputation model is correctly specified. The degree of misspecification is a determining factor of the amount of potential bias introduced. Similar to the Wave 2 analyses, we plan to conduct a sensitivity analysis by using a complete-case analysis with attrition weights using the subsample of respondents who participated in all waves of data collection. The combination of analyses and approaches to handling missing data provides a more rigorous assessment of the potential causal effects and the heterogeneity of those effects across countries.

## Conclusions

The current article provides a description of the methods used in manuscripts reporting on longitudinal outcome-wide analyses that leverage currently available Wave 1 and 2 data from the GFS, intended as a coordinated set of manuscripts providing detailed results across a wide range of focal exposures. Building upon an initial set of coordinated analyses that documented the distribution, sociodemographic correlates, and retrospectively recalled childhood predictors of various indicators of flourishing using Wave 1 GFS data (https://www.nature.com/collections/eaeicjffaf), the current set of planned analyses provides a unique opportunity to explore predictors of numerous indicators of flourishing across 23 geographically and culturally diverse contexts that represent roughly two-thirds of the global population. While there are limitations and caveats to consider, the collective findings that emerge from this coordinated effort could play an important role in shaping policies and practices oriented toward the promotion of flourishing at national and international levels.

## Supplementary information

Supplementary material 1 Contains the additional comments and technical details, and the following tables and figures: Table S1 Summary of top 20 eigenvalues from principal components; Table S2 Summary of top 20 principal components percent of variance explained by each component; Table S3 Benchmarking across a range of observed potential confounders to calibrate E-values for sensitivity analysis for unmeasured confounding; Table S4 Summary of penalized HETOP evaluation of which countries for each outcomes are identified with uniform and nonuniform differential item functioning; Table S5 Reliability corrected meta-analyzed estimates of association, Estimate (95% CI); Table S6 Description of how all outcomes were coded/collapsed and analyzed. Details on every included outcome: the item wording, response options, and how coded for analysis; Figure S1 Violin plot of time (in days) between taking the Annual Survey for respondents in each country; Figure S2 Violin plot of time of years when respondents took each annual survey; and Figure S3 Sensitivity of depression symptoms results to number of included principal components

Supplementary material 2 Provides the results and analyses for traditional MG-CFA invariance testing of the PHQ-4

Supplementary material 3 Provides a technical overview and the results from the single-item DIF analyses

## Data Availability

As of April 8, 2026, the non-sensitive data collected by Gallup for Wave 1 and Wave 2 can be accessed through the Open Science Framework repository (10.17605/osf.io/c8hbk) [86]. The code used to analyze these data is openly available in a Center for Open Science repository (10.17605/osf.io/rbcmp) [35].
