# Supplementary material for: Coordinated outcome-wide analytic methodology for multi-wave analyses of the global flourishing study
Source: BMC Glob Public Health. 2026 Jun 2;4:55. doi: 10.1186/s44263-026-00287-6 (PMC13227717; doi:10.1186/s44263-026-00287-6)

Online Supplement

Coordinated Outcome-wide Analytic Methodology for Multi-Wave Analyses of the Global Flourishing Study

Important links.

- OSF Project: https://doi.org/10.17605/osf.io/rbcmp, this links to the Wave 2 Methodology OSF paper, which had the methods paper, code shared in the zip file shared to GFS team members to facilitate running the analyses. This project is also connected to the GitHub repository that stores the core *Rglobalflourishing* package code. There are tabs on the OSF project landing page labelled 'OSF Storage' and 'GitHub' to view both sets of files.
- GitHub Repository Directly: https://github.com/noah-padgett/Rglobalflourishing, this links directly to the code of the R package created to coordinate all the analyses described in this paper.

Analysis Workflow Overview Flowchart.

Flowchart and high-level summary of procedures and statistical analyses.

1. Data prep
- Drop missing data codes: "(Saw, skipped)", "(Don't Know)", "(Refused)"
- Ensure variable coding: "numeric", "factor", "ordered factor"
- *dplyr* + *forcats* packages

2. Imputation
- Separate by country
- Predictive mean matching
- 20 complete datasets
- *mice* + *future* packages

3. Attrition model
- Logistic regression to predict probability of participating (P) in Wave 2:

$$Pr(P_2 = 1 \mid C)$$

- Separate by country and imputation
- Create stabilized weights (w_i): probability of participating in Wave 2
-Multiply sample weight and stabilized attrition weight and rescale weight to sample size
- *survey* + *svyr* package (svyglm)

Set of predictors: C = [sampling weight, strata, mode of survey in Wave 1, age, gender, education, income, employment status, marital status, race/ethnicity, religious service attendance, urban/rural status of participants, personality, days of exercise, depression, loneliness, and the domains of the flourishing index--happiness, health, meaning, character, social relationships, financial security.]

4. Regress each Y on X + C (covariates)

$$Y_m = f(X,C)$$

- M separate outcomes assessed at Wave 2
  - Weighted linear regression if Y_m continuous
  - Weighted modified Poisson if Y_m binary
- Focal exposure X
- Separate by country
- Pool across imputation
- *survey* package (svyglm)

Set of covariates:
Model 1: C = [demographics + retrospective childhood variables]
Model 2: C = [demographics + retrospective childhood variables + PCA based on all M outcomes assess at Wave 1]

4a. Conduct (4) using full sample
- All participants who completed Wave 1

4b. Conduct (4) using "complete case sample"
- Participants who completed Waves 1 & 2
- Uses updated weights from (3)
- Sensitivity analysis of results to imputation model

5. Pool across countries
- Random effects meta-analysis
- Pool unstandardized and standardized regression coefficients
- Forest plots + heterogeneity estimates (Q-statistic, tau, prediction interval)
- *metafor* package

Global p-value to test joint hypothesis of all country-specific coefficients are null.
- *harmonicmeanp* package

6. Sensitivity analysis E-values
- Test sensitivity of results to unmeasured confounding
- *EValue* package

7. Print out results and then try to make sense of everything ☺

Additional packages to help with tables, documents, formatting, etc.
- Packages: *gtsummary, flextable, officer, ggplot2, patchwork, pandoc, qpdf, flexlsx, openxlsx2, forcats, fastDummies, haven, remotes*

All wrapped into *Rglobalflourishing*

```
remotes::install_github("noah-padgett/Rglobalflourishing")
```

Backed-up on OSF: 10.17605/OSF.IO/RBCMP

Estimating equation. A recurring question we have received from reviewers on substantive papers is what the "estimating equation" is for the country-specific regression analyses. Here we aim to provide a thorough documentation of the "equation" being estimated within each country:

$$\text{Model 1: } Y_{t=2}^{m} = f(X_{t=1}, \boldsymbol{C})$$
$$\text{Model 2: } Y_{t=2}^{m} = f\left(X_{t=1}, \boldsymbol{C}, \boldsymbol{Z}_{(7)}\right)$$

where,

- $f(.)$, link function, is either identity for approximately continuous outcomes or the Poisson log-link function for binary outcomes.
- $Y_{t=2}^{m}$, outcome '*m*' assessed at time 2.
- $X_{t=1}$, focal exposure (predictor) assessed at time 1.
- $\boldsymbol{C}$, exogenous controls/covariates assessed at time 1, includes the 17 demographic and childhood variables: (1) Gender, (2) age (categorized), (3) marital status, (4) employment status, (5) years of education, (6) current religious service, (7) immigration status, (8) current religious affiliation, (9) racial/ethnic, (10) Relationship quality with mother during childhood, (11) relationship quality with father, (12) parental marital status during childhood, (13) childhood financial, (14) history of abuse, (15) subjective family, (16) childhood health, and (17) religious service attendance during childhood.
- $\boldsymbol{Z}_{(7)}$, principal components (first seven) constructed using variables assessed at time 1. Principal components were constructed using all outcomes (***including the outcome assessed at time 1,*** $\boldsymbol{Y_{t=1}^{m}}$), see the list of outcomes in section ***Outcome variables*** in the main text, plus personality (Big-5), number of people living in the household of the participant, living in an urban/rural area, and all these variables were assessed at time 1, which coincides with the assessment of the focal exposure ($X_{t=1}$). The interested reader is referred to Galbraith and Zinde-Walsh (2020) for more technical details on how principal components can be used to reduce the amount of information/multicollinearity in a set of confounders used for regression analyses.
- Note, model 1 does not include the outcome assessed at time because it assessed contemporaneously as the focal exposure.
- Example: $Y_{t=2} = \beta_1 X_{t=1} + \boldsymbol{C}\gamma + \beta_0 + \varepsilon$, where the focal causal effect of interest is β_1, with β_0 is the intercept, and γ is the vector of regression coefficients for each ($\boldsymbol{C}$).

Galbraith, J. W., & Zinde-Walsh, V. (2020). Simple and reliable estimators of coefficients of interest in a model with high-dimensional confounding effects. *Journal of Econometrics*, *218*(2), 609–632. https://doi.org/10.1016/j.jeconom.2020.04.031

Heterogeneity statistic interpretation. First, Cochran's Q-statistic is a weighted sum of squares of the estimated coefficients relative to the pooled estimate. The I^2 statistic is an estimate of the variability in means due to heterogeneity across countries vs. sampling variability.

$$I^2 = \frac{Q - K - 1}{Q}$$

Where *K* is the number of estimates being pooled (generally 23 in our case).

The estimate of heterogeneity (τ) is the standard deviation of the distribution of effect sizes, and is estimated using the Paul-Mandel method.

Global P-Values (Combining P-Values from Country-Specific Tests). A decision was made to report p-values because it is common practice across many disciplines, and the type of p-value reported provides a high-level summary of the country-specific p-values. The harmonic mean p-value was used to combine p-values across different countries [1,2]. The combined p-value was used to test the null hypothesis of no effect of a variable (all categories have an estimated effect of 0 relative to baseline) in all countries, against the alternative hypothesis that in at least one country the group of regression coefficients (or risk ratios) for a given predictor are significantly different than 0 (or 1 for risk ratios). The harmonic mean p-value method is more robust to dependency among pooled p-values [2]. Although the country-specific tests are technically independent—an underlying assumption of most classic approaches to pooling p-values [3]— assuming independence of the p-values may not be entirely tenable given a common underlying set of items, translation procedures, childhood predictors, data cleaning techniques, and imputation models. To account for multiple testing, we present Bonferroni-corrected p-value significance thresholds for the meta-analytic results based on the number of predictors [4,5] in the primary meta-analytic results. The Bonferroni adjustment for multiplicity was applied to the significance level cutoff (alpha) and not the p-values (we divided alpha by the number of tests and not multiplying the p-values by the number of tests). Providing the standard 0.05 significance threshold and Bonferroni-adjusted significance threshold provides transparency in how multiplicity was considered. However, the reported harmonic mean p-value is relatively robust to multiple testing already maintaining a constant Type-I error rate regardless of the number of tests being conducted [2].

1. Vovk V, Wang R. Combining p-values via averaging. Biometrika. 2020;107(4):791-808.
2. Wilson DJ. The harmonic mean p-value for combining dependent tests. Proc Natl Acad Sci USA. 2019;116(4):1195-200.
3. Loughin TM. A systematic comparison of methods for combining p-values from independent tests. Comput Stat Data Anal. 2004;47(3):467-85. Available from: https://doi.org/10.1016/j.csda.2003.11.020
4. Abdi H. Bonferroni and Šidák corrections for multiple comparisons. In: Encyclopedia of Measurement and Statistics. 2007;3(1):1-9.
5. VanderWeele TJ, Mathur MB. Some desirable properties of the Bonferroni correction: Is the Bonferroni correction really so bad? Am J Epidemiol. 2019;188(3):617-8.

Comments on the dichotomization of continuous variables for E-value calibration. In the main text, we suggested the use of a dichotomization that splits continuous variables into a dichotomized variable indicating being in the top or bottom quintile of the distribution—which has the limitation of only utilizing 40% of the sample which is less than optimal. An advantage of this approach is that it creates a dichotomous variable with a noticeable gap between the those classified as 1 (top 20%) and as 0 (bottom 20%) leading to less uncertainty of differences based on uncertainty around the cutoff. Alternatively, one could use a median split to dichotomize around the median to create two equally sized groups, just would though need to handle ties around the median. Similarly, one could use a mean split which may lead to unequal groups.

Differential Item Functioning of Single-item Measures

We developed an approach to testing approximate invariance of single-item measures using a penalized heterogeneous ordered probit model (HETOP, Padgett, 2026). The test provides a preliminary evaluation of the equality of item location and the strength of the association between the latent variable and the observed item (factor loading or discrimination parameter). The penalized HETOP model uses the discrete nature of (nearly) all outcome variables in the GFS, along with an item response theory-like parameterization for ordered categorical variables, to test for differential item functioning.
By modeling the probability of each category as a function of group invariant thresholds and group varying latent response variable location and discrimination parameters, we can identify how changes in a penalty, or regularization term, impact the differences in location and discrimination. The model is

$$\Pr(Y = k) = \Phi\left(\lambda_g(\mu_g - \tau_k)\right) - \Phi\left(\lambda_g(\mu_g - \tau_{k+1})\right),$$

where Φ is the cumulative normal distribution; λ_g is the discrimination parameter for group g , denoting the strength of the relationship between the latent response variable and the observed variable; μ_g is the location of group g on latent response variable; and τ is the group invariant threshold parameter. This parameterization of the HETOP model is similar to common item response theory models for ordinal items, such as the graded response model. The above model for the probability of a given response can then be used to form a likelihood of a set of pre-estimated category counts across a range of groups.

$$\ln L_p = \sum_{g=1}^{G} w_g \sum_{k=0}^{K} n_{gk} \ln\{\Pr(Y = k \mid \mu_g, \lambda_g)\} + P(\boldsymbol{\mu}) + P(\boldsymbol{\lambda})$$

$$P(\theta) = \sum_{\forall g \in G} \sum_{\forall h \neq g \in G} \frac{f(\theta_g, \theta_h)}{\nu}$$

$$f(\theta_g, \theta_h) = \sqrt{|\theta_g - \theta_h| + \varepsilon}, \text{alignment loss function}$$

A major advantage of this model parameterization is that the group counts (n_{gk}) for each category can be pre-computed using complex-survey design adjustments.

Table S1.

Summary of top 20 eigenvalues from principal components.

PC	Argentina	Australia	Brazil	China	Egypt	Germany	Hong Kong	India	Indonesia	Israel	Japan	Kenya	Mexico	Nigeria	Philippines	Poland	South Africa	Spain	Sweden	Tanzania	Turkey	United Kingdom	United States
1	11.17	14.34	12.99	14.42	6.60	11.89	21.49	7.05	8.22	14.66	18.65	6.34	11.43	7.14	7.81	12.60	7.29	10.38	15.37	7.34	12.44	15.63	14.76
2	5.09	6.53	4.22	7.15	2.66	4.50	8.07	3.33	3.40	6.35	4.96	3.33	4.64	3.78	3.33	5.11	3.20	4.92	5.35	2.87	5.05	6.46	6.06
3	2.90	2.69	2.66	3.24	2.25	2.55	3.09	2.39	3.03	2.97	2.47	2.24	3.22	2.70	2.49	3.13	3.01	2.77	2.72	2.43	2.72	2.74	2.52
4	2.30	2.34	2.44	2.08	2.03	2.40	2.04	2.22	2.07	2.26	2.06	2.13	2.86	2.38	1.97	2.60	2.20	2.40	2.40	2.27	2.36	2.24	2.24
5	2.09	2.05	1.86	1.88	1.80	2.08	1.67	1.96	1.93	2.07	1.80	2.08	2.10	2.31	1.93	1.86	2.04	1.96	1.86	2.00	2.03	2.00	2.02
6	1.71	1.86	1.70	1.71	1.79	1.65	1.54	1.84	1.79	1.84	1.66	1.79	1.77	2.13	1.79	1.80	1.66	1.59	1.72	1.78	1.69	1.89	1.81
7	1.64	1.76	1.57	1.64	1.62	1.58	1.41	1.76	1.72	1.74	1.50	1.62	1.67	1.78	1.67	1.72	1.64	1.49	1.62	1.72	1.66	1.70	1.71
8	1.56	1.60	1.45	1.42	1.56	1.50	1.39	1.62	1.65	1.65	1.47	1.53	1.47	1.74	1.56	1.66	1.56	1.45	1.54	1.61	1.60	1.48	1.52
9	1.39	1.49	1.40	1.40	1.44	1.47	1.26	1.50	1.54	1.54	1.37	1.44	1.37	1.63	1.53	1.58	1.44	1.42	1.42	1.49	1.53	1.34	1.44
10	1.37	1.45	1.35	1.35	1.43	1.36	1.20	1.44	1.44	1.47	1.28	1.37	1.33	1.48	1.46	1.46	1.42	1.38	1.38	1.44	1.47	1.31	1.36
11	1.31	1.34	1.32	1.26	1.40	1.32	1.17	1.38	1.36	1.43	1.27	1.28	1.27	1.46	1.38	1.39	1.39	1.33	1.31	1.32	1.44	1.23	1.31
12	1.25	1.25	1.22	1.24	1.31	1.26	1.10	1.30	1.31	1.29	1.25	1.25	1.23	1.33	1.28	1.35	1.35	1.30	1.25	1.28	1.41	1.22	1.21
13	1.22	1.17	1.16	1.18	1.26	1.24	1.06	1.27	1.30	1.21	1.19	1.24	1.22	1.30	1.21	1.26	1.29	1.28	1.20	1.22	1.36	1.14	1.19
14	1.21	1.12	1.14	1.10	1.26	1.21	1.03	1.18	1.29	1.21	1.16	1.21	1.18	1.26	1.18	1.23	1.26	1.23	1.13	1.20	1.27	1.11	1.15
15	1.14	1.10	1.13	1.09	1.21	1.17	1.01	1.17	1.21	1.14	1.12	1.15	1.14	1.25	1.14	1.16	1.20	1.18	1.12	1.18	1.22	1.09	1.10
16	1.14	1.09	1.08	1.06	1.19	1.14	0.97	1.15	1.17	1.12	1.10	1.12	1.10	1.20	1.11	1.15	1.18	1.17	1.09	1.12	1.21	1.06	1.04
17	1.11	1.07	1.04	1.02	1.16	1.07	0.93	1.11	1.16	1.08	1.06	1.11	1.07	1.18	1.09	1.09	1.14	1.11	1.07	1.10	1.16	1.04	1.04
18	1.06	1.04	1.02	0.97	1.13	1.06	0.91	1.08	1.07	1.06	1.04	1.07	1.06	1.15	1.07	1.06	1.12	1.06	1.04	1.08	1.13	1.01	1.02
19	1.05	0.99	1.02	0.96	1.11	1.04	0.87	1.07	1.05	1.01	1.00	1.06	1.03	1.13	1.06	1.02	1.11	1.04	1.00	1.07	1.07	1.00	1.00
20	1.04	0.98	0.99	0.95	1.08	1.02	0.84	1.04	1.02	0.99	0.98	1.03	0.99	1.11	1.05	1.01	1.10	1.03	0.99	1.05	1.05	0.97	0.97

Table S2.

Summary of top 20 principal components percent of variance explained by each component.

PC	Argentina	Australia	Brazil	China	Egypt	Germany	Hong Kong	India	Indonesia	Israel	Japan	Kenya	Mexico	Nigeria	Philippines	Poland	South Africa	Spain	Sweden	Tanzania	Turkey	United Kingdom	United States
1	33.6	34.2	33.5	25.8	34.2	33.3	32.7	29.6	42.5	30.7	28.9	26.3	34.0	38.3	32.0	29.4	31.6	31.2	32.0	27.3	26.9	34.6	37.1
2	5.9	7.8	6.3	9.2	5.0	5.9	10.6	4.6	4.0	7.0	7.5	4.2	5.5	4.6	3.6	7.0	4.2	6.0	7.8	5.1	7.5	7.4	7.4
3	4.6	7.2	3.5	5.8	2.6	4.8	6.8	2.6	2.4	6.6	6.1	2.6	4.4	2.6	3.0	5.4	3.0	5.2	5.2	2.6	3.4	6.7	5.8
4	2.4	2.5	2.3	3.4	2.1	2.3	3.5	2.2	1.8	2.9	2.7	2.3	2.7	2.0	2.1	2.6	2.1	2.5	2.4	2.2	2.5	2.5	2.3
5	2.2	1.8	2.2	2.5	2.0	1.9	1.9	2.0	1.7	2.2	2.0	2.1	2.2	1.9	2.0	2.2	2.0	1.9	2.2	2.2	2.4	2.0	1.9
6	1.9	1.8	1.8	2.0	1.8	1.8	1.7	1.9	1.6	2.0	1.9	1.9	2.0	1.9	1.8	2.0	1.9	1.9	1.7	1.9	2.2	1.8	1.7
7	1.8	1.7	1.6	1.8	1.6	1.6	1.5	1.7	1.6	1.9	1.7	1.8	1.7	1.6	1.7	1.8	1.8	1.6	1.7	1.8	2.0	1.6	1.6
8	1.6	1.6	1.6	1.7	1.6	1.5	1.4	1.6	1.5	1.8	1.6	1.7	1.6	1.5	1.7	1.7	1.6	1.5	1.6	1.6	1.8	1.5	1.5
9	1.5	1.5	1.6	1.6	1.5	1.5	1.4	1.5	1.4	1.6	1.5	1.6	1.5	1.4	1.6	1.6	1.6	1.5	1.6	1.5	1.7	1.4	1.4
10	1.4	1.5	1.4	1.6	1.4	1.4	1.3	1.5	1.3	1.5	1.5	1.4	1.4	1.4	1.5	1.5	1.6	1.5	1.5	1.5	1.6	1.4	1.4
11	1.4	1.4	1.4	1.5	1.4	1.3	1.3	1.4	1.3	1.4	1.4	1.4	1.4	1.3	1.4	1.5	1.5	1.4	1.4	1.4	1.5	1.3	1.3
12	1.3	1.3	1.3	1.4	1.4	1.3	1.2	1.4	1.2	1.4	1.4	1.4	1.4	1.3	1.3	1.4	1.4	1.4	1.4	1.4	1.5	1.3	1.3
13	1.3	1.2	1.3	1.4	1.3	1.3	1.2	1.3	1.2	1.3	1.3	1.3	1.3	1.3	1.3	1.4	1.4	1.3	1.4	1.3	1.4	1.2	1.2
14	1.2	1.2	1.2	1.4	1.2	1.2	1.1	1.3	1.2	1.3	1.3	1.3	1.2	1.3	1.2	1.3	1.3	1.3	1.3	1.3	1.4	1.2	1.1
15	1.2	1.2	1.2	1.3	1.2	1.2	1.1	1.2	1.2	1.2	1.2	1.2	1.2	1.2	1.2	1.3	1.3	1.2	1.2	1.3	1.3	1.1	1.1
16	1.2	1.1	1.2	1.2	1.2	1.2	1.1	1.2	1.1	1.2	1.2	1.2	1.1	1.2	1.2	1.2	1.2	1.2	1.1	1.2	1.3	1.1	1.1
17	1.2	1.1	1.1	1.2	1.2	1.1	1.1	1.1	1.1	1.1	1.2	1.2	1.1	1.2	1.2	1.2	1.2	1.1	1.1	1.2	1.2	1.1	1.0
18	1.1	1.0	1.1	1.2	1.1	1.1	1.0	1.1	1.1	1.1	1.2	1.2	1.1	1.1	1.1	1.2	1.2	1.1	1.0	1.2	1.2	1.0	1.0
19	1.1	1.0	1.1	1.1	1.1	1.1	1.0	1.1	1.1	1.1	1.1	1.1	1.0	1.1	1.1	1.1	1.2	1.1	1.0	1.1	1.2	1.0	1.0
20	1.0	1.0	1.1	1.1	1.1	1.0	0.9	1.1	1.0	1.0	1.1	1.1	1.0	1.0	1.1	1.1	1.1	1.1	1.0	1.1	1.1	1.0	1.0

Table S3. *Benchmarking across a range of observed potential confounders to calibrate E-values for sensitivity analysis for unmeasured confounding.*

		Age Group								Parental Marital Status			
	Gender	18-24	25-29	30-39	40-49	50-59	60-69	70+	Employed	Married	Divorced	Immigrant	Race
RR between Observed Confounder & Exposure	1.11	1.03	1.01	1.02	1.02	1.01	1.24	1.45	1.13	1.10	1.08	1.31	1.19
Outcome													
Human Flourishing													
Secure flourishing index	1.20	2.08	1.75	1.28	1.34	1.88	1.65	1.40	1.10	1.83	4.01	1.63	1.29
Flourishing index	1.30	2.17	1.88	1.41	1.21	1.92	2.22	1.77	1.08	1.72	3.53	1.80	1.54
Happiness & life satisfaction	1.31	1.64	1.51	1.14	1.25	1.64	1.29	1.33	1.14	1.80	2.60	1.45	1.40
Physical & mental health	1.07	2.03	1.81	1.36	1.12	1.87	2.25	2.34	1.07	1.45	3.17	1.91	1.28
Meaning & purpose	1.26	1.77	1.72	1.32	1.17	1.65	1.78	1.56	1.08	1.65	2.24	1.40	1.36
Character & virtue	1.25	1.75	1.73	1.39	1.05	1.56	2.07	2.38	1.03	1.29	1.79	1.28	1.28
Close social relationships	1.31	1.83	1.53	1.30	1.15	1.69	1.78	1.37	1.18	1.63	2.84	1.64	1.31
Financial & material security	1.13	1.39	1.41	1.42	1.39	1.26	1.36	2.34	1.12	1.59	1.61	1.26	1.67
Psychological Well-Being													
Happiness (a)	1.32	1.82	1.59	1.14	1.23	1.75	1.44	1.37	1.14	1.67	2.87	1.80	1.43
Life satisfaction (a)	1.29	1.60	1.48	1.14	1.19	1.62	1.41	1.35	1.17	1.78	2.79	1.55	1.42
Current life evaluation	1.27	1.33	1.26	1.19	1.25	1.36	1.22	1.36	1.09	1.58	1.84	1.14	1.24
Future life evaluation	1.32	2.03	1.88	1.40	1.11	1.66	2.19	2.85	1.20	1.10	2.41	1.10	1.31
Optimism	1.23	1.55	1.45	1.21	1.04	1.36	1.63	1.63	1.07	1.38	1.81	1.31	1.41
Freedom to pursue what's important	1.20	1.73	1.55	1.28	1.16	1.59	1.80	1.62	1.22	1.33	2.11	1.49	1.43
Inner peace	1.03	1.15	1.12	1.11	1.11	1.08	1.11	1.42	1.08	1.20	1.04	1.10	1.14
Life balance	1.04	1.19	1.15	1.17	1.15	1.07	1.19	1.53	1.06	1.24	1.05	1.07	1.18
Sense of mastery	1.02	1.29	1.24	1.10	1.05	1.21	1.11	1.19	1.07	1.13	1.19	1.05	1.08
Meaningful activities (c)	1.28	1.64	1.56	1.25	1.12	1.51	1.58	1.51	1.01	1.55	2.13	1.36	1.19
Understanding purpose (c)	1.14	1.81	1.67	1.30	1.10	1.64	2.08	1.78	1.09	1.56	2.18	1.41	1.43
Self-rated mental health (b)	1.09	1.85	1.64	1.25	1.17	1.65	2.08	1.74	1.16	1.65	2.79	2.08	1.21
Psychological Distress													
Traumatic distress	1.21	1.23	1.23	1.18	1.09	1.07	1.24	1.30	1.12	1.08	1.08	1.09	1.56
Depression -- feel hopeless	1.16	1.80	1.58	1.51	1.20	1.18	1.89	2.63	1.07	1.39	1.22	1.18	1.67
Depression -- loss of interest	1.05	1.67	1.54	1.41	1.20	1.17	1.72	2.14	1.05	1.39	1.28	1.25	1.69
Anxiety -- feel on edge	1.29	1.68	1.56	1.58	1.32	1.17	2.02	2.99	1.04	1.44	1.24	1.17	1.56
Anxiety -- cannot stop worrying	1.30	1.70	1.60	1.54	1.27	1.20	1.91	2.73	1.06	1.41	1.24	1.20	1.77
Suffering	1.14	1.13	1.07	1.06	1.06	1.07	1.07	1.09	1.14	1.10	1.18	1.07	1.19
Social Well-Being													
Relationship contentment (e)	1.26	1.84	1.59	1.29	1.12	1.71	1.85	1.53	1.19	1.47	2.60	1.69	1.37
Relationship satisfaction (e)	1.23	1.59	1.40	1.21	1.14	1.46	1.50	1.19	1.12	1.55	2.26	1.32	1.20
Social support	1.37	1.54	1.40	1.21	1.14	1.47	1.54	1.31	1.08	1.24	1.48	1.13	1.29
Intimate/close friend	1.12	1.11	1.08	1.02	1.03	1.10	1.04	1.03	1.01	1.19	1.17	1.04	1.04
Government approval	1.03	1.42	1.39	1.17	1.06	1.29	1.40	1.37	1.03	1.36	1.81	1.24	1.18
Say in government	1.02	1.35	1.23	1.16	1.07	1.26	1.22	1.21	1.04	1.24	1.45	1.23	1.18
Belonging in country	1.11	1.42	1.32	1.09	1.18	1.36	1.27	1.19	1.18	1.48	1.46	1.22	1.07
City/place satisfaction	1.04	1.09	1.05	1.04	1.05	1.06	1.04	1.16	1.03	1.24	1.15	1.02	1.10
Trust within country	1.04	1.01	1.04	1.08	1.03	1.04	1.09	1.13	1.05	1.10	1.13	1.03	1.08
Social Participation													
Ever been married	1.07	3.28	1.50	1.11	1.07	1.12	1.30	1.43	1.05	8.40	1.44	1.03	1.15
Currently divorced	1.54	20.95	6.61	3.11	1.23	1.84	2.16	1.79	1.12	10.42	74.62	1.41	2.28
Number of children	1.23	1.51	1.59	2.59	2.02	2.05	5.77	14.03	1.32	2.00	3.34	1.56	1.77
Weekly+ community participation	1.18	1.54	1.40	1.07	1.15	1.53	1.26	1.32	1.16	1.06	1.58	1.06	1.42
Weekly+ religious attendance	1.18	1.63	1.52	1.32	1.06	1.36	1.76	2.39	1.15	1.52	2.57	1.83	1.53
Social Distress													
Loneliness	1.08	1.27	1.26	1.21	1.16	1.08	1.33	1.78	1.14	1.52	1.28	1.04	1.28
Perceived discrimination	1.05	1.29	1.23	1.21	1.14	1.04	1.21	1.81	1.02	1.20	1.10	1.33	1.59
Character & Prosocial Behavior													
Orientation to promote good (d)	1.21	1.69	1.70	1.33	1.05	1.52	2.01	2.12	1.08	1.37	1.88	1.33	1.21
Delayed gratification (d)	1.21	1.69	1.67	1.35	1.03	1.47	1.89	2.49	1.01	1.25	1.68	1.21	1.27
Hope	1.26	1.93	1.76	1.42	1.07	1.67	2.29	2.86	1.07	1.29	2.38	1.59	1.45
Gratitude	1.39	1.67	1.59	1.29	1.10	1.46	1.72	1.76	1.11	1.32	1.72	1.21	1.24
Showing love/care	1.41	1.44	1.44	1.30	1.04	1.39	1.56	1.61	1.15	1.47	1.35	1.08	1.15
Forgivingness	1.06	1.13	1.11	1.02	1.05	1.10	1.05	1.07	1.05	1.12	1.02	1.03	1.06
Charitable giving	1.01	1.29	1.19	1.19	1.15	1.21	1.16	1.42	1.13	1.38	1.17	1.22	1.15
Helping strangers	1.07	1.58	1.44	1.22	1.08	1.23	1.46	2.31	1.08	1.09	1.28	1.04	1.29
Volunteering	1.06	1.60	1.40	1.18	1.05	1.39	1.42	1.38	1.11	1.35	1.64	1.23	1.57
Physical Health & Health Behavior													
Self-rated physical health (b)	1.07	2.16	1.91	1.37	1.11	1.87	2.33	2.94	1.16	1.21	2.93	1.39	1.29
Health problems	1.14	1.81	1.72	1.52	1.19	1.18	1.38	1.69	1.71	1.24	1.54	1.09	1.16
Pain in past 4 weeks	1.15	1.19	1.14	1.11	1.05	1.05	1.09	1.17	1.21	1.13	1.18	1.04	1.08
Number of drinks per week	2.10	2.29	1.57	1.37	1.05	1.28	1.48	1.51	1.46	1.05	1.44	1.36	2.50
Days exercise per week	1.33	1.13	1.13	1.08	1.09	1.21	1.07	1.08	1.05	1.13	1.41	1.08	1.16
Socioeconomic Outcomes													
Financial security (f)	1.12	1.32	1.33	1.37	1.32	1.29	1.26	2.03	1.11	1.50	1.63	1.30	1.53
Material security (f)	1.12	1.25	1.32	1.36	1.32	1.31	1.31	1.89	1.13	1.54	1.97	1.12	1.70
Educational attainment (16+ years)	1.05	2.04	1.12	1.16	1.08	1.35	1.11	1.39	1.38	1.25	1.14	2.02	1.62
Currently employed	1.42	1.31	1.25	1.48	1.55	1.47	1.57	6.24	5.68	1.18	1.11	1.03	1.08
Financially comfortable/getting by	1.07	1.19	1.17	1.14	1.10	1.02	1.16	1.34	1.06	1.10	1.08	1.14	1.45
Own home	1.05	1.27	1.34	1.15	1.03	1.09	1.17	1.23	1.02	1.32	1.38	1.08	1.25

Table S4.
Summary of penalized HETOP evaluation of which countries for each outcomes are identified with uniform and nonuniform differential item functioning.

Outcome	# Categories	Uniform	Nonuniform*
Psychological Well-Being			
Happiness (a)	11	JPN, POL	IND, KEN, TZA
Life satisfaction (a)	11	JPN, POL	EGY, IND, KEN, TZA
Current life evaluation	11	IND, JPN, POL	IND, KEN, TZA
Future life evaluation	11	BRA, JPN	IND, KEN, TZA
Optimism	11	KEN	IND, KEN, TZA
Freedom to pursue what's important	11	JPN, TZA	IND, KEN, TZA
Inner peace	4	-	IND
Life balance	4	NGA, TZA	JPN, NGA
Sense of mastery	4	EGY, JPN	IND
Meaningful activities (c)	11	JPN	TZA
Understanding purpose (c)	11	JPN	IND, KEN, TZA
Self-rated mental health (b)	11	KEN, TZA	IND, KEN, TZA
Psychological Distress			
Traumatic distress	4	KEN, NGA, POL	EGY, IND
Depression -- feel hopeless	4	NGA, POL, USA	-
Depression -- loss of interest	4	NGA, PHL, USA	IND, TZA
Anxiety -- feel on edge	4	BRA, EGY	IND, TZA
Anxiety -- cannot stop worrying	4	PHL, POL	IND, TZA
Suffering	4	ISR, PHL, POL	EGY, IND
Social Well-Being			
Relationship contentment (e)	11	JPN	IND, KEN, TZA
Relationship satisfaction (e)	11	JPN	KEN, TZA
Social support	11	JPN	EGY, IND, KEN, TZA
Intimate/close friend	2	JPN	JPN
Government approval	5	-	HKG
Say in government	3	-	HKG
Belonging in country	11	-	HKG
City/place satisfaction	3	JPN, PHL, POL	PHL, SWE
Trust within country	5	CHN, SWE, USA	EGY, IND, IDN, TUR
Social Participation			
Weekly+ community participation	5	EGY, JPN	EGY, JPN
Weekly+ religious attendance	5	JPN, NGA, SWE, TUR	EGY, USA
Social Distress			
Loneliness	11	JPN, POL	IND
Perceived discrimination	4	POL, SWE	EGY, IND
Character & Prosocial Behavior			
Orientation to promote good (d)	11	-	HKG
Delayed gratification (d)	11	JPN	KEN, TZA
Hope	11	JPN	IND, KEN, TZA
Gratitude	11	HKG, JPN	IND, KEN, TZA
Showing love/care	11	HKG, JPN, TZA	IND, KEN, TZA
Forgivingness	4	JPN	IND
Charitable giving	2	IDN, JPN, POL	-
Helping strangers	2	JPN, NGA, POL, SWE	-
Volunteering	2	JPN, NGA, SWE	-
Physical Health & Health Behavior			
Self-rated physical health (b)	11	JPN	IND, KEN, TZA
Health problems	2	PHL, POL	-
Pain in past 4 weeks	4	EGY, ISR, POL	EGY, IND
Days exercise per week	8	EGY	IND
Socioeconomic Outcomes			
Financial security (f)	11	ARG, BRA, SWE, USA	IND, KEN, TZA
Material security (f)	11	ARG, BRA, POL, SWE, USA	IND, KEN, TZA
Educational attainment (16+ years)	3	CHN, IND, TZA, USA	CHN, EGY, IND, GBR
Financially comfortable/getting by	4	KEN, SWE, USA	IND, USA

Note. ARG, Argentina; AUS, Australia; BRA, Brazil; CHN, China; EGY, Egypt; DEU, Germany; HKG, Hong Kong; IND, India; IDN, Indonesia; ISR, Israel; JPN, Japan; KEN, Kenya; MEX, Mexico; NGA, Nigeria; PHL, Philippines; POL, Poland; ZAF, South Africa; ESP, Spain; SWE, Sweden; TZA, Tanzania; TUR, Turkey; GBR, United Kingdom; USA, United States. *Nonuniform DIF is only reported for lower than average discrimination as higher discrimination means the item is operating better, not worse.

Table S5.
Reliability corrected meta-analyzed estimates of association, Estimate (95% CI).

Outcome	Primary Result	Reliability corrected estimates 0.40	0.55	0.7
Human Flourishing				
**Secure flourishing index**	**0.201 (0.164, 0.237)**	**0.502 (0.466, 0.539)**	**0.365 (0.329, 0.402)**	**0.287 (0.250, 0.323)**
Flourishing index	0.204 (0.165, 0.243)	0.510 (0.471, 0.548)	0.371 (0.332, 0.409)	0.291 (0.253, 0.330)
Psychological Well-Being				
Happiness	0.132 (0.097, 0.167)	0.330 (0.295, 0.365)	0.240 (0.205, 0.275)	0.189 (0.154, 0.224)
Life satisfaction	0.134 (0.098, 0.170)	0.335 (0.299, 0.371)	0.244 (0.208, 0.280)	0.192 (0.155, 0.228)
Current life evaluation	0.121 (0.092, 0.151)	0.303 (0.274, 0.333)	0.221 (0.191, 0.250)	0.173 (0.144, 0.203)
Future life evaluation	0.119 (0.091, 0.148)	0.298 (0.270, 0.327)	0.217 (0.189, 0.245)	0.170 (0.142, 0.199)
Optimism	0.134 (0.099, 0.169)	0.335 (0.300, 0.370)	0.244 (0.209, 0.279)	0.192 (0.157, 0.227)
Freedom to pursue what' important	0.137 (0.106, 0.169)	0.344 (0.312, 0.375)	0.250 (0.219, 0.281)	0.196 (0.165, 0.228)
Inner peace	0.086 (0.063, 0.109)	0.215 (0.192, 0.238)	0.156 (0.134, 0.179)	0.123 (0.100, 0.146)
Life balance	0.100 (0.080, 0.121)	0.251 (0.231, 0.272)	0.183 (0.162, 0.203)	0.144 (0.123, 0.164)
Sense of mastery	0.128 (0.085, 0.171)	0.320 (0.277, 0.363)	0.233 (0.190, 0.275)	0.183 (0.140, 0.225)
Meaningful activities	0.158 (0.122, 0.195)	0.396 (0.359, 0.433)	0.288 (0.251, 0.325)	0.226 (0.190, 0.263)
Understanding purpose	0.153 (0.121, 0.185)	0.383 (0.350, 0.415)	0.278 (0.246, 0.311)	0.219 (0.186, 0.251)
Self-rated mental health	0.143 (0.107, 0.179)	0.357 (0.321, 0.393)	0.259 (0.223, 0.295)	0.204 (0.168, 0.240)
Psychological Distress				
Traumatic distress	-0.058 (-0.080, -0.035)	-0.144 (-0.167, -0.122)	-0.105 (-0.128, -0.082)	-0.082 (-0.105, -0.060)
Depression symptoms composite	-0.157 (-0.210, -0.105)	-0.394 (-0.446, -0.341)	-0.286 (-0.339, -0.233)	-0.225 (-0.278, -0.172)
Anxiety symptoms composite	-0.155 (-0.207, -0.103)	-0.388 (-0.440, -0.336)	-0.282 (-0.334, -0.230)	-0.222 (-0.274, -0.169)
Suffering	-0.075 (-0.103, -0.046)	-0.187 (-0.215, -0.158)	-0.136 (-0.164, -0.107)	-0.107 (-0.135, -0.078)
Social Well-Being				
Relationship contentment	0.128 (0.099, 0.157)	0.320 (0.291, 0.349)	0.233 (0.204, 0.262)	0.183 (0.154, 0.212)
Relationship satisfaction	0.127 (0.100, 0.155)	0.319 (0.291, 0.346)	0.232 (0.204, 0.259)	0.182 (0.154, 0.210)
Social support	0.094 (0.072, 0.116)	0.234 (0.213, 0.256)	0.171 (0.149, 0.192)	0.134 (0.112, 0.156)
Intimate/close friend	0.027 (0.019, 0.036)	0.068 (0.059, 0.077)	0.050 (0.041, 0.058)	0.039 (0.030, 0.048)
Government approval	0.038 (0.018, 0.058)	0.095 (0.075, 0.115)	0.069 (0.049, 0.089)	0.054 (0.034, 0.074)
Say in government	0.049 (0.028, 0.070)	0.122 (0.101, 0.143)	0.089 (0.068, 0.109)	0.070 (0.049, 0.091)
Belonging in country	0.101 (0.081, 0.121)	0.252 (0.232, 0.272)	0.183 (0.163, 0.203)	0.144 (0.124, 0.164)
City/place satisfaction	0.040 (0.029, 0.052)	0.100 (0.089, 0.112)	0.073 (0.062, 0.084)	0.057 (0.046, 0.069)
Trust within country	0.034 (0.024, 0.044)	0.085 (0.075, 0.095)	0.062 (0.052, 0.072)	0.049 (0.038, 0.059)
Social Participation				
Ever been married	0.003 (0.001, 0.005)	0.007 (0.005, 0.009)	0.005 (0.003, 0.007)	0.004 (0.002, 0.006)
Currently divorced	0.002 (-0.015, 0.020)	0.006 (-0.012, 0.024)	0.004 (-0.013, 0.022)	0.003 (-0.014, 0.021)
Number of children	-0.001 (-0.010, 0.007)	-0.003 (-0.012, 0.006)	-0.002 (-0.011, 0.007)	-0.002 (-0.010, 0.007)
Weekly+ community participation	0.057 (0.029, 0.084)	0.141 (0.114, 0.169)	0.103 (0.075, 0.130)	0.081 (0.053, 0.108)
Weekly+ religious attendance	0.008 (0.001, 0.015)	0.020 (0.013, 0.028)	0.015 (0.007, 0.022)	0.012 (0.004, 0.019)
Social Distress				
Loneliness	-0.108 (-0.133, -0.083)	-0.269 (-0.294, -0.244)	-0.196 (-0.221, -0.171)	-0.154 (-0.179, -0.129)
Perceived discrimination	-0.028 (-0.056, -0.001)	-0.071 (-0.098, -0.043)	-0.052 (-0.079, -0.024)	-0.040 (-0.068, -0.013)
Character & Prosocial Behavior				
Orientation to promote good	0.125 (0.099, 0.152)	0.313 (0.287, 0.340)	0.228 (0.202, 0.254)	0.179 (0.153, 0.205)
Delayed gratification	0.096 (0.074, 0.118)	0.240 (0.218, 0.262)	0.175 (0.153, 0.197)	0.137 (0.115, 0.159)
Hope	0.155 (0.121, 0.189)	0.388 (0.354, 0.422)	0.282 (0.248, 0.316)	0.222 (0.188, 0.256)
Gratitude	0.119 (0.090, 0.148)	0.298 (0.268, 0.327)	0.216 (0.187, 0.246)	0.170 (0.141, 0.199)
Showing love/care	0.103 (0.081, 0.126)	0.258 (0.236, 0.280)	0.188 (0.165, 0.210)	0.148 (0.125, 0.170)
Forgivingness	0.031 (0.022, 0.040)	0.077 (0.069, 0.086)	0.056 (0.048, 0.065)	0.044 (0.035, 0.053)
Charitable giving	0.029 (0.017, 0.041)	0.072 (0.060, 0.084)	0.052 (0.040, 0.064)	0.041 (0.029, 0.053)
Helping strangers	0.039 (0.026, 0.051)	0.096 (0.084, 0.109)	0.070 (0.057, 0.083)	0.055 (0.042, 0.068)
Volunteering	0.054 (0.031, 0.078)	0.135 (0.112, 0.159)	0.098 (0.075, 0.122)	0.077 (0.054, 0.101)
Physical Health & Health Behavior				
Self-rated physical health	0.121 (0.092, 0.149)	0.302 (0.273, 0.331)	0.220 (0.191, 0.248)	0.173 (0.144, 0.201)
Health problems	-0.092 (-0.122, -0.061)	-0.229 (-0.260, -0.198)	-0.166 (-0.197, -0.136)	-0.131 (-0.162, -0.100)
Pain in past 4 weeks	-0.054 (-0.072, -0.036)	-0.135 (-0.153, -0.117)	-0.098 (-0.116, -0.080)	-0.077 (-0.095, -0.059)
**Daily smoker**	**-0.010 (-0.030, 0.010)**	**-0.025 (-0.045, -0.006)**	**-0.018 (-0.038, 0.001)**	**-0.014 (-0.034, 0.005)**
**Number of drinks per week**	**0.006 (-0.002, 0.013)**	**0.015 (0.007, 0.022)**	**0.011 (0.003, 0.018)**	**0.008 (0.001, 0.016)**
Days exercise per week	0.055 (0.044, 0.067)	0.138 (0.127, 0.150)	0.101 (0.089, 0.112)	0.079 (0.068, 0.090)
Socioeconomic Outcomes				
Financial security	0.087 (0.067, 0.107)	0.217 (0.197, 0.237)	0.158 (0.138, 0.178)	0.124 (0.104, 0.144)
Material security	0.088 (0.067, 0.110)	0.221 (0.199, 0.242)	0.161 (0.139, 0.182)	0.126 (0.105, 0.148)
*Educational attainment (16+ years)	-0.000 (-0.000, 0.000)	-0.000 (-0.000, 0.000)	-0.000 (-0.000, 0.000)	-0.000 (-0.000, 0.000)
Currently employed	0.008 (0.004, 0.013)	0.020 (0.016, 0.025)	0.015 (0.010, 0.019)	0.012 (0.007, 0.016)
Financially comfortable/getting by	0.047 (0.039, 0.055)	0.117 (0.109, 0.125)	0.085 (0.077, 0.093)	0.067 (0.059, 0.075)
Own home	0.015 (0.010, 0.019)	0.037 (0.032, 0.041)	0.027 (0.022, 0.031)	0.021 (0.016, 0.026)

Note. The reliability corrected point estimate and standard errors are computed by dividing the primary result value by reliability (0.40, 0.55, and 0.70). Rows were the primary result is null but the corrected estimate 95% CI does not contain 0 are bolded.

Table S6.
Description of how all outcomes were coded/collapsed and analyzed.

Variable * (Supp. Outcome)	GFS Codebook Name	Question Statement	Response Options (From Codebook)	Recoding Approach/ How it will be analyzed
Human Flourishing				
Secure flourishing index	Secure Flourishing Index		LIFE_SAT, HAPPY, PHYSICAL_HLTH, MENTAL_HEALTH, WORTHWHILE, LIFE_PURPOSE, PROMOTE_GOOD, GIVE_UP, CONTENT, SAT_RELATNSHP, EXPENSES, WORRY_SAFETY	Mean of 12 items treated as approximately continuous
Flourishing index	Flourishing Index		LIFE_SAT, HAPPY, PHYSICAL_HLTH, MENTAL_HEALTH, WORTHWHILE, LIFE_PURPOSE, PROMOTE_GOOD, GIVE_UP, CONTENT, SAT_RELATNSHP	Mean of 10 items treated as approximately continuous
Happiness & life satisfaction*	Happiness & Life Satisfaction		HAPPY, LIFE_SAT	Mean of 2 items treated as approximately continuous
Physical & mental health*	Physical & Mental Health		PHYSICAL_HLTH, MENTAL_HEALTH	Mean of 2 items treated as approximately continuous
Meaning & purpose*	Meaning & Purpose		LIFE_PURPOSE, WORTHWHILE	Mean of 2 items treated as approximately continuous
Character & virtue*	Character & Virtue		PROMOTE_GOOD, GIVE_UP	Mean of 2 items treated as approximately continuous
Close social relationships	Subjective Social Connectedness		CONTENT, SAT_RELATNSHP	Mean of 2 items treated as approximately continuous
Financial & material security*	Financial & Material Security		EXPENSES, WORRY_SAFETY	Mean of 2 items treated as approximately continuous
*Psychological Well-Being**				
Happiness	HAPPY	In general, how happy or unhappy do you usually feel?	"-98 = (Saw, skipped) 0 = Extremely unhappy 10 = Extremely happy 98 = (DK) 99 = (Refused)"	treated as approximately continuous after removing the missing data flags.
Life satisfaction	LIFE_SAT	Overall, how satisfied are you with life as a whole these days?	"-98 = (Saw, skipped) 0 = Not at all satisfied with your life 10 = Completely satisfied with your life 98 = (DK) 99 = (Refused)"	treated as approximately continuous after removing the missing data flags
Current life evaluation	WB_TODAY	On which step of the ladder would you say you personally feel you stand at this time?	"-98 = (Saw, skipped) 0 = Worst possible 10 = Best possible 98 = (DK) 99 = (Refused)"	treated as approximately continuous after removing the missing data flags
Future life evaluation	WB_FIVEYRS	Just your best guess, on which step do you think you will stand in the future, say about five years from now?	"-98 = (Saw, skipped) 0 = Worst possible 10 = Best possible 98 = (DK) 99 = (Refused)"	treated as approximately continuous after removing the missing data flags
Optimism	EXPECT_GOOD	Overall, I expect more good things to happen to me than bad.	"-98 = (Saw, skipped) 0 = Strongly disagree 10 = Strongly agree 98 = (DK) 99 = (Refused)"	treated as approximately continuous after removing the missing data flags

Freedom to pursue what's important	FREEDOM	I have the freedom in my life to pursue the things that are most important to me.	"-98 = (Saw, skipped) 0 = Strongly disagree 10 = Strongly agree 98 = (DK) 99 = (Refused)"	treated as approximately continuous after removing the missing data flags
Inner peace	PEACE	In general, how often do you feel you are at peace with your thoughts and feelings?	"-98 = (Saw, skipped) 1 = Always 2 = Often 3 = Rarely 4 = Never 98 = (DK) 99 = (Refused)"	collapsed with Always/Often = 1 and Rarely/Never=0 as analyzed in Wave 1 analyses for comparability of results
Life balance	LIFE_BALANCE	In general, how often are the various aspects of your life in balance?	"-98 = (Saw, skipped) 1 = Always 2 = Often 3 = Rarely 4 = Never 98 = (DK) 99 = (Refused)"	collapsed with Always/Often = 1 and Rarely/Never=0 as analyzed in Wave 1 analyses for comparability of results
Sense of mastery	CAPABLE	How often do you feel very capable in most things you do in life?	"-98 = (Saw, skipped) 1 = Always 2 = Often 3 = Rarely 4 = Never 98 = (DK) 99 = (Refused)"	collapsed with Always/Often = 1 and Rarely/Never=0 as analyzed in Wave 1 analyses for comparability of results
Meaningful activities	WORTHWHILE	Overall, to what extent do you feel the things you do in your life are worthwhile?	"-98 = (Saw, skipped) 0 = Not at all worthwhile 10 = Completely worthwhile 98 = (DK) 99 = (Refused)"	treated as approximately continuous after removing the missing data flags
Understanding purpose	LIFE_PURPOSE	I understand my purpose in life.	"-98 = (Saw, skipped) 0 = Strongly disagree 10 = Strongly agree 98 = (DK) 99 = (Refused)"	treated as approximately continuous after removing the missing data flags
Self-rated mental health	MENTAL_HEALTH	How would you rate your overall mental health?	"-98 = (Saw, skipped) 0 = Poor mental health 10 = Excellent mental health 98 = (DK) 99 = (Refused)"	treated as approximately continuous after removing the missing data flags
Psychological Distress				
Traumatic distress	THREAT_LIFE	Think about the biggest threat to life you've ever witnessed or experienced first-hand during your life. In the past month, how much have you been bothered by this experience?	"-98 = (Saw, skipped) 1 = A lot 2 = Some 3 = Not very much 4 = None at all 98 = (DK) 99 = (Refused)"	collapsed with A lot/Some = 1 and Not very much/None at all=0 as analyzed in Wave 1 analyses for comparability of results
Depression symptoms composite	Depressive Symptoms		DEPRESSED, INTEREST	Sum DEPRESSED + INTEREST treated as approximately continuous after reverse coding: response options coded as: "3 = Nearly every day 2 = More than half the days 1 = Several days 0 = Not at all; Dichotomized as: 1 if sum is at least 3; 0 if sum is less than 3
Depression -- feel hopeless*	DEPRESSED	Over the last 2 weeks, how often have you been bothered by the following problems? Feeling down, depressed or hopeless?	"-98 = (Saw, skipped) 1 = Nearly every day 2 = More than half the days 3 = Several days 4 = Not at all 98 = (DK) 99 = (Refused)"	collapsed with Nearly every day/More than half the days = 1 and Not at all/Several days=0 as analyzed in Wave 1 analyses for comparability of results
Depression -- loss of interest*	INTEREST	Over the last 2 weeks, how	"-98 = (Saw, skipped) 1 = Nearly	collapsed with Nearly every day/More than half the days = 1; Not at all/Several days=0 as

		often have you been bothered by the following problems? Little interest or pleasure in doing things?	every day 2 = More than half the days 3 = Several days 4 = Not at all 98 = (DK) 99 = (Refused)"	analyzed in Wave 1 analyses for comparability of results
Anxiety symptoms composite	Anxiety Symptoms		FEEL_ANXIOUS, CONTROL_WORRY	Sum of FEEL_ANXIOUS + CONTROL_WORRY treated as approximately continuous after reverse coding: response options coded as: "3 = Nearly every day 2 = More than half the days 1 = Several days 0 = Not at all"; Dichotomized as: 1 if sum is at least 3; 0 if sum is less than 3
Anxiety -- feel on edge*	FEEL_ANXIOUS	Over the last 2 weeks, how often have you been bothered by the following problems? Feeling nervous, anxious or on edge?	"-98 = (Saw, skipped) 1 = Nearly every day 2 = More than half the days 3 = Several days 4 = Not at all 98 = (DK) 99 = (Refused)"	collapsed with Nearly every day/More than half the days = 1; Not at all/Several days=0 as analyzed in Wave 1 analyses for comparability of results
Anxiety -- cannot stop worrying*	CONTROL_WORRY	Over the last 2 weeks, how often have you been bothered by the following problems? Not being able to stop or control worrying?	"-98 = (Saw, skipped) 1 = Nearly every day 2 = More than half the days 3 = Several days 4 = Not at all 98 = (DK) 99 = (Refused)"	collapsed with Nearly every day/More than half the days = 1; Not at all/Several days=0 as analyzed in Wave 1 analyses for comparability of results
Suffering	SUFFERING	To what extent are you suffering? This can be any type of physical or mental suffering.	"-98 = (Saw, skipped) 1 = A lot 2 = Some 3 = Not very much 4 = Not at all 98 = (DK) 99 = (Refused)"	collapsed with A lot/Some = 1; Not very much/None at all=0 as analyzed in Wave 1 analyses for comparability of results
Social Well-Being				
Relationship contentment (e)	CONTENT	I am content with my friendships and relationships.	"-98 = (Saw, skipped) 0 = Strongly disagree 10 = Strongly agree 98 = (DK) 99 = (Refused)"	treated as approximately continuous after removing the missing data flags
Relationship satisfaction (e)	SAT_RELATNSHP	My relationships are as satisfying as I would want them to be.	"-98 = (Saw, skipped) 0 = Strongly disagree 10 = Strongly agree 98 = (DK) 99 = (Refused)"	treated as approximately continuous after removing the missing data flags
Social support	PEOPLE_HELP	If you were in trouble, how often could you count on people in your life, like relatives or friends, to help you whenever you need them?	"-98 = (Saw, skipped) 0 = Never 10 = Always 98 = (DK) 99 = (Refused)"	treated as approximately continuous after removing the missing data flags
Intimate/close friend	CLOSE_TO	Is there any one special person you know that you feel very close to? For example, someone you can confide in and share your feelings with. If you feel very close to more	"-98 = (Saw, skipped) 1 = Yes 2 = No 98 = (DK) 99 = (Refused)"	collapsed with Yes = 1; No = 0 as analyzed in Wave 1 analyses for comparability of results

		than one person, select Yes.		
Government approval	APPROVE_GOVT	How much do you approve or disapprove of the job performance of the national government of this country?	"-98 = (Saw, skipped) 1 = Strongly approve 2 = Somewhat approve 3 = Neither approve nor disapprove 4 = Somewhat disapprove 5 = Strongly disapprove 98 = (DK) 99 = (Refused)"	collapsed with Strongly approve/Somewhat approve = 1; Somewhat disapprove/Strongly disapprove/Neither=0 as analyzed in Wave 1 analyses for comparability of results
Say in government	SAY_IN_GOVT	Do you agree or disagree with the following statement? People like me have a say about what the government does.	"-98 = (Saw, skipped) 1 = Agree 2 = Disagree 3 = Unsure 99 = (Refused)"	collapsed with Agree = 1; Disagree/Unsure = 0 as analyzed in Wave 1 analyses for comparability of results
Belonging in country	BELONGING	How would you describe your sense of belonging in your country?	"-98 = (Saw, skipped) 0 = Very weak 10 = Very strong 98 = (DK) 99 = (Refused)"	treated as approximately continuous after removing the missing data flags
City/place satisfaction	SAT_LIVE	Are you satisfied or dissatisfied with the city or area where you live?	"-98 = (Saw, skipped) 1 = Satisfied 2 = Dissatisfied 3 = Unsure 99 = (Refused)"	collapsed with Satisfied = 1; Dissatisfied/Unsure = 0 as analyzed in Wave 1 analyses for comparability of results
Trust within country	TRUST_PEOPLE	How many people in this country trust one another?	"-98 = (Saw, skipped) 1 = All 2 = Most 3 = Some 4 = Not very many 5 = None 98 = (DK) 99 = (Refused)"	collapsed with All/Most/Some = 1; Not very many/None = 0 as analyzed in Wave 1 analyses for comparability of results
Social Participation				
Ever been married	MARITAL_STATUS	What is your current marital status?	"-98 = (Saw, skipped) 1 = Single/Never been married 2 = Married 3 = Separated 4 = Divorced 5 = Widowed 6 = Domestic partner 98 = (DK) 99 = (Refused)"	collapsed with Married/Separated/Divorced/Widowed = 1; Single/Never been married/Domestic partner = 0 as analyzed in Wave 1 analyses for comparability of results
Currently divorced	MARITAL_STATUS	What is your current marital status?	"-98 = (Saw, skipped) 1 = Single/Never been married 2 = Married 3 = Separated 4 = Divorced 5 = Widowed 6 = Domestic partner 98 = (DK) 99 = (Refused)"	collapsed with Divorced = 1; Single/Never been married/Separated/Married/Widowed/Domestic partner = 0 as analyzed in Wave 1 analyses for comparability of results
Number of children	NUM_CHILDREN	How many children under 18 years of age are now living in your household?	"-98 = (Saw, skipped) 0 = None 97 = 97+ 98 = (DK) 99 = (Refused)"	treated as approximately continuous after removing the missing data flags
Weekly+ community participation	GROUP_NOT_REL	How often do you participate in groups that are not religious, such	"-98 = (Saw, skipped) 1 = More than once a week 2 = Once a week 3 = One to three times	collapsed with More than once a week/Once a week = 1; One to three times a month/A few times a year/Never = 0 as analyzed in Wave 1 analyses for comparability of results

		as book clubs, sports, or political organizations?	a month 4 = A few times a year 5 = Never 98 = (DK) 99 = (Refused)"	
Weekly+ religious attendance	ATTEND_SVCS	How often do you attend religious services?	"-98 = (Saw, skipped) 1 = More than once a week 2 = Once a week 3 = One to three times a month 4 = A few times a year 5 = Never 98 = (DK) 99 = (Refused)"	collapsed with More than once a week/Once a week = 1; One to three times a month/A few times a year/Never = 0 as analyzed in Wave 1 analyses for comparability of results
Social Distress				
Loneliness	LONELY	How often do you feel lonely?	"-98 = (Saw, skipped) 0 = Always 10 = Never 98 = (DK) 99 = (Refused)"	Reversed coded so 0 = Never, 10 = Always; then treated as approximately continuous after removing the missing data flags
Perceived discrimination	DISCRIMINATED	How often do you feel discriminated against because of any group you are a part of? This might include discrimination because of your religion, political affiliation, race, gender, social class, sexual orientation, or involvement in civic organizations or community groups.	"-98 = (Saw, skipped) 1 = Always 2 = Often 3 = Rarely 4 = Never 98 = (DK) 99 = (Refused)"	collapsed with Always/Often = 1; Rarely/Never=0 as analyzed in Wave 1 analyses for comparability of results
Character & Prosocial Behavior				
Orientation to promote good	PROMOTE_GOOD	I always act to promote good in all circumstances, even in difficult and challenging situations.	"-98 = (Saw, skipped) 0 = Not true of you at all 10 = Completely true of you 98 = (DK) 99 = (Refused)"	treated as approximately continuous after removing the missing data flags
Delayed gratification	GIVE_UP	I am always able to give up some happiness now for greater happiness later.	"-98 = (Saw, skipped) 0 = Not true of you at all 10 = Completely true of you 98 = (DK) 99 = (Refused)"	treated as approximately continuous after removing the missing data flags
Hope	HOPE_FUTURE	Despite challenges, I always remain hopeful about the future.	"-98 = (Saw, skipped) 0 = Strongly disagree 10 = Strongly agree 98 = (DK) 99 = (Refused)"	treated as approximately continuous after removing the missing data flags
Gratitude	GRATEFUL	If I had to list everything that I felt grateful for, it would be a very long list.	"-98 = (Saw, skipped) 0 = Strongly disagree 10 = Strongly agree 98 = (DK) 99 = (Refused)"	treated as approximately continuous after removing the missing data flags
Showing love/care	SHOW_LOVE	How often do you show someone in your life that you love or care for them?	"-98 = (Saw, skipped) 0 = Never 10 = Always 98 = (DK) 99 = (Refused)"	treated as approximately continuous after removing the missing data flags

Forgivingness	FORGIVE	How often have you forgiven those who have hurt you?	"-98 = (Saw, skipped) 1 = Always 2 = Often 3 = Rarely 4 = Never 98 = (DK) 99 = (Refused)"	collapsed with Always/Often = 1; Rarely/Never=0 as analyzed in Wave 1 analyses for comparability of results
Charitable giving	DONATED	In the past month, have you donated money to a charity?	"-98 = (Saw, skipped) 1 = Yes 2 = No 98 = (DK) 99 = (Refused)"	collapsed with Yes = 1; No = 0 as analyzed in Wave 1 analyses for comparability of results
Helping strangers	HELP_STRANGER	In the past month, have you helped a stranger or someone you didn't know who needed help?	"-98 = (Saw, skipped) 1 = Yes 2 = No 98 = (DK) 99 = (Refused)"	collapsed with Yes = 1; No = 0 as analyzed in Wave 1 analyses for comparability of results
Volunteering	VOLUNTEERED	In the past month, have you volunteered your time to an organization?	"-98 = (Saw, skipped) 1 = Yes 2 = No 98 = (DK) 99 = (Refused)"	collapsed with Yes = 1; No = 0 as analyzed in Wave 1 analyses for comparability of results
Physical Health & Health Behavior				
Self-rated physical health	PHYSICAL_HLTH	In general, how would you rate your physical health?	"-98 = (Saw, skipped) 0 = Poor physical health 10 = Excellent physical health 98 = (DK) 99 = (Refused)"	treated as approximately continuous after removing the missing data flags
Health problems	HEALTH_PROB	Do you have any health problems that prevent you from doing any of the things people your age normally can do?	"-98 = (Saw, skipped) 1 = Yes 2 = no 98 = (DK) 99 = (Refused)"	collapsed with Yes = 1; No = 0 as analyzed in Wave 1 analyses for comparability of results
Pain in past 4 weeks	BODILY_PAIN	How much bodily pain have you had during the past 4 weeks?	"-98 = (Saw, skipped) 1 = A lot 2 = Some 3 = Not very much 4 = None at all 98 = (DK) 99 = (Refused)"	collapsed with A lot/Some = 1; Not very much/None at all=0 as analyzed in Wave 1 analyses for comparability of results
Daily smoker	CIGARETTES	About how many cigarettes do you smoke each day, if any?	"-98 = (Saw, skipped) 0 = None/Do not smoke 97 = 97+ 98 = (DK) 99 = (Refused)"	collapsed to greater a binary daily smoker indicator where 0 = does not smoke, 1 = smokes at least 1 cigarette per day as analyzed in Wave 1 analyses for comparability of results and to utilize the full sample. See smoking specific paper for more information on how smoker data can be analyzed to understand the effects of severity of smoking on wellbeing.
Number of drinks per week	DRINKS	Approximately how many full drinks of any kind of alcoholic beverage did you drink in the past seven days, if any? A full drink is a glass of wine, a can or bottle of beer, or a shot of hard liquor.	"-98 = (Saw, skipped) 0 = None/Do not drink alcoholic beverages 97 = 97+ 98 = (DK) 99 = (Refused)"	treated as approximately continuous after removing the missing data flags
Days exercise per week	DAYS_EXERCISE	On how many days did you exercise or engage in	"-98 = (Saw, skipped) 0 = 0 days 1 = 1 day 2 = 2 days 3 = 3 days 4 = 4	treated as approximately continuous after removing the missing data flags

		vigorous physical activities for 30 minutes or more in the past week?	days 5 = 5 days 6 = 6 days 7 = 7 days/Every day 98 = (DK) 99 = (Refused)"	
Socioeconomic Outcomes				
Financial security	EXPENSES	How often do you worry about being able to meet normal monthly living expenses?	"-98 = (Saw, skipped) 0 = Worry all of the time 10 = Do not ever worry 98 = (DK) 99 = (Refused)"	treated as approximately continuous after removing the missing data flags
Material security	WORRY_SAFETY	How often do you worry about safety, food, or housing?	"-98 = (Saw, skipped) 0 = Worry all of the time 10 = Do not ever worry 98 = (DK) 99 = (Refused)"	treated as approximately continuous after removing the missing data flags
Educational attainment (16+ years)	`EDUCATION_3	Highest Completed Level of Education (Three Levels)	"-98 = (Saw, skipped) 1 = Completed elementary education or less (up to 8 years of basic education) 2 = Secondary - 3 year TertiarySecondary education and some education beyond secondary education (9-15 years of education) 3 = Completed four years of education beyond high school and/or recieved a 4-year college degree. 98 = (DK) 99 = (RF)"	collapsed with 16+ years = 1; Up to 8/9 to 15 years = 0 as analyzed in Wave 1 analyses for comparability of results
Currently employed	EMPLOYMENT	Which of the following best describes your employment status? If you have more than one job, please answer for your primary job.	"-98 = (Saw, skipped) 1 = Employed for an employer 2 = Self-employed 3 = Retired 4 = Student 5 = Homemaker 6 = Unemployed and looking for a job 7 = None of these/Other 98 = (DK) 99 = (Refused)"	collapsed with Employed for an employer/Self-employed=1; Retired/Student/Homemaker/Unemployed and looking for a job/None of these/Other = 0 as analyzed in Wave 1 analyses for comparability of results
Financially comfortable/getting by	INCOME_FEELINGS	Which one of these phrases comes closest to your own feelings about your household's income these days?	"-98 = (Saw, skipped) 1 = Living comfortably on present income 2 = Getting by on present income 3 = Finding it difficult on present income 4 = Finding it very difficult on present income 98 = (DK) 99 = (Refused)"	collapsed with Living comfortably on present income/Getting by on present income = 1; Finding it difficult on present income/Finding it very difficult on present income = 0 as analyzed in Wave 1 analyses for comparability of results
Own home	OWN_RENT_HOME	Which of the following best describes the home you live in?	"-98 = (Saw, skipped) 1 = Someone in this household OWNS this home 2 = Someone in this household RENTS this home 3 = Both	collapsed with Someone in this household OWNS this home/Own/Both = 1; Someone in this household RENTS this home/Rent/Neither = 0 as analyzed in Wave 1 analyses for comparability of results

			4 = Neither 5 = Rent 6 = Own 7 = Something else 98 = (DK) 99 = (Refused)"	
Income -- top quintile	INCOME		Varies by country see GFS codebook for specific income brackets used in each country	Collapsed into Income Quintiles then dichotomized as being in the top quintile or not
Religion & Spirituality				
Religious/spiritual connection*	CONNECTED_REL	In general, how often do you feel connected to a religion or a form of spirituality?	"-98 = (Saw, skipped) 1 = Always 2 = Often 3 = Rarely 4 = Never 98 = (DK) 99 = (Refused)"	collapsed with Always/Often = 1; Rarely/Never=0 as analyzed in Wave 1 analyses for comparability of results
Belief in life after death*	AFTER_DEATH	Do you believe in life after death, or not?	"-98 = (Saw, skipped) 1 = Yes 2 = No 3 = Unsure 99 = (Refused)"	collapsed with Yes = 1; No/Unsure = 0 as analyzed in Wave 1 analyses for comparability of results
Transformative religious experience*	REL_EXPERIENC	Have you had a profound religious or spiritual awakening or experience that changed the direction of your life, or not?	"-98 = (Saw, skipped) 1 = Yes 2 = No 98 = (DK) 99 = (Refused)"	collapsed with Yes = 1; No = 0 as analyzed in Wave 1 analyses for comparability of results
Religious reading or listening*	SACRED_TEXTS	How often do you read or listen to sacred texts or other religious literature?	"-98 = (Saw, skipped) 1 = More than once a day 2 = About once a day 3 = Sometimes 4 = Never 98 = (DK) 99 = (Refused)"	collapsed with More than once a day/About once a day = 1; Sometimes/Never = 0 as analyzed in Wave 1 analyses for comparability of results
Prayer or meditation*	PRAY_MEDITATE	How often do you pray or meditate?	"-98 = (Saw, skipped) 1 = More than once a day 2 = About once a day 3 = Sometimes 4 = Never 98 = (DK) 99 = (Refused)"	collapsed with More than once a day/About once a day = 1; Sometimes/Never = 0 as analyzed in Wave 1 analyses for comparability of results
Belief in God/gods/spiritual forces*	BELIEVE_GOD	Do you believe in one God, more than one god, an impersonal spiritual force, or none of these?	"-98 = (Saw, skipped) 1 = One God 2 = More than one god 3 = An impersonal spiritual force 4 = None of these 5 = Unsure 99 = (Refused)"	collapsed with One God/More than one god/An impersonal spiritual force = 1; None of these/Unsure = 0 as analyzed in Wave 1 analyses for comparability of results
Religious centrality*	LIFE_APPROACH	My religious beliefs and practices are what really lie behind my whole approach to life.	"-98 = (Saw, skipped) 1 = Agree 2 = Disagree 3 = Not relevant 4 = Unsure 99 = (Refused)"	collapsed with Agree = 1; Disagree/Not relevant/Unsure = 0 as analyzed in Wave 1 analyses for comparability of results
Religious/spiritual comfort*	COMFORT_REL	I find strength or comfort in my religion or spirituality.	"-98 = (Saw, skipped) 1 = Agree 2 = Disagree 3 = Not relevant 4 = Unsure 99 = (Refused)"	collapsed with Agree = 1; Disagree/Not relevant/Unsure = 0 as analyzed in Wave 1 analyses for comparability of results
Feel loved by God*	LOVED_BY_GOD	I feel loved or cared for by God, the main god I worship, or the spiritual force that guides my life.	"-98 = (Saw, skipped) 1 = Agree 2 = Disagree 3 = Not relevant 4 = Unsure 99 = (Refused)"	collapsed with Agree = 1; Disagree/Not relevant/Unsure = 0 as analyzed in Wave 1 analyses for comparability of results

Feel punished by God*	GOD_PUNISH	I feel God, a god, or a spiritual force is punishing me.	"-98 = (Saw, skipped) 1 = Agree 2 = Disagree 3 = Not relevant 4 = Unsure 99 = (Refused)"	collapsed with Agree = 1; Disagree/Not relevant/Unsure = 0 as analyzed in Wave 1 analyses for comparability of results
Experienced religious criticism*	CRITICAL	People in my religious community are critical of me or my lifestyle.	"-98 = (Saw, skipped) 1 = Agree 2 = Disagree 3 = Not relevant 4 = Unsure 99 = (Refused)"	collapsed with Agree = 1; Disagree/Not relevant/Unsure = 0 as analyzed in Wave 1 analyses for comparability of results
Faith-sharing*	TELL_BELIEFS	I tell other people about my religion or spirituality even when they have different beliefs.	"-98 = (Saw, skipped) 1 = Agree 2 = Disagree 3 = Not relevant 4 = Unsure 99 = (Refused)"	collapsed with Agree = 1; Disagree/Not relevant/Unsure = 0 as analyzed in Wave 1 analyses for comparability of results

Figure S1.
Violin plot of time (in days) between taking the Annual Survey for respondents in each country. The distribution varies by country and is multi-modal with high variance within and between countries on the number of days between taking the survey.

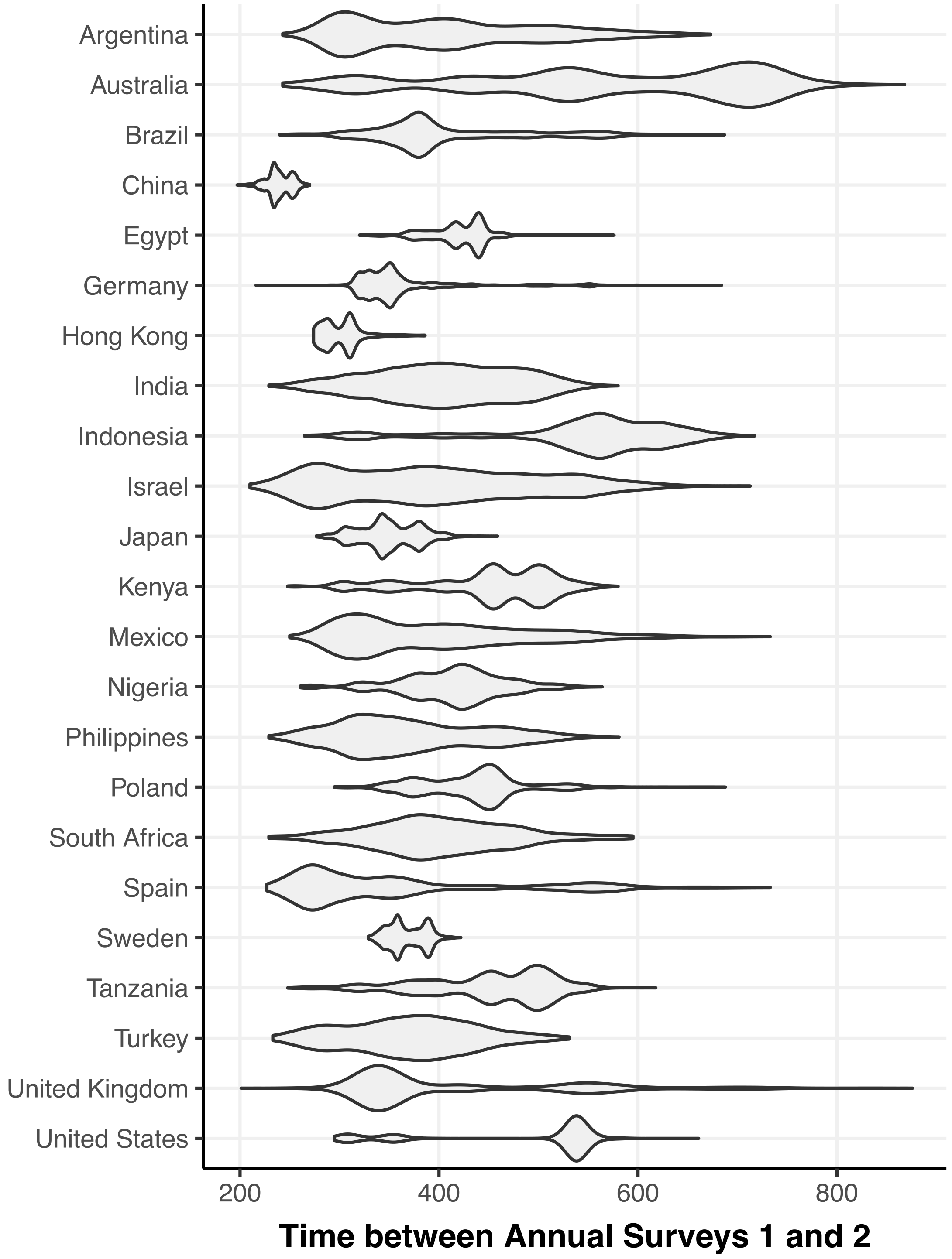

Figure S2.
Violin plot of time of years when respondents took each annual survey. This plot complements Figure S1 by providing the within wave variation in time of data collection by country.

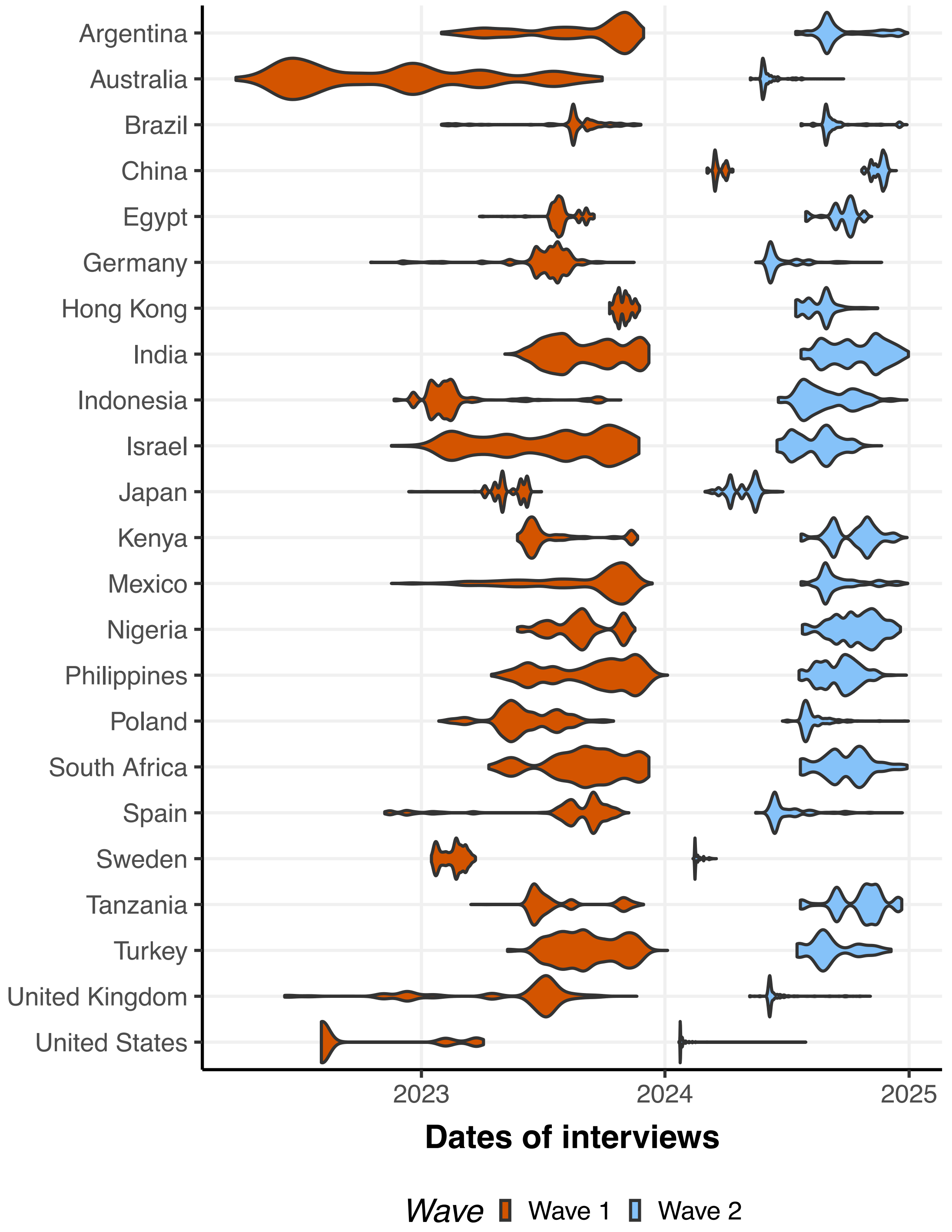

Figure S3.

Sensitivity of depression symptoms results to number of included principal components.

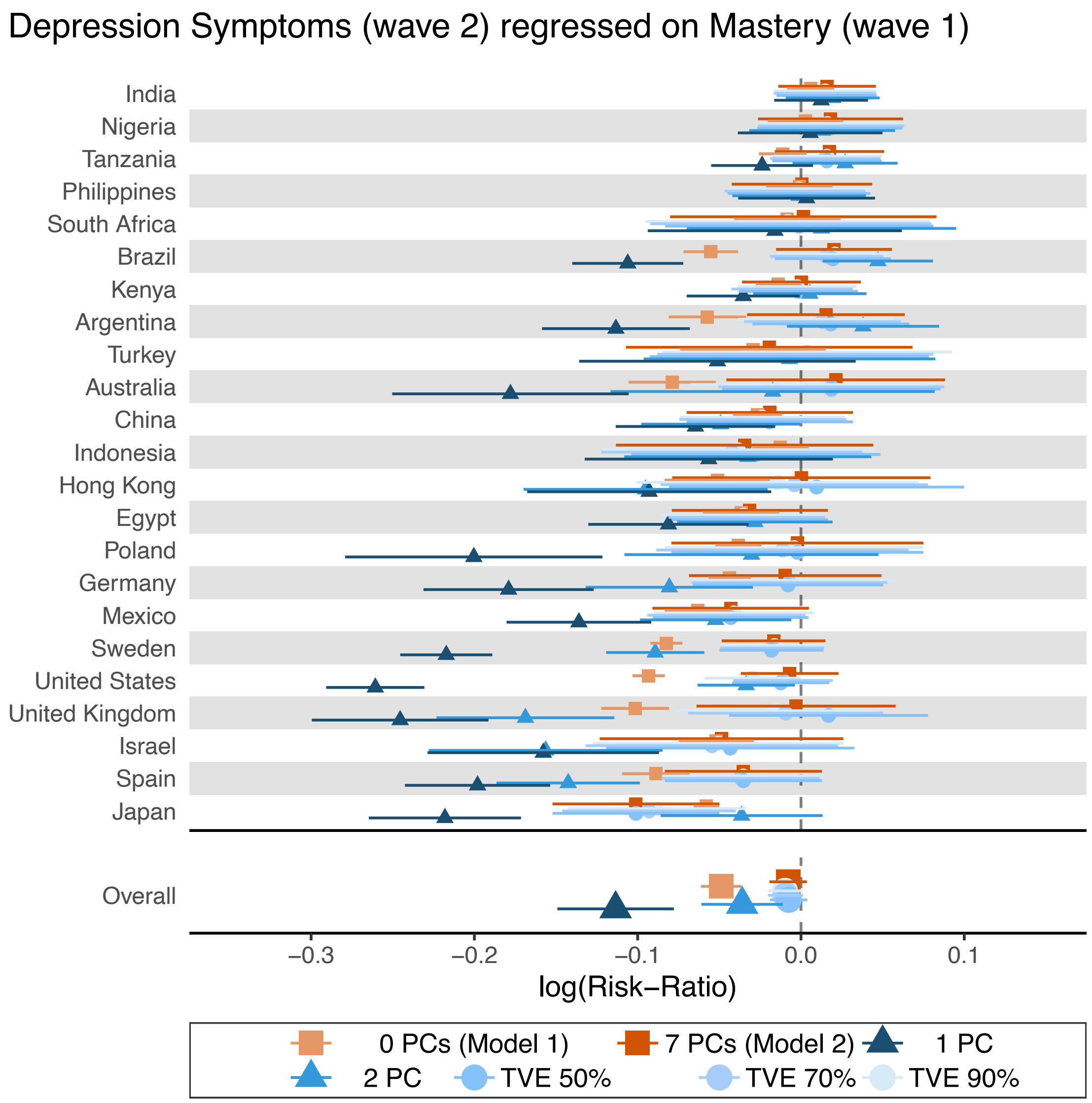

Supplement: Supplementary file 1 — Supplementary material 1 Contains the additional comments and technical details, and the following tables and figures: Table S1 Summary of top 20 eigenvalues from principal components; Table S2 Summary of top 20 principal components percent of variance explained by each component; Table S3 Benchmarking across a range of observed potential confounders to calibrate E-values for sensitivity analysis for unmeasured confounding; Table S4 Summary of penalized HETOP evaluation of which countries for each outcomes are identified with uniform and nonuniform differential item functioning; Table S5 Reliability corrected meta-analyzed estimates of association, Estimate (95% CI); Table S6 Description of how all outcomes were coded/collapsed and analyzed. Details on every included outcome: the item wording, response options, and how coded for analysis; Figure S1 Violin plot of time (in days) between taking the Annual Survey for respondents in each country; Figure S2 Violin plot of time of years when respondents took each annual survey; and Figure S3 Sensitivity of depression symptoms results to number of included principal components [file 44263_2026_287_MOESM1_ESM.pdf]
