# Supplementary material for: Coordinated outcome-wide analytic methodology for multi-wave analyses of the global flourishing study
Source: BMC Glob Public Health. 2026 Jun 2;4:55. doi: 10.1186/s44263-026-00287-6 (PMC13227717; doi:10.1186/s44263-026-00287-6)
Supplement: Supplementary file 2 — Supplementary material 2 Provides the results and analyses for traditional MG-CFA invariance testing of the PHQ-4 [file 44263_2026_287_MOESM2_ESM.pdf]

PHQ-4 Measurement Invariance

Country-wise factor analyses & Multi-Group CFA

Table of contents

1 Analysis and paper setup **3**
Theoretical measurement model . . . 3
Estimate country-specific means and covariances . . . 4

2 Separate Confirmatory Factor Analyses **8**
2.1 **Argentina** . . . 8
2.2 **Australia** . . . 13
2.3 **Brazil** . . . 18
2.4 **China** . . . 23
2.5 **Egypt** . . . 28
2.6 **Germany** . . . 33
2.7 **Hong Kong** . . . 38
2.8 **India** . . . 43
2.9 **Indonesia** . . . 48
2.10 **Israel** . . . 53
2.11 **Japan** . . . 58
2.12 **Kenya** . . . 63
2.13 **Mexico** . . . 68
2.14 **Nigeria** . . . 73
2.15 **Philippines** . . . 78
2.16 **Poland** . . . 83
2.17 **South Africa** . . . 88
2.18 **Spain** . . . 93
2.19 **Sweden** . . . 98
2.20 **Tanzania** . . . 103
2.21 **Turkey** . . . 108
2.22 **United Kingdom** . . . 113
2.23 **United States** . . . 118

3 Fit summary **123**
3.1 Composite reliability and AVE estimates . . . 124
3.2 Coefficient alpha . . . 127

4 Multi-Group CFA **150**
4.1 Covariance matrix input – allows for direct replication . . . 150
4.2 Person-level data input . . . 164

5 Summary and Conclusions **281**

```
knitr::opts_chunk$set(
  warning = FALSE, error = FALSE, message = FALSE,
  cache = TRUE, autodep = TRUE
)
options(width = 200)

library(Rglobalflourishing)
load_packages()
```

```
function (...)
capture_output(.f(...))
<bytecode: 0x11cd57bf0>
<environment: 0x11cd57e90>
```

```
library(lavaan)
library(semTools)
library(dynamic)
library(survey)
library(polycor)
options(survey.lonely.psu = "certainty")

df.raw <- readr::read_rds(here("data", "gfs_all_countries_wave2_raw_recoded.rds"))
DIF.items <- c('COUNTRY')
PHQ4.items <- c(
  "DEPRESSED_Y2",
  "INTEREST_Y2",
  "CONTROL_WORRY_Y2",
  "FEEL_ANXIOUS_Y2"
)

df.cc <- df.raw %>%
  mutate(
```

```
    n_resp = rowSums(across(all_of(PHQ4.items), ~!is.na(.x))),
    across(all_of(PHQ4.items),\(x){
      case_when(
        x == "1. Nearly every day" ~ 3,
        x == "2. More than half the days" ~ 2,
        x == "3. Several days" ~ 1,
        x == "4. Not at all" ~ 0
      )
    })
  ) |>
  filter(n_resp > 0)
```

1 Analysis and paper setup

For the factor analyses, these were conducted in two steps. First by fitting the 2 factor model used as the general structure of the PHQ-4. The fit of the model is provided including the residual correlation and modification indices. Secondly, we fit a series of increasingly restrictive multiple-group confirmatory factor analyses then compare the fit of these increasingly models. The factor scores from these increasingly restrictive models are then estimated for use elsewhere.

Theoretical measurement model

The current document serves as a technical dive into the configural invariance testing for the secure flourishing index (SFI) in Wave 2 of the Global Flourishing Study. The SFI is comprised of six domains each measured by two items.

1. Depressive Symptoms (feel depressed, loss of interest)
2. Anxiety Symptoms (feel anxious, loss of sense of control)

That is, in the lavaan like syntax:

```
mod <- "
dep =~ DEPRESSED_Y2 + INTEREST_Y2
anx =~ CONTROL_WORRY_Y2 + FEEL_ANXIOUS_Y2
"
```

Some evidence for equivalence of psychometric properties across countries exists (Lowe, et al., 2010). But, the invariance of psychometric propertoes has not been explored using the GFS dataset (wave 1 or wave 2) to the best of our knowledge.

Lowe, B., Wahl, I., Rose, M., Spitzer, C., Glaesmer, H., Wingenfeld, K., … & Brahler, E. (2010). A 4-item measure of depression and anxiety: Validation and standardization of the Patient Health Questionnaire-4 (PHQ-4) in the general population. *Journal of Affective Disorders, 122*(1-2), 86-95.

Estimate country-specific means and covariances

To get the input for the factor analyses, we first computed the survey weighted and design adjusted covariance matrix and mean vector for the PHQ-4 items. We used the survey package (Lumely, 2004) to get these matrices to use as input in lavaan for the factor analyses.

Lumley, T. (2004). Analysis of complex survey samples. *Journal of Statistical Software, 9*(1): 1-19

Lumley, T. (2010). *Complex Surveys: A Guide to Analysis Using R*. John Wiley and Sons.

Rosseel Y., Jorgensen T. D., De Wilde L. (2025). lavaan: Latent Variable Analysis. R package version 0.6-20. https://doi.org/10.32614/CRAN.package.lavaan

Rosseel, Y. (2012). lavaan: An R Package for Structural Equation Modeling. *Journal of Statistical Software, 48*(2), 1-36. https://doi.org/10.18637/jss.v048.i02

```
## compute the country-specific covariance matrices
COUNTRIES <- sort(as.character(unique(df.cc$COUNTRY)))
names(COUNTRIES) <- COUNTRIES

sample.stats <- map(COUNTRIES, \(x){

  tmp.dat = df.cc %>%
    filter(COUNTRY == x, !is.na(ANNUAL_WEIGHT_C2))

  ## SURVEY PACKAGE ESTIMATES
  svy.df <- svydesign(
    data = tmp.dat,
    ids = ~PSU,
    strata = ~STRATA,
    weights = ~ANNUAL_WEIGHT_C2
  )

  fit.mn <- svymean(reformulate(PHQ4.items), design = svy.df, na.rm=TRUE)
  fit.cov <- svyvar(reformulate(PHQ4.items), design = svy.df, na.rm=TRUE)
  fit.tbl <- lapply(PHQ4.items, FUN=function(x) svytable(reformulate(x), design = svy.df, ex
  fit.props <- lapply(PHQ4.items, FUN=function(x){
    df.tmp <- as.data.frame(svytable(reformulate(x), design = svy.df, na.rm=TRUE))
```

```r
  df.tmp$Freq <- df.tmp$Freq/sum(df.tmp$Freq)
  df.tmp$Var = x
  colnames(df.tmp) <- c("Category", "Freq", "Var")
  df.tmp$Category <- as.factor(df.tmp$Category)
  df.tmp
}) |>
  bind_rows()


## POLYCOR ESTIMATE W/ WCORR package
fit.polycorr <- matrix(0, ncol=length(PHQ4.items), nrow=length(PHQ4.items))
i <- 1; j<- 2
for(i in 1:length(PHQ4.items)){
  for(j in 1:length(PHQ4.items)){
    if(i == j){
      fit.polycorr[i,j] = 1.0
    } else {
      df <- na.omit(tmp.dat[,c(PHQ4.items[i], PHQ4.items[j], 'ANNUAL_WEIGHT_C2')])
      fit.polycorr[i,j] = wCorr::weightedCorr(
        as.numeric(df[,1,drop=TRUE]), as.numeric(df[,2,drop=TRUE]),
        weights=as.numeric(df[,3,drop=TRUE]),
        method = 'Polychoric', ML = TRUE)
    }
  }
}

## COMPUTE COVARIANCE MATRIX OF SAMPLE STATISTICS (thresholds + correlations)
## Need to estimate a CFA model to copy over the attributes
  fit <- cfa(
    model = mod
    , data = tmp.dat
    , std.lv = TRUE
    , ordered = TRUE
  )
## must use replicate weights
  sb.svy.df <- as.svrepdesign(svy.df,type="subbootstrap") ## use 'auto' if not sure what t
  # okay... auto is very slow for larger sample sizes...switching to "subbootstrap"

  fit.repweights <- withReplicates(sb.svy.df, theta = function(w, data){

      ## thresholds
      fit.th <- lapply(PHQ4.items, FUN=function(x){
```

```r
      y = data[[x]]
      yna = !is.na(y)

      data.frame(
        th = c(
          qnorm(sum(w[yna]*(y[yna] == 0))/sum(w[yna])),
          qnorm(sum(w[yna]*(y[yna] <= 1))/sum(w[yna])),
          qnorm(sum(w[yna]*(y[yna] <= 2))/sum(w[yna]))
        )
      )
      #df.tmp$Freq <-
      #df.tmp$Var = x
      #colnames(df.tmp) <- c("Category", "Freq", "Var")
      #df.tmp$Category <- as.factor(df.tmp$Category)
      #df.tmp
    }) |>
      bind_rows()
    fit.th <- as.numeric(fit.th[,1,drop=TRUE])
    fit.th <- fit.th[is.finite(fit.th)]

    ## polychoric correlations
    data$w <- w
    tmp.sdf <- svydesign(ids=~1, weights=~w, data=data)
    K <- length(PHQ4.items)
    fit.cor <- numeric(K*(K-1)/2)
    ii <- 1
    for(i in 1:K){
      for(j in 1:K){
        if(i < j){
           tmp.tbl <- svytable(reformulate(PHQ4.items[c(i,j)]),design=tmp.sdf)
           fit.cor[ii] <- polychor(tmp.tbl)
           ii = ii + 1
        }
      }
    }

    c(fit.th,fit.cor)
  })
#attributes(fit.polycorr)

sample.th <- fit.repweights[1:12]
attr(sample.th, "th.idx") <- lavInspect(fit, "th.idx")
```

```r
    sample.cov <- diag(1, ncol=4, nrow=4)
    sample.cov[lower.tri(sample.cov)] <- fit.repweights[13:18]
    sample.cov[upper.tri(sample.cov)] <- t(sample.cov)[upper.tri(sample.cov)]
    rownames(sample.cov) <- colnames(sample.cov) <- PHQ4.items
    sample.mean <- rep(0, 4)
    sample.nobs <- sum(tmp.dat$ANNUAL_WEIGHT_C2)
    NACOV <- as.matrix(attr(fit.repweights, "var"))*sample.nobs
    WLS.V <- DWLS.V <- NULL
    try({
      WLS.V <- solve(NACOV)
    })
    try({
      DWLS.V <- solve(diag(diag(NACOV)))
    })

  list(
    country = x,
    mean = fit.mn,
    cov = fit.cov,
    cat.counts = fit.tbl,
    cat.props = fit.props,
    ng = sum(tmp.dat$ANNUAL_WEIGHT_C2),
    sample.th = sample.th,
    sample.cov = sample.cov,
    sample.mean = sample.mean,
    sample.nobs = sample.nobs,
    NACOV = NACOV,
    WLS.V = WLS.V,
    DWLS.V = DWLS.V
  )

})

save(sample.stats, file=here("data", "replication_data.RData"))

LIST.CFA.FIT <- list()
```

2 Separate Confirmatory Factor Analyses

2.1 Argentina

2.1.1 Sample statistics

The following sample statistics, computed using the survey design adjusted estimates from the survey package, are used are input for the factor analyses. This facilitates replication of all analyses without the need to get the raw data from the Center for Open Science.

The reported correlation is the polychoric correlation.

```
ci <- 1
cur.country <- COUNTRIES[ci]
tb_sample_stats(sample.stats[[cur.country]])
```

Variable	DEPRESSED_Y2	INTEREST_Y2	CONTROL_WORRY_Y2	F
DEPRESSED_Y2	1.00	0.74	0.70	
INTEREST_Y2	0.74	1.00	0.57	
CONTROL_WORRY_Y2	0.70	0.57	1.00	
FEEL_ANXIOUS_Y2	0.70	0.60	0.75	
X				
Mean	0.91	1.09	1.03	
Standard.Deviation	1.00	1.00	1.00	
X.1				
Category	0.00	1.00	2.00	
DEPRESSED_Y2.1	45.44	30.40	12.91	
INTEREST_Y2.1	33.05	38.00	15.72	
CONTROL_WORRY_Y2.1	41.82	28.29	14.03	
FEEL_ANXIOUS_Y2.1	35.78	34.19	14.37	

2.1.2 Confirmatory factor analysis

First, all analyses are conducted by country to provide an in-depth assessment of the fit of the two-factor model without adding the complexity of multigroup models or invariance testing.

Secondly, these by-country analyses are conducted using the complex-survey adjusted estimates of the item covariance matrix and the item means. This allows for the a solid within country evaluation of the fit of the model while controlling for the complex sampling design.

The major disadvantage of this approach is that is does not account for the discrete nature of these analyses. One might be able to estimate the complex-survey adjusted

```
fit <- cfa(
  mod, std.lv = TRUE, ordered=TRUE
  , sample.cov = sample.stats[[cur.country]]$sample.cov
  , sample.mean = sample.stats[[cur.country]]$sample.mean
  , sample.nobs = sample.stats[[cur.country]]$sample.nobs
  , sample.th = sample.stats[[cur.country]]$sample.th
  , WLS.V = sample.stats[[cur.country]]$WLS.V
  , NACOV = sample.stats[[cur.country]]$NACOV
)

dynamic::cfaHB(fit)
```

```
Your DFI cutoffs:
            SRMR  RMSEA CFI   Magnitude
Level-0     0.004 0.038 0.999 NONE
Specificity 95%   95%   95%

Level-1     NONE  NONE  NONE  0.228
Sensitivity 1%    4%    1%

Empirical fit indices:
 Chi-Square  df p-value   SRMR   RMSEA    CFI
      1.156   1      NA  0.004   0.007      1
```

```
summary(fit, standardized=TRUE, ci=TRUE, fit.measure=TRUE)
```

```
lavaan 0.6-21 ended normally after 14 iterations

  Estimator                                       DWLS
  Optimization method                           NLMINB
  Number of model parameters                        17

  Number of observations                      2932.00000000153

Model Test User Model:
```

```
                                              Standard      Scaled
  Test Statistic                                 1.156       1.448
  Degrees of freedom                                 1           1
  P-value (Unknown)                                 NA       0.229
  Scaling correction factor                                  0.798
  Shift parameter                                            0.000
    simple second-order correction

Model Test Baseline Model:

  Test statistic                             18733.066    4377.656
  Degrees of freedom                                 6           6
  P-value                                           NA       0.000
  Scaling correction factor                                  4.281

User Model versus Baseline Model:

  Comparative Fit Index (CFI)                    1.000       1.000
  Tucker-Lewis Index (TLI)                       1.000       0.999

  Robust Comparative Fit Index (CFI)                         1.000
  Robust Tucker-Lewis Index (TLI)                            0.998

Root Mean Square Error of Approximation:

  RMSEA                                          0.007       0.012
  90 Percent confidence interval - lower         0.000       0.000
  90 Percent confidence interval - upper         0.050       0.053
  P-value H_0: RMSEA <= 0.050                    0.949       0.934
  P-value H_0: RMSEA >= 0.080                    0.001       0.001

  Robust RMSEA                                               0.028
  90 Percent confidence interval - lower                     0.000
  90 Percent confidence interval - upper                     0.121
  P-value H_0: Robust RMSEA <= 0.050                         0.498
  P-value H_0: Robust RMSEA >= 0.080                         0.248

Standardized Root Mean Square Residual:

  SRMR                                           0.004       0.004

Parameter Estimates:
```

```
  Parameterization                               Delta
  Standard errors                           Robust.sem
  Information                                 Expected
  Information saturated (h1) model        Unstructured

Latent Variables:
                   Estimate  Std.Err  z-value  P(>|z|) ci.lower ci.upper   Std.lv  Std.all
  dep =~
    DEPRESSED_Y2      0.938    0.015   62.263    0.000    0.909    0.968    0.938    0.938
    INTEREST_Y2       0.786    0.016   49.881    0.000    0.755    0.817    0.786    0.786
  anx =~
    CONTROL_WORRY_    0.860    0.017   51.393    0.000    0.828    0.893    0.860    0.860
    FEEL_ANXIOUS_Y    0.874    0.013   68.626    0.000    0.849    0.899    0.874    0.874

Covariances:
                   Estimate  Std.Err  z-value  P(>|z|) ci.lower ci.upper   Std.lv  Std.all
  dep ~~
    anx               0.858    0.023   36.914    0.000    0.812    0.903    0.858    0.858

Thresholds:
                   Estimate  Std.Err  z-value  P(>|z|) ci.lower ci.upper   Std.lv  Std.all
    DEPRESSED_Y2|1   -0.115    0.030   -3.764    0.000   -0.174   -0.055   -
0.115   -0.115
    DEPRESSED_Y2|2    0.701    0.029   24.424    0.000    0.645    0.758    0.701    0.701
    DEPRESSED_Y2|3    1.214    0.038   32.003    0.000    1.139    1.288    1.214    1.214
    INTEREST_Y2|t1   -0.439    0.032  -13.738    0.000   -0.501   -0.376   -
0.439   -0.439
    INTEREST_Y2|t2    0.555    0.033   17.051    0.000    0.491    0.619    0.555    0.555
    INTEREST_Y2|t3    1.116    0.043   26.103    0.000    1.032    1.199    1.116    1.116
    CONTROL_WORRY_   -0.207    0.030   -6.887    0.000   -0.265   -0.148   -
0.207   -0.207
    CONTROL_WORRY_    0.527    0.033   15.999    0.000    0.463    0.592    0.527    0.527
    CONTROL_WORRY_    1.000    0.042   23.761    0.000    0.918    1.083    1.000    1.000
    FEEL_ANXIOUS_Y   -0.364    0.027  -13.676    0.000   -0.417   -0.312   -
0.364   -0.364
    FEEL_ANXIOUS_Y    0.523    0.034   15.480    0.000    0.457    0.590    0.523    0.523
    FEEL_ANXIOUS_Y    1.009    0.040   25.287    0.000    0.930    1.087    1.009    1.009

Variances:
                   Estimate  Std.Err  z-value  P(>|z|) ci.lower ci.upper   Std.lv  Std.all
   .DEPRESSED_Y2      0.119                                0.119    0.119    0.119    0.119
   .INTEREST_Y2       0.382                                0.382    0.382    0.382    0.382
   .CONTROL_WORRY_    0.260                                0.260    0.260    0.260    0.260
```

```
   .FEEL_ANXIOUS_Y     0.236                                      0.236    0.236    0.236    0.236
    dep                1.000                                      1.000    1.000    1.000    1.000
    anx                1.000                                      1.000    1.000    1.000    1.000
```

```r
LIST.CFA.FIT[[cur.country]] <- fit
```

2.1.2.1 Residual correlations

Residual correlation greater than |0.05| are bolded.

```r
tb_residual_cor(fit)
```

Variable	DEPRESSED_Y2	INTEREST_Y2	CONTROL_WORRY_Y2	FE
DEPRESSED_Y2	0.00			
INTEREST_Y2	0.00	0.00		
CONTROL_WORRY_Y2	0.00	-0.01	0.00	
FEEL_ANXIOUS_Y2	-0.01	0.01	0.00	

2.1.2.2 Modification indices

```r
tb_mod_indices(fit, sort.=TRUE, maximum.number = 12)
```

lhs	op	rhs	mi	epc	sepc.lv
DEPRESSED_Y2	~~	FEEL_ANXIOUS_Y2	1.16	-0.03	-0.03
INTEREST_Y2	~~	FEEL_ANXIOUS_Y2	1.16	0.02	0.02
INTEREST_Y2	~~	CONTROL_WORRY_Y2	1.16	-0.02	-0.02
DEPRESSED_Y2	~~	CONTROL_WORRY_Y2	1.16	0.03	0.03

2.2 Australia

2.2.1 Sample statistics

The following sample statistics, computed using the survey design adjusted estimates from the survey package, are used are input for the factor analyses. This facilitates replication of all analyses without the need to get the raw data from the Center for Open Science.

```
ci <- ci + 1
cur.country <- names(sample.stats)[ci]
tb_sample_stats(sample.stats[[cur.country]])
```

Variable	DEPRESSED_Y2	INTEREST_Y2	CONTROL_WORRY_Y2	F
DEPRESSED_Y2	1.00	0.80	0.75	
INTEREST_Y2	0.80	1.00	0.61	
CONTROL_WORRY_Y2	0.75	0.61	1.00	
FEEL_ANXIOUS_Y2	0.74	0.59	0.85	
X				
Mean	0.78	0.83	0.82	
Standard.Deviation	1.00	1.00	1.00	
X.1				
Category	0.00	1.00	2.00	
DEPRESSED_Y2.1	46.99	34.99	10.60	
INTEREST_Y2.1	44.66	35.21	12.43	
CONTROL_WORRY_Y2.1	46.55	33.26	12.16	
FEEL_ANXIOUS_Y2.1	37.65	39.55	13.33	

2.2.2 Confirmatory factor analysis

```
fit <- cfa(
  mod, std.lv = TRUE, ordered=TRUE
  , sample.cov = sample.stats[[cur.country]]$sample.cov
  , sample.mean = sample.stats[[cur.country]]$sample.mean
```

```r
  , sample.nobs = sample.stats[[cur.country]]$sample.nobs
  , sample.th = sample.stats[[cur.country]]$sample.th
  , WLS.V = sample.stats[[cur.country]]$WLS.V
  , NACOV = sample.stats[[cur.country]]$NACOV
)

dynamic::cfaHB(fit)
```

```
Your DFI cutoffs:
            SRMR  RMSEA CFI  Magnitude
Level-0     0.003 0.036 1    NONE
Specificity 95%   95%   95%

Level-1     NONE  NONE  NONE 0.219
Sensitivity 2%    5%    1%

Empirical fit indices:
 Chi-Square  df p-value   SRMR   RMSEA    CFI
      0.763   1      NA  0.003       0      1
```

```r
summary(fit, standardized=TRUE, ci=TRUE, fit.measure=TRUE)
```

```
lavaan 0.6-21 ended normally after 16 iterations

  Estimator                                       DWLS
  Optimization method                           NLMINB
  Number of model parameters                        17

  Number of observations                      2582.00004721915

Model Test User Model:
                                              Standard      Scaled
  Test Statistic                                 0.763       1.461
  Degrees of freedom                                 1           1
  P-value (Unknown)                                 NA       0.227
  Scaling correction factor                                  0.522
  Shift parameter                                           -0.000
    simple second-order correction

Model Test Baseline Model:
```

```
  Test statistic                             27081.552    4125.199
  Degrees of freedom                                 6           6
  P-value                                           NA       0.000
  Scaling correction factor                                  6.568

User Model versus Baseline Model:

  Comparative Fit Index (CFI)                    1.000       1.000
  Tucker-Lewis Index (TLI)                       1.000       0.999

  Robust Comparative Fit Index (CFI)                         1.000
  Robust Tucker-Lewis Index (TLI)                            0.998

Root Mean Square Error of Approximation:

  RMSEA                                          0.000       0.013
  90 Percent confidence interval - lower         0.000       0.000
  90 Percent confidence interval - upper         0.049       0.056
  P-value H_0: RMSEA <= 0.050                    0.953       0.909
  P-value H_0: RMSEA >= 0.080                    0.001       0.002

  Robust RMSEA                                               0.033
  90 Percent confidence interval - lower                     0.000
  90 Percent confidence interval - upper                     0.139
  P-value H_0: Robust RMSEA <= 0.050                         0.439
  P-value H_0: Robust RMSEA >= 0.080                         0.332

Standardized Root Mean Square Residual:

  SRMR                                           0.003       0.003

Parameter Estimates:

  Parameterization                               Delta
  Standard errors                           Robust.sem
  Information                                 Expected
  Information saturated (h1) model        Unstructured

Latent Variables:
                   Estimate  Std.Err  z-value  P(>|z|) ci.lower ci.upper   Std.lv  Std.all
  dep =~
    DEPRESSED_Y2      0.999    0.014   69.039    0.000    0.971    1.027    0.999    0.999
    INTEREST_Y2       0.804    0.020   41.081    0.000    0.766    0.843    0.804    0.804
```

```
  anx =~
    CONTROL_WORRY_    0.931    0.012   77.605    0.000    0.907    0.954    0.931    0.931
    FEEL_ANXIOUS_Y    0.911    0.015   58.949    0.000    0.880    0.941    0.911    0.911

Covariances:
                   Estimate  Std.Err  z-value  P(>|z|) ci.lower ci.upper   Std.lv  Std.all
  dep ~~
    anx               0.809    0.024   34.382    0.000    0.763    0.855    0.809    0.809

Thresholds:
                   Estimate  Std.Err  z-value  P(>|z|) ci.lower ci.upper   Std.lv  Std.all
    DEPRESSED_Y2|1   -0.076    0.031   -2.436    0.015   -0.136   -0.015   -
0.076   -0.076
    DEPRESSED_Y2|2    0.914    0.037   24.813    0.000    0.842    0.987    0.914    0.914
    DEPRESSED_Y2|3    1.445    0.053   27.524    0.000    1.342    1.548    1.445    1.445
    INTEREST_Y2|t1   -0.134    0.034   -3.925    0.000   -0.201   -0.067   -
0.134   -0.134
    INTEREST_Y2|t2    0.837    0.038   21.976    0.000    0.762    0.912    0.837    0.837
    INTEREST_Y2|t3    1.426    0.056   25.496    0.000    1.316    1.535    1.426    1.426
    CONTROL_WORRY_   -0.087    0.030   -2.914    0.004   -0.145   -0.028   -
0.087   -0.087
    CONTROL_WORRY_    0.835    0.037   22.626    0.000    0.762    0.907    0.835    0.835
    CONTROL_WORRY_    1.403    0.054   25.900    0.000    1.297    1.509    1.403    1.403
    FEEL_ANXIOUS_Y   -0.315    0.032   -9.719    0.000   -0.378   -0.251   -
0.315   -0.315
    FEEL_ANXIOUS_Y    0.745    0.034   21.620    0.000    0.678    0.813    0.745    0.745
    FEEL_ANXIOUS_Y    1.312    0.054   24.101    0.000    1.205    1.419    1.312    1.312

Variances:
                   Estimate  Std.Err  z-value  P(>|z|) ci.lower ci.upper   Std.lv  Std.all
   .DEPRESSED_Y2      0.002                               0.002    0.002    0.002    0.002
   .INTEREST_Y2       0.353                               0.353    0.353    0.353    0.353
   .CONTROL_WORRY_    0.134                               0.134    0.134    0.134    0.134
   .FEEL_ANXIOUS_Y    0.171                               0.171    0.171    0.171    0.171
    dep               1.000                               1.000    1.000    1.000    1.000
    anx               1.000                               1.000    1.000    1.000    1.000
```

```
LIST.CFA.FIT[[cur.country]] <- fit
```

2.2.2.1 Residual correlations

Residual correlation greater than |0.05| are bolded.

```r
tb_residual_cor(fit)
```

Variable	DEPRESSED_Y2	INTEREST_Y2	CONTROL_WORRY_Y2	FE
DEPRESSED_Y2	0.00			
INTEREST_Y2	0.00	0.00		
CONTROL_WORRY_Y2	-0.00	0.01	0.00	
FEEL_ANXIOUS_Y2	0.00	-0.01	-0.00	

2.2.2.2 Modification indices

```r
tb_mod_indices(fit, sort.=TRUE, maximum.number = 12)
```

lhs	op	rhs	mi	epc	sepc.lv
INTEREST_Y2	~~	FEEL_ANXIOUS_Y2	0.76	-0.02	-0.02
INTEREST_Y2	~~	CONTROL_WORRY_Y2	0.76	0.02	0.02
DEPRESSED_Y2	~~	FEEL_ANXIOUS_Y2	0.76	0.02	0.02
DEPRESSED_Y2	~~	CONTROL_WORRY_Y2	0.76	-0.02	-0.02

2.3 Brazil

2.3.1 Sample statistics

The following sample statistics, computed using the survey design adjusted estimates from the survey package, are used are input for the factor analyses. This facilitates replication of all analyses without the need to get the raw data from the Center for Open Science.

```
ci <- ci + 1
cur.country <- names(sample.stats)[ci]
tb_sample_stats(sample.stats[[cur.country]])
```

Variable	DEPRESSED_Y2	INTEREST_Y2	CONTROL_WORRY_Y2
DEPRESSED_Y2	1.00	0.68	0.66
INTEREST_Y2	0.68	1.00	0.57
CONTROL_WORRY_Y2	0.66	0.57	1.00
FEEL_ANXIOUS_Y2	0.74	0.58	0.70
X			
Mean	0.95	1.20	1.23
Standard.Deviation	1.00	1.00	1.00
X.1			
Category	0.00	1.00	2.00
DEPRESSED_Y2.1	45.23	27.83	14.22
INTEREST_Y2.1	34.12	29.40	19.02
CONTROL_WORRY_Y2.1	34.67	28.82	15.14
FEEL_ANXIOUS_Y2.1	29.31	33.31	15.26

2.3.2 Confirmatory factor analysis

```
fit <- cfa(
  mod, std.lv = TRUE, ordered=TRUE
  , sample.cov = sample.stats[[cur.country]]$sample.cov
  , sample.mean = sample.stats[[cur.country]]$sample.mean
```

```r
  , sample.nobs = sample.stats[[cur.country]]$sample.nobs
  , sample.th = sample.stats[[cur.country]]$sample.th
  , WLS.V = sample.stats[[cur.country]]$WLS.V
  , NACOV = sample.stats[[cur.country]]$NACOV
)

dynamic::cfaHB(fit)
```

```
Your DFI cutoffs:
            SRMR  RMSEA CFI   Magnitude
Level-0     0.005 0.04  0.999 NONE
Specificity 95%   95%   95%

Level-1     NONE  NONE  NONE  0.263
Sensitivity 0%    4%    1%

Empirical fit indices:
 Chi-Square  df p-value   SRMR   RMSEA    CFI
      5.843   1      NA  0.009   0.034      1
```

```r
summary(fit, standardized=TRUE, ci=TRUE, fit.measure=TRUE)
```

```
lavaan 0.6-21 ended normally after 13 iterations

  Estimator                                         DWLS
  Optimization method                             NLMINB
  Number of model parameters                          17

  Number of observations                        4274.00000072482

Model Test User Model:
                                              Standard      Scaled
  Test Statistic                                 5.843       5.199
  Degrees of freedom                                 1           1
  P-value (Unknown)                                 NA       0.023
  Scaling correction factor                                  1.124
  Shift parameter                                            0.000
    simple second-order correction

Model Test Baseline Model:
```

```
  Test statistic                              23451.536    9067.915
  Degrees of freedom                                  6           6
  P-value                                            NA       0.000
  Scaling correction factor                                   2.586

User Model versus Baseline Model:

  Comparative Fit Index (CFI)                     1.000       1.000
  Tucker-Lewis Index (TLI)                        0.999       0.997

  Robust Comparative Fit Index (CFI)                          0.997
  Robust Tucker-Lewis Index (TLI)                             0.983

Root Mean Square Error of Approximation:

  RMSEA                                           0.034       0.031
  90 Percent confidence interval - lower          0.012       0.009
  90 Percent confidence interval - upper          0.062       0.060
  P-value H_0: RMSEA <= 0.050                     0.803       0.838
  P-value H_0: RMSEA >= 0.080                     0.002       0.002

  Robust RMSEA                                                0.079
  90 Percent confidence interval - lower                      0.024
  90 Percent confidence interval - upper                      0.151
  P-value H_0: Robust RMSEA <= 0.050                          0.162
  P-value H_0: Robust RMSEA >= 0.080                          0.582

Standardized Root Mean Square Residual:

  SRMR                                            0.009       0.009

Parameter Estimates:

  Parameterization                                Delta
  Standard errors                            Robust.sem
  Information                                  Expected
  Information saturated (h1) model          Unstructured

Latent Variables:
                   Estimate  Std.Err  z-value  P(>|z|) ci.lower ci.upper   Std.lv  Std.all
  dep =~
    DEPRESSED_Y2      0.919    0.012   77.250    0.000    0.896    0.942    0.919    0.919
    INTEREST_Y2       0.740    0.014   51.343    0.000    0.712    0.769    0.740    0.740
```

```
  anx =~
    CONTROL_WORRY_    0.807    0.017   47.808    0.000    0.774    0.840    0.807    0.807
    FEEL_ANXIOUS_Y    0.871    0.014   64.502    0.000    0.844    0.897    0.871    0.871

Covariances:
                   Estimate  Std.Err  z-value  P(>|z|) ci.lower ci.upper   Std.lv  Std.all
  dep ~~
    anx               0.914    0.013   67.735    0.000    0.888    0.941    0.914    0.914

Thresholds:
                   Estimate  Std.Err  z-value  P(>|z|) ci.lower ci.upper   Std.lv  Std.all
    DEPRESSED_Y2|1   -0.120    0.023   -5.176    0.000   -0.165   -0.074   -
0.120   -0.120
    DEPRESSED_Y2|2    0.615    0.027   22.747    0.000    0.562    0.668    0.615    0.615
    DEPRESSED_Y2|3    1.140    0.038   30.084    0.000    1.066    1.214    1.140    1.140
    INTEREST_Y2|t1   -0.409    0.028  -14.652    0.000   -0.464   -0.355   -
0.409   -0.409
    INTEREST_Y2|t2    0.345    0.027   12.658    0.000    0.292    0.399    0.345    0.345
    INTEREST_Y2|t3    0.936    0.038   24.926    0.000    0.862    1.010    0.936    0.936
    CONTROL_WORRY_   -0.394    0.020  -20.211    0.000   -0.433   -0.356   -
0.394   -0.394
    CONTROL_WORRY_    0.345    0.022   15.924    0.000    0.302    0.387    0.345    0.345
    CONTROL_WORRY_    0.794    0.033   24.001    0.000    0.729    0.858    0.794    0.794
    FEEL_ANXIOUS_Y   -0.544    0.031  -17.600    0.000   -0.605   -0.484   -
0.544   -0.544
    FEEL_ANXIOUS_Y    0.322    0.028   11.496    0.000    0.267    0.377    0.322    0.322
    FEEL_ANXIOUS_Y    0.768    0.034   22.655    0.000    0.702    0.835    0.768    0.768

Variances:
                   Estimate  Std.Err  z-value  P(>|z|) ci.lower ci.upper   Std.lv  Std.all
   .DEPRESSED_Y2      0.155                               0.155    0.155    0.155    0.155
   .INTEREST_Y2       0.452                               0.452    0.452    0.452    0.452
   .CONTROL_WORRY_    0.348                               0.348    0.348    0.348    0.348
   .FEEL_ANXIOUS_Y    0.241                               0.241    0.241    0.241    0.241
    dep               1.000                               1.000    1.000    1.000    1.000
    anx               1.000                               1.000    1.000    1.000    1.000
```

```
LIST.CFA.FIT[[cur.country]] <- fit
```

2.3.2.1 Residual correlations

Residual correlation greater than |0.05| are bolded.

```r
tb_residual_cor(fit)
```

Variable	DEPRESSED_Y2	INTEREST_Y2	CONTROL_WORRY_Y2	FE
DEPRESSED_Y2	0.00			
INTEREST_Y2	-0.00	0.00		
CONTROL_WORRY_Y2	-0.01	0.02	0.00	
FEEL_ANXIOUS_Y2	0.01	-0.01	-0.00	[illegible]

2.3.2.2 Modification indices

```r
tb_mod_indices(fit, sort.=TRUE, maximum.number = 12)
```

lhs	op	rhs	mi	epc	sepc.lv
DEPRESSED_Y2	~~	FEEL_ANXIOUS_Y2	5.84	0.06	0.06
INTEREST_Y2	~~	FEEL_ANXIOUS_Y2	5.84	-0.05	-0.05
DEPRESSED_Y2	~~	CONTROL_WORRY_Y2	5.84	-0.06	-0.06
INTEREST_Y2	~~	CONTROL_WORRY_Y2	5.84	0.05	0.05

2.4 China

2.4.1 Sample statistics

The following sample statistics, computed using the survey design adjusted estimates from the survey package, are used are input for the factor analyses. This facilitates replication of all analyses without the need to get the raw data from the Center for Open Science.

```r
ci <- ci + 1
cur.country <- names(sample.stats)[ci]
tb_sample_stats(sample.stats[[cur.country]])
```

Variable	DEPRESSED_Y2	INTEREST_Y2	CONTROL_WORRY_Y2	F
DEPRESSED_Y2	1.00	0.54	0.72	
INTEREST_Y2	0.54	1.00	0.54	
CONTROL_WORRY_Y2	0.72	0.54	1.00	
FEEL_ANXIOUS_Y2	0.72	0.48	0.78	
X				
Mean	0.73	0.99	0.61	
Standard.Deviation	1.00	1.00	1.00	
X.1				
Category	0.00	1.00	2.00	
DEPRESSED_Y2.1	39.99	48.97	9.23	
INTEREST_Y2.1	29.93	44.78	21.55	
CONTROL_WORRY_Y2.1	53.37	34.32	9.98	
FEEL_ANXIOUS_Y2.1	37.17	50.68	9.93	

2.4.2 Confirmatory factor analysis

```r
fit <- cfa(
  mod, std.lv = TRUE, ordered=TRUE
  , sample.cov = sample.stats[[cur.country]]$sample.cov
  , sample.mean = sample.stats[[cur.country]]$sample.mean
```

```r
  , sample.nobs = sample.stats[[cur.country]]$sample.nobs
  , sample.th = sample.stats[[cur.country]]$sample.th
  , WLS.V = sample.stats[[cur.country]]$WLS.V
  , NACOV = sample.stats[[cur.country]]$NACOV
)

dynamic::cfaHB(fit)
```

```
Your DFI cutoffs:
            SRMR  RMSEA CFI   Magnitude
Level-0     0.005 0.038 0.999 NONE
Specificity 95%   95%   95%

Level-1     NONE  NONE  NONE  0.376
Sensitivity 0%    5%    1%

Empirical fit indices:
 Chi-Square  df p-value   SRMR   RMSEA    CFI
     11.197   1      NA  0.011   0.047      1
```

```r
summary(fit, standardized=TRUE, ci=TRUE, fit.measure=TRUE)
```

```
lavaan 0.6-21 ended normally after 15 iterations

  Estimator                                       DWLS
  Optimization method                           NLMINB
  Number of model parameters                        17

  Number of observations                      4544.00000002019

Model Test User Model:
                                              Standard      Scaled
  Test Statistic                                11.197      10.744
  Degrees of freedom                                 1           1
  P-value (Unknown)                                 NA       0.001
  Scaling correction factor                                  1.042
  Shift parameter                                            0.000
    simple second-order correction

Model Test Baseline Model:
```

```
  Test statistic                               37008.511   12067.662
  Degrees of freedom                                   6           6
  P-value                                             NA       0.000
  Scaling correction factor                                    3.067

User Model versus Baseline Model:

  Comparative Fit Index (CFI)                      1.000       0.999
  Tucker-Lewis Index (TLI)                         0.998       0.995

  Robust Comparative Fit Index (CFI)                           0.996
  Robust Tucker-Lewis Index (TLI)                              0.975

Root Mean Square Error of Approximation:

  RMSEA                                            0.047       0.046
  90 Percent confidence interval - lower           0.025       0.024
  90 Percent confidence interval - upper           0.074       0.073
  P-value H_0: RMSEA <= 0.050                      0.510       0.537
  P-value H_0: RMSEA >= 0.080                      0.020       0.017

  Robust RMSEA                                                 0.097
  90 Percent confidence interval - lower                       0.051
  90 Percent confidence interval - upper                       0.153
  P-value H_0: Robust RMSEA <= 0.050                           0.047
  P-value H_0: Robust RMSEA >= 0.080                           0.759

Standardized Root Mean Square Residual:

  SRMR                                             0.011       0.011

Parameter Estimates:

  Parameterization                                 Delta
  Standard errors                             Robust.sem
  Information                                   Expected
  Information saturated (h1) model          Unstructured

Latent Variables:
                   Estimate  Std.Err  z-value  P(>|z|) ci.lower ci.upper   Std.lv  Std.all
  dep =~
    DEPRESSED_Y2      0.870    0.013   65.419    0.000    0.844    0.896    0.870    0.870
    INTEREST_Y2       0.620    0.014   42.795    0.000    0.592    0.648    0.620    0.620
```

```
  anx =~
    CONTROL_WORRY_    0.896    0.009  102.918    0.000    0.879    0.913    0.896    0.896
    FEEL_ANXIOUS_Y    0.874    0.008  104.230    0.000    0.857    0.890    0.874    0.874

Covariances:
                   Estimate  Std.Err  z-value  P(>|z|) ci.lower ci.upper   Std.lv  Std.all
  dep ~~
    anx               0.935    0.014   65.099    0.000    0.906    0.963    0.935    0.935

Thresholds:
                   Estimate  Std.Err  z-value  P(>|z|) ci.lower ci.upper   Std.lv  Std.all
    DEPRESSED_Y2|1   -0.254    0.019  -13.301    0.000   -0.291   -0.216   -
0.254   -0.254
    DEPRESSED_Y2|2    1.225    0.025   48.730    0.000    1.176    1.274    1.225    1.225
    DEPRESSED_Y2|3    2.097    0.048   44.150    0.000    2.004    2.190    2.097    2.097
    INTEREST_Y2|t1   -0.526    0.019  -27.677    0.000   -0.564   -0.489   -
0.526   -0.526
    INTEREST_Y2|t2    0.665    0.026   25.693    0.000    0.615    0.716    0.665    0.665
    INTEREST_Y2|t3    1.781    0.036   50.088    0.000    1.711    1.851    1.781    1.781
    CONTROL_WORRY_    0.084    0.022    3.822    0.000    0.041    0.128    0.084    0.084
    CONTROL_WORRY_    1.159    0.030   38.511    0.000    1.100    1.218    1.159    1.159
    CONTROL_WORRY_    1.989    0.040   50.138    0.000    1.911    2.066    1.989    1.989
    FEEL_ANXIOUS_Y   -0.327    0.023  -14.541    0.000   -0.371   -0.283   -
0.327   -0.327
    FEEL_ANXIOUS_Y    1.168    0.028   41.608    0.000    1.113    1.223    1.168    1.168
    FEEL_ANXIOUS_Y    2.010    0.044   45.618    0.000    1.924    2.097    2.010    2.010

Variances:
                   Estimate  Std.Err  z-value  P(>|z|) ci.lower ci.upper   Std.lv  Std.all
   .DEPRESSED_Y2      0.243                               0.243    0.243    0.243    0.243
   .INTEREST_Y2       0.616                               0.616    0.616    0.616    0.616
   .CONTROL_WORRY_    0.197                               0.197    0.197    0.197    0.197
   .FEEL_ANXIOUS_Y    0.237                               0.237    0.237    0.237    0.237
    dep               1.000                               1.000    1.000    1.000    1.000
    anx               1.000                               1.000    1.000    1.000    1.000
```

```r
LIST.CFA.FIT[[cur.country]] <- fit
```

2.4.2.1 Residual correlations

Residual correlation greater than |0.05| are bolded.

```r
tb_residual_cor(fit)
```

Variable	DEPRESSED_Y2	INTEREST_Y2	CONTROL_WORRY_Y2	FE
DEPRESSED_Y2	0.00			
INTEREST_Y2	0.00	0.00		
CONTROL_WORRY_Y2	-0.01	0.02	0.00	
FEEL_ANXIOUS_Y2	0.01	-0.02	0.00	

2.4.2.2 Modification indices

```r
tb_mod_indices(fit, sort.=TRUE, maximum.number = 12)
```

lhs	op	rhs	mi	epc	sepc.lv
INTEREST_Y2	~~	CONTROL_WORRY_Y2	11.19	0.06	0.06
INTEREST_Y2	~~	FEEL_ANXIOUS_Y2	11.19	-0.06	-0.06
DEPRESSED_Y2	~~	FEEL_ANXIOUS_Y2	11.19	0.08	0.08
DEPRESSED_Y2	~~	CONTROL_WORRY_Y2	11.19	-0.08	-0.08

2.5 Egypt

2.5.1 Sample statistics

The following sample statistics, computed using the survey design adjusted estimates from the survey package, are used are input for the factor analyses. This facilitates replication of all analyses without the need to get the raw data from the Center for Open Science.

```
ci <- ci + 1
cur.country <- names(sample.stats)[ci]
tb_sample_stats(sample.stats[[cur.country]])
```

Variable	DEPRESSED_Y2	INTEREST_Y2	CONTROL_WORRY_Y2	F
DEPRESSED_Y2	1.00	0.48	0.61	
INTEREST_Y2	0.48	1.00	0.44	
CONTROL_WORRY_Y2	0.61	0.44	1.00	
FEEL_ANXIOUS_Y2	0.60	0.44	0.71	
X				
Mean	1.08	1.10	1.14	
Standard.Deviation	1.00	1.00	1.00	
X.1				
Category	0.00	1.00	2.00	
DEPRESSED_Y2.1	37.38	33.38	12.64	
INTEREST_Y2.1	32.32	39.56	14.29	
CONTROL_WORRY_Y2.1	34.67	33.71	14.41	
FEEL_ANXIOUS_Y2.1	21.08	37.62	13.49	

2.5.2 Confirmatory factor analysis

```
fit <- cfa(
  mod, std.lv = TRUE, ordered=TRUE
  , sample.cov = sample.stats[[cur.country]]$sample.cov
  , sample.mean = sample.stats[[cur.country]]$sample.mean
```

```r
  , sample.nobs = sample.stats[[cur.country]]$sample.nobs
  , sample.th = sample.stats[[cur.country]]$sample.th
  , WLS.V = sample.stats[[cur.country]]$WLS.V
  , NACOV = sample.stats[[cur.country]]$NACOV
)

dynamic::cfaHB(fit)
```

```
Your DFI cutoffs:
            SRMR  RMSEA CFI   Magnitude
Level-0     0.006 0.038 0.999 NONE
Specificity 95%   95%   95%

Level-1     NONE  NONE  NONE  0.42
Sensitivity 1%    5%    1%

Empirical fit indices:
 Chi-Square  df p-value   SRMR   RMSEA    CFI
      0.082   1      NA  0.001       0      1
```

```r
summary(fit, standardized=TRUE, ci=TRUE, fit.measure=TRUE)
```

```
lavaan 0.6-21 ended normally after 13 iterations

  Estimator                                       DWLS
  Optimization method                           NLMINB
  Number of model parameters                        17

  Number of observations                      3040.00004437456

Model Test User Model:
                                              Standard      Scaled
  Test Statistic                                 0.082       0.095
  Degrees of freedom                                 1           1
  P-value (Unknown)                                 NA       0.758
  Scaling correction factor                                  0.872
  Shift parameter                                           -0.000
    simple second-order correction

Model Test Baseline Model:
```

```
  Test statistic                              12857.131    3706.436
  Degrees of freedom                                  6           6
  P-value                                            NA       0.000
  Scaling correction factor                                   3.470

User Model versus Baseline Model:

  Comparative Fit Index (CFI)                     1.000       1.000
  Tucker-Lewis Index (TLI)                        1.000       1.001

  Robust Comparative Fit Index (CFI)                          1.000
  Robust Tucker-Lewis Index (TLI)                             1.004

Root Mean Square Error of Approximation:

  RMSEA                                           0.000       0.000
  90 Percent confidence interval - lower          0.000       0.000
  90 Percent confidence interval - upper          0.032       0.033
  P-value H_0: RMSEA <= 0.050                     0.994       0.994
  P-value H_0: RMSEA >= 0.080                     0.000       0.000

  Robust RMSEA                                                0.000
  90 Percent confidence interval - lower                      0.000
  90 Percent confidence interval - upper                      0.060
  P-value H_0: Robust RMSEA <= 0.050                          0.918
  P-value H_0: Robust RMSEA >= 0.080                          0.015

Standardized Root Mean Square Residual:

  SRMR                                            0.001       0.001

Parameter Estimates:

  Parameterization                                Delta
  Standard errors                           Robust.sem
  Information                                 Expected
  Information saturated (h1) model        Unstructured

Latent Variables:
                   Estimate  Std.Err  z-value  P(>|z|) ci.lower ci.upper   Std.lv  Std.all
  dep =~
    DEPRESSED_Y2      0.820    0.023   35.442    0.000    0.775    0.866    0.820    0.820
    INTEREST_Y2       0.588    0.022   26.587    0.000    0.545    0.632    0.588    0.588
```

```
  anx =~
    CONTROL_WORRY_    0.847    0.015   55.951    0.000    0.817    0.877    0.847    0.847
    FEEL_ANXIOUS_Y    0.836    0.020   42.102    0.000    0.797    0.875    0.836    0.836

Covariances:
                   Estimate  Std.Err  z-value  P(>|z|) ci.lower ci.upper   Std.lv  Std.all
  dep ~~
    anx               0.882    0.024   36.222    0.000    0.834    0.929    0.882    0.882

Thresholds:
                   Estimate  Std.Err  z-value  P(>|z|) ci.lower ci.upper   Std.lv  Std.all
    DEPRESSED_Y2|1   -0.322    0.032  -10.114    0.000   -0.384   -0.259   -
0.322   -0.322
    DEPRESSED_Y2|2    0.546    0.037   14.575    0.000    0.473    0.620    0.546    0.546
    DEPRESSED_Y2|3    0.970    0.033   29.491    0.000    0.906    1.035    0.970    0.970
    INTEREST_Y2|t1   -0.459    0.027  -17.134    0.000   -0.511   -0.406   -
0.459   -0.459
    INTEREST_Y2|t2    0.579    0.032   18.176    0.000    0.517    0.642    0.579    0.579
    INTEREST_Y2|t3    1.088    0.041   26.448    0.000    1.007    1.169    1.088    1.088
    CONTROL_WORRY_   -0.394    0.033  -12.068    0.000   -0.458   -0.330   -
0.394   -0.394
    CONTROL_WORRY_    0.478    0.036   13.178    0.000    0.407    0.550    0.478    0.478
    CONTROL_WORRY_    0.946    0.045   20.805    0.000    0.857    1.035    0.946    0.946
    FEEL_ANXIOUS_Y   -0.804    0.035  -23.066    0.000   -0.872   -0.735   -
0.804   -0.804
    FEEL_ANXIOUS_Y    0.220    0.032    6.776    0.000    0.156    0.283    0.220    0.220
    FEEL_ANXIOUS_Y    0.588    0.034   17.178    0.000    0.521    0.655    0.588    0.588

Variances:
                   Estimate  Std.Err  z-value  P(>|z|) ci.lower ci.upper   Std.lv  Std.all
   .DEPRESSED_Y2      0.327                               0.327    0.327    0.327    0.327
   .INTEREST_Y2       0.654                               0.654    0.654    0.654    0.654
   .CONTROL_WORRY_    0.282                               0.282    0.282    0.282    0.282
   .FEEL_ANXIOUS_Y    0.301                               0.301    0.301    0.301    0.301
    dep               1.000                               1.000    1.000    1.000    1.000
    anx               1.000                               1.000    1.000    1.000    1.000
```

```
LIST.CFA.FIT[[cur.country]] <- fit
```

2.5.2.1 Residual correlations

Residual correlation greater than |0.05| are bolded.

```r
tb_residual_cor(fit)
```

Variable	DEPRESSED_Y2	INTEREST_Y2	CONTROL_WORRY_Y2	FE
DEPRESSED_Y2	0.00			
INTEREST_Y2	0.00	0.00		
CONTROL_WORRY_Y2	0.00	-0.00	0.00	
FEEL_ANXIOUS_Y2	-0.00	0.00	0.00	

2.5.2.2 Modification indices

```r
tb_mod_indices(fit, sort.=TRUE, maximum.number = 12)
```

lhs	op	rhs	mi	epc	sepc.lv
DEPRESSED_Y2	~~	FEEL_ANXIOUS_Y2	0.08	-0.01	-0.01
INTEREST_Y2	~~	FEEL_ANXIOUS_Y2	0.08	0.01	0.01
DEPRESSED_Y2	~~	CONTROL_WORRY_Y2	0.08	0.01	0.01
INTEREST_Y2	~~	CONTROL_WORRY_Y2	0.08	-0.01	-0.01

2.6 Germany

2.6.1 Sample statistics

The following sample statistics, computed using the survey design adjusted estimates from the survey package, are used are input for the factor analyses. This facilitates replication of all analyses without the need to get the raw data from the Center for Open Science.

```
ci <- ci + 1
cur.country <- names(sample.stats)[ci]
tb_sample_stats(sample.stats[[cur.country]])
```

Variable	DEPRESSED_Y2	INTEREST_Y2	CONTROL_WORRY_Y2	F[illegible]
DEPRESSED_Y2	1.00	0.80	0.71	[illegible]
INTEREST_Y2	0.80	1.00	0.65	[illegible]
CONTROL_WORRY_Y2	0.71	0.65	1.00	[illegible]
FEEL_ANXIOUS_Y2	0.70	0.64	0.67	[illegible]
X				
Mean	0.64	0.57	0.73	[illegible]
Standard.Deviation	1.00	1.00	1.00	[illegible]
X.1				
Category	0.00	1.00	2.00	[illegible]
DEPRESSED_Y2.1	54.09	32.50	8.64	[illegible]
INTEREST_Y2.1	59.94	27.18	8.70	[illegible]
CONTROL_WORRY_Y2.1	50.82	31.93	10.93	[illegible]
FEEL_ANXIOUS_Y2.1	44.55	39.84	10.69	[illegible]

2.6.2 Confirmatory factor analysis

```
fit <- cfa(
  mod, std.lv = TRUE, ordered=TRUE
  , sample.cov = sample.stats[[cur.country]]$sample.cov
  , sample.mean = sample.stats[[cur.country]]$sample.mean
```

```r
  , sample.nobs = sample.stats[[cur.country]]$sample.nobs
  , sample.th = sample.stats[[cur.country]]$sample.th
  , WLS.V = sample.stats[[cur.country]]$WLS.V
  , NACOV = sample.stats[[cur.country]]$NACOV
)

dynamic::cfaHB(fit)
```

```
Your DFI cutoffs:
            SRMR  RMSEA CFI   Magnitude
Level-0     0.004 0.038 0.999 NONE
Specificity 95%   95%   95%

Level-1     NONE  NONE  NONE  0.19
Sensitivity 5%    5%    2%

Empirical fit indices:
 Chi-Square  df p-value   SRMR   RMSEA    CFI
      0.108   1      NA  0.001       0      1
```

```r
summary(fit, standardized=TRUE, ci=TRUE, fit.measure=TRUE)
```

```
lavaan 0.6-21 ended normally after 16 iterations

  Estimator                                       DWLS
  Optimization method                           NLMINB
  Number of model parameters                        17

  Number of observations                      5529.00000403925

Model Test User Model:
                                              Standard      Scaled
  Test Statistic                                 0.108       0.093
  Degrees of freedom                                 1           1
  P-value (Unknown)                                 NA       0.761
  Scaling correction factor                                  1.161
  Shift parameter                                            0.000
    simple second-order correction

Model Test Baseline Model:
```

```
  Test statistic                              48503.795   10440.741
  Degrees of freedom                                  6           6
  P-value                                            NA       0.000
  Scaling correction factor                                   4.647

User Model versus Baseline Model:

  Comparative Fit Index (CFI)                     1.000       1.000
  Tucker-Lewis Index (TLI)                        1.000       1.001

  Robust Comparative Fit Index (CFI)                          1.000
  Robust Tucker-Lewis Index (TLI)                             1.003

Root Mean Square Error of Approximation:

  RMSEA                                           0.000       0.000
  90 Percent confidence interval - lower          0.000       0.000
  90 Percent confidence interval - upper          0.025       0.024
  P-value H_0: RMSEA <= 0.050                     1.000       1.000
  P-value H_0: RMSEA >= 0.080                     0.000       0.000

  Robust RMSEA                                                0.000
  90 Percent confidence interval - lower                      0.000
  90 Percent confidence interval - upper                      0.067
  P-value H_0: Robust RMSEA <= 0.050                          0.899
  P-value H_0: Robust RMSEA >= 0.080                          0.026

Standardized Root Mean Square Residual:

  SRMR                                            0.001       0.001

Parameter Estimates:

  Parameterization                                Delta
  Standard errors                            Robust.sem
  Information                                  Expected
  Information saturated (h1) model         Unstructured

Latent Variables:
                   Estimate  Std.Err  z-value  P(>|z|) ci.lower ci.upper   Std.lv  Std.all
  dep =~
    DEPRESSED_Y2      0.938    0.007  136.628    0.000    0.924    0.951    0.938    0.938
    INTEREST_Y2       0.855    0.010   83.923    0.000    0.835    0.875    0.855    0.855
```

```
  anx =~
    CONTROL_WORRY_    0.826    0.012   70.250    0.000    0.803    0.849    0.826    0.826
    FEEL_ANXIOUS_Y    0.813    0.013   64.511    0.000    0.789    0.838    0.813    0.813

Covariances:
                   Estimate  Std.Err  z-value  P(>|z|) ci.lower ci.upper   Std.lv  Std.all
  dep ~~
    anx               0.917    0.009  101.218    0.000    0.899    0.934    0.917    0.917

Thresholds:
                   Estimate  Std.Err  z-value  P(>|z|) ci.lower ci.upper   Std.lv  Std.all
    DEPRESSED_Y2|1    0.103    0.024    4.208    0.000    0.055    0.151    0.103    0.103
    DEPRESSED_Y2|2    1.107    0.025   43.660    0.000    1.058    1.157    1.107    1.107
    DEPRESSED_Y2|3    1.668    0.036   45.988    0.000    1.597    1.739    1.668    1.668
    INTEREST_Y2|t1    0.252    0.022   11.302    0.000    0.208    0.296    0.252    0.252
    INTEREST_Y2|t2    1.132    0.028   39.940    0.000    1.077    1.188    1.132    1.132
    INTEREST_Y2|t3    1.731    0.036   47.548    0.000    1.660    1.802    1.731    1.731
    CONTROL_WORRY_    0.021    0.023    0.889    0.374   -0.025    0.066    0.021    0.021
    CONTROL_WORRY_    0.944    0.028   34.075    0.000    0.890    0.999    0.944    0.944
    CONTROL_WORRY_    1.529    0.033   46.782    0.000    1.465    1.593    1.529    1.529
    FEEL_ANXIOUS_Y   -0.137    0.022   -6.161    0.000   -0.181   -0.094   -
0.137   -0.137
    FEEL_ANXIOUS_Y    1.010    0.027   37.384    0.000    0.957    1.063    1.010    1.010
    FEEL_ANXIOUS_Y    1.652    0.042   39.307    0.000    1.570    1.735    1.652    1.652

Variances:
                   Estimate  Std.Err  z-value  P(>|z|) ci.lower ci.upper   Std.lv  Std.all
   .DEPRESSED_Y2      0.121                               0.121    0.121    0.121    0.121
   .INTEREST_Y2       0.269                               0.269    0.269    0.269    0.269
   .CONTROL_WORRY_    0.317                               0.317    0.317    0.317    0.317
   .FEEL_ANXIOUS_Y    0.339                               0.339    0.339    0.339    0.339
    dep               1.000                               1.000    1.000    1.000    1.000
    anx               1.000                               1.000    1.000    1.000    1.000
```

```
LIST.CFA.FIT[[cur.country]] <- fit
```

2.6.2.1 Residual correlations

Residual correlation greater than |0.05| are bolded.

```
tb_residual_cor(fit)
```

Variable	DEPRESSED_Y2	INTEREST_Y2	CONTROL_WORRY_Y2	FE
DEPRESSED_Y2	0.00			
INTEREST_Y2	0.00	0.00		
CONTROL_WORRY_Y2	-0.00	0.00	0.00	
FEEL_ANXIOUS_Y2	0.00	-0.00	-0.00	

2.6.2.2 Modification indices

```r
tb_mod_indices(fit, sort.=TRUE, maximum.number = 12)
```

lhs	op	rhs	mi	epc	sepc.lv
DEPRESSED_Y2	~~	FEEL_ANXIOUS_Y2	0.11	0.01	0.01
INTEREST_Y2	~~	FEEL_ANXIOUS_Y2	0.11	-0.01	-0.01
DEPRESSED_Y2	~~	CONTROL_WORRY_Y2	0.11	-0.01	-0.01
INTEREST_Y2	~~	CONTROL_WORRY_Y2	0.11	0.01	0.01

2.7 Hong Kong

2.7.1 Sample statistics

The following sample statistics, computed using the survey design adjusted estimates from the survey package, are used are input for the factor analyses. This facilitates replication of all analyses without the need to get the raw data from the Center for Open Science.

```
ci <- ci + 1
cur.country <- names(sample.stats)[ci]
tb_sample_stats(sample.stats[[cur.country]])
```

Variable	DEPRESSED_Y2	INTEREST_Y2	CONTROL_WORRY_Y2	F
DEPRESSED_Y2	1.00	0.55	0.86	
INTEREST_Y2	0.55	1.00	0.49	
CONTROL_WORRY_Y2	0.86	0.49	1.00	
FEEL_ANXIOUS_Y2	0.84	0.48	0.85	
X				
Mean	0.87	1.17	0.74	
Standard.Deviation	1.00	1.00	1.00	
X.1				
Category	0.00	1.00	2.00	
DEPRESSED_Y2.1	33.59	48.05	15.55	
INTEREST_Y2.1	22.80	44.24	26.28	
CONTROL_WORRY_Y2.1	45.88	36.83	14.85	
FEEL_ANXIOUS_Y2.1	29.76	50.92	15.02	

2.7.2 Confirmatory factor analysis

```
fit <- cfa(
  mod, std.lv = TRUE, ordered=TRUE
  , sample.cov = sample.stats[[cur.country]]$sample.cov
  , sample.mean = sample.stats[[cur.country]]$sample.mean
```

```r
  , sample.nobs = sample.stats[[cur.country]]$sample.nobs
  , sample.th = sample.stats[[cur.country]]$sample.th
  , WLS.V = sample.stats[[cur.country]]$WLS.V
  , NACOV = sample.stats[[cur.country]]$NACOV
)

dynamic::cfaHB(fit)
```

```
Your DFI cutoffs:
            SRMR  RMSEA CFI   Magnitude
Level-0     0.008 0.07  0.998 NONE
Specificity 95%   95%   95%

Level-1     NONE  NONE  NONE  0.431
Sensitivity 0%    4%    0%

Empirical fit indices:
 Chi-Square  df p-value   SRMR   RMSEA    CFI
      0.001   1      NA      0       0      1
```

```r
summary(fit, standardized=TRUE, ci=TRUE, fit.measure=TRUE)
```

```
lavaan 0.6-21 ended normally after 17 iterations

  Estimator                                       DWLS
  Optimization method                           NLMINB
  Number of model parameters                        17

  Number of observations                      707.000248016404

Model Test User Model:
                                              Standard      Scaled
  Test Statistic                                 0.001       0.001
  Degrees of freedom                                 1           1
  P-value (Unknown)                                 NA       0.979
  Scaling correction factor                                  1.683
  Shift parameter                                            0.000
    simple second-order correction

Model Test Baseline Model:
```

```
  Test statistic                             17837.243    2025.433
  Degrees of freedom                                 6           6
  P-value                                           NA       0.000
  Scaling correction factor                                  8.816

User Model versus Baseline Model:

  Comparative Fit Index (CFI)                    1.000       1.000
  Tucker-Lewis Index (TLI)                       1.000       1.003

  Robust Comparative Fit Index (CFI)                         1.000
  Robust Tucker-Lewis Index (TLI)                            1.016

Root Mean Square Error of Approximation:

  RMSEA                                          0.000       0.000
  90 Percent confidence interval - lower         0.000       0.000
  90 Percent confidence interval - upper         0.000       0.000
  P-value H_0: RMSEA <= 0.050                    0.988       0.991
  P-value H_0: RMSEA >= 0.080                    0.003       0.002

  Robust RMSEA                                               0.000
  90 Percent confidence interval - lower                     0.000
  90 Percent confidence interval - upper                     0.000
  P-value H_0: Robust RMSEA <= 0.050                         0.981
  P-value H_0: Robust RMSEA >= 0.080                         0.015

Standardized Root Mean Square Residual:

  SRMR                                           0.000       0.000

Parameter Estimates:

  Parameterization                               Delta
  Standard errors                           Robust.sem
  Information                                 Expected
  Information saturated (h1) model        Unstructured

Latent Variables:
                   Estimate  Std.Err  z-value  P(>|z|) ci.lower ci.upper   Std.lv  Std.all
  dep =~
    DEPRESSED_Y2      0.981    0.024   40.839    0.000    0.934    1.028    0.981    0.981
    INTEREST_Y2       0.562    0.054   10.311    0.000    0.455    0.669    0.562    0.562
```

```
  anx =~
    CONTROL_WORRY_    0.935    0.022   41.726    0.000    0.891    0.979    0.935    0.935
    FEEL_ANXIOUS_Y    0.912    0.017   53.282    0.000    0.878    0.945    0.912    0.912

Covariances:
                   Estimate  Std.Err  z-value  P(>|z|) ci.lower ci.upper   Std.lv  Std.all
  dep ~~
    anx               0.940    0.026   35.644    0.000    0.888    0.992    0.940    0.940

Thresholds:
                   Estimate  Std.Err  z-value  P(>|z|) ci.lower ci.upper   Std.lv  Std.all
    DEPRESSED_Y2|1   -0.424    0.065   -6.487    0.000   -0.552   -0.296   -
0.424   -0.424
    DEPRESSED_Y2|2    0.902    0.074   12.251    0.000    0.758    1.046    0.902    0.902
    DEPRESSED_Y2|3    1.910    0.112   17.018    0.000    1.690    2.130    1.910    1.910
    INTEREST_Y2|t1   -0.745    0.069  -10.788    0.000   -0.881   -0.610   -
0.745   -0.745
    INTEREST_Y2|t2    0.441    0.065    6.836    0.000    0.315    0.568    0.441    0.441
    INTEREST_Y2|t3    1.501    0.122   12.301    0.000    1.261    1.740    1.501    1.501
    CONTROL_WORRY_   -0.103    0.065   -1.595    0.111   -0.230    0.024   -
0.103   -0.103
    CONTROL_WORRY_    0.943    0.075   12.593    0.000    0.796    1.090    0.943    0.943
    CONTROL_WORRY_    1.973    0.128   15.425    0.000    1.722    2.223    1.973    1.973
    FEEL_ANXIOUS_Y   -0.531    0.058   -9.121    0.000   -0.646   -0.417   -
0.531   -0.531
    FEEL_ANXIOUS_Y    0.866    0.061   14.109    0.000    0.746    0.986    0.866    0.866
    FEEL_ANXIOUS_Y    1.717    0.098   17.541    0.000    1.525    1.908    1.717    1.717

Variances:
                   Estimate  Std.Err  z-value  P(>|z|) ci.lower ci.upper   Std.lv  Std.all
   .DEPRESSED_Y2      0.038                               0.038    0.038    0.038    0.038
   .INTEREST_Y2       0.684                               0.684    0.684    0.684    0.684
   .CONTROL_WORRY_    0.125                               0.125    0.125    0.125    0.125
   .FEEL_ANXIOUS_Y    0.169                               0.169    0.169    0.169    0.169
    dep               1.000                               1.000    1.000    1.000    1.000
    anx               1.000                               1.000    1.000    1.000    1.000
```

```
LIST.CFA.FIT[[cur.country]] <- fit
```

2.7.2.1 Residual correlations

Residual correlation greater than |0.05| are bolded.

```r
tb_residual_cor(fit)
```

Variable	DEPRESSED_Y2	INTEREST_Y2	CONTROL_WORRY_Y2	FE
DEPRESSED_Y2	0.00			
INTEREST_Y2	0.00	0.00		
CONTROL_WORRY_Y2	0.00	-0.00	0.00	
FEEL_ANXIOUS_Y2	-0.00	0.00	-0.00	

2.7.2.2 Modification indices

```r
tb_mod_indices(fit, sort.=TRUE, maximum.number = 12)
```

lhs	op	rhs	mi	epc	sepc.
DEPRESSED_Y2	~~	CONTROL_WORRY_Y2	0.00	0.00	0.0
DEPRESSED_Y2	~~	FEEL_ANXIOUS_Y2	0.00	-0.00	-0.0
INTEREST_Y2	~~	FEEL_ANXIOUS_Y2	0.00	0.00	0.0
INTEREST_Y2	~~	CONTROL_WORRY_Y2	0.00	-0.00	-0.0

2.8 India

2.8.1 Sample statistics

The following sample statistics, computed using the survey design adjusted estimates from the survey package, are used are input for the factor analyses. This facilitates replication of all analyses without the need to get the raw data from the Center for Open Science.

```
ci <- ci + 1
cur.country <- names(sample.stats)[ci]
tb_sample_stats(sample.stats[[cur.country]])
```

Variable	DEPRESSED_Y2	INTEREST_Y2	CONTROL_WORRY_Y2	F
DEPRESSED_Y2	1.00	0.37	0.54	
INTEREST_Y2	0.37	1.00	0.32	
CONTROL_WORRY_Y2	0.54	0.32	1.00	
FEEL_ANXIOUS_Y2	0.61	0.29	0.60	
X				
Mean	1.13	1.45	1.12	
Standard.Deviation	1.00	1.00	1.00	
X.1				
Category	0.00	1.00	2.00	
DEPRESSED_Y2.1	38.67	23.79	23.20	
INTEREST_Y2.1	29.75	21.86	21.31	
CONTROL_WORRY_Y2.1	43.28	19.03	19.82	
FEEL_ANXIOUS_Y2.1	41.56	21.37	21.05	

2.8.2 Confirmatory factor analysis

```
fit <- cfa(
  mod, std.lv = TRUE, ordered=TRUE
  , sample.cov = sample.stats[[cur.country]]$sample.cov
  , sample.mean = sample.stats[[cur.country]]$sample.mean
```

```r
  , sample.nobs = sample.stats[[cur.country]]$sample.nobs
  , sample.th = sample.stats[[cur.country]]$sample.th
  , WLS.V = sample.stats[[cur.country]]$WLS.V
  , NACOV = sample.stats[[cur.country]]$NACOV
)

dynamic::cfaHB(fit)
```

```
Your DFI cutoffs:
            SRMR  RMSEA CFI   Magnitude
Level-0     0.008 0.04  0.999 NONE
Specificity 95%   95%   95%

Level-1     NONE  NONE  NONE  0.446
Sensitivity 0%    4%    1%

Empirical fit indices:
 Chi-Square  df p-value   SRMR   RMSEA    CFI
     16.503   1      NA  0.015   0.049  0.999
```

```r
summary(fit, standardized=TRUE, ci=TRUE, fit.measure=TRUE)
```

```
lavaan 0.6-21 ended normally after 14 iterations

  Estimator                                       DWLS
  Optimization method                           NLMINB
  Number of model parameters                        17

  Number of observations                      6374.00000045487

Model Test User Model:
                                              Standard      Scaled
  Test Statistic                                16.503      17.938
  Degrees of freedom                                 1           1
  P-value (Unknown)                                 NA       0.000
  Scaling correction factor                                  0.920
  Shift parameter                                           -0.000
    simple second-order correction

Model Test Baseline Model:
```

```
  Test statistic                              14809.511    5837.654
  Degrees of freedom                                  6           6
  P-value                                            NA       0.000
  Scaling correction factor                                   2.537

User Model versus Baseline Model:

  Comparative Fit Index (CFI)                     0.999       0.997
  Tucker-Lewis Index (TLI)                        0.994       0.983

  Robust Comparative Fit Index (CFI)                          0.993
  Robust Tucker-Lewis Index (TLI)                             0.961

Root Mean Square Error of Approximation:

  RMSEA                                           0.049       0.052
  90 Percent confidence interval - lower          0.030       0.032
  90 Percent confidence interval - upper          0.071       0.074
  P-value H_0: RMSEA <= 0.050                     0.472       0.404
  P-value H_0: RMSEA >= 0.080                     0.010       0.016

  Robust RMSEA                                                0.087
  90 Percent confidence interval - lower                      0.055
  90 Percent confidence interval - upper                      0.125
  P-value H_0: Robust RMSEA <= 0.050                          0.030
  P-value H_0: Robust RMSEA >= 0.080                          0.678

Standardized Root Mean Square Residual:

  SRMR                                            0.015       0.015

Parameter Estimates:

  Parameterization                                Delta
  Standard errors                           Robust.sem
  Information                                  Expected
  Information saturated (h1) model        Unstructured

Latent Variables:
                   Estimate  Std.Err  z-value  P(>|z|) ci.lower ci.upper   Std.lv  Std.all
  dep =~
    DEPRESSED_Y2      0.838    0.025   33.090    0.000    0.788    0.887    0.838    0.838
    INTEREST_Y2       0.446    0.019   23.529    0.000    0.409    0.483    0.446    0.446
```

```
  anx =~
    CONTROL_WORRY_    0.739    0.014   54.239    0.000    0.712    0.765    0.739    0.739
    FEEL_ANXIOUS_Y    0.809    0.012   67.918    0.000    0.786    0.832    0.809    0.809

Covariances:
                   Estimate  Std.Err  z-value  P(>|z|) ci.lower ci.upper   Std.lv  Std.all
  dep ~~
    anx               0.886    0.027   33.095    0.000    0.833    0.938    0.886    0.886

Thresholds:
                   Estimate  Std.Err  z-value  P(>|z|) ci.lower ci.upper   Std.lv  Std.all
    DEPRESSED_Y2|1   -0.288    0.021  -14.035    0.000   -0.328   -0.248   -
0.288   -0.288
    DEPRESSED_Y2|2    0.317    0.019   16.463    0.000    0.280    0.355    0.317    0.317
    DEPRESSED_Y2|3    1.065    0.025   41.940    0.000    1.015    1.115    1.065    1.065
    INTEREST_Y2|t1   -0.532    0.022  -24.245    0.000   -0.575   -0.489   -
0.532   -0.532
    INTEREST_Y2|t2    0.040    0.021    1.887    0.059   -0.002    0.082    0.040    0.040
    INTEREST_Y2|t3    0.610    0.020   30.722    0.000    0.571    0.649    0.610    0.610
    CONTROL_WORRY_   -0.169    0.022   -7.820    0.000   -0.212   -0.127   -
0.169   -0.169
    CONTROL_WORRY_    0.314    0.021   15.262    0.000    0.273    0.354    0.314    0.314
    CONTROL_WORRY_    0.920    0.024   38.222    0.000    0.873    0.967    0.920    0.920
    FEEL_ANXIOUS_Y   -0.213    0.020  -10.701    0.000   -0.252   -0.174   -
0.213   -0.213
    FEEL_ANXIOUS_Y    0.330    0.017   18.981    0.000    0.296    0.364    0.330    0.330
    FEEL_ANXIOUS_Y    0.993    0.028   35.277    0.000    0.938    1.048    0.993    0.993

Variances:
                   Estimate  Std.Err  z-value  P(>|z|) ci.lower ci.upper   Std.lv  Std.all
   .DEPRESSED_Y2      0.298                               0.298    0.298    0.298    0.298
   .INTEREST_Y2       0.801                               0.801    0.801    0.801    0.801
   .CONTROL_WORRY_    0.454                               0.454    0.454    0.454    0.454
   .FEEL_ANXIOUS_Y    0.345                               0.345    0.345    0.345    0.345
    dep               1.000                               1.000    1.000    1.000    1.000
    anx               1.000                               1.000    1.000    1.000    1.000
```

```
LIST.CFA.FIT[[cur.country]] <- fit
```

2.8.2.1 Residual correlations

Residual correlation greater than |0.05| are bolded.

```r
tb_residual_cor(fit)
```

Variable	DEPRESSED_Y2	INTEREST_Y2	CONTROL_WORRY_Y2	FE
DEPRESSED_Y2	0.00			
INTEREST_Y2	0.00	0.00		
CONTROL_WORRY_Y2	-0.01	0.03	0.00	
FEEL_ANXIOUS_Y2	0.01	-0.03	-0.00	

2.8.2.2 Modification indices

```r
tb_mod_indices(fit, sort.=TRUE, maximum.number = 12)
```

lhs	op	rhs	mi	epc	sepc.lv
DEPRESSED_Y2	~~	CONTROL_WORRY_Y2	16.50	-0.13	-0.13
INTEREST_Y2	~~	FEEL_ANXIOUS_Y2	16.50	-0.08	-0.08
INTEREST_Y2	~~	CONTROL_WORRY_Y2	16.50	0.07	0.07
DEPRESSED_Y2	~~	FEEL_ANXIOUS_Y2	16.50	0.15	0.15

2.9 Indonesia

2.9.1 Sample statistics

The following sample statistics, computed using the survey design adjusted estimates from the survey package, are used are input for the factor analyses. This facilitates replication of all analyses without the need to get the raw data from the Center for Open Science.

```
ci <- ci + 1
cur.country <- names(sample.stats)[ci]
tb_sample_stats(sample.stats[[cur.country]])
```

Variable	DEPRESSED_Y2	INTEREST_Y2	CONTROL_WORRY_Y2	F
DEPRESSED_Y2	1.00	0.51	0.69	
INTEREST_Y2	0.51	1.00	0.51	
CONTROL_WORRY_Y2	0.69	0.51	1.00	
FEEL_ANXIOUS_Y2	0.75	0.50	0.75	
X				
Mean	0.63	0.93	0.61	
Standard.Deviation	1.00	1.00	1.00	
X.1				
Category	0.00	1.00	2.00	
DEPRESSED_Y2.1	57.02	30.34	5.91	
INTEREST_Y2.1	37.87	41.83	10.08	
CONTROL_WORRY_Y2.1	60.80	24.86	6.71	
FEEL_ANXIOUS_Y2.1	52.97	30.94	7.02	

2.9.2 Confirmatory factor analysis

```
fit <- cfa(
  mod, std.lv = TRUE, ordered=TRUE
  , sample.cov = sample.stats[[cur.country]]$sample.cov
  , sample.mean = sample.stats[[cur.country]]$sample.mean
```

```r
  , sample.nobs = sample.stats[[cur.country]]$sample.nobs
  , sample.th = sample.stats[[cur.country]]$sample.th
  , WLS.V = sample.stats[[cur.country]]$WLS.V
  , NACOV = sample.stats[[cur.country]]$NACOV
)

dynamic::cfaHB(fit)
```

```
Your DFI cutoffs:
            SRMR  RMSEA CFI   Magnitude
Level-0     0.006 0.04  0.999 NONE
Specificity 95%   95%   95%

Level-1     NONE  NONE  NONE  0.394
Sensitivity 0%    5%    1%

Empirical fit indices:
 Chi-Square  df p-value   SRMR   RMSEA    CFI
      3.453   1      NA   0.01    0.03      1
```

```r
summary(fit, standardized=TRUE, ci=TRUE, fit.measure=TRUE)
```

```
lavaan 0.6-21 ended normally after 13 iterations

  Estimator                                       DWLS
  Optimization method                           NLMINB
  Number of model parameters                        17

  Number of observations                      2684.000001417

Model Test User Model:
                                              Standard      Scaled
  Test Statistic                                 3.453       2.685
  Degrees of freedom                                 1           1
  P-value (Unknown)                                 NA       0.101
  Scaling correction factor                                  1.286
  Shift parameter                                            0.000
    simple second-order correction

Model Test Baseline Model:
```

```
  Test statistic                             13666.105    3842.171
  Degrees of freedom                                 6           6
  P-value                                           NA       0.000
  Scaling correction factor                                  3.558

User Model versus Baseline Model:

  Comparative Fit Index (CFI)                    1.000       1.000
  Tucker-Lewis Index (TLI)                       0.999       0.997

  Robust Comparative Fit Index (CFI)                         0.998
  Robust Tucker-Lewis Index (TLI)                            0.989

Root Mean Square Error of Approximation:

  RMSEA                                          0.030       0.025
  90 Percent confidence interval - lower         0.000       0.000
  90 Percent confidence interval - upper         0.068       0.063
  P-value H_0: RMSEA <= 0.050                    0.768       0.829
  P-value H_0: RMSEA >= 0.080                    0.011       0.006

  Robust RMSEA                                               0.063
  90 Percent confidence interval - lower                     0.000
  90 Percent confidence interval - upper                     0.158
  P-value H_0: Robust RMSEA <= 0.050                         0.278
  P-value H_0: Robust RMSEA >= 0.080                         0.490

Standardized Root Mean Square Residual:

  SRMR                                           0.010       0.010

Parameter Estimates:

  Parameterization                               Delta
  Standard errors                           Robust.sem
  Information                                 Expected
  Information saturated (h1) model        Unstructured

Latent Variables:
                   Estimate  Std.Err  z-value  P(>|z|) ci.lower ci.upper   Std.lv  Std.all
  dep =~
    DEPRESSED_Y2      0.861    0.025   33.783    0.000    0.811    0.910    0.861    0.861
    INTEREST_Y2       0.593    0.027   21.579    0.000    0.539    0.647    0.593    0.593
```

```
  anx =~
    CONTROL_WORRY_    0.841    0.015   56.732    0.000    0.812    0.870    0.841    0.841
    FEEL_ANXIOUS_Y    0.891    0.015   60.748    0.000    0.862    0.919    0.891    0.891

Covariances:
                   Estimate  Std.Err  z-value  P(>|z|) ci.lower ci.upper   Std.lv  Std.all
  dep ~~
    anx               0.970    0.031   31.235    0.000    0.909    1.031    0.970    0.970

Thresholds:
                   Estimate  Std.Err  z-value  P(>|z|) ci.lower ci.upper   Std.lv  Std.all
    DEPRESSED_Y2|1    0.177    0.038    4.598    0.000    0.101    0.252    0.177    0.177
    DEPRESSED_Y2|2    1.144    0.040   28.273    0.000    1.064    1.223    1.144    1.144
    DEPRESSED_Y2|3    1.496    0.049   30.513    0.000    1.400    1.593    1.496    1.496
    INTEREST_Y2|t1   -0.309    0.040   -7.764    0.000   -0.387   -0.231   -
0.309   -0.309
    INTEREST_Y2|t2    0.831    0.048   17.256    0.000    0.737    0.925    0.831    0.831
    INTEREST_Y2|t3    1.269    0.051   24.982    0.000    1.170    1.369    1.269    1.269
    CONTROL_WORRY_    0.274    0.038    7.296    0.000    0.201    0.348    0.274    0.274
    CONTROL_WORRY_    1.065    0.041   26.187    0.000    0.986    1.145    1.065    1.065
    CONTROL_WORRY_    1.431    0.057   24.900    0.000    1.318    1.544    1.431    1.431
    FEEL_ANXIOUS_Y    0.075    0.032    2.338    0.019    0.012    0.137    0.075    0.075
    FEEL_ANXIOUS_Y    0.991    0.044   22.693    0.000    0.905    1.077    0.991    0.991
    FEEL_ANXIOUS_Y    1.337    0.048   27.666    0.000    1.242    1.431    1.337    1.337

Variances:
                   Estimate  Std.Err  z-value  P(>|z|) ci.lower ci.upper   Std.lv  Std.all
   .DEPRESSED_Y2      0.259                               0.259    0.259    0.259    0.259
   .INTEREST_Y2       0.649                               0.649    0.649    0.649    0.649
   .CONTROL_WORRY_    0.293                               0.293    0.293    0.293    0.293
   .FEEL_ANXIOUS_Y    0.207                               0.207    0.207    0.207    0.207
    dep               1.000                               1.000    1.000    1.000    1.000
    anx               1.000                               1.000    1.000    1.000    1.000
```

```r
LIST.CFA.FIT[[cur.country]] <- fit
```

2.9.2.1 Residual correlations

Residual correlation greater than |0.05| are bolded.

```r
tb_residual_cor(fit)
```

Variable	DEPRESSED_Y2	INTEREST_Y2	CONTROL_WORRY_Y2	FE
DEPRESSED_Y2	0.00			
INTEREST_Y2	-0.00	0.00		
CONTROL_WORRY_Y2	-0.01	0.02	0.00	
FEEL_ANXIOUS_Y2	0.01	-0.01	-0.00	

2.9.2.2 Modification indices

```
tb_mod_indices(fit, sort.=TRUE, maximum.number = 12)
```

lhs	op	rhs	mi	epc	sepc.lv
DEPRESSED_Y2	~~	FEEL_ANXIOUS_Y2	3.45	0.07	0.07
DEPRESSED_Y2	~~	CONTROL_WORRY_Y2	3.45	-0.07	-0.07
INTEREST_Y2	~~	CONTROL_WORRY_Y2	3.45	0.05	0.05
INTEREST_Y2	~~	FEEL_ANXIOUS_Y2	3.45	-0.05	-0.05

2.10 Israel

2.10.1 Sample statistics

The following sample statistics, computed using the survey design adjusted estimates from the survey package, are used are input for the factor analyses. This facilitates replication of all analyses without the need to get the raw data from the Center for Open Science.

```
ci <- ci + 1
cur.country <- names(sample.stats)[ci]
tb_sample_stats(sample.stats[[cur.country]])
```

Variable	DEPRESSED_Y2	INTEREST_Y2	CONTROL_WORRY_Y2	F
DEPRESSED_Y2	1.00	0.75	0.77	
INTEREST_Y2	0.75	1.00	0.58	
CONTROL_WORRY_Y2	0.77	0.58	1.00	
FEEL_ANXIOUS_Y2	0.85	0.72	0.82	
X				
Mean	0.64	0.67	0.58	
Standard.Deviation	1.00	1.00	1.00	
X.1				
Category	0.00	1.00	2.00	
DEPRESSED_Y2.1	55.11	29.78	10.07	
INTEREST_Y2.1	53.40	29.59	12.20	
CONTROL_WORRY_Y2.1	59.41	26.69	10.19	
FEEL_ANXIOUS_Y2.1	63.40	25.07	7.92	

2.10.2 Confirmatory factor analysis

```
fit <- cfa(
  mod, std.lv = TRUE, ordered=TRUE
  , sample.cov = sample.stats[[cur.country]]$sample.cov
  , sample.mean = sample.stats[[cur.country]]$sample.mean
```

```r
  , sample.nobs = sample.stats[[cur.country]]$sample.nobs
  , sample.th = sample.stats[[cur.country]]$sample.th
  , WLS.V = sample.stats[[cur.country]]$WLS.V
  , NACOV = sample.stats[[cur.country]]$NACOV
)

dynamic::cfaHB(fit)
```

```
Your DFI cutoffs:
            SRMR  RMSEA CFI  Magnitude
Level-0     0.004 0.041 1    NONE
Specificity 95%   95%   95%

Level-1     NONE  NONE  NONE 0.218
Sensitivity 0%    4%    1%

Empirical fit indices:
 Chi-Square  df p-value   SRMR   RMSEA    CFI
     13.561   1      NA  0.013   0.071      1
```

```r
summary(fit, standardized=TRUE, ci=TRUE, fit.measure=TRUE)
```

```
lavaan 0.6-21 ended normally after 16 iterations

  Estimator                                       DWLS
  Optimization method                           NLMINB
  Number of model parameters                        17

  Number of observations                      2490.00002081761

Model Test User Model:
                                              Standard      Scaled
  Test Statistic                                13.561       9.240
  Degrees of freedom                                 1           1
  P-value (Unknown)                                 NA       0.002
  Scaling correction factor                                  1.468
  Shift parameter                                            0.000
    simple second-order correction

Model Test Baseline Model:
```

```
  Test statistic                             39077.437    2552.711
  Degrees of freedom                                 6           6
  P-value                                           NA       0.000
  Scaling correction factor                                 15.325

User Model versus Baseline Model:

  Comparative Fit Index (CFI)                    1.000       0.997
  Tucker-Lewis Index (TLI)                       0.998       0.981

  Robust Comparative Fit Index (CFI)                         0.990
  Robust Tucker-Lewis Index (TLI)                            0.941

Root Mean Square Error of Approximation:

  RMSEA                                          0.071       0.058
  90 Percent confidence interval - lower         0.041       0.028
  90 Percent confidence interval - upper         0.107       0.094
  P-value H_0: RMSEA <= 0.050                    0.117       0.293
  P-value H_0: RMSEA >= 0.080                    0.379       0.171

  Robust RMSEA                                               0.182
  90 Percent confidence interval - lower                     0.088
  90 Percent confidence interval - upper                     0.298
  P-value H_0: Robust RMSEA <= 0.050                         0.013
  P-value H_0: Robust RMSEA >= 0.080                         0.962

Standardized Root Mean Square Residual:

  SRMR                                           0.013       0.013

Parameter Estimates:

  Parameterization                               Delta
  Standard errors                          Robust.sem
  Information                                 Expected
  Information saturated (h1) model        Unstructured

Latent Variables:
                   Estimate  Std.Err  z-value  P(>|z|) ci.lower ci.upper   Std.lv  Std.all
  dep =~
    DEPRESSED_Y2      0.959    0.015   65.060    0.000    0.930    0.988    0.959    0.959
    INTEREST_Y2       0.784    0.034   23.297    0.000    0.718    0.850    0.784    0.784
```

```
  anx =~
    CONTROL_WORRY_    0.848    0.018   46.209    0.000    0.812    0.884    0.848    0.848
    FEEL_ANXIOUS_Y    0.970    0.013   76.835    0.000    0.945    0.994    0.970    0.970

Covariances:
                   Estimate  Std.Err  z-value  P(>|z|) ci.lower ci.upper   Std.lv  Std.all
  dep ~~
    anx               0.928    0.020   47.206    0.000    0.889    0.966    0.928    0.928

Thresholds:
                   Estimate  Std.Err  z-value  P(>|z|) ci.lower ci.upper   Std.lv  Std.all
    DEPRESSED_Y2|1    0.128    0.073    1.769    0.077   -0.014    0.271    0.128    0.128
    DEPRESSED_Y2|2    1.032    0.070   14.796    0.000    0.895    1.168    1.032    1.032
    DEPRESSED_Y2|3    1.641    0.101   16.194    0.000    1.442    1.839    1.641    1.641
    INTEREST_Y2|t1    0.085    0.078    1.088    0.277   -0.068    0.239    0.085    0.085
    INTEREST_Y2|t2    0.954    0.070   13.629    0.000    0.817    1.091    0.954    0.954
    INTEREST_Y2|t3    1.664    0.100   16.681    0.000    1.469    1.860    1.664    1.664
    CONTROL_WORRY_    0.238    0.061    3.917    0.000    0.119    0.357    0.238    0.238
    CONTROL_WORRY_    1.085    0.063   17.344    0.000    0.962    1.207    1.085    1.085
    CONTROL_WORRY_    1.785    0.113   15.867    0.000    1.565    2.006    1.785    1.785
    FEEL_ANXIOUS_Y    0.343    0.073    4.705    0.000    0.200    0.485    0.343    0.343
    FEEL_ANXIOUS_Y    1.199    0.068   17.632    0.000    1.066    1.332    1.199    1.199
    FEEL_ANXIOUS_Y    1.798    0.087   20.648    0.000    1.627    1.968    1.798    1.798

Variances:
                   Estimate  Std.Err  z-value  P(>|z|) ci.lower ci.upper   Std.lv  Std.all
   .DEPRESSED_Y2      0.081                               0.081    0.081    0.081    0.081
   .INTEREST_Y2       0.386                               0.386    0.386    0.386    0.386
   .CONTROL_WORRY_    0.281                               0.281    0.281    0.281    0.281
   .FEEL_ANXIOUS_Y    0.060                               0.060    0.060    0.060    0.060
    dep               1.000                               1.000    1.000    1.000    1.000
    anx               1.000                               1.000    1.000    1.000    1.000
```

```r
LIST.CFA.FIT[[cur.country]] <- fit
```

2.10.2.1 Residual correlations

Residual correlation greater than |0.05| are bolded.

```r
tb_residual_cor(fit)
```

Variable	DEPRESSED_Y2	INTEREST_Y2	CONTROL_WORRY_Y2	FE
DEPRESSED_Y2	0.00			
INTEREST_Y2	-0.00	0.00		
CONTROL_WORRY_Y2	0.02	-0.03	0.00	
FEEL_ANXIOUS_Y2	-0.01	0.01	0.00	

2.10.2.2 Modification indices

```
tb_mod_indices(fit, sort.=TRUE, maximum.number = 12)
```

lhs	op	rhs	mi	epc	sepc.lv
DEPRESSED_Y2	~~	FEEL_ANXIOUS_Y2	13.56	-0.09	-0.09
INTEREST_Y2	~~	FEEL_ANXIOUS_Y2	13.56	0.08	0.08
INTEREST_Y2	~~	CONTROL_WORRY_Y2	13.56	-0.07	-0.07
DEPRESSED_Y2	~~	CONTROL_WORRY_Y2	13.56	0.08	0.08

2.11 Japan

2.11.1 Sample statistics

The following sample statistics, computed using the survey design adjusted estimates from the survey package, are used are input for the factor analyses. This facilitates replication of all analyses without the need to get the raw data from the Center for Open Science.

```
ci <- ci + 1
cur.country <- names(sample.stats)[ci]
tb_sample_stats(sample.stats[[cur.country]])
```

Variable	DEPRESSED_Y2	INTEREST_Y2	CONTROL_WORRY_Y2	F
DEPRESSED_Y2	1.00	0.85	0.84	
INTEREST_Y2	0.85	1.00	0.74	
CONTROL_WORRY_Y2	0.84	0.74	1.00	
FEEL_ANXIOUS_Y2	0.88	0.77	0.86	
X				
Mean	0.69	0.79	0.54	
Standard.Deviation	1.00	1.00	1.00	
X.1				
Category	0.00	1.00	2.00	
DEPRESSED_Y2.1	53.94	28.69	11.54	
INTEREST_Y2.1	49.49	28.67	15.64	
CONTROL_WORRY_Y2.1	63.73	23.08	9.00	
FEEL_ANXIOUS_Y2.1	43.72	35.98	13.26	

2.11.2 Confirmatory factor analysis

```
fit <- cfa(
  mod, std.lv = TRUE, ordered=TRUE
  , sample.cov = sample.stats[[cur.country]]$sample.cov
  , sample.mean = sample.stats[[cur.country]]$sample.mean
```

```
  , sample.nobs = sample.stats[[cur.country]]$sample.nobs
  , sample.th = sample.stats[[cur.country]]$sample.th
  , WLS.V = sample.stats[[cur.country]]$WLS.V
  , NACOV = sample.stats[[cur.country]]$NACOV
)

dynamic::cfaHB(fit)
```

```
Your DFI cutoffs:
            SRMR  RMSEA CFI  Magnitude
Level-0     0.003 0.04  1    NONE
Specificity 95%   95%   95%

Level-1     NONE  NONE  NONE 0.139
Sensitivity 0%    5%    1%

Empirical fit indices:
 Chi-Square  df p-value   SRMR   RMSEA    CFI
          0   1      NA      0       0      1
```

```
summary(fit, standardized=TRUE, ci=TRUE, fit.measure=TRUE)
```

```
lavaan 0.6-21 ended normally after 19 iterations

  Estimator                                         DWLS
  Optimization method                             NLMINB
  Number of model parameters                          17

  Number of observations                        13972.0000004316

Model Test User Model:
                                              Standard      Scaled
  Test Statistic                                 0.000       0.000
  Degrees of freedom                                 1           1
  P-value (Unknown)                                 NA       0.987
  Scaling correction factor                                  1.071
  Shift parameter                                           -0.000
    simple second-order correction

Model Test Baseline Model:
```

```
  Test statistic                              565441.004   66414.983
  Degrees of freedom                                   6           6
  P-value                                             NA       0.000
  Scaling correction factor                                    8.514

User Model versus Baseline Model:

  Comparative Fit Index (CFI)                      1.000       1.000
  Tucker-Lewis Index (TLI)                         1.000       1.000

  Robust Comparative Fit Index (CFI)                           1.000
  Robust Tucker-Lewis Index (TLI)                              1.001

Root Mean Square Error of Approximation:

  RMSEA                                            0.000       0.000
  90 Percent confidence interval - lower           0.000       0.000
  90 Percent confidence interval - upper           0.000       0.000
  P-value H_0: RMSEA <= 0.050                      1.000       1.000
  P-value H_0: RMSEA >= 0.080                      0.000       0.000

  Robust RMSEA                                                 0.000
  90 Percent confidence interval - lower                       0.000
  90 Percent confidence interval - upper                       0.000
  P-value H_0: Robust RMSEA <= 0.050                           0.999
  P-value H_0: Robust RMSEA >= 0.080                           0.000

Standardized Root Mean Square Residual:

  SRMR                                             0.000       0.000

Parameter Estimates:

  Parameterization                                 Delta
  Standard errors                             Robust.sem
  Information                                   Expected
  Information saturated (h1) model          Unstructured

Latent Variables:
                   Estimate  Std.Err  z-value  P(>|z|) ci.lower ci.upper   Std.lv  Std.all
  dep =~
    DEPRESSED_Y2      0.983    0.003  365.092    0.000    0.978    0.988    0.983    0.983
    INTEREST_Y2       0.861    0.005  169.155    0.000    0.851    0.871    0.861    0.861
```

```
  anx =~
    CONTROL_WORRY_    0.908    0.004  227.109    0.000    0.900    0.916    0.908    0.908
    FEEL_ANXIOUS_Y    0.942    0.004  263.939    0.000    0.935    0.949    0.942    0.942

Covariances:
                   Estimate  Std.Err  z-value  P(>|z|) ci.lower ci.upper   Std.lv  Std.all
  dep ~~
    anx               0.946    0.006  165.281    0.000    0.935    0.957    0.946    0.946

Thresholds:
                   Estimate  Std.Err  z-value  P(>|z|) ci.lower ci.upper   Std.lv  Std.all
    DEPRESSED_Y2|1    0.099    0.014    7.126    0.000    0.072    0.126    0.099    0.099
    DEPRESSED_Y2|2    0.940    0.016   60.234    0.000    0.909    0.970    0.940    0.940
    DEPRESSED_Y2|3    1.569    0.020   79.236    0.000    1.530    1.608    1.569    1.569
    INTEREST_Y2|t1   -0.013    0.014   -0.905    0.366   -0.040    0.015   -
0.013   -0.013
    INTEREST_Y2|t2    0.778    0.015   51.856    0.000    0.748    0.807    0.778    0.778
    INTEREST_Y2|t3    1.538    0.019   82.844    0.000    1.502    1.574    1.538    1.538
    CONTROL_WORRY_    0.351    0.013   26.245    0.000    0.325    0.377    0.351    0.351
    CONTROL_WORRY_    1.118    0.013   83.823    0.000    1.091    1.144    1.118    1.118
    CONTROL_WORRY_    1.729    0.024   71.175    0.000    1.682    1.777    1.729    1.729
    FEEL_ANXIOUS_Y   -0.158    0.012  -12.976    0.000   -0.182   -0.134   -
0.158   -0.158
    FEEL_ANXIOUS_Y    0.831    0.013   62.612    0.000    0.805    0.857    0.831    0.831
    FEEL_ANXIOUS_Y    1.473    0.020   72.439    0.000    1.433    1.513    1.473    1.473

Variances:
                   Estimate  Std.Err  z-value  P(>|z|) ci.lower ci.upper   Std.lv  Std.all
   .DEPRESSED_Y2      0.034                               0.034    0.034    0.034    0.034
   .INTEREST_Y2       0.259                               0.259    0.259    0.259    0.259
   .CONTROL_WORRY_    0.176                               0.176    0.176    0.176    0.176
   .FEEL_ANXIOUS_Y    0.113                               0.113    0.113    0.113    0.113
    dep               1.000                               1.000    1.000    1.000    1.000
    anx               1.000                               1.000    1.000    1.000    1.000
```

```
LIST.CFA.FIT[[cur.country]] <- fit
```

2.11.2.1 Residual correlations

Residual correlation greater than |0.05| are bolded.

```r
tb_residual_cor(fit)
```

Variable	DEPRESSED_Y2	INTEREST_Y2	CONTROL_WORRY_Y2	FE[illegible]
DEPRESSED_Y2	0.00			
INTEREST_Y2	-0.00	0.00		
CONTROL_WORRY_Y2	-0.00	0.00	0.00	
FEEL_ANXIOUS_Y2	0.00	-0.00	0.00	[illegible]

2.11.2.2 Modification indices

```r
tb_mod_indices(fit, sort.=TRUE, maximum.number = 12)
```

lhs	op	rhs	mi	epc
INTEREST_Y2	~~	CONTROL_WORRY_Y2	0.00	0.00
DEPRESSED_Y2	~~	CONTROL_WORRY_Y2	0.00	-0.00
INTEREST_Y2	~~	FEEL_ANXIOUS_Y2	0.00	-0.00
DEPRESSED_Y2	~~	FEEL_ANXIOUS_Y2	0.00	0.00

2.12 Kenya

2.12.1 Sample statistics

The following sample statistics, computed using the survey design adjusted estimates from the survey package, are used are input for the factor analyses. This facilitates replication of all analyses without the need to get the raw data from the Center for Open Science.

```
ci <- ci + 1
cur.country <- names(sample.stats)[ci]
tb_sample_stats(sample.stats[[cur.country]])
```

Variable	DEPRESSED_Y2	INTEREST_Y2	CONTROL_WORRY_Y2	F
DEPRESSED_Y2	1.00	0.47	0.51	
INTEREST_Y2	0.47	1.00	0.43	
CONTROL_WORRY_Y2	0.51	0.43	1.00	
FEEL_ANXIOUS_Y2	0.56	0.46	0.57	
X				
Mean	1.10	1.20	1.10	
Standard.Deviation	1.00	1.00	1.00	
X.1				
Category	0.00	1.00	2.00	
DEPRESSED_Y2.1	32.15	40.90	11.97	
INTEREST_Y2.1	26.36	44.14	12.33	
CONTROL_WORRY_Y2.1	35.34	35.54	12.43	
FEEL_ANXIOUS_Y2.1	36.65	37.20	12.70	

2.12.2 Confirmatory factor analysis

```
fit <- cfa(
  mod, std.lv = TRUE, ordered=TRUE
  , sample.cov = sample.stats[[cur.country]]$sample.cov
  , sample.mean = sample.stats[[cur.country]]$sample.mean
```

```
  , sample.nobs = sample.stats[[cur.country]]$sample.nobs
  , sample.th = sample.stats[[cur.country]]$sample.th
  , WLS.V = sample.stats[[cur.country]]$WLS.V
  , NACOV = sample.stats[[cur.country]]$NACOV
)

dynamic::cfaHB(fit)
```

```
Your DFI cutoffs:
            SRMR  RMSEA CFI   Magnitude
Level-0     0.007 0.039 0.999 NONE
Specificity 95%   95%   95%

Level-1     NONE  NONE  NONE  0.367
Sensitivity 2%    5%    1%

Empirical fit indices:
 Chi-Square  df p-value   SRMR   RMSEA    CFI
      0.223   1      NA  0.002       0      1
```

```
summary(fit, standardized=TRUE, ci=TRUE, fit.measure=TRUE)
```

```
lavaan 0.6-21 ended normally after 14 iterations

  Estimator                                       DWLS
  Optimization method                           NLMINB
  Number of model parameters                        17

  Number of observations                      7698.00001112736

Model Test User Model:
                                              Standard      Scaled
  Test Statistic                                 0.223       0.222
  Degrees of freedom                                 1           1
  P-value (Unknown)                                 NA       0.637
  Scaling correction factor                                  1.005
  Shift parameter                                           -0.000
    simple second-order correction

Model Test Baseline Model:
```

```
  Test statistic                               14905.655    5119.430
  Degrees of freedom                                   6           6
  P-value                                             NA       0.000
  Scaling correction factor                                    2.912

User Model versus Baseline Model:

  Comparative Fit Index (CFI)                      1.000       1.000
  Tucker-Lewis Index (TLI)                         1.000       1.001

  Robust Comparative Fit Index (CFI)                           1.000
  Robust Tucker-Lewis Index (TLI)                              1.002

Root Mean Square Error of Approximation:

  RMSEA                                            0.000       0.000
  90 Percent confidence interval - lower           0.000       0.000
  90 Percent confidence interval - upper           0.024       0.024
  P-value H_0: RMSEA <= 0.050                      1.000       1.000
  P-value H_0: RMSEA >= 0.080                      0.000       0.000

  Robust RMSEA                                                 0.000
  90 Percent confidence interval - lower                       0.000
  90 Percent confidence interval - upper                       0.045
  P-value H_0: Robust RMSEA <= 0.050                           0.967
  P-value H_0: Robust RMSEA >= 0.080                           0.001

Standardized Root Mean Square Residual:

  SRMR                                             0.002       0.002

Parameter Estimates:

  Parameterization                                 Delta
  Standard errors                             Robust.sem
  Information                                   Expected
  Information saturated (h1) model            Unstructured

Latent Variables:
                   Estimate  Std.Err  z-value  P(>|z|) ci.lower ci.upper   Std.lv  Std.all
  dep =~
    DEPRESSED_Y2      0.756    0.015   51.388    0.000    0.727    0.785    0.756    0.756
    INTEREST_Y2       0.627    0.017   36.977    0.000    0.594    0.660    0.627    0.627
```

```
  anx =~
    CONTROL_WORRY_    0.724    0.014   53.017    0.000    0.697    0.750    0.724    0.724
    FEEL_ANXIOUS_Y    0.785    0.014   54.767    0.000    0.757    0.813    0.785    0.785

Covariances:
                   Estimate  Std.Err  z-value  P(>|z|) ci.lower ci.upper   Std.lv  Std.all
  dep ~~
    anx               0.944    0.020   47.459    0.000    0.905    0.983    0.944    0.944

Thresholds:
                   Estimate  Std.Err  z-value  P(>|z|) ci.lower ci.upper   Std.lv  Std.all
    DEPRESSED_Y2|1   -0.464    0.017  -26.627    0.000   -0.498   -0.429   -
0.464   -0.464
    DEPRESSED_Y2|2    0.614    0.021   29.543    0.000    0.573    0.655    0.614    0.614
    DEPRESSED_Y2|3    1.037    0.018   56.773    0.000    1.001    1.073    1.037    1.037
    INTEREST_Y2|t1   -0.632    0.026  -24.102    0.000   -0.684   -0.581   -
0.632   -0.632
    INTEREST_Y2|t2    0.539    0.025   21.918    0.000    0.490    0.587    0.539    0.539
    INTEREST_Y2|t3    0.947    0.028   34.421    0.000    0.893    1.001    0.947    0.947
    CONTROL_WORRY_   -0.376    0.019  -19.689    0.000   -0.414   -0.339   -
0.376   -0.376
    CONTROL_WORRY_    0.550    0.018   31.312    0.000    0.516    0.584    0.550    0.550
    CONTROL_WORRY_    0.967    0.026   37.345    0.000    0.916    1.017    0.967    0.967
    FEEL_ANXIOUS_Y   -0.341    0.022  -15.423    0.000   -0.384   -0.298   -
0.341   -0.341
    FEEL_ANXIOUS_Y    0.639    0.023   27.751    0.000    0.594    0.684    0.639    0.639
    FEEL_ANXIOUS_Y    1.106    0.024   46.080    0.000    1.059    1.153    1.106    1.106

Variances:
                   Estimate  Std.Err  z-value  P(>|z|) ci.lower ci.upper   Std.lv  Std.all
   .DEPRESSED_Y2      0.428                               0.428    0.428    0.428    0.428
   .INTEREST_Y2       0.607                               0.607    0.607    0.607    0.607
   .CONTROL_WORRY_    0.477                               0.477    0.477    0.477    0.477
   .FEEL_ANXIOUS_Y    0.384                               0.384    0.384    0.384    0.384
    dep               1.000                               1.000    1.000    1.000    1.000
    anx               1.000                               1.000    1.000    1.000    1.000
```

```r
LIST.CFA.FIT[[cur.country]] <- fit
```

2.12.2.1 Residual correlations

Residual correlation greater than |0.05| are bolded.

```r
tb_residual_cor(fit)
```

Variable	DEPRESSED_Y2	INTEREST_Y2	CONTROL_WORRY_Y2	FE
DEPRESSED_Y2	0.00			
INTEREST_Y2	-0.00	0.00		
CONTROL_WORRY_Y2	-0.00	0.00	0.00	
FEEL_ANXIOUS_Y2	0.00	-0.00	0.00	

2.12.2.2 Modification indices

```r
tb_mod_indices(fit, sort.=TRUE, maximum.number = 12)
```

lhs	op	rhs	mi	epc	sepc.lv
DEPRESSED_Y2	~~	CONTROL_WORRY_Y2	0.22	-0.01	-0.01
INTEREST_Y2	~~	FEEL_ANXIOUS_Y2	0.22	-0.01	-0.01
INTEREST_Y2	~~	CONTROL_WORRY_Y2	0.22	0.01	0.01
DEPRESSED_Y2	~~	FEEL_ANXIOUS_Y2	0.22	0.01	0.01

2.13 Mexico

2.13.1 Sample statistics

The following sample statistics, computed using the survey design adjusted estimates from the survey package, are used are input for the factor analyses. This facilitates replication of all analyses without the need to get the raw data from the Center for Open Science.

```
ci <- ci + 1
cur.country <- names(sample.stats)[ci]
tb_sample_stats(sample.stats[[cur.country]])
```

Variable	DEPRESSED_Y2	INTEREST_Y2	CONTROL_WORRY_Y2	F
DEPRESSED_Y2	1.00	0.76	0.69	
INTEREST_Y2	0.76	1.00	0.61	
CONTROL_WORRY_Y2	0.69	0.61	1.00	
FEEL_ANXIOUS_Y2	0.77	0.64	0.75	
X				
Mean	0.88	0.98	0.96	
Standard.Deviation	1.00	1.00	1.00	
X.1				
Category	0.00	1.00	2.00	
DEPRESSED_Y2.1	44.97	32.25	12.29	
INTEREST_Y2.1	39.35	34.74	14.82	
CONTROL_WORRY_Y2.1	43.03	31.00	13.38	
FEEL_ANXIOUS_Y2.1	43.26	33.90	13.44	

2.13.2 Confirmatory factor analysis

```
fit <- cfa(
  mod, std.lv = TRUE, ordered=TRUE
  , sample.cov = sample.stats[[cur.country]]$sample.cov
  , sample.mean = sample.stats[[cur.country]]$sample.mean
```

```r
  , sample.nobs = sample.stats[[cur.country]]$sample.nobs
  , sample.th = sample.stats[[cur.country]]$sample.th
  , WLS.V = sample.stats[[cur.country]]$WLS.V
  , NACOV = sample.stats[[cur.country]]$NACOV
)

dynamic::cfaHB(fit)
```

```
Your DFI cutoffs:
            SRMR  RMSEA CFI   Magnitude
Level-0     0.004 0.041 0.999 NONE
Specificity 95%   95%   95%

Level-1     NONE  NONE  NONE  0.201
Sensitivity 0%    4%    1%

Empirical fit indices:
 Chi-Square  df p-value   SRMR   RMSEA    CFI
      2.137   1      NA  0.006   0.022      1
```

```r
summary(fit, standardized=TRUE, ci=TRUE, fit.measure=TRUE)
```

```
lavaan 0.6-21 ended normally after 15 iterations

  Estimator                                       DWLS
  Optimization method                           NLMINB
  Number of model parameters                        17

  Number of observations                      2278.00000004553

Model Test User Model:
                                              Standard      Scaled
  Test Statistic                                 2.137       2.206
  Degrees of freedom                                 1           1
  P-value (Unknown)                                 NA       0.137
  Scaling correction factor                                  0.969
  Shift parameter                                           -0.000
    simple second-order correction

Model Test Baseline Model:
```

```
  Test statistic                             20700.330    3968.938
  Degrees of freedom                                 6           6
  P-value                                           NA       0.000
  Scaling correction factor                                  5.218

User Model versus Baseline Model:

  Comparative Fit Index (CFI)                    1.000       1.000
  Tucker-Lewis Index (TLI)                       1.000       0.998

  Robust Comparative Fit Index (CFI)                         0.999
  Robust Tucker-Lewis Index (TLI)                            0.992

Root Mean Square Error of Approximation:

  RMSEA                                          0.022       0.023
  90 Percent confidence interval - lower         0.000       0.000
  90 Percent confidence interval - upper         0.065       0.066
  P-value H_0: RMSEA <= 0.050                    0.822       0.816
  P-value H_0: RMSEA >= 0.080                    0.009       0.010

  Robust RMSEA                                               0.059
  90 Percent confidence interval - lower                     0.000
  90 Percent confidence interval - upper                     0.167
  P-value H_0: Robust RMSEA <= 0.050                         0.297
  P-value H_0: Robust RMSEA >= 0.080                         0.495

Standardized Root Mean Square Residual:

  SRMR                                           0.006       0.006

Parameter Estimates:

  Parameterization                               Delta
  Standard errors                           Robust.sem
  Information                                 Expected
  Information saturated (h1) model        Unstructured

Latent Variables:
                   Estimate  Std.Err  z-value  P(>|z|) ci.lower ci.upper   Std.lv  Std.all
  dep =~
    DEPRESSED_Y2      0.940    0.012   76.432    0.000    0.916    0.964    0.940    0.940
    INTEREST_Y2       0.806    0.019   41.433    0.000    0.768    0.845    0.806    0.806
```

```
  anx =~
    CONTROL_WORRY_    0.831    0.016   52.587    0.000    0.800    0.862    0.831    0.831
    FEEL_ANXIOUS_Y    0.902    0.011   84.640    0.000    0.881    0.923    0.902    0.902

Covariances:
                   Estimate  Std.Err  z-value  P(>|z|) ci.lower ci.upper   Std.lv  Std.all
  dep ~~
    anx               0.896    0.024   37.978    0.000    0.850    0.942    0.896    0.896

Thresholds:
                   Estimate  Std.Err  z-value  P(>|z|) ci.lower ci.upper   Std.lv  Std.all
    DEPRESSED_Y2|1   -0.126    0.040   -3.160    0.002   -0.205   -0.048   -
0.126   -0.126
    DEPRESSED_Y2|2    0.746    0.047   15.955    0.000    0.654    0.838    0.746    0.746
    DEPRESSED_Y2|3    1.254    0.049   25.629    0.000    1.158    1.350    1.254    1.254
    INTEREST_Y2|t1   -0.270    0.040   -6.671    0.000   -0.349   -0.191   -
0.270   -0.270
    INTEREST_Y2|t2    0.646    0.041   15.935    0.000    0.567    0.726    0.646    0.646
    INTEREST_Y2|t3    1.222    0.046   26.833    0.000    1.133    1.311    1.222    1.222
    CONTROL_WORRY_   -0.176    0.038   -4.595    0.000   -0.251   -0.101   -
0.176   -0.176
    CONTROL_WORRY_    0.644    0.043   14.932    0.000    0.560    0.729    0.644    0.644
    CONTROL_WORRY_    1.146    0.041   27.697    0.000    1.065    1.227    1.146    1.146
    FEEL_ANXIOUS_Y   -0.170    0.039   -4.401    0.000   -0.245   -0.094   -
0.170   -0.170
    FEEL_ANXIOUS_Y    0.744    0.043   17.310    0.000    0.660    0.829    0.744    0.744
    FEEL_ANXIOUS_Y    1.317    0.044   29.645    0.000    1.230    1.404    1.317    1.317

Variances:
                   Estimate  Std.Err  z-value  P(>|z|) ci.lower ci.upper   Std.lv  Std.all
   .DEPRESSED_Y2      0.117                               0.117    0.117    0.117    0.117
   .INTEREST_Y2       0.350                               0.350    0.350    0.350    0.350
   .CONTROL_WORRY_    0.310                               0.310    0.310    0.310    0.310
   .FEEL_ANXIOUS_Y    0.186                               0.186    0.186    0.186    0.186
    dep               1.000                               1.000    1.000    1.000    1.000
    anx               1.000                               1.000    1.000    1.000    1.000
```

```
LIST.CFA.FIT[[cur.country]] <- fit
```

2.13.2.1 Residual correlations

Residual correlation greater than |0.05| are bolded.

```r
tb_residual_cor(fit)
```

Variable	DEPRESSED_Y2	INTEREST_Y2	CONTROL_WORRY_Y2	FE
DEPRESSED_Y2	0.00			
INTEREST_Y2	0.00	0.00		
CONTROL_WORRY_Y2	-0.01	0.01	0.00	
FEEL_ANXIOUS_Y2	0.01	-0.01	0.00	

2.13.2.2 Modification indices

```r
tb_mod_indices(fit, sort.=TRUE, maximum.number = 12)
```

lhs	op	rhs	mi	epc	sepc.lv
INTEREST_Y2	~~	FEEL_ANXIOUS_Y2	2.14	-0.04	-0.04
INTEREST_Y2	~~	CONTROL_WORRY_Y2	2.14	0.03	0.03
DEPRESSED_Y2	~~	CONTROL_WORRY_Y2	2.14	-0.04	-0.04
DEPRESSED_Y2	~~	FEEL_ANXIOUS_Y2	2.14	0.04	0.04

2.14 Nigeria

2.14.1 Sample statistics

The following sample statistics, computed using the survey design adjusted estimates from the survey package, are used are input for the factor analyses. This facilitates replication of all analyses without the need to get the raw data from the Center for Open Science.

```
ci <- ci + 1
cur.country <- names(sample.stats)[ci]
tb_sample_stats(sample.stats[[cur.country]])
```

Variable	DEPRESSED_Y2	INTEREST_Y2	CONTROL_WORRY_Y2
DEPRESSED_Y2	1.00	0.45	0.46
INTEREST_Y2	0.45	1.00	0.41
CONTROL_WORRY_Y2	0.46	0.41	1.00
FEEL_ANXIOUS_Y2	0.50	0.39	0.49
X			
Mean	1.24	1.48	1.21
Standard.Deviation	1.00	1.00	1.00
X.1			
Category	0.00	1.00	2.00
DEPRESSED_Y2.1	30.10	31.18	23.70
INTEREST_Y2.1	20.43	33.85	22.95
CONTROL_WORRY_Y2.1	32.11	31.56	19.43
FEEL_ANXIOUS_Y2.1	36.57	30.52	20.59

2.14.2 Confirmatory factor analysis

```
fit <- cfa(
  mod, std.lv = TRUE, ordered=TRUE
  , sample.cov = sample.stats[[cur.country]]$sample.cov
  , sample.mean = sample.stats[[cur.country]]$sample.mean
```

```r
  , sample.nobs = sample.stats[[cur.country]]$sample.nobs
  , sample.th = sample.stats[[cur.country]]$sample.th
  , WLS.V = sample.stats[[cur.country]]$WLS.V
  , NACOV = sample.stats[[cur.country]]$NACOV
)

dynamic::cfaHB(fit)
```

```
Your DFI cutoffs:
            SRMR  RMSEA CFI   Magnitude
Level-0     0.008 0.039 0.998 NONE
Specificity 95%   95%   95%

Level-1     NONE  NONE  NONE  0.381
Sensitivity 3%    4%    0%

Empirical fit indices:
 Chi-Square  df p-value   SRMR   RMSEA    CFI
      1.861   1      NA  0.009   0.017      1
```

```r
summary(fit, standardized=TRUE, ci=TRUE, fit.measure=TRUE)
```

```
lavaan 0.6-21 ended normally after 15 iterations

  Estimator                                       DWLS
  Optimization method                           NLMINB
  Number of model parameters                        17

  Number of observations                      3146.00011189409

Model Test User Model:
                                              Standard      Scaled
  Test Statistic                                 1.861       2.086
  Degrees of freedom                                 1           1
  P-value (Unknown)                                 NA       0.149
  Scaling correction factor                                  0.892
  Shift parameter                                           -0.000
    simple second-order correction

Model Test Baseline Model:
```

```
  Test statistic                                3021.126     854.455
  Degrees of freedom                                   6           6
  P-value                                             NA       0.000
  Scaling correction factor                                    3.542

User Model versus Baseline Model:

  Comparative Fit Index (CFI)                      1.000       0.999
  Tucker-Lewis Index (TLI)                         0.998       0.992

  Robust Comparative Fit Index (CFI)                           0.998
  Robust Tucker-Lewis Index (TLI)                              0.991

Root Mean Square Error of Approximation:

  RMSEA                                            0.017       0.019
  90 Percent confidence interval - lower           0.000       0.000
  90 Percent confidence interval - upper           0.054       0.055
  P-value H_0: RMSEA <= 0.050                      0.925       0.913
  P-value H_0: RMSEA >= 0.080                      0.001       0.001

  Robust RMSEA                                                 0.039
  90 Percent confidence interval - lower                       0.000
  90 Percent confidence interval - upper                       0.114
  P-value H_0: Robust RMSEA <= 0.050                           0.464
  P-value H_0: Robust RMSEA >= 0.080                           0.237

Standardized Root Mean Square Residual:

  SRMR                                             0.009       0.009

Parameter Estimates:

  Parameterization                                 Delta
  Standard errors                             Robust.sem
  Information                                   Expected
  Information saturated (h1) model          Unstructured

Latent Variables:
                   Estimate  Std.Err  z-value  P(>|z|) ci.lower ci.upper   Std.lv  Std.all
  dep =~
    DEPRESSED_Y2      0.736    0.030   24.765    0.000    0.677    0.794    0.736    0.736
    INTEREST_Y2       0.613    0.037   16.623    0.000    0.541    0.686    0.613    0.613
```

```
  anx =~
    CONTROL_WORRY_    0.690    0.035   19.555    0.000    0.621    0.759    0.690    0.690
    FEEL_ANXIOUS_Y    0.709    0.028   25.746    0.000    0.655    0.763    0.709    0.709

Covariances:
                   Estimate  Std.Err  z-value  P(>|z|) ci.lower ci.upper   Std.lv  Std.all
  dep ~~
    anx               0.939    0.045   20.911    0.000    0.851    1.027    0.939    0.939

Thresholds:
                   Estimate  Std.Err  z-value  P(>|z|) ci.lower ci.upper   Std.lv  Std.all
    DEPRESSED_Y2|1   -0.522    0.037  -13.982    0.000   -0.595   -0.448   -
0.522   -0.522
    DEPRESSED_Y2|2    0.287    0.039    7.386    0.000    0.211    0.363    0.287    0.287
    DEPRESSED_Y2|3    1.036    0.045   22.988    0.000    0.947    1.124    1.036    1.036
    INTEREST_Y2|t1   -0.827    0.037  -22.216    0.000   -0.899   -0.754   -
0.827   -0.827
    INTEREST_Y2|t2    0.107    0.036    3.014    0.003    0.038    0.177    0.107    0.107
    INTEREST_Y2|t3    0.746    0.034   21.710    0.000    0.679    0.814    0.746    0.746
    CONTROL_WORRY_   -0.465    0.039  -11.833    0.000   -0.541   -0.388   -
0.465   -0.465
    CONTROL_WORRY_    0.350    0.040    8.715    0.000    0.271    0.429    0.350    0.350
    CONTROL_WORRY_    0.958    0.039   24.579    0.000    0.882    1.035    0.958    0.958
    FEEL_ANXIOUS_Y   -0.343    0.037   -9.208    0.000   -0.416   -0.270   -
0.343   -0.343
    FEEL_ANXIOUS_Y    0.442    0.037   11.911    0.000    0.370    0.515    0.442    0.442
    FEEL_ANXIOUS_Y    1.159    0.039   29.553    0.000    1.082    1.236    1.159    1.159

Variances:
                   Estimate  Std.Err  z-value  P(>|z|) ci.lower ci.upper   Std.lv  Std.all
   .DEPRESSED_Y2      0.459                               0.459    0.459    0.459    0.459
   .INTEREST_Y2       0.624                               0.624    0.624    0.624    0.624
   .CONTROL_WORRY_    0.524                               0.524    0.524    0.524    0.524
   .FEEL_ANXIOUS_Y    0.498                               0.498    0.498    0.498    0.498
    dep               1.000                               1.000    1.000    1.000    1.000
    anx               1.000                               1.000    1.000    1.000    1.000
```

```r
LIST.CFA.FIT[[cur.country]] <- fit
```

2.14.2.1 Residual correlations

Residual correlation greater than |0.05| are bolded.

```r
tb_residual_cor(fit)
```

Variable	DEPRESSED_Y2	INTEREST_Y2	CONTROL_WORRY_Y2	FE
DEPRESSED_Y2	0.00			
INTEREST_Y2	0.00	0.00		
CONTROL_WORRY_Y2	-0.01	0.02	0.00	
FEEL_ANXIOUS_Y2	0.01	-0.02	-0.00	

2.14.2.2 Modification indices

```r
tb_mod_indices(fit, sort.=TRUE, maximum.number = 12)
```

lhs	op	rhs	mi	epc	sepc.lv
INTEREST_Y2	~~	CONTROL_WORRY_Y2	1.86	0.05	0.05
DEPRESSED_Y2	~~	CONTROL_WORRY_Y2	1.86	-0.06	-0.06
INTEREST_Y2	~~	FEEL_ANXIOUS_Y2	1.86	-0.05	-0.05
DEPRESSED_Y2	~~	FEEL_ANXIOUS_Y2	1.86	0.06	0.06

2.15 Philippines

2.15.1 Sample statistics

The following sample statistics, computed using the survey design adjusted estimates from the survey package, are used are input for the factor analyses. This facilitates replication of all analyses without the need to get the raw data from the Center for Open Science.

```
ci <- ci + 1
cur.country <- names(sample.stats)[ci]
tb_sample_stats(sample.stats[[cur.country]])
```

Variable	DEPRESSED_Y2	INTEREST_Y2	CONTROL_WORRY_Y2	F
DEPRESSED_Y2	1.00	0.42	0.49	
INTEREST_Y2	0.42	1.00	0.41	
CONTROL_WORRY_Y2	0.49	0.41	1.00	
FEEL_ANXIOUS_Y2	0.57	0.41	0.62	
X				
Mean	1.07	1.46	1.44	
Standard.Deviation	1.00	1.00	1.00	
X.1				
Category	0.00	1.00	2.00	
DEPRESSED_Y2.1	38.64	28.22	21.06	
INTEREST_Y2.1	22.35	29.26	28.34	
CONTROL_WORRY_Y2.1	28.33	25.92	18.63	
FEEL_ANXIOUS_Y2.1	40.50	25.96	21.13	

2.15.2 Confirmatory factor analysis

```
fit <- cfa(
  mod, std.lv = TRUE, ordered=TRUE
  , sample.cov = sample.stats[[cur.country]]$sample.cov
  , sample.mean = sample.stats[[cur.country]]$sample.mean
```

```r
  , sample.nobs = sample.stats[[cur.country]]$sample.nobs
  , sample.th = sample.stats[[cur.country]]$sample.th
  , WLS.V = sample.stats[[cur.country]]$WLS.V
  , NACOV = sample.stats[[cur.country]]$NACOV
)

dynamic::cfaHB(fit)
```

```
Your DFI cutoffs:
            SRMR  RMSEA CFI   Magnitude
Level-0     0.007 0.04  0.999 NONE
Specificity 95%   95%   95%

Level-1     NONE  NONE  NONE  0.429
Sensitivity 2%    4%    0%

Empirical fit indices:
 Chi-Square  df p-value   SRMR   RMSEA    CFI
      3.145   1      NA   0.01   0.028      1
```

```r
summary(fit, standardized=TRUE, ci=TRUE, fit.measure=TRUE)
```

```
lavaan 0.6-21 ended normally after 12 iterations

  Estimator                                         DWLS
  Optimization method                             NLMINB
  Number of model parameters                          17

  Number of observations                        2682.00024216278

Model Test User Model:
                                              Standard      Scaled
  Test Statistic                                 3.145       2.349
  Degrees of freedom                                 1           1
  P-value (Unknown)                                 NA       0.125
  Scaling correction factor                                  1.339
  Shift parameter                                            0.000
    simple second-order correction

Model Test Baseline Model:
```

```
  Test statistic                              6177.145    2316.117
  Degrees of freedom                                 6           6
  P-value                                           NA       0.000
  Scaling correction factor                                  2.668

User Model versus Baseline Model:

  Comparative Fit Index (CFI)                    1.000       0.999
  Tucker-Lewis Index (TLI)                       0.998       0.996

  Robust Comparative Fit Index (CFI)                         0.998
  Robust Tucker-Lewis Index (TLI)                            0.988

Root Mean Square Error of Approximation:

  RMSEA                                          0.028       0.022
  90 Percent confidence interval - lower         0.000       0.000
  90 Percent confidence interval - upper         0.066       0.061
  P-value H_0: RMSEA <= 0.050                    0.793       0.855
  P-value H_0: RMSEA >= 0.080                    0.009       0.005

  Robust RMSEA                                               0.049
  90 Percent confidence interval - lower                     0.000
  90 Percent confidence interval - upper                     0.133
  P-value H_0: Robust RMSEA <= 0.050                         0.371
  P-value H_0: Robust RMSEA >= 0.080                         0.352

Standardized Root Mean Square Residual:

  SRMR                                           0.010       0.010

Parameter Estimates:

  Parameterization                               Delta
  Standard errors                           Robust.sem
  Information                                 Expected
  Information saturated (h1) model        Unstructured

Latent Variables:
                   Estimate  Std.Err  z-value  P(>|z|) ci.lower ci.upper   Std.lv  Std.all
  dep =~
    DEPRESSED_Y2      0.737    0.029   25.027    0.000    0.679    0.795    0.737    0.737
    INTEREST_Y2       0.571    0.026   22.245    0.000    0.521    0.621    0.571    0.571
```

```
  anx =~
    CONTROL_WORRY_    0.747    0.023   32.162    0.000    0.702    0.793    0.747    0.747
    FEEL_ANXIOUS_Y    0.833    0.021   39.309    0.000    0.791    0.874    0.833    0.833

Covariances:
                   Estimate  Std.Err  z-value  P(>|z|) ci.lower ci.upper   Std.lv  Std.all
  dep ~~
    anx               0.913    0.034   26.654    0.000    0.846    0.980    0.913    0.913

Thresholds:
                   Estimate  Std.Err  z-value  P(>|z|) ci.lower ci.upper   Std.lv  Std.all
    DEPRESSED_Y2|1   -0.289    0.040   -7.295    0.000   -0.366   -0.211   -
0.289   -0.289
    DEPRESSED_Y2|2    0.436    0.031   13.959    0.000    0.375    0.497    0.436    0.436
    DEPRESSED_Y2|3    1.171    0.039   29.661    0.000    1.094    1.248    1.171    1.171
    INTEREST_Y2|t1   -0.760    0.040  -18.816    0.000   -0.840   -0.681   -
0.760   -0.760
    INTEREST_Y2|t2    0.041    0.029    1.382    0.167   -0.017    0.098    0.041    0.041
    INTEREST_Y2|t3    0.840    0.035   23.864    0.000    0.771    0.909    0.840    0.840
    CONTROL_WORRY_   -0.573    0.030  -19.118    0.000   -0.632   -0.514   -
0.573   -0.573
    CONTROL_WORRY_    0.107    0.030    3.576    0.000    0.048    0.165    0.107    0.107
    CONTROL_WORRY_    0.609    0.032   19.111    0.000    0.547    0.672    0.609    0.609
    FEEL_ANXIOUS_Y   -0.240    0.035   -6.822    0.000   -0.309   -0.171   -
0.240   -0.240
    FEEL_ANXIOUS_Y    0.425    0.033   13.031    0.000    0.361    0.489    0.425    0.425
    FEEL_ANXIOUS_Y    1.155    0.044   26.108    0.000    1.068    1.241    1.155    1.155

Variances:
                   Estimate  Std.Err  z-value  P(>|z|) ci.lower ci.upper   Std.lv  Std.all
   .DEPRESSED_Y2      0.457                               0.457    0.457    0.457    0.457
   .INTEREST_Y2       0.674                               0.674    0.674    0.674    0.674
   .CONTROL_WORRY_    0.442                               0.442    0.442    0.442    0.442
   .FEEL_ANXIOUS_Y    0.307                               0.307    0.307    0.307    0.307
    dep               1.000                               1.000    1.000    1.000    1.000
    anx               1.000                               1.000    1.000    1.000    1.000
```

```
LIST.CFA.FIT[[cur.country]] <- fit
```

2.15.2.1 Residual correlations

Residual correlation greater than |0.05| are bolded.

```r
tb_residual_cor(fit)
```

Variable	DEPRESSED_Y2	INTEREST_Y2	CONTROL_WORRY_Y2	FE
DEPRESSED_Y2	0.00			
INTEREST_Y2	-0.00	0.00		
CONTROL_WORRY_Y2	-0.01	0.02	0.00	
FEEL_ANXIOUS_Y2	0.01	-0.02	-0.00	

2.15.2.2 Modification indices

```r
tb_mod_indices(fit, sort.=TRUE, maximum.number = 12)
```

lhs	op	rhs	mi	epc	sepc.lv
DEPRESSED_Y2	~~	FEEL_ANXIOUS_Y2	3.14	0.07	0.07
DEPRESSED_Y2	~~	CONTROL_WORRY_Y2	3.14	-0.07	-0.07
INTEREST_Y2	~~	FEEL_ANXIOUS_Y2	3.14	-0.06	-0.06
INTEREST_Y2	~~	CONTROL_WORRY_Y2	3.14	0.05	0.05

2.16 Poland

2.16.1 Sample statistics

The following sample statistics, computed using the survey design adjusted estimates from the survey package, are used are input for the factor analyses. This facilitates replication of all analyses without the need to get the raw data from the Center for Open Science.

```
ci <- ci + 1
cur.country <- names(sample.stats)[ci]
tb_sample_stats(sample.stats[[cur.country]])
```

Variable	DEPRESSED_Y2	INTEREST_Y2	CONTROL_WORRY_Y2
DEPRESSED_Y2	1.00	0.73	0.77
INTEREST_Y2	0.73	1.00	0.67
CONTROL_WORRY_Y2	0.77	0.67	1.00
FEEL_ANXIOUS_Y2	0.69	0.61	0.75
X			
Mean	0.38	0.56	0.37
Standard.Deviation	1.00	1.00	1.00
X.1			
Category	0.00	1.00	2.00
DEPRESSED_Y2.1	69.77	23.50	4.95
INTEREST_Y2.1	56.19	33.19	8.38
CONTROL_WORRY_Y2.1	70.49	22.86	4.92
FEEL_ANXIOUS_Y2.1	53.32	38.43	6.29

2.16.2 Confirmatory factor analysis

```
fit <- cfa(
  mod, std.lv = TRUE, ordered=TRUE
  , sample.cov = sample.stats[[cur.country]]$sample.cov
  , sample.mean = sample.stats[[cur.country]]$sample.mean
```

```r
  , sample.nobs = sample.stats[[cur.country]]$sample.nobs
  , sample.th = sample.stats[[cur.country]]$sample.th
  , WLS.V = sample.stats[[cur.country]]$WLS.V
  , NACOV = sample.stats[[cur.country]]$NACOV
)

dynamic::cfaHB(fit)
```

```
Your DFI cutoffs:
            SRMR  RMSEA CFI  Magnitude
Level-0     0.004 0.036 1    NONE
Specificity 95%   95%   95%

Level-1     NONE  NONE  NONE 0.201
Sensitivity 2%    7%    3%

Empirical fit indices:
 Chi-Square  df p-value   SRMR   RMSEA    CFI
      0.391   1      NA  0.002       0      1
```

```r
summary(fit, standardized=TRUE, ci=TRUE, fit.measure=TRUE)
```

```
lavaan 0.6-21 ended normally after 12 iterations

  Estimator                                       DWLS
  Optimization method                           NLMINB
  Number of model parameters                        17

  Number of observations                        6478.00001620523

Model Test User Model:
                                              Standard      Scaled
  Test Statistic                                 0.391       0.396
  Degrees of freedom                                 1           1
  P-value (Unknown)                                 NA       0.529
  Scaling correction factor                                  0.988
  Shift parameter                                            0.000
    simple second-order correction

Model Test Baseline Model:
```

```
  Test statistic                             36103.473    5946.254
  Degrees of freedom                                 6           6
  P-value                                           NA       0.000
  Scaling correction factor                                  6.074

User Model versus Baseline Model:

  Comparative Fit Index (CFI)                    1.000       1.000
  Tucker-Lewis Index (TLI)                       1.000       1.001

  Robust Comparative Fit Index (CFI)                         1.000
  Robust Tucker-Lewis Index (TLI)                            1.002

Root Mean Square Error of Approximation:

  RMSEA                                          0.000       0.000
  90 Percent confidence interval - lower         0.000       0.000
  90 Percent confidence interval - upper         0.028       0.028
  P-value H_0: RMSEA <= 0.050                    1.000       1.000
  P-value H_0: RMSEA >= 0.080                    0.000       0.000

  Robust RMSEA                                               0.000
  90 Percent confidence interval - lower                     0.000
  90 Percent confidence interval - upper                     0.089
  P-value H_0: Robust RMSEA <= 0.050                         0.768
  P-value H_0: Robust RMSEA >= 0.080                         0.076

Standardized Root Mean Square Residual:

  SRMR                                           0.002       0.002

Parameter Estimates:

  Parameterization                               Delta
  Standard errors                           Robust.sem
  Information                                 Expected
  Information saturated (h1) model        Unstructured

Latent Variables:
                   Estimate  Std.Err  z-value  P(>|z|) ci.lower ci.upper   Std.lv  Std.all
  dep =~
    DEPRESSED_Y2      0.915    0.010   94.090    0.000    0.895    0.934    0.915    0.915
    INTEREST_Y2       0.802    0.014   57.049    0.000    0.774    0.829    0.802    0.802
```

```
  anx =~
    CONTROL_WORRY_    0.918    0.012   73.621    0.000    0.893    0.942    0.918    0.918
    FEEL_ANXIOUS_Y    0.821    0.013   63.136    0.000    0.796    0.847    0.821    0.821

Covariances:
                   Estimate  Std.Err  z-value  P(>|z|) ci.lower ci.upper   Std.lv  Std.all
  dep ~~
    anx               0.920    0.014   64.966    0.000    0.892    0.948    0.920    0.920

Thresholds:
                   Estimate  Std.Err  z-value  P(>|z|) ci.lower ci.upper   Std.lv  Std.all
    DEPRESSED_Y2|1    0.518    0.032   15.972    0.000    0.454    0.581    0.518    0.518
    DEPRESSED_Y2|2    1.496    0.042   35.516    0.000    1.413    1.579    1.496    1.496
    DEPRESSED_Y2|3    2.101    0.060   35.228    0.000    1.985    2.218    2.101    2.101
    INTEREST_Y2|t1    0.156    0.036    4.282    0.000    0.084    0.227    0.156    0.156
    INTEREST_Y2|t2    1.247    0.039   31.884    0.000    1.170    1.323    1.247    1.247
    INTEREST_Y2|t3    2.007    0.056   35.878    0.000    1.897    2.116    2.007    2.007
    CONTROL_WORRY_    0.539    0.035   15.463    0.000    0.470    0.607    0.539    0.539
    CONTROL_WORRY_    1.503    0.037   40.584    0.000    1.430    1.575    1.503    1.503
    CONTROL_WORRY_    2.115    0.065   32.401    0.000    1.987    2.243    2.115    2.115
    FEEL_ANXIOUS_Y    0.083    0.035    2.394    0.017    0.015    0.152    0.083    0.083
    FEEL_ANXIOUS_Y    1.389    0.031   44.549    0.000    1.328    1.450    1.389    1.389
    FEEL_ANXIOUS_Y    2.063    0.054   38.012    0.000    1.957    2.169    2.063    2.063

Variances:
                   Estimate  Std.Err  z-value  P(>|z|) ci.lower ci.upper   Std.lv  Std.all
   .DEPRESSED_Y2      0.164                               0.164    0.164    0.164    0.164
   .INTEREST_Y2       0.357                               0.357    0.357    0.357    0.357
   .CONTROL_WORRY_    0.158                               0.158    0.158    0.158    0.158
   .FEEL_ANXIOUS_Y    0.326                               0.326    0.326    0.326    0.326
    dep               1.000                               1.000    1.000    1.000    1.000
    anx               1.000                               1.000    1.000    1.000    1.000
```

```
LIST.CFA.FIT[[cur.country]] <- fit
```

2.16.2.1 Residual correlations

Residual correlation greater than |0.05| are bolded.

```
tb_residual_cor(fit)
```

Variable	DEPRESSED_Y2	INTEREST_Y2	CONTROL_WORRY_Y2	FE
DEPRESSED_Y2	0.00			
INTEREST_Y2	-0.00	0.00		
CONTROL_WORRY_Y2	0.00	-0.00	0.00	
FEEL_ANXIOUS_Y2	-0.00	0.00	-0.00	

2.16.2.2 Modification indices

```
tb_mod_indices(fit, sort.=TRUE, maximum.number = 12)
```

lhs	op	rhs	mi	epc	sepc.lv
DEPRESSED_Y2	~~	FEEL_ANXIOUS_Y2	0.39	-0.01	-0.01
INTEREST_Y2	~~	FEEL_ANXIOUS_Y2	0.39	0.01	0.01
DEPRESSED_Y2	~~	CONTROL_WORRY_Y2	0.39	0.01	0.01
INTEREST_Y2	~~	CONTROL_WORRY_Y2	0.39	-0.01	-0.01

2.17 South Africa

2.17.1 Sample statistics

The following sample statistics, computed using the survey design adjusted estimates from the survey package, are used are input for the factor analyses. This facilitates replication of all analyses without the need to get the raw data from the Center for Open Science.

```
ci <- ci + 1
cur.country <- names(sample.stats)[ci]
tb_sample_stats(sample.stats[[cur.country]])
```

Variable	DEPRESSED_Y2	INTEREST_Y2	CONTROL_WORRY_Y2	[illegible]
DEPRESSED_Y2	1.00	0.45	0.47	
INTEREST_Y2	0.45	1.00	0.42	
CONTROL_WORRY_Y2	0.47	0.42	1.00	
FEEL_ANXIOUS_Y2	0.47	0.41	0.47	
X				
Mean	1.13	1.08	1.23	
Standard.Deviation	1.00	1.00	1.00	
X.1				
Category	0.00	1.00	2.00	
DEPRESSED_Y2.1	35.32	32.85	15.70	
INTEREST_Y2.1	34.04	37.50	14.71	
CONTROL_WORRY_Y2.1	31.33	33.29	16.59	
FEEL_ANXIOUS_Y2.1	37.90	32.24	15.61	

2.17.2 Confirmatory factor analysis

```
fit <- cfa(
  mod, std.lv = TRUE, ordered=TRUE
  , sample.cov = sample.stats[[cur.country]]$sample.cov
  , sample.mean = sample.stats[[cur.country]]$sample.mean
```

```r
  , sample.nobs = sample.stats[[cur.country]]$sample.nobs
  , sample.th = sample.stats[[cur.country]]$sample.th
  , WLS.V = sample.stats[[cur.country]]$WLS.V
  , NACOV = sample.stats[[cur.country]]$NACOV
)

dynamic::cfaHB(fit)
```

```
Your DFI cutoffs:
            SRMR  RMSEA CFI   Magnitude
Level-0     0.012 0.06  0.996 NONE
Specificity 95%   95%   95%

Level-1     NONE  NONE  NONE  0.364
Sensitivity 2%    5%    0%

Empirical fit indices:
 Chi-Square  df p-value   SRMR   RMSEA    CFI
      0.004   1      NA  0.001       0      1
```

```r
summary(fit, standardized=TRUE, ci=TRUE, fit.measure=TRUE)
```

```
lavaan 0.6-21 ended normally after 12 iterations

  Estimator                                       DWLS
  Optimization method                           NLMINB
  Number of model parameters                        17

  Number of observations                      978.000000002357

Model Test User Model:
                                              Standard      Scaled
  Test Statistic                                 0.004       0.002
  Degrees of freedom                                 1           1
  P-value (Unknown)                                 NA       0.961
  Scaling correction factor                                  1.662
  Shift parameter                                           -0.000
    simple second-order correction

Model Test Baseline Model:
```

```
  Test statistic                              1256.426     462.507
  Degrees of freedom                                 6           6
  P-value                                           NA       0.000
  Scaling correction factor                                  2.723

User Model versus Baseline Model:

  Comparative Fit Index (CFI)                    1.000       1.000
  Tucker-Lewis Index (TLI)                       1.005       1.013

  Robust Comparative Fit Index (CFI)                         1.000
  Robust Tucker-Lewis Index (TLI)                            1.043

Root Mean Square Error of Approximation:

  RMSEA                                          0.000       0.000
  90 Percent confidence interval - lower         0.000       0.000
  90 Percent confidence interval - upper         0.005       0.000
  P-value H_0: RMSEA <= 0.050                    0.985       0.988
  P-value H_0: RMSEA >= 0.080                    0.002       0.002

  Robust RMSEA                                               0.000
  90 Percent confidence interval - lower                     0.000
  90 Percent confidence interval - upper                     0.000
  P-value H_0: Robust RMSEA <= 0.050                         0.967
  P-value H_0: Robust RMSEA >= 0.080                         0.024

Standardized Root Mean Square Residual:

  SRMR                                           0.001       0.001

Parameter Estimates:

  Parameterization                               Delta
  Standard errors                           Robust.sem
  Information                                 Expected
  Information saturated (h1) model        Unstructured

Latent Variables:
                   Estimate  Std.Err  z-value  P(>|z|) ci.lower ci.upper   Std.lv  Std.all
  dep =~
    DEPRESSED_Y2      0.718    0.053   13.557    0.000    0.614    0.822    0.718    0.718
    INTEREST_Y2       0.628    0.041   15.361    0.000    0.548    0.708    0.628    0.628
```

```
  anx =~
    CONTROL_WORRY_    0.692    0.044   15.672    0.000    0.605    0.778    0.692    0.692
    FEEL_ANXIOUS_Y    0.685    0.050   13.565    0.000    0.586    0.784    0.685    0.685

Covariances:
                   Estimate  Std.Err  z-value  P(>|z|) ci.lower ci.upper   Std.lv  Std.all
  dep ~~
    anx               0.954    0.059   16.110    0.000    0.838    1.070    0.954    0.954

Thresholds:
                   Estimate  Std.Err  z-value  P(>|z|) ci.lower ci.upper   Std.lv  Std.all
    DEPRESSED_Y2|1   -0.377    0.070   -5.366    0.000   -0.514   -0.239   -
0.377   -0.377
    DEPRESSED_Y2|2    0.472    0.065    7.311    0.000    0.346    0.599    0.472    0.472
    DEPRESSED_Y2|3    0.989    0.071   13.982    0.000    0.850    1.128    0.989    0.989
    INTEREST_Y2|t1   -0.411    0.075   -5.516    0.000   -0.558   -0.265   -
0.411   -0.411
    INTEREST_Y2|t2    0.569    0.062    9.217    0.000    0.448    0.690    0.569    0.569
    INTEREST_Y2|t3    1.091    0.068   16.114    0.000    0.959    1.224    1.091    1.091
    CONTROL_WORRY_   -0.486    0.058   -8.444    0.000   -0.599   -0.374   -
0.486   -0.486
    CONTROL_WORRY_    0.375    0.055    6.778    0.000    0.267    0.484    0.375    0.375
    CONTROL_WORRY_    0.886    0.067   13.267    0.000    0.755    1.016    0.886    0.886
    FEEL_ANXIOUS_Y   -0.308    0.065   -4.754    0.000   -0.435   -0.181   -
0.308   -0.308
    FEEL_ANXIOUS_Y    0.529    0.059    9.003    0.000    0.413    0.644    0.529    0.529
    FEEL_ANXIOUS_Y    1.069    0.068   15.726    0.000    0.936    1.203    1.069    1.069

Variances:
                   Estimate  Std.Err  z-value  P(>|z|) ci.lower ci.upper   Std.lv  Std.all
   .DEPRESSED_Y2      0.484                               0.484    0.484    0.484    0.484
   .INTEREST_Y2       0.605                               0.605    0.605    0.605    0.605
   .CONTROL_WORRY_    0.522                               0.522    0.522    0.522    0.522
   .FEEL_ANXIOUS_Y    0.531                               0.531    0.531    0.531    0.531
    dep               1.000                               1.000    1.000    1.000    1.000
    anx               1.000                               1.000    1.000    1.000    1.000
```

```r
LIST.CFA.FIT[[cur.country]] <- fit
```

2.17.2.1 Residual correlations

Residual correlation greater than |0.05| are bolded.

```r
tb_residual_cor(fit)
```

Variable	DEPRESSED_Y2	INTEREST_Y2	CONTROL_WORRY_Y2	FE
DEPRESSED_Y2	0.00			
INTEREST_Y2	0.00	0.00		
CONTROL_WORRY_Y2	-0.00	0.00	0.00	
FEEL_ANXIOUS_Y2	0.00	-0.00	-0.00	

2.17.2.2 Modification indices

```r
tb_mod_indices(fit, sort.=TRUE, maximum.number = 12)
```

lhs	op	rhs	mi	epc	sepc.
INTEREST_Y2	~~	CONTROL_WORRY_Y2	0.00	0.00	0.0[illegible]
INTEREST_Y2	~~	FEEL_ANXIOUS_Y2	0.00	-0.00	-0.0
DEPRESSED_Y2	~~	FEEL_ANXIOUS_Y2	0.00	0.00	0.0[illegible]
DEPRESSED_Y2	~~	CONTROL_WORRY_Y2	0.00	-0.00	-0.0

2.18 Spain

2.18.1 Sample statistics

The following sample statistics, computed using the survey design adjusted estimates from the survey package, are used are input for the factor analyses. This facilitates replication of all analyses without the need to get the raw data from the Center for Open Science.

```
ci <- ci + 1
cur.country <- names(sample.stats)[ci]
tb_sample_stats(sample.stats[[cur.country]])
```

Variable	DEPRESSED_Y2	INTEREST_Y2	CONTROL_WORRY_Y2
DEPRESSED_Y2	1.00	0.70	0.71
INTEREST_Y2	0.70	1.00	0.64
CONTROL_WORRY_Y2	0.71	0.64	1.00
FEEL_ANXIOUS_Y2	0.72	0.60	0.71
X			
Mean	0.93	1.02	0.95
Standard.Deviation	1.00	1.00	1.00
X.1			
Category	0.00	1.00	2.00
DEPRESSED_Y2.1	41.07	35.28	13.61
INTEREST_Y2.1	33.09	40.96	16.59
CONTROL_WORRY_Y2.1	41.77	31.81	15.81
FEEL_ANXIOUS_Y2.1	25.20	43.82	16.48

2.18.2 Confirmatory factor analysis

```
fit <- cfa(
  mod, std.lv = TRUE, ordered=TRUE
  , sample.cov = sample.stats[[cur.country]]$sample.cov
  , sample.mean = sample.stats[[cur.country]]$sample.mean
```

```r
  , sample.nobs = sample.stats[[cur.country]]$sample.nobs
  , sample.th = sample.stats[[cur.country]]$sample.th
  , WLS.V = sample.stats[[cur.country]]$WLS.V
  , NACOV = sample.stats[[cur.country]]$NACOV
)

dynamic::cfaHB(fit)
```

```
Your DFI cutoffs:
            SRMR  RMSEA CFI   Magnitude
Level-0     0.004 0.038 0.999 NONE
Specificity 95%   95%   95%

Level-1     NONE  NONE  NONE  0.22
Sensitivity 1%    5%    2%

Empirical fit indices:
 Chi-Square  df p-value   SRMR   RMSEA    CFI
      4.755   1      NA  0.009   0.036      1
```

```r
summary(fit, standardized=TRUE, ci=TRUE, fit.measure=TRUE)
```

```
lavaan 0.6-21 ended normally after 14 iterations

  Estimator                                       DWLS
  Optimization method                           NLMINB
  Number of model parameters                        17

  Number of observations                      2924.00000076902

Model Test User Model:
                                              Standard      Scaled
  Test Statistic                                 4.755       3.249
  Degrees of freedom                                 1           1
  P-value (Unknown)                                 NA       0.071
  Scaling correction factor                                  1.463
  Shift parameter                                           -0.000
    simple second-order correction

Model Test Baseline Model:
```

```
  Test statistic                             18286.175    4425.760
  Degrees of freedom                                 6           6
  P-value                                           NA       0.000
  Scaling correction factor                                  4.133

User Model versus Baseline Model:

  Comparative Fit Index (CFI)                    1.000       0.999
  Tucker-Lewis Index (TLI)                       0.999       0.997

  Robust Comparative Fit Index (CFI)                         0.997
  Robust Tucker-Lewis Index (TLI)                            0.985

Root Mean Square Error of Approximation:

  RMSEA                                          0.036       0.028
  90 Percent confidence interval - lower         0.009       0.000
  90 Percent confidence interval - upper         0.071       0.064
  P-value H_0: RMSEA <= 0.050                    0.699       0.816
  P-value H_0: RMSEA >= 0.080                    0.016       0.006

  Robust RMSEA                                               0.078
  90 Percent confidence interval - lower                     0.000
  90 Percent confidence interval - upper                     0.179
  P-value H_0: Robust RMSEA <= 0.050                         0.203
  P-value H_0: Robust RMSEA >= 0.080                         0.603

Standardized Root Mean Square Residual:

  SRMR                                           0.009       0.009

Parameter Estimates:

  Parameterization                               Delta
  Standard errors                           Robust.sem
  Information                                 Expected
  Information saturated (h1) model        Unstructured

Latent Variables:
                   Estimate  Std.Err  z-value  P(>|z|) ci.lower ci.upper   Std.lv  Std.all
  dep =~
    DEPRESSED_Y2      0.899    0.017   54.413    0.000    0.867    0.932    0.899    0.899
    INTEREST_Y2       0.778    0.017   44.785    0.000    0.744    0.812    0.778    0.778
```

```
  anx =~
    CONTROL_WORRY_    0.850    0.016   54.555    0.000    0.819    0.880    0.850    0.850
    FEEL_ANXIOUS_Y    0.831    0.017   49.813    0.000    0.798    0.863    0.831    0.831

Covariances:
                   Estimate  Std.Err  z-value  P(>|z|) ci.lower ci.upper   Std.lv  Std.all
  dep ~~
    anx               0.948    0.017   57.070    0.000    0.915    0.981    0.948    0.948

Thresholds:
                   Estimate  Std.Err  z-value  P(>|z|) ci.lower ci.upper   Std.lv  Std.all
    DEPRESSED_Y2|1   -0.226    0.038   -5.957    0.000   -0.300   -0.152   -
0.226   -0.226
    DEPRESSED_Y2|2    0.718    0.040   17.837    0.000    0.639    0.796    0.718    0.718
    DEPRESSED_Y2|3    1.279    0.049   26.252    0.000    1.183    1.374    1.279    1.279
    INTEREST_Y2|t1   -0.437    0.034  -12.969    0.000   -0.504   -0.371   -
0.437   -0.437
    INTEREST_Y2|t2    0.645    0.038   17.035    0.000    0.571    0.719    0.645    0.645
    INTEREST_Y2|t3    1.319    0.053   25.090    0.000    1.216    1.422    1.319    1.319
    CONTROL_WORRY_   -0.208    0.032   -6.541    0.000   -0.270   -0.146   -
0.208   -0.208
    CONTROL_WORRY_    0.630    0.042   15.190    0.000    0.549    0.712    0.630    0.630
    CONTROL_WORRY_    1.247    0.047   26.707    0.000    1.156    1.339    1.247    1.247
    FEEL_ANXIOUS_Y   -0.668    0.030  -22.188    0.000   -0.727   -0.609   -
0.668   -0.668
    FEEL_ANXIOUS_Y    0.496    0.033   15.109    0.000    0.432    0.561    0.496    0.496
    FEEL_ANXIOUS_Y    1.058    0.041   26.040    0.000    0.978    1.138    1.058    1.058

Variances:
                   Estimate  Std.Err  z-value  P(>|z|) ci.lower ci.upper   Std.lv  Std.all
   .DEPRESSED_Y2      0.191                               0.191    0.191    0.191    0.191
   .INTEREST_Y2       0.395                               0.395    0.395    0.395    0.395
   .CONTROL_WORRY_    0.278                               0.278    0.278    0.278    0.278
   .FEEL_ANXIOUS_Y    0.310                               0.310    0.310    0.310    0.310
    dep               1.000                               1.000    1.000    1.000    1.000
    anx               1.000                               1.000    1.000    1.000    1.000
```

```r
LIST.CFA.FIT[[cur.country]] <- fit
```

2.18.2.1 Residual correlations

Residual correlation greater than |0.05| are bolded.

```r
tb_residual_cor(fit)
```

Variable	DEPRESSED_Y2	INTEREST_Y2	CONTROL_WORRY_Y2	FE
DEPRESSED_Y2	0.00			
INTEREST_Y2	-0.00	0.00		
CONTROL_WORRY_Y2	-0.01	0.01	0.00	
FEEL_ANXIOUS_Y2	0.01	-0.02	-0.00	

2.18.2.2 Modification indices

```r
tb_mod_indices(fit, sort.=TRUE, maximum.number = 12)
```

lhs	op	rhs	mi	epc	sepc.lv
INTEREST_Y2	~~	FEEL_ANXIOUS_Y2	4.75	-0.05	-0.05
DEPRESSED_Y2	~~	CONTROL_WORRY_Y2	4.75	-0.06	-0.06
INTEREST_Y2	~~	CONTROL_WORRY_Y2	4.75	0.05	0.05
DEPRESSED_Y2	~~	FEEL_ANXIOUS_Y2	4.75	0.06	0.06

2.19 Sweden

2.19.1 Sample statistics

The following sample statistics, computed using the survey design adjusted estimates from the survey package, are used are input for the factor analyses. This facilitates replication of all analyses without the need to get the raw data from the Center for Open Science.

```
ci <- ci + 1
cur.country <- names(sample.stats)[ci]
tb_sample_stats(sample.stats[[cur.country]])
```

Variable	DEPRESSED_Y2	INTEREST_Y2	CONTROL_WORRY_Y2	F
DEPRESSED_Y2	1.00	0.65	0.80	
INTEREST_Y2	0.65	1.00	0.57	
CONTROL_WORRY_Y2	0.80	0.57	1.00	
FEEL_ANXIOUS_Y2	0.77	0.53	0.89	
X				
Mean	0.65	0.77	0.54	
Standard.Deviation	1.00	1.00	1.00	
X.1				
Category	0.00	1.00	2.00	
DEPRESSED_Y2.1	55.12	30.78	8.14	
INTEREST_Y2.1	51.20	27.37	14.57	
CONTROL_WORRY_Y2.1	63.25	24.10	7.86	
FEEL_ANXIOUS_Y2.1	47.71	36.97	9.04	

2.19.2 Confirmatory factor analysis

```
fit <- cfa(
  mod, std.lv = TRUE, ordered=TRUE
  , sample.cov = sample.stats[[cur.country]]$sample.cov
  , sample.mean = sample.stats[[cur.country]]$sample.mean
```

```r
  , sample.nobs = sample.stats[[cur.country]]$sample.nobs
  , sample.th = sample.stats[[cur.country]]$sample.th
  , WLS.V = sample.stats[[cur.country]]$WLS.V
  , NACOV = sample.stats[[cur.country]]$NACOV
)

dynamic::cfaHB(fit)
```

```
Your DFI cutoffs:
            SRMR  RMSEA CFI  Magnitude
Level-0     0.003 0.036 1    NONE
Specificity 95%   95%   95%

Level-1     NONE  NONE  NONE 0.337
Sensitivity 0%    5%    1%

Empirical fit indices:
 Chi-Square  df p-value   SRMR   RMSEA    CFI
      6.201   1      NA  0.005   0.021      1
```

```r
summary(fit, standardized=TRUE, ci=TRUE, fit.measure=TRUE)
```

```
lavaan 0.6-21 ended normally after 15 iterations

  Estimator                                         DWLS
  Optimization method                             NLMINB
  Number of model parameters                          17

  Number of observations                        11609.0108778427

Model Test User Model:
                                              Standard      Scaled
  Test Statistic                                 6.201       7.397
  Degrees of freedom                                 1           1
  P-value (Unknown)                                 NA       0.007
  Scaling correction factor                                  0.838
  Shift parameter                                           -0.000
    simple second-order correction

Model Test Baseline Model:
```

```
  Test statistic                              160300.240   32726.852
  Degrees of freedom                                   6           6
  P-value                                             NA       0.000
  Scaling correction factor                                    4.898

User Model versus Baseline Model:

  Comparative Fit Index (CFI)                      1.000       1.000
  Tucker-Lewis Index (TLI)                         1.000       0.999

  Robust Comparative Fit Index (CFI)                           0.999
  Robust Tucker-Lewis Index (TLI)                              0.992

Root Mean Square Error of Approximation:

  RMSEA                                            0.021       0.023
  90 Percent confidence interval - lower           0.008       0.010
  90 Percent confidence interval - upper           0.038       0.041
  P-value H_0: RMSEA <= 0.050                      0.998       0.996
  P-value H_0: RMSEA >= 0.080                      0.000       0.000

  Robust RMSEA                                                 0.065
  90 Percent confidence interval - lower                       0.027
  90 Percent confidence interval - upper                       0.112
  P-value H_0: Robust RMSEA <= 0.050                           0.222
  P-value H_0: Robust RMSEA >= 0.080                           0.343

Standardized Root Mean Square Residual:

  SRMR                                             0.005       0.005

Parameter Estimates:

  Parameterization                                 Delta
  Standard errors                             Robust.sem
  Information                                   Expected
  Information saturated (h1) model          Unstructured

Latent Variables:
                   Estimate  Std.Err  z-value  P(>|z|) ci.lower ci.upper   Std.lv  Std.all
  dep =~
    DEPRESSED_Y2      0.965    0.005  175.714    0.000    0.954    0.976    0.965    0.965
    INTEREST_Y2       0.678    0.011   60.798    0.000    0.656    0.699    0.678    0.678
```

```
  anx =~
    CONTROL_WORRY_    0.961    0.004  245.552    0.000    0.953    0.968    0.961    0.961
    FEEL_ANXIOUS_Y    0.924    0.005  186.890    0.000    0.914    0.934    0.924    0.924

Covariances:
                   Estimate  Std.Err  z-value  P(>|z|) ci.lower ci.upper   Std.lv  Std.all
  dep ~~
    anx               0.865    0.008  102.921    0.000    0.848    0.881    0.865    0.865

Thresholds:
                   Estimate  Std.Err  z-value  P(>|z|) ci.lower ci.upper   Std.lv  Std.all
    DEPRESSED_Y2|1    0.129    0.015    8.671    0.000    0.100    0.158    0.129    0.129
    DEPRESSED_Y2|2    1.076    0.018   58.875    0.000    1.040    1.112    1.076    1.076
    DEPRESSED_Y2|3    1.559    0.025   62.614    0.000    1.510    1.608    1.559    1.559
    INTEREST_Y2|t1    0.030    0.015    1.948    0.051   -0.000    0.060    0.030    0.030
    INTEREST_Y2|t2    0.792    0.018   44.786    0.000    0.757    0.826    0.792    0.792
    INTEREST_Y2|t3    1.486    0.020   73.860    0.000    1.447    1.525    1.486    1.486
    CONTROL_WORRY_    0.339    0.014   23.452    0.000    0.310    0.367    0.339    0.339
    CONTROL_WORRY_    1.143    0.017   66.879    0.000    1.110    1.177    1.143    1.143
    CONTROL_WORRY_    1.666    0.022   74.844    0.000    1.622    1.709    1.666    1.666
    FEEL_ANXIOUS_Y   -0.058    0.015   -3.807    0.000   -0.087   -0.028   -
0.058   -0.058
    FEEL_ANXIOUS_Y    1.023    0.017   58.824    0.000    0.989    1.057    1.023    1.023
    FEEL_ANXIOUS_Y    1.531    0.024   63.768    0.000    1.484    1.578    1.531    1.531

Variances:
                   Estimate  Std.Err  z-value  P(>|z|) ci.lower ci.upper   Std.lv  Std.all
   .DEPRESSED_Y2      0.069                               0.069    0.069    0.069    0.069
   .INTEREST_Y2       0.541                               0.541    0.541    0.541    0.541
   .CONTROL_WORRY_    0.077                               0.077    0.077    0.077    0.077
   .FEEL_ANXIOUS_Y    0.146                               0.146    0.146    0.146    0.146
    dep               1.000                               1.000    1.000    1.000    1.000
    anx               1.000                               1.000    1.000    1.000    1.000
```

```
LIST.CFA.FIT[[cur.country]] <- fit
```

2.19.2.1 Residual correlations

Residual correlation greater than |0.05| are bolded.

```
tb_residual_cor(fit)
```

Variable	DEPRESSED_Y2	INTEREST_Y2	CONTROL_WORRY_Y2	FE
DEPRESSED_Y2	0.00			
INTEREST_Y2	0.00	0.00		
CONTROL_WORRY_Y2	-0.00	0.01	0.00	
FEEL_ANXIOUS_Y2	0.00	-0.01	0.00	

2.19.2.2 Modification indices

```
tb_mod_indices(fit, sort.=TRUE, maximum.number = 12)
```

lhs	op	rhs	mi	epc	sepc.lv
DEPRESSED_Y2	~~	FEEL_ANXIOUS_Y2	6.20	0.03	0.03
INTEREST_Y2	~~	FEEL_ANXIOUS_Y2	6.20	-0.02	-0.02
INTEREST_Y2	~~	CONTROL_WORRY_Y2	6.20	0.02	0.02
DEPRESSED_Y2	~~	CONTROL_WORRY_Y2	6.20	-0.03	-0.03

2.20 Tanzania

2.20.1 Sample statistics

The following sample statistics, computed using the survey design adjusted estimates from the survey package, are used are input for the factor analyses. This facilitates replication of all analyses without the need to get the raw data from the Center for Open Science.

```
ci <- ci + 1
cur.country <- names(sample.stats)[ci]
tb_sample_stats(sample.stats[[cur.country]])
```

Variable	DEPRESSED_Y2	INTEREST_Y2	CONTROL_WORRY_Y2	F
DEPRESSED_Y2	1.00	0.36	0.52	
INTEREST_Y2	0.36	1.00	0.34	
CONTROL_WORRY_Y2	0.52	0.34	1.00	
FEEL_ANXIOUS_Y2	0.55	0.36	0.64	
X				
Mean	1.20	1.17	0.85	
Standard.Deviation	1.00	1.00	1.00	
X.1				
Category	0.00	1.00	2.00	
DEPRESSED_Y2.1	38.93	16.96	29.04	
INTEREST_Y2.1	42.45	16.31	22.78	
CONTROL_WORRY_Y2.1	55.43	13.40	21.44	
FEEL_ANXIOUS_Y2.1	54.19	13.26	22.96	

2.20.2 Confirmatory factor analysis

```
fit <- cfa(
  mod, std.lv = TRUE, ordered=TRUE
  , sample.cov = sample.stats[[cur.country]]$sample.cov
  , sample.mean = sample.stats[[cur.country]]$sample.mean
```

```r
  , sample.nobs = sample.stats[[cur.country]]$sample.nobs
  , sample.th = sample.stats[[cur.country]]$sample.th
  , WLS.V = sample.stats[[cur.country]]$WLS.V
  , NACOV = sample.stats[[cur.country]]$NACOV
)

dynamic::cfaHB(fit)
```

```
Your DFI cutoffs:
            SRMR  RMSEA CFI   Magnitude
Level-0     0.007 0.04  0.999 NONE
Specificity 95%   95%   95%

Level-1     NONE  NONE  NONE  0.484
Sensitivity 1%    4%    0%

Empirical fit indices:
 Chi-Square  df p-value   SRMR   RMSEA    CFI
      0.087   1      NA  0.001       0      1
```

```r
summary(fit, standardized=TRUE, ci=TRUE, fit.measure=TRUE)
```

```
lavaan 0.6-21 ended normally after 14 iterations

  Estimator                                       DWLS
  Optimization method                           NLMINB
  Number of model parameters                        17

  Number of observations                      5583.00000008529

Model Test User Model:
                                              Standard      Scaled
  Test Statistic                                 0.087       0.074
  Degrees of freedom                                 1           1
  P-value (Unknown)                                 NA       0.785
  Scaling correction factor                                  1.172
  Shift parameter                                           -0.000
    simple second-order correction

Model Test Baseline Model:
```

```
  Test statistic                              9217.728    3281.746
  Degrees of freedom                                 6           6
  P-value                                           NA       0.000
  Scaling correction factor                                  2.810

User Model versus Baseline Model:

  Comparative Fit Index (CFI)                    1.000       1.000
  Tucker-Lewis Index (TLI)                       1.001       1.002

  Robust Comparative Fit Index (CFI)                         1.000
  Robust Tucker-Lewis Index (TLI)                            1.005

Root Mean Square Error of Approximation:

  RMSEA                                          0.000       0.000
  90 Percent confidence interval - lower         0.000       0.000
  90 Percent confidence interval - upper         0.024       0.023
  P-value H_0: RMSEA <= 0.050                    1.000       1.000
  P-value H_0: RMSEA >= 0.080                    0.000       0.000

  Robust RMSEA                                               0.000
  90 Percent confidence interval - lower                     0.000
  90 Percent confidence interval - upper                     0.055
  P-value H_0: Robust RMSEA <= 0.050                         0.935
  P-value H_0: Robust RMSEA >= 0.080                         0.010

Standardized Root Mean Square Residual:

  SRMR                                           0.001       0.001

Parameter Estimates:

  Parameterization                               Delta
  Standard errors                           Robust.sem
  Information                                 Expected
  Information saturated (h1) model        Unstructured

Latent Variables:
                   Estimate  Std.Err  z-value  P(>|z|) ci.lower ci.upper   Std.lv  Std.all
  dep =~
    DEPRESSED_Y2      0.737    0.020   36.085    0.000    0.697    0.777    0.737    0.737
    INTEREST_Y2       0.484    0.019   25.503    0.000    0.447    0.522    0.484    0.484
```

```
  anx =~
    CONTROL_WORRY_    0.779    0.017   47.006    0.000    0.747    0.812    0.779    0.779
    FEEL_ANXIOUS_Y    0.827    0.016   50.766    0.000    0.796    0.859    0.827    0.827

Covariances:
                   Estimate  Std.Err  z-value  P(>|z|) ci.lower ci.upper   Std.lv  Std.all
  dep ~~
    anx               0.900    0.027   33.854    0.000    0.848    0.952    0.900    0.900

Thresholds:
                   Estimate  Std.Err  z-value  P(>|z|) ci.lower ci.upper   Std.lv  Std.all
    DEPRESSED_Y2|1   -0.281    0.019  -14.772    0.000   -0.318   -0.244   -
0.281   -0.281
    DEPRESSED_Y2|2    0.148    0.019    7.704    0.000    0.111    0.186    0.148    0.148
    DEPRESSED_Y2|3    1.034    0.029   35.654    0.000    0.977    1.091    1.034    1.034
    INTEREST_Y2|t1   -0.190    0.025   -7.706    0.000   -0.239   -0.142   -
0.190   -0.190
    INTEREST_Y2|t2    0.221    0.021   10.309    0.000    0.179    0.263    0.221    0.221
    INTEREST_Y2|t3    0.898    0.023   38.659    0.000    0.852    0.944    0.898    0.898
    CONTROL_WORRY_    0.137    0.018    7.579    0.000    0.101    0.172    0.137    0.137
    CONTROL_WORRY_    0.491    0.019   25.692    0.000    0.453    0.528    0.491    0.491
    CONTROL_WORRY_    1.297    0.025   51.757    0.000    1.248    1.346    1.297    1.297
    FEEL_ANXIOUS_Y    0.105    0.026    4.129    0.000    0.055    0.155    0.105    0.105
    FEEL_ANXIOUS_Y    0.452    0.026   17.110    0.000    0.401    0.504    0.452    0.452
    FEEL_ANXIOUS_Y    1.305    0.031   42.156    0.000    1.245    1.366    1.305    1.305

Variances:
                   Estimate  Std.Err  z-value  P(>|z|) ci.lower ci.upper   Std.lv  Std.all
   .DEPRESSED_Y2      0.457                               0.457    0.457    0.457    0.457
   .INTEREST_Y2       0.765                               0.765    0.765    0.765    0.765
   .CONTROL_WORRY_    0.393                               0.393    0.393    0.393    0.393
   .FEEL_ANXIOUS_Y    0.315                               0.315    0.315    0.315    0.315
    dep               1.000                               1.000    1.000    1.000    1.000
    anx               1.000                               1.000    1.000    1.000    1.000
```

```r
LIST.CFA.FIT[[cur.country]] <- fit
```

2.20.2.1 Residual correlations

Residual correlation greater than |0.05| are bolded.

```r
tb_residual_cor(fit)
```

Variable	DEPRESSED_Y2	INTEREST_Y2	CONTROL_WORRY_Y2	FE
DEPRESSED_Y2	0.00			
INTEREST_Y2	0.00	0.00		
CONTROL_WORRY_Y2	0.00	-0.00	0.00	
FEEL_ANXIOUS_Y2	-0.00	0.00	-0.00	

2.20.2.2 Modification indices

```r
tb_mod_indices(fit, sort.=TRUE, maximum.number = 12)
```

lhs	op	rhs	mi	epc	sepc.lv
DEPRESSED_Y2	~~	CONTROL_WORRY_Y2	0.09	0.01	0.01
INTEREST_Y2	~~	CONTROL_WORRY_Y2	0.09	-0.01	-0.01
DEPRESSED_Y2	~~	FEEL_ANXIOUS_Y2	0.09	-0.01	-0.01
INTEREST_Y2	~~	FEEL_ANXIOUS_Y2	0.09	0.01	0.01

2.21 Turkey

2.21.1 Sample statistics

The following sample statistics, computed using the survey design adjusted estimates from the survey package, are used are input for the factor analyses. This facilitates replication of all analyses without the need to get the raw data from the Center for Open Science.

```
ci <- ci + 1
cur.country <- names(sample.stats)[ci]
tb_sample_stats(sample.stats[[cur.country]])
```

Variable	DEPRESSED_Y2	INTEREST_Y2	CONTROL_WORRY_Y2	F[illegible]
DEPRESSED_Y2	1.00	0.65	0.65	
INTEREST_Y2	0.65	1.00	0.56	
CONTROL_WORRY_Y2	0.65	0.56	1.00	
FEEL_ANXIOUS_Y2	0.68	0.62	0.70	
X				
Mean	1.32	1.28	0.94	
Standard.Deviation	1.00	1.00	1.00	
X.1				
Category	0.00	1.00	2.00	
DEPRESSED_Y2.1	33.29	23.16	22.41	
INTEREST_Y2.1	37.26	20.41	19.25	
CONTROL_WORRY_Y2.1	50.44	20.87	13.21	
FEEL_ANXIOUS_Y2.1	30.28	26.05	17.38	

2.21.2 Confirmatory factor analysis

```
fit <- cfa(
  mod, std.lv = TRUE, ordered=TRUE
  , sample.cov = sample.stats[[cur.country]]$sample.cov
  , sample.mean = sample.stats[[cur.country]]$sample.mean
```

```r
  , sample.nobs = sample.stats[[cur.country]]$sample.nobs
  , sample.th = sample.stats[[cur.country]]$sample.th
  , WLS.V = sample.stats[[cur.country]]$WLS.V
  , NACOV = sample.stats[[cur.country]]$NACOV
)

dynamic::cfaHB(fit)
```

```
Your DFI cutoffs:
            SRMR RMSEA CFI   Magnitude
Level-0     0.01 0.075 0.997 NONE
Specificity 95%  95%   95%

Level-1     NONE NONE  NONE  0.243
Sensitivity 1%   4%    0%

Empirical fit indices:
 Chi-Square  df p-value   SRMR   RMSEA    CFI
      0.216   1      NA  0.005       0      1
```

```r
summary(fit, standardized=TRUE, ci=TRUE, fit.measure=TRUE)
```

```
lavaan 0.6-21 ended normally after 13 iterations

  Estimator                                       DWLS
  Optimization method                           NLMINB
  Number of model parameters                        17

  Number of observations                      500.000000452455

Model Test User Model:
                                              Standard      Scaled
  Test Statistic                                 0.216       0.240
  Degrees of freedom                                 1           1
  P-value (Unknown)                                 NA       0.624
  Scaling correction factor                                  0.899
  Shift parameter                                            0.000
    simple second-order correction

Model Test Baseline Model:
```

```
  Test statistic                              2428.102     712.802
  Degrees of freedom                                 6           6
  P-value                                           NA       0.000
  Scaling correction factor                                  3.414

User Model versus Baseline Model:

  Comparative Fit Index (CFI)                    1.000       1.000
  Tucker-Lewis Index (TLI)                       1.002       1.006

  Robust Comparative Fit Index (CFI)                         1.000
  Robust Tucker-Lewis Index (TLI)                            1.022

Root Mean Square Error of Approximation:

  RMSEA                                          0.000       0.000
  90 Percent confidence interval - lower         0.000       0.000
  90 Percent confidence interval - upper         0.092       0.093
  P-value H_0: RMSEA <= 0.050                    0.800       0.789
  P-value H_0: RMSEA >= 0.080                    0.081       0.086

  Robust RMSEA                                               0.000
  90 Percent confidence interval - lower                     0.000
  90 Percent confidence interval - upper                     0.210
  P-value H_0: Robust RMSEA <= 0.050                         0.664
  P-value H_0: Robust RMSEA >= 0.080                         0.281

Standardized Root Mean Square Residual:

  SRMR                                           0.005       0.005

Parameter Estimates:

  Parameterization                               Delta
  Standard errors                           Robust.sem
  Information                                 Expected
  Information saturated (h1) model        Unstructured

Latent Variables:
                   Estimate  Std.Err  z-value  P(>|z|) ci.lower ci.upper   Std.lv  Std.all
  dep =~
    DEPRESSED_Y2      0.856    0.043   20.136    0.000    0.773    0.939    0.856    0.856
    INTEREST_Y2       0.758    0.042   17.863    0.000    0.675    0.841    0.758    0.758
```

```
  anx =~
    CONTROL_WORRY_    0.808    0.040   20.004    0.000    0.729    0.887    0.808    0.808
    FEEL_ANXIOUS_Y    0.867    0.041   20.916    0.000    0.786    0.948    0.867    0.867

Covariances:
                   Estimate  Std.Err  z-value  P(>|z|) ci.lower ci.upper   Std.lv  Std.all
  dep ~~
    anx               0.928    0.045   20.405    0.000    0.839    1.017    0.928    0.928

Thresholds:
                   Estimate  Std.Err  z-value  P(>|z|) ci.lower ci.upper   Std.lv  Std.all
    DEPRESSED_Y2|1   -0.432    0.069   -6.251    0.000   -0.567   -0.296   -
0.432   -0.432
    DEPRESSED_Y2|2    0.163    0.086    1.893    0.058   -0.006    0.331    0.163    0.163
    DEPRESSED_Y2|3    0.802    0.091    8.778    0.000    0.623    0.981    0.802    0.802
    INTEREST_Y2|t1   -0.325    0.070   -4.624    0.000   -0.463   -0.187   -
0.325   -0.325
    INTEREST_Y2|t2    0.193    0.074    2.615    0.009    0.048    0.338    0.193    0.193
    INTEREST_Y2|t3    0.736    0.086    8.599    0.000    0.568    0.904    0.736    0.736
    CONTROL_WORRY_    0.011    0.081    0.138    0.890   -0.147    0.169    0.011    0.011
    CONTROL_WORRY_    0.562    0.092    6.115    0.000    0.382    0.743    0.562    0.562
    CONTROL_WORRY_    1.016    0.093   10.925    0.000    0.834    1.198    1.016    1.016
    FEEL_ANXIOUS_Y   -0.517    0.092   -5.615    0.000   -0.697   -0.336   -
0.517   -0.517
    FEEL_ANXIOUS_Y    0.159    0.076    2.105    0.035    0.011    0.307    0.159    0.159
    FEEL_ANXIOUS_Y    0.634    0.098    6.459    0.000    0.442    0.827    0.634    0.634

Variances:
                   Estimate  Std.Err  z-value  P(>|z|) ci.lower ci.upper   Std.lv  Std.all
   .DEPRESSED_Y2      0.267                               0.267    0.267    0.267    0.267
   .INTEREST_Y2       0.425                               0.425    0.425    0.425    0.425
   .CONTROL_WORRY_    0.347                               0.347    0.347    0.347    0.347
   .FEEL_ANXIOUS_Y    0.248                               0.248    0.248    0.248    0.248
    dep               1.000                               1.000    1.000    1.000    1.000
    anx               1.000                               1.000    1.000    1.000    1.000
```

```
LIST.CFA.FIT[[cur.country]] <- fit
```

2.21.2.1 Residual correlations

Residual correlation greater than |0.05| are bolded.

```r
tb_residual_cor(fit)
```

Variable	DEPRESSED_Y2	INTEREST_Y2	CONTROL_WORRY_Y2	FE
DEPRESSED_Y2	0.00			
INTEREST_Y2	0.00	0.00		
CONTROL_WORRY_Y2	0.01	-0.01	0.00	
FEEL_ANXIOUS_Y2	-0.01	0.01	0.00	

2.21.2.2 Modification indices

```r
tb_mod_indices(fit, sort.=TRUE, maximum.number = 12)
```

lhs	op	rhs	mi	epc	sepc.lv
INTEREST_Y2	~~	FEEL_ANXIOUS_Y2	0.22	0.03	0.03
DEPRESSED_Y2	~~	FEEL_ANXIOUS_Y2	0.22	-0.03	-0.03
INTEREST_Y2	~~	CONTROL_WORRY_Y2	0.22	-0.03	-0.03
DEPRESSED_Y2	~~	CONTROL_WORRY_Y2	0.22	0.03	0.03

2.22 United Kingdom

2.22.1 Sample statistics

The following sample statistics, computed using the survey design adjusted estimates from the survey package, are used are input for the factor analyses. This facilitates replication of all analyses without the need to get the raw data from the Center for Open Science.

```
ci <- ci + 1
cur.country <- names(sample.stats)[ci]
tb_sample_stats(sample.stats[[cur.country]])
```

Variable	DEPRESSED_Y2	INTEREST_Y2	CONTROL_WORRY_Y2	F
DEPRESSED_Y2	1.00	0.83	0.81	
INTEREST_Y2	0.83	1.00	0.72	
CONTROL_WORRY_Y2	0.81	0.72	1.00	
FEEL_ANXIOUS_Y2	0.78	0.68	0.87	
X				
Mean	0.83	0.89	0.85	
Standard.Deviation	1.00	1.00	1.00	
X.1				
Category	0.00	1.00	2.00	
DEPRESSED_Y2.1	48.04	29.90	13.45	
INTEREST_Y2.1	45.44	29.33	16.36	
CONTROL_WORRY_Y2.1	48.27	28.99	12.86	
FEEL_ANXIOUS_Y2.1	42.88	32.83	14.32	

2.22.2 Confirmatory factor analysis

```
fit <- cfa(
  mod, std.lv = TRUE, ordered=TRUE
  , sample.cov = sample.stats[[cur.country]]$sample.cov
  , sample.mean = sample.stats[[cur.country]]$sample.mean
```

```r
  , sample.nobs = sample.stats[[cur.country]]$sample.nobs
  , sample.th = sample.stats[[cur.country]]$sample.th
  , WLS.V = sample.stats[[cur.country]]$WLS.V
  , NACOV = sample.stats[[cur.country]]$NACOV
)

dynamic::cfaHB(fit)
```

```
Your DFI cutoffs:
            SRMR  RMSEA CFI  Magnitude
Level-0     0.003 0.037 1    NONE
Specificity 95%   95%   95%

Level-1     NONE  NONE  NONE 0.154
Sensitivity 0%    6%    2%

Empirical fit indices:
 Chi-Square  df p-value   SRMR   RMSEA    CFI
      0.766   1      NA  0.003       0      1
```

```r
summary(fit, standardized=TRUE, ci=TRUE, fit.measure=TRUE)
```

```
lavaan 0.6-21 ended normally after 17 iterations

  Estimator                                         DWLS
  Optimization method                             NLMINB
  Number of model parameters                          17

  Number of observations                        3619.00000002024

Model Test User Model:
                                              Standard      Scaled
  Test Statistic                                 0.766       0.685
  Degrees of freedom                                 1           1
  P-value (Unknown)                                 NA       0.408
  Scaling correction factor                                  1.118
  Shift parameter                                            0.000
    simple second-order correction

Model Test Baseline Model:
```

```
  Test statistic                             57888.352    9720.348
  Degrees of freedom                                 6           6
  P-value                                           NA       0.000
  Scaling correction factor                                  5.957

User Model versus Baseline Model:

  Comparative Fit Index (CFI)                    1.000       1.000
  Tucker-Lewis Index (TLI)                       1.000       1.000

  Robust Comparative Fit Index (CFI)                         1.000
  Robust Tucker-Lewis Index (TLI)                            1.002

Root Mean Square Error of Approximation:

  RMSEA                                          0.000       0.000
  90 Percent confidence interval - lower         0.000       0.000
  90 Percent confidence interval - upper         0.042       0.041
  P-value H_0: RMSEA <= 0.050                    0.984       0.985
  P-value H_0: RMSEA >= 0.080                    0.000       0.000

  Robust RMSEA                                               0.000
  90 Percent confidence interval - lower                     0.000
  90 Percent confidence interval - upper                     0.147
  P-value H_0: Robust RMSEA <= 0.050                         0.552
  P-value H_0: Robust RMSEA >= 0.080                         0.289

Standardized Root Mean Square Residual:

  SRMR                                           0.003       0.003

Parameter Estimates:

  Parameterization                               Delta
  Standard errors                           Robust.sem
  Information                                 Expected
  Information saturated (h1) model        Unstructured

Latent Variables:
                   Estimate  Std.Err  z-value  P(>|z|) ci.lower ci.upper   Std.lv  Std.all
  dep =~
    DEPRESSED_Y2      0.970    0.008  116.311    0.000    0.954    0.987    0.970    0.970
    INTEREST_Y2       0.854    0.014   63.000    0.000    0.828    0.881    0.854    0.854
```

```
  anx =~
    CONTROL_WORRY_    0.948    0.008  124.018    0.000    0.933    0.963    0.948    0.948
    FEEL_ANXIOUS_Y    0.913    0.010   95.893    0.000    0.894    0.931    0.913    0.913

Covariances:
                   Estimate  Std.Err  z-value  P(>|z|) ci.lower ci.upper   Std.lv  Std.all
  dep ~~
    anx               0.882    0.013   67.898    0.000    0.857    0.908    0.882    0.882

Thresholds:
                   Estimate  Std.Err  z-value  P(>|z|) ci.lower ci.upper   Std.lv  Std.all
    DEPRESSED_Y2|1   -0.049    0.032   -1.518    0.129   -0.112    0.014   -
0.049   -0.049
    DEPRESSED_Y2|2    0.770    0.033   23.489    0.000    0.706    0.835    0.770    0.770
    DEPRESSED_Y2|3    1.366    0.047   28.800    0.000    1.273    1.459    1.366    1.366
    INTEREST_Y2|t1   -0.115    0.031   -3.709    0.000   -0.175   -0.054   -
0.115   -0.115
    INTEREST_Y2|t2    0.667    0.032   21.122    0.000    0.605    0.729    0.667    0.667
    INTEREST_Y2|t3    1.348    0.039   34.179    0.000    1.271    1.426    1.348    1.348
    CONTROL_WORRY_   -0.043    0.032   -1.371    0.170   -0.105    0.019   -
0.043   -0.043
    CONTROL_WORRY_    0.748    0.039   19.382    0.000    0.672    0.823    0.748    0.748
    CONTROL_WORRY_    1.288    0.043   29.976    0.000    1.204    1.373    1.288    1.288
    FEEL_ANXIOUS_Y   -0.180    0.032   -5.679    0.000   -0.241   -0.118   -
0.180   -0.180
    FEEL_ANXIOUS_Y    0.697    0.034   20.390    0.000    0.630    0.764    0.697    0.697
    FEEL_ANXIOUS_Y    1.283    0.040   32.087    0.000    1.205    1.362    1.283    1.283

Variances:
                   Estimate  Std.Err  z-value  P(>|z|) ci.lower ci.upper   Std.lv  Std.all
   .DEPRESSED_Y2      0.059                               0.059    0.059    0.059    0.059
   .INTEREST_Y2       0.270                               0.270    0.270    0.270    0.270
   .CONTROL_WORRY_    0.101                               0.101    0.101    0.101    0.101
   .FEEL_ANXIOUS_Y    0.167                               0.167    0.167    0.167    0.167
    dep               1.000                               1.000    1.000    1.000    1.000
    anx               1.000                               1.000    1.000    1.000    1.000
```

```
LIST.CFA.FIT[[cur.country]] <- fit
```

2.22.2.1 Residual correlations

Residual correlation greater than |0.05| are bolded.

```r
tb_residual_cor(fit)
```

Variable	DEPRESSED_Y2	INTEREST_Y2	CONTROL_WORRY_Y2	FE
DEPRESSED_Y2	0.00			
INTEREST_Y2	0.00	0.00		
CONTROL_WORRY_Y2	-0.00	0.00	0.00	
FEEL_ANXIOUS_Y2	0.00	-0.01	-0.00	

2.22.2.2 Modification indices

```r
tb_mod_indices(fit, sort.=TRUE, maximum.number = 12)
```

lhs	op	rhs	mi	epc	sepc.lv
DEPRESSED_Y2	~~	CONTROL_WORRY_Y2	0.77	-0.02	-0.02
INTEREST_Y2	~~	FEEL_ANXIOUS_Y2	0.77	-0.01	-0.01
INTEREST_Y2	~~	CONTROL_WORRY_Y2	0.77	0.01	0.01
DEPRESSED_Y2	~~	FEEL_ANXIOUS_Y2	0.77	0.02	0.02

2.23 United States

2.23.1 Sample statistics

The following sample statistics, computed using the survey design adjusted estimates from the survey package, are used are input for the factor analyses. This facilitates replication of all analyses without the need to get the raw data from the Center for Open Science.

```
ci <- ci + 1
cur.country <- names(sample.stats)[ci]
tb_sample_stats(sample.stats[[cur.country]])
```

Variable	DEPRESSED_Y2	INTEREST_Y2	CONTROL_WORRY_Y2	F
DEPRESSED_Y2	1.00	0.84	0.77	
INTEREST_Y2	0.84	1.00	0.68	
CONTROL_WORRY_Y2	0.77	0.68	1.00	
FEEL_ANXIOUS_Y2	0.75	0.66	0.85	
X				
Mean	0.61	0.67	0.69	
Standard.Deviation	1.00	1.00	1.00	
X.1				
Category	0.00	1.00	2.00	
DEPRESSED_Y2.1	56.84	29.86	8.58	
INTEREST_Y2.1	53.56	31.08	10.57	
CONTROL_WORRY_Y2.1	53.74	30.03	9.33	
FEEL_ANXIOUS_Y2.1	44.09	37.30	11.25	

2.23.2 Confirmatory factor analysis

```
fit <- cfa(
  mod, std.lv = TRUE, ordered=TRUE
  , sample.cov = sample.stats[[cur.country]]$sample.cov
  , sample.mean = sample.stats[[cur.country]]$sample.mean
```

```r
  , sample.nobs = sample.stats[[cur.country]]$sample.nobs
  , sample.th = sample.stats[[cur.country]]$sample.th
  , WLS.V = sample.stats[[cur.country]]$WLS.V
  , NACOV = sample.stats[[cur.country]]$NACOV
)

dynamic::cfaHB(fit)
```

```
Your DFI cutoffs:
            SRMR  RMSEA CFI  Magnitude
Level-0     0.003 0.038 1    NONE
Specificity 95%   95%   95%

Level-1     NONE  NONE  NONE 0.153
Sensitivity 1%    5%    1%

Empirical fit indices:
 Chi-Square  df p-value   SRMR   RMSEA    CFI
      0.107   1      NA      0       0      1
```

```r
summary(fit, standardized=TRUE, ci=TRUE, fit.measure=TRUE)
```

```
lavaan 0.6-21 ended normally after 15 iterations

  Estimator                                       DWLS
  Optimization method                           NLMINB
  Number of model parameters                        17

  Number of observations                      32245.0003114482

Model Test User Model:
                                              Standard      Scaled
  Test Statistic                                 0.107       0.150
  Degrees of freedom                                 1           1
  P-value (Unknown)                                 NA       0.699
  Scaling correction factor                                  0.710
  Shift parameter                                           -0.000
    simple second-order correction

Model Test Baseline Model:
```

```
  Test statistic                              325492.972   54618.447
  Degrees of freedom                                   6           6
  P-value                                             NA       0.000
  Scaling correction factor                                    5.960

User Model versus Baseline Model:

  Comparative Fit Index (CFI)                      1.000       1.000
  Tucker-Lewis Index (TLI)                         1.000       1.000

  Robust Comparative Fit Index (CFI)                           1.000
  Robust Tucker-Lewis Index (TLI)                              1.001

Root Mean Square Error of Approximation:

  RMSEA                                            0.000       0.000
  90 Percent confidence interval - lower           0.000       0.000
  90 Percent confidence interval - upper           0.010       0.011
  P-value H_0: RMSEA <= 0.050                      1.000       1.000
  P-value H_0: RMSEA >= 0.080                      0.000       0.000

  Robust RMSEA                                                 0.000
  90 Percent confidence interval - lower                       0.000
  90 Percent confidence interval - upper                       0.037
  P-value H_0: Robust RMSEA <= 0.050                           0.989
  P-value H_0: Robust RMSEA >= 0.080                           0.000

Standardized Root Mean Square Residual:

  SRMR                                             0.000       0.000

Parameter Estimates:

  Parameterization                                 Delta
  Standard errors                             Robust.sem
  Information                                   Expected
  Information saturated (h1) model          Unstructured

Latent Variables:
                   Estimate  Std.Err  z-value  P(>|z|) ci.lower ci.upper   Std.lv  Std.all
  dep =~
    DEPRESSED_Y2      0.976    0.004  276.098    0.000    0.969    0.983    0.976    0.976
    INTEREST_Y2       0.861    0.005  173.645    0.000    0.851    0.871    0.861    0.861
```

```
  anx =~
    CONTROL_WORRY_    0.934    0.004  243.128    0.000    0.927    0.942    0.934    0.934
    FEEL_ANXIOUS_Y    0.912    0.005  202.287    0.000    0.903    0.921    0.912    0.912

Covariances:
                   Estimate  Std.Err  z-value  P(>|z|) ci.lower ci.upper   Std.lv  Std.all
  dep ~~
    anx               0.841    0.006  144.876    0.000    0.829    0.852    0.841    0.841

Thresholds:
                   Estimate  Std.Err  z-value  P(>|z|) ci.lower ci.upper   Std.lv  Std.all
    DEPRESSED_Y2|1    0.172    0.013   12.986    0.000    0.146    0.198    0.172    0.172
    DEPRESSED_Y2|2    1.112    0.016   69.382    0.000    1.081    1.144    1.112    1.112
    DEPRESSED_Y2|3    1.673    0.028   60.823    0.000    1.619    1.727    1.673    1.673
    INTEREST_Y2|t1    0.089    0.011    8.170    0.000    0.068    0.111    0.089    0.089
    INTEREST_Y2|t2    1.021    0.016   64.749    0.000    0.990    1.052    1.021    1.021
    INTEREST_Y2|t3    1.665    0.022   74.045    0.000    1.621    1.709    1.665    1.665
    CONTROL_WORRY_    0.094    0.013    7.344    0.000    0.069    0.119    0.094    0.094
    CONTROL_WORRY_    0.985    0.016   61.917    0.000    0.954    1.016    0.985    0.985
    CONTROL_WORRY_    1.483    0.020   74.159    0.000    1.444    1.522    1.483    1.483
    FEEL_ANXIOUS_Y   -0.149    0.011  -13.111    0.000   -0.171   -0.126   -
0.149   -0.149
    FEEL_ANXIOUS_Y    0.892    0.015   59.970    0.000    0.863    0.922    0.892    0.892
    FEEL_ANXIOUS_Y    1.449    0.020   73.237    0.000    1.411    1.488    1.449    1.449

Variances:
                   Estimate  Std.Err  z-value  P(>|z|) ci.lower ci.upper   Std.lv  Std.all
   .DEPRESSED_Y2      0.047                               0.047    0.047    0.047    0.047
   .INTEREST_Y2       0.259                               0.259    0.259    0.259    0.259
   .CONTROL_WORRY_    0.127                               0.127    0.127    0.127    0.127
   .FEEL_ANXIOUS_Y    0.168                               0.168    0.168    0.168    0.168
    dep               1.000                               1.000    1.000    1.000    1.000
    anx               1.000                               1.000    1.000    1.000    1.000
```

```
LIST.CFA.FIT[[cur.country]] <- fit
```

2.23.2.1 Residual correlations

Residual correlation greater than |0.05| are bolded.

```
tb_residual_cor(fit)
```

Variable	DEPRESSED_Y2	INTEREST_Y2	CONTROL_WORRY_Y2	FE
DEPRESSED_Y2	0.00			
INTEREST_Y2	0.00	0.00		
CONTROL_WORRY_Y2	-0.00	0.00	0.00	
FEEL_ANXIOUS_Y2	0.00	-0.00	0.00	

2.23.2.2 Modification indices

```
tb_mod_indices(fit, sort.=TRUE, maximum.number = 12)
```

lhs	op	rhs	mi	epc	sepc.lv
DEPRESSED_Y2	~~	CONTROL_WORRY_Y2	0.11	-0.00	-0.00
DEPRESSED_Y2	~~	FEEL_ANXIOUS_Y2	0.11	0.00	0.00
INTEREST_Y2	~~	CONTROL_WORRY_Y2	0.11	0.00	0.00
INTEREST_Y2	~~	FEEL_ANXIOUS_Y2	0.11	-0.00	-0.00

3 Fit summary

Summary of fitted models ordered by RMSEA.

```
## samples sizes
fit.n <- lapply(sample.stats, \(x) x$ng) |> bind_rows() |> t() |> as.data.frame()
colnames(fit.n) <- "N"
fit.n$N <- .round(fit.n$N,0)
fit.n <- fit.n |> rownames_to_column("Country")

fit.list <- lapply(LIST.CFA.FIT, function(x){
  fitMeasures(
    object = x,
    fit.measures = c("chisq.scaled", "df.scaled", "pvalue.scaled", "rmsea.scaled", "rmsea.ci
    baseline.model = NULL
    )
})

fit.tb <- fit.list |> bind_rows()
rownames(fit.tb) <- names(fit.list)
fit.tb <- fit.tb |> rownames_to_column("Country")
fit.tb <- left_join(fit.n, fit.tb)
fit.tb |>
  arrange(rmsea.scaled) |>
  flextable()  |>
  colformat_double(digits = 3)
```

Country	N	chisq.scaled	df.scaled	pvalue.scaled	rmsea.scaled	rmsea.ci.lower.scaled	rmsea.ci.upper.scaled	cfi.scaled
Egypt	3040	0.095	1.000	0.758	0.000	0.000	0.033	1.000
Germany	5529	0.093	1.000	0.761	0.000	0.000	0.024	1.000
Hong Kong	707	0.001	1.000	0.979	0.000	0.000	0.000	1.000
Japan	13972	0.000	1.000	0.987	0.000	0.000	0.000	1.000
Kenya	7698	0.222	1.000	0.637	0.000	0.000	0.024	1.000
Poland	6478	0.396	1.000	0.529	0.000	0.000	0.028	1.000

Country	N	chisq.scaled	df.scaled	pvalue.scaled	rmsea.scaled	rmsea.ci.lower.scaled	rmsea.ci.upper.scaled	cfi.scaled
South Africa	978	0.002	1.000	0.961	0.000	0.000	0.000	1.000
Tanzania	5583	0.074	1.000	0.785	0.000	0.000	0.023	1.000
Turkey	500	0.240	1.000	0.624	0.000	0.000	0.093	1.000
United Kingdom	3619	0.685	1.000	0.408	0.000	0.000	0.041	1.000
United States	32245	0.150	1.000	0.699	0.000	0.000	0.011	1.000
Argentina	2932	1.448	1.000	0.229	0.012	0.000	0.053	1.000
Australia	2582	1.461	1.000	0.227	0.013	0.000	0.056	1.000
Nigeria	3146	2.086	1.000	0.149	0.019	0.000	0.055	0.999
Philippines	2682	2.349	1.000	0.125	0.022	0.000	0.061	0.999
Mexico	2278	2.206	1.000	0.137	0.023	0.000	0.066	1.000
Sweden	11609	7.397	1.000	0.007	0.023	0.010	0.041	1.000
Indonesia	2684	2.685	1.000	0.101	0.025	0.000	0.063	1.000
Spain	2924	3.249	1.000	0.071	0.028	0.000	0.064	0.999
Brazil	4274	5.199	1.000	0.023	0.031	0.009	0.060	1.000
China	4544	10.744	1.000	0.001	0.046	0.024	0.073	0.999
India	6374	17.938	1.000	0.000	0.052	0.032	0.074	0.997
Israel	2490	9.240	1.000	0.002	0.058	0.028	0.094	0.997

3.1 Composite reliability and AVE estimates

```
fit.rel <- lapply(LIST.CFA.FIT, function(x){
  y=semTools::compRelSEM(x, return.total = TRUE)
  data.frame(DEP = y[1], ANX=y[2], total=y[3])
}) |> bind_rows(.id="Country")

flextable(fit.rel) |>
```

```
colformat_double(digits = 3) |>
add_header_lines("Composite reliability estimates (coefficient omega).") |>
autofit()
```

Composite reliability estimates (coefficient omega).

Country	DEP	ANX	total
Argentina	0.802	0.801	0.881
Australia	0.861	0.863	0.917
Brazil	0.759	0.767	0.859
China	0.623	0.797	0.825
Egypt	0.611	0.767	0.810
Germany	0.823	0.730	0.868
Hong Kong	0.687	0.853	0.868
India	0.526	0.680	0.743
Indonesia	0.607	0.781	0.820
Israel	0.802	0.841	0.900
Japan	0.869	0.863	0.926
Kenya	0.581	0.656	0.760
Mexico	0.812	0.796	0.886
Nigeria	0.572	0.596	0.731
Philippines	0.544	0.702	0.765
Poland	0.754	0.767	0.858
South Africa	0.563	0.579	0.722
Spain	0.770	0.767	0.867
Sweden	0.738	0.883	0.887
Tanzania	0.477	0.700	0.728
Turkey	0.727	0.759	0.847
United Kingdom	0.860	0.880	0.926
United States	0.858	0.863	0.918

```
fit.ave <- lapply(LIST.CFA.FIT, function(x){
  y=semTools::AVE(x)
  data.frame(DEP = y[1], ANX=y[2])
}) |> bind_rows(.id="Country")
flextable(fit.ave) |>
  colformat_double(digits = 3) |>
  add_header_lines("Average variance explained.") |>
  autofit()
```

Average variance explained.		
Country	DEP	ANX
Argentina	0.749	0.752
Australia	0.822	0.848
Brazil	0.696	0.705
China	0.571	0.783
Egypt	0.509	0.708
Germany	0.805	0.672
Hong Kong	0.639	0.853
India	0.450	0.600
Indonesia	0.546	0.750
Israel	0.767	0.830
Japan	0.854	0.856
Kenya	0.482	0.570
Mexico	0.767	0.752
Nigeria	0.459	0.489
Philippines	0.435	0.626
Poland	0.739	0.758
South Africa	0.455	0.474
Spain	0.707	0.706
Sweden	0.695	0.888
Tanzania	0.389	0.646

Average variance explained.		
Country	DEP	ANX
Turkey	0.654	0.702
United Kingdom	0.836	0.866
United States	0.847	0.852

3.2 Coefficient alpha

```
## USE psych package
## Coefficient Alpha of the 12-item composite
COUNTRIES <- sort(as.character(unique(df.cc$COUNTRY)))
names(COUNTRIES) <- COUNTRIES
x=COUNTRIES[1]
list.alpha <- map(COUNTRIES, \(x){

  cat("## ============================================================================ ##\n"
  cat(paste0("## Country: ", x, "\n\n"))

  tmp.dat = df.cc %>%
    filter(COUNTRY == x)
  svy.df <- svydesign(
    data = tmp.dat,
    ids = ~PSU,
    strata = ~STRATA,
    weights = ~AVG.SAMP.ATTR.WGT
  )

  fit.alpha <- svycralpha(reformulate(PHQ4.items), design = svy.df, na.rm=TRUE)
  cat(paste0("## Coefficient alpha (survey weighted): ", .round(fit.alpha,3)), "\n\n")
  suppressMessages({psych.alpha <- psych::alpha(tmp.dat[,PHQ4.items], warnings = FALSE)})
  cat("## Coefficeint alpha without survey adjustments:\n")
  print(psych.alpha)

  data.frame(svyalpha = fit.alpha, unadj.alpha=psych.alpha$total[1], unadj.alpha.lb = psych.a
})
```

```
## ============================================================================ ##
## Country: Argentina
```

```
## Coefficient alpha (survey weighted): 0.852

## Coefficeint alpha without survey adjustments:

Reliability analysis
Call: psych::alpha(x = tmp.dat[, PHQ4.items], warnings = FALSE)

  raw_alpha std.alpha G6(smc) average_r S/N    ase mean   sd median_r
      0.86      0.86    0.83       0.6   6 0.0029 0.94 0.83     0.61

    95% confidence boundaries
         lower alpha upper
Feldt     0.85  0.86  0.86
Duhachek  0.85  0.86  0.86

 Reliability if an item is dropped:
                 raw_alpha std.alpha G6(smc) average_r S/N alpha se  var.r med.r
DEPRESSED_Y2          0.80      0.80    0.74      0.57 4.0   0.0043 0.0083  0.53
INTEREST_Y2           0.84      0.84    0.78      0.63 5.2   0.0034 0.0013  0.62
CONTROL_WORRY_Y2      0.83      0.83    0.77      0.61 4.7   0.0037 0.0053  0.62
FEEL_ANXIOUS_Y2       0.81      0.81    0.76      0.59 4.4   0.0040 0.0080  0.60

 Item statistics
                    n raw.r std.r r.cor r.drop mean   sd
DEPRESSED_Y2     2918  0.86  0.87  0.81   0.75 0.82 0.97
INTEREST_Y2      2911  0.81  0.81  0.72   0.65 1.01 0.97
CONTROL_WORRY_Y2 2919  0.83  0.83  0.75   0.69 0.93 1.04
FEEL_ANXIOUS_Y2  2917  0.85  0.85  0.77   0.72 1.02 1.01

Non missing response frequency for each item
                    0    1    2    3 miss
DEPRESSED_Y2     0.48 0.31 0.11 0.09 0.57
INTEREST_Y2      0.36 0.39 0.14 0.11 0.57
CONTROL_WORRY_Y2 0.44 0.31 0.12 0.13 0.57
FEEL_ANXIOUS_Y2  0.37 0.37 0.13 0.13 0.57
## ========================================================================== ##
## Country: Australia

## Coefficient alpha (survey weighted): 0.874

## Coefficeint alpha without survey adjustments:
```

```
Reliability analysis
Call: psych::alpha(x = tmp.dat[, PHQ4.items], warnings = FALSE)

  raw_alpha std.alpha G6(smc) average_r S/N    ase mean   sd median_r
      0.87      0.87    0.86      0.63 6.8 0.0034 0.67 0.72     0.63

    95% confidence boundaries
         lower alpha upper
Feldt     0.87  0.87  0.88
Duhachek  0.87  0.87  0.88

 Reliability if an item is dropped:
                 raw_alpha std.alpha G6(smc) average_r S/N alpha se var.r med.r
DEPRESSED_Y2          0.82      0.82    0.77      0.60 4.4   0.0052 0.016  0.52
INTEREST_Y2           0.86      0.86    0.81      0.67 6.1   0.0039 0.004  0.64
CONTROL_WORRY_Y2      0.83      0.83    0.79      0.62 5.0   0.0048 0.011  0.63
FEEL_ANXIOUS_Y2       0.84      0.84    0.79      0.63 5.1   0.0047 0.010  0.64

 Item statistics
                    n raw.r std.r r.cor r.drop mean   sd
DEPRESSED_Y2     2578  0.88  0.88  0.84   0.78 0.62 0.83
INTEREST_Y2      2573  0.81  0.81  0.73   0.67 0.66 0.85
CONTROL_WORRY_Y2 2577  0.86  0.86  0.80   0.74 0.66 0.86
FEEL_ANXIOUS_Y2  2577  0.85  0.85  0.79   0.73 0.76 0.86

Non missing response frequency for each item
                    0    1    2    3 miss
DEPRESSED_Y2     0.56 0.31 0.08 0.05 0.33
INTEREST_Y2      0.53 0.32 0.10 0.05 0.33
CONTROL_WORRY_Y2 0.54 0.32 0.09 0.06 0.33
FEEL_ANXIOUS_Y2  0.46 0.39 0.09 0.06 0.33
## ============================================================================ ##
## Country: Brazil

## Coefficient alpha (survey weighted): 0.841

## Coefficeint alpha without survey adjustments:

Reliability analysis
Call: psych::alpha(x = tmp.dat[, PHQ4.items], warnings = FALSE)

  raw_alpha std.alpha G6(smc) average_r S/N    ase mean  sd median_r
      0.85      0.85    0.82      0.59 5.8 0.0021  1.2 0.9     0.61
```

```
    95% confidence boundaries
         lower alpha upper
Feldt     0.85  0.85  0.86
Duhachek  0.85  0.85  0.86

 Reliability if an item is dropped:
                 raw_alpha std.alpha G6(smc) average_r S/N alpha se   var.r med.r
DEPRESSED_Y2          0.80      0.80    0.73      0.57 3.9   0.0031 0.00600  0.53
INTEREST_Y2           0.84      0.84    0.78      0.63 5.1   0.0025 0.00095  0.64
CONTROL_WORRY_Y2      0.82      0.82    0.75      0.60 4.4   0.0028 0.00362  0.62
FEEL_ANXIOUS_Y2       0.80      0.80    0.74      0.58 4.1   0.0030 0.00299  0.60

 Item statistics
                    n raw.r std.r r.cor r.drop mean  sd
DEPRESSED_Y2     4269  0.85  0.86  0.80   0.73 0.94 1.0
INTEREST_Y2      4257  0.80  0.80  0.69   0.64 1.18 1.1
CONTROL_WORRY_Y2 4259  0.84  0.83  0.75   0.69 1.22 1.1
FEEL_ANXIOUS_Y2  4255  0.85  0.85  0.78   0.72 1.30 1.1

Non missing response frequency for each item
                    0    1    2    3 miss
DEPRESSED_Y2     0.44 0.30 0.14 0.12 0.68
INTEREST_Y2      0.33 0.31 0.19 0.16 0.68
CONTROL_WORRY_Y2 0.34 0.29 0.16 0.20 0.68
FEEL_ANXIOUS_Y2  0.28 0.34 0.16 0.22 0.68
## ============================================================================ ##
## Country: China

## Coefficient alpha (survey weighted): 0.811

## Coefficeint alpha without survey adjustments:

Reliability analysis
Call: psych::alpha(x = tmp.dat[, PHQ4.items], warnings = FALSE)

  raw_alpha std.alpha G6(smc) average_r S/N    ase mean  sd median_r
      0.81      0.82    0.78      0.53 4.5 0.0044 0.78 0.6     0.53

    95% confidence boundaries
         lower alpha upper
Feldt     0.81  0.81  0.82
Duhachek  0.81  0.81  0.82
```

```
 Reliability if an item is dropped:
                 raw_alpha std.alpha G6(smc) average_r S/N alpha se   var.r med.r
DEPRESSED_Y2          0.75      0.75    0.69      0.50 3.0   0.0063 0.01934  0.44
INTEREST_Y2           0.84      0.84    0.77      0.63 5.1   0.0040 0.00082  0.61
CONTROL_WORRY_Y2      0.74      0.74    0.67      0.49 2.9   0.0065 0.01170  0.45
FEEL_ANXIOUS_Y2       0.74      0.75    0.68      0.50 3.0   0.0063 0.00930  0.45

 Item statistics
                    n raw.r std.r r.cor r.drop mean   sd
DEPRESSED_Y2     4541  0.82  0.83  0.76   0.68 0.73 0.71
INTEREST_Y2      4544  0.73  0.71  0.54   0.50 1.00 0.82
CONTROL_WORRY_Y2 4543  0.84  0.84  0.78   0.69 0.62 0.76
FEEL_ANXIOUS_Y2  4541  0.82  0.83  0.77   0.68 0.78 0.71

Non missing response frequency for each item
                    0    1    2    3 miss
DEPRESSED_Y2     0.40 0.48 0.10 0.02  0.1
INTEREST_Y2      0.29 0.45 0.22 0.04  0.1
CONTROL_WORRY_Y2 0.53 0.35 0.10 0.02  0.1
FEEL_ANXIOUS_Y2  0.36 0.51 0.10 0.02  0.1
## ============================================================================ ##
## Country: Egypt

## Coefficient alpha (survey weighted): 0.777

## Coefficeint alpha without survey adjustments:

Reliability analysis
Call: psych::alpha(x = tmp.dat[, PHQ4.items], warnings = FALSE)

  raw_alpha std.alpha G6(smc) average_r S/N    ase mean   sd median_r
      0.77      0.76    0.72      0.45 3.2 0.0055  1.2 0.81     0.45

    95% confidence boundaries
         lower alpha upper
Feldt     0.75  0.77  0.78
Duhachek  0.75  0.77  0.78

 Reliability if an item is dropped:
                 raw_alpha std.alpha G6(smc) average_r S/N alpha se  var.r med.r
DEPRESSED_Y2          0.69      0.69    0.61      0.42 2.2   0.0078 0.0195  0.34
INTEREST_Y2           0.77      0.77    0.70      0.53 3.4   0.0057 0.0021  0.51
```

```
CONTROL_WORRY_Y2      0.68      0.68    0.60      0.42 2.1   0.0080 0.0062  0.41
FEEL_ANXIOUS_Y2       0.69      0.69    0.60      0.42 2.2   0.0079 0.0074  0.41

 Item statistics
                    n raw.r std.r r.cor r.drop mean   sd
DEPRESSED_Y2     3039  0.79  0.79  0.69   0.60  1.0 1.05
INTEREST_Y2      3023  0.67  0.68  0.50   0.44  1.1 0.98
CONTROL_WORRY_Y2 3039  0.80  0.79  0.71   0.62  1.2 1.06
FEEL_ANXIOUS_Y2  3039  0.80  0.79  0.70   0.61  1.5 1.11

Non missing response frequency for each item
                    0    1    2    3 miss
DEPRESSED_Y2     0.38 0.35 0.12 0.15 0.36
INTEREST_Y2      0.32 0.41 0.13 0.13 0.36
CONTROL_WORRY_Y2 0.33 0.36 0.14 0.17 0.36
FEEL_ANXIOUS_Y2  0.20 0.38 0.14 0.28 0.36
## ============================================================================ ##
## Country: Germany

## Coefficient alpha (survey weighted): 0.849

## Coefficeint alpha without survey adjustments:

Reliability analysis
Call: psych::alpha(x = tmp.dat[, PHQ4.items], warnings = FALSE)

  raw_alpha std.alpha G6(smc) average_r S/N    ase mean   sd median_r
      0.86      0.86    0.83      0.61 6.3 0.0023  0.7 0.73     0.61

    95% confidence boundaries
         lower alpha upper
Feldt     0.86  0.86  0.87
Duhachek  0.86  0.86  0.87

 Reliability if an item is dropped:
                 raw_alpha std.alpha G6(smc) average_r S/N alpha se   var.r med.r
DEPRESSED_Y2          0.80      0.80    0.73      0.57 4.0   0.0036 0.00049  0.57
INTEREST_Y2           0.82      0.82    0.76      0.61 4.7   0.0031 0.00022  0.62
CONTROL_WORRY_Y2      0.84      0.84    0.78      0.63 5.1   0.0029 0.00759  0.62
FEEL_ANXIOUS_Y2       0.84      0.84    0.79      0.64 5.2   0.0029 0.00635  0.62

 Item statistics
                    n raw.r std.r r.cor r.drop mean   sd
```

```
DEPRESSED_Y2     5512  0.88  0.88  0.84   0.77 0.67 0.85
INTEREST_Y2      5517  0.84  0.84  0.78   0.71 0.60 0.84
CONTROL_WORRY_Y2 5517  0.83  0.83  0.73   0.68 0.76 0.92
FEEL_ANXIOUS_Y2  5506  0.82  0.82  0.72   0.68 0.78 0.85

Non missing response frequency for each item
                    0    1    2    3 miss
DEPRESSED_Y2     0.53 0.32 0.09 0.05 0.42
INTEREST_Y2      0.58 0.28 0.09 0.05 0.42
CONTROL_WORRY_Y2 0.50 0.31 0.11 0.07 0.42
FEEL_ANXIOUS_Y2  0.44 0.39 0.11 0.05 0.42
## ============================================================================ ##
## Country: Hong Kong

## Coefficient alpha (survey weighted): 0.853

## Coefficeint alpha without survey adjustments:

Reliability analysis
Call: psych::alpha(x = tmp.dat[, PHQ4.items], warnings = FALSE)

  raw_alpha std.alpha G6(smc) average_r S/N    ase mean   sd median_r
      0.85      0.85    0.83      0.58 5.6 0.0046    1 0.66     0.59

    95% confidence boundaries
         lower alpha upper
Feldt     0.84  0.85  0.86
Duhachek  0.84  0.85  0.86

 Reliability if an item is dropped:
                 raw_alpha std.alpha G6(smc) average_r S/N alpha se   var.r med.r
DEPRESSED_Y2          0.77      0.77    0.73      0.53 3.4   0.0074 0.03155  0.44
INTEREST_Y2           0.89      0.89    0.84      0.72 7.7   0.0036 0.00043  0.72
CONTROL_WORRY_Y2      0.77      0.78    0.72      0.54 3.5   0.0073 0.02083  0.49
FEEL_ANXIOUS_Y2       0.78      0.79    0.74      0.55 3.7   0.0070 0.02313  0.49

 Item statistics
                   n raw.r std.r r.cor r.drop mean   sd
DEPRESSED_Y2     704  0.87  0.88  0.83   0.77 0.94 0.77
INTEREST_Y2      707  0.71  0.71  0.53   0.50 1.23 0.82
CONTROL_WORRY_Y2 706  0.87  0.87  0.84   0.76 0.81 0.81
FEEL_ANXIOUS_Y2  706  0.86  0.86  0.81   0.74 1.01 0.79
```

```
Non missing response frequency for each item
                    0    1    2    3 miss
DEPRESSED_Y2     0.30 0.49 0.18 0.03 0.77
INTEREST_Y2      0.18 0.47 0.28 0.07 0.77
CONTROL_WORRY_Y2 0.41 0.40 0.17 0.03 0.77
FEEL_ANXIOUS_Y2  0.25 0.53 0.17 0.05 0.77
## ============================================================================ ##
## Country: India

## Coefficient alpha (survey weighted): 0.695

## Coefficeint alpha without survey adjustments:

Reliability analysis
Call: psych::alpha(x = tmp.dat[, PHQ4.items], warnings = FALSE)

  raw_alpha std.alpha G6(smc) average_r S/N    ase mean   sd median_r
       0.7       0.7    0.65      0.37 2.3 0.0044  1.2 0.81     0.37

    95% confidence boundaries
         lower alpha upper
Feldt     0.69   0.7  0.71
Duhachek  0.69   0.7  0.71

 Reliability if an item is dropped:
                 raw_alpha std.alpha G6(smc) average_r S/N alpha se  var.r med.r
DEPRESSED_Y2          0.59      0.59    0.51      0.32 1.4   0.0064 0.0203  0.26
INTEREST_Y2           0.73      0.73    0.65      0.48 2.8   0.0041 0.0014  0.49
CONTROL_WORRY_Y2      0.61      0.61    0.54      0.34 1.6   0.0061 0.0228  0.30
FEEL_ANXIOUS_Y2       0.59      0.60    0.51      0.33 1.5   0.0063 0.0093  0.30

 Item statistics
                    n raw.r std.r r.cor r.drop mean  sd
DEPRESSED_Y2     6357  0.77  0.77  0.67   0.56  1.1 1.1
INTEREST_Y2      6359  0.62  0.61  0.38   0.32  1.4 1.2
CONTROL_WORRY_Y2 6347  0.76  0.75  0.63   0.52  1.1 1.2
FEEL_ANXIOUS_Y2  6351  0.76  0.77  0.67   0.55  1.1 1.1

Non missing response frequency for each item
                    0    1    2    3 miss
DEPRESSED_Y2     0.38 0.24 0.23 0.14  0.5
INTEREST_Y2      0.30 0.22 0.22 0.26  0.5
CONTROL_WORRY_Y2 0.43 0.20 0.19 0.18  0.5
```

```
FEEL_ANXIOUS_Y2  0.43 0.22 0.21 0.14  0.5
## ============================================================================ ##
## Country: Indonesia

## Coefficient alpha (survey weighted): 0.799

## Coefficeint alpha without survey adjustments:

Reliability analysis
Call: psych::alpha(x = tmp.dat[, PHQ4.items], warnings = FALSE)

  raw_alpha std.alpha G6(smc) average_r S/N    ase mean   sd median_r
      0.82      0.82    0.78      0.53 4.5 0.0036 0.71 0.72     0.52

    95% confidence boundaries
         lower alpha upper
Feldt     0.81  0.82  0.82
Duhachek  0.81  0.82  0.82

 Reliability if an item is dropped:
                 raw_alpha std.alpha G6(smc) average_r S/N alpha se  var.r med.r
DEPRESSED_Y2          0.75      0.75    0.69      0.50 3.0   0.0053 0.0183  0.42
INTEREST_Y2           0.83      0.83    0.77      0.63 5.0   0.0034 0.0010  0.63
CONTROL_WORRY_Y2      0.75      0.75    0.68      0.50 3.0   0.0053 0.0135  0.44
FEEL_ANXIOUS_Y2       0.74      0.74    0.66      0.49 2.8   0.0055 0.0088  0.44

 Item statistics
                    n raw.r std.r r.cor r.drop mean   sd
DEPRESSED_Y2     2681  0.83  0.83  0.76   0.68 0.65 0.87
INTEREST_Y2      2662  0.71  0.71  0.54   0.49 0.89 0.90
CONTROL_WORRY_Y2 2678  0.83  0.83  0.76   0.68 0.59 0.89
FEEL_ANXIOUS_Y2  2677  0.84  0.84  0.79   0.70 0.73 0.90

Non missing response frequency for each item
                    0    1    2    3 miss
DEPRESSED_Y2     0.55 0.32 0.06 0.07 0.62
INTEREST_Y2      0.38 0.44 0.10 0.09 0.62
CONTROL_WORRY_Y2 0.61 0.26 0.06 0.07 0.62
FEEL_ANXIOUS_Y2  0.50 0.35 0.07 0.08 0.62
## ============================================================================ ##
## Country: Israel

## Coefficient alpha (survey weighted): 0.876
```

```
## Coefficeint alpha without survey adjustments:

Reliability analysis
Call: psych::alpha(x = tmp.dat[, PHQ4.items], warnings = FALSE)

  raw_alpha std.alpha G6(smc) average_r S/N    ase mean   sd median_r
      0.87      0.87    0.85      0.63 6.9 0.0035 0.56 0.68     0.65

    95% confidence boundaries
         lower alpha upper
Feldt     0.86  0.87  0.88
Duhachek  0.86  0.87  0.88

 Reliability if an item is dropped:
                 raw_alpha std.alpha G6(smc) average_r S/N alpha se  var.r med.r
DEPRESSED_Y2          0.81      0.81    0.76      0.59 4.3   0.0056 0.0135  0.60
INTEREST_Y2           0.87      0.87    0.82      0.69 6.7   0.0037 0.0016  0.70
CONTROL_WORRY_Y2      0.85      0.85    0.80      0.66 5.7   0.0043 0.0042  0.65
FEEL_ANXIOUS_Y2       0.81      0.81    0.76      0.59 4.3   0.0054 0.0103  0.65

 Item statistics
                    n raw.r std.r r.cor r.drop mean   sd
DEPRESSED_Y2     2471  0.89  0.89  0.84   0.79 0.60 0.82
INTEREST_Y2      2472  0.81  0.80  0.69   0.64 0.62 0.83
CONTROL_WORRY_Y2 2454  0.83  0.83  0.75   0.69 0.54 0.79
FEEL_ANXIOUS_Y2  2467  0.88  0.89  0.85   0.79 0.47 0.76

Non missing response frequency for each item
                    0    1    2    3 miss
DEPRESSED_Y2     0.58 0.29 0.09 0.04 0.33
INTEREST_Y2      0.57 0.28 0.11 0.04 0.33
CONTROL_WORRY_Y2 0.61 0.26 0.09 0.03 0.33
FEEL_ANXIOUS_Y2  0.66 0.24 0.07 0.03 0.33
## ============================================================================ ##
## Country: Japan

## Coefficient alpha (survey weighted): 0.910

## Coefficeint alpha without survey adjustments:

Reliability analysis
Call: psych::alpha(x = tmp.dat[, PHQ4.items], warnings = FALSE)
```

```
  raw_alpha std.alpha G6(smc) average_r S/N     ase mean   sd median_r
      0.91      0.91     0.9      0.73  11 0.00098 0.73 0.81     0.75

    95% confidence boundaries
         lower alpha upper
Feldt     0.91  0.91  0.92
Duhachek  0.91  0.91  0.92

 Reliability if an item is dropped:
                 raw_alpha std.alpha G6(smc) average_r  S/N alpha se   var.r med.r
DEPRESSED_Y2          0.87      0.87    0.82      0.68  6.5   0.0016 0.00482  0.68
INTEREST_Y2           0.91      0.91    0.87      0.77 10.2   0.0011 0.00078  0.76
CONTROL_WORRY_Y2      0.90      0.90    0.86      0.74  8.7   0.0013 0.00410  0.75
FEEL_ANXIOUS_Y2       0.88      0.88    0.84      0.71  7.3   0.0015 0.00626  0.75

 Item statistics
                     n raw.r std.r r.cor r.drop mean   sd
DEPRESSED_Y2     13900  0.93  0.93  0.91   0.87 0.72 0.91
INTEREST_Y2      13905  0.86  0.85  0.78   0.74 0.81 0.94
CONTROL_WORRY_Y2 13938  0.87  0.88  0.82   0.78 0.54 0.84
FEEL_ANXIOUS_Y2  13848  0.91  0.91  0.87   0.83 0.85 0.92

Non missing response frequency for each item
                    0    1    2    3 miss
DEPRESSED_Y2     0.53 0.29 0.11 0.07 0.32
INTEREST_Y2      0.48 0.29 0.16 0.07 0.32
CONTROL_WORRY_Y2 0.64 0.22 0.09 0.05 0.32
FEEL_ANXIOUS_Y2  0.43 0.36 0.13 0.08 0.33
## ========================================================================== ##
## Country: Kenya

## Coefficient alpha (survey weighted): 0.738

## Coefficeint alpha without survey adjustments:

Reliability analysis
Call: psych::alpha(x = tmp.dat[, PHQ4.items], warnings = FALSE)

  raw_alpha std.alpha G6(smc) average_r S/N   ase mean   sd median_r
      0.74      0.74    0.68      0.41 2.8 0.004  1.1 0.77     0.41

    95% confidence boundaries
```

```
         lower alpha upper
Feldt     0.73  0.74  0.74
Duhachek  0.73  0.74  0.74

 Reliability if an item is dropped:
                 raw_alpha std.alpha G6(smc) average_r S/N alpha se   var.r med.r
DEPRESSED_Y2          0.66      0.66    0.57      0.39 1.9   0.0055 0.00472  0.37
INTEREST_Y2           0.72      0.72    0.63      0.46 2.5   0.0046 0.00061  0.47
CONTROL_WORRY_Y2      0.67      0.67    0.58      0.41 2.1   0.0053 0.00319  0.39
FEEL_ANXIOUS_Y2       0.65      0.65    0.56      0.39 1.9   0.0056 0.00187  0.39

 Item statistics
                    n raw.r std.r r.cor r.drop mean  sd
DEPRESSED_Y2     7695  0.76  0.77  0.65   0.56  1.1 1.0
INTEREST_Y2      7693  0.70  0.70  0.53   0.46  1.2 1.0
CONTROL_WORRY_Y2 7696  0.76  0.75  0.62   0.53  1.1 1.1
FEEL_ANXIOUS_Y2  7696  0.77  0.77  0.66   0.57  1.0 1.0

Non missing response frequency for each item
                    0    1    2    3 miss
DEPRESSED_Y2     0.32 0.42 0.12 0.15 0.32
INTEREST_Y2      0.27 0.44 0.12 0.17 0.32
CONTROL_WORRY_Y2 0.35 0.36 0.12 0.17 0.32
FEEL_ANXIOUS_Y2  0.36 0.38 0.13 0.13 0.32
## ================================================================================ ##
## Country: Mexico

## Coefficient alpha (survey weighted): 0.859

## Coefficeint alpha without survey adjustments:

Reliability analysis
Call: psych::alpha(x = tmp.dat[, PHQ4.items], warnings = FALSE)

  raw_alpha std.alpha G6(smc) average_r S/N   ase mean   sd median_r
      0.86      0.86    0.83      0.61 6.4 0.003 0.88 0.82     0.62

    95% confidence boundaries
         lower alpha upper
Feldt     0.86  0.86  0.87
Duhachek  0.86  0.86  0.87

 Reliability if an item is dropped:
```

```
                 raw_alpha std.alpha G6(smc) average_r S/N alpha se  var.r med.r
DEPRESSED_Y2          0.80      0.80    0.74      0.58 4.1   0.0045 0.0031  0.57
INTEREST_Y2           0.84      0.85    0.79      0.65 5.5   0.0036 0.0021  0.64
CONTROL_WORRY_Y2      0.84      0.84    0.79      0.64 5.3   0.0037 0.0043  0.65
FEEL_ANXIOUS_Y2       0.81      0.82    0.75      0.60 4.4   0.0043 0.0036  0.60

 Item statistics
                    n raw.r std.r r.cor r.drop mean   sd
DEPRESSED_Y2     2272  0.87  0.87  0.83   0.76 0.82 0.96
INTEREST_Y2      2268  0.81  0.82  0.72   0.67 0.94 0.97
CONTROL_WORRY_Y2 2269  0.83  0.82  0.73   0.68 0.90 1.02
FEEL_ANXIOUS_Y2  2272  0.86  0.86  0.80   0.74 0.86 0.95

Non missing response frequency for each item
                    0    1    2    3 miss
DEPRESSED_Y2     0.47 0.32 0.12 0.09 0.61
INTEREST_Y2      0.40 0.37 0.13 0.10 0.61
CONTROL_WORRY_Y2 0.46 0.30 0.13 0.12 0.61
FEEL_ANXIOUS_Y2  0.44 0.35 0.12 0.09 0.61
## ============================================================================ ##
## Country: Nigeria

## Coefficient alpha (survey weighted): 0.713

## Coefficeint alpha without survey adjustments:

Reliability analysis
Call: psych::alpha(x = tmp.dat[, PHQ4.items], warnings = FALSE)

  raw_alpha std.alpha G6(smc) average_r S/N    ase mean   sd median_r
      0.72      0.72    0.66      0.39 2.5 0.0056  1.2 0.78     0.39

    95% confidence boundaries
         lower alpha upper
Feldt     0.71  0.72  0.73
Duhachek  0.71  0.72  0.73

 Reliability if an item is dropped:
                 raw_alpha std.alpha G6(smc) average_r S/N alpha se   var.r med.r
DEPRESSED_Y2          0.63      0.63    0.54      0.37 1.7   0.0077 0.00365  0.35
INTEREST_Y2           0.70      0.70    0.61      0.43 2.3   0.0064 0.00031  0.43
CONTROL_WORRY_Y2      0.64      0.65    0.55      0.38 1.8   0.0075 0.00463  0.36
FEEL_ANXIOUS_Y2       0.64      0.64    0.55      0.38 1.8   0.0075 0.00124  0.36
```

```
 Item statistics
                    n raw.r std.r r.cor r.drop mean  sd
DEPRESSED_Y2     3139  0.76  0.76  0.64   0.54  1.2 1.0
INTEREST_Y2      3138  0.69  0.69  0.51   0.44  1.5 1.1
CONTROL_WORRY_Y2 3135  0.75  0.75  0.62   0.52  1.2 1.1
FEEL_ANXIOUS_Y2  3131  0.75  0.75  0.62   0.52  1.1 1.0

Non missing response frequency for each item
                    0    1    2    3 miss
DEPRESSED_Y2     0.33 0.32 0.21 0.14 0.54
INTEREST_Y2      0.22 0.34 0.22 0.23 0.54
CONTROL_WORRY_Y2 0.33 0.32 0.17 0.17 0.54
FEEL_ANXIOUS_Y2  0.37 0.30 0.19 0.13 0.54
## ============================================================================ ##
## Country: Philippines

## Coefficient alpha (survey weighted): 0.726

## Coefficeint alpha without survey adjustments:

Reliability analysis
Call: psych::alpha(x = tmp.dat[, PHQ4.items], warnings = FALSE)

  raw_alpha std.alpha G6(smc) average_r S/N    ase mean   sd median_r
      0.72      0.72    0.67      0.39 2.6 0.0062  1.3 0.79     0.37

    95% confidence boundaries
         lower alpha upper
Feldt     0.71  0.72  0.73
Duhachek  0.71  0.72  0.73

 Reliability if an item is dropped:
                 raw_alpha std.alpha G6(smc) average_r S/N alpha se  var.r med.r
DEPRESSED_Y2          0.65      0.65    0.56      0.38 1.9   0.0083 0.0087  0.34
INTEREST_Y2           0.71      0.71    0.63      0.46 2.5   0.0068 0.0015  0.46
CONTROL_WORRY_Y2      0.64      0.64    0.55      0.37 1.8   0.0086 0.0059  0.33
FEEL_ANXIOUS_Y2       0.63      0.63    0.53      0.36 1.7   0.0088 0.0021  0.34

 Item statistics
                    n raw.r std.r r.cor r.drop mean  sd
DEPRESSED_Y2     2680  0.74  0.75  0.62   0.52  1.1 1.0
INTEREST_Y2      2677  0.67  0.67  0.48   0.41  1.5 1.1
```

```
CONTROL_WORRY_Y2 2681  0.77  0.76  0.64   0.54  1.5 1.2
FEEL_ANXIOUS_Y2  2682  0.77  0.77  0.67   0.56  1.1 1.1

Non missing response frequency for each item
                    0    1    2    3 miss
DEPRESSED_Y2     0.37 0.30 0.22 0.12 0.49
INTEREST_Y2      0.22 0.30 0.28 0.21 0.49
CONTROL_WORRY_Y2 0.25 0.26 0.20 0.29 0.49
FEEL_ANXIOUS_Y2  0.38 0.27 0.22 0.13 0.49
## ============================================================================ ##
## Country: Poland

## Coefficient alpha (survey weighted): 0.854

## Coefficeint alpha without survey adjustments:

Reliability analysis
Call: psych::alpha(x = tmp.dat[, PHQ4.items], warnings = FALSE)

  raw_alpha std.alpha G6(smc) average_r S/N    ase mean   sd median_r
      0.83      0.83     0.8      0.56   5 0.0027 0.43 0.54     0.56

    95% confidence boundaries
         lower alpha upper
Feldt     0.83  0.83  0.84
Duhachek  0.83  0.83  0.84

 Reliability if an item is dropped:
                 raw_alpha std.alpha G6(smc) average_r S/N alpha se  var.r med.r
DEPRESSED_Y2          0.77      0.78    0.70      0.54 3.5   0.0039 0.0039  0.52
INTEREST_Y2           0.81      0.81    0.74      0.58 4.2   0.0033 0.0026  0.61
CONTROL_WORRY_Y2      0.77      0.77    0.70      0.53 3.4   0.0039 0.0027  0.53
FEEL_ANXIOUS_Y2       0.80      0.80    0.74      0.58 4.1   0.0034 0.0026  0.59

 Item statistics
                    n raw.r std.r r.cor r.drop mean   sd
DEPRESSED_Y2     6445  0.83  0.84  0.76   0.69 0.35 0.63
INTEREST_Y2      6453  0.80  0.79  0.68   0.62 0.52 0.70
CONTROL_WORRY_Y2 6443  0.83  0.84  0.77   0.70 0.34 0.63
FEEL_ANXIOUS_Y2  6434  0.80  0.80  0.70   0.63 0.52 0.67

Non missing response frequency for each item
                    0    1    2    3 miss
```

```
DEPRESSED_Y2     0.72 0.22 0.04 0.01 0.38
INTEREST_Y2      0.59 0.32 0.07 0.02 0.38
CONTROL_WORRY_Y2 0.73 0.21 0.04 0.01 0.38
FEEL_ANXIOUS_Y2  0.56 0.37 0.05 0.02 0.38
## ============================================================================ ##
## Country: South Africa

## Coefficient alpha (survey weighted): 0.699

## Coefficeint alpha without survey adjustments:

Reliability analysis
Call: psych::alpha(x = tmp.dat[, PHQ4.items], warnings = FALSE)

  raw_alpha std.alpha G6(smc) average_r S/N    ase mean   sd median_r
      0.7       0.7    0.64      0.37 2.3 0.0095  1.2 0.75     0.38

    95% confidence boundaries
         lower alpha upper
Feldt     0.68   0.7  0.72
Duhachek  0.68   0.7  0.72

 Reliability if an item is dropped:
                 raw_alpha std.alpha G6(smc) average_r S/N alpha se   var.r med.r
DEPRESSED_Y2          0.61      0.61    0.51      0.34 1.5    0.013 0.00172  0.33
INTEREST_Y2           0.66      0.66    0.57      0.40 2.0    0.011 0.00027  0.39
CONTROL_WORRY_Y2      0.63      0.63    0.53      0.36 1.7    0.013 0.00207  0.38
FEEL_ANXIOUS_Y2       0.64      0.64    0.55      0.37 1.8    0.012 0.00204  0.38

 Item statistics
                   n raw.r std.r r.cor r.drop mean  sd
DEPRESSED_Y2     977  0.75  0.75  0.63   0.53  1.2 1.0
INTEREST_Y2      976  0.69  0.69  0.52   0.44  1.2 1.0
CONTROL_WORRY_Y2 977  0.74  0.73  0.60   0.50  1.3 1.1
FEEL_ANXIOUS_Y2  978  0.72  0.72  0.56   0.47  1.1 1.0

Non missing response frequency for each item
                    0    1    2    3 miss
DEPRESSED_Y2     0.29 0.37 0.18 0.16 0.63
INTEREST_Y2      0.30 0.38 0.17 0.14 0.63
CONTROL_WORRY_Y2 0.30 0.32 0.18 0.19 0.63
FEEL_ANXIOUS_Y2  0.36 0.35 0.16 0.13 0.63
## ============================================================================ ##
```

```
## Country: Spain

## Coefficient alpha (survey weighted): 0.854

## Coefficeint alpha without survey adjustments:

Reliability analysis
Call: psych::alpha(x = tmp.dat[, PHQ4.items], warnings = FALSE)

  raw_alpha std.alpha G6(smc) average_r S/N    ase mean   sd median_r
      0.86      0.86    0.82       0.6   6 0.0029  1.1 0.82     0.62

    95% confidence boundaries
         lower alpha upper
Feldt     0.85  0.86  0.86
Duhachek  0.85  0.86  0.86

 Reliability if an item is dropped:
                 raw_alpha std.alpha G6(smc) average_r S/N alpha se   var.r med.r
DEPRESSED_Y2          0.80      0.80    0.73      0.57 4.0   0.0043 3.2e-
03  0.55
INTEREST_Y2           0.84      0.84    0.78      0.63 5.2   0.0035 3.2e-
05  0.64
CONTROL_WORRY_Y2      0.81      0.81    0.75      0.59 4.4   0.0041 2.7e-
03  0.62
FEEL_ANXIOUS_Y2       0.82      0.82    0.75      0.60 4.5   0.0040 2.0e-
03  0.62

 Item statistics
                    n raw.r std.r r.cor r.drop mean   sd
DEPRESSED_Y2     2918  0.86  0.86  0.80   0.74 0.97 0.98
INTEREST_Y2      2919  0.80  0.81  0.70   0.65 1.05 0.94
CONTROL_WORRY_Y2 2920  0.85  0.84  0.77   0.71 0.99 1.01
FEEL_ANXIOUS_Y2  2915  0.84  0.84  0.76   0.70 1.23 0.98

Non missing response frequency for each item
                    0    1    2    3 miss
DEPRESSED_Y2     0.38 0.37 0.14 0.11 0.54
INTEREST_Y2      0.32 0.41 0.18 0.10 0.54
CONTROL_WORRY_Y2 0.40 0.33 0.16 0.12 0.54
FEEL_ANXIOUS_Y2  0.23 0.45 0.16 0.15 0.54
## ============================================================================ ##
## Country: Sweden
```

```
## Coefficient alpha (survey weighted): 0.854

## Coefficeint alpha without survey adjustments:

Reliability analysis
Call: psych::alpha(x = tmp.dat[, PHQ4.items], warnings = FALSE)

  raw_alpha std.alpha G6(smc) average_r S/N   ase mean   sd median_r
      0.85      0.86    0.84       0.6   6 0.002 0.67 0.74     0.61

    95% confidence boundaries
         lower alpha upper
Feldt     0.85  0.85  0.86
Duhachek  0.85  0.85  0.86

 Reliability if an item is dropped:
                 raw_alpha std.alpha G6(smc) average_r S/N alpha se  var.r med.r
DEPRESSED_Y2          0.78      0.79    0.76      0.56 3.8   0.0032 0.0408  0.45
INTEREST_Y2           0.89      0.89    0.85      0.73 8.1   0.0016 0.0028  0.70
CONTROL_WORRY_Y2      0.78      0.79    0.74      0.55 3.7   0.0031 0.0174  0.53
FEEL_ANXIOUS_Y2       0.79      0.79    0.74      0.56 3.8   0.0030 0.0170  0.53

 Item statistics
                     n raw.r std.r r.cor r.drop mean   sd
DEPRESSED_Y2     11575  0.87  0.88  0.82   0.76 0.65 0.87
INTEREST_Y2      11515  0.73  0.72  0.56   0.52 0.78 0.94
CONTROL_WORRY_Y2 11565  0.87  0.88  0.85   0.77 0.54 0.84
FEEL_ANXIOUS_Y2  11560  0.87  0.87  0.84   0.75 0.73 0.87

Non missing response frequency for each item
                    0    1    2    3 miss
DEPRESSED_Y2     0.56 0.30 0.08 0.06 0.23
INTEREST_Y2      0.51 0.27 0.15 0.07 0.24
CONTROL_WORRY_Y2 0.64 0.23 0.08 0.05 0.23
FEEL_ANXIOUS_Y2  0.49 0.35 0.09 0.06 0.23
## ============================================================================ ##
## Country: Tanzania

## Coefficient alpha (survey weighted): 0.692

## Coefficeint alpha without survey adjustments:
```

```
Reliability analysis
Call: psych::alpha(x = tmp.dat[, PHQ4.items], warnings = FALSE)

  raw_alpha std.alpha G6(smc) average_r S/N    ase mean  sd median_r
       0.7       0.7    0.65      0.37 2.4 0.0052    1 0.8     0.35

    95% confidence boundaries
         lower alpha upper
Feldt     0.69   0.7  0.71
Duhachek  0.69   0.7  0.71

 Reliability if an item is dropped:
                 raw_alpha std.alpha G6(smc) average_r S/N alpha se  var.r med.r
DEPRESSED_Y2          0.62      0.62    0.55      0.36 1.7   0.0071 0.0214  0.27
INTEREST_Y2           0.72      0.72    0.64      0.47 2.6   0.0051 0.0030  0.46
CONTROL_WORRY_Y2      0.61      0.61    0.52      0.34 1.6   0.0072 0.0103  0.29
FEEL_ANXIOUS_Y2       0.59      0.59    0.50      0.33 1.4   0.0075 0.0064  0.29

 Item statistics
                    n raw.r std.r r.cor r.drop mean  sd
DEPRESSED_Y2     5571  0.74  0.75  0.61   0.51 1.18 1.1
INTEREST_Y2      5567  0.65  0.63  0.41   0.35 1.21 1.2
CONTROL_WORRY_Y2 5568  0.75  0.76  0.65   0.53 0.87 1.1
FEEL_ANXIOUS_Y2  5572  0.77  0.78  0.68   0.56 0.87 1.1

Non missing response frequency for each item
                    0    1    2    3 miss
DEPRESSED_Y2     0.40 0.17 0.29 0.14 0.39
INTEREST_Y2      0.42 0.16 0.23 0.20 0.39
CONTROL_WORRY_Y2 0.55 0.13 0.22 0.10 0.39
FEEL_ANXIOUS_Y2  0.54 0.13 0.24 0.09 0.39
## ============================================================================ ##
## Country: Turkey

## Coefficient alpha (survey weighted): 0.821

## Coefficeint alpha without survey adjustments:

Reliability analysis
Call: psych::alpha(x = tmp.dat[, PHQ4.items], warnings = FALSE)

  raw_alpha std.alpha G6(smc) average_r S/N    ase mean   sd median_r
      0.85      0.85    0.82      0.58 5.6 0.0065  1.2 0.94      0.6
```

```
    95% confidence boundaries
         lower alpha upper
Feldt     0.84  0.85  0.86
Duhachek  0.84  0.85  0.86

 Reliability if an item is dropped:
                 raw_alpha std.alpha G6(smc) average_r S/N alpha se  var.r med.r
DEPRESSED_Y2          0.78      0.78    0.71      0.54 3.5   0.0101 0.0055  0.54
INTEREST_Y2           0.84      0.84    0.77      0.63 5.1   0.0074 0.0020  0.61
CONTROL_WORRY_Y2      0.82      0.82    0.77      0.61 4.7   0.0080 0.0050  0.62
FEEL_ANXIOUS_Y2       0.79      0.79    0.73      0.56 3.8   0.0095 0.0066  0.59

 Item statistics
                   n raw.r std.r r.cor r.drop mean  sd
DEPRESSED_Y2     498  0.87  0.87  0.82   0.76 1.22 1.1
INTEREST_Y2      497  0.79  0.79  0.68   0.62 1.26 1.2
CONTROL_WORRY_Y2 499  0.80  0.81  0.70   0.65 0.92 1.1
FEEL_ANXIOUS_Y2  499  0.85  0.85  0.79   0.72 1.36 1.1

Non missing response frequency for each item
                    0    1    2    3 miss
DEPRESSED_Y2     0.36 0.25 0.18 0.20 0.66
INTEREST_Y2      0.37 0.23 0.19 0.22 0.66
CONTROL_WORRY_Y2 0.49 0.24 0.13 0.14 0.66
FEEL_ANXIOUS_Y2  0.29 0.29 0.19 0.23 0.66
## ============================================================================ ##
## Country: United Kingdom

## Coefficient alpha (survey weighted): 0.904

## Coefficeint alpha without survey adjustments:

Reliability analysis
Call: psych::alpha(x = tmp.dat[, PHQ4.items], warnings = FALSE)

  raw_alpha std.alpha G6(smc) average_r S/N    ase mean   sd median_r
       0.9       0.9    0.89       0.7 9.2 0.0022 0.79 0.83     0.71

    95% confidence boundaries
         lower alpha upper
Feldt      0.9   0.9  0.91
Duhachek   0.9   0.9  0.91
```

```
 Reliability if an item is dropped:
                 raw_alpha std.alpha G6(smc) average_r S/N alpha se  var.r med.r
DEPRESSED_Y2          0.86      0.86    0.82      0.67 6.2   0.0034 0.0109  0.62
INTEREST_Y2           0.89      0.89    0.85      0.73 8.3   0.0025 0.0026  0.72
CONTROL_WORRY_Y2      0.87      0.87    0.82      0.69 6.5   0.0032 0.0057  0.70
FEEL_ANXIOUS_Y2       0.87      0.87    0.83      0.70 6.9   0.0030 0.0050  0.72

 Item statistics
                    n raw.r std.r r.cor r.drop mean   sd
DEPRESSED_Y2     3596  0.90  0.90  0.86   0.82 0.76 0.93
INTEREST_Y2      3610  0.85  0.85  0.77   0.73 0.82 0.96
CONTROL_WORRY_Y2 3612  0.89  0.89  0.85   0.80 0.75 0.95
FEEL_ANXIOUS_Y2  3601  0.88  0.88  0.83   0.78 0.82 0.94

Non missing response frequency for each item
                    0    1    2    3 miss
DEPRESSED_Y2     0.51 0.30 0.12 0.07 0.33
INTEREST_Y2      0.48 0.29 0.14 0.08 0.33
CONTROL_WORRY_Y2 0.53 0.28 0.11 0.08 0.33
FEEL_ANXIOUS_Y2  0.47 0.32 0.12 0.08 0.33
## ==================================================================== ##
## Country: United States

## Coefficient alpha (survey weighted): 0.886

## Coefficeint alpha without survey adjustments:

Reliability analysis
Call: psych::alpha(x = tmp.dat[, PHQ4.items], warnings = FALSE)

  raw_alpha std.alpha G6(smc) average_r S/N    ase mean   sd median_r
      0.87      0.87    0.86      0.63 6.8 0.0011 0.57 0.67     0.62

    95% confidence boundaries
         lower alpha upper
Feldt     0.87  0.87  0.87
Duhachek  0.87  0.87  0.87

 Reliability if an item is dropped:
                 raw_alpha std.alpha G6(smc) average_r S/N alpha se  var.r med.r
DEPRESSED_Y2          0.82      0.82    0.76      0.60 4.4   0.0016 0.0133  0.54
INTEREST_Y2           0.85      0.85    0.80      0.66 5.8   0.0013 0.0037  0.63
```

```
CONTROL_WORRY_Y2      0.83      0.83     0.78      0.62 5.0   0.0015 0.0111  0.62
FEEL_ANXIOUS_Y2       0.84      0.84     0.79      0.63 5.2   0.0015 0.0094  0.63

 Item statistics
                     n raw.r std.r r.cor r.drop mean   sd
DEPRESSED_Y2     32155  0.87  0.88  0.83   0.77 0.50 0.75
INTEREST_Y2      32118  0.82  0.82  0.74   0.68 0.54 0.78
CONTROL_WORRY_Y2 32194  0.86  0.85  0.79   0.73 0.55 0.80
FEEL_ANXIOUS_Y2  32147  0.85  0.84  0.78   0.72 0.67 0.81

Non missing response frequency for each item
                    0    1    2    3 miss
DEPRESSED_Y2     0.63 0.28 0.06 0.03 0.16
INTEREST_Y2      0.60 0.29 0.08 0.03 0.16
CONTROL_WORRY_Y2 0.60 0.28 0.07 0.04 0.16
FEEL_ANXIOUS_Y2  0.50 0.37 0.08 0.05 0.16
```

3.2.1 Summary table

```r
## Alpha summary table
list.alpha |>
  bind_rows(.id="Country") |>
  rename(
    unadj.alpha = 3,
    unadj.alpha.ci.lb = 4,
    unadj.alpha.ci.ub = 5
  )|>
  arrange(svyalpha) |>
  flextable() |>
  colformat_double(digits = 3) |>
  add_header_lines("Summary of coefficient alpha estimates") |>
  autofit()
```

Summary of coefficient alpha estimates				
Country	svyalpha	unadj.alpha	unadj.alpha.ci.lb	unadj.alpha.ci.ub
Tanzania	0.692	0.699	0.689	0.709
India	0.695	0.698	0.689	0.706
South Africa	0.699	0.699	0.680	0.717

Summary of coefficient alpha estimates				
Country	svyalpha	unadj.alpha	unadj.alpha.ci.lb	unadj.alpha.ci.ub
Nigeria	0.713	0.717	0.706	0.728
Philippines	0.726	0.721	0.709	0.733
Kenya	0.738	0.736	0.728	0.744
Egypt	0.777	0.766	0.754	0.776
Indonesia	0.799	0.817	0.810	0.824
China	0.811	0.814	0.805	0.822
Turkey	0.821	0.848	0.835	0.861
Brazil	0.841	0.853	0.849	0.857
Germany	0.849	0.862	0.857	0.866
Argentina	0.852	0.858	0.852	0.863
Hong Kong	0.853	0.848	0.839	0.856
Poland	0.854	0.832	0.827	0.837
Sweden	0.854	0.853	0.849	0.857
Spain	0.854	0.857	0.851	0.863
Mexico	0.859	0.863	0.858	0.869
Australia	0.874	0.872	0.865	0.879
Israel	0.876	0.872	0.865	0.879
United States	0.886	0.871	0.868	0.873
United Kingdom	0.904	0.902	0.898	0.906
Japan	0.910	0.914	0.912	0.916

4 Multi-Group CFA

Next, multi-group CFA models were estimates using increasingly restrictive tests of invariance for completeness. The separate country CFA analsyes conducted previously provide a more holistic assessment of the uniqueness of the measurement properties within each country.

```
## Need to re-structure the lists for input into lavaan

lst.cov <- list()
lst.mean <- list()
lst.ng <- list()
#lst.th <- list()
#lst.nacov <- list()
#lst.wls.v <- list()

for(cur.country in COUNTRIES){
  lst.cov[[cur.country]] <- sample.stats[[cur.country]]$cov
  lst.mean[[cur.country]] <- sample.stats[[cur.country]]$mean
  lst.ng[[cur.country]] <- sample.stats[[cur.country]]$sample.nobs
  #lst.th[[cur.country]] <- sample.stats[[cur.country]]$sample.th
  #lst.nacov[[cur.country]] <- sample.stats[[cur.country]]$NACOV
  #lst.wls.v[[cur.country]] <- sample.stats[[cur.country]]$WLS.V
}
```

Note. Do to how many groups there are (ng=23), the output of the specific models is not printed.

4.1 Covariance matrix input – allows for direct replication

- Pro: Fully accounts for complex sampling design within each country.
- Con: Does not account for the discrete nature of these data with a categorical model.

4.1.1 Configural Model

```
fit.config <- cfa(
  mod, std.lv = TRUE, ordered=TRUE,
  sample.cov = lst.cov,
  sample.mean = lst.mean,
  sample.nobs = lst.ng
)

fit.config
```

```
lavaan 0.6-21 ended normally after 267 iterations

  Estimator                                         ML
  Optimization method                           NLMINB
  Number of model parameters                       299

  Number of observations per group:
    Argentina                                 2932.00000000153
    Australia                                 2582.00004721915
    Brazil                                    4274.00000072482
    China                                     4544.00000002019
    Egypt                                     3040.00004437456
    Germany                                   5529.00000403925
    Hong Kong                                 707.000248016404
    India                                     6374.00000045487
    Indonesia                                   2684.000001417
    Israel                                    2490.00002081761
    Japan                                     13972.0000004316
    Kenya                                     7698.00001112736
    Mexico                                    2278.00000004553
    Nigeria                                   3146.00011189409
    Philippines                               2682.00024216278
    Poland                                    6478.00001620523
    South Africa                              978.000000002357
    Spain                                     2924.00000076902
    Sweden                                    11609.0108778427
    Tanzania                                  5583.00000008529
    Turkey                                    500.000000452455
    United Kingdom                            3619.00000002024
    United States                             32245.0003114482
```

```
Model Test User Model:

  Test statistic                               164.448
  Degrees of freedom                                23
  P-value (Chi-square)                           0.000
  Test statistic for each group:
    Argentina                                    6.916
    Australia                                    1.092
    Brazil                                      15.986
    China                                        9.389
    Egypt                                        0.110
    Germany                                      0.023
    Hong Kong                                    0.042
    India                                       26.214
    Indonesia                                   12.422
    Israel                                      26.343
    Japan                                       11.075
    Kenya                                        2.321
    Mexico                                       7.137
    Nigeria                                      4.365
    Philippines                                  7.918
    Poland                                      13.170
    South Africa                                 0.001
    Spain                                       11.909
    Sweden                                       1.741
    Tanzania                                     0.582
    Turkey                                       0.884
    United Kingdom                               1.554
    United States                                3.255
```

```
#### Modification indices
tb_mod_indices(fit.config, sort.=TRUE, maximum.number = 20)
```

lhs	op	rhs	block	group	level	mi
DEPRESSED_Y2	~~	CONTROL_WORRY_Y2	10	10	1	26.20
INTEREST_Y2	~~	FEEL_ANXIOUS_Y2	10	10	1	26.20
INTEREST_Y2	~~	CONTROL_WORRY_Y2	10	10	1	26.20
DEPRESSED_Y2	~~	FEEL_ANXIOUS_Y2	10	10	1	26.20

lhs	op	rhs	block	group	level	mi
DEPRESSED_Y2	~~	FEEL_ANXIOUS_Y2	8	8	1	26.16
INTEREST_Y2	~~	FEEL_ANXIOUS_Y2	8	8	1	26.16
INTEREST_Y2	~~	CONTROL_WORRY_Y2	8	8	1	26.16
DEPRESSED_Y2	~~	CONTROL_WORRY_Y2	8	8	1	26.16
INTEREST_Y2	~~	FEEL_ANXIOUS_Y2	3	3	1	15.96
DEPRESSED_Y2	~~	CONTROL_WORRY_Y2	3	3	1	15.96
INTEREST_Y2	~~	CONTROL_WORRY_Y2	3	3	1	15.96
DEPRESSED_Y2	~~	FEEL_ANXIOUS_Y2	3	3	1	15.96
DEPRESSED_Y2	~~	FEEL_ANXIOUS_Y2	16	16	1	13.16
INTEREST_Y2	~~	FEEL_ANXIOUS_Y2	16	16	1	13.16
DEPRESSED_Y2	~~	CONTROL_WORRY_Y2	16	16	1	13.16
INTEREST_Y2	~~	CONTROL_WORRY_Y2	16	16	1	13.16
DEPRESSED_Y2	~~	FEEL_ANXIOUS_Y2	9	9	1	12.39
DEPRESSED_Y2	~~	CONTROL_WORRY_Y2	9	9	1	12.39
INTEREST_Y2	~~	FEEL_ANXIOUS_Y2	9	9	1	12.39
INTEREST_Y2	~~	CONTROL_WORRY_Y2	9	9	1	12.39

4.1.2 Metric Invariance Model

```r
fit.metric <- cfa(
  mod, std.lv = TRUE,
  sample.cov = lst.cov,
  sample.mean = lst.mean,
  sample.nobs = lst.ng,
  group.equal = "loadings"
)

fit.metric
```

```
lavaan 0.6-21 ended normally after 356 iterations

  Estimator                                         ML
  Optimization method                           NLMINB
  Number of model parameters                       343
  Number of equality constraints                    88

  Number of observations per group:
    Argentina                            2932.00000000153
    Australia                            2582.00004721915
    Brazil                               4274.00000072482
    China                                4544.00000002019
    Egypt                                3040.00004437456
    Germany                              5529.00000403925
    Hong Kong                            707.000248016404
    India                                6374.00000045487
    Indonesia                              2684.000001417
    Israel                               2490.00002081761
    Japan                                13972.0000004316
    Kenya                                7698.00001112736
    Mexico                               2278.00000004553
    Nigeria                              3146.00011189409
    Philippines                          2682.00024216278
    Poland                               6478.00001620523
    South Africa                         978.000000002357
    Spain                                2924.00000076902
    Sweden                               11609.0108778427
    Tanzania                             5583.00000008529
    Turkey                               500.000000452455
```

```
    United Kingdom                              3619.00000002024
    United States                               32245.0003114482

Model Test User Model:

  Test statistic                              1228.537
  Degrees of freedom                                67
  P-value (Chi-square)                           0.000
  Test statistic for each group:
    Argentina                                    9.375
    Australia                                    9.694
    Brazil                                      25.169
    China                                       41.106
    Egypt                                       42.362
    Germany                                     28.282
    Hong Kong                                   29.240
    India                                      153.808
    Indonesia                                   51.963
    Israel                                      48.200
    Japan                                      341.269
    Kenya                                        5.839
    Mexico                                       8.697
    Nigeria                                      4.839
    Philippines                                 12.627
    Poland                                      80.887
    South Africa                                 2.058
    Spain                                       19.883
    Sweden                                     138.476
    Tanzania                                    35.314
    Turkey                                       4.899
    United Kingdom                              28.989
    United States                              105.561
```

```r
#### Modification indices
tb_mod_indices(fit.metric, sort.=TRUE, maximum.number = 20)
```

lhs	op	rhs	block	group	level	mi
dep	=~	FEEL_ANXIOUS_Y2	11	11	1	366.60
dep	=~	CONTROL_WORRY_Y2	11	11	1	366.60
anx	=~	DEPRESSED_Y2	19	19	1	145.49

lhs	op	rhs	block	group	level	mi
anx	=~	INTEREST_Y2	19	19	1	145.49
anx	=~	DEPRESSED_Y2	8	8	1	122.02
anx	=~	INTEREST_Y2	8	8	1	122.02
INTEREST_Y2	~~	FEEL_ANXIOUS_Y2	8	8	1	92.27
dep	=~	CONTROL_WORRY_Y2	23	23	1	87.63
dep	=~	FEEL_ANXIOUS_Y2	23	23	1	87.63
DEPRESSED_Y2	~~	FEEL_ANXIOUS_Y2	8	8	1	78.49
anx	=~	INTEREST_Y2	23	23	1	67.21
anx	=~	DEPRESSED_Y2	23	23	1	67.21
DEPRESSED_Y2	~~	CONTROL_WORRY_Y2	11	11	1	60.60
DEPRESSED_Y2	~~	FEEL_ANXIOUS_Y2	23	23	1	58.36
INTEREST_Y2	~~	FEEL_ANXIOUS_Y2	11	11	1	58.22
DEPRESSED_Y2	~~	FEEL_ANXIOUS_Y2	16	16	1	54.45
anx	=~	DEPRESSED_Y2	16	16	1	44.48
anx	=~	INTEREST_Y2	16	16	1	44.48
DEPRESSED_Y2	~~	FEEL_ANXIOUS_Y2	9	9	1	43.03
anx	=~	DEPRESSED_Y2	5	5	1	41.44

4.1.3 Scalar Invariance Model

```
fit.scalar <- cfa(
  mod, std.lv = TRUE,
  sample.cov = lst.cov,
  sample.mean = lst.mean,
  sample.nobs = lst.ng,
  group.equal = c("loadings", "intercepts")
)

fit.scalar
```

```
lavaan 0.6-21 ended normally after 398 iterations

  Estimator                                         ML
  Optimization method                           NLMINB
  Number of model parameters                       387
  Number of equality constraints                   176

  Number of observations per group:
    Argentina                                2932.00000000153
    Australia                                2582.00004721915
    Brazil                                   4274.00000072482
    China                                    4544.00000002019
    Egypt                                    3040.00004437456
    Germany                                  5529.00000403925
    Hong Kong                                707.000248016404
    India                                    6374.00000045487
    Indonesia                                  2684.000001417
    Israel                                   2490.00002081761
    Japan                                    13972.0000004316
    Kenya                                    7698.00001112736
    Mexico                                   2278.00000004553
    Nigeria                                  3146.00011189409
    Philippines                              2682.00024216278
    Poland                                   6478.00001620523
    South Africa                             978.000000002357
    Spain                                    2924.00000076902
    Sweden                                   11609.0108778427
    Tanzania                                 5583.00000008529
    Turkey                                   500.000000452455
```

```
    United Kingdom                          3619.00000002024
    United States                           32245.0003114482

Model Test User Model:

  Test statistic                              6620.295
  Degrees of freedom                               111
  P-value (Chi-square)                           0.000
  Test statistic for each group:
    Argentina                                  103.415
    Australia                                   22.722
    Brazil                                     235.722
    China                                      267.819
    Egypt                                      181.012
    Germany                                    535.131
    Hong Kong                                   95.954
    India                                      599.286
    Indonesia                                  185.466
    Israel                                     355.942
    Japan                                     1307.276
    Kenya                                      350.820
    Mexico                                     153.423
    Nigeria                                    284.882
    Philippines                                830.715
    Poland                                     146.147
    South Africa                                70.615
    Spain                                       73.750
    Sweden                                     329.139
    Tanzania                                   126.377
    Turkey                                      50.148
    United Kingdom                              55.421
    United States                              259.111
```

```r
#### Modification indices
tb_mod_indices(fit.scalar, sort.=TRUE, maximum.number = 20)
```

lhs	op	rhs	block	group	level	mi
anx	=~	DEPRESSED_Y2	19	19	1	190.46[illegible]
anx	=~	INTEREST_Y2	19	19	1	190.46[illegible]
anx	=~	DEPRESSED_Y2	9	9	1	163.79[illegible]

lhs	op	rhs	block	group	level	mi
dep	=~	CONTROL_WORRY_Y2	15	15	1	143.64
INTEREST_Y2	~~	FEEL_ANXIOUS_Y2	8	8	1	125.81
dep	=~	CONTROL_WORRY_Y2	10	10	1	121.46
dep	=~	FEEL_ANXIOUS_Y2	10	10	1	121.45
DEPRESSED_Y2	~~	FEEL_ANXIOUS_Y2	8	8	1	120.33
dep	=~	FEEL_ANXIOUS_Y2	11	11	1	118.86
dep	=~	CONTROL_WORRY_Y2	11	11	1	118.86
DEPRESSED_Y2	~~	FEEL_ANXIOUS_Y2	9	9	1	117.07
anx	=~	INTEREST_Y2	4	4	1	116.86
anx	=~	DEPRESSED_Y2	4	4	1	116.86
anx	=~	INTEREST_Y2	6	6	1	113.87
anx	=~	DEPRESSED_Y2	6	6	1	113.87
CONTROL_WORRY_Y2	~~	FEEL_ANXIOUS_Y2	9	9	1	112.62
dep	=~	FEEL_ANXIOUS_Y2	15	15	1	112.18
anx	=~	INTEREST_Y2	8	8	1	101.93
anx	=~	DEPRESSED_Y2	8	8	1	101.93
DEPRESSED_Y2	~~	FEEL_ANXIOUS_Y2	15	15	1	92.39

4.1.4 Strict Invariance Model

```
fit.strict <- cfa(
  mod, std.lv = TRUE,
  sample.cov = lst.cov,
  sample.mean = lst.mean,
  sample.nobs = lst.ng,
  group.equal = c("loadings", "intercepts", "residuals")
)

fit.strict
```

```
lavaan 0.6-21 ended normally after 338 iterations

  Estimator                                         ML
  Optimization method                           NLMINB
  Number of model parameters                       387
  Number of equality constraints                   264

  Number of observations per group:
    Argentina                           2932.00000000153
    Australia                           2582.00004721915
    Brazil                              4274.00000072482
    China                               4544.00000002019
    Egypt                               3040.00004437456
    Germany                             5529.00000403925
    Hong Kong                           707.000248016404
    India                               6374.00000045487
    Indonesia                            2684.000001417
    Israel                              2490.00002081761
    Japan                               13972.0000004316
    Kenya                               7698.00001112736
    Mexico                              2278.00000004553
    Nigeria                             3146.00011189409
    Philippines                         2682.00024216278
    Poland                              6478.00001620523
    South Africa                        978.000000002357
    Spain                               2924.00000076902
    Sweden                              11609.0108778427
    Tanzania                            5583.00000008529
    Turkey                              500.000000452455
```

```
    United Kingdom                          3619.00000002024
    United States                           32245.0003114482

Model Test User Model:

  Test statistic                             47824.968
  Degrees of freedom                               199
  P-value (Chi-square)                           0.000
  Test statistic for each group:
    Argentina                                  113.875
    Australia                                  503.190
    Brazil                                     910.253
    China                                      800.341
    Egypt                                     1120.177
    Germany                                   1409.220
    Hong Kong                                  237.058
    India                                     6123.654
    Indonesia                                  285.494
    Israel                                     778.077
    Japan                                     5085.612
    Kenya                                     3319.665
    Mexico                                     216.073
    Nigeria                                   2171.421
    Philippines                               2847.441
    Poland                                    2210.430
    South Africa                               736.982
    Spain                                      202.186
    Sweden                                    2881.518
    Tanzania                                  4050.378
    Turkey                                     284.100
    United Kingdom                             644.615
    United States                            10893.208
```

```r
#### Modification indices
tb_mod_indices(fit.strict, sort.=TRUE, maximum.number = 20)
```

lhs	op	rhs	block	group	level	mi
DEPRESSED_Y2	~~	INTEREST_Y2	8	8	1	6,067.73
DEPRESSED_Y2	~~	INTEREST_Y2	20	20	1	5,691.27
DEPRESSED_Y2	~~	INTEREST_Y2	23	23	1	4,519.85

lhs	op	rhs	block	group	level	mi
CONTROL_WORRY_Y2	~~	FEEL_ANXIOUS_Y2	8	8	1	3,804.79
CONTROL_WORRY_Y2	~~	FEEL_ANXIOUS_Y2	23	23	1	3,375.01
CONTROL_WORRY_Y2	~~	FEEL_ANXIOUS_Y2	12	12	1	2,910.87
CONTROL_WORRY_Y2	~~	FEEL_ANXIOUS_Y2	15	15	1	2,147.70
CONTROL_WORRY_Y2	~~	FEEL_ANXIOUS_Y2	14	14	1	1,946.31
DEPRESSED_Y2	~~	INTEREST_Y2	12	12	1	1,906.53
CONTROL_WORRY_Y2	~~	FEEL_ANXIOUS_Y2	19	19	1	1,620.58
DEPRESSED_Y2	~~	INTEREST_Y2	15	15	1	1,473.30
CONTROL_WORRY_Y2	~~	FEEL_ANXIOUS_Y2	20	20	1	1,466.89
DEPRESSED_Y2	~~	INTEREST_Y2	14	14	1	1,278.69
DEPRESSED_Y2	~~	FEEL_ANXIOUS_Y2	11	11	1	966.09
DEPRESSED_Y2	~~	INTEREST_Y2	11	11	1	814.22
CONTROL_WORRY_Y2	~~	FEEL_ANXIOUS_Y2	17	17	1	800.73
DEPRESSED_Y2	~~	INTEREST_Y2	5	5	1	750.13
CONTROL_WORRY_Y2	~~	FEEL_ANXIOUS_Y2	3	3	1	730.54
CONTROL_WORRY_Y2	~~	FEEL_ANXIOUS_Y2	5	5	1	508.51
anx	=~	INTEREST_Y2	23	23	1	417.37

4.1.5 Fit Comparison

```r
# model comparison tests
summary(compareFit(fit.config, fit.metric, fit.scalar, fit.strict))
```

```
################### Nested Model Comparison #########################

Chi-Squared Difference Test

            Df     AIC     BIC    Chisq Chisq diff    RMSEA Df diff Pr(>Chisq)
fit.config  23 1138560 1141480   164.45
fit.metric  67 1139536 1142027  1228.54       1064 0.064326      44  < 2.2e-
16 ***
fit.scalar 111 1144840 1146901  6620.29       5392 0.147282      44  < 2.2e-
16 ***
fit.strict 199 1185869 1187070 47824.97      41205 0.288775      88  < 2.2e-
16 ***
---
Signif. codes:  0 '***' 0.001 '**' 0.01 '*' 0.05 '.' 0.1 ' ' 1

####################### Model Fit Indices ###########################
                chisq  df pvalue rmsea    cfi    tli  srmr          aic          bic
fit.config   164.448†  23   .000 .033† 0.999† 0.996† .003† 1138560.053† 1141480.249†
fit.metric  1228.537   67   .000 .056  0.995   .990  .025  1139536.142  1142026.610
fit.scalar  6620.295  111   .000 .102   .973   .966  .043  1144839.900  1146900.641
fit.strict 47824.968  199   .000 .207   .800   .861  .085  1185868.573  1187069.858

################## Differences in Fit Indices #######################
                        df rmsea    cfi    tli  srmr       aic       bic
fit.metric - fit.config 44 0.022 -0.004 -0.006 0.022   976.089   546.361
fit.scalar - fit.metric 44 0.047 -0.022 -0.024 0.018  5303.758  4874.030
fit.strict - fit.scalar 88 0.104 -0.173 -0.105 0.043 41028.673 40169.218
```

4.2 Person-level data input

- Pro: Accounted for the discrete nature of these data with a categorical model.
- Con: Does not account for complex sampling design within each country.

Summary of models to be fitted:

1. A fully invariant (baseline) model to estimate the degree of potential misfit.
2. Configural model

4.2.1 Full invariant–ignoring group membership

Model only have 1 degree of freedom so there is no room for freeing parameters.

```
fit.fullinvar <- cfa(
  model = mod
  , data = df.cc
  , std.lv = TRUE
  , ordered = TRUE
  , parameterization = "theta"
  , missing = "pairwise"
)

summary(fit.fullinvar, ci=TRUE, fit.measure=TRUE, standardize=TRUE)
```

```
lavaan 0.6-21 ended normally after 55 iterations

  Estimator                                         DWLS
  Optimization method                             NLMINB
  Number of model parameters                          17

                                                    Used       Total
  Number of observations                          128842      207919
  Number of missing patterns                          15

Model Test User Model:
                                                Standard      Scaled
  Test Statistic                                  36.919     135.375
  Degrees of freedom                                   1           1
  P-value (Unknown)                                   NA       0.000
  Scaling correction factor                                    0.273
  Shift parameter                                              0.000
```

```
    simple second-order correction

Model Test Baseline Model:

  Test statistic                              933071.313  645034.952
  Degrees of freedom                                   6           6
  P-value                                             NA       0.000
  Scaling correction factor                                    1.447

User Model versus Baseline Model:

  Comparative Fit Index (CFI)                      1.000       1.000
  Tucker-Lewis Index (TLI)                         1.000       0.999

  Robust Comparative Fit Index (CFI)                           0.999
  Robust Tucker-Lewis Index (TLI)                              0.995

Root Mean Square Error of Approximation:

  RMSEA                                            0.017       0.032
  90 Percent confidence interval - lower           0.012       0.028
  90 Percent confidence interval - upper           0.022       0.037
  P-value H_0: RMSEA <= 0.050                      1.000       1.000
  P-value H_0: RMSEA >= 0.080                      0.000       0.000

  Robust RMSEA                                                 0.044
  90 Percent confidence interval - lower                       0.038
  90 Percent confidence interval - upper                       0.051
  P-value H_0: Robust RMSEA <= 0.050                           0.930
  P-value H_0: Robust RMSEA >= 0.080                           0.000

Standardized Root Mean Square Residual:

  SRMR                                             0.004       0.004

Parameter Estimates:

  Parameterization                                 Theta
  Standard errors                             Robust.sem
  Information                                   Expected
  Information saturated (h1) model          Unstructured

Latent Variables:
```

```
                  Estimate  Std.Err  z-value  P(>|z|) ci.lower ci.upper   Std.lv  Std.all
  dep =~
    DEPRESSED_Y2     2.383    0.022  106.366    0.000    2.339    2.426    2.383    0.922
    INTEREST_Y2      1.172    0.006  191.938    0.000    1.160    1.184    1.172    0.761
  anx =~
    CONTROL_WORRY_   1.789    0.011  163.070    0.000    1.768    1.811    1.789    0.873
    FEEL_ANXIOUS_Y   1.715    0.010  180.359    0.000    1.696    1.733    1.715    0.864

Covariances:
                  Estimate  Std.Err  z-value  P(>|z|) ci.lower ci.upper   Std.lv  Std.all
  dep ~~
    anx              0.891    0.001  610.482    0.000    0.888    0.894    0.891    0.891

Thresholds:
                  Estimate  Std.Err  z-value  P(>|z|) ci.lower ci.upper   Std.lv  Std.all
    DEPRESSED_Y2|1   0.085    0.009    9.283    0.000    0.067    0.103    0.085    0.033
    DEPRESSED_Y2|2   2.318    0.020  115.581    0.000    2.279    2.357    2.318    0.897
    DEPRESSED_Y2|3   3.740    0.029  128.881    0.000    3.683    3.796    3.740    1.447
    INTEREST_Y2|t1  -0.152    0.005  -28.365    0.000   -0.162   -0.141   -
0.152   -0.099
    INTEREST_Y2|t2   1.150    0.006  178.862    0.000    1.138    1.163    1.150    0.747
    INTEREST_Y2|t3   2.057    0.008  259.828    0.000    2.042    2.073    2.057    1.335
    CONTROL_WORRY_   0.192    0.007   26.093    0.000    0.177    0.206    0.192    0.094
    CONTROL_WORRY_   1.787    0.011  168.670    0.000    1.766    1.808    1.787    0.872
    CONTROL_WORRY_   2.823    0.013  211.624    0.000    2.797    2.849    2.823    1.377
    FEEL_ANXIOUS_Y  -0.264    0.007  -38.616    0.000   -0.277   -0.251   -
0.264   -0.133
    FEEL_ANXIOUS_Y   1.643    0.009  175.460    0.000    1.625    1.661    1.643    0.828
    FEEL_ANXIOUS_Y   2.737    0.012  222.150    0.000    2.712    2.761    2.737    1.379

Variances:
                  Estimate  Std.Err  z-value  P(>|z|) ci.lower ci.upper   Std.lv  Std.all
   .DEPRESSED_Y2     1.000                               1.000    1.000    1.000    0.150
   .INTEREST_Y2      1.000                               1.000    1.000    1.000    0.421
   .CONTROL_WORRY_   1.000                               1.000    1.000    1.000    0.238
   .FEEL_ANXIOUS_Y   1.000                               1.000    1.000    1.000    0.254
    dep              1.000                               1.000    1.000    1.000    1.000
    anx              1.000                               1.000    1.000    1.000    1.000

Scales y*:
                  Estimate  Std.Err  z-value  P(>|z|) ci.lower ci.upper   Std.lv  Std.all
    DEPRESSED_Y2     0.387                               0.387    0.387    0.387    1.000
    INTEREST_Y2      0.649                               0.649    0.649    0.649    1.000
```

```
    CONTROL_WORRY_    0.488                               0.488    0.488    0.488    1.000
    FEEL_ANXIOUS_Y    0.504                               0.504    0.504    0.504    1.000
```

```r
#### Residuals
residuals(fit.fullinvar)
```

```
$type
[1] "raw"

$cov
                 DEPRES INTERE CONTRO FEEL_A
DEPRESSED_Y2      0.000
INTEREST_Y2       0.000  0.000
CONTROL_WORRY_Y2 -0.004  0.008  0.000
FEEL_ANXIOUS_Y2   0.004 -0.008  0.000  0.000

$mean
    DEPRESSED_Y2      INTEREST_Y2 CONTROL_WORRY_Y2  FEEL_ANXIOUS_Y2
               0                0                0                0

$th
    DEPRESSED_Y2|t1     DEPRESSED_Y2|t2     DEPRESSED_Y2|t3      INTEREST_Y2|t1      INTEREST
                  0                   0                   0                   0
 FEEL_ANXIOUS_Y2|t2  FEEL_ANXIOUS_Y2|t3
                  0                   0
```

Note. The largest residual correlation is between items Interest and Feel Anxious. However, given that analyses are conducted separately for the Depressed and Anxious factors, not accounting for a residual correlation cross-factors is not very important. Any major sources of potential non-invariance in factor loadings are of primary concern. Differences in level/location/scale of each item is not entirely problematic as the focal issue is on differences in how responses to the items relate to underlying factor and to other constructs (e.g., covariance not location).

We will now proceed with testing the invariance of the above model across countries to identify which countries contribute the most to misfit–the chi-square contribution specially.

4.2.2 Configural Model

Identification constraints:

- Latent response variable (LRV) distribution for each observed item cannot be uniquely identified in each country. Comparisons in the item location and scale not possible.
- LRV residual variance fixed to 1 in all countries
- LRV location fixed to 0 in all countries
- loadings freely estimated (not comparable across countries)
- thresholds freely estimated (not comparable across countries)
- factor means fixed to 0 in all countries (not comparable across countries)
- factor variances fixed to 1 in all countries (not comparable across countries)

```r
mod.config <- semTools::measEq.syntax(
  configural.model = mod
  , data = df.cc
  , group = "COUNTRY"
  , parameterization = "theta"
  , ID.fac = "std.lv"
  , ID.cat = "Wu.Estabrook.2016"
  , ordered = TRUE
)
summary(mod.config)
```

```
This lavaan model syntax specifies a CFA with 4 manifest indicators (4 of which are ordinal)

To identify the location and scale of each common factor, the factor means and variances were

The location and scale of each latent item-response underlying 4 ordinal indicators were iden

    https://doi.org/10.1007/s11336-016-9506-0

Pattern matrix indicating num(eric), ord(ered), and lat(ent) indicators per factor:

                 dep anx
DEPRESSED_Y2     ord
INTEREST_Y2      ord
CONTROL_WORRY_Y2     ord
FEEL_ANXIOUS_Y2      ord


This model hypothesizes only configural invariance.
```

```r
#cat(as.character(mod.config))
##
fit.config <- cfa(
  model = as.character(mod.config)
  , data = df.cc
  , group = "COUNTRY"
  , ordered = TRUE
  , parameterization = "theta"
  , missing = "pairwise"
)

fit.config
```

```
lavaan 0.6-21 ended normally after 2199 iterations

  Estimator                                         DWLS
  Optimization method                             NLMINB
  Number of model parameters                         391

  Number of observations per group:               Used       Total
    Sweden                                       11607       15068
    United Kingdom                                3619        5368
    Germany                                       5528        9506
    China                                         4544        5022
    Argentina                                     2928        6724
    Australia                                     2581        3844
    Brazil                                        4274       13203
    Egypt                                         3040        4729
    India                                         6372       12765
    Indonesia                                     2681        6992
    Israel                                        2489        3669
    Japan                                        13968       20543
    Kenya                                         7698       11389
    Mexico                                        2278        5776
    Nigeria                                       3146        6827
    Philippines                                   2682        5292
    Poland                                        6478       10389
    South Africa                                   978        2651
    Spain                                         2923        6290
    Tanzania                                      5583        9075
    Turkey                                         499        1473
    United States                                32239       38312
```

```
    Hong Kong                                    707        3012
  Number of missing patterns per group:
    Sweden                                        13
    United Kingdom                                 6
    Germany                                        8
    China                                          4
    Argentina                                     10
    Australia                                      7
    Brazil                                         8
    Egypt                                          5
    India                                         13
    Indonesia                                      6
    Israel                                         9
    Japan                                          9
    Kenya                                          6
    Mexico                                         8
    Nigeria                                        9
    Philippines                                    4
    Poland                                         9
    South Africa                                   4
    Spain                                          7
    Tanzania                                      10
    Turkey                                         3
    United States                                 14
    Hong Kong                                      3

Model Test User Model:
                                            Standard      Scaled
  Test Statistic                              54.100     169.061
  Degrees of freedom                              23          23
  P-value (Unknown)                               NA       0.000
  Scaling correction factor                                0.323
  Shift parameter                                          1.430
    simple second-order correction
  Test statistic for each group:
    Sweden                                    13.164      13.164
    United Kingdom                             0.044       0.044
    Germany                                    3.963       3.963
    China                                     20.178      20.178
    Argentina                                  0.596       0.596
    Australia                                  0.054       0.054
    Brazil                                     7.125       7.125
    Egypt                                      0.190       0.190
```

```
India                                                    54.867      54.867
Indonesia                                                 2.425       2.425
Israel                                                   21.580      21.580
Japan                                                     0.842       0.842
Kenya                                                     0.286       0.286
Mexico                                                    5.993       5.993
Nigeria                                                  14.838      14.838
Philippines                                               6.677       6.677
Poland                                                    5.935       5.935
South Africa                                              0.089       0.089
Spain                                                     1.359       1.359
Tanzania                                                  4.286       4.286
Turkey                                                    0.100       0.100
United States                                             4.344       4.344
Hong Kong                                                 0.127       0.127
```

```r
#### Residuals
residuals(fit.config)
```

```
$Sweden
$Sweden$type
[1] "raw"

$Sweden$cov
                 DEPRES INTERE CONTRO FEEL_A
DEPRESSED_Y2      0.000
INTEREST_Y2       0.000  0.000
CONTROL_WORRY_Y2 -0.002  0.011  0.000
FEEL_ANXIOUS_Y2   0.003 -0.011  0.000  0.000

$Sweden$mean
    DEPRESSED_Y2      INTEREST_Y2 CONTROL_WORRY_Y2  FEEL_ANXIOUS_Y2
               0                0                0                0

$Sweden$th
    DEPRESSED_Y2|t1     DEPRESSED_Y2|t2     DEPRESSED_Y2|t3      INTEREST_Y2|t1      INTERES
                  0                   0                   0                   0
 FEEL_ANXIOUS_Y2|t2  FEEL_ANXIOUS_Y2|t3
                  0                   0


$`United Kingdom`
```

```
$`United Kingdom`$type
[1] "raw"

$`United Kingdom`$cov
                 DEPRES INTERE CONTRO FEEL_A
DEPRESSED_Y2          0
INTEREST_Y2           0      0
CONTROL_WORRY_Y2      0      0      0
FEEL_ANXIOUS_Y2       0      0      0      0

$`United Kingdom`$mean
    DEPRESSED_Y2      INTEREST_Y2 CONTROL_WORRY_Y2  FEEL_ANXIOUS_Y2
               0                0                0                0

$`United Kingdom`$th
    DEPRESSED_Y2|t1     DEPRESSED_Y2|t2     DEPRESSED_Y2|t3      INTEREST_Y2|t1      INTEREST
                  0                   0                   0                   0
 FEEL_ANXIOUS_Y2|t2  FEEL_ANXIOUS_Y2|t3
                  0                   0


$Germany
$Germany$type
[1] "raw"

$Germany$cov
                 DEPRES INTERE CONTRO FEEL_A
DEPRESSED_Y2      0.000
INTEREST_Y2       0.000  0.000
CONTROL_WORRY_Y2 -0.004  0.006  0.000
FEEL_ANXIOUS_Y2   0.005 -0.007  0.000  0.000

$Germany$mean
    DEPRESSED_Y2      INTEREST_Y2 CONTROL_WORRY_Y2  FEEL_ANXIOUS_Y2
               0                0                0                0

$Germany$th
    DEPRESSED_Y2|t1     DEPRESSED_Y2|t2     DEPRESSED_Y2|t3      INTEREST_Y2|t1      INTEREST
                  0                   0                   0                   0
 FEEL_ANXIOUS_Y2|t2  FEEL_ANXIOUS_Y2|t3
                  0                   0
```

```
$China
$China$type
[1] "raw"

$China$cov
                 DEPRES INTERE CONTRO FEEL_A
DEPRESSED_Y2      0.000
INTEREST_Y2       0.000  0.000
CONTROL_WORRY_Y2 -0.008  0.020  0.000
FEEL_ANXIOUS_Y2   0.008 -0.019  0.000  0.000

$China$mean
    DEPRESSED_Y2      INTEREST_Y2 CONTROL_WORRY_Y2  FEEL_ANXIOUS_Y2
               0                0                0                0

$China$th
    DEPRESSED_Y2|t1     DEPRESSED_Y2|t2     DEPRESSED_Y2|t3      INTEREST_Y2|t1      INTEREST
                  0                   0                   0                   0
 FEEL_ANXIOUS_Y2|t2  FEEL_ANXIOUS_Y2|t3
                  0                   0


$Argentina
$Argentina$type
[1] "raw"

$Argentina$cov
                 DEPRES INTERE CONTRO FEEL_A
DEPRESSED_Y2      0.000
INTEREST_Y2       0.000  0.000
CONTROL_WORRY_Y2  0.002 -0.004  0.000
FEEL_ANXIOUS_Y2  -0.002  0.003  0.000  0.000

$Argentina$mean
    DEPRESSED_Y2      INTEREST_Y2 CONTROL_WORRY_Y2  FEEL_ANXIOUS_Y2
               0                0                0                0

$Argentina$th
    DEPRESSED_Y2|t1     DEPRESSED_Y2|t2     DEPRESSED_Y2|t3      INTEREST_Y2|t1      INTEREST
                  0                   0                   0                   0
 FEEL_ANXIOUS_Y2|t2  FEEL_ANXIOUS_Y2|t3
                  0                   0
```

```
$Australia
$Australia$type
[1] "raw"

$Australia$cov
                 DEPRES INTERE CONTRO FEEL_A
DEPRESSED_Y2      0.000
INTEREST_Y2       0.000  0.000
CONTROL_WORRY_Y2  0.000 -0.001  0.000
FEEL_ANXIOUS_Y2   0.000  0.001  0.000  0.000

$Australia$mean
    DEPRESSED_Y2      INTEREST_Y2 CONTROL_WORRY_Y2  FEEL_ANXIOUS_Y2
               0                0                0                0

$Australia$th
    DEPRESSED_Y2|t1     DEPRESSED_Y2|t2     DEPRESSED_Y2|t3      INTEREST_Y2|t1      INTEREST
                  0                   0                   0                   0
 FEEL_ANXIOUS_Y2|t2  FEEL_ANXIOUS_Y2|t3
                  0                   0


$Brazil
$Brazil$type
[1] "raw"

$Brazil$cov
                 DEPRES INTERE CONTRO FEEL_A
DEPRESSED_Y2      0.000
INTEREST_Y2       0.000  0.000
CONTROL_WORRY_Y2 -0.006  0.010  0.000
FEEL_ANXIOUS_Y2   0.005 -0.009  0.000  0.000

$Brazil$mean
    DEPRESSED_Y2      INTEREST_Y2 CONTROL_WORRY_Y2  FEEL_ANXIOUS_Y2
               0                0                0                0

$Brazil$th
    DEPRESSED_Y2|t1     DEPRESSED_Y2|t2     DEPRESSED_Y2|t3      INTEREST_Y2|t1      INTEREST
                  0                   0                   0                   0
 FEEL_ANXIOUS_Y2|t2  FEEL_ANXIOUS_Y2|t3
                  0                   0
```

```
$Egypt
$Egypt$type
[1] "raw"

$Egypt$cov
                 DEPRES INTERE CONTRO FEEL_A
DEPRESSED_Y2      0.000
INTEREST_Y2       0.000  0.000
CONTROL_WORRY_Y2  0.001 -0.002  0.000
FEEL_ANXIOUS_Y2  -0.001  0.002  0.000  0.000

$Egypt$mean
    DEPRESSED_Y2      INTEREST_Y2 CONTROL_WORRY_Y2  FEEL_ANXIOUS_Y2
               0                0                0                0

$Egypt$th
    DEPRESSED_Y2|t1     DEPRESSED_Y2|t2     DEPRESSED_Y2|t3      INTEREST_Y2|t1      INTEREST
                  0                   0                   0                   0
 FEEL_ANXIOUS_Y2|t2  FEEL_ANXIOUS_Y2|t3
                  0                   0


$India
$India$type
[1] "raw"

$India$cov
                 DEPRES INTERE CONTRO FEEL_A
DEPRESSED_Y2      0.000
INTEREST_Y2       0.000  0.000
CONTROL_WORRY_Y2 -0.012  0.038  0.000
FEEL_ANXIOUS_Y2   0.008 -0.035  0.000  0.000

$India$mean
    DEPRESSED_Y2      INTEREST_Y2 CONTROL_WORRY_Y2  FEEL_ANXIOUS_Y2
               0                0                0                0

$India$th
    DEPRESSED_Y2|t1     DEPRESSED_Y2|t2     DEPRESSED_Y2|t3      INTEREST_Y2|t1      INTEREST
                  0                   0                   0                   0
 FEEL_ANXIOUS_Y2|t2  FEEL_ANXIOUS_Y2|t3
```

```
                 0                   0


$Indonesia
$Indonesia$type
[1] "raw"

$Indonesia$cov
                 DEPRES INTERE CONTRO FEEL_A
DEPRESSED_Y2      0.000
INTEREST_Y2       0.000  0.000
CONTROL_WORRY_Y2 -0.004  0.010  0.000
FEEL_ANXIOUS_Y2   0.003 -0.008  0.000  0.000

$Indonesia$mean
    DEPRESSED_Y2      INTEREST_Y2 CONTROL_WORRY_Y2  FEEL_ANXIOUS_Y2
               0                0                0                0

$Indonesia$th
    DEPRESSED_Y2|t1     DEPRESSED_Y2|t2     DEPRESSED_Y2|t3      INTEREST_Y2|t1      INTEREST
                  0                   0                   0                   0
 FEEL_ANXIOUS_Y2|t2  FEEL_ANXIOUS_Y2|t3
                  0                   0


$Israel
$Israel$type
[1] "raw"

$Israel$cov
                 DEPRES INTERE CONTRO FEEL_A
DEPRESSED_Y2      0.000
INTEREST_Y2       0.000  0.000
CONTROL_WORRY_Y2  0.015 -0.034  0.000
FEEL_ANXIOUS_Y2  -0.007  0.016  0.000  0.000

$Israel$mean
    DEPRESSED_Y2      INTEREST_Y2 CONTROL_WORRY_Y2  FEEL_ANXIOUS_Y2
               0                0                0                0

$Israel$th
    DEPRESSED_Y2|t1     DEPRESSED_Y2|t2     DEPRESSED_Y2|t3      INTEREST_Y2|t1      INTEREST
                  0                   0                   0                   0
```

```
 FEEL_ANXIOUS_Y2|t2  FEEL_ANXIOUS_Y2|t3
                 0                   0


$Japan
$Japan$type
[1] "raw"

$Japan$cov
                 DEPRES INTERE CONTRO FEEL_A
DEPRESSED_Y2      0.000
INTEREST_Y2       0.000  0.000
CONTROL_WORRY_Y2  0.001 -0.002  0.000
FEEL_ANXIOUS_Y2   0.000  0.001  0.000  0.000

$Japan$mean
    DEPRESSED_Y2      INTEREST_Y2 CONTROL_WORRY_Y2  FEEL_ANXIOUS_Y2
               0                0                0                0

$Japan$th
    DEPRESSED_Y2|t1     DEPRESSED_Y2|t2     DEPRESSED_Y2|t3      INTEREST_Y2|t1      INTEREST
                 0                   0                   0                   0
 FEEL_ANXIOUS_Y2|t2  FEEL_ANXIOUS_Y2|t3
                 0                   0


$Kenya
$Kenya$type
[1] "raw"

$Kenya$cov
                 DEPRES INTERE CONTRO FEEL_A
DEPRESSED_Y2      0.000
INTEREST_Y2       0.000  0.000
CONTROL_WORRY_Y2 -0.001  0.002  0.000
FEEL_ANXIOUS_Y2   0.001 -0.001  0.000  0.000

$Kenya$mean
    DEPRESSED_Y2      INTEREST_Y2 CONTROL_WORRY_Y2  FEEL_ANXIOUS_Y2
               0                0                0                0

$Kenya$th
    DEPRESSED_Y2|t1     DEPRESSED_Y2|t2     DEPRESSED_Y2|t3      INTEREST_Y2|t1      INTEREST
```

```
                0                   0                   0                   0
 FEEL_ANXIOUS_Y2|t2  FEEL_ANXIOUS_Y2|t3
                0                   0


$Mexico
$Mexico$type
[1] "raw"

$Mexico$cov
                 DEPRES INTERE CONTRO FEEL_A
DEPRESSED_Y2      0.000
INTEREST_Y2       0.000  0.000
CONTROL_WORRY_Y2 -0.008  0.012  0.000
FEEL_ANXIOUS_Y2   0.005 -0.011  0.000  0.000

$Mexico$mean
    DEPRESSED_Y2      INTEREST_Y2 CONTROL_WORRY_Y2  FEEL_ANXIOUS_Y2
               0                0                0                0

$Mexico$th
    DEPRESSED_Y2|t1     DEPRESSED_Y2|t2     DEPRESSED_Y2|t3      INTEREST_Y2|t1      INTEREST
                0                   0                   0                   0
 FEEL_ANXIOUS_Y2|t2  FEEL_ANXIOUS_Y2|t3
                0                   0


$Nigeria
$Nigeria$type
[1] "raw"

$Nigeria$cov
                 DEPRES INTERE CONTRO FEEL_A
DEPRESSED_Y2      0.000
INTEREST_Y2       0.000  0.000
CONTROL_WORRY_Y2 -0.013  0.021  0.000
FEEL_ANXIOUS_Y2   0.012 -0.022  0.000  0.000

$Nigeria$mean
    DEPRESSED_Y2      INTEREST_Y2 CONTROL_WORRY_Y2  FEEL_ANXIOUS_Y2
               0                0                0                0

$Nigeria$th
```

```
    DEPRESSED_Y2|t1     DEPRESSED_Y2|t2     DEPRESSED_Y2|t3      INTEREST_Y2|t1      INTEREST
                  0                   0                   0                   0
 FEEL_ANXIOUS_Y2|t2  FEEL_ANXIOUS_Y2|t3
                  0                   0


$Philippines
$Philippines$type
[1] "raw"

$Philippines$cov
                 DEPRES INTERE CONTRO FEEL_A
DEPRESSED_Y2      0.000
INTEREST_Y2       0.000  0.000
CONTROL_WORRY_Y2 -0.011  0.015  0.000
FEEL_ANXIOUS_Y2   0.008 -0.015  0.000  0.000

$Philippines$mean
    DEPRESSED_Y2      INTEREST_Y2 CONTROL_WORRY_Y2  FEEL_ANXIOUS_Y2
               0                0                0                0

$Philippines$th
    DEPRESSED_Y2|t1     DEPRESSED_Y2|t2     DEPRESSED_Y2|t3      INTEREST_Y2|t1      INTEREST
                  0                   0                   0                   0
 FEEL_ANXIOUS_Y2|t2  FEEL_ANXIOUS_Y2|t3
                  0                   0


$Poland
$Poland$type
[1] "raw"

$Poland$cov
                 DEPRES INTERE CONTRO FEEL_A
DEPRESSED_Y2      0.000
INTEREST_Y2       0.000  0.000
CONTROL_WORRY_Y2  0.005 -0.007  0.000
FEEL_ANXIOUS_Y2  -0.008  0.008  0.000  0.000

$Poland$mean
    DEPRESSED_Y2      INTEREST_Y2 CONTROL_WORRY_Y2  FEEL_ANXIOUS_Y2
               0                0                0                0
```

```
$Poland$th
    DEPRESSED_Y2|t1     DEPRESSED_Y2|t2     DEPRESSED_Y2|t3      INTEREST_Y2|t1      INTEREST
                  0                   0                   0                   0
 FEEL_ANXIOUS_Y2|t2  FEEL_ANXIOUS_Y2|t3
                  0                   0


$`South Africa`
$`South Africa`$type
[1] "raw"

$`South Africa`$cov
                 DEPRES INTERE CONTRO FEEL_A
DEPRESSED_Y2      0.000
INTEREST_Y2       0.000  0.000
CONTROL_WORRY_Y2  0.002 -0.003  0.000
FEEL_ANXIOUS_Y2  -0.002  0.003  0.000  0.000

$`South Africa`$mean
    DEPRESSED_Y2      INTEREST_Y2 CONTROL_WORRY_Y2  FEEL_ANXIOUS_Y2
               0                0                0                0

$`South Africa`$th
    DEPRESSED_Y2|t1     DEPRESSED_Y2|t2     DEPRESSED_Y2|t3      INTEREST_Y2|t1      INTEREST
                  0                   0                   0                   0
 FEEL_ANXIOUS_Y2|t2  FEEL_ANXIOUS_Y2|t3
                  0                   0


$Spain
$Spain$type
[1] "raw"

$Spain$cov
                 DEPRES INTERE CONTRO FEEL_A
DEPRESSED_Y2      0.000
INTEREST_Y2       0.000  0.000
CONTROL_WORRY_Y2 -0.003  0.005  0.000
FEEL_ANXIOUS_Y2   0.003 -0.005  0.000  0.000

$Spain$mean
    DEPRESSED_Y2      INTEREST_Y2 CONTROL_WORRY_Y2  FEEL_ANXIOUS_Y2
               0                0                0                0
```

```
$Spain$th
    DEPRESSED_Y2|t1     DEPRESSED_Y2|t2     DEPRESSED_Y2|t3      INTEREST_Y2|t1      INTEREST
                  0                   0                   0                   0
 FEEL_ANXIOUS_Y2|t2  FEEL_ANXIOUS_Y2|t3
                  0                   0


$Tanzania
$Tanzania$type
[1] "raw"

$Tanzania$cov
                 DEPRES INTERE CONTRO FEEL_A
DEPRESSED_Y2      0.000
INTEREST_Y2       0.000  0.000
CONTROL_WORRY_Y2 -0.005  0.011  0.000
FEEL_ANXIOUS_Y2   0.004 -0.010  0.000  0.000

$Tanzania$mean
    DEPRESSED_Y2      INTEREST_Y2 CONTROL_WORRY_Y2  FEEL_ANXIOUS_Y2
               0                0                0                0

$Tanzania$th
    DEPRESSED_Y2|t1     DEPRESSED_Y2|t2     DEPRESSED_Y2|t3      INTEREST_Y2|t1      INTEREST
                  0                   0                   0                   0
 FEEL_ANXIOUS_Y2|t2  FEEL_ANXIOUS_Y2|t3
                  0                   0


$Turkey
$Turkey$type
[1] "raw"

$Turkey$cov
                 DEPRES INTERE CONTRO FEEL_A
DEPRESSED_Y2      0.000
INTEREST_Y2       0.000  0.000
CONTROL_WORRY_Y2  0.002 -0.004  0.000
FEEL_ANXIOUS_Y2  -0.001  0.003  0.000  0.000

$Turkey$mean
    DEPRESSED_Y2      INTEREST_Y2 CONTROL_WORRY_Y2  FEEL_ANXIOUS_Y2
```

```
             0                 0                 0                 0

$Turkey$th
    DEPRESSED_Y2|t1     DEPRESSED_Y2|t2     DEPRESSED_Y2|t3      INTEREST_Y2|t1      INTEREST
                  0                   0                   0                   0
 FEEL_ANXIOUS_Y2|t2  FEEL_ANXIOUS_Y2|t3
                  0                   0


$`United States`
$`United States`$type
[1] "raw"

$`United States`$cov
                 DEPRES INTERE CONTRO FEEL_A
DEPRESSED_Y2      0.000
INTEREST_Y2       0.000  0.000
CONTROL_WORRY_Y2 -0.002  0.003  0.000
FEEL_ANXIOUS_Y2   0.002 -0.003  0.000  0.000

$`United States`$mean
    DEPRESSED_Y2      INTEREST_Y2 CONTROL_WORRY_Y2  FEEL_ANXIOUS_Y2
             0                 0                 0                 0

$`United States`$th
    DEPRESSED_Y2|t1     DEPRESSED_Y2|t2     DEPRESSED_Y2|t3      INTEREST_Y2|t1      INTEREST
                  0                   0                   0                   0
 FEEL_ANXIOUS_Y2|t2  FEEL_ANXIOUS_Y2|t3
                  0                   0


$`Hong Kong`
$`Hong Kong`$type
[1] "raw"

$`Hong Kong`$cov
                 DEPRES INTERE CONTRO FEEL_A
DEPRESSED_Y2      0.000
INTEREST_Y2       0.000  0.000
CONTROL_WORRY_Y2 -0.001  0.004  0.000
FEEL_ANXIOUS_Y2   0.001 -0.004  0.000  0.000

$`Hong Kong`$mean
```

```
   DEPRESSED_Y2      INTEREST_Y2 CONTROL_WORRY_Y2  FEEL_ANXIOUS_Y2
              0                0                0                0

$`Hong Kong`$th
   DEPRESSED_Y2|t1     DEPRESSED_Y2|t2     DEPRESSED_Y2|t3      INTEREST_Y2|t1      INTERES
                 0                   0                   0                   0
 FEEL_ANXIOUS_Y2|t2  FEEL_ANXIOUS_Y2|t3
                 0                   0
```

Based on the residuals, we found unexplained correlation between items Interest and Feel Anxious in China, India, Indonesia, and Israel, which is not concerning.

4.2.3 Threshold Invariance Model

Identification constraints:

- LRV distribution for each observed item is identified based on fixed thresholds. Comparisons in the item location and scale not possible.
- LRV residual variance freely estimated (except for group 1, Sweden, for identification fixed to 1.0)
- LRV location freely estimated (except for group 1, Sweden, for identification fixed to 0.0)
- loadings freely estimated (comparable across countries)
- thresholds freely estimated but constrained to equal across countries
- factor means fixed to 0 in all countries (not comparable across countries)
- factor variances fixed to 1 in all countries (not comparable across countries)

```
mod.threshold <- semTools::measEq.syntax(
  configural.model = mod
  , data = df.cc
  , group = "COUNTRY"
  , parameterization = "theta"
  , ID.fac = "std.lv"
  , ID.cat = "Wu.Estabrook.2016"
  , ordered = TRUE
  , group.equal = "thresholds"
)

summary(mod.threshold)
```

```
This lavaan model syntax specifies a CFA with 4 manifest indicators (4 of which are ordinal)

To identify the location and scale of each common factor, the factor means and variances wer

The location and scale of each latent item-response underlying 4 ordinal indicators were ide

    https://doi.org/10.1007/s11336-016-9506-0

Pattern matrix indicating num(eric), ord(ered), and lat(ent) indicators per factor:

                 dep anx
DEPRESSED_Y2     ord
INTEREST_Y2      ord
CONTROL_WORRY_Y2     ord
FEEL_ANXIOUS_Y2      ord
```

```
The following types of parameter were constrained to equality across groups:

    thresholds
```

```r
cat(as.character(mod.threshold))
```

```
## LOADINGS:

dep =~ c(NA, NA, NA, NA, NA, NA, NA, NA, NA, NA, NA, NA, NA, NA, NA, NA, NA, NA, NA, NA, NA,
dep =~ c(NA, NA, NA, NA, NA, NA, NA, NA, NA, NA, NA, NA, NA, NA, NA, NA, NA, NA, NA, NA, NA,
anx =~ c(NA, NA, NA, NA, NA, NA, NA, NA, NA, NA, NA, NA, NA, NA, NA, NA, NA, NA, NA, NA, NA,
anx =~ c(NA, NA, NA, NA, NA, NA, NA, NA, NA, NA, NA, NA, NA, NA, NA, NA, NA, NA, NA, NA, NA,

## THRESHOLDS:

DEPRESSED_Y2 | c(NA, NA, NA, NA, NA, NA, NA, NA, NA, NA, NA, NA, NA, NA, NA, NA, NA, NA, NA,
DEPRESSED_Y2 | c(NA, NA, NA, NA, NA, NA, NA, NA, NA, NA, NA, NA, NA, NA, NA, NA, NA, NA, NA,
DEPRESSED_Y2 | c(NA, NA, NA, NA, NA, NA, NA, NA, NA, NA, NA, NA, NA, NA, NA, NA, NA, NA, NA,
INTEREST_Y2 | c(NA, NA, NA, NA, NA, NA, NA, NA, NA, NA, NA, NA, NA, NA, NA, NA, NA, NA, NA, N
INTEREST_Y2 | c(NA, NA, NA, NA, NA, NA, NA, NA, NA, NA, NA, NA, NA, NA, NA, NA, NA, NA, NA, N
INTEREST_Y2 | c(NA, NA, NA, NA, NA, NA, NA, NA, NA, NA, NA, NA, NA, NA, NA, NA, NA, NA, NA, N
CONTROL_WORRY_Y2 | c(NA, NA, NA, NA, NA, NA, NA, NA, NA, NA, NA, NA, NA, NA, NA, NA, NA, NA,
CONTROL_WORRY_Y2 | c(NA, NA, NA, NA, NA, NA, NA, NA, NA, NA, NA, NA, NA, NA, NA, NA, NA, NA,
CONTROL_WORRY_Y2 | c(NA, NA, NA, NA, NA, NA, NA, NA, NA, NA, NA, NA, NA, NA, NA, NA, NA, NA,
FEEL_ANXIOUS_Y2 | c(NA, NA, NA, NA, NA, NA, NA, NA, NA, NA, NA, NA, NA, NA, NA, NA, NA, NA, N
FEEL_ANXIOUS_Y2 | c(NA, NA, NA, NA, NA, NA, NA, NA, NA, NA, NA, NA, NA, NA, NA, NA, NA, NA, N
FEEL_ANXIOUS_Y2 | c(NA, NA, NA, NA, NA, NA, NA, NA, NA, NA, NA, NA, NA, NA, NA, NA, NA, NA, N

## INTERCEPTS:

DEPRESSED_Y2 ~ c(0, NA, NA, NA, NA, NA, NA, NA, NA, NA, NA, NA, NA, NA, NA, NA, NA, NA, NA, N
INTEREST_Y2 ~ c(0, NA, NA, NA, NA, NA, NA, NA, NA, NA, NA, NA, NA, NA, NA, NA, NA, NA, NA, NA
CONTROL_WORRY_Y2 ~ c(0, NA, NA, NA, NA, NA, NA, NA, NA, NA, NA, NA, NA, NA, NA, NA, NA, N
FEEL_ANXIOUS_Y2 ~ c(0, NA, NA, NA, NA, NA, NA, NA, NA, NA, NA, NA, NA, NA, NA, NA, NA, NA

## UNIQUE-FACTOR VARIANCES:

DEPRESSED_Y2 ~~ c(1, NA, NA, NA, NA, NA, NA, NA, NA, NA, NA, NA, NA, NA, NA, NA, NA, NA, NA,
INTEREST_Y2 ~~ c(1, NA, NA, NA, NA, NA, NA, NA, NA, NA, NA, NA, NA, NA, NA, NA, NA, NA, NA, N
CONTROL_WORRY_Y2 ~~ c(1, NA, NA, NA, NA, NA, NA, NA, NA, NA, NA, NA, NA, NA, NA, NA, NA,
FEEL_ANXIOUS_Y2 ~~ c(1, NA, NA, NA, NA, NA, NA, NA, NA, NA, NA, NA, NA, NA, NA, NA, NA, N
```

```
## LATENT MEANS/INTERCEPTS:

dep ~ c(0, 0, 0, 0, 0, 0, 0, 0, 0, 0, 0, 0, 0, 0, 0, 0, 0, 0, 0, 0, 0, 0, 0)*1 + c(alpha.1.g
anx ~ c(0, 0, 0, 0, 0, 0, 0, 0, 0, 0, 0, 0, 0, 0, 0, 0, 0, 0, 0, 0, 0, 0, 0)*1 + c(alpha.2.g

## COMMON-FACTOR VARIANCES:

dep ~~ c(1, 1, 1, 1, 1, 1, 1, 1, 1, 1, 1, 1, 1, 1, 1, 1, 1, 1, 1, 1, 1, 1, 1)*dep + c(psi.1_
anx ~~ c(1, 1, 1, 1, 1, 1, 1, 1, 1, 1, 1, 1, 1, 1, 1, 1, 1, 1, 1, 1, 1, 1, 1)*anx + c(psi.2_

## COMMON-FACTOR COVARIANCES:

dep ~~ c(NA, NA, NA, NA, NA, NA, NA, NA, NA, NA, NA, NA, NA, NA, NA, NA, NA, NA, NA, NA, NA,
```

```
fit.threshold <- cfa(
  model = as.character(mod.threshold)
  , data = df.cc
  , group = "COUNTRY"
  , ordered = TRUE
  , parameterization = "theta"
  , missing = "pairwise"
)

fit.threshold
```

```
lavaan 0.6-21 ended normally after 1994 iterations

  Estimator                                         DWLS
  Optimization method                             NLMINB
  Number of model parameters                         567
  Number of equality constraints                     264

  Number of observations per group:               Used       Total
    Sweden                                       11607       15068
    United Kingdom                                3619        5368
    Germany                                       5528        9506
    China                                         4544        5022
    Argentina                                     2928        6724
    Australia                                     2581        3844
    Brazil                                        4274       13203
    Egypt                                         3040        4729
```

```
    India                                         6372       12765
    Indonesia                                     2681        6992
    Israel                                        2489        3669
    Japan                                        13968       20543
    Kenya                                         7698       11389
    Mexico                                        2278        5776
    Nigeria                                       3146        6827
    Philippines                                   2682        5292
    Poland                                        6478       10389
    South Africa                                   978        2651
    Spain                                         2923        6290
    Tanzania                                      5583        9075
    Turkey                                         499        1473
    United States                                32239       38312
    Hong Kong                                      707        3012
  Number of missing patterns per group:
    Sweden                                          13
    United Kingdom                                   6
    Germany                                          8
    China                                            4
    Argentina                                       10
    Australia                                        7
    Brazil                                           8
    Egypt                                            5
    India                                           13
    Indonesia                                        6
    Israel                                           9
    Japan                                            9
    Kenya                                            6
    Mexico                                           8
    Nigeria                                          9
    Philippines                                      4
    Poland                                           9
    South Africa                                     4
    Spain                                            7
    Tanzania                                        10
    Turkey                                           3
    United States                                   14
    Hong Kong                                        3

Model Test User Model:
                                              Standard      Scaled
  Test Statistic                              2256.698    5592.781
```

```
  Degrees of freedom                                   111         111
  P-value (Unknown)                                     NA       0.000
  Scaling correction factor                                      0.404
  Shift parameter                                               11.291
    simple second-order correction
  Test statistic for each group:
    Sweden                                         140.843     140.843
    United Kingdom                                  11.747      11.747
    Germany                                         14.303      14.303
    China                                           95.741      95.741
    Argentina                                       40.549      40.549
    Australia                                       23.648      23.648
    Brazil                                          13.470      13.470
    Egypt                                          175.749     175.749
    India                                          882.097     882.097
    Indonesia                                      232.105     232.105
    Israel                                          41.380      41.380
    Japan                                          115.548     115.548
    Kenya                                          764.211     764.211
    Mexico                                          19.578      19.578
    Nigeria                                         86.640      86.640
    Philippines                                    222.929     222.929
    Poland                                          14.934      14.934
    South Africa                                     5.281       5.281
    Spain                                           32.349      32.349
    Tanzania                                      2384.784    2384.784
    Turkey                                           8.970       8.970
    United States                                  239.600     239.600
    Hong Kong                                       26.325      26.325
```

```
#### Residuals
residuals(fit.threshold)
```

```
$Sweden
$Sweden$type
[1] "raw"

$Sweden$cov
                 DEPRES INTERE CONTRO FEEL_A
DEPRESSED_Y2      0.000
INTEREST_Y2       0.000  0.000
CONTROL_WORRY_Y2 -0.003  0.011  0.000
```

```
FEEL_ANXIOUS_Y2   0.003 -0.011  0.000  0.000

$Sweden$mean
    DEPRESSED_Y2      INTEREST_Y2 CONTROL_WORRY_Y2  FEEL_ANXIOUS_Y2
               0                0                0                0

$Sweden$th
    DEPRESSED_Y2|t1     DEPRESSED_Y2|t2     DEPRESSED_Y2|t3      INTEREST_Y2|t1      INTEREST
             -0.011               0.044              -0.043               0.017
0.050               0.053               0.001              -0.004               0.004
0.009
 FEEL_ANXIOUS_Y2|t2  FEEL_ANXIOUS_Y2|t3
              0.035              -0.037


$`United Kingdom`
$`United Kingdom`$type
[1] "raw"

$`United Kingdom`$cov
                 DEPRES INTERE CONTRO FEEL_A
DEPRESSED_Y2          0
INTEREST_Y2           0      0
CONTROL_WORRY_Y2      0      0      0
FEEL_ANXIOUS_Y2       0      0      0      0

$`United Kingdom`$mean
    DEPRESSED_Y2      INTEREST_Y2 CONTROL_WORRY_Y2  FEEL_ANXIOUS_Y2
               0                0                0                0

$`United Kingdom`$th
    DEPRESSED_Y2|t1     DEPRESSED_Y2|t2     DEPRESSED_Y2|t3      INTEREST_Y2|t1      INTEREST
              0.006              -0.021               0.022               0.009
0.026               0.025               0.002              -0.008               0.008
 FEEL_ANXIOUS_Y2|t2  FEEL_ANXIOUS_Y2|t3
             -0.018               0.018


$Germany
$Germany$type
[1] "raw"

$Germany$cov
```

```
                 DEPRES INTERE CONTRO FEEL_A
DEPRESSED_Y2      0.000
INTEREST_Y2       0.000  0.000
CONTROL_WORRY_Y2 -0.004  0.006  0.000
FEEL_ANXIOUS_Y2   0.005 -0.007  0.000  0.000

$Germany$mean
    DEPRESSED_Y2      INTEREST_Y2 CONTROL_WORRY_Y2  FEEL_ANXIOUS_Y2
               0                0                0                0

$Germany$th
    DEPRESSED_Y2|t1     DEPRESSED_Y2|t2     DEPRESSED_Y2|t3      INTEREST_Y2|t1      INTEREST
             -0.006               0.025              -0.027                   -
0.005              0.019               -0.021              -0.002               0.008
0.008             -0.001
 FEEL_ANXIOUS_Y2|t2  FEEL_ANXIOUS_Y2|t3
              0.003              -0.003


$China
$China$type
[1] "raw"

$China$cov
                 DEPRES INTERE CONTRO FEEL_A
DEPRESSED_Y2      0.000
INTEREST_Y2       0.000  0.000
CONTROL_WORRY_Y2 -0.008  0.020  0.000
FEEL_ANXIOUS_Y2   0.008 -0.019  0.000  0.000

$China$mean
    DEPRESSED_Y2      INTEREST_Y2 CONTROL_WORRY_Y2  FEEL_ANXIOUS_Y2
               0                0                0                0

$China$th
    DEPRESSED_Y2|t1     DEPRESSED_Y2|t2     DEPRESSED_Y2|t3      INTEREST_Y2|t1      INTEREST
             -0.001               0.006              -0.012               0.029
0.073              0.123                0.007              -0.030               0.052
 FEEL_ANXIOUS_Y2|t2  FEEL_ANXIOUS_Y2|t3
              0.001              -0.002


$Argentina
```

```
$Argentina$type
[1] "raw"

$Argentina$cov
                 DEPRES INTERE CONTRO FEEL_A
DEPRESSED_Y2      0.000
INTEREST_Y2       0.000  0.000
CONTROL_WORRY_Y2  0.002 -0.004  0.000
FEEL_ANXIOUS_Y2  -0.002  0.003  0.000  0.000

$Argentina$mean
    DEPRESSED_Y2      INTEREST_Y2 CONTROL_WORRY_Y2  FEEL_ANXIOUS_Y2
               0                0                0                0

$Argentina$th
    DEPRESSED_Y2|t1     DEPRESSED_Y2|t2     DEPRESSED_Y2|t3      INTEREST_Y2|t1      INTEREST
           -0.005               0.017              -0.016                   -
0.023              0.061              -0.053              -0.011              0.034
0.028             -0.011
 FEEL_ANXIOUS_Y2|t2  FEEL_ANXIOUS_Y2|t3
            0.032             -0.028


$Australia
$Australia$type
[1] "raw"

$Australia$cov
                 DEPRES INTERE CONTRO FEEL_A
DEPRESSED_Y2      0.000
INTEREST_Y2       0.000  0.000
CONTROL_WORRY_Y2  0.000 -0.001  0.000
FEEL_ANXIOUS_Y2   0.000  0.001  0.000  0.000

$Australia$mean
    DEPRESSED_Y2      INTEREST_Y2 CONTROL_WORRY_Y2  FEEL_ANXIOUS_Y2
               0                0                0                0

$Australia$th
    DEPRESSED_Y2|t1     DEPRESSED_Y2|t2     DEPRESSED_Y2|t3      INTEREST_Y2|t1      INTEREST
           -0.007               0.028              -0.031                   -
0.009              0.031              -0.034              -0.010              0.037
0.039             -0.011
```

```
 FEEL_ANXIOUS_Y2|t2  FEEL_ANXIOUS_Y2|t3
             0.043              -0.046


$Brazil
$Brazil$type
[1] "raw"

$Brazil$cov
                 DEPRES INTERE CONTRO FEEL_A
DEPRESSED_Y2      0.000
INTEREST_Y2       0.000  0.000
CONTROL_WORRY_Y2 -0.006  0.010  0.000
FEEL_ANXIOUS_Y2   0.005 -0.009  0.000  0.000

$Brazil$mean
    DEPRESSED_Y2      INTEREST_Y2 CONTROL_WORRY_Y2  FEEL_ANXIOUS_Y2
               0                0                0                0

$Brazil$th
    DEPRESSED_Y2|t1     DEPRESSED_Y2|t2     DEPRESSED_Y2|t3      INTEREST_Y2|t1      INTERES
             0.002              -0.005               0.005               0.005
0.013              0.010               -0.002               0.004               -
0.003             -0.009
 FEEL_ANXIOUS_Y2|t2  FEEL_ANXIOUS_Y2|t3
             0.023              -0.017


$Egypt
$Egypt$type
[1] "raw"

$Egypt$cov
                 DEPRES INTERE CONTRO FEEL_A
DEPRESSED_Y2      0.000
INTEREST_Y2       0.000  0.000
CONTROL_WORRY_Y2  0.001 -0.002  0.000
FEEL_ANXIOUS_Y2  -0.001  0.002  0.000  0.000

$Egypt$mean
    DEPRESSED_Y2      INTEREST_Y2 CONTROL_WORRY_Y2  FEEL_ANXIOUS_Y2
               0                0                0                0
```

```
$Egypt$th
    DEPRESSED_Y2|t1     DEPRESSED_Y2|t2     DEPRESSED_Y2|t3      INTEREST_Y2|t1      INTEREST
            -0.022               0.062              -0.049               -
0.039              0.101              -0.082              -0.021               0.053
0.041             -0.042
 FEEL_ANXIOUS_Y2|t2  FEEL_ANXIOUS_Y2|t3
             0.090              -0.063


$India
$India$type
[1] "raw"

$India$cov
                 DEPRES INTERE CONTRO FEEL_A
DEPRESSED_Y2      0.000
INTEREST_Y2       0.000  0.000
CONTROL_WORRY_Y2 -0.012  0.038  0.000
FEEL_ANXIOUS_Y2   0.008 -0.035  0.000  0.000

$India$mean
    DEPRESSED_Y2      INTEREST_Y2 CONTROL_WORRY_Y2  FEEL_ANXIOUS_Y2
               0                0                0                0

$India$th
    DEPRESSED_Y2|t1     DEPRESSED_Y2|t2     DEPRESSED_Y2|t3      INTEREST_Y2|t1      INTEREST
             0.051              -0.133               0.121               0.030
0.066              0.045               0.038              -0.098               0.078
 FEEL_ANXIOUS_Y2|t2  FEEL_ANXIOUS_Y2|t3
            -0.141               0.131


$Indonesia
$Indonesia$type
[1] "raw"

$Indonesia$cov
                 DEPRES INTERE CONTRO FEEL_A
DEPRESSED_Y2      0.000
INTEREST_Y2       0.000  0.000
CONTROL_WORRY_Y2 -0.004  0.010  0.000
FEEL_ANXIOUS_Y2   0.003 -0.008  0.000  0.000
```

```
$Indonesia$mean
    DEPRESSED_Y2      INTEREST_Y2 CONTROL_WORRY_Y2  FEEL_ANXIOUS_Y2
               0                0                0                0

$Indonesia$th
    DEPRESSED_Y2|t1     DEPRESSED_Y2|t2     DEPRESSED_Y2|t3      INTEREST_Y2|t1      INTEREST
             -0.028               0.117              -0.101                   -
0.046              0.145              -0.126              -0.018              0.069
0.060             -0.024
 FEEL_ANXIOUS_Y2|t2  FEEL_ANXIOUS_Y2|t3
              0.099              -0.088


$Israel
$Israel$type
[1] "raw"

$Israel$cov
                 DEPRES INTERE CONTRO FEEL_A
DEPRESSED_Y2      0.000
INTEREST_Y2       0.000  0.000
CONTROL_WORRY_Y2  0.015 -0.034  0.000
FEEL_ANXIOUS_Y2  -0.007  0.016  0.000  0.000

$Israel$mean
    DEPRESSED_Y2      INTEREST_Y2 CONTROL_WORRY_Y2  FEEL_ANXIOUS_Y2
               0                0                0                0

$Israel$th
    DEPRESSED_Y2|t1     DEPRESSED_Y2|t2     DEPRESSED_Y2|t3      INTEREST_Y2|t1      INTEREST
              0.003              -0.014               0.017               0.011
0.041              0.054               0.011              -0.043              0.061
 FEEL_ANXIOUS_Y2|t2  FEEL_ANXIOUS_Y2|t3
             -0.039               0.055


$Japan
$Japan$type
[1] "raw"

$Japan$cov
                 DEPRES INTERE CONTRO FEEL_A
DEPRESSED_Y2      0.000
```

```
INTEREST_Y2       0.000  0.000
CONTROL_WORRY_Y2  0.001 -0.002  0.000
FEEL_ANXIOUS_Y2   0.000  0.001  0.000  0.000

$Japan$mean
    DEPRESSED_Y2      INTEREST_Y2 CONTROL_WORRY_Y2  FEEL_ANXIOUS_Y2
               0                0                0                0

$Japan$th
    DEPRESSED_Y2|t1     DEPRESSED_Y2|t2     DEPRESSED_Y2|t3      INTEREST_Y2|t1      INTEREST
              0.004              -0.014               0.015               0.017
0.052               0.057               0.007               -0.028               0.031
 FEEL_ANXIOUS_Y2|t2  FEEL_ANXIOUS_Y2|t3
             -0.012               0.013


$Kenya
$Kenya$type
[1] "raw"

$Kenya$cov
                 DEPRES INTERE CONTRO FEEL_A
DEPRESSED_Y2      0.000
INTEREST_Y2       0.000  0.000
CONTROL_WORRY_Y2 -0.001  0.002  0.000
FEEL_ANXIOUS_Y2   0.001 -0.001  0.000  0.000

$Kenya$mean
    DEPRESSED_Y2      INTEREST_Y2 CONTROL_WORRY_Y2  FEEL_ANXIOUS_Y2
               0                0                0                0

$Kenya$th
    DEPRESSED_Y2|t1     DEPRESSED_Y2|t2     DEPRESSED_Y2|t3      INTEREST_Y2|t1      INTEREST
             -0.039               0.108              -0.085                   -
0.067               0.159              -0.117               -0.028               0.076
0.058              -0.014
 FEEL_ANXIOUS_Y2|t2  FEEL_ANXIOUS_Y2|t3
              0.041              -0.036


$Mexico
$Mexico$type
[1] "raw"
```

```
$Mexico$cov
                 DEPRES INTERE CONTRO FEEL_A
DEPRESSED_Y2      0.000
INTEREST_Y2       0.000  0.000
CONTROL_WORRY_Y2 -0.008  0.012  0.000
FEEL_ANXIOUS_Y2   0.005 -0.011  0.000  0.000

$Mexico$mean
    DEPRESSED_Y2      INTEREST_Y2 CONTROL_WORRY_Y2  FEEL_ANXIOUS_Y2
               0                0                0                0

$Mexico$th
    DEPRESSED_Y2|t1     DEPRESSED_Y2|t2     DEPRESSED_Y2|t3      INTEREST_Y2|t1      INTEREST
             -0.005               0.015              -0.014                   -
0.018              0.051               -0.045              -0.004              0.013
0.011              -0.001
 FEEL_ANXIOUS_Y2|t2  FEEL_ANXIOUS_Y2|t3
              0.003              -0.003


$Nigeria
$Nigeria$type
[1] "raw"

$Nigeria$cov
                 DEPRES INTERE CONTRO FEEL_A
DEPRESSED_Y2      0.000
INTEREST_Y2       0.000  0.000
CONTROL_WORRY_Y2 -0.013  0.021  0.000
FEEL_ANXIOUS_Y2   0.012 -0.022  0.000  0.000

$Nigeria$mean
    DEPRESSED_Y2      INTEREST_Y2 CONTROL_WORRY_Y2  FEEL_ANXIOUS_Y2
               0                0                0                0

$Nigeria$th
    DEPRESSED_Y2|t1     DEPRESSED_Y2|t2     DEPRESSED_Y2|t3      INTEREST_Y2|t1      INTEREST
              0.022              -0.054               0.048                   -
0.006              0.012               -0.008               0.000              0.001
0.001               0.028
 FEEL_ANXIOUS_Y2|t2  FEEL_ANXIOUS_Y2|t3
             -0.080               0.075
```

```
$Philippines
$Philippines$type
[1] "raw"

$Philippines$cov
                 DEPRES INTERE CONTRO FEEL_A
DEPRESSED_Y2      0.000
INTEREST_Y2       0.000  0.000
CONTROL_WORRY_Y2 -0.011  0.015  0.000
FEEL_ANXIOUS_Y2   0.008 -0.015  0.000  0.000

$Philippines$mean
    DEPRESSED_Y2      INTEREST_Y2 CONTROL_WORRY_Y2  FEEL_ANXIOUS_Y2
               0                0                0                0

$Philippines$th
    DEPRESSED_Y2|t1     DEPRESSED_Y2|t2     DEPRESSED_Y2|t3      INTEREST_Y2|t1      INTEREST
             0.039              -0.101               0.099               0.036
0.069               0.052               0.010               -0.021               0.014
 FEEL_ANXIOUS_Y2|t2  FEEL_ANXIOUS_Y2|t3
            -0.126               0.122


$Poland
$Poland$type
[1] "raw"

$Poland$cov
                 DEPRES INTERE CONTRO FEEL_A
DEPRESSED_Y2      0.000
INTEREST_Y2       0.000  0.000
CONTROL_WORRY_Y2  0.005 -0.007  0.000
FEEL_ANXIOUS_Y2  -0.008  0.008  0.000  0.000

$Poland$mean
    DEPRESSED_Y2      INTEREST_Y2 CONTROL_WORRY_Y2  FEEL_ANXIOUS_Y2
               0                0                0                0

$Poland$th
    DEPRESSED_Y2|t1     DEPRESSED_Y2|t2     DEPRESSED_Y2|t3      INTEREST_Y2|t1      INTEREST
            -0.002               0.010              -0.016               -
```

```
0.001               0.007               -0.012              -0.001              0.003
0.005              -0.005
 FEEL_ANXIOUS_Y2|t2  FEEL_ANXIOUS_Y2|t3
             0.030              -0.051


$`South Africa`
$`South Africa`$type
[1] "raw"

$`South Africa`$cov
                 DEPRES INTERE CONTRO FEEL_A
DEPRESSED_Y2      0.000
INTEREST_Y2       0.000  0.000
CONTROL_WORRY_Y2  0.002 -0.003  0.000
FEEL_ANXIOUS_Y2  -0.002  0.003  0.000  0.000

$`South Africa`$mean
    DEPRESSED_Y2      INTEREST_Y2 CONTROL_WORRY_Y2  FEEL_ANXIOUS_Y2
               0                0                0                0

$`South Africa`$th
    DEPRESSED_Y2|t1     DEPRESSED_Y2|t2     DEPRESSED_Y2|t3      INTEREST_Y2|t1      INTERES
          -0.005               0.012              -0.009                  -
0.019               0.044               -0.036               0.001                  -
0.002               0.002                0.004
 FEEL_ANXIOUS_Y2|t2  FEEL_ANXIOUS_Y2|t3
          -0.011               0.010


$Spain
$Spain$type
[1] "raw"

$Spain$cov
                 DEPRES INTERE CONTRO FEEL_A
DEPRESSED_Y2      0.000
INTEREST_Y2       0.000  0.000
CONTROL_WORRY_Y2 -0.003  0.005  0.000
FEEL_ANXIOUS_Y2   0.003 -0.005  0.000  0.000

$Spain$mean
    DEPRESSED_Y2      INTEREST_Y2 CONTROL_WORRY_Y2  FEEL_ANXIOUS_Y2
```

```
              0                0                0                0

$Spain$th
    DEPRESSED_Y2|t1     DEPRESSED_Y2|t2     DEPRESSED_Y2|t3      INTEREST_Y2|t1      INTEREST
             -0.010              0.030              -0.027                -
0.009              0.022              -0.022              0.003              -
0.008              0.007              -0.022
 FEEL_ANXIOUS_Y2|t2  FEEL_ANXIOUS_Y2|t3
              0.055              -0.047


$Tanzania
$Tanzania$type
[1] "raw"

$Tanzania$cov
                 DEPRES INTERE CONTRO FEEL_A
DEPRESSED_Y2      0.000
INTEREST_Y2       0.000  0.000
CONTROL_WORRY_Y2 -0.005  0.011  0.000
FEEL_ANXIOUS_Y2   0.004 -0.010  0.000  0.000

$Tanzania$mean
    DEPRESSED_Y2      INTEREST_Y2 CONTROL_WORRY_Y2  FEEL_ANXIOUS_Y2
              0                0                0                0

$Tanzania$th
    DEPRESSED_Y2|t1     DEPRESSED_Y2|t2     DEPRESSED_Y2|t3      INTEREST_Y2|t1      INTEREST
              0.086             -0.218               0.202               0.057
0.136              0.103               0.073              -0.199               0.205
 FEEL_ANXIOUS_Y2|t2  FEEL_ANXIOUS_Y2|t3
             -0.236               0.273


$Turkey
$Turkey$type
[1] "raw"

$Turkey$cov
                 DEPRES INTERE CONTRO FEEL_A
DEPRESSED_Y2      0.000
INTEREST_Y2       0.000  0.000
CONTROL_WORRY_Y2  0.003 -0.005  0.000
```

```
FEEL_ANXIOUS_Y2  -0.001  0.002  0.000  0.000

$Turkey$mean
    DEPRESSED_Y2      INTEREST_Y2 CONTROL_WORRY_Y2  FEEL_ANXIOUS_Y2
               0                0                0                0

$Turkey$th
    DEPRESSED_Y2|t1     DEPRESSED_Y2|t2     DEPRESSED_Y2|t3      INTEREST_Y2|t1      INTEREST
              0.020              -0.052               0.041               0.021
0.050               0.036               0.009              -0.026               0.021
 FEEL_ANXIOUS_Y2|t2  FEEL_ANXIOUS_Y2|t3
             -0.036               0.028


$`United States`
$`United States`$type
[1] "raw"

$`United States`$cov
                 DEPRES INTERE CONTRO FEEL_A
DEPRESSED_Y2      0.000
INTEREST_Y2       0.000  0.000
CONTROL_WORRY_Y2 -0.002  0.003  0.000
FEEL_ANXIOUS_Y2   0.002 -0.003  0.000  0.000

$`United States`$mean
    DEPRESSED_Y2      INTEREST_Y2 CONTROL_WORRY_Y2  FEEL_ANXIOUS_Y2
               0                0                0                0

$`United States`$th
    DEPRESSED_Y2|t1     DEPRESSED_Y2|t2     DEPRESSED_Y2|t3      INTEREST_Y2|t1      INTEREST
             -0.008               0.038              -0.045                   -
0.005               0.019              -0.024              -0.010               0.041
0.045              -0.007
 FEEL_ANXIOUS_Y2|t2  FEEL_ANXIOUS_Y2|t3
              0.033              -0.039


$`Hong Kong`
$`Hong Kong`$type
[1] "raw"

$`Hong Kong`$cov
```

```
                  DEPRES INTERE CONTRO FEEL_A
DEPRESSED_Y2       0.000
INTEREST_Y2        0.000  0.000
CONTROL_WORRY_Y2 -0.001  0.004  0.000
FEEL_ANXIOUS_Y2    0.001 -0.004  0.000  0.000

$`Hong Kong`$mean
    DEPRESSED_Y2      INTEREST_Y2 CONTROL_WORRY_Y2  FEEL_ANXIOUS_Y2
               0                0                0                0

$`Hong Kong`$th
    DEPRESSED_Y2|t1     DEPRESSED_Y2|t2     DEPRESSED_Y2|t3      INTEREST_Y2|t1      INTEREST
              0.018              -0.054               0.100               0.029
0.054               0.070               0.026              -0.086               0.164
 FEEL_ANXIOUS_Y2|t2  FEEL_ANXIOUS_Y2|t3
             -0.014               0.021
```

```
fit.res.thr0 <- lapply(residuals(fit.threshold), \(x) x$th)
fit.res.thr <- fit.res.thr0 |> bind_rows() |> as.data.frame()
rownames(fit.res.thr) <- paste0(names(fit.res.thr0), " (", 1:length(names(fit.res.thr0)), ")"
fit.res.thr |> select(contains("t3")) |> mutate(across(everything(), ~round(.,2)))
```

```
                   DEPRESSED_Y2|t3 INTEREST_Y2|t3 CONTROL_WORRY_Y2|t3 FEEL_ANXIOUS_Y2|t3
Sweden (1)                   -0.04           0.05                0.00                  -
0.04
United Kingdom (2)            0.02           0.03                0.01               0.02
Germany (3)                  -0.03          -0.02               -0.01               0.00
China (4)                    -0.01           0.12                0.05               0.00
Argentina (5)                -0.02          -0.05               -0.03                  -
0.03
Australia (6)                -0.03          -0.03               -0.04                  -
0.05
Brazil (7)                    0.00           0.01                0.00                  -
0.02
Egypt (8)                    -0.05          -0.08               -0.04                  -
0.06
India (9)                     0.12           0.04                0.08               0.13
Indonesia (10)               -0.10          -0.13               -0.06                  -
0.09
Israel (11)                   0.02           0.05                0.06               0.05
Japan (12)                    0.01           0.06                0.03               0.01
```

Kenya (13)	-0.09	-0.12	-0.06	-0.04
Mexico (14)	-0.01	-0.05	-0.01	0.00
Nigeria (15)	0.05	-0.01	0.00	0.08
Philippines (16)	0.10	0.05	0.01	0.12
Poland (17)	-0.02	-0.01	-0.01	-0.05
South Africa (18)	-0.01	-0.04	0.00	0.01
Spain (19)	-0.03	-0.02	0.01	-0.05
Tanzania (20)	0.20	0.10	0.20	0.27
Turkey (21)	0.04	0.04	0.02	0.03
United States (22)	-0.05	-0.02	-0.04	-0.04
Hong Kong (23)	0.10	0.07	0.16	0.02

Lack of fit, likely due to non-invariance in some pair of thresholds. Next, relax the threshold invariance model to only constrain the first two thresholds to equality and freely estimate the third in a "partial" invariance model in countries where the threshold residual is "large (>0.10)" for each item.

- DEPRESSED: India (9), Indonesia (10), Philippines (16), Tanzania (20), and Hong Kong (23)
- INTEREST: China (4), Indonesia (10), Kenya (13), Tanzania (20)
- CONTROL_WORRY: Tanzania (20), and Hong Kong (23)
- FEEL_ANXIOUS: India (9), Philippines (16), Tanzania (20)

4.2.4 Threshold (Partial) Invariance Model

Identification constraints:

- LRV distribution for each observed item is identified based on fixed thresholds. Comparisons in the item location and scale not possible.
- LRV residual variance freely estimated (except for group 1, Sweden, for identification fixed to 1.0)
- LRV location freely estimated (except for group 1, Sweden, for identification fixed to 0.0)
- loadings freely estimated (comparable across countries)
- thresholds freely estimated but constrained to equal across countries except for the third threshold which is freely estimated in some countries
- factor means fixed to 0 in all countries (not comparable across countries)
- factor variances fixed to 1 in all countries (not comparable across countries)

```
mod.threshold.partial <- semTools::measEq.syntax(
  configural.model = mod
  , data = df.cc
  , group = "COUNTRY"
  , parameterization = "theta"
  , ID.fac = "std.lv"
  , ID.cat = "Wu.Estabrook.2016"
  , ordered = TRUE
  , group.equal = "thresholds"
  , group.partial = c(
    'DEPRESSED_Y2 | t3',
    'INTEREST_Y2 | t3',
    'CONTROL_WORRY_Y2 | t3',
    'FEEL_ANXIOUS_Y2 | t3'
  )
)

summary(mod.threshold.partial)
```

```
This lavaan model syntax specifies a CFA with 4 manifest indicators (4 of which are ordinal)

To identify the location and scale of each common factor, the factor means and variances were

The location and scale of each latent item-response underlying 4 ordinal indicators were ide

    https://doi.org/10.1007/s11336-016-9506-0
```

```
Pattern matrix indicating num(eric), ord(ered), and lat(ent) indicators per factor:

                 dep anx
DEPRESSED_Y2     ord
INTEREST_Y2      ord
CONTROL_WORRY_Y2     ord
FEEL_ANXIOUS_Y2      ord

The following types of parameter were constrained to equality across groups:

    thresholds, with the exception of:

                                 lhs op rhs
            row-1:       DEPRESSED_Y2  |  t3
            row-2:        INTEREST_Y2  |  t3
            row-3:    CONTROL_WORRY_Y2  |  t3
            row-4:     FEEL_ANXIOUS_Y2  |  t3
```

```
#cat(as.character(mod.threshold.partial))
## freeing on some thresholds requires modifying the model directly.
mod.threshold.partial <- "
## LOADINGS:

dep =~ c(NA, NA, NA, NA, NA, NA, NA, NA, NA, NA, NA, NA, NA, NA, NA, NA, NA, NA, NA, NA, NA,
dep =~ c(NA, NA, NA, NA, NA, NA, NA, NA, NA, NA, NA, NA, NA, NA, NA, NA, NA, NA, NA, NA, NA,
anx =~ c(NA, NA, NA, NA, NA, NA, NA, NA, NA, NA, NA, NA, NA, NA, NA, NA, NA, NA, NA, NA, NA,
anx =~ c(NA, NA, NA, NA, NA, NA, NA, NA, NA, NA, NA, NA, NA, NA, NA, NA, NA, NA, NA, NA, NA,

## THRESHOLDS:

DEPRESSED_Y2 | c(NA, NA, NA, NA, NA, NA, NA, NA, NA, NA, NA, NA, NA, NA, NA, NA, NA, NA, NA,
DEPRESSED_Y2 | c(NA, NA, NA, NA, NA, NA, NA, NA, NA, NA, NA, NA, NA, NA, NA, NA, NA, NA, NA,
DEPRESSED_Y2 | c(NA, NA, NA, NA, NA, NA, NA, NA, NA, NA, NA, NA, NA, NA, NA, NA, NA, NA, NA,
INTEREST_Y2 | c(NA, NA, NA, NA, NA, NA, NA, NA, NA, NA, NA, NA, NA, NA, NA, NA, NA, NA, NA,
INTEREST_Y2 | c(NA, NA, NA, NA, NA, NA, NA, NA, NA, NA, NA, NA, NA, NA, NA, NA, NA, NA, NA,
INTEREST_Y2 | c(NA, NA, NA, NA, NA, NA, NA, NA, NA, NA, NA, NA, NA, NA, NA, NA, NA, NA, NA,
CONTROL_WORRY_Y2 | c(NA, NA, NA, NA, NA, NA, NA, NA, NA, NA, NA, NA, NA, NA, NA, NA, NA, NA,
CONTROL_WORRY_Y2 | c(NA, NA, NA, NA, NA, NA, NA, NA, NA, NA, NA, NA, NA, NA, NA, NA, NA, NA,
CONTROL_WORRY_Y2 | c(NA, NA, NA, NA, NA, NA, NA, NA, NA, NA, NA, NA, NA, NA, NA, NA, NA, NA,
FEEL_ANXIOUS_Y2 | c(NA, NA, NA, NA, NA, NA, NA, NA, NA, NA, NA, NA, NA, NA, NA, NA, NA, NA,
FEEL_ANXIOUS_Y2 | c(NA, NA, NA, NA, NA, NA, NA, NA, NA, NA, NA, NA, NA, NA, NA, NA, NA, NA,
FEEL_ANXIOUS_Y2 | c(NA, NA, NA, NA, NA, NA, NA, NA, NA, NA, NA, NA, NA, NA, NA, NA, NA, NA,
```

```
## INTERCEPTS:

DEPRESSED_Y2 ~ c(0, NA, NA, NA, NA, NA, NA, NA, NA, NA, NA, NA, NA, NA, NA, NA, NA, NA, NA, N
INTEREST_Y2 ~ c(0, NA, NA, NA, NA, NA, NA, NA, NA, NA, NA, NA, NA, NA, NA, NA, NA, NA, NA, NA
CONTROL_WORRY_Y2 ~ c(0, NA, NA, NA, NA, NA, NA, NA, NA, NA, NA, NA, NA, NA, NA, NA, NA, NA, N
FEEL_ANXIOUS_Y2 ~ c(0, NA, NA, NA, NA, NA, NA, NA, NA, NA, NA, NA, NA, NA, NA, NA, NA, NA, NA

## UNIQUE-FACTOR VARIANCES:

DEPRESSED_Y2 ~~ c(1, NA, NA, NA, NA, NA, NA, NA, NA, NA, NA, NA, NA, NA, NA, NA, NA, NA, NA,
INTEREST_Y2 ~~ c(1, NA, NA, NA, NA, NA, NA, NA, NA, NA, NA, NA, NA, NA, NA, NA, NA, NA, NA, N
CONTROL_WORRY_Y2 ~~ c(1, NA, NA, NA, NA, NA, NA, NA, NA, NA, NA, NA, NA, NA, NA, NA, NA, NA,
FEEL_ANXIOUS_Y2 ~~ c(1, NA, NA, NA, NA, NA, NA, NA, NA, NA, NA, NA, NA, NA, NA, NA, NA, NA, N

## LATENT MEANS/INTERCEPTS:

dep ~ c(0, 0, 0, 0, 0, 0, 0, 0, 0, 0, 0, 0, 0, 0, 0, 0, 0, 0, 0, 0, 0, 0, 0)*1 + c(alpha.1.g
anx ~ c(0, 0, 0, 0, 0, 0, 0, 0, 0, 0, 0, 0, 0, 0, 0, 0, 0, 0, 0, 0, 0, 0, 0)*1 + c(alpha.2.g

## COMMON-FACTOR VARIANCES:

dep ~~ c(1, 1, 1, 1, 1, 1, 1, 1, 1, 1, 1, 1, 1, 1, 1, 1, 1, 1, 1, 1, 1, 1, 1)*dep + c(psi.1_
anx ~~ c(1, 1, 1, 1, 1, 1, 1, 1, 1, 1, 1, 1, 1, 1, 1, 1, 1, 1, 1, 1, 1, 1, 1)*anx + c(psi.2_

## COMMON-FACTOR COVARIANCES:

dep ~~ c(NA, NA, NA, NA, NA, NA, NA, NA, NA, NA, NA, NA, NA, NA, NA, NA, NA, NA, NA, NA, NA,
"
fit.threshold.partial <- cfa(
  model = as.character(mod.threshold.partial)
  , data = df.cc
  , group = "COUNTRY"
  , ordered = TRUE
  , parameterization = "theta"
  , missing = "pairwise"
)

fit.threshold.partial
```

```
lavaan 0.6-21 ended normally after 2637 iterations
```

```
Estimator                                         DWLS
Optimization method                             NLMINB
Number of model parameters                         567
Number of equality constraints                     250

Number of observations per group:                 Used       Total
  Sweden                                         11607       15068
  United Kingdom                                  3619        5368
  Germany                                         5528        9506
  China                                           4544        5022
  Argentina                                       2928        6724
  Australia                                       2581        3844
  Brazil                                          4274       13203
  Egypt                                           3040        4729
  India                                           6372       12765
  Indonesia                                       2681        6992
  Israel                                          2489        3669
  Japan                                          13968       20543
  Kenya                                           7698       11389
  Mexico                                          2278        5776
  Nigeria                                         3146        6827
  Philippines                                     2682        5292
  Poland                                          6478       10389
  South Africa                                     978        2651
  Spain                                           2923        6290
  Tanzania                                        5583        9075
  Turkey                                           499        1473
  United States                                  32239       38312
  Hong Kong                                        707        3012
Number of missing patterns per group:
  Sweden                                            13
  United Kingdom                                     6
  Germany                                            8
  China                                              4
  Argentina                                         10
  Australia                                          7
  Brazil                                             8
  Egypt                                              5
  India                                             13
  Indonesia                                          6
  Israel                                             9
  Japan                                              9
  Kenya                                              6
```

```
    Mexico                                        8
    Nigeria                                       9
    Philippines                                   4
    Poland                                        9
    South Africa                                  4
    Spain                                         7
    Tanzania                                     10
    Turkey                                        3
    United States                                14
    Hong Kong                                     3

Model Test User Model:
                                          Standard      Scaled
  Test Statistic                           604.883    1551.415
  Degrees of freedom                            97          97
  P-value (Unknown)                             NA       0.000
  Scaling correction factor                              0.392
  Shift parameter                                        8.655
    simple second-order correction
  Test statistic for each group:
    Sweden                                  75.894      75.894
    United Kingdom                          32.310      32.310
    Germany                                 14.562      14.562
    China                                   43.769      43.769
    Argentina                               37.582      37.582
    Australia                               13.168      13.168
    Brazil                                  15.281      15.281
    Egypt                                  142.947     142.947
    India                                  285.441     285.441
    Indonesia                               82.998      82.998
    Israel                                  57.111      57.111
    Japan                                  176.074     176.074
    Kenya                                  192.798     192.798
    Mexico                                  24.400      24.400
    Nigeria                                148.348     148.348
    Philippines                             39.133      39.133
    Poland                                  10.179      10.179
    South Africa                             9.918       9.918
    Spain                                   17.465      17.465
    Tanzania                                 3.852       3.852
    Turkey                                  14.780      14.780
    United States                          106.277     106.277
    Hong Kong                                7.129       7.129
```

```r
#### Residuals
residuals(fit.threshold.partial)
```

```
$Sweden
$Sweden$type
[1] "raw"

$Sweden$cov
                 DEPRES INTERE CONTRO FEEL_A
DEPRESSED_Y2      0.000
INTEREST_Y2       0.000  0.000
CONTROL_WORRY_Y2 -0.003  0.011  0.000
FEEL_ANXIOUS_Y2   0.003 -0.011  0.000  0.000

$Sweden$mean
    DEPRESSED_Y2      INTEREST_Y2 CONTROL_WORRY_Y2  FEEL_ANXIOUS_Y2
               0                0                0                0

$Sweden$th
    DEPRESSED_Y2|t1     DEPRESSED_Y2|t2     DEPRESSED_Y2|t3      INTEREST_Y2|t1      INTEREST
             -0.004               0.018              -0.018               0.015
0.043              0.046              0.003             -0.012              0.014
0.003
 FEEL_ANXIOUS_Y2|t2  FEEL_ANXIOUS_Y2|t3
              0.015              -0.016


$`United Kingdom`
$`United Kingdom`$type
[1] "raw"

$`United Kingdom`$cov
                 DEPRES INTERE CONTRO FEEL_A
DEPRESSED_Y2          0
INTEREST_Y2           0      0
CONTROL_WORRY_Y2      0      0      0
FEEL_ANXIOUS_Y2       0      0      0      0

$`United Kingdom`$mean
    DEPRESSED_Y2      INTEREST_Y2 CONTROL_WORRY_Y2  FEEL_ANXIOUS_Y2
               0                0                0                0
```

```
$`United Kingdom`$th
    DEPRESSED_Y2|t1     DEPRESSED_Y2|t2     DEPRESSED_Y2|t3      INTEREST_Y2|t1      INTEREST
              0.012              -0.045               0.050               0.006
0.019               0.018               0.005              -0.017               0.017
 FEEL_ANXIOUS_Y2|t2  FEEL_ANXIOUS_Y2|t3
             -0.036               0.038


$Germany
$Germany$type
[1] "raw"

$Germany$cov
                 DEPRES INTERE CONTRO FEEL_A
DEPRESSED_Y2      0.000
INTEREST_Y2       0.000  0.000
CONTROL_WORRY_Y2 -0.004  0.006  0.000
FEEL_ANXIOUS_Y2   0.005 -0.007  0.000  0.000

$Germany$mean
    DEPRESSED_Y2      INTEREST_Y2 CONTROL_WORRY_Y2  FEEL_ANXIOUS_Y2
               0                0                0                0

$Germany$th
    DEPRESSED_Y2|t1     DEPRESSED_Y2|t2     DEPRESSED_Y2|t3      INTEREST_Y2|t1      INTEREST
              0.000              -0.002               0.002                   -
0.007               0.026              -0.028               0.001                   -
0.002               0.002               0.004
 FEEL_ANXIOUS_Y2|t2  FEEL_ANXIOUS_Y2|t3
             -0.018               0.023


$China
$China$type
[1] "raw"

$China$cov
                 DEPRES INTERE CONTRO FEEL_A
DEPRESSED_Y2      0.000
INTEREST_Y2       0.000  0.000
CONTROL_WORRY_Y2 -0.008  0.020  0.000
FEEL_ANXIOUS_Y2   0.008 -0.019  0.000  0.000
```

```
$China$mean
    DEPRESSED_Y2      INTEREST_Y2 CONTROL_WORRY_Y2  FEEL_ANXIOUS_Y2
               0                0                0                0

$China$th
    DEPRESSED_Y2|t1     DEPRESSED_Y2|t2     DEPRESSED_Y2|t3      INTEREST_Y2|t1      INTEREST
              0.005              -0.024               0.051               0.000
0.041              0.070               0.005
 FEEL_ANXIOUS_Y2|t2  FEEL_ANXIOUS_Y2|t3
             -0.021               0.042


$Argentina
$Argentina$type
[1] "raw"

$Argentina$cov
                 DEPRES INTERE CONTRO FEEL_A
DEPRESSED_Y2      0.000
INTEREST_Y2       0.000  0.000
CONTROL_WORRY_Y2  0.002 -0.004  0.000
FEEL_ANXIOUS_Y2  -0.002  0.003  0.000  0.000

$Argentina$mean
    DEPRESSED_Y2      INTEREST_Y2 CONTROL_WORRY_Y2  FEEL_ANXIOUS_Y2
               0                0                0                0

$Argentina$th
    DEPRESSED_Y2|t1     DEPRESSED_Y2|t2     DEPRESSED_Y2|t3      INTEREST_Y2|t1      INTEREST
              0.002              -0.008               0.008                   -
0.026               0.069               -0.059              -0.008               0.024
0.020              -0.004
 FEEL_ANXIOUS_Y2|t2  FEEL_ANXIOUS_Y2|t3
              0.012              -0.011


$Australia
$Australia$type
[1] "raw"

$Australia$cov
                 DEPRES INTERE CONTRO FEEL_A
DEPRESSED_Y2      0.000
```

```
INTEREST_Y2       0.000  0.000
CONTROL_WORRY_Y2  0.000 -0.001  0.000
FEEL_ANXIOUS_Y2   0.000  0.001  0.000  0.000

$Australia$mean
    DEPRESSED_Y2      INTEREST_Y2 CONTROL_WORRY_Y2  FEEL_ANXIOUS_Y2
               0                0                0                0

$Australia$th
    DEPRESSED_Y2|t1     DEPRESSED_Y2|t2     DEPRESSED_Y2|t3      INTEREST_Y2|t1      INTEREST
              0.000               0.001              -0.001                   -
0.011              0.039               -0.042              -0.007               0.027
0.028             -0.005
 FEEL_ANXIOUS_Y2|t2  FEEL_ANXIOUS_Y2|t3
              0.021              -0.024


$Brazil
$Brazil$type
[1] "raw"

$Brazil$cov
                 DEPRES INTERE CONTRO FEEL_A
DEPRESSED_Y2      0.000
INTEREST_Y2       0.000  0.000
CONTROL_WORRY_Y2 -0.006  0.010  0.000
FEEL_ANXIOUS_Y2   0.005 -0.009  0.000  0.000

$Brazil$mean
    DEPRESSED_Y2      INTEREST_Y2 CONTROL_WORRY_Y2  FEEL_ANXIOUS_Y2
               0                0                0                0

$Brazil$th
    DEPRESSED_Y2|t1     DEPRESSED_Y2|t2     DEPRESSED_Y2|t3      INTEREST_Y2|t1      INTEREST
              0.009              -0.029               0.026               0.002
0.006              0.004                0.002              -0.005               0.004
 FEEL_ANXIOUS_Y2|t2  FEEL_ANXIOUS_Y2|t3
              0.000               0.000


$Egypt
$Egypt$type
[1] "raw"
```

```
$Egypt$cov
                 DEPRES INTERE CONTRO FEEL_A
DEPRESSED_Y2      0.000
INTEREST_Y2       0.000  0.000
CONTROL_WORRY_Y2  0.001 -0.002  0.000
FEEL_ANXIOUS_Y2  -0.001  0.002  0.000  0.000

$Egypt$mean
    DEPRESSED_Y2      INTEREST_Y2 CONTROL_WORRY_Y2  FEEL_ANXIOUS_Y2
               0                0                0                0

$Egypt$th
    DEPRESSED_Y2|t1     DEPRESSED_Y2|t2     DEPRESSED_Y2|t3      INTEREST_Y2|t1      INTEREST
         -0.011               0.036              -0.030                   -
0.043               0.109              -0.088              -0.016               0.042
0.033              -0.030
 FEEL_ANXIOUS_Y2|t2  FEEL_ANXIOUS_Y2|t3
              0.070              -0.051


$India
$India$type
[1] "raw"

$India$cov
                 DEPRES INTERE CONTRO FEEL_A
DEPRESSED_Y2      0.000
INTEREST_Y2       0.000  0.000
CONTROL_WORRY_Y2 -0.012  0.038  0.000
FEEL_ANXIOUS_Y2   0.008 -0.035  0.000  0.000

$India$mean
    DEPRESSED_Y2      INTEREST_Y2 CONTROL_WORRY_Y2  FEEL_ANXIOUS_Y2
               0                0                0                0

$India$th
    DEPRESSED_Y2|t1     DEPRESSED_Y2|t2     DEPRESSED_Y2|t3      INTEREST_Y2|t1      INTEREST
              0.000               0.000               0.000               0.028
0.060               0.040               0.039              -0.106               0.086
 FEEL_ANXIOUS_Y2|t2  FEEL_ANXIOUS_Y2|t3
              0.000               0.000
```

```
$Indonesia
$Indonesia$type
[1] "raw"

$Indonesia$cov
                 DEPRES INTERE CONTRO FEEL_A
DEPRESSED_Y2      0.000
INTEREST_Y2       0.000  0.000
CONTROL_WORRY_Y2 -0.004  0.010  0.000
FEEL_ANXIOUS_Y2   0.003 -0.008  0.000  0.000

$Indonesia$mean
    DEPRESSED_Y2      INTEREST_Y2 CONTROL_WORRY_Y2  FEEL_ANXIOUS_Y2
               0                0                0                0

$Indonesia$th
    DEPRESSED_Y2|t1     DEPRESSED_Y2|t2     DEPRESSED_Y2|t3      INTEREST_Y2|t1      INTERES
          -0.020               0.089              -0.081               0.000
0.015               0.059              -0.053              -0.018
 FEEL_ANXIOUS_Y2|t2  FEEL_ANXIOUS_Y2|t3
           0.079              -0.072


$Israel
$Israel$type
[1] "raw"

$Israel$cov
                 DEPRES INTERE CONTRO FEEL_A
DEPRESSED_Y2      0.000
INTEREST_Y2       0.000  0.000
CONTROL_WORRY_Y2  0.015 -0.034  0.000
FEEL_ANXIOUS_Y2  -0.007  0.016  0.000  0.000

$Israel$mean
    DEPRESSED_Y2      INTEREST_Y2 CONTROL_WORRY_Y2  FEEL_ANXIOUS_Y2
               0                0                0                0

$Israel$th
    DEPRESSED_Y2|t1     DEPRESSED_Y2|t2     DEPRESSED_Y2|t3      INTEREST_Y2|t1      INTERES
           0.009              -0.038               0.051               0.010
0.034               0.044               0.013              -0.052               0.075
```

```
 FEEL_ANXIOUS_Y2|t2  FEEL_ANXIOUS_Y2|t3
            -0.055               0.079


$Japan
$Japan$type
[1] "raw"

$Japan$cov
                 DEPRES INTERE CONTRO FEEL_A
DEPRESSED_Y2      0.000
INTEREST_Y2       0.000  0.000
CONTROL_WORRY_Y2  0.001 -0.002  0.000
FEEL_ANXIOUS_Y2   0.000  0.001  0.000  0.000

$Japan$mean
    DEPRESSED_Y2      INTEREST_Y2 CONTROL_WORRY_Y2  FEEL_ANXIOUS_Y2
               0                0                0                0

$Japan$th
    DEPRESSED_Y2|t1     DEPRESSED_Y2|t2     DEPRESSED_Y2|t3      INTEREST_Y2|t1      INTEREST
             0.010              -0.038               0.042               0.015
0.045               0.049               0.009              -0.036               0.042
 FEEL_ANXIOUS_Y2|t2  FEEL_ANXIOUS_Y2|t3
            -0.031               0.034


$Kenya
$Kenya$type
[1] "raw"

$Kenya$cov
                 DEPRES INTERE CONTRO FEEL_A
DEPRESSED_Y2      0.000
INTEREST_Y2       0.000  0.000
CONTROL_WORRY_Y2 -0.001  0.002  0.000
FEEL_ANXIOUS_Y2   0.001 -0.001  0.000  0.000

$Kenya$mean
    DEPRESSED_Y2      INTEREST_Y2 CONTROL_WORRY_Y2  FEEL_ANXIOUS_Y2
               0                0                0                0

$Kenya$th
```

```
   DEPRESSED_Y2|t1     DEPRESSED_Y2|t2     DEPRESSED_Y2|t3      INTEREST_Y2|t1      INTEREST
            -0.026               0.077              -0.064               0.000
0.024               0.066              -0.051              -0.007
 FEEL_ANXIOUS_Y2|t2  FEEL_ANXIOUS_Y2|t3
             0.021              -0.019


$Mexico
$Mexico$type
[1] "raw"

$Mexico$cov
                 DEPRES INTERE CONTRO FEEL_A
DEPRESSED_Y2      0.000
INTEREST_Y2       0.000  0.000
CONTROL_WORRY_Y2 -0.008  0.012  0.000
FEEL_ANXIOUS_Y2   0.005 -0.011  0.000  0.000

$Mexico$mean
   DEPRESSED_Y2      INTEREST_Y2 CONTROL_WORRY_Y2  FEEL_ANXIOUS_Y2
              0                0                0                0

$Mexico$th
   DEPRESSED_Y2|t1     DEPRESSED_Y2|t2     DEPRESSED_Y2|t3      INTEREST_Y2|t1      INTEREST
             0.003              -0.011               0.011                -
0.021               0.059              -0.051              -0.001               0.003
0.003               0.004
 FEEL_ANXIOUS_Y2|t2  FEEL_ANXIOUS_Y2|t3
            -0.016               0.017


$Nigeria
$Nigeria$type
[1] "raw"

$Nigeria$cov
                 DEPRES INTERE CONTRO FEEL_A
DEPRESSED_Y2      0.000
INTEREST_Y2       0.000  0.000
CONTROL_WORRY_Y2 -0.013  0.021  0.000
FEEL_ANXIOUS_Y2   0.012 -0.022  0.000  0.000

$Nigeria$mean
```

```
   DEPRESSED_Y2      INTEREST_Y2 CONTROL_WORRY_Y2  FEEL_ANXIOUS_Y2
              0                0                0                0

$Nigeria$th
   DEPRESSED_Y2|t1     DEPRESSED_Y2|t2     DEPRESSED_Y2|t3      INTEREST_Y2|t1      INTEREST
             0.029              -0.081               0.075                   -
0.010               0.019              -0.014               0.004                  -
0.009               0.008               0.032
 FEEL_ANXIOUS_Y2|t2  FEEL_ANXIOUS_Y2|t3
            -0.097               0.095


$Philippines
$Philippines$type
[1] "raw"

$Philippines$cov
                 DEPRES INTERE CONTRO FEEL_A
DEPRESSED_Y2      0.000
INTEREST_Y2       0.000  0.000
CONTROL_WORRY_Y2 -0.011  0.015  0.000
FEEL_ANXIOUS_Y2   0.008 -0.015  0.000  0.000

$Philippines$mean
   DEPRESSED_Y2      INTEREST_Y2 CONTROL_WORRY_Y2  FEEL_ANXIOUS_Y2
              0                0                0                0

$Philippines$th
   DEPRESSED_Y2|t1     DEPRESSED_Y2|t2     DEPRESSED_Y2|t3      INTEREST_Y2|t1      INTEREST
             0.000               0.000               0.000               0.032
0.062               0.045               0.014              -0.030               0.021
 FEEL_ANXIOUS_Y2|t2  FEEL_ANXIOUS_Y2|t3
             0.000               0.000


$Poland
$Poland$type
[1] "raw"

$Poland$cov
                 DEPRES INTERE CONTRO FEEL_A
DEPRESSED_Y2      0.000
INTEREST_Y2       0.000  0.000
```

```
CONTROL_WORRY_Y2  0.005 -0.007  0.000
FEEL_ANXIOUS_Y2  -0.008  0.008  0.000  0.000

$Poland$mean
    DEPRESSED_Y2      INTEREST_Y2 CONTROL_WORRY_Y2  FEEL_ANXIOUS_Y2
               0                0                0                0

$Poland$th
    DEPRESSED_Y2|t1     DEPRESSED_Y2|t2     DEPRESSED_Y2|t3      INTEREST_Y2|t1      INTEREST
              0.002              -0.014               0.023                  -
0.003               0.014              -0.025               0.001                  -
0.006               0.010              -0.001
 FEEL_ANXIOUS_Y2|t2  FEEL_ANXIOUS_Y2|t3
              0.009              -0.015


$`South Africa`
$`South Africa`$type
[1] "raw"

$`South Africa`$cov
                 DEPRES INTERE CONTRO FEEL_A
DEPRESSED_Y2      0.000
INTEREST_Y2       0.000  0.000
CONTROL_WORRY_Y2  0.002 -0.003  0.000
FEEL_ANXIOUS_Y2  -0.002  0.003  0.000  0.000

$`South Africa`$mean
    DEPRESSED_Y2      INTEREST_Y2 CONTROL_WORRY_Y2  FEEL_ANXIOUS_Y2
               0                0                0                0

$`South Africa`$th
    DEPRESSED_Y2|t1     DEPRESSED_Y2|t2     DEPRESSED_Y2|t3      INTEREST_Y2|t1      INTEREST
              0.006              -0.017               0.015                  -
0.022               0.052              -0.042               0.005                  -
0.012               0.010               0.010
 FEEL_ANXIOUS_Y2|t2  FEEL_ANXIOUS_Y2|t3
             -0.030               0.028


$Spain
$Spain$type
[1] "raw"
```

```
$Spain$cov
                 DEPRES INTERE CONTRO FEEL_A
DEPRESSED_Y2      0.000
INTEREST_Y2       0.000  0.000
CONTROL_WORRY_Y2 -0.003  0.005  0.000
FEEL_ANXIOUS_Y2   0.003 -0.005  0.000  0.000

$Spain$mean
    DEPRESSED_Y2      INTEREST_Y2 CONTROL_WORRY_Y2  FEEL_ANXIOUS_Y2
               0                0                0                0

$Spain$th
    DEPRESSED_Y2|t1     DEPRESSED_Y2|t2     DEPRESSED_Y2|t3      INTEREST_Y2|t1      INTEREST
             -0.001               0.002              -0.002                   -
0.012              0.031              -0.030              0.006              -
0.018              0.017              -0.012
 FEEL_ANXIOUS_Y2|t2  FEEL_ANXIOUS_Y2|t3
              0.031              -0.028


$Tanzania
$Tanzania$type
[1] "raw"

$Tanzania$cov
                 DEPRES INTERE CONTRO FEEL_A
DEPRESSED_Y2      0.000
INTEREST_Y2       0.000  0.000
CONTROL_WORRY_Y2 -0.005  0.011  0.000
FEEL_ANXIOUS_Y2   0.004 -0.010  0.000  0.000

$Tanzania$mean
    DEPRESSED_Y2      INTEREST_Y2 CONTROL_WORRY_Y2  FEEL_ANXIOUS_Y2
               0                0                0                0

$Tanzania$th
    DEPRESSED_Y2|t1     DEPRESSED_Y2|t2     DEPRESSED_Y2|t3      INTEREST_Y2|t1      INTEREST
                  0                   0                   0                   0
 FEEL_ANXIOUS_Y2|t2  FEEL_ANXIOUS_Y2|t3
                  0                   0
```

```
$Turkey
$Turkey$type
[1] "raw"

$Turkey$cov
                 DEPRES INTERE CONTRO FEEL_A
DEPRESSED_Y2      0.000
INTEREST_Y2       0.000  0.000
CONTROL_WORRY_Y2  0.003 -0.005  0.000
FEEL_ANXIOUS_Y2  -0.001  0.002  0.000  0.000

$Turkey$mean
    DEPRESSED_Y2      INTEREST_Y2 CONTROL_WORRY_Y2  FEEL_ANXIOUS_Y2
               0                0                0                0

$Turkey$th
    DEPRESSED_Y2|t1     DEPRESSED_Y2|t2     DEPRESSED_Y2|t3      INTEREST_Y2|t1      INTEREST
              0.027              -0.074               0.060               0.019
0.044               0.031               0.012              -0.034               0.029
 FEEL_ANXIOUS_Y2|t2  FEEL_ANXIOUS_Y2|t3
             -0.053               0.042


$`United States`
$`United States`$type
[1] "raw"

$`United States`$cov
                 DEPRES INTERE CONTRO FEEL_A
DEPRESSED_Y2      0.000
INTEREST_Y2       0.000  0.000
CONTROL_WORRY_Y2 -0.002  0.003  0.000
FEEL_ANXIOUS_Y2   0.002 -0.003  0.000  0.000

$`United States`$mean
    DEPRESSED_Y2      INTEREST_Y2 CONTROL_WORRY_Y2  FEEL_ANXIOUS_Y2
               0                0                0                0

$`United States`$th
    DEPRESSED_Y2|t1     DEPRESSED_Y2|t2     DEPRESSED_Y2|t3      INTEREST_Y2|t1      INTEREST
             -0.002               0.011              -0.014                   -
0.006               0.026              -0.032              -0.007               0.030
0.034              -0.003
```

```
 FEEL_ANXIOUS_Y2|t2  FEEL_ANXIOUS_Y2|t3
             0.012              -0.015


$`Hong Kong`
$`Hong Kong`$type
[1] "raw"

$`Hong Kong`$cov
                 DEPRES INTERE CONTRO FEEL_A
DEPRESSED_Y2      0.000
INTEREST_Y2       0.000  0.000
CONTROL_WORRY_Y2 -0.001  0.004  0.000
FEEL_ANXIOUS_Y2   0.001 -0.004  0.000  0.000

$`Hong Kong`$mean
    DEPRESSED_Y2      INTEREST_Y2 CONTROL_WORRY_Y2  FEEL_ANXIOUS_Y2
               0                0                0                0

$`Hong Kong`$th
    DEPRESSED_Y2|t1     DEPRESSED_Y2|t2     DEPRESSED_Y2|t3      INTEREST_Y2|t1      INTERES
             0.000               0.000               0.000               0.024
0.044               0.057               0.000               0.000               0.000
 FEEL_ANXIOUS_Y2|t2  FEEL_ANXIOUS_Y2|t3
            -0.039               0.059
```

```
fit.res.thr0 <- lapply(residuals(fit.threshold.partial), \(x) x$th)
fit.res.thr <- fit.res.thr0 |> bind_rows() |> as.data.frame()
rownames(fit.res.thr) <- paste0(names(fit.res.thr0), " (", 1:length(names(fit.res.thr0)), ")'
fit.res.thr |> select(contains("t3")) |> mutate(across(everything(), ~round(.,2)))
```

```
                   DEPRESSED_Y2|t3 INTEREST_Y2|t3 CONTROL_WORRY_Y2|t3 FEEL_ANXIOUS_Y2|t3
Sweden (1)                   -0.02           0.05                0.01                  -
0.02
United Kingdom (2)            0.05           0.02                0.02               0.04
Germany (3)                   0.00          -0.03                0.00               0.02
China (4)                     0.05           0.00                0.07               0.04
Argentina (5)                 0.01          -0.06               -0.02                  -
0.01
Australia (6)                 0.00          -0.04               -0.03                  -
0.02
Brazil (7)                    0.03           0.00                0.00               0.00
```

```
Egypt (8)                 -0.03          -0.09               -0.03            -
0.05
India (9)                  0.00           0.04                0.09          0.00
Indonesia (10)            -0.08           0.00               -0.05            -
0.07
Israel (11)                0.05           0.04                0.07          0.08
Japan (12)                 0.04           0.05                0.04          0.03
Kenya (13)                -0.06           0.00               -0.05            -
0.02
Mexico (14)                0.01          -0.05                0.00          0.02
Nigeria (15)               0.07          -0.01                0.01          0.09
Philippines (16)           0.00           0.05                0.02          0.00
Poland (17)                0.02          -0.02                0.01            -
0.02
South Africa (18)          0.01          -0.04                0.01          0.03
Spain (19)                 0.00          -0.03                0.02            -
0.03
Tanzania (20)              0.00           0.00                0.00          0.00
Turkey (21)                0.06           0.03                0.03          0.04
United States (22)        -0.01          -0.03               -0.03            -
0.01
Hong Kong (23)             0.00           0.06                0.00          0.06
```

Model fit is much better, nearly back to the fit of the configural model (as expected).

4.2.5 Metric Invariance Model

```
mod.metric <- semTools::measEq.syntax(
  configural.model = mod
  , data = df.cc
  , group = "COUNTRY"
  , parameterization = "theta"
  , ID.fac = "std.lv"
  , ID.cat = "Wu.Estabrook.2016"
  , ordered = TRUE
  , group.equal = c("thresholds","loadings")
  , group.partial = c(
    'DEPRESSED_Y2 | t3',
    'INTEREST_Y2 | t3',
    'CONTROL_WORRY_Y2 | t3',
    'FEEL_ANXIOUS_Y2 | t3'
  )
)

summary(mod.metric)
```

```
This lavaan model syntax specifies a CFA with 4 manifest indicators (4 of which are ordinal)

To identify the location and scale of each common factor, the factor means and variances were

The location and scale of each latent item-response underlying 4 ordinal indicators were iden

    https://doi.org/10.1007/s11336-016-9506-0

Pattern matrix indicating num(eric), ord(ered), and lat(ent) indicators per factor:

                 dep anx
DEPRESSED_Y2     ord
INTEREST_Y2      ord
CONTROL_WORRY_Y2     ord
FEEL_ANXIOUS_Y2      ord

The following types of parameter were constrained to equality across groups:

    thresholds, with the exception of:
```

```
                    lhs op rhs
row-1:        DEPRESSED_Y2  |  t3
row-2:         INTEREST_Y2  |  t3
row-3:    CONTROL_WORRY_Y2  |  t3
row-4:     FEEL_ANXIOUS_Y2  |  t3
```

```
loadings
```

```
#cat(as.character(mod.metric))
# copy the threshold partial invariance from previous model over
mod.metric <- "
## LOADINGS:

dep =~ c(NA, NA, NA, NA, NA, NA, NA, NA, NA, NA, NA, NA, NA, NA, NA, NA, NA, NA, NA, NA, NA,
dep =~ c(NA, NA, NA, NA, NA, NA, NA, NA, NA, NA, NA, NA, NA, NA, NA, NA, NA, NA, NA, NA, NA,
anx =~ c(NA, NA, NA, NA, NA, NA, NA, NA, NA, NA, NA, NA, NA, NA, NA, NA, NA, NA, NA, NA, NA,
anx =~ c(NA, NA, NA, NA, NA, NA, NA, NA, NA, NA, NA, NA, NA, NA, NA, NA, NA, NA, NA, NA, NA,

## THRESHOLDS:

DEPRESSED_Y2 | c(NA, NA, NA, NA, NA, NA, NA, NA, NA, NA, NA, NA, NA, NA, NA, NA, NA, NA, NA,
DEPRESSED_Y2 | c(NA, NA, NA, NA, NA, NA, NA, NA, NA, NA, NA, NA, NA, NA, NA, NA, NA, NA, NA,
DEPRESSED_Y2 | c(NA, NA, NA, NA, NA, NA, NA, NA, NA, NA, NA, NA, NA, NA, NA, NA, NA, NA, NA,
INTEREST_Y2 | c(NA, NA, NA, NA, NA, NA, NA, NA, NA, NA, NA, NA, NA, NA, NA, NA, NA, NA, NA, N
INTEREST_Y2 | c(NA, NA, NA, NA, NA, NA, NA, NA, NA, NA, NA, NA, NA, NA, NA, NA, NA, NA, NA, N
INTEREST_Y2 | c(NA, NA, NA, NA, NA, NA, NA, NA, NA, NA, NA, NA, NA, NA, NA, NA, NA, NA, NA, N
CONTROL_WORRY_Y2 | c(NA, NA, NA, NA, NA, NA, NA, NA, NA, NA, NA, NA, NA, NA, NA, NA, NA, NA,
CONTROL_WORRY_Y2 | c(NA, NA, NA, NA, NA, NA, NA, NA, NA, NA, NA, NA, NA, NA, NA, NA, NA, NA,
CONTROL_WORRY_Y2 | c(NA, NA, NA, NA, NA, NA, NA, NA, NA, NA, NA, NA, NA, NA, NA, NA, NA, NA,
FEEL_ANXIOUS_Y2 | c(NA, NA, NA, NA, NA, NA, NA, NA, NA, NA, NA, NA, NA, NA, NA, NA, NA, NA, N
FEEL_ANXIOUS_Y2 | c(NA, NA, NA, NA, NA, NA, NA, NA, NA, NA, NA, NA, NA, NA, NA, NA, NA, NA, N
FEEL_ANXIOUS_Y2 | c(NA, NA, NA, NA, NA, NA, NA, NA, NA, NA, NA, NA, NA, NA, NA, NA, NA, NA, N

## INTERCEPTS:

DEPRESSED_Y2 ~ c(0, NA, NA, NA, NA, NA, NA, NA, NA, NA, NA, NA, NA, NA, NA, NA, NA, NA, NA, N
INTEREST_Y2 ~ c(0, NA, NA, NA, NA, NA, NA, NA, NA, NA, NA, NA, NA, NA, NA, NA, NA, NA, NA, NA
CONTROL_WORRY_Y2 ~ c(0, NA, NA, NA, NA, NA, NA, NA, NA, NA, NA, NA, NA, NA, NA, NA, NA, NA, N
FEEL_ANXIOUS_Y2 ~ c(0, NA, NA, NA, NA, NA, NA, NA, NA, NA, NA, NA, NA, NA, NA, NA, NA, NA, NA

## UNIQUE-FACTOR VARIANCES:
```

```
DEPRESSED_Y2 ~~ c(1, NA, NA, NA, NA, NA, NA, NA, NA, NA, NA, NA, NA, NA, NA, NA, NA, NA, NA, NA,
INTEREST_Y2 ~~ c(1, NA, NA, NA, NA, NA, NA, NA, NA, NA, NA, NA, NA, NA, NA, NA, NA, NA, NA, N
CONTROL_WORRY_Y2 ~~ c(1, NA, NA, NA, NA, NA, NA, NA, NA, NA, NA, NA, NA, NA, NA, NA, NA, NA, NA,
FEEL_ANXIOUS_Y2 ~~ c(1, NA, NA, NA, NA, NA, NA, NA, NA, NA, NA, NA, NA, NA, NA, NA, NA, NA, N

## LATENT MEANS/INTERCEPTS:

dep ~ c(0, 0, 0, 0, 0, 0, 0, 0, 0, 0, 0, 0, 0, 0, 0, 0, 0, 0, 0, 0, 0, 0, 0)*1 + c(alpha.1.g
anx ~ c(0, 0, 0, 0, 0, 0, 0, 0, 0, 0, 0, 0, 0, 0, 0, 0, 0, 0, 0, 0, 0, 0, 0)*1 + c(alpha.2.g

## COMMON-FACTOR VARIANCES:

dep ~~ c(1, NA, NA, NA, NA, NA, NA, NA, NA, NA, NA, NA, NA, NA, NA, NA, NA, NA, NA, NA, NA, N
anx ~~ c(1, NA, NA, NA, NA, NA, NA, NA, NA, NA, NA, NA, NA, NA, NA, NA, NA, NA, NA, NA, NA, N

## COMMON-FACTOR COVARIANCES:

dep ~~ c(NA, NA, NA, NA, NA, NA, NA, NA, NA, NA, NA, NA, NA, NA, NA, NA, NA, NA, NA, NA, NA,
"


fit.metric <- cfa(
  model = as.character(mod.metric)
  , data = df.cc
  , group = "COUNTRY"
  , ordered = TRUE
  , parameterization = "theta"
  , missing = "pairwise"
)

fit.metric
```

```
lavaan 0.6-21 ended normally after 1919 iterations

  Estimator                                       DWLS
  Optimization method                           NLMINB
  Number of model parameters                       611
  Number of equality constraints                   338

  Number of observations per group:               Used       Total
    Sweden                                       11607       15068
    United Kingdom                                3619        5368
```

```
  Germany                                    5528      9506
  China                                      4544      5022
  Argentina                                  2928      6724
  Australia                                  2581      3844
  Brazil                                     4274     13203
  Egypt                                      3040      4729
  India                                      6372     12765
  Indonesia                                  2681      6992
  Israel                                     2489      3669
  Japan                                     13968     20543
  Kenya                                      7698     11389
  Mexico                                     2278      5776
  Nigeria                                    3146      6827
  Philippines                                2682      5292
  Poland                                     6478     10389
  South Africa                                978      2651
  Spain                                      2923      6290
  Tanzania                                   5583      9075
  Turkey                                      499      1473
  United States                             32239     38312
  Hong Kong                                   707      3012
Number of missing patterns per group:
  Sweden                                       13
  United Kingdom                                6
  Germany                                       8
  China                                         4
  Argentina                                    10
  Australia                                     7
  Brazil                                        8
  Egypt                                         5
  India                                        13
  Indonesia                                     6
  Israel                                        9
  Japan                                         9
  Kenya                                         6
  Mexico                                        8
  Nigeria                                       9
  Philippines                                   4
  Poland                                        9
  South Africa                                  4
  Spain                                         7
  Tanzania                                     10
  Turkey                                        3
```

```
    United States                                   14
    Hong Kong                                        3

Model Test User Model:
                                              Standard      Scaled
  Test Statistic                              1396.760    2962.036
  Degrees of freedom                               141         141
  P-value (Unknown)                                 NA       0.000
  Scaling correction factor                                  0.474
  Shift parameter                                           13.208
    simple second-order correction
  Test statistic for each group:
    Sweden                                     139.347     139.347
    United Kingdom                              61.284      61.284
    Germany                                    139.151     139.151
    China                                       64.101      64.101
    Argentina                                   33.825      33.825
    Australia                                   18.264      18.264
    Brazil                                      19.868      19.868
    Egypt                                      209.750     209.750
    India                                      701.948     701.948
    Indonesia                                  215.764     215.764
    Israel                                     140.455     140.455
    Japan                                      165.052     165.052
    Kenya                                      320.276     320.276
    Mexico                                      28.348      28.348
    Nigeria                                    137.155     137.155
    Philippines                                133.523     133.523
    Poland                                      99.999      99.999
    South Africa                                 7.328       7.328
    Spain                                       24.876      24.876
    Tanzania                                    28.060      28.060
    Turkey                                      16.406      16.406
    United States                              208.330     208.330
    Hong Kong                                   48.925      48.925
```

```r
#### Residuals
residuals(fit.metric)
```

```
$Sweden
$Sweden$type
[1] "raw"
```

```
$Sweden$cov
                 DEPRES INTERE CONTRO FEEL_A
DEPRESSED_Y2      0.000
INTEREST_Y2      -0.001  0.000
CONTROL_WORRY_Y2  0.004  0.003  0.000
FEEL_ANXIOUS_Y2   0.001 -0.024  0.000  0.000

$Sweden$mean
    DEPRESSED_Y2      INTEREST_Y2 CONTROL_WORRY_Y2  FEEL_ANXIOUS_Y2
               0                0                0                0

$Sweden$th
    DEPRESSED_Y2|t1     DEPRESSED_Y2|t2     DEPRESSED_Y2|t3      INTEREST_Y2|t1      INTEREST
              0.016               0.009              -0.053                   -
0.007              -0.037               0.084               0.022                  -
0.022              -0.021              -0.021
 FEEL_ANXIOUS_Y2|t2  FEEL_ANXIOUS_Y2|t3
              0.028               0.004


$`United Kingdom`
$`United Kingdom`$type
[1] "raw"

$`United Kingdom`$cov
                 DEPRES INTERE CONTRO FEEL_A
DEPRESSED_Y2      0.000
INTEREST_Y2       0.000  0.000
CONTROL_WORRY_Y2 -0.006  0.007  0.000
FEEL_ANXIOUS_Y2  -0.003  0.009  0.000  0.000

$`United Kingdom`$mean
    DEPRESSED_Y2      INTEREST_Y2 CONTROL_WORRY_Y2  FEEL_ANXIOUS_Y2
               0                0                0                0

$`United Kingdom`$th
    DEPRESSED_Y2|t1     DEPRESSED_Y2|t2     DEPRESSED_Y2|t3      INTEREST_Y2|t1      INTEREST
             -0.031              -0.020               0.104               0.044
0.037              -0.029              -0.005              -0.011               0.028
 FEEL_ANXIOUS_Y2|t2  FEEL_ANXIOUS_Y2|t3
             -0.037               0.024
```

```
$Germany
$Germany$type
[1] "raw"

$Germany$cov
                 DEPRES INTERE CONTRO FEEL_A
DEPRESSED_Y2      0.000
INTEREST_Y2       0.000  0.000
CONTROL_WORRY_Y2 -0.012  0.030  0.000
FEEL_ANXIOUS_Y2  -0.013  0.008  0.000  0.000

$Germany$mean
    DEPRESSED_Y2      INTEREST_Y2 CONTROL_WORRY_Y2  FEEL_ANXIOUS_Y2
               0                0                0                0

$Germany$th
    DEPRESSED_Y2|t1     DEPRESSED_Y2|t2     DEPRESSED_Y2|t3      INTEREST_Y2|t1      INTEREST
          -0.058               0.038               0.088               0.045
0.008              -0.112               0.017              -0.008                   -
0.025              -0.012
 FEEL_ANXIOUS_Y2|t2  FEEL_ANXIOUS_Y2|t3
          -0.005               0.041


$China
$China$type
[1] "raw"

$China$cov
                 DEPRES INTERE CONTRO FEEL_A
DEPRESSED_Y2      0.000
INTEREST_Y2       0.000  0.000
CONTROL_WORRY_Y2  0.001  0.034  0.000
FEEL_ANXIOUS_Y2  -0.006 -0.021  0.000  0.000

$China$mean
    DEPRESSED_Y2      INTEREST_Y2 CONTROL_WORRY_Y2  FEEL_ANXIOUS_Y2
               0                0                0                0

$China$th
    DEPRESSED_Y2|t1     DEPRESSED_Y2|t2     DEPRESSED_Y2|t3      INTEREST_Y2|t1      INTEREST
          -0.004              -0.012               0.060               0.013
```

```
0.013               0.000               0.037               -0.063               0.007
0.021
 FEEL_ANXIOUS_Y2|t2  FEEL_ANXIOUS_Y2|t3
             0.005               0.083


$Argentina
$Argentina$type
[1] "raw"

$Argentina$cov
                 DEPRES INTERE CONTRO FEEL_A
DEPRESSED_Y2      0.000
INTEREST_Y2       0.000  0.000
CONTROL_WORRY_Y2  0.003 -0.011  0.000
FEEL_ANXIOUS_Y2   0.003  0.000  0.000  0.000

$Argentina$mean
    DEPRESSED_Y2      INTEREST_Y2 CONTROL_WORRY_Y2  FEEL_ANXIOUS_Y2
               0                0                0                0

$Argentina$th
    DEPRESSED_Y2|t1     DEPRESSED_Y2|t2     DEPRESSED_Y2|t3      INTEREST_Y2|t1      INTEREST
             0.016              -0.011              -0.014               -
0.037               0.068               -0.040               -0.017               0.029
0.012               0.003
 FEEL_ANXIOUS_Y2|t2  FEEL_ANXIOUS_Y2|t3
             0.013              -0.022


$Australia
$Australia$type
[1] "raw"

$Australia$cov
                 DEPRES INTERE CONTRO FEEL_A
DEPRESSED_Y2      0.000
INTEREST_Y2       0.000  0.000
CONTROL_WORRY_Y2 -0.005  0.007  0.000
FEEL_ANXIOUS_Y2  -0.003  0.010  0.000  0.000

$Australia$mean
    DEPRESSED_Y2      INTEREST_Y2 CONTROL_WORRY_Y2  FEEL_ANXIOUS_Y2
```

```
              0                 0                 0                 0

$Australia$th
    DEPRESSED_Y2|t1     DEPRESSED_Y2|t2     DEPRESSED_Y2|t3      INTEREST_Y2|t1      INTERES
          -0.021               0.018               0.027               0.008
0.068              -0.011               0.031              -0.026              -
0.003
 FEEL_ANXIOUS_Y2|t2  FEEL_ANXIOUS_Y2|t3
           0.023              -0.032


$Brazil
$Brazil$type
[1] "raw"

$Brazil$cov
                 DEPRES INTERE CONTRO FEEL_A
DEPRESSED_Y2      0.000
INTEREST_Y2       0.000  0.000
CONTROL_WORRY_Y2 -0.010  0.008  0.000
FEEL_ANXIOUS_Y2   0.007 -0.005  0.000  0.000

$Brazil$mean
    DEPRESSED_Y2      INTEREST_Y2 CONTROL_WORRY_Y2  FEEL_ANXIOUS_Y2
               0                0                0                0

$Brazil$th
    DEPRESSED_Y2|t1     DEPRESSED_Y2|t2     DEPRESSED_Y2|t3      INTEREST_Y2|t1      INTERES
           0.004              -0.024               0.028               0.008
0.010               0.003              -0.015               0.000               0.018
 FEEL_ANXIOUS_Y2|t2  FEEL_ANXIOUS_Y2|t3
          -0.019               0.000


$Egypt
$Egypt$type
[1] "raw"

$Egypt$cov
                 DEPRES INTERE CONTRO FEEL_A
DEPRESSED_Y2      0.000
INTEREST_Y2       0.000  0.000
CONTROL_WORRY_Y2  0.008 -0.055  0.000
```

```
FEEL_ANXIOUS_Y2   0.036 -0.027  0.000  0.000

$Egypt$mean
    DEPRESSED_Y2      INTEREST_Y2 CONTROL_WORRY_Y2  FEEL_ANXIOUS_Y2
               0                0                0                0

$Egypt$th
    DEPRESSED_Y2|t1     DEPRESSED_Y2|t2     DEPRESSED_Y2|t3      INTEREST_Y2|t1      INTEREST
              0.039               0.023              -0.086                   -
0.093              0.117               -0.024              -0.045              0.052
0.007             -0.002
 FEEL_ANXIOUS_Y2|t2  FEEL_ANXIOUS_Y2|t3
              0.069              -0.075


$India
$India$type
[1] "raw"

$India$cov
                 DEPRES INTERE CONTRO FEEL_A
DEPRESSED_Y2      0.000
INTEREST_Y2       0.000  0.000
CONTROL_WORRY_Y2 -0.023 -0.037  0.000
FEEL_ANXIOUS_Y2   0.052 -0.083  0.000  0.000

$India$mean
    DEPRESSED_Y2      INTEREST_Y2 CONTROL_WORRY_Y2  FEEL_ANXIOUS_Y2
               0                0                0                0

$India$th
    DEPRESSED_Y2|t1     DEPRESSED_Y2|t2     DEPRESSED_Y2|t3      INTEREST_Y2|t1      INTEREST
              0.081              -0.082               0.000                   -
0.040             -0.055               0.106              -0.021              -
0.089              0.145               0.072
 FEEL_ANXIOUS_Y2|t2  FEEL_ANXIOUS_Y2|t3
             -0.074               0.000


$Indonesia
$Indonesia$type
[1] "raw"
```

```
$Indonesia$cov
                 DEPRES INTERE CONTRO FEEL_A
DEPRESSED_Y2      0.000
INTEREST_Y2       0.000  0.000
CONTROL_WORRY_Y2  0.012 -0.027  0.000
FEEL_ANXIOUS_Y2   0.019 -0.047  0.000  0.000

$Indonesia$mean
    DEPRESSED_Y2      INTEREST_Y2 CONTROL_WORRY_Y2  FEEL_ANXIOUS_Y2
               0                0                0                0

$Indonesia$th
    DEPRESSED_Y2|t1     DEPRESSED_Y2|t2     DEPRESSED_Y2|t3      INTEREST_Y2|t1      INTEREST
              0.047               0.054              -0.182               -
0.098              0.128               0.000              -0.015              0.061
0.055             -0.019
 FEEL_ANXIOUS_Y2|t2  FEEL_ANXIOUS_Y2|t3
              0.082              -0.074


$Israel
$Israel$type
[1] "raw"

$Israel$cov
                 DEPRES INTERE CONTRO FEEL_A
DEPRESSED_Y2      0.000
INTEREST_Y2       0.000  0.000
CONTROL_WORRY_Y2 -0.008 -0.044  0.000
FEEL_ANXIOUS_Y2   0.000  0.031  0.000  0.000

$Israel$mean
    DEPRESSED_Y2      INTEREST_Y2 CONTROL_WORRY_Y2  FEEL_ANXIOUS_Y2
               0                0                0                0

$Israel$th
    DEPRESSED_Y2|t1     DEPRESSED_Y2|t2     DEPRESSED_Y2|t3      INTEREST_Y2|t1      INTEREST
             -0.012              -0.020               0.081               0.029
0.050              0.015              -0.068               0.012              0.223
 FEEL_ANXIOUS_Y2|t2  FEEL_ANXIOUS_Y2|t3
             -0.108              -0.045
```

```
$Japan
$Japan$type
[1] "raw"

$Japan$cov
                 DEPRES INTERE CONTRO FEEL_A
DEPRESSED_Y2      0.000
INTEREST_Y2       0.000  0.000
CONTROL_WORRY_Y2  0.000  0.001  0.000
FEEL_ANXIOUS_Y2  -0.001  0.003  0.000  0.000

$Japan$mean
    DEPRESSED_Y2      INTEREST_Y2 CONTROL_WORRY_Y2  FEEL_ANXIOUS_Y2
               0                0                0                0

$Japan$th
    DEPRESSED_Y2|t1     DEPRESSED_Y2|t2     DEPRESSED_Y2|t3      INTEREST_Y2|t1      INTEREST
             -0.007              -0.026               0.062               0.031
0.055              0.032              0.015              -0.038              0.029
 FEEL_ANXIOUS_Y2|t2  FEEL_ANXIOUS_Y2|t3
             -0.026               0.037


$Kenya
$Kenya$type
[1] "raw"

$Kenya$cov
                 DEPRES INTERE CONTRO FEEL_A
DEPRESSED_Y2      0.000
INTEREST_Y2       0.000  0.000
CONTROL_WORRY_Y2 -0.002 -0.036  0.000
FEEL_ANXIOUS_Y2   0.032 -0.013  0.000  0.000

$Kenya$mean
    DEPRESSED_Y2      INTEREST_Y2 CONTROL_WORRY_Y2  FEEL_ANXIOUS_Y2
               0                0                0                0

$Kenya$th
    DEPRESSED_Y2|t1     DEPRESSED_Y2|t2     DEPRESSED_Y2|t3      INTEREST_Y2|t1      INTEREST
              0.005               0.072              -0.100                   -
0.046              0.045              0.000              -0.055              0.076
0.022              0.019
```

```
 FEEL_ANXIOUS_Y2|t2  FEEL_ANXIOUS_Y2|t3
             0.015              -0.049


$Mexico
$Mexico$type
[1] "raw"

$Mexico$cov
                 DEPRES INTERE CONTRO FEEL_A
DEPRESSED_Y2      0.000
INTEREST_Y2       0.000  0.000
CONTROL_WORRY_Y2 -0.015  0.010  0.000
FEEL_ANXIOUS_Y2   0.007 -0.004  0.000  0.000

$Mexico$mean
    DEPRESSED_Y2      INTEREST_Y2 CONTROL_WORRY_Y2  FEEL_ANXIOUS_Y2
               0                0                0                0

$Mexico$th
    DEPRESSED_Y2|t1     DEPRESSED_Y2|t2     DEPRESSED_Y2|t3      INTEREST_Y2|t1      INTEREST
            -0.008              -0.003               0.020              -
0.010              0.051              -0.060              -0.030              0.015
 FEEL_ANXIOUS_Y2|t2  FEEL_ANXIOUS_Y2|t3
            -0.024              -0.016


$Nigeria
$Nigeria$type
[1] "raw"

$Nigeria$cov
                 DEPRES INTERE CONTRO FEEL_A
DEPRESSED_Y2      0.000
INTEREST_Y2       0.000  0.000
CONTROL_WORRY_Y2 -0.030  0.003  0.000
FEEL_ANXIOUS_Y2   0.032 -0.010  0.000  0.000

$Nigeria$mean
    DEPRESSED_Y2      INTEREST_Y2 CONTROL_WORRY_Y2  FEEL_ANXIOUS_Y2
               0                0                0                0

$Nigeria$th
```

```
    DEPRESSED_Y2|t1     DEPRESSED_Y2|t2     DEPRESSED_Y2|t3      INTEREST_Y2|t1      INTEREST
              0.032              -0.078               0.068               -
0.012               0.015              -0.007              -0.028               0.000
 FEEL_ANXIOUS_Y2|t2  FEEL_ANXIOUS_Y2|t3
             -0.104               0.063


$Philippines
$Philippines$type
[1] "raw"

$Philippines$cov
                 DEPRES INTERE CONTRO FEEL_A
DEPRESSED_Y2      0.000
INTEREST_Y2       0.000  0.000
CONTROL_WORRY_Y2 -0.018 -0.032  0.000
FEEL_ANXIOUS_Y2   0.049 -0.024  0.000  0.000

$Philippines$mean
    DEPRESSED_Y2      INTEREST_Y2 CONTROL_WORRY_Y2  FEEL_ANXIOUS_Y2
               0                0                0                0

$Philippines$th
    DEPRESSED_Y2|t1     DEPRESSED_Y2|t2     DEPRESSED_Y2|t3      INTEREST_Y2|t1      INTEREST
              0.054              -0.055               0.000               -
0.005              -0.062               0.084              -0.039               -
0.022               0.061               0.059
 FEEL_ANXIOUS_Y2|t2  FEEL_ANXIOUS_Y2|t3
             -0.061               0.000


$Poland
$Poland$type
[1] "raw"

$Poland$cov
                 DEPRES INTERE CONTRO FEEL_A
DEPRESSED_Y2      0.000
INTEREST_Y2       0.000  0.000
CONTROL_WORRY_Y2  0.021  0.005  0.000
FEEL_ANXIOUS_Y2  -0.026 -0.011  0.000  0.000

$Poland$mean
```

```
   DEPRESSED_Y2      INTEREST_Y2 CONTROL_WORRY_Y2  FEEL_ANXIOUS_Y2
              0                0                0                0

$Poland$th
   DEPRESSED_Y2|t1     DEPRESSED_Y2|t2     DEPRESSED_Y2|t3      INTEREST_Y2|t1      INTEREST
             0.008              -0.019               0.001                -
0.007              0.015              -0.004              0.043              -
0.058             -0.109              -0.036
 FEEL_ANXIOUS_Y2|t2  FEEL_ANXIOUS_Y2|t3
             0.058               0.068


$`South Africa`
$`South Africa`$type
[1] "raw"

$`South Africa`$cov
                 DEPRES INTERE CONTRO FEEL_A
DEPRESSED_Y2      0.000
INTEREST_Y2       0.000  0.000
CONTROL_WORRY_Y2 -0.006 -0.004  0.000
FEEL_ANXIOUS_Y2   0.003  0.011  0.000  0.000

$`South Africa`$mean
   DEPRESSED_Y2      INTEREST_Y2 CONTROL_WORRY_Y2  FEEL_ANXIOUS_Y2
              0                0                0                0

$`South Africa`$th
   DEPRESSED_Y2|t1     DEPRESSED_Y2|t2     DEPRESSED_Y2|t3      INTEREST_Y2|t1      INTEREST
             0.002              -0.012               0.014                -
0.017              0.047              -0.042             -0.004              -
0.008              0.015               0.016
 FEEL_ANXIOUS_Y2|t2  FEEL_ANXIOUS_Y2|t3
            -0.029               0.017


$Spain
$Spain$type
[1] "raw"

$Spain$cov
                 DEPRES INTERE CONTRO FEEL_A
DEPRESSED_Y2      0.000
```

```
INTEREST_Y2       0.000  0.000
CONTROL_WORRY_Y2  0.004  0.008  0.000
FEEL_ANXIOUS_Y2  -0.002 -0.012  0.000  0.000

$Spain$mean
    DEPRESSED_Y2      INTEREST_Y2 CONTROL_WORRY_Y2  FEEL_ANXIOUS_Y2
               0                0                0                0

$Spain$th
    DEPRESSED_Y2|t1     DEPRESSED_Y2|t2     DEPRESSED_Y2|t3      INTEREST_Y2|t1      INTEREST
              0.004               0.004              -0.012                   -
0.015               0.028              -0.019               0.030                   -
0.024              -0.015              -0.038
 FEEL_ANXIOUS_Y2|t2  FEEL_ANXIOUS_Y2|t3
              0.041              -0.008


$Tanzania
$Tanzania$type
[1] "raw"

$Tanzania$cov
                 DEPRES INTERE CONTRO FEEL_A
DEPRESSED_Y2      0.000
INTEREST_Y2       0.000  0.000
CONTROL_WORRY_Y2 -0.007  0.001  0.000
FEEL_ANXIOUS_Y2   0.011 -0.014  0.000  0.000

$Tanzania$mean
    DEPRESSED_Y2      INTEREST_Y2 CONTROL_WORRY_Y2  FEEL_ANXIOUS_Y2
               0                0                0                0

$Tanzania$th
    DEPRESSED_Y2|t1     DEPRESSED_Y2|t2     DEPRESSED_Y2|t3      INTEREST_Y2|t1      INTEREST
              0.014              -0.014               0.000                   -
0.018               0.018               0.000              -0.024               0.026
 FEEL_ANXIOUS_Y2|t2  FEEL_ANXIOUS_Y2|t3
             -0.021               0.000


$Turkey
$Turkey$type
[1] "raw"
```

```
$Turkey$cov
                DEPRES INTERE CONTRO FEEL_A
DEPRESSED_Y2     0.000
INTEREST_Y2      0.000  0.000
CONTROL_WORRY_Y2 -0.009 -0.002  0.000
FEEL_ANXIOUS_Y2  -0.002  0.016  0.000  0.000

$Turkey$mean
    DEPRESSED_Y2      INTEREST_Y2 CONTROL_WORRY_Y2  FEEL_ANXIOUS_Y2
               0                0                0                0

$Turkey$th
    DEPRESSED_Y2|t1     DEPRESSED_Y2|t2     DEPRESSED_Y2|t3      INTEREST_Y2|t1      INTEREST
             -0.008              -0.063               0.090               0.052
0.053               0.003               -0.016               -0.024               0.058
 FEEL_ANXIOUS_Y2|t2  FEEL_ANXIOUS_Y2|t3
             -0.055               0.020


$`United States`
$`United States`$type
[1] "raw"

$`United States`$cov
                DEPRES INTERE CONTRO FEEL_A
DEPRESSED_Y2     0.000
INTEREST_Y2      0.000  0.000
CONTROL_WORRY_Y2 -0.004  0.013  0.000
FEEL_ANXIOUS_Y2  -0.005  0.003  0.000  0.000

$`United States`$mean
    DEPRESSED_Y2      INTEREST_Y2 CONTROL_WORRY_Y2  FEEL_ANXIOUS_Y2
               0                0                0                0

$`United States`$th
    DEPRESSED_Y2|t1     DEPRESSED_Y2|t2     DEPRESSED_Y2|t3      INTEREST_Y2|t1      INTEREST
             -0.024               0.033               0.022               0.013
0.065              -0.001               0.028               -0.049               -
0.009
 FEEL_ANXIOUS_Y2|t2  FEEL_ANXIOUS_Y2|t3
              0.020              -0.009
```

```
$`Hong Kong`
$`Hong Kong`$type
[1] "raw"

$`Hong Kong`$cov
                 DEPRES INTERE CONTRO FEEL_A
DEPRESSED_Y2      0.000
INTEREST_Y2       0.000  0.000
CONTROL_WORRY_Y2  0.015 -0.035  0.000
FEEL_ANXIOUS_Y2   0.002 -0.050  0.000  0.000

$`Hong Kong`$mean
    DEPRESSED_Y2      INTEREST_Y2 CONTROL_WORRY_Y2  FEEL_ANXIOUS_Y2
               0                0                0                0

$`Hong Kong`$th
    DEPRESSED_Y2|t1     DEPRESSED_Y2|t2     DEPRESSED_Y2|t3      INTEREST_Y2|t1      INTEREST
              0.089              -0.102               0.000                 -
0.062                 -0.027                0.167                0.048                 -
0.061                  0.000               -0.025
 FEEL_ANXIOUS_Y2|t2  FEEL_ANXIOUS_Y2|t3
             -0.016               0.100
```

```r
## identify which pairs of correlations (items within the same factr) have largest residuals
## same factor item rescor
fit.res.cor0 <- lapply(residuals(fit.metric), \(x) data.frame(DEPRESS = x$cov[2,1], ANX=x$co
fit.res.cor <- fit.res.cor0 |> bind_rows() |> as.data.frame()
rownames(fit.res.cor) <- paste0(names(fit.res.cor0), " (", 1:length(names(fit.res.cor0)), ")"
fit.res.cor #|> mutate(across(everything(), ~round(.,2)))
```

```
                          DEPRESS           ANX
Sweden (1)          -6.325246e-04  8.021178e-05
United Kingdom (2)   3.426883e-06 -6.566824e-07
Germany (3)          4.381306e-06 -1.802459e-05
China (4)            8.030347e-06  1.236484e-06
Argentina (5)       -7.114621e-06  2.009582e-06
Australia (6)        2.308530e-06  2.287873e-06
Brazil (7)           1.303293e-06 -8.252728e-07
Egypt (8)            2.686466e-05 -1.262557e-05
India (9)            5.052292e-05 -8.423313e-06
Indonesia (10)       2.987899e-05  7.159464e-07
```

Israel (11)	-2.267338e-05	6.316134e-06
Japan (12)	-8.875708e-08	-4.057255e-07
Kenya (13)	2.418550e-06	3.804180e-06
Mexico (14)	1.680052e-05	8.150453e-07
Nigeria (15)	4.453965e-05	-4.476163e-05
Philippines (16)	3.134651e-05	1.090393e-05
Poland (17)	8.209925e-06	-2.492574e-07
South Africa (18)	7.976064e-05	-2.771396e-05
Spain (19)	2.607833e-05	7.538354e-06
Tanzania (20)	8.379747e-05	-1.842326e-04
Turkey (21)	1.768660e-04	-2.165513e-05
United States (22)	1.853053e-07	1.899777e-08
Hong Kong (23)	8.101316e-05	3.188586e-05

4.2.6 Scalar Invariance Model

```
mod.scalar <- semTools::measEq.syntax(
  configural.model = mod
  , data = df.cc
  , group = "COUNTRY"
  , parameterization = "theta"
  , ID.fac = "std.lv"
  , ID.cat = "Wu.Estabrook.2016"
  , ordered = TRUE
  , group.equal = c("thresholds","loadings", "intercepts")
  , group.partial = c(
    'DEPRESSED_Y2 | t3',
    'INTEREST_Y2 | t3',
    'CONTROL_WORRY_Y2 | t3',
    'FEEL_ANXIOUS_Y2 | t3'
  )
)

summary(mod.scalar)
```

```
This lavaan model syntax specifies a CFA with 4 manifest indicators (4 of which are ordinal)

To identify the location and scale of each common factor, the factor means and variances were

The location and scale of each latent item-response underlying 4 ordinal indicators were iden

    https://doi.org/10.1007/s11336-016-9506-0

Pattern matrix indicating num(eric), ord(ered), and lat(ent) indicators per factor:

                 dep anx
DEPRESSED_Y2     ord
INTEREST_Y2      ord
CONTROL_WORRY_Y2     ord
FEEL_ANXIOUS_Y2      ord

The following types of parameter were constrained to equality across groups:

    thresholds, with the exception of:
```

```
                              lhs op rhs
row-1:        DEPRESSED_Y2  |  t3
row-2:         INTEREST_Y2  |  t3
row-3:    CONTROL_WORRY_Y2  |  t3
row-4:     FEEL_ANXIOUS_Y2  |  t3

loadings
intercepts
```

```
#cat(as.character(mod.scalar))

mod.scalar <- "
## LOADINGS:

dep =~ c(NA, NA, NA, NA, NA, NA, NA, NA, NA, NA, NA, NA, NA, NA, NA, NA, NA, NA, NA, NA, NA,
dep =~ c(NA, NA, NA, NA, NA, NA, NA, NA, NA, NA, NA, NA, NA, NA, NA, NA, NA, NA, NA, NA, NA,
anx =~ c(NA, NA, NA, NA, NA, NA, NA, NA, NA, NA, NA, NA, NA, NA, NA, NA, NA, NA, NA, NA, NA,
anx =~ c(NA, NA, NA, NA, NA, NA, NA, NA, NA, NA, NA, NA, NA, NA, NA, NA, NA, NA, NA, NA, NA,

## THRESHOLDS:

DEPRESSED_Y2 | c(NA, NA, NA, NA, NA, NA, NA, NA, NA, NA, NA, NA, NA, NA, NA, NA, NA, NA, NA,
DEPRESSED_Y2 | c(NA, NA, NA, NA, NA, NA, NA, NA, NA, NA, NA, NA, NA, NA, NA, NA, NA, NA, NA,
DEPRESSED_Y2 | c(NA, NA, NA, NA, NA, NA, NA, NA, NA, NA, NA, NA, NA, NA, NA, NA, NA, NA, NA,
INTEREST_Y2 | c(NA, NA, NA, NA, NA, NA, NA, NA, NA, NA, NA, NA, NA, NA, NA, NA, NA, NA, NA, N
INTEREST_Y2 | c(NA, NA, NA, NA, NA, NA, NA, NA, NA, NA, NA, NA, NA, NA, NA, NA, NA, NA, NA, N
INTEREST_Y2 | c(NA, NA, NA, NA, NA, NA, NA, NA, NA, NA, NA, NA, NA, NA, NA, NA, NA, NA, NA, N
CONTROL_WORRY_Y2 | c(NA, NA, NA, NA, NA, NA, NA, NA, NA, NA, NA, NA, NA, NA, NA, NA, NA, NA,
CONTROL_WORRY_Y2 | c(NA, NA, NA, NA, NA, NA, NA, NA, NA, NA, NA, NA, NA, NA, NA, NA, NA, NA,
CONTROL_WORRY_Y2 | c(NA, NA, NA, NA, NA, NA, NA, NA, NA, NA, NA, NA, NA, NA, NA, NA, NA, NA,
FEEL_ANXIOUS_Y2 | c(NA, NA, NA, NA, NA, NA, NA, NA, NA, NA, NA, NA, NA, NA, NA, NA, NA, NA, N
FEEL_ANXIOUS_Y2 | c(NA, NA, NA, NA, NA, NA, NA, NA, NA, NA, NA, NA, NA, NA, NA, NA, NA, NA, N
FEEL_ANXIOUS_Y2 | c(NA, NA, NA, NA, NA, NA, NA, NA, NA, NA, NA, NA, NA, NA, NA, NA, NA, NA, N

## INTERCEPTS:

DEPRESSED_Y2 ~ c(0, 0, 0, 0, 0, 0, 0, 0, 0, 0, 0, 0, 0, 0, 0, 0, 0, 0, 0, 0, 0, 0, 0)*1 + c(n
INTEREST_Y2 ~ c(0, 0, 0, 0, 0, 0, 0, 0, 0, 0, 0, 0, 0, 0, 0, 0, 0, 0, 0, 0, 0, 0, 0)*1 + c(nu
CONTROL_WORRY_Y2 ~ c(0, 0, 0, 0, 0, 0, 0, 0, 0, 0, 0, 0, 0, 0, 0, 0, 0, 0, 0, 0, 0, 0, 0)*1 +
FEEL_ANXIOUS_Y2 ~ c(0, 0, 0, 0, 0, 0, 0, 0, 0, 0, 0, 0, 0, 0, 0, 0, 0, 0, 0, 0, 0, 0, 0)*1 +

## UNIQUE-FACTOR VARIANCES:
```

```
DEPRESSED_Y2 ~~ c(1, NA, NA, NA, NA, NA, NA, NA, NA, NA, NA, NA, NA, NA, NA, NA, NA, NA, NA,
INTEREST_Y2 ~~ c(1, NA, NA, NA, NA, NA, NA, NA, NA, NA, NA, NA, NA, NA, NA, NA, NA, NA, N
CONTROL_WORRY_Y2 ~~ c(1, NA, NA, NA, NA, NA, NA, NA, NA, NA, NA, NA, NA, NA, NA, NA, NA, NA,
FEEL_ANXIOUS_Y2 ~~ c(1, NA, NA, NA, NA, NA, NA, NA, NA, NA, NA, NA, NA, NA, NA, NA, NA, N

## LATENT MEANS/INTERCEPTS:

dep ~ c(0, NA, NA, NA, NA, NA, NA, NA, NA, NA, NA, NA, NA, NA, NA, NA, NA, NA, NA, NA, NA, N
anx ~ c(0, NA, NA, NA, NA, NA, NA, NA, NA, NA, NA, NA, NA, NA, NA, NA, NA, NA, NA, NA, NA, N

## COMMON-FACTOR VARIANCES:

dep ~~ c(1, NA, NA, NA, NA, NA, NA, NA, NA, NA, NA, NA, NA, NA, NA, NA, NA, NA, NA, NA, NA, N
anx ~~ c(1, NA, NA, NA, NA, NA, NA, NA, NA, NA, NA, NA, NA, NA, NA, NA, NA, NA, NA, NA, NA, N

## COMMON-FACTOR COVARIANCES:

dep ~~ c(NA, NA, NA, NA, NA, NA, NA, NA, NA, NA, NA, NA, NA, NA, NA, NA, NA, NA, NA, NA, NA,
"
fit.scalar <- cfa(
  model = as.character(mod.scalar)
  , data = df.cc
  , group = "COUNTRY"
  , ordered = TRUE
  , parameterization = "theta"
  , missing = "pairwise"
)

fit.scalar
```

```
lavaan 0.6-21 ended normally after 1717 iterations

  Estimator                                       DWLS
  Optimization method                           NLMINB
  Number of model parameters                       567
  Number of equality constraints                   338

  Number of observations per group:               Used       Total
    Sweden                                       11607       15068
    United Kingdom                                3619        5368
    Germany                                       5528        9506
```

China	4544	5022
Argentina	2928	6724
Australia	2581	3844
Brazil	4274	13203
Egypt	3040	4729
India	6372	12765
Indonesia	2681	6992
Israel	2489	3669
Japan	13968	20543
Kenya	7698	11389
Mexico	2278	5776
Nigeria	3146	6827
Philippines	2682	5292
Poland	6478	10389
South Africa	978	2651
Spain	2923	6290
Tanzania	5583	9075
Turkey	499	1473
United States	32239	38312
Hong Kong	707	3012

Number of missing patterns per group:

Sweden	13
United Kingdom	6
Germany	8
China	4
Argentina	10
Australia	7
Brazil	8
Egypt	5
India	13
Indonesia	6
Israel	9
Japan	9
Kenya	6
Mexico	8
Nigeria	9
Philippines	4
Poland	9
South Africa	4
Spain	7
Tanzania	10
Turkey	3
United States	14

```
    Hong Kong                                        3

Model Test User Model:
                                              Standard      Scaled
  Test Statistic                              4483.002    8014.899
  Degrees of freedom                               185         185
  P-value (Unknown)                                 NA       0.000
  Scaling correction factor                                  0.561
  Shift parameter                                           21.058
    simple second-order correction
  Test statistic for each group:
    Sweden                                     291.331     291.331
    United Kingdom                              74.725      74.725
    Germany                                    458.888     458.888
    China                                      238.878     238.878
    Argentina                                   87.995      87.995
    Australia                                   50.182      50.182
    Brazil                                     216.765     216.765
    Egypt                                      418.630     418.630
    India                                      898.124     898.124
    Indonesia                                  226.898     226.898
    Israel                                     229.842     229.842
    Japan                                     1317.661    1317.661
    Kenya                                      656.355     656.355
    Mexico                                      87.668      87.668
    Nigeria                                    632.157     632.157
    Philippines                                986.352     986.352
    Poland                                     143.201     143.201
    South Africa                               168.708     168.708
    Spain                                       84.512      84.512
    Tanzania                                    51.928      51.928
    Turkey                                     107.398     107.398
    United States                              499.138     499.138
    Hong Kong                                   87.564      87.564
```

```r
#### Residuals
residuals(fit.scalar)
```

```
$Sweden
$Sweden$type
[1] "raw"
```

```
$Sweden$cov
                 DEPRES INTERE CONTRO FEEL_A
DEPRESSED_Y2      0.000
INTEREST_Y2       0.000  0.000
CONTROL_WORRY_Y2  0.001 -0.021  0.000
FEEL_ANXIOUS_Y2   0.013 -0.037  0.000  0.000

$Sweden$mean
    DEPRESSED_Y2      INTEREST_Y2 CONTROL_WORRY_Y2  FEEL_ANXIOUS_Y2
               0                0                0                0

$Sweden$th
    DEPRESSED_Y2|t1     DEPRESSED_Y2|t2     DEPRESSED_Y2|t3      INTEREST_Y2|t1      INTERES
             -0.018              -0.026              -0.072               0.037
0.007              -0.055
 FEEL_ANXIOUS_Y2|t2  FEEL_ANXIOUS_Y2|t3
             -0.006              -0.017


$`United Kingdom`
$`United Kingdom`$type
[1] "raw"

$`United Kingdom`$cov
                 DEPRES INTERE CONTRO FEEL_A
DEPRESSED_Y2      0.000
INTEREST_Y2       0.000  0.000
CONTROL_WORRY_Y2  0.001  0.008  0.000
FEEL_ANXIOUS_Y2  -0.006  0.001  0.000  0.000

$`United Kingdom`$mean
    DEPRESSED_Y2      INTEREST_Y2 CONTROL_WORRY_Y2  FEEL_ANXIOUS_Y2
               0                0                0                0

$`United Kingdom`$th
    DEPRESSED_Y2|t1     DEPRESSED_Y2|t2     DEPRESSED_Y2|t3      INTEREST_Y2|t1      INTERES
             -0.037              -0.040               0.092               0.053
0.022              -0.011              -0.045              -0.041               0.002
 FEEL_ANXIOUS_Y2|t2  FEEL_ANXIOUS_Y2|t3
             -0.006               0.067


$Germany
```

```
$Germany$type
[1] "raw"

$Germany$cov
                 DEPRES INTERE CONTRO FEEL_A
DEPRESSED_Y2      0.000
INTEREST_Y2       0.000  0.000
CONTROL_WORRY_Y2  0.023  0.014  0.000
FEEL_ANXIOUS_Y2  -0.008 -0.035  0.000  0.000

$Germany$mean
    DEPRESSED_Y2      INTEREST_Y2 CONTROL_WORRY_Y2  FEEL_ANXIOUS_Y2
               0                0                0                0

$Germany$th
    DEPRESSED_Y2|t1     DEPRESSED_Y2|t2     DEPRESSED_Y2|t3      INTEREST_Y2|t1      INTEREST
             -0.158              -0.036               0.044               0.140
0.038              -0.064              -0.058              -0.057               0.052
 FEEL_ANXIOUS_Y2|t2  FEEL_ANXIOUS_Y2|t3
              0.052               0.109


$China
$China$type
[1] "raw"

$China$cov
                 DEPRES INTERE CONTRO FEEL_A
DEPRESSED_Y2      0.000
INTEREST_Y2       0.000  0.000
CONTROL_WORRY_Y2 -0.002  0.042  0.000
FEEL_ANXIOUS_Y2  -0.009 -0.012  0.000  0.000

$China$mean
    DEPRESSED_Y2      INTEREST_Y2 CONTROL_WORRY_Y2  FEEL_ANXIOUS_Y2
               0                0                0                0

$China$th
    DEPRESSED_Y2|t1     DEPRESSED_Y2|t2     DEPRESSED_Y2|t3      INTEREST_Y2|t1      INTEREST
              0.096               0.040               0.110                  -
0.113              -0.116               0.000               0.043                  -
0.061               0.004              -0.025
 FEEL_ANXIOUS_Y2|t2  FEEL_ANXIOUS_Y2|t3
```

```
            -0.003               0.093


$Argentina
$Argentina$type
[1] "raw"

$Argentina$cov
                 DEPRES INTERE CONTRO FEEL_A
DEPRESSED_Y2      0.000
INTEREST_Y2       0.000  0.000
CONTROL_WORRY_Y2  0.000  0.003  0.000
FEEL_ANXIOUS_Y2  -0.006  0.009  0.000  0.000

$Argentina$mean
    DEPRESSED_Y2      INTEREST_Y2 CONTROL_WORRY_Y2  FEEL_ANXIOUS_Y2
               0                0                0                0

$Argentina$th
    DEPRESSED_Y2|t1     DEPRESSED_Y2|t2     DEPRESSED_Y2|t3      INTEREST_Y2|t1      INTEREST
             0.084               0.026               0.022                   -
0.114              0.016               -0.077              -0.033              0.017
0.024              0.017
 FEEL_ANXIOUS_Y2|t2  FEEL_ANXIOUS_Y2|t3
             0.022              -0.003


$Australia
$Australia$type
[1] "raw"

$Australia$cov
                 DEPRES INTERE CONTRO FEEL_A
DEPRESSED_Y2      0.000
INTEREST_Y2       0.000  0.000
CONTROL_WORRY_Y2  0.011 -0.007  0.000
FEEL_ANXIOUS_Y2   0.000 -0.013  0.000  0.000

$Australia$mean
    DEPRESSED_Y2      INTEREST_Y2 CONTROL_WORRY_Y2  FEEL_ANXIOUS_Y2
               0                0                0                0

$Australia$th
```

```
    DEPRESSED_Y2|t1     DEPRESSED_Y2|t2     DEPRESSED_Y2|t3      INTEREST_Y2|t1      INTEREST
             -0.069              -0.026               0.003               0.059
0.028              -0.039               0.013              -0.041               0.021
 FEEL_ANXIOUS_Y2|t2  FEEL_ANXIOUS_Y2|t3
              0.040              -0.004


$Brazil
$Brazil$type
[1] "raw"

$Brazil$cov
                 DEPRES INTERE CONTRO FEEL_A
DEPRESSED_Y2      0.000
INTEREST_Y2       0.000  0.000
CONTROL_WORRY_Y2 -0.018  0.022  0.000
FEEL_ANXIOUS_Y2  -0.001  0.010  0.000  0.000

$Brazil$mean
    DEPRESSED_Y2      INTEREST_Y2 CONTROL_WORRY_Y2  FEEL_ANXIOUS_Y2
               0                0                0                0

$Brazil$th
    DEPRESSED_Y2|t1     DEPRESSED_Y2|t2     DEPRESSED_Y2|t3      INTEREST_Y2|t1      INTEREST
              0.099               0.043               0.095                -
0.105              -0.097              -0.066              -0.034                -
0.010               0.011               0.032
 FEEL_ANXIOUS_Y2|t2  FEEL_ANXIOUS_Y2|t3
              0.004               0.000


$Egypt
$Egypt$type
[1] "raw"

$Egypt$cov
                 DEPRES INTERE CONTRO FEEL_A
DEPRESSED_Y2      0.000
INTEREST_Y2       0.000  0.000
CONTROL_WORRY_Y2  0.005 -0.075  0.000
FEEL_ANXIOUS_Y2   0.054 -0.028  0.000  0.000

$Egypt$mean
```

```
   DEPRESSED_Y2      INTEREST_Y2 CONTROL_WORRY_Y2  FEEL_ANXIOUS_Y2
              0                0                0                0

$Egypt$th
   DEPRESSED_Y2|t1     DEPRESSED_Y2|t2     DEPRESSED_Y2|t3      INTEREST_Y2|t1      INTEREST
             0.010              -0.008              -0.102                -
0.055              0.151               0.003               0.081               0.153
0.128
 FEEL_ANXIOUS_Y2|t2  FEEL_ANXIOUS_Y2|t3
            -0.046              -0.172


$India
$India$type
[1] "raw"

$India$cov
                 DEPRES INTERE CONTRO FEEL_A
DEPRESSED_Y2      0.000
INTEREST_Y2       0.000  0.000
CONTROL_WORRY_Y2 -0.019 -0.032  0.000
FEEL_ANXIOUS_Y2   0.046 -0.083  0.000  0.000

$India$mean
   DEPRESSED_Y2      INTEREST_Y2 CONTROL_WORRY_Y2  FEEL_ANXIOUS_Y2
              0                0                0                0

$India$th
   DEPRESSED_Y2|t1     DEPRESSED_Y2|t2     DEPRESSED_Y2|t3      INTEREST_Y2|t1      INTEREST
             0.145               0.011               0.000                -
0.166             -0.136               0.056              -0.060                -
0.107              0.138               0.096
 FEEL_ANXIOUS_Y2|t2  FEEL_ANXIOUS_Y2|t3
            -0.036               0.000


$Indonesia
$Indonesia$type
[1] "raw"

$Indonesia$cov
                 DEPRES INTERE CONTRO FEEL_A
DEPRESSED_Y2      0.000
```

```
INTEREST_Y2       0.000  0.000
CONTROL_WORRY_Y2  0.001 -0.025  0.000
FEEL_ANXIOUS_Y2   0.021 -0.033  0.000  0.000

$Indonesia$mean
    DEPRESSED_Y2      INTEREST_Y2 CONTROL_WORRY_Y2  FEEL_ANXIOUS_Y2
               0                0                0                0

$Indonesia$th
    DEPRESSED_Y2|t1     DEPRESSED_Y2|t2     DEPRESSED_Y2|t3      INTEREST_Y2|t1      INTEREST
              0.105               0.073              -0.167                   -
0.160               0.080               0.000               0.018               0.080
0.048              -0.044
 FEEL_ANXIOUS_Y2|t2  FEEL_ANXIOUS_Y2|t3
              0.058              -0.086


$Israel
$Israel$type
[1] "raw"

$Israel$cov
                 DEPRES INTERE CONTRO FEEL_A
DEPRESSED_Y2      0.000
INTEREST_Y2       0.000  0.000
CONTROL_WORRY_Y2  0.026 -0.041  0.000
FEEL_ANXIOUS_Y2  -0.003 -0.001  0.000  0.000

$Israel$mean
    DEPRESSED_Y2      INTEREST_Y2 CONTROL_WORRY_Y2  FEEL_ANXIOUS_Y2
               0                0                0                0

$Israel$th
    DEPRESSED_Y2|t1     DEPRESSED_Y2|t2     DEPRESSED_Y2|t3      INTEREST_Y2|t1      INTEREST
             -0.076              -0.077               0.045               0.095
0.162              -0.044               0.188               0.139
 FEEL_ANXIOUS_Y2|t2  FEEL_ANXIOUS_Y2|t3
             -0.042               0.035


$Japan
$Japan$type
[1] "raw"
```

```
$Japan$cov
                 DEPRES INTERE CONTRO FEEL_A
DEPRESSED_Y2      0.000
INTEREST_Y2       0.000  0.000
CONTROL_WORRY_Y2 -0.014 -0.012  0.000
FEEL_ANXIOUS_Y2   0.007  0.010  0.000  0.000

$Japan$mean
    DEPRESSED_Y2      INTEREST_Y2 CONTROL_WORRY_Y2  FEEL_ANXIOUS_Y2
               0                0                0                0

$Japan$th
    DEPRESSED_Y2|t1     DEPRESSED_Y2|t2     DEPRESSED_Y2|t3      INTEREST_Y2|t1      INTEREST
             -0.001              -0.037               0.057               0.027
0.050               0.041               0.172               0.091               0.139
0.114
 FEEL_ANXIOUS_Y2|t2  FEEL_ANXIOUS_Y2|t3
             -0.151              -0.079


$Kenya
$Kenya$type
[1] "raw"

$Kenya$cov
                 DEPRES INTERE CONTRO FEEL_A
DEPRESSED_Y2      0.000
INTEREST_Y2       0.000  0.000
CONTROL_WORRY_Y2  0.024 -0.018  0.000
FEEL_ANXIOUS_Y2   0.014 -0.035  0.000  0.000

$Kenya$mean
    DEPRESSED_Y2      INTEREST_Y2 CONTROL_WORRY_Y2  FEEL_ANXIOUS_Y2
               0                0                0                0

$Kenya$th
    DEPRESSED_Y2|t1     DEPRESSED_Y2|t2     DEPRESSED_Y2|t3      INTEREST_Y2|t1      INTEREST
             -0.010               0.053              -0.104                   -
0.033               0.074               0.000              -0.184               0.000
0.066               0.111
 FEEL_ANXIOUS_Y2|t2  FEEL_ANXIOUS_Y2|t3
              0.090               0.029
```

```
$Mexico
$Mexico$type
[1] "raw"

$Mexico$cov
                 DEPRES INTERE CONTRO FEEL_A
DEPRESSED_Y2      0.000
INTEREST_Y2       0.000  0.000
CONTROL_WORRY_Y2 -0.001  0.022  0.000
FEEL_ANXIOUS_Y2  -0.003 -0.011  0.000  0.000

$Mexico$mean
    DEPRESSED_Y2      INTEREST_Y2 CONTROL_WORRY_Y2  FEEL_ANXIOUS_Y2
               0                0                0                0

$Mexico$th
    DEPRESSED_Y2|t1     DEPRESSED_Y2|t2     DEPRESSED_Y2|t3      INTEREST_Y2|t1      INTEREST
              0.015               0.001               0.030                   -
0.033               0.039               -0.067               -0.116               -
0.046              -0.019                0.089
 FEEL_ANXIOUS_Y2|t2  FEEL_ANXIOUS_Y2|t3
              0.039               0.061


$Nigeria
$Nigeria$type
[1] "raw"

$Nigeria$cov
                 DEPRES INTERE CONTRO FEEL_A
DEPRESSED_Y2      0.000
INTEREST_Y2       0.000  0.000
CONTROL_WORRY_Y2 -0.013  0.037  0.000
FEEL_ANXIOUS_Y2  -0.001 -0.015  0.000  0.000

$Nigeria$mean
    DEPRESSED_Y2      INTEREST_Y2 CONTROL_WORRY_Y2  FEEL_ANXIOUS_Y2
               0                0                0                0

$Nigeria$th
    DEPRESSED_Y2|t1     DEPRESSED_Y2|t2     DEPRESSED_Y2|t3      INTEREST_Y2|t1      INTEREST
```

```
             0.154              0.016              0.163              -
0.191              -0.118             -0.109             -0.180             -
0.090              -0.017              0.169
 FEEL_ANXIOUS_Y2|t2  FEEL_ANXIOUS_Y2|t3
            -0.010              0.160


$Philippines
$Philippines$type
[1] "raw"

$Philippines$cov
                 DEPRES INTERE CONTRO FEEL_A
DEPRESSED_Y2      0.000
INTEREST_Y2       0.000  0.000
CONTROL_WORRY_Y2 -0.024 -0.045  0.000
FEEL_ANXIOUS_Y2   0.062 -0.021  0.000  0.000

$Philippines$mean
    DEPRESSED_Y2      INTEREST_Y2 CONTROL_WORRY_Y2  FEEL_ANXIOUS_Y2
               0                0                0                0

$Philippines$th
    DEPRESSED_Y2|t1     DEPRESSED_Y2|t2     DEPRESSED_Y2|t3      INTEREST_Y2|t1      INTEREST
             0.179              0.130              0.000              -
0.202              -0.188              0.007             -0.292             -
0.155               0.003              0.202
 FEEL_ANXIOUS_Y2|t2  FEEL_ANXIOUS_Y2|t3
             0.248              0.000


$Poland
$Poland$type
[1] "raw"

$Poland$cov
                 DEPRES INTERE CONTRO FEEL_A
DEPRESSED_Y2      0.000
INTEREST_Y2       0.000  0.000
CONTROL_WORRY_Y2  0.008  0.014  0.000
FEEL_ANXIOUS_Y2  -0.032  0.004  0.000  0.000

$Poland$mean
```

```
   DEPRESSED_Y2      INTEREST_Y2 CONTROL_WORRY_Y2  FEEL_ANXIOUS_Y2
              0                0                0                0

$Poland$th
   DEPRESSED_Y2|t1     DEPRESSED_Y2|t2     DEPRESSED_Y2|t3      INTEREST_Y2|t1      INTEREST
             0.062              -0.008               0.007                   -
0.055              -0.009              -0.014               0.062              -
0.051              -0.112              -0.051
 FEEL_ANXIOUS_Y2|t2  FEEL_ANXIOUS_Y2|t3
             0.043               0.068


$`South Africa`
$`South Africa`$type
[1] "raw"

$`South Africa`$cov
                 DEPRES INTERE CONTRO FEEL_A
DEPRESSED_Y2      0.000
INTEREST_Y2       0.001  0.000
CONTROL_WORRY_Y2  0.037  0.012  0.000
FEEL_ANXIOUS_Y2  -0.023 -0.030  0.000  0.000

$`South Africa`$mean
   DEPRESSED_Y2      INTEREST_Y2 CONTROL_WORRY_Y2  FEEL_ANXIOUS_Y2
              0                0                0                0

$`South Africa`$th
   DEPRESSED_Y2|t1     DEPRESSED_Y2|t2     DEPRESSED_Y2|t3      INTEREST_Y2|t1      INTEREST
            -0.054              -0.060              -0.012               0.046
0.233              -0.143              -0.061               0.178
 FEEL_ANXIOUS_Y2|t2  FEEL_ANXIOUS_Y2|t3
             0.110               0.157


$Spain
$Spain$type
[1] "raw"

$Spain$cov
                 DEPRES INTERE CONTRO FEEL_A
DEPRESSED_Y2      0.000
INTEREST_Y2       0.000  0.000
```

```
CONTROL_WORRY_Y2 -0.001  0.001  0.000
FEEL_ANXIOUS_Y2   0.005 -0.008  0.000  0.000

$Spain$mean
    DEPRESSED_Y2      INTEREST_Y2 CONTROL_WORRY_Y2  FEEL_ANXIOUS_Y2
               0                0                0                0

$Spain$th
    DEPRESSED_Y2|t1     DEPRESSED_Y2|t2     DEPRESSED_Y2|t3      INTEREST_Y2|t1      INTEREST
              0.016              -0.002              -0.011                   -
0.026               0.027               -0.015               0.102               0.033
0.104
 FEEL_ANXIOUS_Y2|t2  FEEL_ANXIOUS_Y2|t3
             -0.024              -0.058


$Tanzania
$Tanzania$type
[1] "raw"

$Tanzania$cov
                 DEPRES INTERE CONTRO FEEL_A
DEPRESSED_Y2      0.000
INTEREST_Y2       0.000  0.000
CONTROL_WORRY_Y2 -0.004  0.005  0.000
FEEL_ANXIOUS_Y2   0.007 -0.015  0.000  0.000

$Tanzania$mean
    DEPRESSED_Y2      INTEREST_Y2 CONTROL_WORRY_Y2  FEEL_ANXIOUS_Y2
               0                0                0                0

$Tanzania$th
    DEPRESSED_Y2|t1     DEPRESSED_Y2|t2     DEPRESSED_Y2|t3      INTEREST_Y2|t1      INTEREST
             -0.009              -0.037               0.000               0.010
0.038               0.018                0.000               0.028
 FEEL_ANXIOUS_Y2|t2  FEEL_ANXIOUS_Y2|t3
             -0.009               0.000


$Turkey
$Turkey$type
[1] "raw"
```

```
$Turkey$cov
                 DEPRES INTERE CONTRO FEEL_A
DEPRESSED_Y2      0.000
INTEREST_Y2       0.000  0.000
CONTROL_WORRY_Y2 -0.040 -0.026  0.000
FEEL_ANXIOUS_Y2   0.014  0.028  0.000  0.000

$Turkey$mean
    DEPRESSED_Y2      INTEREST_Y2 CONTROL_WORRY_Y2  FEEL_ANXIOUS_Y2
               0                0                0                0

$Turkey$th
    DEPRESSED_Y2|t1     DEPRESSED_Y2|t2     DEPRESSED_Y2|t3      INTEREST_Y2|t1      INTEREST
             -0.002              -0.070               0.087               0.048
0.050               0.009               0.193               0.151               0.208
0.131
 FEEL_ANXIOUS_Y2|t2  FEEL_ANXIOUS_Y2|t3
             -0.222              -0.132


$`United States`
$`United States`$type
[1] "raw"

$`United States`$cov
                 DEPRES INTERE CONTRO FEEL_A
DEPRESSED_Y2      0.000
INTEREST_Y2       0.000  0.000
CONTROL_WORRY_Y2  0.010 -0.003  0.000
FEEL_ANXIOUS_Y2   0.003 -0.018  0.000  0.000

$`United States`$mean
    DEPRESSED_Y2      INTEREST_Y2 CONTROL_WORRY_Y2  FEEL_ANXIOUS_Y2
               0                0                0                0

$`United States`$th
    DEPRESSED_Y2|t1     DEPRESSED_Y2|t2     DEPRESSED_Y2|t3      INTEREST_Y2|t1      INTEREST
             -0.073              -0.008               0.001               0.062
0.027              -0.012               0.020              -0.057               0.000
 FEEL_ANXIOUS_Y2|t2  FEEL_ANXIOUS_Y2|t3
              0.024               0.008
```

```
$`Hong Kong`
$`Hong Kong`$type
[1] "raw"

$`Hong Kong`$cov
                 DEPRES INTERE CONTRO FEEL_A
DEPRESSED_Y2      0.000
INTEREST_Y2       0.000  0.000
CONTROL_WORRY_Y2  0.014 -0.032  0.000
FEEL_ANXIOUS_Y2   0.002 -0.047  0.000  0.000

$`Hong Kong`$mean
    DEPRESSED_Y2      INTEREST_Y2 CONTROL_WORRY_Y2  FEEL_ANXIOUS_Y2
               0                0                0                0

$`Hong Kong`$th
    DEPRESSED_Y2|t1     DEPRESSED_Y2|t2     DEPRESSED_Y2|t3      INTEREST_Y2|t1      INTEREST
              0.181               0.007               0.000                   -
0.228              -0.130              0.104              0.057               -
0.055               0.000             -0.033
 FEEL_ANXIOUS_Y2|t2  FEEL_ANXIOUS_Y2|t3
             -0.028               0.104
```

```
## identify which pairs of correlations (items within the same factr) have largest residuals
## same factor item rescor
fit.res.cor0 <- lapply(residuals(fit.scalar), \(x) data.frame(DEPRESS = x$cov[2,1], ANX=x$cov
fit.res.cor <- fit.res.cor0 |> bind_rows() |> as.data.frame()
rownames(fit.res.cor) <- paste0(names(fit.res.cor0), " (", 1:length(names(fit.res.cor0)), ")"
fit.res.cor #|> mutate(across(everything(), ~round(.,2)))
```

```
                         DEPRESS           ANX
Sweden (1)         -1.325120e-04  1.861820e-06
United Kingdom (2)  7.328099e-07  1.363651e-06
Germany (3)         1.685685e-06  6.337867e-06
China (4)           8.312900e-07  1.069608e-06
Argentina (5)       2.674380e-05 -3.466722e-05
Australia (6)      -2.493032e-06  2.763891e-06
Brazil (7)          5.374889e-06 -7.270129e-06
Egypt (8)           7.308517e-05 -5.823201e-06
India (9)          -3.901486e-05 -1.420966e-05
Indonesia (10)      4.045523e-05  2.804949e-05
Israel (11)         1.706749e-06 -2.449710e-06
```

Japan (12)	-4.470905e-08	-7.228998e-08
Kenya (13)	4.942008e-06	-6.068164e-06
Mexico (14)	1.961333e-06	8.076772e-06
Nigeria (15)	9.879651e-06	-4.612385e-05
Philippines (16)	-1.188783e-04	3.704020e-05
Poland (17)	4.434515e-07	2.483007e-06
South Africa (18)	7.008936e-04	-5.468614e-05
Spain (19)	-3.695377e-06	2.912375e-05
Tanzania (20)	-1.180213e-04	2.067985e-05
Turkey (21)	2.929232e-04	-1.183088e-04
United States (22)	6.638926e-08	1.781706e-07
Hong Kong (23)	-5.466957e-05	3.457035e-05

4.2.7 Strict Invariance Model

```r
mod.strict <- semTools::measEq.syntax(
  configural.model = mod
  , data = df.cc
  , group = "COUNTRY"
  , parameterization = "theta"
  , ID.fac = "std.lv"
  , ID.cat = "Wu.Estabrook.2016"
  , ordered = TRUE
  , group.equal = c("thresholds","loadings", "intercepts", "residuals")
  , group.partial = c(
    'DEPRESSED_Y2 | t3',
    'INTEREST_Y2 | t3',
    'CONTROL_WORRY_Y2 | t3',
    'FEEL_ANXIOUS_Y2 | t3'
  )
)

summary(mod.strict)
```

```
This lavaan model syntax specifies a CFA with 4 manifest indicators (4 of which are ordinal)

To identify the location and scale of each common factor, the factor means and variances were

The location and scale of each latent item-response underlying 4 ordinal indicators were iden

    https://doi.org/10.1007/s11336-016-9506-0

Pattern matrix indicating num(eric), ord(ered), and lat(ent) indicators per factor:

                 dep anx
DEPRESSED_Y2     ord
INTEREST_Y2      ord
CONTROL_WORRY_Y2     ord
FEEL_ANXIOUS_Y2      ord

The following types of parameter were constrained to equality across groups:

    thresholds, with the exception of:
```

```
                              lhs op rhs
        row-1:        DEPRESSED_Y2  |  t3
        row-2:         INTEREST_Y2  |  t3
        row-3:    CONTROL_WORRY_Y2  |  t3
        row-4:     FEEL_ANXIOUS_Y2  |  t3

    loadings
    intercepts
    residuals
```

```
#cat(as.character(mod.strict))
mod.strict <- "
## LOADINGS:

dep =~ c(NA, NA, NA, NA, NA, NA, NA, NA, NA, NA, NA, NA, NA, NA, NA, NA, NA, NA, NA, NA, NA,
dep =~ c(NA, NA, NA, NA, NA, NA, NA, NA, NA, NA, NA, NA, NA, NA, NA, NA, NA, NA, NA, NA, NA,
anx =~ c(NA, NA, NA, NA, NA, NA, NA, NA, NA, NA, NA, NA, NA, NA, NA, NA, NA, NA, NA, NA, NA,
anx =~ c(NA, NA, NA, NA, NA, NA, NA, NA, NA, NA, NA, NA, NA, NA, NA, NA, NA, NA, NA, NA, NA,

## THRESHOLDS:

DEPRESSED_Y2 | c(NA, NA, NA, NA, NA, NA, NA, NA, NA, NA, NA, NA, NA, NA, NA, NA, NA, NA, NA,
DEPRESSED_Y2 | c(NA, NA, NA, NA, NA, NA, NA, NA, NA, NA, NA, NA, NA, NA, NA, NA, NA, NA, NA,
DEPRESSED_Y2 | c(NA, NA, NA, NA, NA, NA, NA, NA, NA, NA, NA, NA, NA, NA, NA, NA, NA, NA, NA,
INTEREST_Y2 | c(NA, NA, NA, NA, NA, NA, NA, NA, NA, NA, NA, NA, NA, NA, NA, NA, NA, NA, NA, N
INTEREST_Y2 | c(NA, NA, NA, NA, NA, NA, NA, NA, NA, NA, NA, NA, NA, NA, NA, NA, NA, NA, NA, N
INTEREST_Y2 | c(NA, NA, NA, NA, NA, NA, NA, NA, NA, NA, NA, NA, NA, NA, NA, NA, NA, NA, NA, N
CONTROL_WORRY_Y2 | c(NA, NA, NA, NA, NA, NA, NA, NA, NA, NA, NA, NA, NA, NA, NA, NA, NA, NA,
CONTROL_WORRY_Y2 | c(NA, NA, NA, NA, NA, NA, NA, NA, NA, NA, NA, NA, NA, NA, NA, NA, NA, NA,
CONTROL_WORRY_Y2 | c(NA, NA, NA, NA, NA, NA, NA, NA, NA, NA, NA, NA, NA, NA, NA, NA, NA, NA,
FEEL_ANXIOUS_Y2 | c(NA, NA, NA, NA, NA, NA, NA, NA, NA, NA, NA, NA, NA, NA, NA, NA, NA, NA, N
FEEL_ANXIOUS_Y2 | c(NA, NA, NA, NA, NA, NA, NA, NA, NA, NA, NA, NA, NA, NA, NA, NA, NA, NA, N
FEEL_ANXIOUS_Y2 | c(NA, NA, NA, NA, NA, NA, NA, NA, NA, NA, NA, NA, NA, NA, NA, NA, NA, NA, N

## INTERCEPTS:

DEPRESSED_Y2 ~ c(0, 0, 0, 0, 0, 0, 0, 0, 0, 0, 0, 0, 0, 0, 0, 0, 0, 0, 0, 0, 0, 0, 0)*1 + c(n
INTEREST_Y2 ~ c(0, 0, 0, 0, 0, 0, 0, 0, 0, 0, 0, 0, 0, 0, 0, 0, 0, 0, 0, 0, 0, 0, 0)*1 + c(nu
CONTROL_WORRY_Y2 ~ c(0, 0, 0, 0, 0, 0, 0, 0, 0, 0, 0, 0, 0, 0, 0, 0, 0, 0, 0, 0, 0, 0, 0)*1 +
FEEL_ANXIOUS_Y2 ~ c(0, 0, 0, 0, 0, 0, 0, 0, 0, 0, 0, 0, 0, 0, 0, 0, 0, 0, 0, 0, 0, 0, 0)*1 +

## UNIQUE-FACTOR VARIANCES:
```

```
DEPRESSED_Y2 ~~ c(1, 1, 1, 1, 1, 1, 1, 1, 1, 1, 1, 1, 1, 1, 1, 1, 1, 1, 1, 1, 1, 1, 1)*DEPRES
INTEREST_Y2 ~~ c(1, 1, 1, 1, 1, 1, 1, 1, 1, 1, 1, 1, 1, 1, 1, 1, 1, 1, 1, 1, 1, 1, 1)*INTERES
CONTROL_WORRY_Y2 ~~ c(1, 1, 1, 1, 1, 1, 1, 1, 1, 1, 1, 1, 1, 1, 1, 1, 1, 1, 1, 1, 1, 1, 1)*CO
FEEL_ANXIOUS_Y2 ~~ c(1, 1, 1, 1, 1, 1, 1, 1, 1, 1, 1, 1, 1, 1, 1, 1, 1, 1, 1, 1, 1, 1, 1)*FEI

## LATENT MEANS/INTERCEPTS:

dep ~ c(0, NA, NA, NA, NA, NA, NA, NA, NA, NA, NA, NA, NA, NA, NA, NA, NA, NA, NA, NA, NA, N
anx ~ c(0, NA, NA, NA, NA, NA, NA, NA, NA, NA, NA, NA, NA, NA, NA, NA, NA, NA, NA, NA, NA, N

## COMMON-FACTOR VARIANCES:

dep ~~ c(1, NA, NA, NA, NA, NA, NA, NA, NA, NA, NA, NA, NA, NA, NA, NA, NA, NA, NA, NA, NA, N
anx ~~ c(1, NA, NA, NA, NA, NA, NA, NA, NA, NA, NA, NA, NA, NA, NA, NA, NA, NA, NA, NA, NA, N

## COMMON-FACTOR COVARIANCES:

dep ~~ c(NA, NA, NA, NA, NA, NA, NA, NA, NA, NA, NA, NA, NA, NA, NA, NA, NA, NA, NA, NA, NA,
"


fit.strict <- cfa(
  model = as.character(mod.strict)
  , data = df.cc
  , group = "COUNTRY"
  , ordered = TRUE
  , parameterization = "theta"
  , missing = "pairwise"
)

fit.strict
```

```
lavaan 0.6-21 ended normally after 602 iterations

  Estimator                                         DWLS
  Optimization method                             NLMINB
  Number of model parameters                         479
  Number of equality constraints                     338

  Number of observations per group:               Used       Total
    Sweden                                       11607       15068
```

```
  United Kingdom                                       3619        5368
  Germany                                              5528        9506
  China                                                4544        5022
  Argentina                                            2928        6724
  Australia                                            2581        3844
  Brazil                                               4274       13203
  Egypt                                                3040        4729
  India                                                6372       12765
  Indonesia                                            2681        6992
  Israel                                               2489        3669
  Japan                                               13968       20543
  Kenya                                                7698       11389
  Mexico                                               2278        5776
  Nigeria                                              3146        6827
  Philippines                                          2682        5292
  Poland                                               6478       10389
  South Africa                                          978        2651
  Spain                                                2923        6290
  Tanzania                                             5583        9075
  Turkey                                                499        1473
  United States                                       32239       38312
  Hong Kong                                             707        3012
Number of missing patterns per group:
  Sweden                                                 13
  United Kingdom                                          6
  Germany                                                 8
  China                                                   4
  Argentina                                              10
  Australia                                               7
  Brazil                                                  8
  Egypt                                                   5
  India                                                  13
  Indonesia                                               6
  Israel                                                  9
  Japan                                                   9
  Kenya                                                   6
  Mexico                                                  8
  Nigeria                                                 9
  Philippines                                             4
  Poland                                                  9
  South Africa                                            4
  Spain                                                   7
  Tanzania                                               10
```

```
    Turkey                                         3
    United States                                 14
    Hong Kong                                      3

Model Test User Model:
                                              Standard      Scaled
  Test Statistic                             17240.131   29765.275
  Degrees of freedom                               273         273
  P-value (Unknown)                                 NA       0.000
  Scaling correction factor                                  0.580
  Shift parameter                                           31.059
    simple second-order correction
  Test statistic for each group:
    Sweden                                   1552.858    1552.858
    United Kingdom                            242.631     242.631
    Germany                                   823.895     823.895
    China                                     641.161     641.161
    Argentina                                 156.895     156.895
    Australia                                 213.786     213.786
    Brazil                                    462.248     462.248
    Egypt                                     991.282     991.282
    India                                    5538.998    5538.998
    Indonesia                                 329.990     329.990
    Israel                                    411.968     411.968
    Japan                                    2248.470    2248.470
    Kenya                                    2588.792    2588.792
    Mexico                                    231.129     231.129
    Nigeria                                  1780.996    1780.996
    Philippines                              2254.471    2254.471
    Poland                                    408.791     408.791
    South Africa                              537.071     537.071
    Spain                                     178.427     178.427
    Tanzania                                 4827.496    4827.496
    Turkey                                    187.715     187.715
    United States                            2919.554    2919.554
    Hong Kong                                 236.650     236.650
```

```r
#### Residuals
residuals(fit.strict)
```

$Sweden
$Sweden$type

```
[1] "raw"

$Sweden$cov
                 DEPRES INTERE CONTRO FEEL_A
DEPRESSED_Y2      0.000
INTEREST_Y2      -0.053  0.000
CONTROL_WORRY_Y2  0.033 -0.066  0.000
FEEL_ANXIOUS_Y2   0.014 -0.104  0.033  0.000

$Sweden$mean
    DEPRESSED_Y2      INTEREST_Y2 CONTROL_WORRY_Y2  FEEL_ANXIOUS_Y2
               0                0                0                0

$Sweden$th
    DEPRESSED_Y2|t1     DEPRESSED_Y2|t2     DEPRESSED_Y2|t3      INTEREST_Y2|t1      INTEREST
              0.041              -0.019              -0.103               0.109
0.080              -0.064               0.020               0.077               0.155
0.140
 FEEL_ANXIOUS_Y2|t2  FEEL_ANXIOUS_Y2|t3
              0.005               0.039


$`United Kingdom`
$`United Kingdom`$type
[1] "raw"

$`United Kingdom`$cov
                 DEPRES INTERE CONTRO FEEL_A
DEPRESSED_Y2      0.000
INTEREST_Y2       0.032  0.000
CONTROL_WORRY_Y2  0.007  0.013  0.000
FEEL_ANXIOUS_Y2  -0.013 -0.005  0.019  0.000

$`United Kingdom`$mean
    DEPRESSED_Y2      INTEREST_Y2 CONTROL_WORRY_Y2  FEEL_ANXIOUS_Y2
               0                0                0                0

$`United Kingdom`$th
    DEPRESSED_Y2|t1     DEPRESSED_Y2|t2     DEPRESSED_Y2|t3      INTEREST_Y2|t1      INTEREST
             -0.104              -0.009               0.169                  -
0.011               0.008               0.096              -0.073                  -
0.011               0.085               0.002
 FEEL_ANXIOUS_Y2|t2  FEEL_ANXIOUS_Y2|t3
```

```
            -0.004              0.089


$Germany
$Germany$type
[1] "raw"

$Germany$cov
                 DEPRES INTERE CONTRO FEEL_A
DEPRESSED_Y2      0.000
INTEREST_Y2       0.026  0.000
CONTROL_WORRY_Y2 -0.015  0.033  0.000
FEEL_ANXIOUS_Y2  -0.015  0.012 -0.068  0.000

$Germany$mean
    DEPRESSED_Y2      INTEREST_Y2 CONTROL_WORRY_Y2  FEEL_ANXIOUS_Y2
               0                0                0                0

$Germany$th
    DEPRESSED_Y2|t1     DEPRESSED_Y2|t2     DEPRESSED_Y2|t3      INTEREST_Y2|t1      INTEREST
            -0.217              -0.038               0.067               0.096
0.024              -0.095              -0.123               0.099
 FEEL_ANXIOUS_Y2|t2  FEEL_ANXIOUS_Y2|t3
             0.042               0.063


$China
$China$type
[1] "raw"

$China$cov
                 DEPRES INTERE CONTRO FEEL_A
DEPRESSED_Y2      0.000
INTEREST_Y2       0.048  0.000
CONTROL_WORRY_Y2 -0.006  0.022  0.000
FEEL_ANXIOUS_Y2   0.005 -0.020  0.077  0.000

$China$mean
    DEPRESSED_Y2      INTEREST_Y2 CONTROL_WORRY_Y2  FEEL_ANXIOUS_Y2
               0                0                0                0

$China$th
    DEPRESSED_Y2|t1     DEPRESSED_Y2|t2     DEPRESSED_Y2|t3      INTEREST_Y2|t1      INTEREST
```

```
             0.057              0.086              0.192              -
0.121              -0.103              0.000              -0.021              0.022
0.145
 FEEL_ANXIOUS_Y2|t2  FEEL_ANXIOUS_Y2|t3
             0.078              0.271


$Argentina
$Argentina$type
[1] "raw"

$Argentina$cov
                 DEPRES INTERE CONTRO FEEL_A
DEPRESSED_Y2      0.000
INTEREST_Y2       0.021  0.000
CONTROL_WORRY_Y2 -0.010 -0.011  0.000
FEEL_ANXIOUS_Y2   0.007  0.014 -0.047  0.000

$Argentina$mean
    DEPRESSED_Y2      INTEREST_Y2 CONTROL_WORRY_Y2  FEEL_ANXIOUS_Y2
               0                0                0                0

$Argentina$th
    DEPRESSED_Y2|t1     DEPRESSED_Y2|t2     DEPRESSED_Y2|t3      INTEREST_Y2|t1      INTEREST
             0.054              0.037              0.048              -
0.131               0.024              -0.040              0.009              -
0.005              -0.070               0.060
 FEEL_ANXIOUS_Y2|t2  FEEL_ANXIOUS_Y2|t3
             0.012             -0.048


$Australia
$Australia$type
[1] "raw"

$Australia$cov
                 DEPRES INTERE CONTRO FEEL_A
DEPRESSED_Y2      0.000
INTEREST_Y2       0.047  0.000
CONTROL_WORRY_Y2  0.011 -0.003  0.000
FEEL_ANXIOUS_Y2  -0.003 -0.013  0.031  0.000

$Australia$mean
```

```
   DEPRESSED_Y2      INTEREST_Y2 CONTROL_WORRY_Y2  FEEL_ANXIOUS_Y2
              0                0                0                0

$Australia$th
   DEPRESSED_Y2|t1     DEPRESSED_Y2|t2     DEPRESSED_Y2|t3      INTEREST_Y2|t1      INTERES
            -0.142               0.019               0.106                   -
0.011              0.117               0.115              -0.068              0.048
0.033
 FEEL_ANXIOUS_Y2|t2  FEEL_ANXIOUS_Y2|t3
             0.052               0.035


$Brazil
$Brazil$type
[1] "raw"

$Brazil$cov
                 DEPRES INTERE CONTRO FEEL_A
DEPRESSED_Y2      0.000
INTEREST_Y2      -0.033  0.000
CONTROL_WORRY_Y2 -0.023 -0.006  0.000
FEEL_ANXIOUS_Y2   0.021  0.003 -0.063  0.000

$Brazil$mean
   DEPRESSED_Y2      INTEREST_Y2 CONTROL_WORRY_Y2  FEEL_ANXIOUS_Y2
              0                0                0                0

$Brazil$th
   DEPRESSED_Y2|t1     DEPRESSED_Y2|t2     DEPRESSED_Y2|t3      INTEREST_Y2|t1      INTERES
             0.131               0.039               0.064                   -
0.047             -0.113              -0.125               0.034                   -
0.045             -0.072               0.103
 FEEL_ANXIOUS_Y2|t2  FEEL_ANXIOUS_Y2|t3
            -0.026               0.000


$Egypt
$Egypt$type
[1] "raw"

$Egypt$cov
                 DEPRES INTERE CONTRO FEEL_A
DEPRESSED_Y2      0.000
```

```
INTEREST_Y2      -0.186  0.000
CONTROL_WORRY_Y2  0.025 -0.051  0.000
FEEL_ANXIOUS_Y2   0.025 -0.044 -0.098  0.000

$Egypt$mean
    DEPRESSED_Y2      INTEREST_Y2 CONTROL_WORRY_Y2  FEEL_ANXIOUS_Y2
               0                0                0                0

$Egypt$th
    DEPRESSED_Y2|t1     DEPRESSED_Y2|t2     DEPRESSED_Y2|t3      INTEREST_Y2|t1      INTEREST
              0.086              -0.040              -0.203               0.031
0.063               0.123               0.163               0.086               -
0.020
 FEEL_ANXIOUS_Y2|t2  FEEL_ANXIOUS_Y2|t3
             -0.071              -0.274


$India
$India$type
[1] "raw"

$India$cov
                 DEPRES INTERE CONTRO FEEL_A
DEPRESSED_Y2      0.000
INTEREST_Y2      -0.398  0.000
CONTROL_WORRY_Y2  0.017 -0.122  0.000
FEEL_ANXIOUS_Y2   0.097 -0.163 -0.280  0.000

$India$mean
    DEPRESSED_Y2      INTEREST_Y2 CONTROL_WORRY_Y2  FEEL_ANXIOUS_Y2
               0                0                0                0

$India$th
    DEPRESSED_Y2|t1     DEPRESSED_Y2|t2     DEPRESSED_Y2|t3      INTEREST_Y2|t1      INTEREST
              0.254               0.010               0.000               0.083
0.193              -0.210               0.027              -0.165               0.004
 FEEL_ANXIOUS_Y2|t2  FEEL_ANXIOUS_Y2|t3
             -0.084               0.000


$Indonesia
$Indonesia$type
[1] "raw"
```

```
$Indonesia$cov
                 DEPRES INTERE CONTRO FEEL_A
DEPRESSED_Y2      0.000
INTEREST_Y2      -0.079  0.000
CONTROL_WORRY_Y2 -0.006 -0.024  0.000
FEEL_ANXIOUS_Y2   0.023 -0.025 -0.048  0.000

$Indonesia$mean
    DEPRESSED_Y2      INTEREST_Y2 CONTROL_WORRY_Y2  FEEL_ANXIOUS_Y2
               0                0                0                0

$Indonesia$th
    DEPRESSED_Y2|t1     DEPRESSED_Y2|t2     DEPRESSED_Y2|t3      INTEREST_Y2|t1      INTEREST
              0.133               0.038              -0.247                   -
0.116               0.070               0.000               0.049               0.056
0.088              -0.002
 FEEL_ANXIOUS_Y2|t2  FEEL_ANXIOUS_Y2|t3
              0.038              -0.145


$Israel
$Israel$type
[1] "raw"

$Israel$cov
                 DEPRES INTERE CONTRO FEEL_A
DEPRESSED_Y2      0.000
INTEREST_Y2       0.015  0.000
CONTROL_WORRY_Y2 -0.054 -0.091  0.000
FEEL_ANXIOUS_Y2   0.030  0.048 -0.008  0.000

$Israel$mean
    DEPRESSED_Y2      INTEREST_Y2 CONTROL_WORRY_Y2  FEEL_ANXIOUS_Y2
               0                0                0                0

$Israel$th
    DEPRESSED_Y2|t1     DEPRESSED_Y2|t2     DEPRESSED_Y2|t3      INTEREST_Y2|t1      INTEREST
             -0.109              -0.072               0.063               0.075
0.174              -0.127               0.079               0.178
 FEEL_ANXIOUS_Y2|t2  FEEL_ANXIOUS_Y2|t3
              0.044               0.139
```

```
$Japan
$Japan$type
[1] "raw"

$Japan$cov
                 DEPRES INTERE CONTRO FEEL_A
DEPRESSED_Y2      0.000
INTEREST_Y2       0.039  0.000
CONTROL_WORRY_Y2 -0.019 -0.031  0.000
FEEL_ANXIOUS_Y2   0.015  0.002  0.027  0.000

$Japan$mean
    DEPRESSED_Y2      INTEREST_Y2 CONTROL_WORRY_Y2  FEEL_ANXIOUS_Y2
               0                0                0                0

$Japan$th
    DEPRESSED_Y2|t1     DEPRESSED_Y2|t2     DEPRESSED_Y2|t3      INTEREST_Y2|t1      INTEREST
             -0.068               0.004               0.150                   -
0.037              -0.027               0.137               0.135               0.117
0.174
 FEEL_ANXIOUS_Y2|t2  FEEL_ANXIOUS_Y2|t3
             -0.128              -0.019


$Kenya
$Kenya$type
[1] "raw"

$Kenya$cov
                 DEPRES INTERE CONTRO FEEL_A
DEPRESSED_Y2      0.000
INTEREST_Y2      -0.154  0.000
CONTROL_WORRY_Y2 -0.031 -0.004  0.000
FEEL_ANXIOUS_Y2   0.013  0.027 -0.207  0.000

$Kenya$mean
    DEPRESSED_Y2      INTEREST_Y2 CONTROL_WORRY_Y2  FEEL_ANXIOUS_Y2
               0                0                0                0

$Kenya$th
    DEPRESSED_Y2|t1     DEPRESSED_Y2|t2     DEPRESSED_Y2|t3      INTEREST_Y2|t1      INTEREST
              0.070               0.013              -0.219                   -
```

```
0.015               0.116               0.000               -0.080               -
0.050              -0.194               0.207
 FEEL_ANXIOUS_Y2|t2  FEEL_ANXIOUS_Y2|t3
             0.091              -0.026


$Mexico
$Mexico$type
[1] "raw"

$Mexico$cov
                 DEPRES INTERE CONTRO FEEL_A
DEPRESSED_Y2      0.000
INTEREST_Y2       0.008  0.000
CONTROL_WORRY_Y2 -0.049 -0.010  0.000
FEEL_ANXIOUS_Y2   0.028  0.023 -0.060  0.000

$Mexico$mean
    DEPRESSED_Y2      INTEREST_Y2 CONTROL_WORRY_Y2  FEEL_ANXIOUS_Y2
               0                0                0                0

$Mexico$th
    DEPRESSED_Y2|t1     DEPRESSED_Y2|t2     DEPRESSED_Y2|t3      INTEREST_Y2|t1      INTEREST
             -0.003              -0.001               0.028                   -
0.040               0.052              -0.029              -0.059               -
0.100              -0.124               0.149
 FEEL_ANXIOUS_Y2|t2  FEEL_ANXIOUS_Y2|t3
              0.055               0.048


$Nigeria
$Nigeria$type
[1] "raw"

$Nigeria$cov
                 DEPRES INTERE CONTRO FEEL_A
DEPRESSED_Y2      0.000
INTEREST_Y2      -0.217  0.000
CONTROL_WORRY_Y2 -0.018  0.006  0.000
FEEL_ANXIOUS_Y2   0.025 -0.022 -0.264  0.000

$Nigeria$mean
    DEPRESSED_Y2      INTEREST_Y2 CONTROL_WORRY_Y2  FEEL_ANXIOUS_Y2
```

```
               0                 0                 0                 0

$Nigeria$th
    DEPRESSED_Y2|t1     DEPRESSED_Y2|t2     DEPRESSED_Y2|t3      INTEREST_Y2|t1      INTEREST
              0.252               0.002               0.078                   -
0.042              -0.137              -0.238              -0.073                   -
0.131              -0.132               0.284
 FEEL_ANXIOUS_Y2|t2  FEEL_ANXIOUS_Y2|t3
             -0.028               0.066


$Philippines
$Philippines$type
[1] "raw"

$Philippines$cov
                 DEPRES INTERE CONTRO FEEL_A
DEPRESSED_Y2      0.000
INTEREST_Y2      -0.274  0.000
CONTROL_WORRY_Y2 -0.016 -0.015  0.000
FEEL_ANXIOUS_Y2   0.034 -0.022 -0.277  0.000

$Philippines$mean
    DEPRESSED_Y2      INTEREST_Y2 CONTROL_WORRY_Y2  FEEL_ANXIOUS_Y2
               0                0                0                0

$Philippines$th
    DEPRESSED_Y2|t1     DEPRESSED_Y2|t2     DEPRESSED_Y2|t3      INTEREST_Y2|t1      INTEREST
              0.311               0.042               0.000                   -
0.077              -0.236              -0.154              -0.229                   -
0.204              -0.103               0.329
 FEEL_ANXIOUS_Y2|t2  FEEL_ANXIOUS_Y2|t3
              0.185               0.000


$Poland
$Poland$type
[1] "raw"

$Poland$cov
                 DEPRES INTERE CONTRO FEEL_A
DEPRESSED_Y2      0.000
INTEREST_Y2       0.034  0.000
```

```
CONTROL_WORRY_Y2  0.015  0.058  0.000
FEEL_ANXIOUS_Y2  -0.078  0.001  0.029  0.000

$Poland$mean
    DEPRESSED_Y2      INTEREST_Y2 CONTROL_WORRY_Y2  FEEL_ANXIOUS_Y2
               0                0                0                0

$Poland$th
    DEPRESSED_Y2|t1     DEPRESSED_Y2|t2     DEPRESSED_Y2|t3      INTEREST_Y2|t1      INTEREST
              0.000               0.005               0.052                   -
0.088               0.087               0.187               0.072               0.037
0.086
 FEEL_ANXIOUS_Y2|t2  FEEL_ANXIOUS_Y2|t3
             -0.002               0.011


$`South Africa`
$`South Africa`$type
[1] "raw"

$`South Africa`$cov
                 DEPRES INTERE CONTRO FEEL_A
DEPRESSED_Y2      0.000
INTEREST_Y2      -0.190  0.000
CONTROL_WORRY_Y2  0.007  0.012  0.000
FEEL_ANXIOUS_Y2  -0.016  0.002 -0.314  0.000

$`South Africa`$mean
    DEPRESSED_Y2      INTEREST_Y2 CONTROL_WORRY_Y2  FEEL_ANXIOUS_Y2
               0                0                0                0

$`South Africa`$th
    DEPRESSED_Y2|t1     DEPRESSED_Y2|t2     DEPRESSED_Y2|t3      INTEREST_Y2|t1      INTEREST
              0.049              -0.099              -0.139               0.126
0.037              -0.115              -0.182              -0.181               0.296
 FEEL_ANXIOUS_Y2|t2  FEEL_ANXIOUS_Y2|t3
              0.100               0.074


$Spain
$Spain$type
[1] "raw"
```

```
$Spain$cov
                 DEPRES INTERE CONTRO FEEL_A
DEPRESSED_Y2      0.000
INTEREST_Y2       0.002  0.000
CONTROL_WORRY_Y2 -0.014  0.034  0.000
FEEL_ANXIOUS_Y2  -0.015  0.019 -0.030  0.000

$Spain$mean
    DEPRESSED_Y2      INTEREST_Y2 CONTROL_WORRY_Y2  FEEL_ANXIOUS_Y2
               0                0                0                0

$Spain$th
    DEPRESSED_Y2|t1     DEPRESSED_Y2|t2     DEPRESSED_Y2|t3      INTEREST_Y2|t1      INTEREST
             -0.003              -0.017              -0.031                   -
0.059              0.062               0.082               0.131               0.031
0.068
 FEEL_ANXIOUS_Y2|t2  FEEL_ANXIOUS_Y2|t3
             -0.044              -0.114


$Tanzania
$Tanzania$type
[1] "raw"

$Tanzania$cov
                 DEPRES INTERE CONTRO FEEL_A
DEPRESSED_Y2      0.000
INTEREST_Y2      -0.427  0.000
CONTROL_WORRY_Y2  0.020 -0.090  0.000
FEEL_ANXIOUS_Y2   0.072 -0.083 -0.292  0.000

$Tanzania$mean
    DEPRESSED_Y2      INTEREST_Y2 CONTROL_WORRY_Y2  FEEL_ANXIOUS_Y2
               0                0                0                0

$Tanzania$th
    DEPRESSED_Y2|t1     DEPRESSED_Y2|t2     DEPRESSED_Y2|t3      INTEREST_Y2|t1      INTEREST
              0.122              -0.227               0.000               0.263
0.139              0.000               0.034              -0.112               0.000
 FEEL_ANXIOUS_Y2|t2  FEEL_ANXIOUS_Y2|t3
             -0.099               0.000
```

```
$Turkey
$Turkey$type
[1] "raw"

$Turkey$cov
                 DEPRES INTERE CONTRO FEEL_A
DEPRESSED_Y2      0.000
INTEREST_Y2      -0.077  0.000
CONTROL_WORRY_Y2 -0.040 -0.077  0.000
FEEL_ANXIOUS_Y2   0.041 -0.001 -0.116  0.000

$Turkey$mean
    DEPRESSED_Y2      INTEREST_Y2 CONTROL_WORRY_Y2  FEEL_ANXIOUS_Y2
               0                0                0                0

$Turkey$th
    DEPRESSED_Y2|t1     DEPRESSED_Y2|t2     DEPRESSED_Y2|t3      INTEREST_Y2|t1      INTEREST
              0.057              -0.079               0.034               0.154
0.075              -0.101               0.250               0.126               0.148
0.071
 FEEL_ANXIOUS_Y2|t2  FEEL_ANXIOUS_Y2|t3
             -0.238              -0.194


$`United States`
$`United States`$type
[1] "raw"

$`United States`$cov
                 DEPRES INTERE CONTRO FEEL_A
DEPRESSED_Y2      0.000
INTEREST_Y2       0.048  0.000
CONTROL_WORRY_Y2  0.010  0.010  0.000
FEEL_ANXIOUS_Y2  -0.008 -0.014  0.030  0.000

$`United States`$mean
    DEPRESSED_Y2      INTEREST_Y2 CONTROL_WORRY_Y2  FEEL_ANXIOUS_Y2
               0                0                0                0

$`United States`$th
    DEPRESSED_Y2|t1     DEPRESSED_Y2|t2     DEPRESSED_Y2|t3      INTEREST_Y2|t1      INTEREST
             -0.156               0.051               0.132                   -
0.019               0.129               0.176              -0.035               0.062
```

```
0.048
 FEEL_ANXIOUS_Y2|t2  FEEL_ANXIOUS_Y2|t3
              0.032               0.037


$`Hong Kong`
$`Hong Kong`$type
[1] "raw"

$`Hong Kong`$cov
                 DEPRES INTERE CONTRO FEEL_A
DEPRESSED_Y2      0.000
INTEREST_Y2       0.065  0.000
CONTROL_WORRY_Y2  0.027 -0.044  0.000
FEEL_ANXIOUS_Y2  -0.003 -0.071  0.071  0.000

$`Hong Kong`$mean
    DEPRESSED_Y2      INTEREST_Y2 CONTROL_WORRY_Y2  FEEL_ANXIOUS_Y2
               0                0                0                0

$`Hong Kong`$th
    DEPRESSED_Y2|t1     DEPRESSED_Y2|t2     DEPRESSED_Y2|t3      INTEREST_Y2|t1      INTEREST
              0.113               0.061               0.000                 -
0.250                -0.107                0.177                -0.077                0.113
0.246
 FEEL_ANXIOUS_Y2|t2  FEEL_ANXIOUS_Y2|t3
              0.085               0.380
```

```r
## identify which pairs of correlations (items within the same factr) have largest residuals
## same factor item rescor
fit.res.cor0 <- lapply(residuals(fit.strict), \(x) data.frame(DEPRESS = x$cov[2,1], ANX=x$co
fit.res.cor <- fit.res.cor0 |> bind_rows() |> as.data.frame()
rownames(fit.res.cor) <- paste0(names(fit.res.cor0), " (", 1:length(names(fit.res.cor0)), ")"
fit.res.cor #|> mutate(across(everything(), ~round(.,2)))
```

```
                        DEPRESS          ANX
Sweden (1)          -0.053035145  0.033074148
United Kingdom (2)   0.032243256  0.019130252
Germany (3)          0.025862792 -0.068041088
China (4)            0.047893337  0.077313942
Argentina (5)        0.021142736 -0.047364341
Australia (6)        0.046502178  0.030612553
```

Brazil (7)	-0.033266111	-0.062544056
Egypt (8)	-0.186343957	-0.098343127
India (9)	-0.398301298	-0.280329565
Indonesia (10)	-0.078628875	-0.048058698
Israel (11)	0.015475790	-0.008085568
Japan (12)	0.039208463	0.026607969
Kenya (13)	-0.153819977	-0.207360034
Mexico (14)	0.008124919	-0.060065800
Nigeria (15)	-0.217098149	-0.264464503
Philippines (16)	-0.274159408	-0.277210981
Poland (17)	0.034064594	0.029362783
South Africa (18)	-0.190354154	-0.313516251
Spain (19)	0.001593898	-0.030403776
Tanzania (20)	-0.427481044	-0.291590323
Turkey (21)	-0.077143107	-0.115878463
United States (22)	0.047953895	0.029837192
Hong Kong (23)	0.064620088	0.071109710

4.2.8 Fit Comparison

```
# model comparison tests
summary(compareFit(fit.config, fit.threshold, fit.threshold.partial))
```

```
################### Nested Model Comparison #########################

Scaled Chi-Squared Difference Test (method = "satorra.2000")

lavaan->unknown():
   lavaan NOTE: The "Chisq" column contains standard test statistics, not the robust test tha
   statistics.

                       Df AIC BIC   Chisq Chisq diff Df diff Pr(>Chisq)
fit.config             23            54.10
fit.threshold.partial  97           604.88     1346.7      74  < 2.2e-16 ***
fit.threshold         111          2256.70     3719.0      14  < 2.2e-16 ***
---
Signif. codes:  0 '***' 0.001 '**' 0.01 '*' 0.05 '.' 0.1 ' ' 1

####################### Model Fit Indices ###########################
                      chisq.scaled df.scaled pvalue.scaled rmsea.scaled cfi.scaled tli.scalec
fit.config                169.061†        23          .000        .034†     1.000†     0.999†
fit.threshold.partial    1551.415         97          .000        .052      0.998      0.998
fit.threshold            5592.781        111          .000        .094       .994       .993

################## Differences in Fit Indices #######################
                                        df.scaled rmsea.scaled cfi.scaled tli.scaled srmr
fit.threshold.partial - fit.config             74        0.018     -0.001     -
0.001    0
fit.threshold - fit.threshold.partial          14        0.042     -0.004     -
0.005    0
```

```
## compare all remaining models
summary(compareFit(fit.config, fit.threshold.partial, fit.metric, fit.scalar, fit.strict))
```

```
################### Nested Model Comparison #########################

Scaled Chi-Squared Difference Test (method = "satorra.2000")
```

```
lavaan->unknown():
   lavaan NOTE: The "Chisq" column contains standard test statistics, not the robust test tha
   statistics.

                        Df AIC BIC    Chisq Chisq diff Df diff Pr(>Chisq)
fit.config              23             54.10
fit.threshold.partial   97            604.88     1346.7      74  < 2.2e-16 ***
fit.metric             141           1396.76     1300.5      44  < 2.2e-16 ***
fit.scalar             185           4483.00     4088.2      44  < 2.2e-16 ***
fit.strict             273          17240.13    21776.7      88  < 2.2e-16 ***
---
Signif. codes:  0 '***' 0.001 '**' 0.01 '*' 0.05 '.' 0.1 ' ' 1

####################### Model Fit Indices ###########################
                      chisq.scaled df.scaled pvalue.scaled rmsea.scaled cfi.scaled tli.scalec
fit.config                169.061†        23          .000        .034†     1.000†     0.999
fit.threshold.partial    1551.415         97          .000        .052      0.998      0.998
fit.metric               2962.036        141          .000        .060      0.997      0.997
fit.scalar               8014.899        185          .000        .087       .992       .994
fit.strict              29765.275        273          .000        .139       .970       .985

################## Differences in Fit Indices #######################
                                   df.scaled rmsea.scaled cfi.scaled tli.scaled  srmr
fit.threshold.partial - fit.config        74        0.018     -0.001     -
0.001 0.000
fit.metric - fit.threshold.partial        44        0.008     -0.001     -
0.001 0.005
fit.scalar - fit.metric                   44        0.027     -0.005     -
0.003 0.002
fit.strict - fit.scalar                   88        0.052     -0.022     -
0.009 0.037
```

5 Summary and Conclusions

The above tests of invariance and country-specific analyses provide evidence that the 2-factor model for the PHQ-4 is statistically approximately invariant across countries. First, the country-specific results only highlighted potential misfit of the two-factor model in the a potential lack of adequate correlation among items in difference factors–an unaccounted for residual variance or cross-loading. Such an un accounted for residual correlation is not particularly meaningful or impactful for our purposes of analyzing each factor of the PHQ-4 separately. We conclude from these analyses that within each country the separate factor construction is adequate.

Conclusion: The PHQ-4 two-factor model has evidence of at least configural invariance in Wave 2 of the GFS.

Secondly, based on the multi-group CFA analyses (with covariance matrix input and the person-level data input analyses), we conclude that there is partial invariance with respect to the thresholds. Once the third threshold is allowed to be freely estimated for these items and countries,

- DEPRESSED: India (9), Indonesia (10), Philippines (16), Tanzania (20), and Hong Kong (23)
- INTEREST: China (4), Indonesia (10), Kenya (13), Tanzania (20)
- CONTROL_WORRY: Tanzania (20), and Hong Kong (23)
- FEEL_ANXIOUS: India (9), Philippines (16), Tanzania (20),

that there is approximate metric invariance. Meaning that factor loadings are approximately equal across all countries implying that these items load on the depression and anxiety factors approximately the same across all countries. The model fit statistics did not show a meaningful discrepancy between the configural model, partial-threshold invariance model, and the metric invariance model. While the scaled-RMSEA is relatively high (0.06) in the metric invariance model, the other fit indices (scaled-CFI=0.997, scaled-TLI=0.997, SRMR=0.009) are quite good for such a large sample and large number of countries. **The SRMR is especially excellent where the model-implied correlations were, on average, less than 0.01 correlation units off.** While the "largest" discrepancy tended to occurred in the correlation between loss of interest (INTEREST) and worry about control (CONTROL_WORRY), even then the largests was '-0.06' in Egypt.

Conclusion: The PHQ-4 two-factor model has evidence of at least metric invariance in Wave 2 of the GFS.
