# Supplementary material for: Coordinated outcome-wide analytic methodology for multi-wave analyses of the global flourishing study
Source: BMC Glob Public Health. 2026 Jun 2;4:55. doi: 10.1186/s44263-026-00287-6 (PMC13227717; doi:10.1186/s44263-026-00287-6)
Supplement: Supplementary file 3 — Supplementary material 3 Provides a technical overview and the results from the single-item DIF analyses [file 44263_2026_287_MOESM3_ESM.pdf]

Differential Item Functioning for Single-item Measures

Penalized HETOP for measurement invariance

This document provides the results for differential item functioning analyses of the single-item measures used as outcomes in the GFS.

```
knitr::opts_chunk$set(
  warning = FALSE, error = FALSE, message = FALSE,
  cache = TRUE, autodep = TRUE
)
options(width = 200)

library(Rglobalflourishing)
load_packages()
```

```
function (...)
capture_output(.f(...))
<bytecode: 0x1084711f0>
<environment: 0x108470f50>
```

```
options(survey.lonely.psu = "certainty")

## LOAD DATA
df.raw <- gfs_get_labelled_raw_data(
  file = here::here("data", "gfs_all_countries_wave2.sav"),
  list.composites = get_variable_codes('LIST.COMPOSITES')
)

df.raw <- df.raw |>
  mutate(
    across(where(is.factor), ~forcats::fct_na_level_to_value(.,"(Missing)"))
  )
```

1 Single-item Differential Item Functioning

At the core of differential item functioning (DIF) is evaluating whether the distribution function of an observed indicator Y conditional on the unobserved latent variable θ is invariant with respect group membership G. Using the general definition for a distribution function of a random variable, $F(.)$, the fundamental evaluative aim of any DIF analysis is the following:

$$F(Y|\theta) = F(Y|\theta, G)$$

For single-item assessments, we have the unfortunate situation where we have no proxy for θ. All existing methods for DIF evaluation rely on having some proxy for θ, at least to the best of our knowledge. This means that we only have access to

$$F(Y|G) = \int F(Y|\theta, G)F(\theta|G)d\theta$$

With only access to $F(Y|G)$, we have a fundamental identification issues where we need to make some set of assumptions about the distribution of the latent variable within each group, $F(\theta|G)$. Without some assumptions or theory to guide this distribution, any DIF analysis relying solely on $F(Y|G)$ cannot distinguish between bias due to DIF (e.g., the discrepancy between $F(Y|\theta, G)$ and $F(Y|\theta)$) verses any true functional difference in the distribution of the latent variable due to group membership: $F(\theta|G)$.

This indeterminacy is resolved by using some proxy for θ. Either the proxy is based on another variable related to θ or a group of covariates that are predictive of θ. The latter method is typical of propensity score matching approaches where, aside from a focal characteristic, participants are matched on a propensity score created, in theory, to be related to the proxy.

One way to resolve this indeterminacy is to assume no relationship between group membership and the latent variable:

$$F(\theta|G) = F(\theta)$$

then DIF can be identified as differences in the observed distribution conditional on group membership: $F(Y|G)$.

We take a middle ground approach, not fully assuming independence of θ and G, while acknowledging a lack of separation between $F(Y|G)$ and $F(\theta|G)$.

1.1 Heteroskedastic ordered probit model

The heteroskedastic ordered probit (HETOP) model is a discrete outcome model to predict how the probability of endorsing a response category varies as a function of covariates while allowing for heterogeneity in a underlying "latent response distribution" (McCullagh, 1980; Alvarez and Brehm, 1995; Agresti, 2002; Williams, 2009; Reardon et al., 2016; Padgett, 2026). We use the latent response distribution formulation to help emphasize the relationship between the current method and existing factor analytic and item response theory methods of DIF evaluation. Consider an observed variable $Y = 1, 2, \dots, K$, with K response categories for $g = 1, 2, \dots, G$ groups of respondents. For the HETOP model, we assume there is a latent response variable Y^* that is normally distributed within each group g, but not necessarily jointly normally distributed, that is:

$$Y^*|G = g \sim Normal(\mu_g^*, \sigma_g^{*2})$$

where each group, g, has it's own location (μ_g^*) and variance (σ_g^{*2}). The observed variable Y can be "recovered" through the discretization of the latent response variable Y^* using the cut-points or threshold parameters τ_k^* where

$$-\infty = \tau_0^* < \tau_1^* < \tau_2^* < \dots < \tau_{K-1}^* < \tau_K^* = \infty \tau_{k-1}^* < Y^* < \tau_k^* \iff Y = k$$

for $K-1$ thresholds for any K ordered responses. Under the assumption of normality of the latent response distribution within each group, we can model the probability of any response, k, as the difference in cumulative normal distribution around each threshold:

$$Pr(Y = k) = Pr(\tau_{k-1}^* < Y^* < \tau_k^*) = \Phi\left(\frac{\mu_g^* - \tau_{k-1}^*}{\sigma_g^*}\right) - \Phi\left(\frac{\mu_g^* - \tau_k^*}{\sigma_g^*}\right)$$

Estimating this model does not require having person-item level data, meaning summary statistics can be used. This is a major computational advantage because the complexities of estimating the number of participants endorsing each category of a variable can be pre-computed, saving significant time while allowing the estimation of those frequencies to use the full complex sampling design. Given a resulting $G \times K$ matrix of frequencies for each group and category, the parameters $\mathbf{M}^* = [\mu_1^*, \cdots, \mu_G^*]$, $\mathbf{\Sigma}^* = [\sigma_1^*, \cdots, \sigma_G^*]$, and $\mathbf{T}^* = [\tau_1^*, \cdots, \tau_{k-1}^*]$ can be estimated by maximizing the following log-likelihood:

$$\begin{aligned} L &= \sum_{g=1}^{G}\left\{\ln(n_g!) + \sum_{k=1}^{K}\left[n_{gk}\ln(Pr(Y_g = k)) - \ln(n_{gk}!)\right]\right\} \\ &= \sum_{g=1}^{G}\sum_{k=1}^{K} n_{gk}\left\{\Phi\left(\frac{\mu_g^* - \tau_{k-1}^*}{\sigma_g^*}\right) - \Phi\left(\frac{\mu_g^* - \tau_k^*}{\sigma_g^*}\right)\right\} + A \\ A &= \ln\left(\frac{\prod_{g=1}^{G} n_g!}{\prod_{g=1}^{G}\prod_{k=1}^{K} n_{gk}!}\right) \end{aligned}$$

without some constraints, the scale of $\mathbf{M}^*, \mathbf{\Sigma}^*$, and $\mathbf{T}^*$ are indeterminate up to a linear transformation, so some constraints on the magnitude of the parameter estimates are necessary. Reardon and colleagues recommended using:

$$\mathbf{P}(\mathbf{M}^*)^t \equiv \sum_{g=1}^{G} p_g \mu_g^* = 0$$
$$\mathbf{P}(\ln(\mathbf{\Sigma}^*)^t \equiv \sum_{g=1}^{G} p_g \ln(\sigma_g^*) = 0$$

where $\mathbf{P}$ is a vector of group weights. using a unit vector is simplest unless you want to differentially weight the information from each group. These two constraints imply that (a) on average the location parameter is zero across groups, and (b) the residual variance within each group is on average one.

The power of the above HETOP model is two put each group g on the same metric, but each group potentially differs in location and scale on that common metric. This allows for heterogeneity in the distribution of Y across groups to be evaluated not a function of the observed distribution $F(Y|G)$, but as a function of an common metric $F(Y^*|G)$. The common metric is obtained by the common mapping $f : Y \rightarrow Y^*|\mathbf{T}^*$ that is invariant across groups. We have one part of the HETOP model that is assumed invariant (the threshold model), while we have the potentially non-invariant latent response parameters $\mathbf{M}^*, \mathbf{\Sigma}^*$ describing the distribution function of the latent response variable as a function of group membership.

The above parameterization would make the most sense if we were interested in the location and variance. However, in item response theory applications of DIF analysis, we are more often interested in the discrimination parameter of an item characteristic curve. The discrimination parameter is the slope of the item characteristic curve, and is the inverse of the latent response distribution standard deviation $\lambda_g^* = \frac{1}{\sigma_g^*}$. We can therefore rewrite the HETOP model in a form similar to a traditional item response theory model:

$$Pr(Y = k) = \Phi\left[\lambda_g^*(\mu_g^* - \tau_{k-1}^*)\right] - \Phi\left[\lambda_g^*(\mu_g^* - \tau_k^*)\right]$$

the reparameterized HETOP model now has (a) a location parameter μ_g^* for each group that gives each group a higher or lower average response, and (b) a discrimination parameter λ_g^* for each group that determines the relative difference in the strength of the relationship between the latent response variable and the probability of endorsing each category.

Of particular interest for DIF analysis is the relationship between cross-group invariant the latent response variable (defined by the threshold model) and the expected item score (defined by the group item parameters). The expected score can be easily obtained by through the cumulative category probabilties for every level of the latent response variable

$$\begin{aligned} E[Y|Y^* = y^*, G] &= \sum_{k=1}^{K} Pr(Y^* < y^*|G) \\ &= \sum_{k=0}^{K-1} \Phi\left[\lambda_g^*(y^* + \mu_g^* - \tau_k)\right] \end{aligned}$$

The resulting item characteristic function provides a straightforward comparison of the potential DIF.

1.2 Identifying and Penalizing DIF

The above section outlines our single-item DIF model, but we still have the fundamental problem of a lack of complete separation of potential DIF ($F(Y|\theta, G) \neq F(Y|\theta)$) and actual differences among groups on the construct ($F(\theta|G) \neq F(\theta)$). In order to provide a way to test the sensitivity of the differences in the item parameters ($\mathbf{M}^*, \mathbf{\Sigma}^*$), we can add a penalty to the estimation of these model parameters that makes groups the least different collapse to be approximately the same on the latent response variable. Remaining differences that are large after all minor deviations are accounted for may be indicative of DIF.

The updated penalized log-likelihood estimated for a range of penalty values is then:

$$\begin{aligned} L_p &= \sum_{g=1}^{G}\sum_{k=1}^{K} n_{gk}\left\{\Phi\left[\lambda_g^*(\mu_g^* - \tau_{k-1}^*)\right] - \Phi\left[\lambda_g^*(\mu_g^* - \tau_k^*)\right]\right\} + A + P(\mathbf{M}^*) + P(\mathbf{L}^*) \\ P(\mathbf{x}) &= \sum_{\forall g \in G}\sum_{\forall h \neq g \in G} \frac{f(x_g, x_h)}{\nu} \\ f(x_g, x_h) &= \sqrt{|x_g - x_h| + \varepsilon)}, \text{ alignment penalty} \end{aligned}$$

The different penalty methods are discusses in Asparouhov and Muthen (2024) in the context of penalized structural equation models. Changing the magnitude of ν changes the degree of regularization, where the smaller the magnitude, the larger the penalization.

Of particular concern is any non-invariance of the item discrimination parameters. DIF in these parameters implied two potential issues that could impact the interpretation of cross-group comparisons. First, a significantly lower discrimination parameter (below 1.0) implies that the item characteristics curve is flatter relative to other groups. This mean that a the difference between a high versus low response are less indicative of differences on the latent response distribution (i.e., the single-item assessment does not **discriminate** between levels of the latent variable as well). Second, a flattened item characteristic curve implies more variability within group. Any cross-group comparisons will then potentially misinterpret biased due to the greater variability within the group with lower discrimination: lower discrimination of the item means greater variability among people providing the same response that cannot be accounted for.

More information about the proposed penalized heteroskedastic ordered probit model is available in the draft technical report on the proposed method (Padgett, 2026).

Key references

Agresti, A. (2002). Categorical data analysis (2nd ed.). Hoboken, NJ: John Wiley.

Alvarez, R. M., & Brehm, J. (1995). American ambivalence towards abortion policy: Development of a heteroskedastic probit model of competing values. American Journal of Political Science, 39, 1055–1082.

Asparouhov, T. and Muthén, B. (2024). Penalized structural equation models. Structural Equation Modeling: A Multidisciplinary Journal, 31(3):429–454.

McCullagh, P. (1980). Regression models for ordinal data. Journal of the Royal Statistical Society, Series B (Methodological), 42, 109–142.

Padgett, R.N. (2026). A penalized heteroskedastic ordered probit model for DIF (measurement invariance) testing of single-item assessments in cross-cultural research. Preprint available on arXiv. https://arxiv.org/abs/2601.18889

Reardon, S. F., Shear, B. R., Castellano, K. E., and Ho, A. D. (2017). Using heteroskedastic ordered probit models to recover moments of continuous test score distributions from coarsened data. Journal of Educational and Behavioral Statistics, 42(1), 3–45. https://doi.org/10.3102/1076998616666279

Williams, R. (2009). Using heterogeneous choice models to compare logit and probit coefficients across groups. Sociological Methods & Research, 37, 531–559. Retrieved from http://doi.org/10.1177/0049124109335735

The analyses are broken up by construct type, similar to the breakdown of the tables in the online supplement.

2 Psychological Well-Being

2.1 Happiness

Happiness is assessed using the single-item

with categories ranging from '0 -' to '10 -'

```
i <- 1
cur.var <- OUTCOME.LIST[i]
fit <- hetop_pml(
```

```
  data = df.raw |> filter(!is.na(ANNUAL_WEIGHT_C2))
  , var = cur.var
  , group = as.name("COUNTRY")
  , wgt = as.name("ANNUAL_WEIGHT_C2")
  , psu =  as.name("PSU")
  , strata = as.name("STRATA")
  , pen = exp(seq(-5,5,0.5))
  , pen.type="alf"
)

get_plot_latent_mean(fit)
```

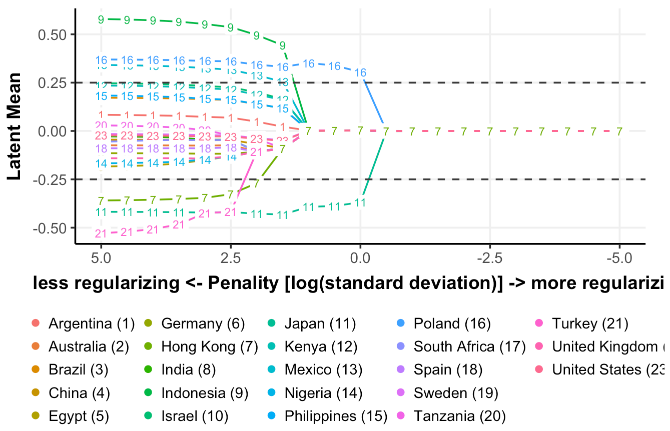


```
get_plot_discrimination(fit)
```

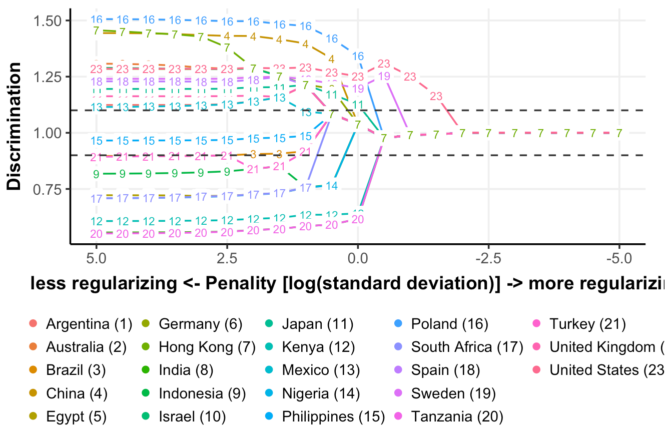


```
plot_iccs(fit, "icc")
```

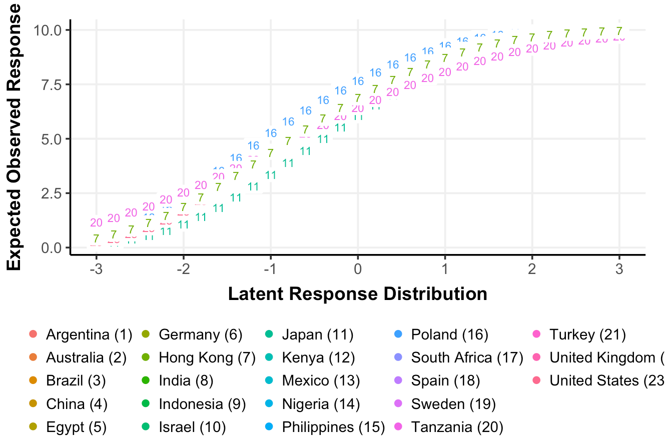


```
format_ft_hetop(fit, 2, cur.var)
```

Happiness (HAPPY_Y2): Threshold Table.				
threshold	est.unstd	se.unstd	est.unstd.lb	est.unstd.ub
c[1]	-2.05	0.05	-2.14	-1.95
c[2]	-1.85	0.04	-1.93	-1.77
c[3]	-1.61	0.04	-1.69	-1.54
c[4]	-1.34	0.03	-1.41	-1.28
c[5]	-1.10	0.03	-1.15	-1.04
c[6]	-0.65	0.03	-0.70	-0.60
c[7]	-0.33	0.02	-0.38	-0.28
c[8]	0.13	0.03	0.07	0.18
c[9]	0.71	0.03	0.65	0.77
c[10]	1.17	0.04	1.10	1.24

```
format_ft_hetop(fit, 1, cur.var)
```

Happiness (HAPPY_Y2): HETOP summary table when penality is nu=0.												
COUNTRY	mu.std	lambda.std	mu.unstd	mu.unstd.se	mu.unstd.lb	mu.unstd.ub	log.lambda.unstd	log.lambda.unstd.se	log.lambda.unstd.lb	log.lambda.unstd.ub	mu.nonzero	log.lambda.nonzero
Poland	0.29	1.32	0.31	0.05	0.21	0.40	0.29	0.05	0.19	0.40	*	*
Indonesia	0.00	1.04	0.00	0.11	-0.21	0.22	0.04	0.08	-0.12	0.19		
Mexico	0.00	1.04	0.00	0.11	-0.21	0.22	0.04	0.09	-0.15	0.22		
Israel	0.00	1.04	0.00	0.10	-0.20	0.20	0.04	0.10	-0.15	0.22		
Brazil	0.00	1.04	0.00	0.07	-0.14	0.15	0.04	0.06	-0.07	0.15		
Kenya	0.00	0.66	0.00	0.09	-0.17	0.18	-0.44	0.05	-0.55	-0.34		*
Philippines	0.00	1.04	0.00	0.09	-0.18	0.18	0.04	0.07	-0.11	0.18		
Argentina	0.00	1.04	0.00	0.09	-0.17	0.17	0.04	0.07	-0.11	0.18		
Tanzania	0.00	0.63	0.00	0.11	-0.21	0.21	-0.48	0.06	-0.60	-0.37		*
South Africa	0.00	1.04	0.00	0.15	-0.29	0.30	0.04	0.10	-0.15	0.23		
India	0.00	0.63	0.00	0.10	-0.20	0.20	-0.48	0.06	-0.59	-0.37		*
China	0.00	1.04	0.00	0.07	-0.14	0.14	0.04	0.07	-0.10	0.18		
Australia	0.00	1.04	0.00	0.09	-0.18	0.18	0.04	0.09	-0.13	0.20		
Spain	0.00	1.04	0.00	0.09	-0.17	0.17	0.04	0.08	-0.11	0.19		
Hong Kong	0.00	1.04	0.00	0.22	-0.43	0.43	0.04	0.20	-0.35	0.43		
Turkey	0.00	1.04	0.00	0.27	-0.53	0.53	0.04	0.17	-0.29	0.37		
Sweden	0.00	1.19	0.00	0.04	-0.08	0.09	0.18	0.04	0.11	0.25		*
United Kingdom	0.00	1.04	0.00	0.08	-0.16	0.16	0.04	0.07	-0.10	0.17		
Germany	0.00	1.04	0.00	0.07	-0.13	0.13	0.04	0.06	-0.08	0.15		
Egypt	0.00	1.03	0.00	0.09	-0.18	0.18	0.04	0.06	-0.08	0.15		
Nigeria	0.00	1.03	0.00	0.09	-0.17	0.18	0.04	0.06	-0.07	0.14		
United States	0.00	1.24	0.00	0.03	-0.06	0.06	0.22	0.03	0.17	0.28		*
Japan	-0.35	1.12	-0.37	0.04	-0.45	-0.29	0.12	0.04	0.05	0.19	*	*

Note. *significant mu/lambda identified from whether the 95% CI for the parameter contains 0.0. Due to rounding, sometimes the estimate may be '-0.00' [slightly below zero] or '0.00' [slightly above zero]. Standard errors estimated using the inverse of the observed information matrix and can sometimes be singular leading to no estimated standard error for some parameters.

2.2 Life satisfaction

```
i <- i + 1
cur.var <- OUTCOME.LIST[i]
fit <- hetop_pml(
  data = df.raw |> filter(!is.na(ANNUAL_WEIGHT_C2))
  , var = cur.var
  , group = as.name("COUNTRY")
  , wgt = as.name("ANNUAL_WEIGHT_C2")
  , psu =  as.name("PSU")
  , strata = as.name("STRATA")
  , pen = exp(seq(-5,5,0.5))
  , pen.type="alf"
)

get_plot_latent_mean(fit)
```

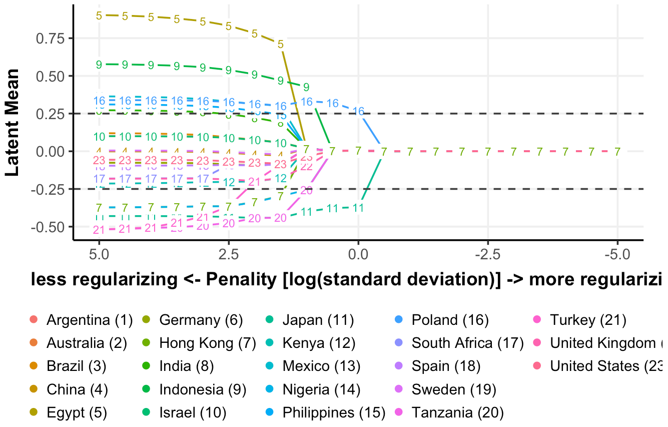


```
get_plot_discrimination(fit)
```

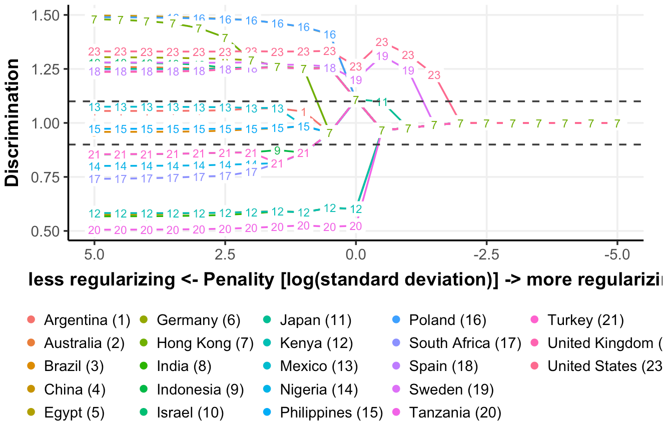


```
plot_iccs(fit, "icc")
```

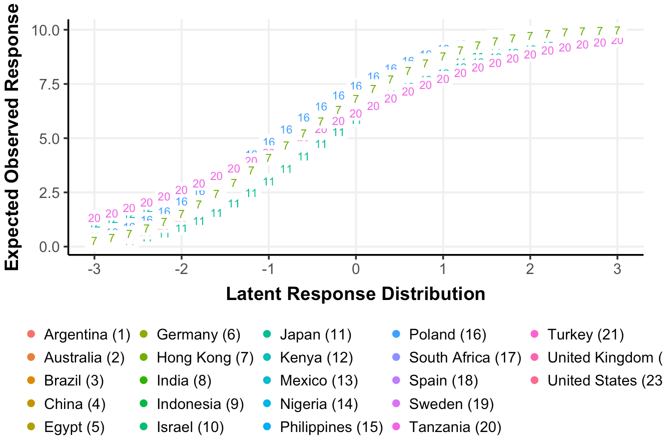

```r
format_ft_hetop(fit, 2, cur.var)
```

Life satisfaction (LIFE_SAT_Y2): Threshold Table.				
**threshold**	**est.unstd**	**se.unstd**	**est.unstd.lb**	**est.unstd.ub**
c[1]	-1.87	0.04	-1.96	-1.78
c[2]	-1.70	0.04	-1.78	-1.62
c[3]	-1.47	0.04	-1.54	-1.40
c[4]	-1.21	0.03	-1.27	-1.15
c[5]	-0.96	0.03	-1.02	-0.91
c[6]	-0.60	0.03	-0.65	-0.54
c[7]	-0.29	0.03	-0.34	-0.24
c[8]	0.14	0.03	0.08	0.19
c[9]	0.67	0.03	0.61	0.73
c[10]	1.13	0.04	1.06	1.20

```r
format_ft_hetop(fit, 1, cur.var)
```

Life satisfaction (LIFE_SAT_Y2): HETOP summary table when penality is nu=0.												
**COUNTRY**	**mu.std**	**lambda.std**	**mu.unstd**	**mu.unstd.se**	**mu.unstd.lb**	**mu.unstd.ub**	**log.lambda.unstd**	**log.lambda.unstd.se**	**log.lambda.unstd.lb**	**log.lambda.unstd.ub**	**mu.nonzero**	**log.lambda.nonzero**
Poland	0.25	1.10	0.27	0.06	0.15	0.38	0.10	0.06	-0.02	0.23	*	
Indonesia	0.01	1.10	0.01	0.11	-0.20	0.21	0.10	0.08	-0.05	0.26		
Mexico	0.00	1.10	0.01	0.11	-0.20	0.21	0.10	0.09	-0.07	0.28		
Philippines	0.00	1.10	0.01	0.09	-0.17	0.18	0.10	0.07	-0.04	0.24		
Egypt	0.00	0.63	0.01	0.19	-0.38	0.39	-0.51	0.12	-0.74	-0.28		*
Sweden	0.00	1.18	0.01	0.04	-0.08	0.09	0.18	0.04	0.11	0.26		*
Brazil	0.00	1.10	0.01	0.07	-0.13	0.14	0.10	0.05	-0.00	0.21		
India	0.00	0.63	0.01	0.10	-0.20	0.21	-0.51	0.06	-0.62	-0.39		*
Argentina	0.00	1.10	0.01	0.08	-0.16	0.17	0.10	0.07	-0.03	0.24		
Israel	0.00	1.10	0.01	0.09	-0.17	0.18	0.10	0.08	-0.06	0.27		
China	0.00	1.10	0.01	0.07	-0.13	0.14	0.10	0.07	-0.03	0.24		
Australia	0.00	1.10	0.00	0.09	-0.17	0.18	0.10	0.08	-0.05	0.26		
South Africa	0.00	1.10	0.00	0.15	-0.28	0.29	0.10	0.10	-0.08	0.29		
Spain	0.00	1.10	0.00	0.08	-0.16	0.17	0.10	0.07	-0.04	0.25		
Turkey	0.00	1.10	0.00	0.25	-0.49	0.50	0.10	0.16	-0.21	0.42		
Hong Kong	0.00	1.10	0.00	0.21	-0.41	0.42	0.10	0.19	-0.27	0.48		
Germany	0.00	1.10	0.00	0.06	-0.12	0.13	0.10	0.06	-0.01	0.21		
Kenya	0.00	0.63	0.00	0.10	-0.19	0.20	-0.51	0.06	-0.62	-0.40		*
Tanzania	0.00	0.55	0.00	0.15	-0.28	0.29	-0.65	0.07	-0.79	-0.50		*
United Kingdom	0.00	1.10	0.00	0.08	-0.15	0.16	0.10	0.07	-0.03	0.23		
Nigeria	0.00	1.10	0.00	0.09	-0.18	0.18	0.10	0.06	-0.02	0.22		
United States	0.00	1.24	0.00	0.03	-0.06	0.07	0.23	0.03	0.18	0.28		*

Note. *significant mu/lambda identified from whether the 95% CI for the parameter contains 0.0. Due to rounding, sometimes the estimate may be '-0.00' [slightly below zero] or '0.00' [slightly above zero]. Standard errors estimated using the inverse of the observed information matrix and can sometimes be singular leading to no estimated standard error for some parameters.

Life satisfaction (LIFE_SAT_Y2): HETOP summary table when penality is nu=0.													
COUNTRY	mu.std	lambda.std	mu.unstd	mu.unstd.se	mu.unstd.lb	mu.unstd.ub	log.lambda.unstd	log.lambda.unstd.se	log.lambda.unstd.lb	log.lambda.unstd.ub	mu.nonzero	log.lambda.nonzero
Japan	-0.34	1.10	-0.37	0.04	-0.46	-0.29	0.10	0.04	0.03	0.18	*	*

Note. *significant mu/lambda identified from whether the 95% CI for the parameter contains 0.0. Due to rounding, sometimes the estimate may be '-0.00' [slightly below zero] or '0.00' [slightly above zero]. Standard errors estimated using the inverse of the observed information matrix and can sometimes be singular leading to no estimated standard error for some parameters.

2.3 Current life evaluation

```r
i <- i + 1
cur.var <- OUTCOME.LIST[i]
fit <- hetop_pml(
  data = df.raw |> filter(!is.na(ANNUAL_WEIGHT_C2))
  , var = cur.var
  , group = as.name("COUNTRY")
  , wgt = as.name("ANNUAL_WEIGHT_C2")
  , psu =  as.name("PSU")
  , strata = as.name("STRATA")
  , pen = exp(seq(-5,5,0.5))
  , pen.type="alf"
)

get_plot_latent_mean(fit)
```

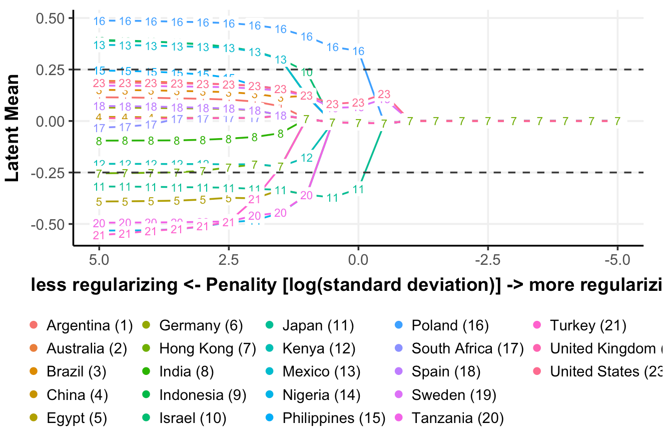


```r
get_plot_discrimination(fit)
```

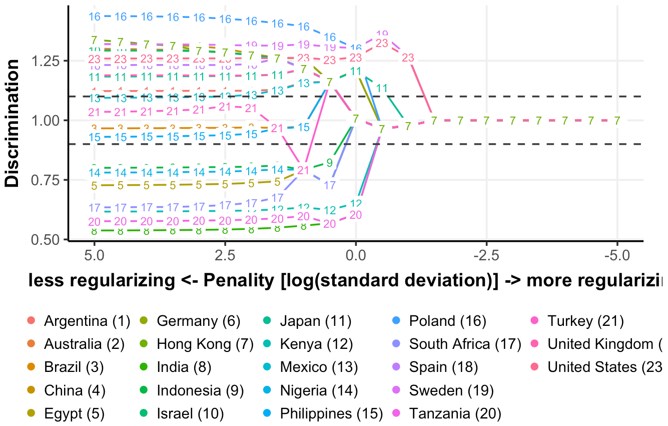


```r
plot_iccs(fit, "icc")
```

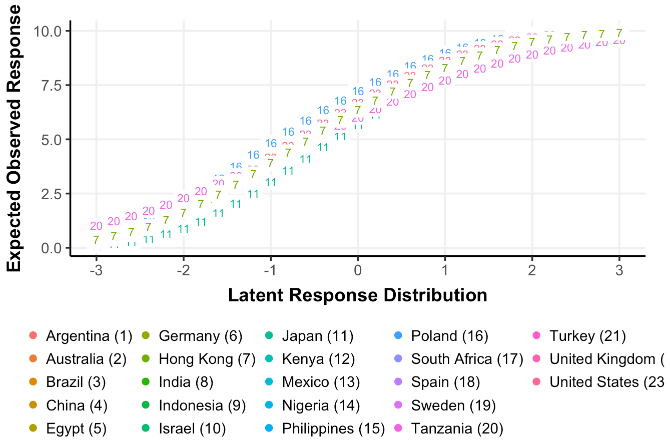


```r
format_ft_hetop(fit, 2, cur.var)
```

Current life evaluation (WB_TODAY_Y2): Threshold Table.

threshold	est.unstd	se.unstd	est.unstd.lb	est.unstd.ub
c[1]	-2.03	0.05	-2.13	-1.93
c[2]	-1.75	0.04	-1.83	-1.67
c[3]	-1.47	0.04	-1.54	-1.40
c[4]	-1.17	0.03	-1.23	-1.11
c[5]	-0.92	0.03	-0.97	-0.86
c[6]	-0.45	0.03	-0.50	-0.40
c[7]	-0.10	0.03	-0.15	-0.05
c[8]	0.39	0.03	0.33	0.44
c[9]	0.99	0.04	0.91	1.06
c[10]	1.39	0.04	1.31	1.48

```r
format_ft_hetop(fit, 1, cur.var)
```

Current life evaluation (WB_TODAY_Y2): HETOP summary table when penality is nu=0.

COUNTRY	mu.std	lambda.std	mu.unstd	mu.unstd.se	mu.unstd.lb	mu.unstd.ub	log.lambda.unstd	log.lambda.unstd.se	log.lambda.unstd.lb	log.lambda.unstd.ub	mu.nonzero	log.lambda.nonzero
Poland	0.32	1.28	0.34	0.05	0.24	0.44	0.26	0.05	0.16	0.37	*	*
United States	0.09	1.25	0.09	0.03	0.03	0.16	0.23	0.03	0.18	0.29	*	*
Sweden	0.06	1.28	0.07	0.04	-0.01	0.15	0.26	0.04	0.19	0.34		*
Israel	-0.01	1.01	-0.01	0.11	-0.23	0.21	0.01	0.10	-0.19	0.21		
Indonesia	-0.01	1.01	-0.01	0.10	-0.20	0.18	0.01	0.07	-0.13	0.14		
Mexico	-0.01	1.01	-0.01	0.11	-0.23	0.21	0.01	0.09	-0.17	0.19		
Philippines	-0.01	1.01	-0.01	0.09	-0.19	0.18	0.01	0.07	-0.13	0.15		
Brazil	-0.01	1.01	-0.01	0.08	-0.16	0.14	0.01	0.06	-0.11	0.12		
Australia	-0.01	1.01	-0.01	0.10	-0.20	0.18	0.01	0.09	-0.17	0.18		
Argentina	-0.01	1.01	-0.01	0.09	-0.18	0.17	0.01	0.08	-0.14	0.16		
Germany	-0.01	1.20	-0.01	0.06	-0.12	0.10	0.19	0.05	0.09	0.29		*
Spain	-0.01	1.01	-0.01	0.09	-0.18	0.17	0.01	0.08	-0.15	0.17		

Note. *significant mu/lambda identified from whether the 95% CI for the parameter contains 0.0. Due to rounding, sometimes the estimate may be '-0.00' [slightly below zero] or '0.00' [slightly above zero]. Standard errors estimated using the inverse of the observed information matrix and can sometimes be singular leading to no estimated standard error for some parameters.

Current life evaluation (WB_TODAY_Y2): HETOP summary table when penality is nu=0.

COUNTRY	mu.std	lambda.std	mu.unstd	mu.unstd.se	mu.unstd.lb	mu.unstd.ub	log.lambda.unstd	log.lambda.unstd.se	log.lambda.unstd.lb	log.lambda.unstd.ub	mu.nonzero	log.lambda.nonzero
South Africa	-0.01	1.01	-0.01	0.15	-0.31	0.29	0.01	0.09	-0.17	0.19		
United Kingdom	-0.01	1.01	-0.01	0.08	-0.17	0.15	0.01	0.07	-0.13	0.15		
Hong Kong	-0.01	1.01	-0.01	0.21	-0.41	0.40	0.01	0.19	-0.36	0.37		
China	-0.01	1.20	-0.01	0.06	-0.13	0.11	0.19	0.06	0.07	0.31		*
Turkey	-0.01	1.01	-0.01	0.33	-0.66	0.64	0.01	0.23	-0.44	0.45		
India	-0.01	0.62	-0.01	0.10	-0.21	0.20	-0.50	0.06	-0.61	-0.39		*
Kenya	-0.01	0.67	-0.01	0.09	-0.19	0.17	-0.43	0.05	-0.53	-0.33		*
Tanzania	-0.01	0.62	-0.01	0.13	-0.26	0.24	-0.50	0.07	-0.63	-0.37		*
Egypt	-0.01	1.01	-0.01	0.10	-0.21	0.19	0.01	0.06	-0.12	0.13		
Nigeria	-0.01	1.01	-0.01	0.12	-0.24	0.22	0.01	0.07	-0.13	0.14		
Japan	-0.31	1.20	-0.33	0.04	-0.41	-0.25	0.19	0.03	0.12	0.26	*	*

Note. *significant mu/lambda identified from whether the 95% CI for the parameter contains 0.0. Due to rounding, sometimes the estimate may be '-0.00' [slightly below zero] or '0.00' [slightly above zero]. Standard errors estimated using the inverse of the observed information matrix and can sometimes be singular leading to no estimated standard error for some parameters.

2.4 Future life evaluation

```
i <- i + 1
cur.var <- OUTCOME.LIST[i]
fit <- hetop_pml(
  data = df.raw |> filter(!is.na(ANNUAL_WEIGHT_C2))
  , var = cur.var
  , group = as.name("COUNTRY")
  , wgt = as.name("ANNUAL_WEIGHT_C2")
  , psu =  as.name("PSU")
  , strata = as.name("STRATA")
  , pen = exp(seq(-5,5,0.5))
  , pen.type="alf"
)

get_plot_latent_mean(fit)
```

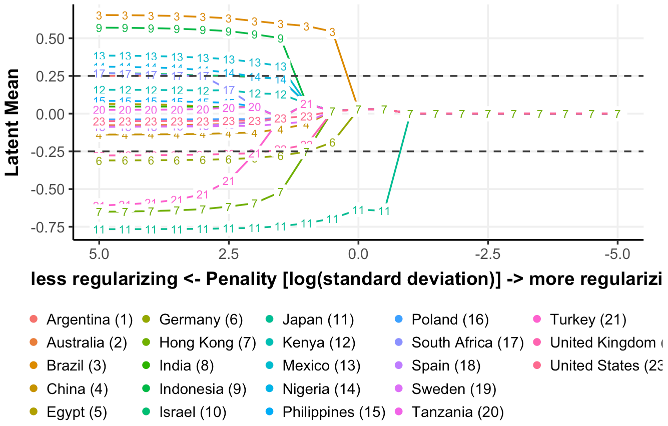


```
get_plot_discrimination(fit)
```

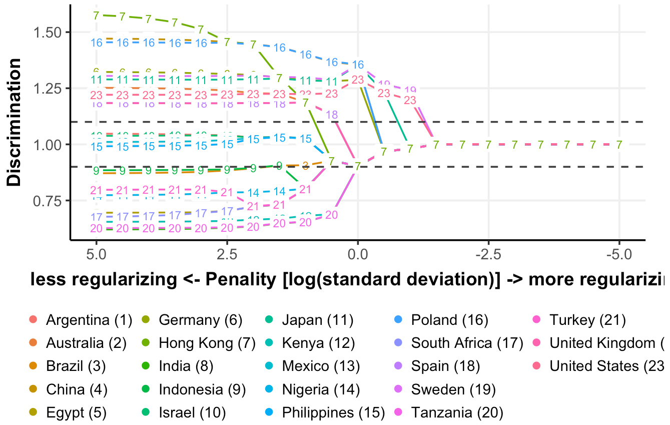


```
plot_iccs(fit, "icc")
```

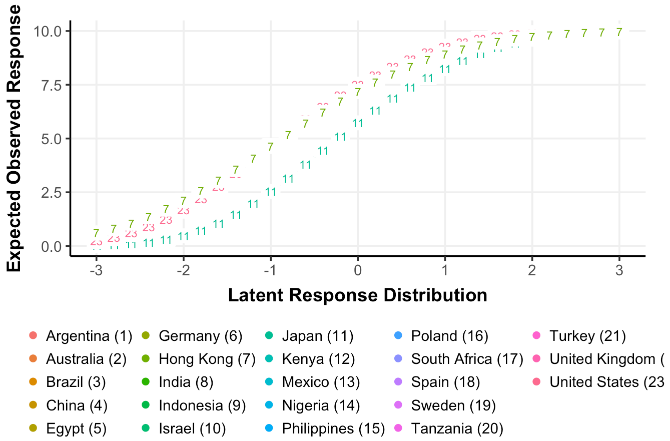


```
format_ft_hetop(fit, 2, cur.var)
```

Future life evaluation (WB_FIVEYRS_Y2): Threshold Table.

threshold	est.unstd	se.unstd	est.unstd.lb	est.unstd.ub
c[1]	-2.03	0.06	-2.14	-1.92
c[2]	-1.79	0.05	-1.88	-1.70
c[3]	-1.56	0.04	-1.64	-1.48
c[4]	-1.33	0.04	-1.40	-1.25
c[5]	-1.13	0.03	-1.20	-1.06
c[6]	-0.77	0.03	-0.83	-0.70
c[7]	-0.51	0.04	-0.58	-0.44
c[8]	-0.13	0.04	-0.21	-0.05
c[9]	0.39	0.05	0.29	0.50
c[10]	0.89	0.06	0.76	1.02

```
format_ft_hetop(fit, 1, cur.var)
```

Future life evaluation (WB_FIVEYRS_Y2): HETOP summary table when penality is nu=0.

COUNTRY	mu.std	lambda.std	mu.unstd	mu.unstd.se	mu.unstd.lb	mu.unstd.ub	log.lambda.unstd	log.lambda.unstd.se	log.lambda.unstd.lb	log.lambda.unstd.ub	mu.nonzero	log.lambda.nonzero
Brazil	0.03	0.91	0.03	0.12	-0.21	0.27	-0.10	0.11	-0.32	0.12		
Indonesia	0.03	0.91	0.03	0.14	-0.24	0.30	-0.10	0.12	-0.34	0.14		
Mexico	0.03	0.91	0.03	0.13	-0.23	0.29	-0.10	0.12	-0.34	0.14		
Nigeria	0.03	0.91	0.03	0.10	-0.18	0.23	-0.10	0.08	-0.26	0.06		
Argentina	0.03	0.91	0.03	0.11	-0.19	0.25	-0.10	0.10	-0.30	0.10		
Israel	0.03	0.91	0.03	0.12	-0.21	0.27	-0.10	0.11	-0.32	0.12		
Kenya	0.03	0.91	0.03	0.07	-0.12	0.17	-0.10	0.05	-0.20	-0.00		*
Philippines	0.03	0.91	0.03	0.11	-0.19	0.24	-0.10	0.09	-0.29	0.09		
South Africa	0.03	0.91	0.03	0.18	-0.32	0.38	-0.10	0.13	-0.35	0.15		
Egypt	0.03	0.91	0.03	0.11	-0.20	0.25	-0.10	0.08	-0.26	0.05		
Poland	0.03	1.34	0.03	0.06	-0.09	0.15	0.30	0.06	0.19	0.42		*
Australia	0.03	0.91	0.03	0.11	-0.19	0.25	-0.10	0.11	-0.31	0.11		
Spain	0.03	0.91	0.03	0.11	-0.18	0.24	-0.10	0.10	-0.29	0.09		
India	0.03	0.91	0.03	0.08	-0.13	0.19	-0.10	0.05	-0.21	0.00		
Tanzania	0.03	0.91	0.03	0.09	-0.14	0.20	-0.10	0.06	-0.21	0.01		
Turkey	0.03	0.91	0.03	0.34	-0.64	0.70	-0.10	0.21	-0.52	0.31		
Hong Kong	0.03	0.91	0.03	0.71	-1.37	1.42	-0.10	0.61	-1.30	1.10		
United Kingdom	0.03	0.91	0.03	0.11	-0.18	0.24	-0.10	0.09	-0.28	0.08		
China	0.03	1.34	0.03	0.07	-0.11	0.17	0.30	0.07	0.17	0.44		*
United States	0.03	1.28	0.03	0.05	-0.07	0.12	0.25	0.04	0.18	0.32		*
Sweden	0.03	1.34	0.03	0.06	-0.08	0.14	0.30	0.05	0.21	0.39		*
Germany	0.03	1.28	0.03	0.08	-0.12	0.18	0.25	0.06	0.13	0.38		*
Japan	-0.61	1.34	-0.64	0.04	-0.72	-0.55	0.30	0.04	0.22	0.39	*	*

Note. *significant mu/lambda identified from whether the 95% CI for the parameter contains 0.0. Due to rounding, sometimes the estimate may be '-0.00' [slightly below zero] or '0.00' [slightly above zero]. Standard errors estimated using the inverse of the observed information matrix and can sometimes be singular leading to no estimated standard error for some parameters.

2.5 Optimism

```
i <- i + 1
cur.var <- OUTCOME.LIST[i]
fit <- hetop_pml(
  data = df.raw |> filter(!is.na(ANNUAL_WEIGHT_C2))
  , var = cur.var
  , group = as.name("COUNTRY")
  , wgt = as.name("ANNUAL_WEIGHT_C2")
  , psu =  as.name("PSU")
  , strata = as.name("STRATA")
  , pen = exp(seq(-5,5,0.5))
  , pen.type="alf"
)

get_plot_latent_mean(fit)
```

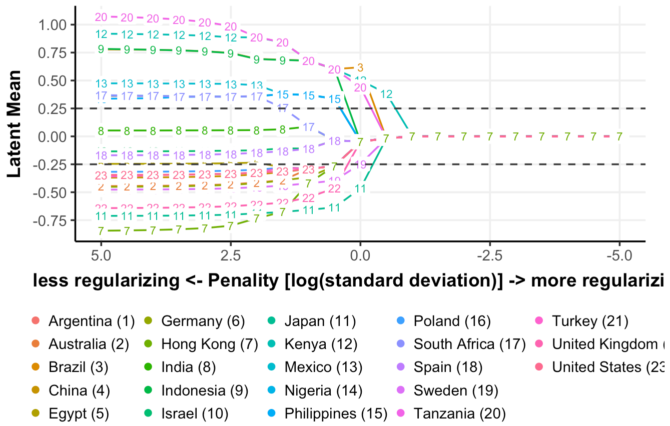


```
get_plot_discrimination(fit)
```

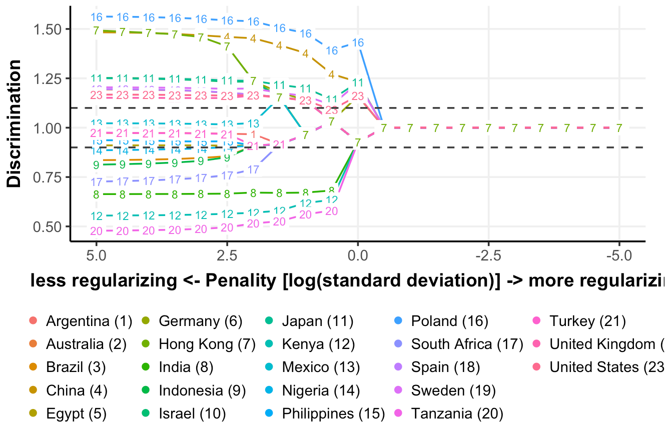


```
plot_iccs(fit, "icc")
```

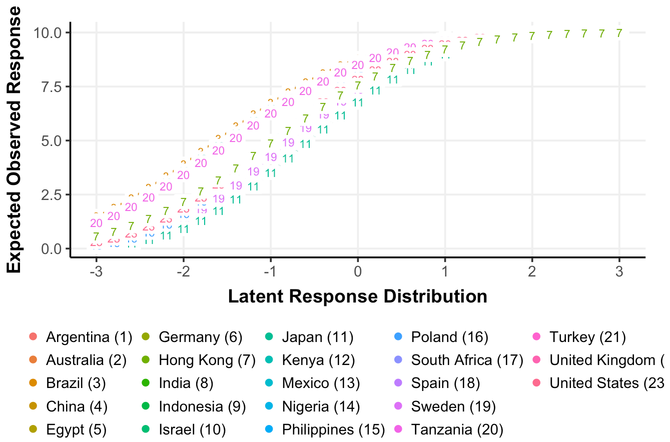


```
format_ft_hetop(fit, 2, cur.var)
```

Optimism (EXPECT_GOOD_Y2): Threshold Table.

threshold	est.unstd	se.unstd	est.unstd.lb	est.unstd.ub
c[1]	-1.99	0.05	-2.10	-1.89
c[2]	-1.86	0.05	-1.96	-1.76
c[3]	-1.69	0.04	-1.77	-1.61

Optimism (EXPECT_GOOD_Y2): Threshold Table.				
threshold	est.unstd	se.unstd	est.unstd.lb	est.unstd.ub
c[4]	-1.49	0.04	-1.57	-1.42
c[5]	-1.30	0.03	-1.37	-1.24
c[6]	-0.93	0.02	-0.97	-0.88
c[7]	-0.68	0.02	-0.72	-0.65
c[8]	-0.36	0.02	-0.40	-0.33
c[9]	0.07	0.02	0.04	0.10
c[10]	0.43	0.02	0.38	0.47

```
format_ft_hetop(fit, 1, cur.var)
```

Optimism (EXPECT_GOOD_Y2): HETOP summary table when penality is nu=0.													
COUNTRY	mu.std	lambda.std	mu.unstd	mu.unstd.se	mu.unstd.lb	mu.unstd.ub	log.lambda.unstd	log.lambda.unstd.se	log.lambda.unstd.lb	log.lambda.unstd.ub	mu.nonzero	log.lambda.nonzero
Brazil	0.59	0.93	0.62	0.09	0.43	0.80	-0.07	0.10	-0.26	0.11	*	
Kenya	0.48	0.93	0.50	0.07	0.36	0.64	-0.08	0.06	-0.19	0.04	*	
Tanzania	0.42	0.93	0.44	0.08	0.28	0.60	-0.08	0.06	-0.20	0.05	*	
Indonesia	-0.04	0.93	-0.05				-0.08					
Argentina	-0.04	0.93	-0.05	0.13	-0.31	0.22	-0.08	0.15	-0.38	0.23		
Nigeria	-0.04	0.93	-0.05	0.11	-0.27	0.18	-0.08	0.12	-0.31	0.16		
Mexico	-0.04	0.93	-0.05	0.27	-0.58	0.49	-0.08	0.35	-0.77	0.62		
Philippines	-0.04	0.93	-0.05	0.13	-0.29	0.20	-0.08	0.14	-0.35	0.20		
India	-0.04	0.93	-0.05	0.07	-0.18	0.09	-0.08	0.06	-0.19	0.04		
Israel	-0.05	0.93	-0.05	0.11	-0.25	0.16	-0.07	0.12	-0.31	0.16		
South Africa	-0.05	0.93	-0.05	0.18	-0.40	0.31	-0.08	0.16	-0.39	0.24		
Spain	-0.05	0.93	-0.05	0.10	-0.24	0.14	-0.07	0.11	-0.28	0.13		
Egypt	-0.05	0.93	-0.05	0.10	-0.24	0.15	-0.08	0.09	-0.25	0.10		
Turkey	-0.05	0.93	-0.05	0.25	-0.55	0.45	-0.08	0.21	-0.49	0.34		
Australia	-0.05	0.93	-0.05	0.12	-0.27	0.18	-0.07	0.11	-0.29	0.14		
Hong Kong	-0.05	0.93	-0.05	0.47	-0.96	0.87	-0.08	0.36	-0.78	0.63		
China	-0.05	1.22	-0.05	0.06	-0.17	0.08	0.21	0.08	0.06	0.36		*
Poland	-0.05	1.41	-0.05	0.05	-0.14	0.04	0.36	0.06	0.24	0.48		*
United Kingdom	-0.05	0.93	-0.05	0.12	-0.28	0.19	-0.07	0.10	-0.27	0.12		
Germany	-0.05	1.15	-0.05	0.07	-0.18	0.08	0.15	0.07	0.02	0.28		*
United States	-0.05	1.16	-0.05	0.03	-0.11	0.01	0.15	0.04	0.08	0.22		*
Sweden	-0.24	1.22	-0.25	0.04	-0.32	-0.17	0.20	0.05	0.12	0.29	*	*
Japan	-0.45	1.22	-0.47	0.04	-0.53	-0.40	0.21	0.04	0.12	0.29	*	*

Note. *significant mu/lambda identified from whether the 95% CI for the parameter contains 0.0. Due to rounding, sometimes the estimate may be '-0.00' [slightly below zero] or '0.00' [slightly above zero]. Standard errors estimated using the inverse of the observed information matrix and can sometimes be singular leading to no estimated standard error for some parameters.

2.6 Freedom to pursue what's important

```
i <- i + 1
cur.var <- OUTCOME.LIST[i]
fit <- hetop_pml(
  data = df.raw |> filter(!is.na(ANNUAL_WEIGHT_C2))
```

```
  , var = cur.var
  , group = as.name("COUNTRY")
  , wgt = as.name("ANNUAL_WEIGHT_C2")
  , psu =  as.name("PSU")
  , strata = as.name("STRATA")
  , pen = exp(seq(-5,5,0.5))
  , pen.type="alf"
)

get_plot_latent_mean(fit)
```

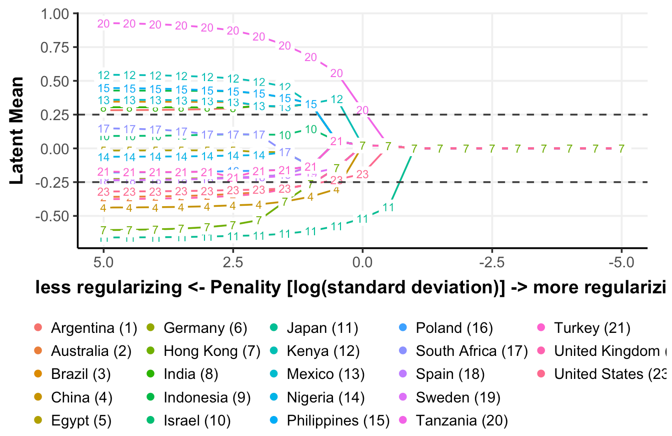


```
get_plot_discrimination(fit)
```

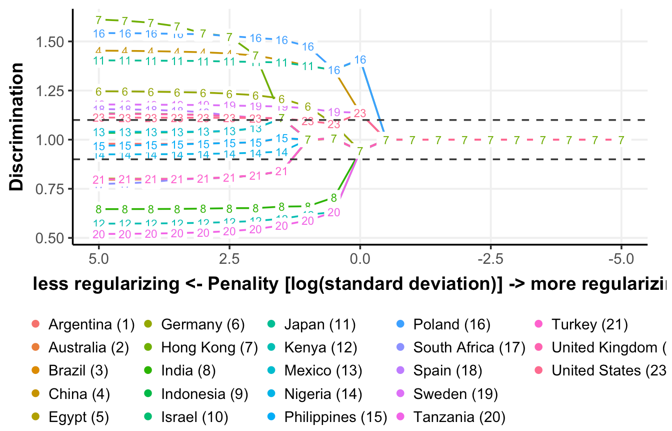


```
plot_iccs(fit, "icc")
```

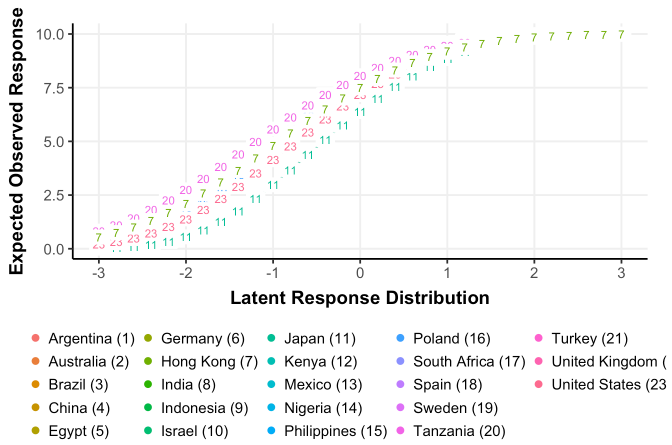


```
format_ft_hetop(fit, 2, cur.var)
```

Freedom to pursue what's important (FREEDOM_Y2): Threshold Table.				
**threshold**	**est.unstd**	**se.unstd**	**est.unstd.lb**	**est.unstd.ub**
c[1]	-1.93	0.05	-2.03	-1.83
c[2]	-1.79	0.05	-1.88	-1.70
c[3]	-1.60	0.04	-1.68	-1.52
c[4]	-1.39	0.04	-1.46	-1.32
c[5]	-1.18	0.03	-1.25	-1.12
c[6]	-0.85	0.03	-0.91	-0.79
c[7]	-0.59	0.03	-0.65	-0.53
c[8]	-0.25	0.03	-0.31	-0.19
c[9]	0.19	0.03	0.12	0.25
c[10]	0.56	0.04	0.48	0.63

```
format_ft_hetop(fit, 1, cur.var)
```

Freedom to pursue what's important (FREEDOM_Y2): HETOP summary table when penality is nu=0.												
**COUNTRY**	**mu.std**	**lambda.std**	**mu.unstd**	**mu.unstd.se**	**mu.unstd.lb**	**mu.unstd.ub**	**log.lambda.unstd**	**log.lambda.unstd.se**	**log.lambda.unstd.lb**	**log.lambda.unstd.ub**	**mu.nonzero**	**log.lambda.nonzero**
Tanzania	0.28	0.94	0.29	0.08	0.13	0.44	-0.06	0.06	-0.17	0.05	*	
Kenya	0.02	0.94	0.02	0.07	-0.11	0.15	-0.06	0.05	-0.16	0.04		
Brazil	0.02	0.94	0.02	0.09	-0.15	0.19	-0.06	0.08	-0.21	0.09		
Indonesia	0.02	0.94	0.02	0.13	-0.24	0.28	-0.06	0.14	-0.34	0.22		
Philippines	0.02	0.94	0.02	0.13	-0.22	0.27	-0.06	0.13	-0.31	0.19		
India	0.02	0.94	0.02	0.07	-0.12	0.16	-0.06	0.05	-0.17	0.05		
Argentina	0.02	0.94	0.02	0.11	-0.18	0.23	-0.06	0.10	-0.26	0.14		
Mexico	0.02	0.94	0.02	0.13	-0.24	0.28	-0.06	0.14	-0.33	0.21		
Israel	0.02	0.94	0.02	0.11	-0.19	0.23	-0.06	0.11	-0.28	0.16		
South Africa	0.02	0.94	0.02	0.17	-0.31	0.35	-0.06	0.14	-0.33	0.21		
Nigeria	0.02	0.94	0.02	0.10	-0.17	0.21	-0.06	0.08	-0.22	0.10		
Egypt	0.02	0.94	0.02	0.10	-0.18	0.22	-0.06	0.08	-0.22	0.10		
Turkey	0.02	0.94	0.02	0.25	-0.47	0.51	-0.06	0.19	-0.42	0.31		
Spain	0.02	0.94	0.02	0.11	-0.19	0.23	-0.06	0.10	-0.25	0.13		
Hong Kong	0.02	0.94	0.02	0.44	-0.84	0.88	-0.06	0.40	-0.85	0.73		
Germany	0.02	0.94	0.02	0.08	-0.13	0.18	-0.06	0.07	-0.20	0.09		
Australia	0.02	0.94	0.02	0.12	-0.21	0.26	-0.06	0.10	-0.26	0.14		
United Kingdom	0.02	0.94	0.02	0.11	-0.19	0.23	-0.06	0.09	-0.23	0.12		
Poland	0.02	1.40	0.02	0.05	-0.09	0.13	0.34	0.06	0.23	0.46		*
China	0.02	1.13	0.02	0.09	-0.16	0.20	0.13	0.09	-0.04	0.30		
Sweden	0.02	1.13	0.02	0.05	-0.09	0.12	0.13	0.05	0.04	0.22		*
United States	-0.19	1.13	-0.19	0.04	-0.26	-0.12	0.13	0.03	0.06	0.19	*	*
Japan	-0.51	1.40	-0.52	0.04	-0.60	-0.44	0.34	0.04	0.26	0.42	*	*

Freedom to pursue what's important (FREEDOM_Y2): HETOP summary table when penality is nu=0.

COUNTRY	mu.std	lambda.std	mu.unstd	mu.unstd.se	mu.unstd.lb	mu.unstd.ub	log.lambda.unstd	log.lambda.unstd.se	log.lambda.unstd.lb	log.lambda.unstd.ub	mu.nonzero	log.lambda.nonzero

Note. *significant mu/lambda identified from whether the 95% CI for the parameter contains 0.0. Due to rounding, sometimes the estimate may be '-0.00' [slightly below zero] or '0.00' [slightly above zero]. Standard errors estimated using the inverse of the observed information matrix and can sometimes be singular leading to no estimated standard error for some parameters.

2.7 Inner peace

```
i <- i + 1
cur.var <- OUTCOME.LIST[i]
fit <- hetop_pml(
  data = df.raw |> filter(!is.na(ANNUAL_WEIGHT_C2))
  , var = cur.var
  , group = as.name("COUNTRY")
  , wgt = as.name("ANNUAL_WEIGHT_C2")
  , psu =  as.name("PSU")
  , strata = as.name("STRATA")
  , pen = exp(seq(-5,5,0.5))
  , pen.type="alf"
)

get_plot_latent_mean(fit)
```

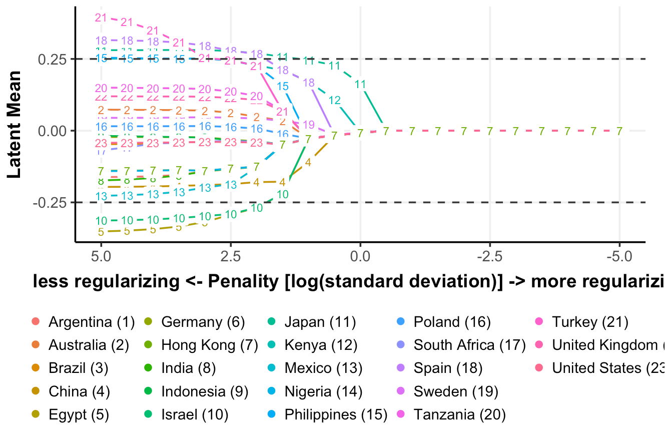


```
get_plot_discrimination(fit)
```

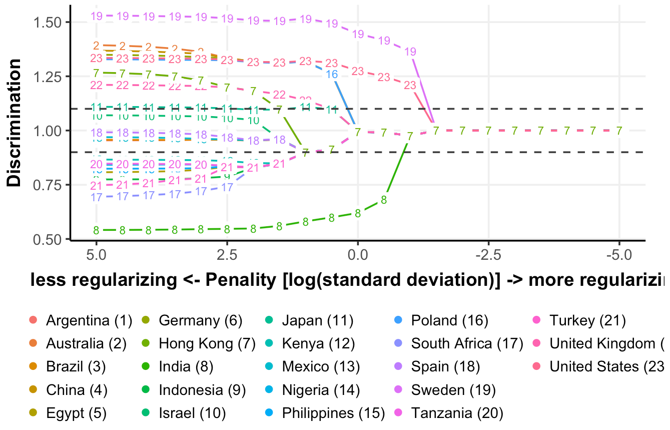


```
plot_iccs(fit, "icc")
```

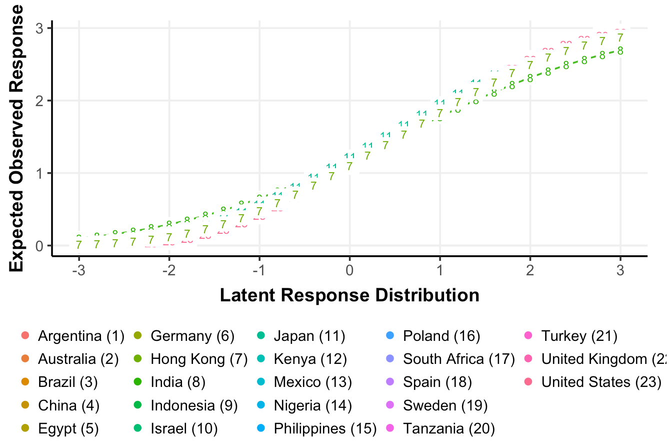


```
format_ft_hetop(fit, 2, cur.var)
```

Inner peace (PEACE_Y2): Threshold Table.

threshold	est.unstd	se.unstd	est.unstd.lb	est.unstd.ub
c[1]	-0.80	0.03	-0.86	-0.74
c[2]	0.58	0.03	0.53	0.64
c[3]	1.79	0.04	1.71	1.88

```
format_ft_hetop(fit, 1, cur.var)
```

Inner peace (PEACE_Y2): HETOP summary table when penality is nu=0.

COUNTRY	mu.std	lambda.std	mu.unstd	mu.unstd.se	mu.unstd.lb	mu.unstd.ub	log.lambda.unstd	log.lambda.unstd.se	log.lambda.unstd.lb	log.lambda.unstd.ub	mu.nonzero	log.lambda.nonzero
Japan	0.16	0.99	0.16	0.05	0.07	0.26	-0.01	0.04	-0.09	0.08	*	
Kenya	-0.01	0.99	-0.01	0.07	-0.14	0.13	-0.01	0.05	-0.11	0.09		
Sweden	-0.01	1.44	-0.01	0.04	-0.09	0.08	0.37	0.04	0.28	0.45		*
Tanzania	-0.01	0.99	-0.01	0.07	-0.15	0.14	-0.01	0.06	-0.12	0.10		
Spain	-0.01	0.99	-0.01	0.11	-0.22	0.21	-0.01	0.08	-0.17	0.16		
Philippines	-0.01	0.99	-0.01	0.11	-0.22	0.21	-0.01	0.08	-0.16	0.15		
United Kingdom	-0.01	0.99	-0.01	0.09	-0.18	0.17	-0.01	0.08	-0.16	0.15		
Turkey	-0.01	0.99	-0.01	0.27	-0.54	0.52	-0.01	0.18	-0.35	0.34		
Australia	-0.01	0.99	-0.01	0.10	-0.21	0.19	-0.01	0.10	-0.20	0.19		
Indonesia	-0.01	0.99	-0.01	0.10	-0.21	0.19	-0.01	0.07	-0.15	0.14		
South Africa	-0.01	0.99	-0.01	0.16	-0.33	0.31	-0.01	0.12	-0.23	0.22		
Poland	-0.01	0.99	-0.01	0.07	-0.14	0.12	-0.01	0.06	-0.13	0.12		
Hong Kong	-0.01	0.99	-0.01	0.19	-0.39	0.37	-0.01	0.19	-0.38	0.36		
Brazil	-0.01	0.99	-0.01	0.08	-0.17	0.15	-0.01	0.07	-0.14	0.12		
Germany	-0.01	0.99	-0.01	0.07	-0.15	0.13	-0.01	0.07	-0.14	0.13		
India	-0.01	0.63	-0.01	0.10	-0.21	0.20	-0.48	0.06	-0.60	-0.36		*
Nigeria	-0.01	0.99	-0.01	0.09	-0.19	0.17	-0.01	0.07	-0.15	0.14		
Argentina	-0.01	0.99	-0.01	0.10	-0.20	0.18	-0.01	0.08	-0.17	0.15		
Mexico	-0.01	0.99	-0.01	0.11	-0.23	0.21	-0.01	0.09	-0.19	0.18		

Note. *significant mu/lambda identified from whether the 95% CI for the parameter contains 0.0. Due to rounding, sometimes the estimate may be '-0.00' [slightly below zero] or '0.00' [slightly above zero]. Standard errors estimated using the inverse of the observed information matrix and can sometimes be singular leading to no estimated standard error for some parameters.

Inner peace (PEACE_Y2): HETOP summary table when penality is nu=0.												
COUNTRY	mu.std	lambda.std	mu.unstd	mu.unstd.se	mu.unstd.lb	mu.unstd.ub	log.lambda.unstd	log.lambda.unstd.se	log.lambda.unstd.lb	log.lambda.unstd.ub	mu.nonzero	log.lambda.nonzero
Israel	-0.01	0.99	-0.01	0.11	-0.23	0.21	-0.01	0.10	-0.21	0.19		
Egypt	-0.01	0.99	-0.01	0.10	-0.20	0.19	-0.01	0.08	-0.16	0.15		
China	-0.01	0.99	-0.01	0.08	-0.17	0.15	-0.01	0.08	-0.17	0.15		
United States	-0.01	1.27	-0.01	0.03	-0.07	0.06	0.24	0.03	0.18	0.30		*

Note. *significant mu/lambda identified from whether the 95% CI for the parameter contains 0.0. Due to rounding, sometimes the estimate may be '-0.00' [slightly below zero] or '0.00' [slightly above zero]. Standard errors estimated using the inverse of the observed information matrix and can sometimes be singular leading to no estimated standard error for some parameters.

2.8 Life balance

```
i <- i + 1
cur.var <- OUTCOME.LIST[i]
fit <- hetop_pml(
  data = df.raw |> filter(!is.na(ANNUAL_WEIGHT_C2))
  , var = cur.var
  , group = as.name("COUNTRY")
  , wgt = as.name("ANNUAL_WEIGHT_C2")
  , psu =  as.name("PSU")
  , strata = as.name("STRATA")
  , pen = exp(seq(-5,5,0.5))
  , pen.type="alf"
)

get_plot_latent_mean(fit)
```

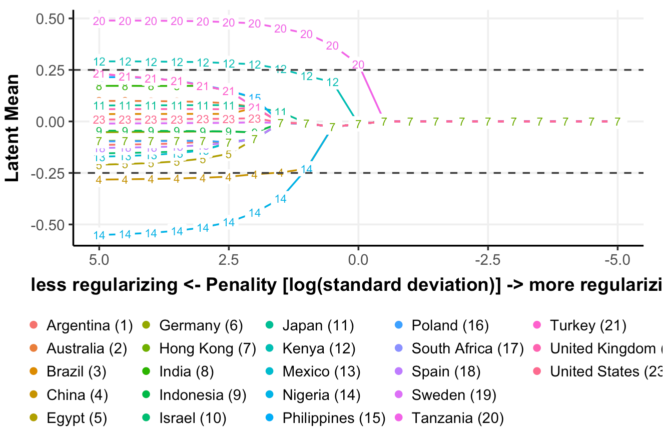


```
get_plot_discrimination(fit)
```

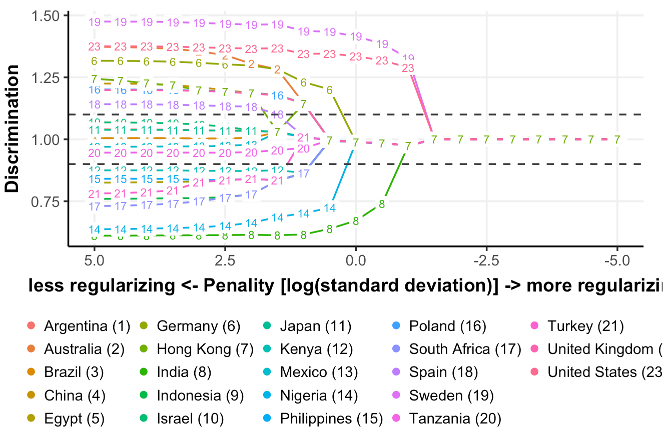


```
plot_iccs(fit, "icc")
```

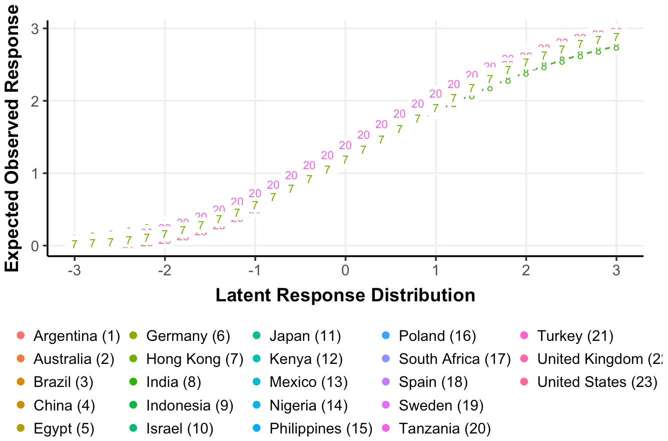


```
format_ft_hetop(fit, 2, cur.var)
```

Life balance (LIFE_BALANCE_Y2): Threshold Table.				
**threshold**	**est.unstd**	**se.unstd**	**est.unstd.lb**	**est.unstd.ub**
c[1]	-0.98	0.03	-1.04	-0.92
c[2]	0.48	0.03	0.42	0.53
c[3]	1.72	0.04	1.64	1.80

```
format_ft_hetop(fit, 1, cur.var)
```

Life balance (LIFE_BALANCE_Y2): HETOP summary table when penality is nu=0.

COUNTRY	mu.std	lambda.std	mu.unstd	mu.unstd.se	mu.unstd.lb	mu.unstd.ub	log.lambda.unstd	log.lambda.unstd.se	log.lambda.unstd.lb	log.lambda.unstd.ub	mu.nonzero	log.lambda.nonzero
Tanzania	0.27	0.99	0.28	0.07	0.13	0.42	-0.01	0.06	-0.12	0.10	*	
Kenya	-0.01	0.99	-0.01	0.07	-0.14	0.12	-0.01	0.05	-0.11	0.09		
Japan	-0.01	0.99	-0.01	0.05	-0.11	0.08	-0.01	0.04	-0.09	0.07		
Philippines	-0.01	0.99	-0.01	0.11	-0.22	0.20	-0.01	0.08	-0.16	0.14		
India	-0.01	0.68	-0.01	0.10	-0.21	0.18	-0.40	0.06	-0.52	-0.28		*
South Africa	-0.01	0.99	-0.01	0.17	-0.35	0.33	-0.01	0.12	-0.24	0.22		
Australia	-0.01	0.99	-0.01	0.10	-0.22	0.19	-0.01	0.10	-0.20	0.18		
United States	-0.01	1.33	-0.01	0.03	-0.08	0.05	0.29	0.03	0.23	0.35		*
United Kingdom	-0.01	0.99	-0.01	0.09	-0.18	0.16	-0.01	0.08	-0.16	0.14		
Turkey	-0.01	0.99	-0.01	0.25	-0.49	0.47	-0.01	0.17	-0.35	0.32		
Brazil	-0.01	0.99	-0.01	0.08	-0.17	0.15	-0.01	0.07	-0.14	0.12		
Indonesia	-0.01	0.99	-0.01	0.10	-0.21	0.18	-0.01	0.07	-0.15	0.13		
Hong Kong	-0.01	0.99	-0.01	0.19	-0.39	0.36	-0.01	0.18	-0.36	0.34		
Argentina	-0.01	0.99	-0.01	0.10	-0.20	0.18	-0.01	0.08	-0.17	0.15		
Germany	-0.01	0.99	-0.01	0.07	-0.15	0.13	-0.01	0.07	-0.14	0.12		
Mexico	-0.01	0.99	-0.01	0.11	-0.23	0.20	-0.01	0.09	-0.19	0.16		
Israel	-0.01	0.99	-0.01	0.11	-0.22	0.20	-0.01	0.09	-0.19	0.17		
Spain	-0.01	0.99	-0.01	0.10	-0.20	0.18	-0.01	0.09	-0.18	0.16		

Note. *significant mu/lambda identified from whether the 95% CI for the parameter contains 0.0. Due to rounding, sometimes the estimate may be '-0.00' [slightly below zero] or '0.00' [slightly above zero]. Standard errors estimated using the inverse of the observed information matrix and can sometimes be singular leading to no estimated standard error for some parameters.

Life balance (LIFE_BALANCE_Y2): HETOP summary table when penality is nu=0.												
COUNTRY	mu.std	lambda.std	mu.unstd	mu.unstd.se	mu.unstd.lb	mu.unstd.ub	log.lambda.unstd	log.lambda.unstd.se	log.lambda.unstd.lb	log.lambda.unstd.ub	mu.nonzero	log.lambda.nonzero
Egypt	-0.01	0.99	-0.01	0.10	-0.20	0.18	-0.01	0.07	-0.15	0.13		
Sweden	-0.01	1.41	-0.01	0.04	-0.10	0.07	0.35	0.04	0.26	0.43		*
Poland	-0.01	0.99	-0.01	0.07	-0.15	0.12	-0.01	0.06	-0.13	0.11		
Nigeria	-0.01	0.99	-0.01	0.10	-0.21	0.18	-0.01	0.07	-0.15	0.12		
China	-0.01	0.99	-0.01	0.08	-0.18	0.15	-0.01	0.08	-0.16	0.14		

Note. *significant mu/lambda identified from whether the 95% CI for the parameter contains 0.0. Due to rounding, sometimes the estimate may be '-0.00' [slightly below zero] or '0.00' [slightly above zero]. Standard errors estimated using the inverse of the observed information matrix and can sometimes be singular leading to no estimated standard error for some parameters.

2.9 Sense of mastery

```
i <- i + 1
cur.var <- OUTCOME.LIST[i]
fit <- hetop_pml(
  data = df.raw |> filter(!is.na(ANNUAL_WEIGHT_C2))
  , var = cur.var
  , group = as.name("COUNTRY")
  , wgt = as.name("ANNUAL_WEIGHT_C2")
  , psu =  as.name("PSU")
  , strata = as.name("STRATA")
  , pen = exp(seq(-5,5,0.5))
  , pen.type="alf"
)

get_plot_latent_mean(fit)
```

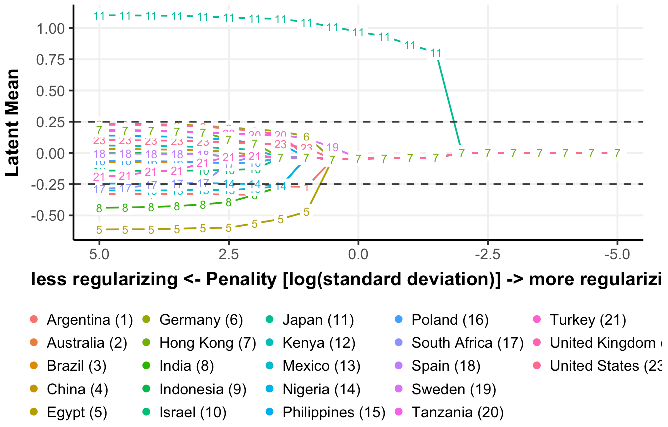


```
get_plot_discrimination(fit)
```

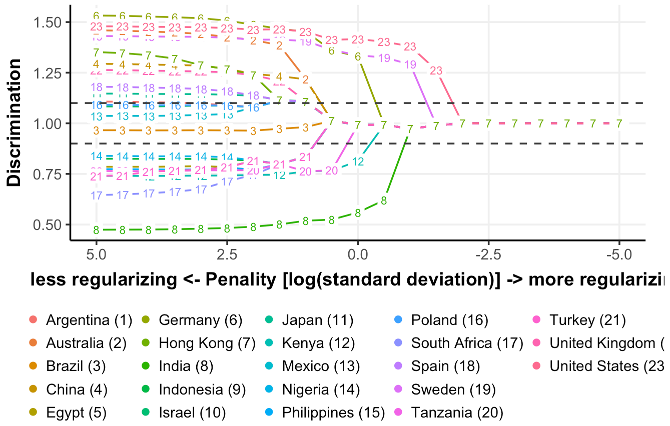


```
plot_iccs(fit, "icc")
```

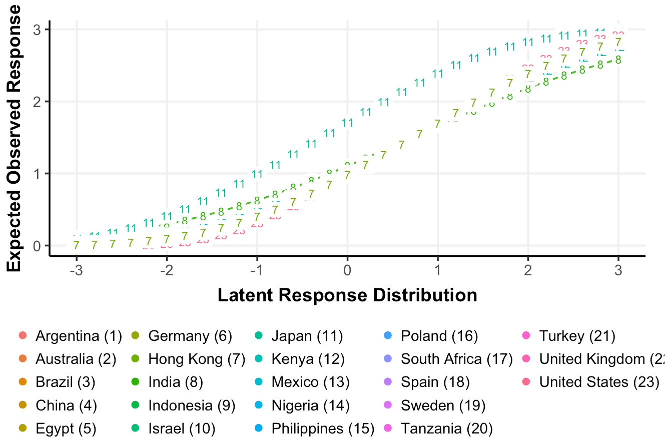


```
format_ft_hetop(fit, 2, cur.var)
```

Sense of mastery (CAPABLE_Y2): Threshold Table.

threshold	est.unstd	se.unstd	est.unstd.lb	est.unstd.ub
c[1]	-0.69	0.03	-0.74	-0.63
c[2]	0.73	0.03	0.68	0.79
c[3]	1.95	0.05	1.86	2.05

```
format_ft_hetop(fit, 1, cur.var)
```

Sense of mastery (CAPABLE_Y2): HETOP summary table when penality is nu=0.

COUNTRY	mu.std	lambda.std	mu.unstd	mu.unstd.se	mu.unstd.lb	mu.unstd.ub	log.lambda.unstd	log.lambda.unstd.se	log.lambda.unstd.lb	log.lambda.unstd.ub	mu.nonzero	log.lambda.nonzero
Japan	0.92	0.99	0.97	0.05	0.87	1.07	-0.01	0.04	-0.09	0.08	*	
Sweden	-0.04	1.32	-0.04	0.04	-0.13	0.04	0.29	0.04	0.20	0.38		*
United States	-0.04	1.39	-0.04	0.03	-0.11	0.02	0.35	0.03	0.29	0.41		*
Germany	-0.04	1.31	-0.04	0.06	-0.16	0.08	0.29	0.06	0.16	0.41		*
Tanzania	-0.04	0.99	-0.04	0.08	-0.19	0.11	-0.01	0.06	-0.12	0.10		
United Kingdom	-0.04	0.99	-0.04	0.09	-0.22	0.14	-0.01	0.08	-0.17	0.16		
Australia	-0.04	0.99	-0.04	0.11	-0.25	0.17	-0.01	0.11	-0.22	0.20		
Philippines	-0.04	0.99	-0.04	0.10	-0.25	0.16	-0.01	0.08	-0.16	0.14		
Kenya	-0.04	0.82	-0.04	0.08	-0.19	0.10	-0.21	0.05	-0.31	-0.10		*
Hong Kong	-0.04	0.99	-0.04	0.20	-0.43	0.34	-0.01	0.19	-0.39	0.37		
Indonesia	-0.04	0.99	-0.04	0.10	-0.24	0.16	-0.01	0.08	-0.16	0.15		
Turkey	-0.04	0.99	-0.04	0.23	-0.49	0.41	-0.01	0.18	-0.35	0.34		
South Africa	-0.04	0.99	-0.04	0.17	-0.37	0.28	-0.01	0.12	-0.24	0.23		
Spain	-0.04	0.99	-0.04	0.10	-0.23	0.15	-0.01	0.09	-0.19	0.17		
China	-0.04	0.99	-0.04	0.08	-0.20	0.11	-0.01	0.08	-0.16	0.14		
Israel	-0.04	0.99	-0.04	0.11	-0.25	0.17	-0.01	0.10	-0.20	0.19		
Brazil	-0.04	0.99	-0.04	0.08	-0.20	0.12	-0.01	0.07	-0.15	0.13		
India	-0.04	0.58	-0.04	0.12	-0.27	0.18	-0.58	0.07	-0.71	-0.45		*

Note. *significant mu/lambda identified from whether the 95% CI for the parameter contains 0.0. Due to rounding, sometimes the estimate may be '-0.00' [slightly below zero] or '0.00' [slightly above zero]. Standard errors estimated using the inverse of the observed information matrix and can sometimes be singular leading to no estimated standard error for some parameters.

Sense of mastery (CAPABLE_Y2): HETOP summary table when penality is nu=0.												
COUNTRY	mu.std	lambda.std	mu.unstd	mu.unstd.se	mu.unstd.lb	mu.unstd.ub	log.lambda.unstd	log.lambda.unstd.se	log.lambda.unstd.lb	log.lambda.unstd.ub	mu.nonzero	log.lambda.nonzero
Nigeria	-0.04	0.99	-0.04	0.10	-0.23	0.14	-0.01	0.08	-0.16	0.15		
Mexico	-0.04	0.99	-0.04	0.12	-0.28	0.19	-0.01	0.11	-0.23	0.21		
Poland	-0.04	0.99	-0.04	0.07	-0.18	0.09	-0.01	0.06	-0.13	0.11		
Argentina	-0.04	0.99	-0.04	0.11	-0.26	0.17	-0.01	0.11	-0.22	0.20		
Egypt	-0.04	0.99	-0.04	0.12	-0.27	0.18	-0.01	0.10	-0.20	0.19		

Note. *significant mu/lambda identified from whether the 95% CI for the parameter contains 0.0. Due to rounding, sometimes the estimate may be '-0.00' [slightly below zero] or '0.00' [slightly above zero]. Standard errors estimated using the inverse of the observed information matrix and can sometimes be singular leading to no estimated standard error for some parameters.

2.10 Meaningful activities

```
i <- i + 1
cur.var <- OUTCOME.LIST[i]
fit <- hetop_pml(
  data = df.raw |> filter(!is.na(ANNUAL_WEIGHT_C2))
  , var = cur.var
  , group = as.name("COUNTRY")
  , wgt = as.name("ANNUAL_WEIGHT_C2")
  , psu =  as.name("PSU")
  , strata = as.name("STRATA")
  , pen = exp(seq(-5,5,0.5))
  , pen.type="alf"
)

get_plot_latent_mean(fit)
```

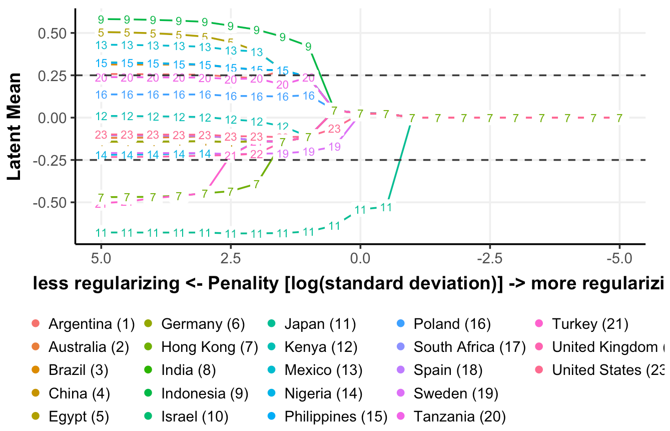


```
get_plot_discrimination(fit)
```

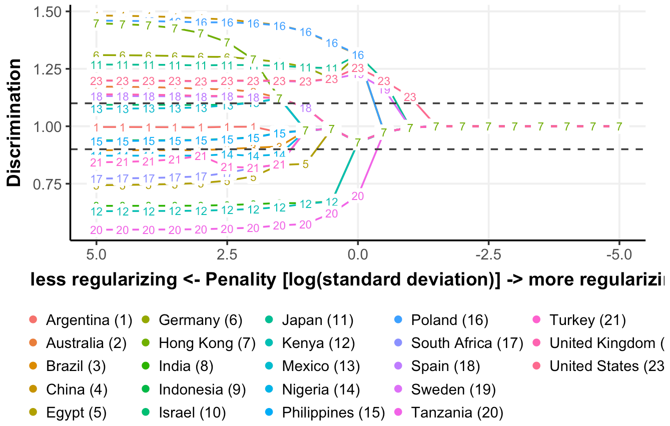


```
plot_iccs(fit, "icc")
```

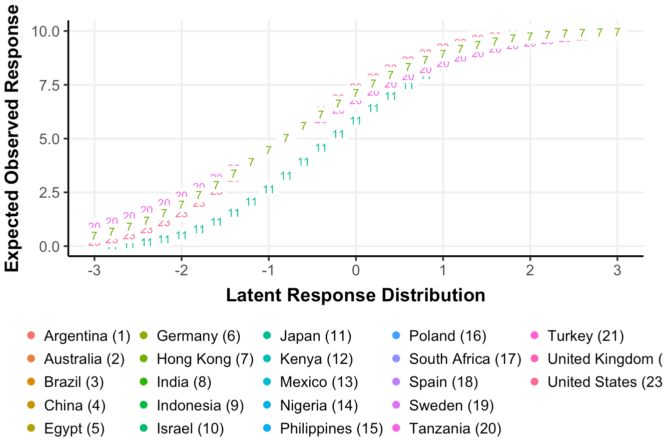


```
format_ft_hetop(fit, 2, cur.var)
```

Meaningful activities (WORTHWHILE_Y2): Threshold Table.

threshold	est.unstd	se.unstd	est.unstd.lb	est.unstd.ub
c[1]	-1.90	0.05	-1.99	-1.80
c[2]	-1.73	0.04	-1.81	-1.64
c[3]	-1.52	0.04	-1.60	-1.44
c[4]	-1.30	0.04	-1.37	-1.23
c[5]	-1.08	0.03	-1.15	-1.02
c[6]	-0.72	0.03	-0.78	-0.66
c[7]	-0.43	0.03	-0.49	-0.38
c[8]	-0.06	0.03	-0.12	-0.00
c[9]	0.42	0.03	0.35	0.48
c[10]	0.81	0.04	0.74	0.89

```
format_ft_hetop(fit, 1, cur.var)
```

Meaningful activities (WORTHWHILE_Y2): HETOP summary table when penality is nu=0.

COUNTRY	mu.std	lambda.std	mu.unstd	mu.unstd.se	mu.unstd.lb	mu.unstd.ub	log.lambda.unstd	log.lambda.unstd.se	log.lambda.unstd.lb	log.lambda.unstd.ub	mu.nonzero	log.lambda.nonzero
Poland	0.02	1.30	0.03	0.05	-0.08	0.13	0.27	0.06	0.15	0.39		*
Indonesia	0.02	0.93	0.03	0.15	-0.27	0.32	-0.07	0.14	-0.34	0.19		
Brazil	0.02	0.93	0.03	0.09	-0.15	0.20	-0.07	0.08	-0.22	0.08		
Egypt	0.02	0.93	0.03	0.11	-0.18	0.23	-0.07	0.09	-0.24	0.10		
Mexico	0.02	0.93	0.03	0.15	-0.26	0.31	-0.07	0.14	-0.34	0.20		
Israel	0.02	0.93	0.03	0.12	-0.22	0.27	-0.07	0.12	-0.30	0.16		
Philippines	0.02	0.93	0.03	0.11	-0.19	0.24	-0.07	0.10	-0.27	0.12		
Argentina	0.02	0.93	0.02	0.10	-0.18	0.23	-0.07	0.10	-0.26	0.12		
Tanzania	0.02	0.71	0.02	0.10	-0.17	0.22	-0.36	0.06	-0.48	-0.24		*
South Africa	0.02	0.93	0.02	0.17	-0.31	0.36	-0.07	0.13	-0.32	0.18		
Spain	0.02	0.93	0.02	0.10	-0.17	0.22	-0.07	0.09	-0.25	0.11		
Australia	0.02	0.93	0.02	0.10	-0.18	0.23	-0.07	0.10	-0.26	0.12		

Note. *significant mu/lambda identified from whether the 95% CI for the parameter contains 0.0. Due to rounding, sometimes the estimate may be '-0.00' [slightly below zero] or '0.00' [slightly above zero]. Standard errors estimated using the inverse of the observed information matrix and can sometimes be singular leading to no estimated standard error for some parameters.

Meaningful activities (WORTHWHILE_Y2): HETOP summary table when penality is nu=0.													
COUNTRY	mu.std	lambda.std	mu.unstd	mu.unstd.se	mu.unstd.lb	mu.unstd.ub	log.lambda.unstd	log.lambda.unstd.se	log.lambda.unstd.lb	log.lambda.unstd.ub	mu.nonzero	log.lambda.nonzero
Turkey	0.02	0.93	0.02	0.30	-0.56	0.61	-0.07	0.20	-0.47	0.32		
Kenya	0.02	0.93	0.02	0.07	-0.11	0.16	-0.07	0.05	-0.16	0.02		
Hong Kong	0.02	0.93	0.02	0.28	-0.52	0.57	-0.07	0.26	-0.58	0.44		
Nigeria	0.02	0.93	0.02	0.10	-0.17	0.22	-0.07	0.08	-0.22	0.08		
United Kingdom	0.02	0.93	0.02	0.09	-0.16	0.21	-0.07	0.08	-0.23	0.09		
India	0.02	0.93	0.02	0.07	-0.12	0.17	-0.07	0.05	-0.17	0.02		
China	0.02	1.30	0.02	0.06	-0.10	0.14	0.27	0.07	0.14	0.40		*
Germany	0.02	1.30	0.02	0.06	-0.09	0.14	0.27	0.06	0.16	0.38		*
United States	0.02	1.25	0.02	0.04	-0.05	0.09	0.23	0.03	0.17	0.29		*
Sweden	0.02	1.22	0.02	0.05	-0.07	0.11	0.21	0.04	0.12	0.29		*
Japan	-0.53	1.30	-0.54	0.04	-0.62	-0.46	0.27	0.04	0.20	0.34	*	*

Note. *significant mu/lambda identified from whether the 95% CI for the parameter contains 0.0. Due to rounding, sometimes the estimate may be '-0.00' [slightly below zero] or '0.00' [slightly above zero]. Standard errors estimated using the inverse of the observed information matrix and can sometimes be singular leading to no estimated standard error for some parameters.

2.11 Understanding purpose

```r
i <- i + 1
cur.var <- OUTCOME.LIST[i]
fit <- hetop_pml(
  data = df.raw |> filter(!is.na(ANNUAL_WEIGHT_C2))
  , var = cur.var
  , group = as.name("COUNTRY")
  , wgt = as.name("ANNUAL_WEIGHT_C2")
  , psu =  as.name("PSU")
  , strata = as.name("STRATA")
  , pen = exp(seq(-5,5,0.5))
  , pen.type="alf"
)

get_plot_latent_mean(fit)
```

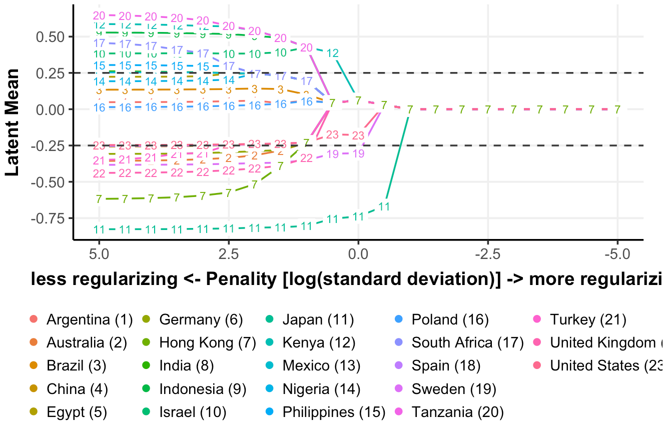


```r
get_plot_discrimination(fit)
```

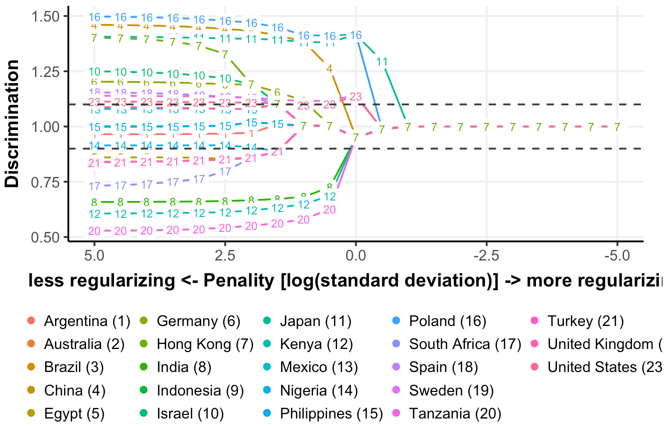


```
plot_iccs(fit, "icc")
```

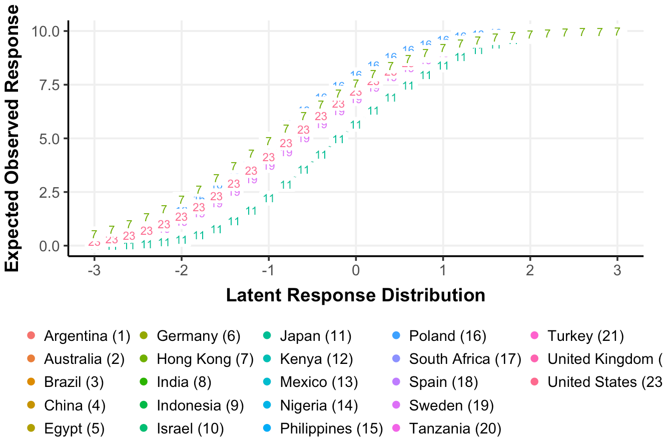


```
format_ft_hetop(fit, 2, cur.var)
```

Understanding purpose (LIFE_PURPOSE_Y2): Threshold Table.

threshold	est.unstd	se.unstd	est.unstd.lb	est.unstd.ub
c[1]	-1.89	0.05	-1.98	-1.80
c[2]	-1.76	0.04	-1.84	-1.67
c[3]	-1.59	0.04	-1.67	-1.51
c[4]	-1.39	0.04	-1.46	-1.31
c[5]	-1.20	0.03	-1.26	-1.13
c[6]	-0.84	0.03	-0.90	-0.78
c[7]	-0.58	0.03	-0.64	-0.52
c[8]	-0.24	0.03	-0.31	-0.17
c[9]	0.20	0.04	0.13	0.28
c[10]	0.56	0.05	0.47	0.66

```
format_ft_hetop(fit, 1, cur.var)
```

Understanding purpose (LIFE_PURPOSE_Y2): HETOP summary table when penality is nu=0.												
**COUNTRY**	**mu.std**	**lambda.std**	**mu.unstd**	**mu.unstd.se**	**mu.unstd.lb**	**mu.unstd.ub**	**log.lambda.unstd**	**log.lambda.unstd.se**	**log.lambda.unstd.lb**	**log.lambda.unstd.ub**	**mu.nonzero**	**log.lambda.nonzero**
Kenya	0.06	0.95	0.06	0.07	-0.07	0.20	-0.05	0.05	-0.16	0.05		
Indonesia	0.06	0.95	0.06	0.13	-0.18	0.31	-0.05	0.14	-0.32	0.22		
Tanzania	0.06	0.95	0.06	0.08	-0.09	0.21	-0.05	0.06	-0.16	0.06		
Israel	0.06	0.95	0.06	0.13	-0.19	0.31	-0.05	0.15	-0.35	0.25		
Philippines	0.06	0.95	0.06	0.11	-0.15	0.27	-0.05	0.11	-0.26	0.16		
Mexico	0.06	0.95	0.06	0.11	-0.16	0.29	-0.05	0.12	-0.28	0.18		
Egypt	0.06	0.95	0.06	0.10	-0.13	0.26	-0.05	0.09	-0.22	0.12		
Nigeria	0.06	0.95	0.06	0.10	-0.13	0.25	-0.05	0.09	-0.22	0.12		
India	0.06	0.95	0.06	0.08	-0.09	0.21	-0.05	0.05	-0.16	0.06		
Brazil	0.06	0.95	0.06	0.09	-0.11	0.23	-0.05	0.07	-0.19	0.09		
South Africa	0.06	0.95	0.06	0.17	-0.27	0.40	-0.05	0.15	-0.34	0.24		
Argentina	0.06	0.95	0.06	0.10	-0.14	0.26	-0.05	0.09	-0.23	0.13		
Poland	0.06	1.40	0.06	0.06	-0.05	0.17	0.35	0.06	0.23	0.46		*
Turkey	0.06	0.95	0.06	0.29	-0.51	0.63	-0.05	0.20	-0.45	0.35		
Hong Kong	0.06	0.95	0.06	0.62	-1.15	1.27	-0.05	0.49	-1.00	0.90		
Spain	0.06	0.95	0.06	0.12	-0.17	0.29	-0.05	0.10	-0.25	0.15		
Australia	0.06	0.95	0.06	0.14	-0.20	0.33	-0.05	0.11	-0.26	0.16		
China	0.06	0.95	0.06	0.10	-0.13	0.25	-0.05	0.10	-0.24	0.14		
United Kingdom	0.06	0.95	0.06	0.13	-0.19	0.32	-0.05	0.10	-0.24	0.14		
Germany	0.06	0.95	0.06	0.09	-0.13	0.25	-0.05	0.08	-0.21	0.11		
United States	-0.17	1.13	-0.18	0.04	-0.26	-0.10	0.13	0.03	0.06	0.20	*	*
Sweden	-0.29	1.14	-0.30	0.05	-0.40	-0.20	0.13	0.04	0.05	0.22	*	*
Japan	-0.72	1.40	-0.74	0.04	-0.82	-0.66	0.35	0.04	0.27	0.43	*	*

Note. *significant mu/lambda identified from whether the 95% CI for the parameter contains 0.0. Due to rounding, sometimes the estimate may be '-0.00' [slightly below zero] or '0.00' [slightly above zero]. Standard errors estimated using the inverse of the observed information matrix and can sometimes be singular leading to no estimated standard error for some parameters.

2.12 Self-rated mental health

```
i <- i + 1
cur.var <- OUTCOME.LIST[i]
fit <- hetop_pml(
  data = df.raw |> filter(!is.na(ANNUAL_WEIGHT_C2))
  , var = cur.var
  , group = as.name("COUNTRY")
  , wgt = as.name("ANNUAL_WEIGHT_C2")
  , psu =  as.name("PSU")
  , strata = as.name("STRATA")
  , pen = exp(seq(-5,5,0.5))
  , pen.type="alf"
)

get_plot_latent_mean(fit)
```

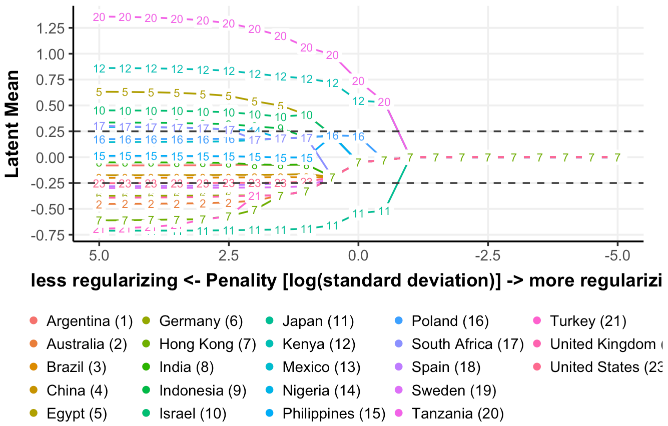


```
get_plot_discrimination(fit)
```

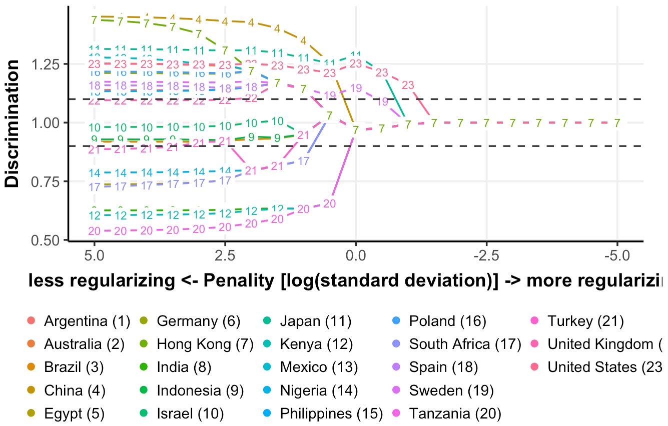


```
plot_iccs(fit, "icc")
```

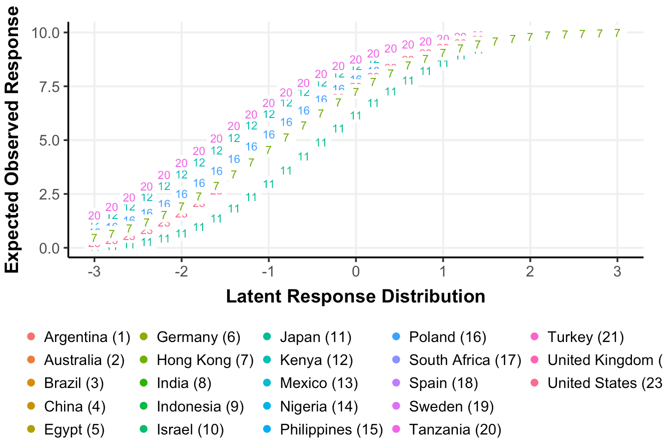


```
format_ft_hetop(fit, 2, cur.var)
```

Self-rated mental health (MENTAL_HEALTH_Y2): Threshold Table.

threshold	est.unstd	se.unstd	est.unstd.lb	est.unstd.ub
c[1]	-2.02	0.05	-2.13	-1.92
c[2]	-1.85	0.05	-1.94	-1.76

Self-rated mental health (MENTAL_HEALTH_Y2): Threshold Table.				
threshold	est.unstd	se.unstd	est.unstd.lb	est.unstd.ub
c[3]	-1.62	0.04	-1.70	-1.54
c[4]	-1.38	0.04	-1.45	-1.31
c[5]	-1.14	0.03	-1.20	-1.07
c[6]	-0.81	0.03	-0.87	-0.75
c[7]	-0.56	0.03	-0.61	-0.50
c[8]	-0.20	0.03	-0.26	-0.15
c[9]	0.25	0.03	0.19	0.31
c[10]	0.70	0.04	0.63	0.77

```
format_ft_hetop(fit, 1, cur.var)
```

Self-rated mental health (MENTAL_HEALTH_Y2): HETOP summary table when penality is nu=0.												
COUNTRY	mu.std	lambda.std	mu.unstd	mu.unstd.se	mu.unstd.lb	mu.unstd.ub	log.lambda.unstd	log.lambda.unstd.se	log.lambda.unstd.lb	log.lambda.unstd.ub	mu.nonzero	log.lambda.nonzero
Tanzania	0.71	0.97	0.74	0.08	0.57	0.90	-0.03	0.06	-0.15	0.08	*	
Kenya	0.53	0.97	0.55	0.07	0.41	0.68	-0.03	0.05	-0.13	0.07	*	
Poland	0.20	0.97	0.20	0.07	0.07	0.34	-0.03	0.07	-0.17	0.11	*	
Egypt	-0.05	0.97	-0.05	0.12	-0.29	0.19	-0.03	0.10	-0.23	0.17		
Israel	-0.05	0.97	-0.05	0.15	-0.35	0.25	-0.03	0.15	-0.32	0.26		
Indonesia	-0.05	0.97	-0.05	0.12	-0.28	0.18	-0.03	0.11	-0.24	0.18		
Nigeria	-0.05	0.97	-0.05	0.10	-0.24	0.14	-0.03	0.08	-0.19	0.13		
Mexico	-0.05	0.97	-0.05	0.12	-0.29	0.19	-0.03	0.13	-0.29	0.22		
Philippines	-0.05	0.97	-0.05	0.10	-0.25	0.15	-0.03	0.10	-0.22	0.16		
Argentina	-0.05	0.97	-0.05	0.09	-0.23	0.14	-0.03	0.09	-0.21	0.15		
South Africa	-0.05	0.97	-0.05	0.17	-0.38	0.28	-0.03	0.13	-0.29	0.23		
China	-0.05	0.97	-0.05	0.08	-0.20	0.10	-0.03	0.08	-0.19	0.13		
India	-0.05	0.97	-0.05	0.07	-0.19	0.09	-0.03	0.05	-0.12	0.06		
Brazil	-0.05	0.97	-0.05	0.08	-0.21	0.11	-0.03	0.06	-0.16	0.10		
Spain	-0.05	0.97	-0.05	0.10	-0.24	0.14	-0.03	0.09	-0.20	0.14		
Turkey	-0.05	0.97	-0.05	0.31	-0.66	0.56	-0.03	0.21	-0.44	0.38		
Hong Kong	-0.05	0.97	-0.05	0.28	-0.59	0.49	-0.03	0.25	-0.52	0.46		
Australia	-0.05	0.97	-0.05	0.11	-0.27	0.17	-0.03	0.10	-0.23	0.17		
United Kingdom	-0.05	0.97	-0.05	0.09	-0.23	0.13	-0.03	0.08	-0.18	0.12		
Germany	-0.05	0.97	-0.05	0.08	-0.20	0.10	-0.03	0.07	-0.16	0.10		
Sweden	-0.05	1.14	-0.05	0.05	-0.15	0.04	0.14	0.04	0.06	0.22		*
United States	-0.05	1.24	-0.05	0.04	-0.12	0.02	0.23	0.03	0.17	0.28		*
Japan	-0.52	1.28	-0.54	0.04	-0.62	-0.46	0.25	0.04	0.18	0.33	*	*

Note. *significant mu/lambda identified from whether the 95% CI for the parameter contains 0.0. Due to rounding, sometimes the estimate may be '-0.00' [slightly below zero] or '0.00' [slightly above zero]. Standard errors estimated using the inverse of the observed information matrix and can sometimes be singular leading to no estimated standard error for some parameters.

3 Psychological Distress

3.1 Traumatic distress

```
i <- i + 1
cur.var <- OUTCOME.LIST[i]
fit <- hetop_pml(
  data = df.raw |> filter(!is.na(ANNUAL_WEIGHT_C2))
  , var = cur.var
  , group = as.name("COUNTRY")
  , wgt = as.name("ANNUAL_WEIGHT_C2")
  , psu =  as.name("PSU")
  , strata = as.name("STRATA")
  , pen = exp(seq(-5,5,0.5))
  , pen.type="alf"
)

get_plot_latent_mean(fit)
```

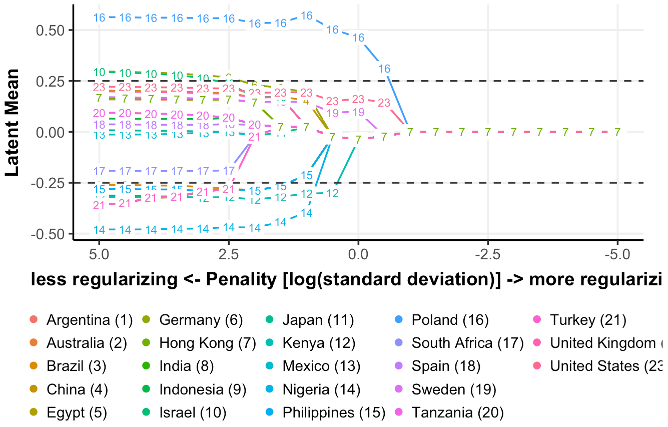


```
get_plot_discrimination(fit)
```

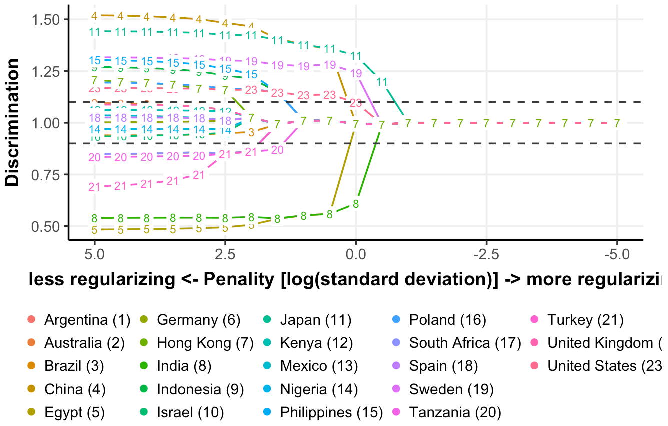


```
plot_iccs(fit, "icc")
```

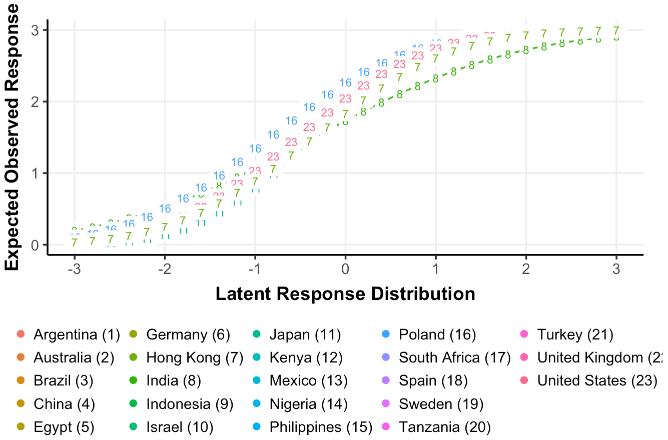

```r
format_ft_hetop(fit, 2, cur.var)
```

Traumatic distress (THREAT_LIFE_Y2): Threshold Table.				
threshold	est.unstd	se.unstd	est.unstd.lb	est.unstd.ub
c[1]	-1.18	0.03	-1.25	-1.12
c[2]	-0.37	0.03	-0.42	-0.32
c[3]	0.40	0.03	0.35	0.46

```r
format_ft_hetop(fit, 1, cur.var)
```

Traumatic distress (THREAT_LIFE_Y2): HETOP summary table when penality is nu=0.												
COUNTRY	mu.std	lambda.std	mu.unstd	mu.unstd.se	mu.unstd.lb	mu.unstd.ub	log.lambda.unstd	log.lambda.unstd.se	log.lambda.unstd.lb	log.lambda.unstd.ub	mu.nonzero	log.lambda.nonzero
Poland	0.45	1.00	0.46	0.07	0.32	0.61	-0.00	0.08	-0.17	0.16	*	
United States	0.16	1.10	0.16	0.04	0.09	0.23	0.09	0.04	0.02	0.17	*	*
Sweden	0.10	1.24	0.10	0.05	0.01	0.19	0.22	0.05	0.11	0.32	*	*
Germany	-0.03	1.00	-0.04	0.07	-0.18	0.11	-0.01	0.08	-0.16	0.15		
China	-0.03	1.00	-0.04	0.08	-0.20	0.12	-0.00	0.10	-0.21	0.20		
United Kingdom	-0.04	1.00	-0.04	0.09	-0.21	0.14	-0.00	0.10	-0.19	0.18		
Israel	-0.04	0.99	-0.04	0.11	-0.25	0.17	-0.01	0.11	-0.22	0.21		
Australia	-0.04	1.00	-0.04	0.10	-0.24	0.16	-0.01	0.11	-0.22	0.21		
Indonesia	-0.04	1.00	-0.04	0.10	-0.23	0.16	-0.00	0.11	-0.22	0.21		
Tanzania	-0.04	0.99	-0.04	0.07	-0.18	0.11	-0.01	0.07	-0.14	0.13		
Japan	-0.04	1.32	-0.04	0.04	-0.11	0.04	0.28	0.05	0.19	0.37		*
Hong Kong	-0.04	1.00	-0.04	0.19	-0.41	0.34	-0.01	0.22	-0.44	0.43		
Argentina	-0.04	0.99	-0.04	0.10	-0.23	0.15	-0.01	0.09	-0.19	0.18		
Spain	-0.04	1.00	-0.04	0.10	-0.22	0.15	-0.01	0.10	-0.20	0.19		
Mexico	-0.04	1.00	-0.04	0.11	-0.25	0.18	-0.01	0.11	-0.22	0.21		
Turkey	-0.04	0.99	-0.04	0.29	-0.60	0.52	-0.01	0.23	-0.46	0.45		
South Africa	-0.04	0.99	-0.04	0.18	-0.39	0.31	-0.01	0.16	-0.32	0.31		
India	-0.04	0.62	-0.04	0.13	-0.28	0.21	-0.50	0.09	-0.67	-0.32		*
Philippines	-0.04	1.00	-0.04	0.12	-0.27	0.20	-0.00	0.12	-0.24	0.23		
Egypt	-0.04	0.99	-0.04	0.12	-0.26	0.19	-0.01	0.09	-0.18	0.17		
Brazil	-0.04	0.99	-0.04	0.09	-0.22	0.15	-0.01	0.08	-0.17	0.16		
Nigeria	-0.04	0.99	-0.04	0.13	-0.30	0.23	-0.01	0.12	-0.23	0.22		
Kenya	-0.04	0.99	-0.04	0.08	-0.19	0.11	-0.01	0.07	-0.15	0.14		

Note. *significant mu/lambda identified from whether the 95% CI for the parameter contains 0.0. Due to rounding, sometimes the estimate may be '-0.00' [slightly below zero] or '0.00' [slightly above zero]. Standard errors estimated using the inverse of the observed information matrix and can sometimes be singular leading to no estimated standard error for some parameters.

3.2 Depression – feel hopeless

```r
i <- i + 1
cur.var <- OUTCOME.LIST[i]
fit <- hetop_pml(
  data = df.raw |> filter(!is.na(ANNUAL_WEIGHT_C2))
  , var = cur.var
  , group = as.name("COUNTRY")
  , wgt = as.name("ANNUAL_WEIGHT_C2")
  , psu =  as.name("PSU")
  , strata = as.name("STRATA")
  , pen = exp(seq(-5,5,0.5))
  , pen.type="alf"
```

```
)

get_plot_latent_mean(fit)
```

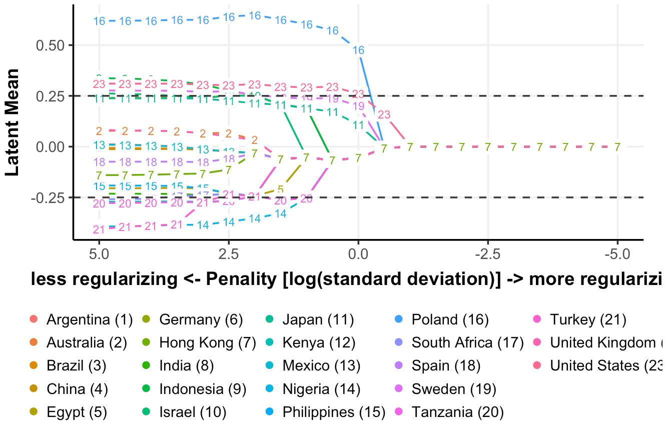


```
get_plot_discrimination(fit)
```

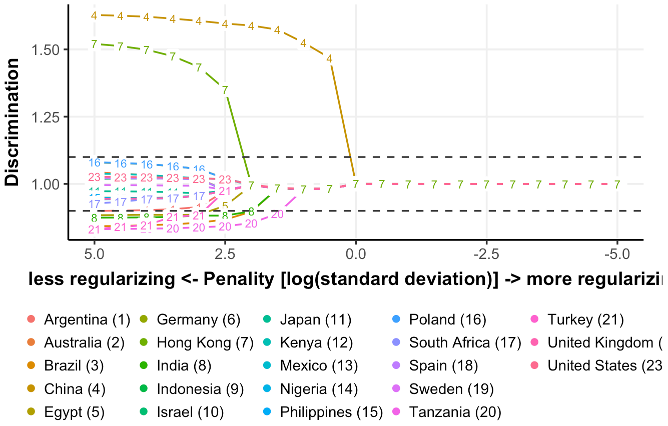


```
plot_iccs(fit, "icc")
```

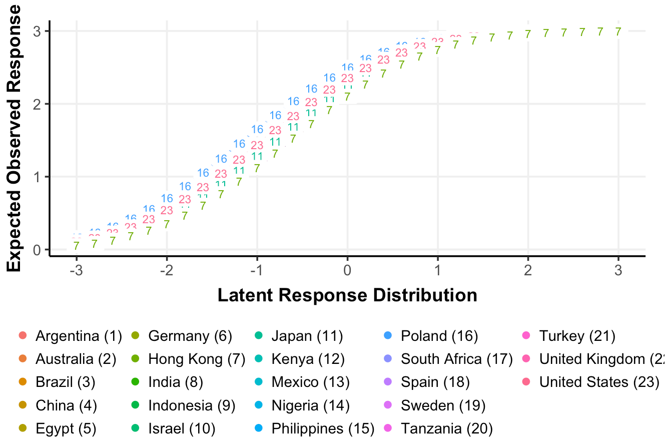


```
format_ft_hetop(fit, 2, cur.var)
```

Depression -- feel hopeless (DEPRESSED_Y2): Threshold Table.

threshold	est.unstd	se.unstd	est.unstd.lb	est.unstd.ub
c[1]	-1.34	0.03	-1.41	-1.28

Depression -- feel hopeless (DEPRESSED_Y2): Threshold Table.				
threshold	est.unstd	se.unstd	est.unstd.lb	est.unstd.ub
c[2]	-0.77	0.03	-0.82	-0.72
c[3]	0.10	0.03	0.05	0.15

```
format_ft_hetop(fit, 1, cur.var)
```

Depression -- feel hopeless (DEPRESSED_Y2): HETOP summary table when penality is nu=0.												
COUNTRY	mu.std	lambda.std	mu.unstd	mu.unstd.se	mu.unstd.lb	mu.unstd.ub	log.lambda.unstd	log.lambda.unstd.se	log.lambda.unstd.lb	log.lambda.unstd.ub	mu.nonzero	log.lambda.nonzero
Poland	0.47	1.00	0.48	0.08	0.31	0.64	0.00	0.09	-0.18	0.18	*	
United States	0.26	1.00	0.26	0.04	0.18	0.34	0.00	0.04	-0.08	0.08	*	
Sweden	0.20	1.00	0.20	0.06	0.08	0.32	0.00	0.06	-0.12	0.12	*	
Japan	0.11	1.00	0.11	0.05	0.00	0.21	0.00	0.05	-0.11	0.11	*	
Germany	-0.05	1.00	-0.05	0.07	-0.20	0.09	0.00	0.09	-0.18	0.18		
Indonesia	-0.05	1.00	-0.05	0.10	-0.26	0.15	0.00	0.12	-0.23	0.23		
Israel	-0.05	1.00	-0.05	0.11	-0.26	0.15	0.00	0.11	-0.21	0.21		
China	-0.05	1.00	-0.05	0.08	-0.21	0.10	0.00	0.11	-0.21	0.21		
United Kingdom	-0.05	1.00	-0.05	0.09	-0.23	0.12	-0.00					
Australia	-0.05	1.00	-0.05	0.10	-0.26	0.15	0.00					
Mexico	-0.05	1.00	-0.05	0.11	-0.27	0.16	-0.00	0.05	-0.10	0.10		
Argentina	-0.05	1.00	-0.05	0.10	-0.25	0.14	-0.00	0.05	-0.09	0.09		
Hong Kong	-0.05	1.00	-0.06	0.18	-0.42	0.31	0.00					
Spain	-0.05	1.00	-0.06	0.10	-0.26	0.15	0.00	0.10	-0.19	0.19		
Brazil	-0.05	1.00	-0.06	0.09	-0.23	0.12	-0.00	0.08	-0.16	0.16		
Turkey	-0.05	1.00	-0.06	0.23	-0.51	0.40	-0.00	0.06	-0.11	0.11		
South Africa	-0.05	1.00	-0.06	0.17	-0.38	0.27	-0.00	0.03	-0.07	0.07		
Philippines	-0.05	1.00	-0.06	0.11	-0.28	0.17	-0.00	0.10	-0.19	0.19		
Egypt	-0.05	1.00	-0.06	0.11	-0.27	0.16	-0.00	0.10	-0.20	0.20		
Nigeria	-0.05	1.00	-0.06	0.14	-0.33	0.22	-0.00	0.12	-0.24	0.24		
India	-0.06	1.00	-0.06	0.08	-0.22	0.11	-0.00	0.07	-0.15	0.15		
Tanzania	-0.06	1.00	-0.06	0.09	-0.24	0.13	-0.00	0.08	-0.16	0.16		
Kenya	-0.06	1.00	-0.06	0.08	-0.20	0.09	-0.00	0.07	-0.14	0.14		

Note. *significant mu/lambda identified from whether the 95% CI for the parameter contains 0.0. Due to rounding, sometimes the estimate may be '-0.00' [slightly below zero] or '0.00' [slightly above zero]. Standard errors estimated using the inverse of the observed information matrix and can sometimes be singular leading to no estimated standard error for some parameters.

3.3 Depression – loss of interest

```
i <- i + 1
cur.var <- OUTCOME.LIST[i]
fit <- hetop_pml(
  data = df.raw |> filter(!is.na(ANNUAL_WEIGHT_C2))
  , var = cur.var
  , group = as.name("COUNTRY")
  , wgt = as.name("ANNUAL_WEIGHT_C2")
  , psu =  as.name("PSU")
  , strata = as.name("STRATA")
  , pen = exp(seq(-5,5,0.5))
  , pen.type="alf"
)

get_plot_latent_mean(fit)
```

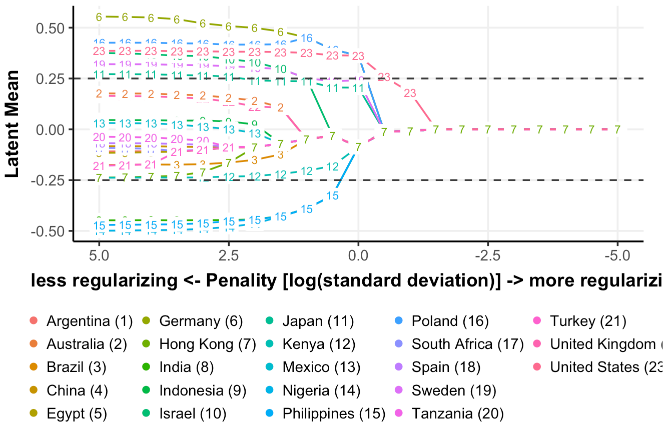


```
get_plot_discrimination(fit)
```

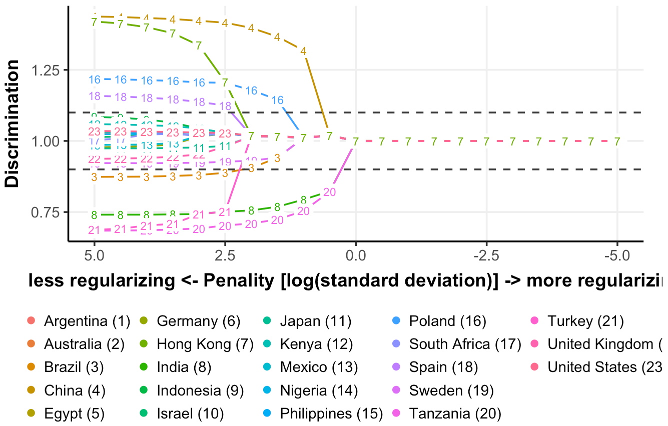


```
plot_iccs(fit, "icc")
```

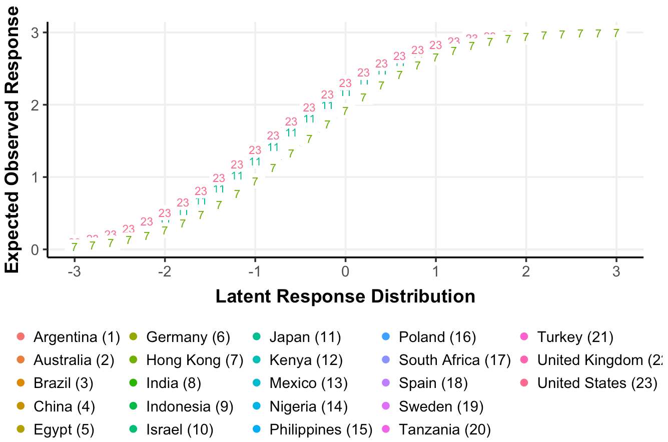


```
format_ft_hetop(fit, 2, cur.var)
```

Depression -- loss of interest (INTEREST_Y2): Threshold Table.

threshold	est.unstd	se.unstd	est.unstd.lb	est.unstd.ub
c[1]	-1.21	0.03	-1.27	-1.14
c[2]	-0.59	0.03	-0.64	-0.54

Depression -- loss of interest (INTEREST_Y2): Threshold Table.				
**threshold**	**est.unstd**	**se.unstd**	**est.unstd.lb**	**est.unstd.ub**
c[3]	0.28	0.03	0.23	0.33

```
format_ft_hetop(fit, 1, cur.var)
```

Depression -- loss of interest (INTEREST_Y2): HETOP summary table when penality is nu=0.

COUNTRY	mu.std	lambda.std	mu.unstd	mu.unstd.se	mu.unstd.lb	mu.unstd.ub	log.lambda.unstd	log.lambda.unstd.se	log.lambda.unstd.lb	log.lambda.unstd.ub	mu.nonzero	log.lambda.nonzero
United States	0.36	1.00	0.36	0.04	0.28	0.44	0.00	0.04	-0.08	0.08	*	
Poland	0.36	1.00	0.36	0.07	0.22	0.51	0.00	0.09	-0.17	0.17	*	
Germany	0.36	1.00	0.36	0.08	0.21	0.52	0.00	0.08	-0.16	0.16	*	
Sweden	0.24	1.00	0.24	0.06	0.13	0.35	-0.00	0.06	-0.11	0.11	*	
Japan	0.20	1.00	0.20	0.05	0.10	0.31	0.00	0.04	-0.08	0.08	*	
Israel	-0.08	1.00	-0.08	0.12	-0.32	0.15	0.00	0.14	-0.27	0.27		
United Kingdom	-0.08	1.00	-0.08	0.09	-0.26	0.09	-0.00	0.09	-0.18	0.18		
Australia	-0.08	1.00	-0.08	0.10	-0.29	0.12	0.00	0.13	-0.26	0.26		
Indonesia	-0.08	1.00	-0.08	0.10	-0.28	0.11	0.00	0.12	-0.23	0.23		
China	-0.08	1.00	-0.09	0.08	-0.24	0.07	0.00	0.09	-0.18	0.18		
Mexico	-0.08	1.00	-0.09	0.11	-0.30	0.13	0.00	0.11	-0.23	0.23		
Spain	-0.08	1.00	-0.09	0.10	-0.27	0.10	0.00	0.09	-0.18	0.18		
South Africa	-0.08	1.00	-0.09	0.16	-0.41	0.24	-0.00	0.16	-0.32	0.32		
Argentina	-0.08	1.00	-0.09	0.10	-0.27	0.10	0.00	0.01	-0.02	0.02		
Hong Kong	-0.08	1.00	-0.09	0.20	-0.48	0.31	0.00	0.21	-0.41	0.41		
Turkey	-0.08	1.00	-0.09	0.23	-0.53	0.36	-0.00	0.05	-0.10	0.10		
Egypt	-0.08	1.00	-0.09	0.09	-0.27	0.10	0.00	0.03	-0.05	0.05		
Tanzania	-0.08	1.00	-0.09	0.07	-0.23	0.06	-0.00	0.07	-0.13	0.13		
Brazil	-0.08	1.00	-0.09	0.09	-0.25	0.08	-0.00	0.08	-0.16	0.16		
Philippines	-0.08	1.00	-0.09	0.13	-0.34	0.17	-0.00	0.12	-0.23	0.23		
Kenya	-0.08	1.00	-0.09	0.07	-0.21	0.04	0.00	0.07	-0.13	0.13		
Nigeria	-0.08	1.00	-0.09	0.13	-0.33	0.16	-0.00	0.11	-0.22	0.22		
India	-0.08	1.00	-0.09	0.09	-0.25	0.08	-0.00	0.07	-0.15	0.15		

Note. *significant mu/lambda identified from whether the 95% CI for the parameter contains 0.0. Due to rounding, sometimes the estimate may be '-0.00' [slightly below zero] or '0.00' [slightly above zero]. Standard errors estimated using the inverse of the observed information matrix and can sometimes be singular leading to no estimated standard error for some parameters.

3.4 Anxiety – feel on edge

```
i <- i + 1
cur.var <- OUTCOME.LIST[i]
fit <- hetop_pml(
  data = df.raw |> filter(!is.na(ANNUAL_WEIGHT_C2))
  , var = cur.var
  , group = as.name("COUNTRY")
  , wgt = as.name("ANNUAL_WEIGHT_C2")
  , psu =  as.name("PSU")
  , strata = as.name("STRATA")
  , pen = exp(seq(-5,5,0.5))
  , pen.type="alf"
)

get_plot_latent_mean(fit)
```

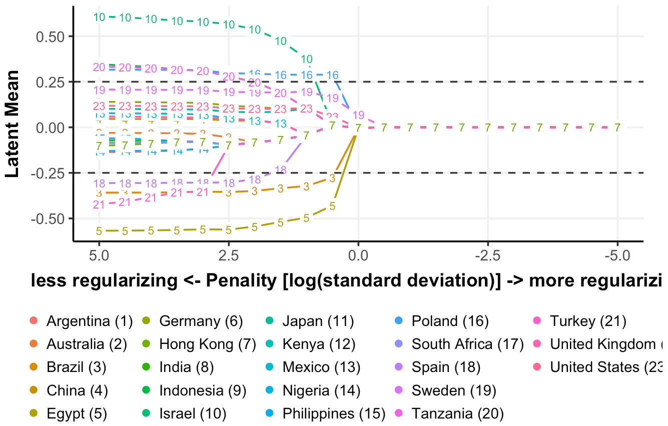


```
get_plot_discrimination(fit)
```

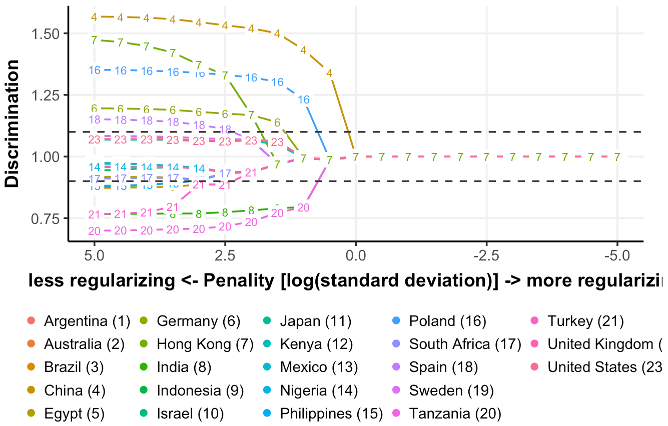


```
plot_iccs(fit, "icc")
```

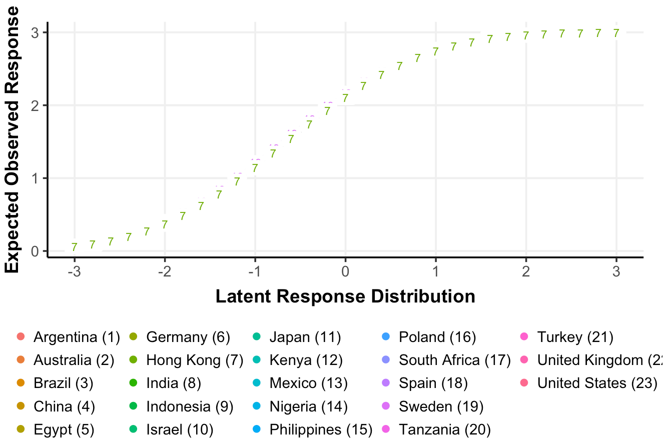


```
format_ft_hetop(fit, 2, cur.var)
```

Anxiety -- feel on edge (FEEL_ANXIOUS_Y2): Threshold Table.

threshold	est.unstd	se.unstd	est.unstd.lb	est.unstd.ub
c[1]	-1.32	0.03	-1.39	-1.25
c[2]	-0.76	0.03	-0.82	-0.70

Anxiety -- feel on edge (FEEL_ANXIOUS_Y2): Threshold Table.				
threshold	est.unstd	se.unstd	est.unstd.lb	est.unstd.ub
c[3]	0.19	0.04	0.11	0.26

```
format_ft_hetop(fit, 1, cur.var)
```

Anxiety -- feel on edge (FEEL_ANXIOUS_Y2): HETOP summary table when penality is nu=0.												
COUNTRY	mu.std	lambda.std	mu.unstd	mu.unstd.se	mu.unstd.lb	mu.unstd.ub	log.lambda.unstd	log.lambda.unstd.se	log.lambda.unstd.lb	log.lambda.unstd.ub	mu.nonzero	log.lambda.nonzero
Sweden	0.07	1.00	0.07	0.06	-0.05	0.18	0.00	0.06	-0.12	0.12		
United States	-0.00	1.00	-0.00	0.04	-0.09	0.08	0.00	0.04	-0.08	0.08		
Poland	-0.00	1.00	-0.00	0.07	-0.14	0.14	0.00	0.11	-0.21	0.21		
Israel	-0.00	1.00	-0.00	0.12	-0.24	0.23	-0.00	0.15	-0.28	0.28		
Japan	-0.00	1.00	-0.00	0.06	-0.11	0.11	0.00	0.06	-0.11	0.11		
Germany	-0.00	1.00	-0.00	0.08	-0.15	0.14	0.00	0.09	-0.17	0.17		
Indonesia	-0.00	1.00	-0.00	0.10	-0.21	0.20	-0.00	0.19	-0.37	0.37		
Tanzania	-0.00	1.00	-0.00	0.08	-0.16	0.15	-0.00	0.07	-0.14	0.14		
China	-0.00	1.00	-0.00	0.08	-0.16	0.16	0.00	0.10	-0.20	0.20		
Mexico	-0.00	1.00	-0.00	0.12	-0.25	0.24	-0.00	0.14	-0.28	0.28		
United Kingdom	-0.00	1.00	-0.00	0.17	-0.33	0.32	-0.00	0.38	-0.74	0.74		
Hong Kong	-0.00	1.00	-0.00	0.21	-0.41	0.41	0.00	0.21	-0.42	0.42		
South Africa	-0.00	1.00	-0.00	0.17	-0.35	0.34	-0.00	0.13	-0.25	0.25		
Australia	-0.00	1.00	-0.00	0.13	-0.25	0.25	0.00	0.15	-0.29	0.29		
Turkey	-0.00	1.00	-0.00	0.39	-0.77	0.76	-0.00	0.31	-0.60	0.60		
Philippines	-0.00	1.00	-0.00	0.13	-0.26	0.25	-0.00	0.12	-0.24	0.24		
Nigeria	-0.00	1.00	-0.00	0.11	-0.22	0.21	-0.00	0.09	-0.18	0.18		
Argentina	-0.00	1.00	-0.00	0.12	-0.23	0.22	-0.00	0.10	-0.20	0.19		
Spain	-0.00	1.00	-0.00	0.23	-0.45	0.44	-0.00	0.28	-0.54	0.54		
Kenya	-0.00	1.00	-0.00	0.07	-0.15	0.14	-0.00	0.07	-0.13	0.13		
India	-0.00	1.00	-0.00	0.08	-0.17	0.16	-0.00	0.07	-0.14	0.13		
Brazil	-0.00	1.00	-0.00	0.12	-0.24	0.23	-0.00	0.10	-0.19	0.19		
Egypt	-0.00	1.00	-0.00	0.21	-0.42	0.41	-0.00	0.17	-0.33	0.33		

Note. *significant mu/lambda identified from whether the 95% CI for the parameter contains 0.0. Due to rounding, sometimes the estimate may be '-0.00' [slightly below zero] or '0.00' [slightly above zero]. Standard errors estimated using the inverse of the observed information matrix and can sometimes be singular leading to no estimated standard error for some parameters.

3.5 Anxiety – cannot stop worrying

```
i <- i + 1
cur.var <- OUTCOME.LIST[i]
fit <- hetop_pml(
  data = df.raw |> filter(!is.na(ANNUAL_WEIGHT_C2))
  , var = cur.var
  , group = as.name("COUNTRY")
  , wgt = as.name("ANNUAL_WEIGHT_C2")
  , psu =  as.name("PSU")
  , strata = as.name("STRATA")
  , pen = exp(seq(-5,5,0.5))
  , pen.type="alf"
)

get_plot_latent_mean(fit)
```

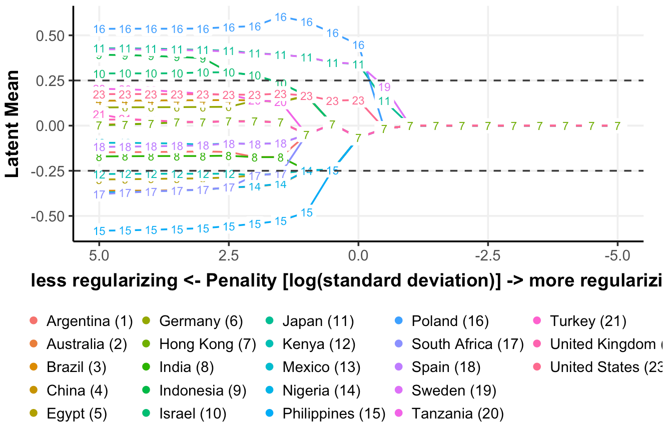


```
get_plot_discrimination(fit)
```

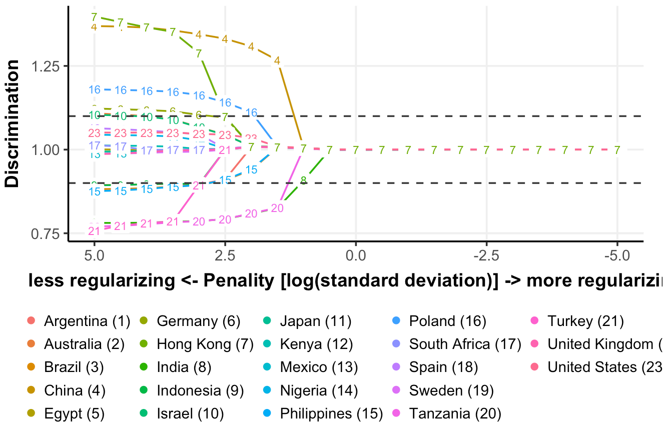


```
plot_iccs(fit, "icc")
```

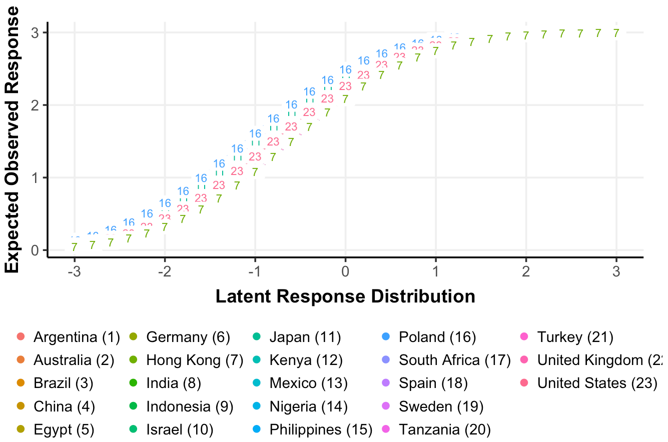


```
format_ft_hetop(fit, 2, cur.var)
```

Anxiety -- cannot stop worrying (CONTROL_WORRY_Y2): Threshold Table.

threshold	est.unstd	se.unstd	est.unstd.lb	est.unstd.ub
c[1]	-1.27	0.04	-1.34	-1.20
c[2]	-0.74	0.03	-0.80	-0.68

Anxiety -- cannot stop worrying (CONTROL_WORRY_Y2): Threshold Table.				
threshold	est.unstd	se.unstd	est.unstd.lb	est.unstd.ub
c[3]	0.05	0.03	-0.00	0.10

```
format_ft_hetop(fit, 1, cur.var)
```

Anxiety -- cannot stop worrying (CONTROL_WORRY_Y2): HETOP summary table when penality is nu=0.												
COUNTRY	mu.std	lambda.std	mu.unstd	mu.unstd.se	mu.unstd.lb	mu.unstd.ub	log.lambda.unstd	log.lambda.unstd.se	log.lambda.unstd.lb	log.lambda.unstd.ub	mu.nonzero	log.lambda.nonzero
Poland	0.44	1.00	0.45	0.09	0.28	0.62	0.00	0.10	-0.21	0.21	*	
Sweden	0.34	1.00	0.34	0.07	0.21	0.48	0.00	0.07	-0.14	0.14	*	
Japan	0.34	1.00	0.34	0.06	0.22	0.46	0.00	0.06	-0.12	0.12	*	
United States	0.14	1.00	0.14	0.04	0.06	0.23	0.00	0.04	-0.08	0.09	*	
China	-0.07	1.00	-0.07	0.08	-0.22	0.09	0.00	0.12	-0.24	0.24		
Germany	-0.07	1.00	-0.07	0.07	-0.21	0.08	0.00	0.09	-0.17	0.17		
Indonesia	-0.07	1.00	-0.07	0.11	-0.27	0.14	-0.00	0.14	-0.28	0.28		
Israel	-0.07	1.00	-0.07	0.11	-0.28	0.15	0.00					
Tanzania	-0.07	1.00	-0.07	0.08	-0.22	0.08	-0.00	0.08	-0.15	0.15		
United Kingdom	-0.07	1.00	-0.07	0.09	-0.25	0.11	0.00	0.10	-0.20	0.20		
Australia	-0.07	1.00	-0.07	0.10	-0.27	0.13	0.00					
Hong Kong	-0.07	1.00	-0.07	0.20	-0.45	0.32	0.00	0.28	-0.55	0.55		
Turkey	-0.07	1.00	-0.07	0.23	-0.51	0.38	-0.00					
Mexico	-0.07	1.00	-0.07	0.10	-0.27	0.14	-0.00					
Spain	-0.07	1.00	-0.07	0.09	-0.25	0.11	0.00					
South Africa	-0.07	1.00	-0.07	0.21	-0.48	0.35	-0.00	0.18	-0.35	0.35		
Argentina	-0.07	1.00	-0.07	0.11	-0.28	0.14	-0.00	0.09	-0.18	0.18		
Egypt	-0.07	1.00	-0.07	0.12	-0.30	0.16	-0.00	0.11	-0.22	0.22		
Nigeria	-0.07	1.00	-0.07	0.13	-0.32	0.19	-0.00	0.12	-0.24	0.24		
India	-0.07	1.00	-0.07	0.08	-0.23	0.10	-0.00	0.08	-0.15	0.15		
Brazil	-0.07	1.00	-0.07	0.11	-0.29	0.15	-0.00	0.10	-0.20	0.20		
Philippines	-0.07	1.00	-0.07	0.20	-0.46	0.33	-0.00	0.16	-0.32	0.32		
Kenya	-0.07	1.00	-0.07	0.08	-0.22	0.08	-0.00	0.07	-0.15	0.15		

Note. *significant mu/lambda identified from whether the 95% CI for the parameter contains 0.0. Due to rounding, sometimes the estimate may be '-0.00' [slightly below zero] or '0.00' [slightly above zero]. Standard errors estimated using the inverse of the observed information matrix and can sometimes be singular leading to no estimated standard error for some parameters.

3.6 Suffering

```
i <- i + 1
cur.var <- OUTCOME.LIST[i]
fit <- hetop_pml(
  data = df.raw |> filter(!is.na(ANNUAL_WEIGHT_C2))
  , var = cur.var
  , group = as.name("COUNTRY")
  , wgt = as.name("ANNUAL_WEIGHT_C2")
  , psu =  as.name("PSU")
  , strata = as.name("STRATA")
  , pen = exp(seq(-5,5,0.5))
  , pen.type="alf"
)

get_plot_latent_mean(fit)
```

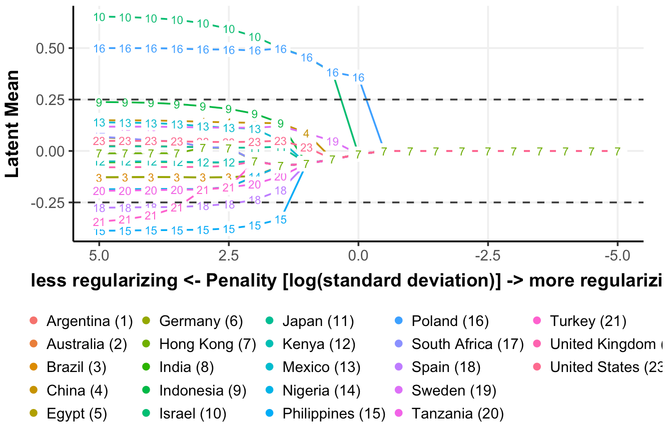


```
get_plot_discrimination(fit)
```

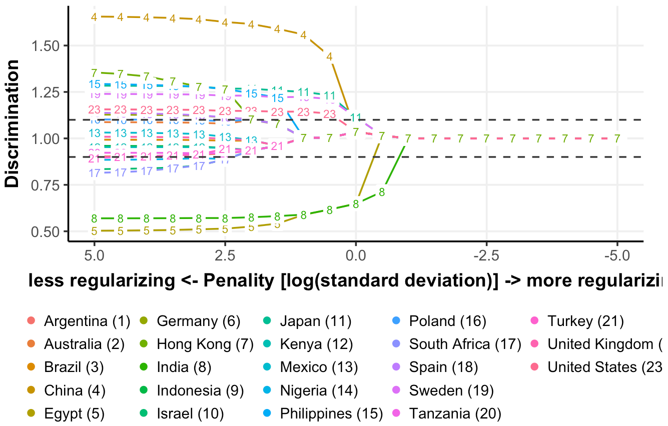


```
plot_iccs(fit, "icc")
```

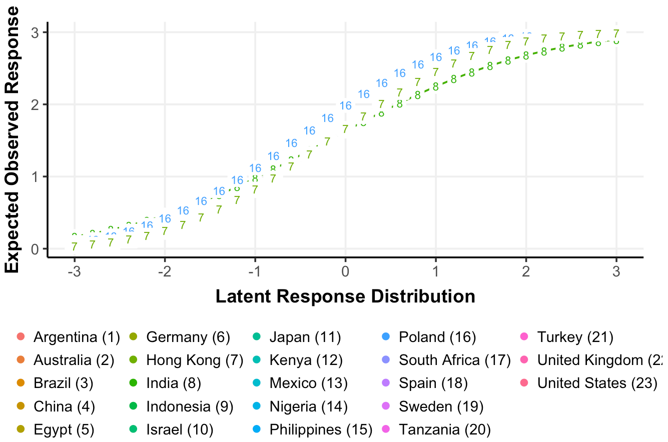


```
format_ft_hetop(fit, 2, cur.var)
```

Suffering (SUFFERING_Y2): Threshold Table.

threshold	est.unstd	se.unstd	est.unstd.lb	est.unstd.ub
c[1]	-1.28	0.03	-1.34	-1.21
c[2]	-0.15	0.02	-0.20	-0.10
c[3]	0.78	0.03	0.73	0.84

```r
format_ft_hetop(fit, 1, cur.var)
```

Suffering (SUFFERING_Y2): HETOP summary table when penality is nu=0.												
**COUNTRY**	**mu.std**	**lambda.std**	**mu.unstd**	**mu.unstd.se**	**mu.unstd.lb**	**mu.unstd.ub**	**log.lambda.unstd**	**log.lambda.unstd.se**	**log.lambda.unstd.lb**	**log.lambda.unstd.ub**	**mu.nonzero**	**log.lambda.nonzero**
Poland	0.35	1.03	0.36	0.07	0.23	0.49	0.03	0.07	-0.09	0.16	*	
Sweden	-0.01	1.11	-0.02	0.05	-0.11	0.08	0.11	0.05	0.02	0.20		*
United States	-0.02	1.04	-0.02	0.04	-0.08	0.05	0.04	0.03	-0.03	0.10		
Israel	-0.02	1.03	-0.02	0.13	-0.27	0.24	0.03	0.11	-0.19	0.26		
China	-0.02	1.03	-0.02	0.08	-0.16	0.13	0.04	0.09	-0.13	0.20		
Indonesia	-0.02	1.03	-0.02	0.10	-0.21	0.18	0.03	0.09	-0.14	0.21		
Mexico	-0.02	1.03	-0.02	0.10	-0.22	0.19	0.03	0.09	-0.15	0.22		
Japan	-0.02	1.11	-0.02	0.04	-0.10	0.07	0.11	0.04	0.02	0.19		*
South Africa	-0.02	1.03	-0.02	0.15	-0.32	0.29	0.03	0.13	-0.22	0.28		
Egypt	-0.02	0.66	-0.02	0.14	-0.30	0.26	-0.43	0.09	-0.62	-0.25		*
Argentina	-0.02	1.03	-0.02	0.09	-0.20	0.16	0.03	0.08	-0.12	0.19		
Hong Kong	-0.02	1.03	-0.02	0.18	-0.37	0.33	0.03	0.19	-0.33	0.40		
Turkey	-0.02	1.03	-0.02	0.25	-0.51	0.47	0.03	0.20	-0.36	0.43		
India	-0.02	0.66	-0.02	0.10	-0.22	0.18	-0.43	0.07	-0.57	-0.30		*
United Kingdom	-0.02	1.03	-0.02	0.08	-0.18	0.15	0.03	0.07	-0.11	0.18		
Australia	-0.02	1.03	-0.02	0.10	-0.22	0.18	0.03	0.09	-0.15	0.21		
Kenya	-0.02	1.03	-0.02	0.06	-0.13	0.10	0.03	0.05	-0.07	0.13		
Nigeria	-0.02	1.03	-0.02	0.09	-0.20	0.16	0.03	0.08	-0.11	0.18		
Brazil	-0.02	1.03	-0.02	0.08	-0.17	0.14	0.03	0.07	-0.10	0.17		
Germany	-0.02	1.03	-0.02	0.07	-0.15	0.12	0.03	0.06	-0.09	0.16		
Spain	-0.02	1.03	-0.02	0.10	-0.22	0.18	0.03	0.09	-0.14	0.21		
Philippines	-0.02	1.03	-0.02	0.12	-0.25	0.22	0.03	0.11	-0.18	0.25		
Tanzania	-0.02	1.03	-0.02	0.07	-0.16	0.13	0.03	0.06	-0.09	0.15		

Note. *significant mu/lambda identified from whether the 95% CI for the parameter contains 0.0. Due to rounding, sometimes the estimate may be '-0.00' [slightly below zero] or '0.00' [slightly above zero]. Standard errors estimated using the inverse of the observed information matrix and can sometimes be singular leading to no estimated standard error for some parameters.

4 Social Well-Being

4.1 Relationship contentment

```r
i <- i + 1
cur.var <- OUTCOME.LIST[i]
fit <- hetop_pml(
  data = df.raw |> filter(!is.na(ANNUAL_WEIGHT_C2))
  , var = cur.var
  , group = as.name("COUNTRY")
  , wgt = as.name("ANNUAL_WEIGHT_C2")
  , psu =  as.name("PSU")
  , strata = as.name("STRATA")
  , pen = exp(seq(-5,5,0.5))
  , pen.type="alf"
)

get_plot_latent_mean(fit)
```

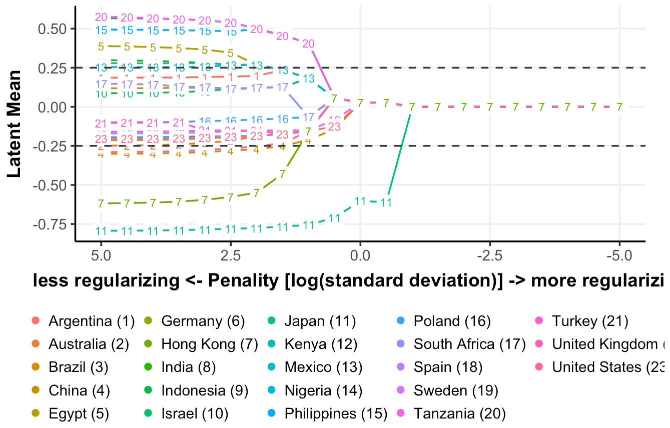


```
get_plot_discrimination(fit)
```

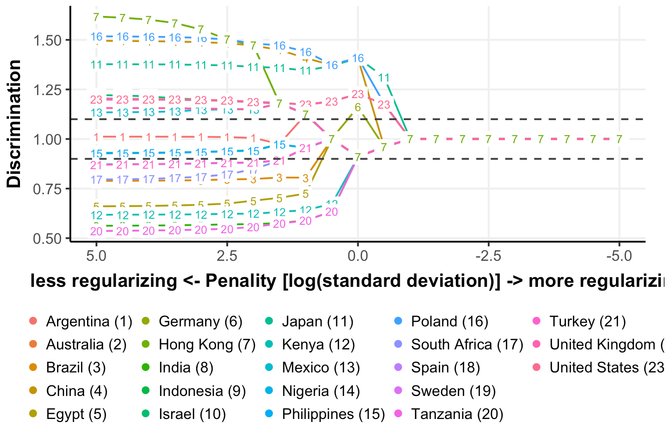


```
plot_iccs(fit, "icc")
```

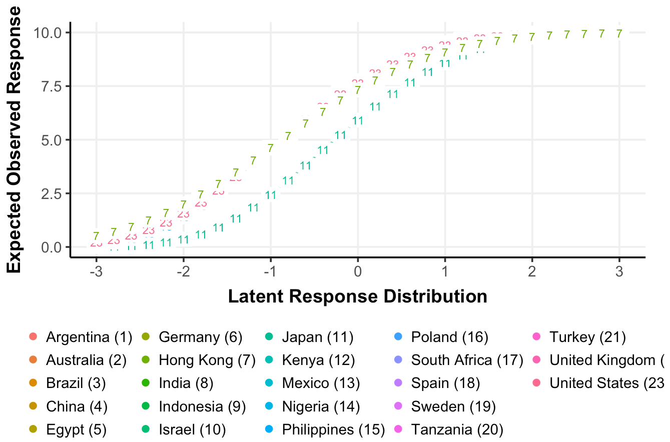


```
format_ft_hetop(fit, 2, cur.var)
```

Relationship contentment (CONTENT_Y2): Threshold Table.

threshold	est.unstd	se.unstd	est.unstd.lb	est.unstd.ub
c[1]	-1.84	0.05	-1.95	-1.74
c[2]	-1.70	0.05	-1.79	-1.61

Relationship contentment (CONTENT_Y2): Threshold Table.				
threshold	est.unstd	se.unstd	est.unstd.lb	est.unstd.ub
c[3]	-1.52	0.04	-1.60	-1.43
c[4]	-1.31	0.04	-1.39	-1.24
c[5]	-1.11	0.03	-1.18	-1.04
c[6]	-0.78	0.03	-0.84	-0.72
c[7]	-0.54	0.03	-0.60	-0.48
c[8]	-0.20	0.03	-0.26	-0.14
c[9]	0.24	0.04	0.17	0.31
c[10]	0.63	0.04	0.55	0.71

```
format_ft_hetop(fit, 1, cur.var)
```

Relationship contentment (CONTENT_Y2): HETOP summary table when penality is nu=0.

COUNTRY	mu.std	lambda.std	mu.unstd	mu.unstd.se	mu.unstd.lb	mu.unstd.ub	log.lambda.unstd	log.lambda.unstd.se	log.lambda.unstd.lb	log.lambda.unstd.ub	mu.nonzero	log.lambda.nonzero
Tanzania	0.03	0.91	0.03	0.08	-0.13	0.18	-0.09	0.06	-0.21	0.02		
Indonesia	0.03	0.91	0.03	0.17	-0.30	0.36	-0.09	0.17	-0.43	0.24		
Kenya	0.03	0.91	0.03	0.07	-0.11	0.16	-0.09	0.05	-0.20	0.01		
Philippines	0.03	0.91	0.03	0.14	-0.25	0.31	-0.09	0.14	-0.37	0.18		
India	0.03	0.91	0.03	0.07	-0.12	0.17	-0.09	0.05	-0.20	0.01		
Egypt	0.03	0.91	0.03	0.10	-0.18	0.23	-0.09	0.08	-0.26	0.07		
Mexico	0.03	0.91	0.03	0.13	-0.23	0.29	-0.09	0.14	-0.37	0.18		
Argentina	0.03	0.91	0.03	0.11	-0.18	0.24	-0.09	0.10	-0.30	0.11		
Brazil	0.03	0.91	0.03	0.09	-0.14	0.20	-0.09	0.07	-0.23	0.05		
Israel	0.03	0.91	0.03	0.11	-0.20	0.25	-0.09	0.12	-0.33	0.15		
Poland	0.03	1.40	0.03	0.05	-0.08	0.13	0.34	0.06	0.23	0.46		*
South Africa	0.03	0.91	0.03	0.17	-0.31	0.37	-0.09	0.15	-0.38	0.19		
Turkey	0.03	0.91	0.03	0.24	-0.45	0.50	-0.09	0.20	-0.49	0.30		
Spain	0.03	0.91	0.03	0.10	-0.17	0.23	-0.09	0.10	-0.29	0.11		
Nigeria	0.03	0.91	0.03	0.10	-0.18	0.23	-0.09	0.08	-0.26	0.07		
Australia	0.03	0.91	0.03	0.11	-0.19	0.25	-0.09	0.10	-0.30	0.11		
Hong Kong	0.03	0.91	0.03	0.41	-0.78	0.84	-0.09	0.39	-0.86	0.67		
United Kingdom	0.03	0.91	0.03	0.10	-0.17	0.22	-0.09	0.09	-0.27	0.08		
Germany	0.03	1.16	0.03	0.07	-0.10	0.16	0.15	0.06	0.03	0.27		*
China	0.03	1.39	0.03	0.06	-0.10	0.15	0.34	0.07	0.21	0.47		*
Sweden	0.03	1.22	0.03	0.05	-0.07	0.12	0.21	0.04	0.12	0.29		*
United States	0.02	1.22	0.03	0.04	-0.05	0.10	0.21	0.03	0.14	0.27		*
Japan	-0.58	1.39	-0.60	0.04	-0.68	-0.52	0.34	0.04	0.26	0.42	*	*

Note. *significant mu/lambda identified from whether the 95% CI for the parameter contains 0.0. Due to rounding, sometimes the estimate may be '-0.00' [slightly below zero] or '0.00' [slightly above zero]. Standard errors estimated using the inverse of the observed information matrix and can sometimes be singular leading to no estimated standard error for some parameters.

4.2 Relationship satisfaction

```r
i <- i + 1
cur.var <- OUTCOME.LIST[i]
fit <- hetop_pml(
  data = df.raw |> filter(!is.na(ANNUAL_WEIGHT_C2))
  , var = cur.var
  , group = as.name("COUNTRY")
  , wgt = as.name("ANNUAL_WEIGHT_C2")
  , psu =  as.name("PSU")
  , strata = as.name("STRATA")
  , pen = exp(seq(-5,5,0.5))
  , pen.type="alf"
)

get_plot_latent_mean(fit)
```

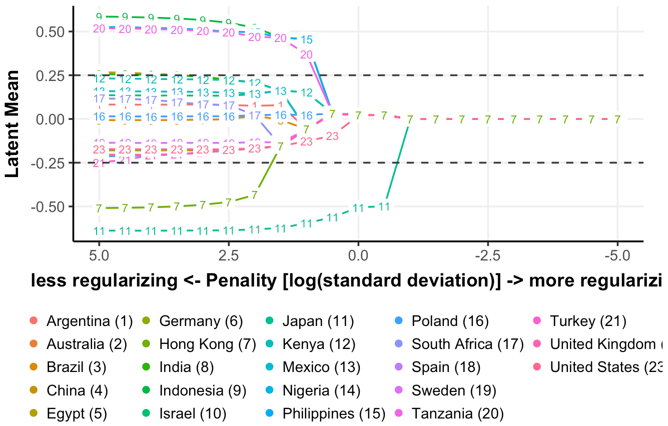


```r
get_plot_discrimination(fit)
```

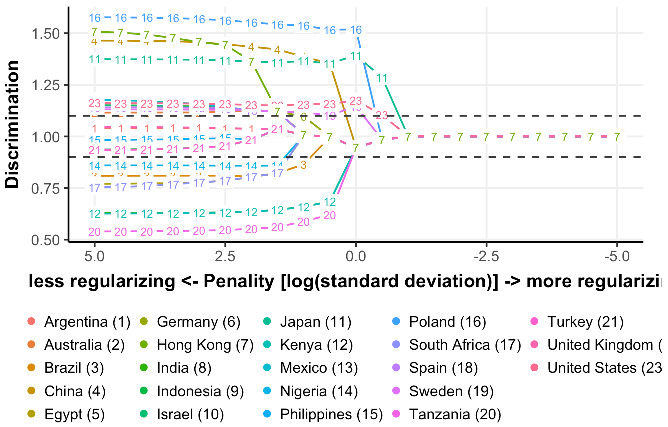


```r
plot_iccs(fit, "icc")
```

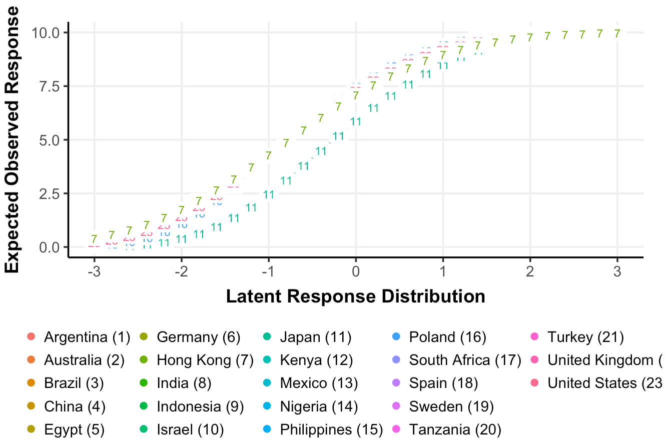

```
format_ft_hetop(fit, 2, cur.var)
```

Relationship satisfaction (SAT_RELATNSHP_Y2): Threshold Table.				
**threshold**	**est.unstd**	**se.unstd**	**est.unstd.lb**	**est.unstd.ub**
c[1]	-1.76	0.05	-1.85	-1.67
c[2]	-1.62	0.04	-1.70	-1.53
c[3]	-1.44	0.04	-1.51	-1.36
c[4]	-1.21	0.03	-1.28	-1.15
c[5]	-1.00	0.03	-1.06	-0.94
c[6]	-0.67	0.03	-0.73	-0.62
c[7]	-0.42	0.03	-0.47	-0.37
c[8]	-0.08	0.03	-0.13	-0.02
c[9]	0.38	0.03	0.32	0.44
c[10]	0.79	0.04	0.72	0.86

```
format_ft_hetop(fit, 1, cur.var)
```

Relationship satisfaction (SAT_RELATNSHP_Y2): HETOP summary table when penality is nu=0.												
**COUNTRY**	**mu.std**	**lambda.std**	**mu.unstd**	**mu.unstd.se**	**mu.unstd.lb**	**mu.unstd.ub**	**log.lambda.unstd**	**log.lambda.unstd.se**	**log.lambda.unstd.lb**	**log.lambda.unstd.ub**	**mu.nonzero**	**log.lambda.nonzero**
Indonesia	0.02	0.95	0.02	0.15	-0.27	0.32	-0.06	0.14	-0.33	0.22		
Tanzania	0.02	0.95	0.02	0.07	-0.12	0.17	-0.06	0.05	-0.16	0.05		
Philippines	0.02	0.95	0.02	0.14	-0.26	0.30	-0.06	0.13	-0.32	0.21		
Poland	0.02	1.51	0.02	0.05	-0.07	0.12	0.42	0.05	0.31	0.52		*
Kenya	0.02	0.95	0.02	0.06	-0.10	0.15	-0.06	0.05	-0.15	0.04		
India	0.02	0.95	0.02	0.07	-0.11	0.16	-0.06	0.05	-0.15	0.04		
Egypt	0.02	0.95	0.02	0.10	-0.17	0.21	-0.06	0.08	-0.21	0.10		
Mexico	0.02	0.95	0.02	0.11	-0.20	0.25	-0.05	0.11	-0.28	0.17		
Israel	0.02	0.95	0.02	0.11	-0.19	0.23	-0.05	0.11	-0.26	0.15		
Argentina	0.02	0.95	0.02	0.10	-0.17	0.21	-0.05	0.09	-0.23	0.12		
Brazil	0.02	0.95	0.02	0.08	-0.14	0.18	-0.06	0.06	-0.18	0.07		
South Africa	0.02	0.95	0.02	0.16	-0.30	0.35	-0.06	0.13	-0.30	0.19		
Turkey	0.02	0.95	0.02	0.25	-0.46	0.50	-0.05	0.20	-0.44	0.33		
Australia	0.02	0.95	0.02	0.11	-0.18	0.23	-0.05	0.09	-0.24	0.13		
Hong Kong	0.02	0.95	0.02	0.30	-0.57	0.61	-0.05	0.29	-0.61	0.50		
Spain	0.02	0.95	0.02	0.10	-0.17	0.22	-0.05	0.09	-0.23	0.12		
China	0.02	0.95	0.02	0.08	-0.13	0.18	-0.05	0.09	-0.22	0.11		
United Kingdom	0.02	0.95	0.02	0.09	-0.16	0.20	-0.05	0.08	-0.21	0.10		
Nigeria	0.02	0.95	0.02	0.10	-0.17	0.22	-0.06	0.08	-0.20	0.09		
Germany	0.02	0.95	0.02	0.07	-0.12	0.17	-0.05	0.07	-0.19	0.08		
Sweden	0.02	1.14	0.02	0.05	-0.07	0.12	0.14	0.04	0.05	0.22		*
United	0.02	1.17	0.02	0.04	-0.05	0.09	0.16	0.03	0.10	0.22		*

Note. *significant mu/lambda identified from whether the 95% CI for the parameter contains 0.0. Due to rounding, sometimes the estimate may be '-0.00' [slightly below zero] or '0.00' [slightly above zero]. Standard errors estimated using the inverse of the observed information matrix and can sometimes be singular leading to no estimated standard error for some parameters.

Relationship satisfaction (SAT_RELATNSHP_Y2): HETOP summary table when penality is nu=0.												
COUNTRY	mu.std	lambda.std	mu.unstd	mu.unstd.se	mu.unstd.lb	mu.unstd.ub	log.lambda.unstd	log.lambda.unstd.se	log.lambda.unstd.lb	log.lambda.unstd.ub	mu.nonzero	log.lambda.nonzero
States												
Japan	-0.50	1.38	-0.51	0.04	-0.58	-0.43	0.33	0.04	0.26	0.41	*	*

Note. *significant mu/lambda identified from whether the 95% CI for the parameter contains 0.0. Due to rounding, sometimes the estimate may be '-0.00' [slightly below zero] or '0.00' [slightly above zero]. Standard errors estimated using the inverse of the observed information matrix and can sometimes be singular leading to no estimated standard error for some parameters.

4.3 Social support

```
i <- i + 1
cur.var <- OUTCOME.LIST[i]
fit <- hetop_pml(
  data = df.raw |> filter(!is.na(ANNUAL_WEIGHT_C2))
  , var = cur.var
  , group = as.name("COUNTRY")
  , wgt = as.name("ANNUAL_WEIGHT_C2")
  , psu =  as.name("PSU")
  , strata = as.name("STRATA")
  , pen = exp(seq(-5,5,0.5))
  , pen.type="alf"
)

get_plot_latent_mean(fit)
```

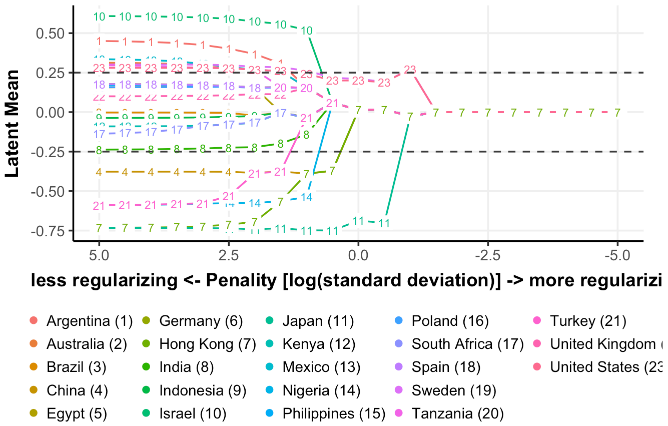


```
get_plot_discrimination(fit)
```

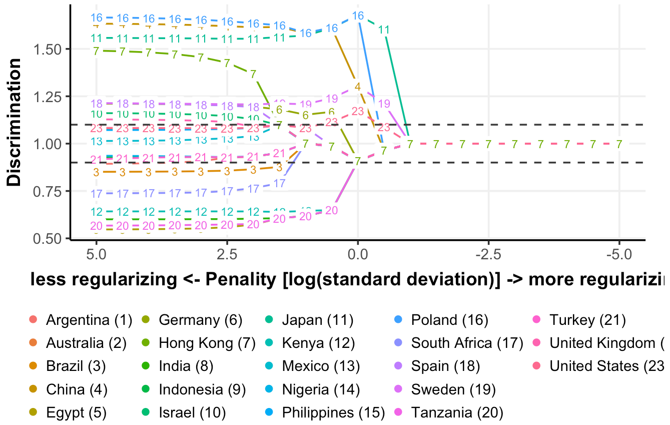


```
plot_iccs(fit, "icc")
```

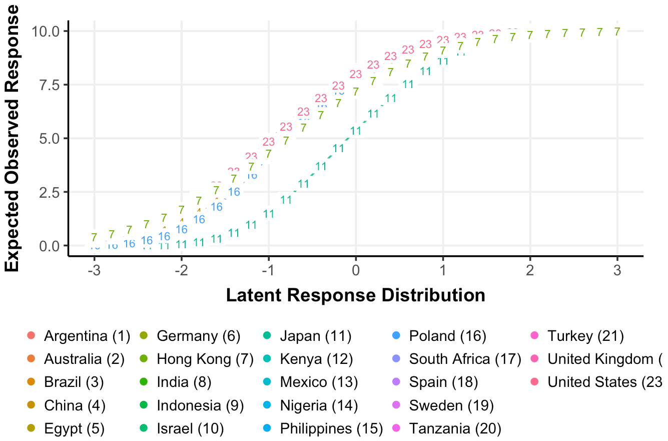


```
format_ft_hetop(fit, 2, cur.var)
```

Social support (PEOPLE_HELP_Y2): Threshold Table.

threshold	est.unstd	se.unstd	est.unstd.lb	est.unstd.ub
c[1]	-1.65	0.05	-1.75	-1.55
c[2]	-1.50	0.05	-1.59	-1.41
c[3]	-1.32	0.04	-1.41	-1.24
c[4]	-1.14	0.04	-1.22	-1.07
c[5]	-0.99	0.04	-1.06	-0.92
c[6]	-0.69	0.03	-0.75	-0.63
c[7]	-0.48	0.03	-0.54	-0.42
c[8]	-0.21	0.03	-0.26	-0.16
c[9]	0.15	0.03	0.11	0.20
c[10]	0.47	0.03	0.42	0.52

```
format_ft_hetop(fit, 1, cur.var)
```

Social support (PEOPLE_HELP_Y2): HETOP summary table when penality is nu=0.

COUNTRY	mu.std	lambda.std	mu.unstd	mu.unstd.se	mu.unstd.lb	mu.unstd.ub	log.lambda.unstd	log.lambda.unstd.se	log.lambda.unstd.lb	log.lambda.unstd.ub	mu.nonzero	log.lambda.nonzero
Sweden	0.20	1.29	0.21	0.04	0.13	0.29	0.26	0.05	0.17	0.36	*	*
United States	0.19	1.16	0.20	0.03	0.13	0.27	0.16	0.03	0.09	0.22	*	*
Poland	0.01	1.64	0.01	0.04	-0.07	0.10	0.52	0.06	0.40	0.63		*
Israel	0.01	0.91	0.01	0.25	-0.47	0.50	-0.10	0.30	-0.68	0.49		
Germany	0.01	0.91	0.01	0.08	-0.14	0.16	-0.09	0.09	-0.26	0.07		
Argentina	0.01	0.91	0.01	0.11	-0.20	0.23	-0.10	0.11	-0.31	0.12		
Australia	0.01	0.91	0.01	0.11	-0.21	0.24	-0.10	0.12	-0.34	0.15		
Mexico	0.01	0.91	0.01	0.12	-0.22	0.25	-0.10	0.13	-0.35	0.15		
Spain	0.01	0.91	0.01	0.10	-0.19	0.21	-0.10	0.12	-0.32	0.13		
Philippines	0.01	0.91	0.01	0.11	-0.19	0.22	-0.10	0.11	-0.31	0.12		
United Kingdom	0.01	0.91	0.01	0.09	-0.17	0.19	-0.10	0.10	-0.28	0.09		
Tanzania	0.01	0.91	0.01	0.08	-0.14	0.17	-0.10	0.06	-0.21	0.02		

Note. *significant mu/lambda identified from whether the 95% CI for the parameter contains 0.0. Due to rounding, sometimes the estimate may be '-0.00' [slightly below zero] or '0.00' [slightly above zero]. Standard errors estimated using the inverse of the observed information matrix and can sometimes be singular leading to no estimated standard error for some parameters.

Social support (PEOPLE_HELP_Y2): HETOP summary table when penality is nu=0.												
COUNTRY	mu.std	lambda.std	mu.unstd	mu.unstd.se	mu.unstd.lb	mu.unstd.ub	log.lambda.unstd	log.lambda.unstd.se	log.lambda.unstd.lb	log.lambda.unstd.ub	mu.nonzero	log.lambda.nonzero
Indonesia	0.01	0.91	0.01	0.11	-0.20	0.23	-0.10	0.10	-0.29	0.10		
Egypt	0.01	0.91	0.01	0.11	-0.19	0.22	-0.10	0.08	-0.25	0.06		
Brazil	0.01	0.91	0.01	0.09	-0.16	0.19	-0.10	0.08	-0.24	0.05		
South Africa	0.01	0.91	0.01	0.19	-0.37	0.39	-0.10	0.15	-0.39	0.20		
Turkey	0.01	0.91	0.01	0.43	-0.83	0.86	-0.10	0.31	-0.70	0.51		
Hong Kong	0.01	0.91	0.01				-0.10					
Kenya	0.01	0.91	0.01	0.07	-0.13	0.16	-0.10	0.05	-0.20	0.01		
Nigeria	0.01	0.91	0.01	0.18	-0.34	0.36	-0.10	0.13	-0.35	0.16		
India	0.01	0.91	0.01	0.08	-0.15	0.18	-0.10	0.06	-0.21	0.02		
China	0.01	1.29	0.01	0.10	-0.18	0.20	0.27	0.11	0.05	0.48		*
Japan	-0.66	1.64	-0.69	0.04	-0.76	-0.61	0.52	0.04	0.44	0.59	*	*

Note. *significant mu/lambda identified from whether the 95% CI for the parameter contains 0.0. Due to rounding, sometimes the estimate may be '-0.00' [slightly below zero] or '0.00' [slightly above zero]. Standard errors estimated using the inverse of the observed information matrix and can sometimes be singular leading to no estimated standard error for some parameters.

4.4 Intimate/close friend

```
i <- i + 1
cur.var <- OUTCOME.LIST[i]
fit <- hetop_pml(
  data = df.raw |> filter(!is.na(ANNUAL_WEIGHT_C2))
  , var = cur.var
  , group = as.name("COUNTRY")
  , wgt = as.name("ANNUAL_WEIGHT_C2")
  , psu =  as.name("PSU")
  , strata = as.name("STRATA")
  , pen = exp(seq(-5,5,0.5))
  , pen.type="alf"
)

get_plot_latent_mean(fit)
```

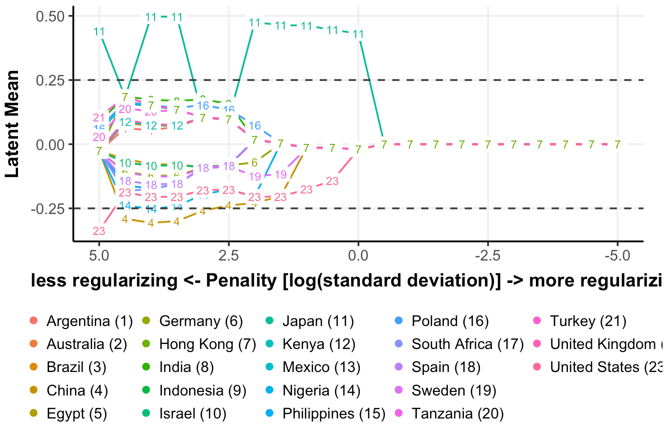


```
get_plot_discrimination(fit)
```

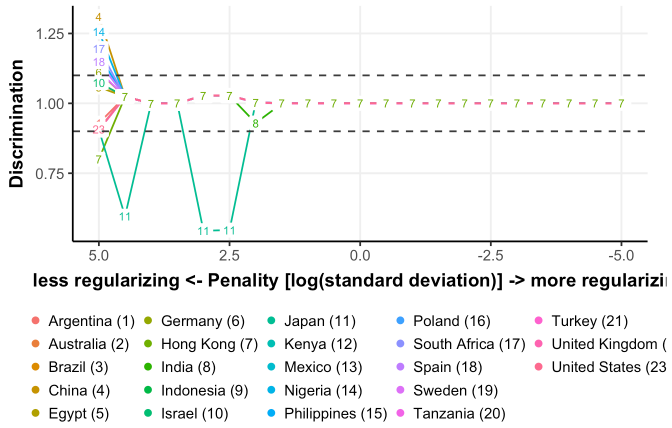


```
plot_iccs(fit, "icc")
```

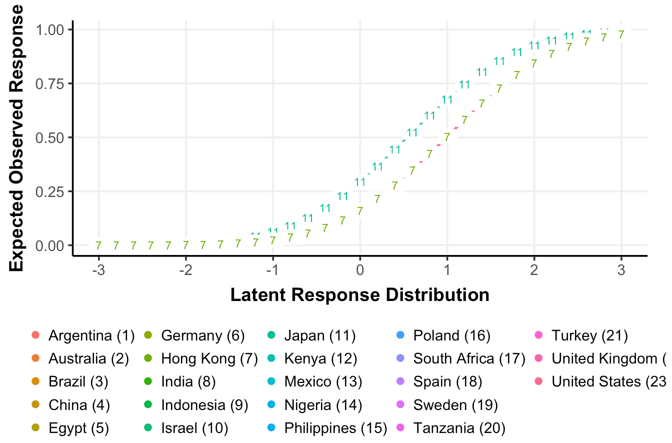


```
format_ft_hetop(fit, 2, cur.var)
```

Intimate/close friend (CLOSE_TO_Y2): Threshold Table.

threshold	est.unstd	se.unstd	est.unstd.lb	est.unstd.ub
c[1]	0.97			

```
format_ft_hetop(fit, 1, cur.var)
```

Intimate/close friend (CLOSE_TO_Y2): HETOP summary table when penality is nu=0.

COUNTRY	mu.std	lambda.std	mu.unstd	mu.unstd.se	mu.unstd.lb	mu.unstd.ub	log.lambda.unstd	log.lambda.unstd.se	log.lambda.unstd.lb	log.lambda.unstd.ub	mu.nonzero	log.lambda.nonzero
Japan	0.43	1.00	0.43				-0.00					
India	-0.02	1.00	-0.02				-0.00					
Poland	-0.02	1.00	-0.02				-0.00					
Tanzania	-0.02	1.00	-0.02				-0.00					
Kenya	-0.02	1.00	-0.02				-0.00					
United Kingdom	-0.02	1.00	-0.02				-0.00					
Brazil	-0.02	1.00	-0.02				-0.00					
Mexico	-0.02	1.00	-0.02				-0.00					
Indonesia	-0.02	1.00	-0.02				-0.00					

Note. *significant mu/lambda identified from whether the 95% CI for the parameter contains 0.0. Due to rounding, sometimes the estimate may be '-0.00' [slightly below zero] or '0.00' [slightly above zero]. Standard errors estimated using the inverse of the observed information matrix and can sometimes be singular leading to no estimated standard error for some parameters.

Intimate/close friend (CLOSE_TO_Y2): HETOP summary table when penality is nu=0.												
**COUNTRY**	**mu.std**	**lambda.std**	**mu.unstd**	**mu.unstd.se**	**mu.unstd.lb**	**mu.unstd.ub**	**log.lambda.unstd**	**log.lambda.unstd.se**	**log.lambda.unstd.lb**	**log.lambda.unstd.ub**	**mu.nonzero**	**log.lambda.nonzero**
Argentina	-0.02	1.00	-0.02				-0.00					
Hong Kong	-0.02	1.00	-0.02				-0.00					
Turkey	-0.02	1.00	-0.02	0.38	-0.76	0.72	-0.00	0.19	-0.36	0.36		
Egypt	-0.02	1.00	-0.02				0.00					
Australia	-0.02	1.00	-0.02				0.00					
Israel	-0.02	1.00	-0.02				0.00					
South Africa	-0.02	1.00	-0.02	0.40	-0.81	0.77	0.00	0.34	-0.67	0.67		
Spain	-0.02	1.00	-0.02				0.00					
Philippines	-0.02	1.00	-0.02				0.00					
Germany	-0.02	1.00	-0.02				0.00					
Nigeria	-0.02	1.00	-0.02	0.13	-0.28	0.24	0.00					
China	-0.02	1.00	-0.02				0.00					
Sweden	-0.02	1.00	-0.02				0.00					
United States	-0.02	1.00	-0.02	0.03	-0.09	0.05	0.00	0.09	-0.17	0.17		

Note. *significant mu/lambda identified from whether the 95% CI for the parameter contains 0.0. Due to rounding, sometimes the estimate may be '-0.00' [slightly below zero] or '0.00' [slightly above zero]. Standard errors estimated using the inverse of the observed information matrix and can sometimes be singular leading to no estimated standard error for some parameters.

4.5 Government approval

```r
i <- i + 1
cur.var <- OUTCOME.LIST[i]
fit <- hetop_pml(
  data = df.raw |> filter(!is.na(ANNUAL_WEIGHT_C2))
  , var = cur.var
  , group = as.name("COUNTRY")
  , wgt = as.name("ANNUAL_WEIGHT_C2")
  , psu =  as.name("PSU")
  , strata = as.name("STRATA")
  , pen = exp(seq(-5,5,0.5))
  , pen.type="alf"
)

get_plot_latent_mean(fit)
```

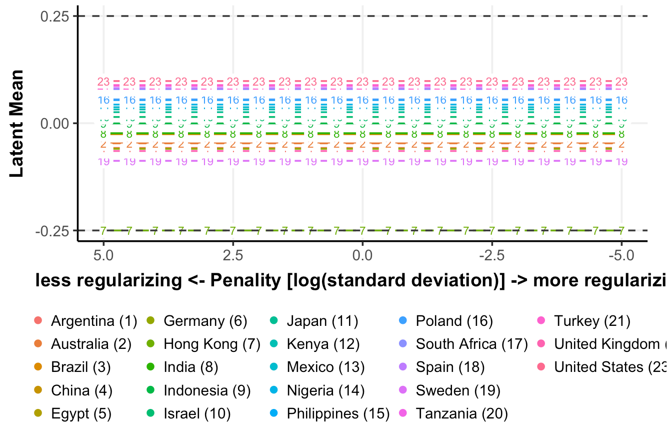


```r
get_plot_discrimination(fit)
```

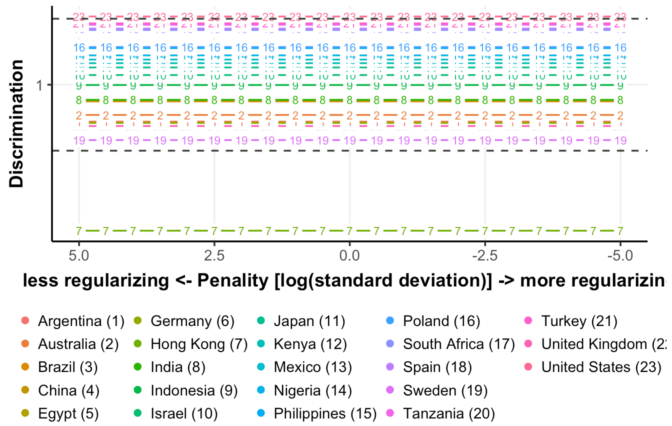


```
plot_iccs(fit, "icc")
```

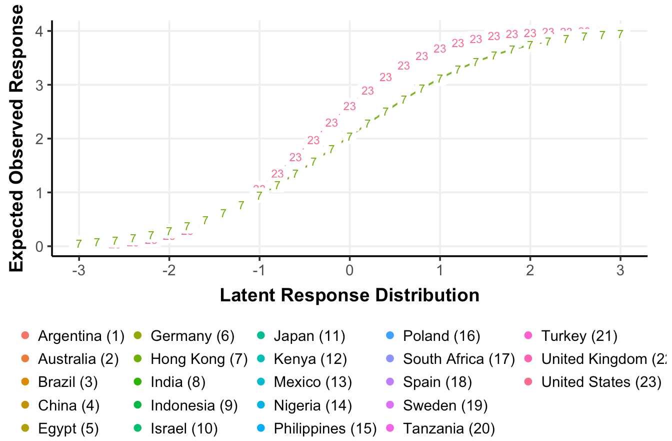


```
format_ft_hetop(fit, 2, cur.var)
```

Government approval (APPROVE_GOVT_Y2): Threshold Table.

threshold	est.unstd	se.unstd	est.unstd.lb	est.unstd.ub
c[1]	-0.75			
c[2]	-0.47			
c[3]	-0.23			
c[4]	0.30			

```
format_ft_hetop(fit, 1, cur.var)
```

Government approval (APPROVE_GOVT_Y2): HETOP summary table when penality is nu=0.

COUNTRY	mu.std	lambda.std	mu.unstd	mu.unstd.se	mu.unstd.lb	mu.unstd.ub	log.lambda.unstd	log.lambda.unstd.se	log.lambda.unstd.lb	log.lambda.unstd.ub	mu.nonzero	log.lambda.nonzero
Sweden			-0.09				-0.09					
United Kingdom			-0.06				-0.06					
Germany			-0.06				-0.06					
China			-0.06				-0.06					
Argentina			-0.06				-0.06					

Note. *significant mu/lambda identified from whether the 95% CI for the parameter contains 0.0. Due to rounding, sometimes the estimate may be '-0.00' [slightly below zero] or '0.00' [slightly above zero]. Standard errors estimated using the inverse of the observed information matrix and can sometimes be singular leading to no estimated standard error for some parameters.

Government approval (APPROVE_GOVT_Y2): HETOP summary table when penality is nu=0.												
COUNTRY	mu.std	lambda.std	mu.unstd	mu.unstd.se	mu.unstd.lb	mu.unstd.ub	log.lambda.unstd	log.lambda.unstd.se	log.lambda.unstd.lb	log.lambda.unstd.ub	mu.nonzero	log.lambda.nonzero
Australia			-0.05				-0.05					
Brazil			-0.03				-0.03					
Egypt			-0.02				-0.02					
India			-0.02				-0.02					
Indonesia			-0.00				-0.00					
Israel			0.01				0.01					
Japan			0.03				0.03					
Kenya			0.03				0.03					
Mexico			0.04				0.04					
Nigeria			0.04				0.04					
Philippines			0.05				0.05					
Poland			0.06				0.06					
South Africa			0.08				0.08					
Spain			0.08				0.08					
Tanzania			0.09				0.09					
Turkey			0.09				0.09					
United States			0.10				0.10					
Hong Kong			-0.25				-0.25					

Note. *significant mu/lambda identified from whether the 95% CI for the parameter contains 0.0. Due to rounding, sometimes the estimate may be '-0.00' [slightly below zero] or '0.00' [slightly above zero]. Standard errors estimated using the inverse of the observed information matrix and can sometimes be singular leading to no estimated standard error for some parameters.

4.6 Say in government

```
i <- i + 1
cur.var <- OUTCOME.LIST[i]
fit <- hetop_pml(
  data = df.raw |> filter(!is.na(ANNUAL_WEIGHT_C2))
  , var = cur.var
  , group = as.name("COUNTRY")
  , wgt = as.name("ANNUAL_WEIGHT_C2")
  , psu =  as.name("PSU")
  , strata = as.name("STRATA")
  , pen = exp(seq(-5,5,0.5))
  , pen.type="alf"
)

get_plot_latent_mean(fit)
```

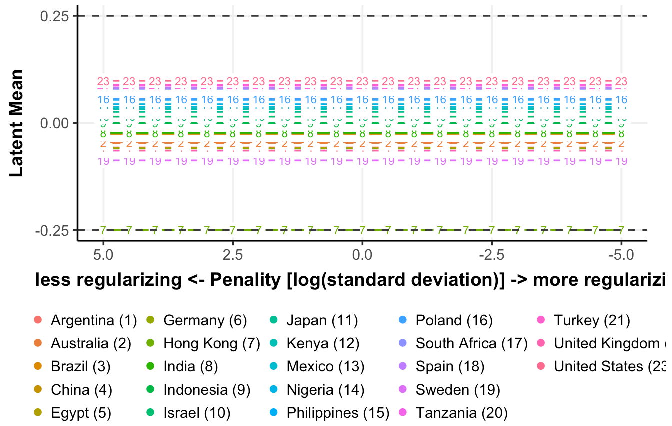


```
get_plot_discrimination(fit)
```

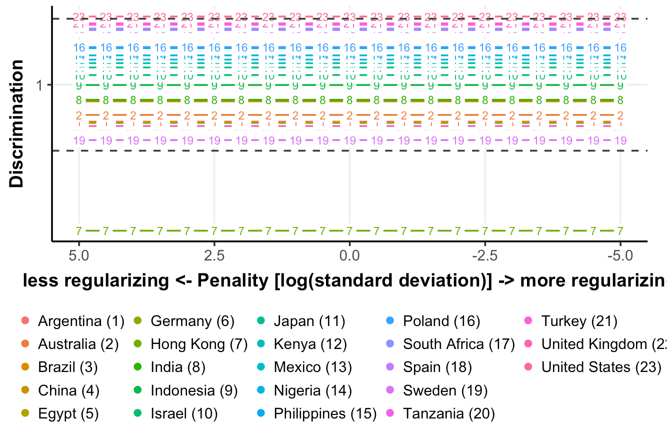


```
plot_iccs(fit, "icc")
```

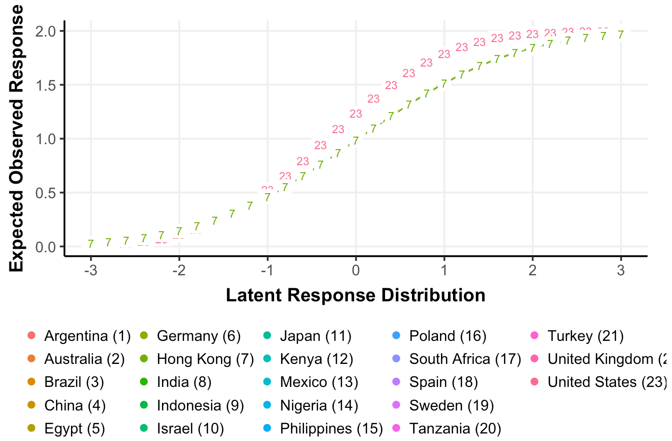


```
format_ft_hetop(fit, 2, cur.var)
```

Say in government (SAY_IN_GOVT_Y2): Threshold Table.

threshold	est.unstd	se.unstd	est.unstd.lb	est.unstd.ub
c[1]	-0.75			
c[2]	0.30			

```
format_ft_hetop(fit, 1, cur.var)
```

Say in government (SAY_IN_GOVT_Y2): HETOP summary table when penality is nu=0.

COUNTRY	mu.std	lambda.std	mu.unstd	mu.unstd.se	mu.unstd.lb	mu.unstd.ub	log.lambda.unstd	log.lambda.unstd.se	log.lambda.unstd.lb	log.lambda.unstd.ub	mu.nonzero	log.lambda.nonzero
Sweden			-0.09				-0.09					
United Kingdom			-0.06				-0.06					
Germany			-0.06				-0.06					
China			-0.06				-0.06					
Argentina			-0.06				-0.06					
Australia			-0.05				-0.05					
Brazil			-0.03				-0.03					
Egypt			-0.02				-0.02					

Note. *significant mu/lambda identified from whether the 95% CI for the parameter contains 0.0. Due to rounding, sometimes the estimate may be '-0.00' [slightly below zero] or '0.00' [slightly above zero]. Standard errors estimated using the inverse of the observed information matrix and can sometimes be singular leading to no estimated standard error for some parameters.

Say in government (SAY_IN_GOVT_Y2): HETOP summary table when penality is nu=0.

COUNTRY	mu.std	lambda.std	mu.unstd	mu.unstd.se	mu.unstd.lb	mu.unstd.ub	log.lambda.unstd	log.lambda.unstd.se	log.lambda.unstd.lb	log.lambda.unstd.ub	mu.nonzero	log.lambda.nonzero
India			-0.02				-0.02					
Indonesia			-0.00				-0.00					
Israel			0.01				0.01					
Japan			0.03				0.03					
Kenya			0.03				0.03					
Mexico			0.04				0.04					
Nigeria			0.04				0.04					
Philippines			0.05				0.05					
Poland			0.06				0.06					
South Africa			0.08				0.08					
Spain			0.08				0.08					
Tanzania			0.09				0.09					
Turkey			0.09				0.09					
United States			0.10				0.10					
Hong Kong			-0.25				-0.25					

Note. *significant mu/lambda identified from whether the 95% CI for the parameter contains 0.0. Due to rounding, sometimes the estimate may be '-0.00' [slightly below zero] or '0.00' [slightly above zero]. Standard errors estimated using the inverse of the observed information matrix and can sometimes be singular leading to no estimated standard error for some parameters.

4.7 Belonging in country

```
i <- i + 1
cur.var <- OUTCOME.LIST[i]
fit <- hetop_pml(
  data = df.raw |> filter(!is.na(ANNUAL_WEIGHT_C2))
  , var = cur.var
  , group = as.name("COUNTRY")
  , wgt = as.name("ANNUAL_WEIGHT_C2")
  , psu =  as.name("PSU")
  , strata = as.name("STRATA")
  , pen = exp(seq(-5,5,0.5))
  , pen.type="alf"
)

get_plot_latent_mean(fit)
```

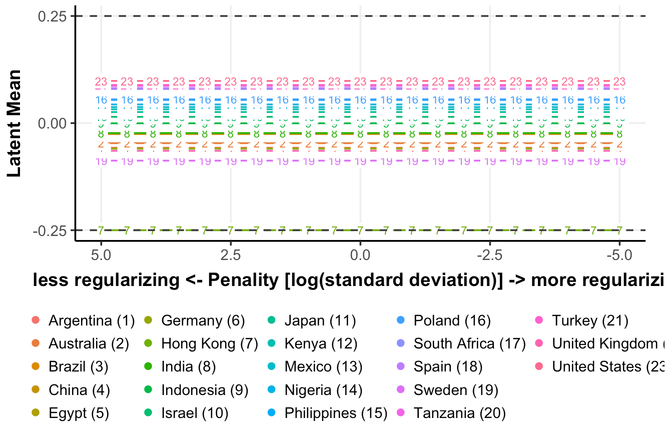


```
get_plot_discrimination(fit)
```

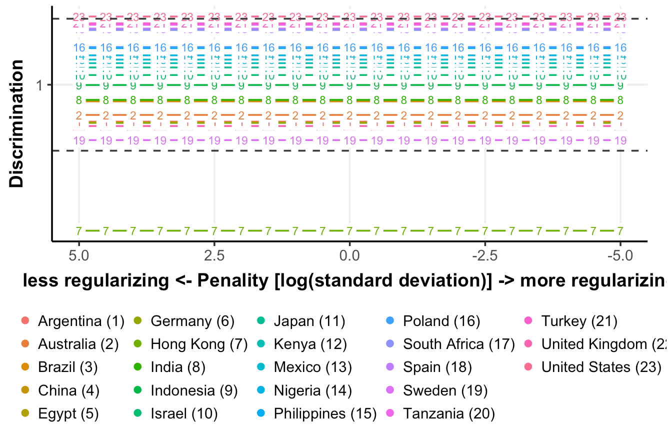


```r
plot_iccs(fit, "icc")
```

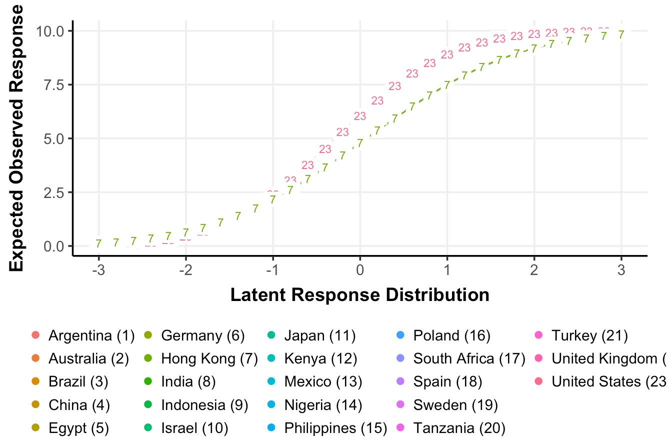


```r
format_ft_hetop(fit, 2, cur.var)
```

Belonging in country (BELONGING_Y2): Threshold Table.

threshold	est.unstd	se.unstd	est.unstd.lb	est.unstd.ub
c[1]	-0.97			
c[2]	-0.75			
c[3]	-0.47			
c[4]	-0.32			
c[5]	-0.24			
c[6]	-0.23			
c[7]	-0.04			
c[8]	0.20			
c[9]	0.30			
c[10]	0.74			

```r
format_ft_hetop(fit, 1, cur.var)
```

Belonging in country (BELONGING_Y2): HETOP summary table when penality is nu=0.

COUNTRY	mu.std	lambda.std	mu.unstd	mu.unstd.se	mu.unstd.lb	mu.unstd.ub	log.lambda.unstd	log.lambda.unstd.se	log.lambda.unstd.lb	log.lambda.unstd.ub	mu.nonzero	log.lambda.nonzero
Sweden			-0.09				-0.09					

Note. *significant mu/lambda identified from whether the 95% CI for the parameter contains 0.0. Due to rounding, sometimes the estimate may be '-0.00' [slightly below zero] or '0.00' [slightly above zero]. Standard errors estimated using the inverse of the observed information matrix and can sometimes be singular leading to no estimated standard error for some parameters.

Belonging in country (BELONGING_Y2): HETOP summary table when penality is nu=0.

COUNTRY	mu.std	lambda.std	mu.unstd	mu.unstd.se	mu.unstd.lb	mu.unstd.ub	log.lambda.unstd	log.lambda.unstd.se	log.lambda.unstd.lb	log.lambda.unstd.ub	mu.nonzero	log.lambda.nonzero
United Kingdom			-0.06				-0.06					
Germany			-0.06				-0.06					
China			-0.06				-0.06					
Argentina			-0.06				-0.06					
Australia			-0.05				-0.05					
Brazil			-0.03				-0.03					
Egypt			-0.02				-0.02					
India			-0.02				-0.02					
Indonesia			-0.00				-0.00					
Israel			0.01				0.01					
Japan			0.03				0.03					
Kenya			0.03				0.03					
Mexico			0.04				0.04					
Nigeria			0.04				0.04					
Philippines			0.05				0.05					
Poland			0.06				0.06					
South Africa			0.08				0.08					
Spain			0.08				0.08					
Tanzania			0.09				0.09					
Turkey			0.09				0.09					
United States			0.10				0.10					
Hong Kong			-0.25				-0.25					

Note. *significant mu/lambda identified from whether the 95% CI for the parameter contains 0.0. Due to rounding, sometimes the estimate may be '-0.00' [slightly below zero] or '0.00' [slightly above zero]. Standard errors estimated using the inverse of the observed information matrix and can sometimes be singular leading to no estimated standard error for some parameters.

4.8 City/place satisfaction

```
i <- i + 1
cur.var <- OUTCOME.LIST[i]
fit <- hetop_pml(
  data = df.raw |> filter(!is.na(ANNUAL_WEIGHT_C2))
  , var = cur.var
  , group = as.name("COUNTRY")
  , wgt = as.name("ANNUAL_WEIGHT_C2")
  , psu =  as.name("PSU")
  , strata = as.name("STRATA")
  , pen = exp(seq(-5,5,0.5))
  , pen.type="alf"
)

get_plot_latent_mean(fit)
```

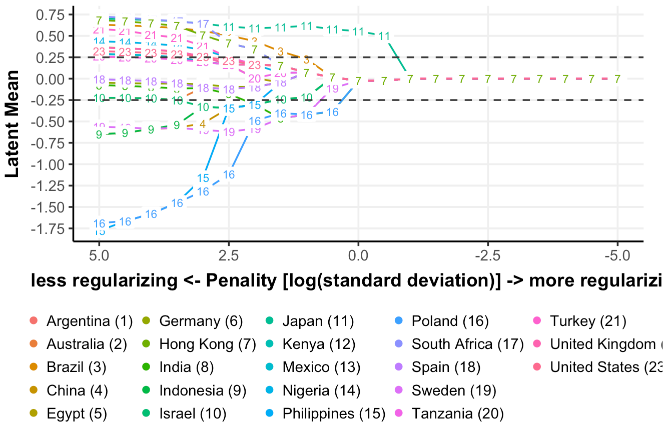


```
get_plot_discrimination(fit)
```

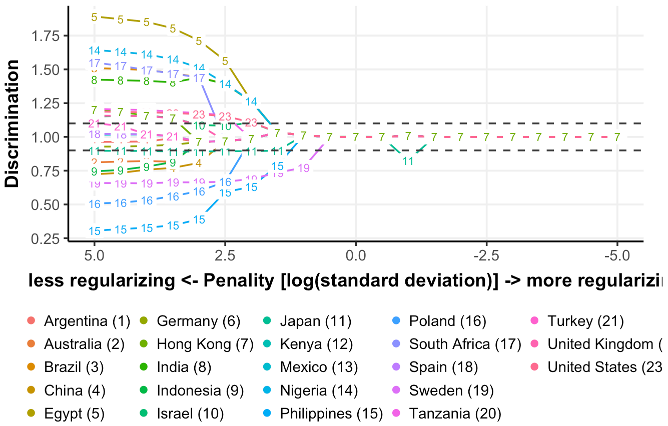


```
plot_iccs(fit, "icc")
```

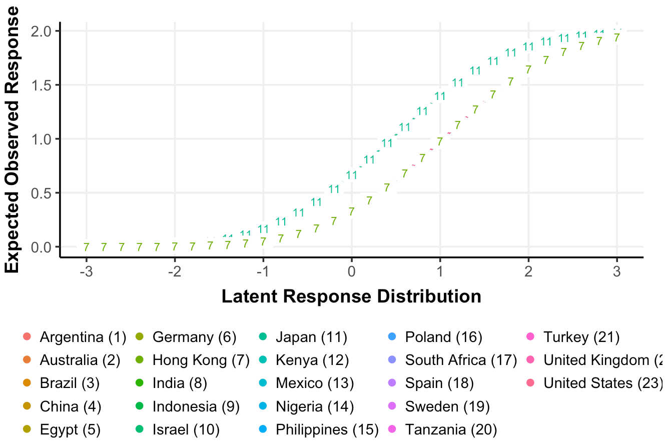


```
format_ft_hetop(fit, 2, cur.var)
```

City/place satisfaction (SAT_LIVE_Y2): Threshold Table.

threshold	est.unstd	se.unstd	est.unstd.lb	est.unstd.ub
c[1]	0.69	0.03	0.63	0.74
c[2]	1.32	0.04	1.24	1.40

```
format_ft_hetop(fit, 1, cur.var)
```

City/place satisfaction (SAT_LIVE_Y2): HETOP summary table when penality is nu=0.												
**COUNTRY**	**mu.std**	**lambda.std**	**mu.unstd**	**mu.unstd.se**	**mu.unstd.lb**	**mu.unstd.ub**	**log.lambda.unstd**	**log.lambda.unstd.se**	**log.lambda.unstd.lb**	**log.lambda.unstd.ub**	**mu.nonzero**	**log.lambda.nonzero**
Japan	0.55	1.00	0.55	0.07	0.41	0.70	-0.00	0.14	-0.28	0.27	*	
United States	-0.02	1.00	-0.02	0.20	-0.42	0.37	-0.00	0.19	-0.38	0.38		
Brazil	-0.02	1.00	-0.02				-0.00					
Kenya	-0.02	1.00	-0.02	0.59	-1.17	1.12	-0.00	0.53	-1.05	1.05		
United Kingdom	-0.02	1.00	-0.03	0.64	-1.28	1.23	-0.00	0.56	-1.10	1.10		
South Africa	-0.02	1.00	-0.03	0.90	-1.79	1.74	-0.00	0.62	-1.22	1.22		
Argentina	-0.02	1.00	-0.03	0.26	-0.54	0.49	-0.00	0.22	-0.43	0.43		
Hong Kong	-0.02	1.00	-0.03	0.28	-0.57	0.52	-0.00	0.03	-0.06	0.06		
Mexico	-0.02	1.00	-0.03	0.25	-0.51	0.46	-0.00	0.16	-0.30	0.30		
Turkey	-0.02	1.00	-0.03	0.93	-1.85	1.80	-0.00	0.64	-1.26	1.26		
Tanzania	-0.02	1.00	-0.03	0.10	-0.21	0.16	0.00	0.05	-0.11	0.11		
Philippines	-0.02	1.00	-0.03	0.21	-0.44	0.39	-0.00	0.21	-0.42	0.41		
Nigeria	-0.02	1.00	-0.03	0.60	-1.20	1.15	0.00	0.66	-1.29	1.29		
Australia	-0.03	1.00	-0.03	0.28	-0.58	0.53	0.00	0.34	-0.66	0.66		
Spain	-0.03	1.00	-0.03	0.17	-0.36	0.31	0.00	0.25	-0.50	0.50		
Germany	-0.03	1.00	-0.03	0.08	-0.19	0.14	0.00					
Egypt	-0.03	1.00	-0.03	0.16	-0.34	0.29	0.00	0.25	-0.48	0.48		
Indonesia	-0.03	1.00	-0.03	0.23	-0.47	0.42	0.00	0.49	-0.96	0.96		
Israel	-0.03	1.00	-0.03	0.12	-0.26	0.21	0.00	0.18	-0.34	0.34		
China	-0.03	1.00	-0.03	0.10	-0.22	0.17	0.00	0.11	-0.22	0.22		
Poland	-0.03	1.00	-0.03	0.09	-0.20	0.15	0.00	0.12	-0.23	0.23		
India	-0.03	1.00	-0.03	0.10	-0.23	0.17	0.00	0.18	-0.36	0.36		
Sweden	-0.03	1.00	-0.03	0.07	-0.17	0.12	0.00	0.09	-0.18	0.18		

Note. *significant mu/lambda identified from whether the 95% CI for the parameter contains 0.0. Due to rounding, sometimes the estimate may be '-0.00' [slightly below zero] or '0.00' [slightly above zero]. Standard errors estimated using the inverse of the observed information matrix and can sometimes be singular leading to no estimated standard error for some parameters.

4.9 Trust within country

```
i <- i + 1
cur.var <- OUTCOME.LIST[i]
fit <- hetop_pml(
  data = df.raw |> filter(!is.na(ANNUAL_WEIGHT_C2))
  , var = cur.var
  , group = as.name("COUNTRY")
  , wgt = as.name("ANNUAL_WEIGHT_C2")
  , psu =  as.name("PSU")
  , strata = as.name("STRATA")
  , pen = exp(seq(-5,5,0.5))
  , pen.type="alf"
)

get_plot_latent_mean(fit)
```

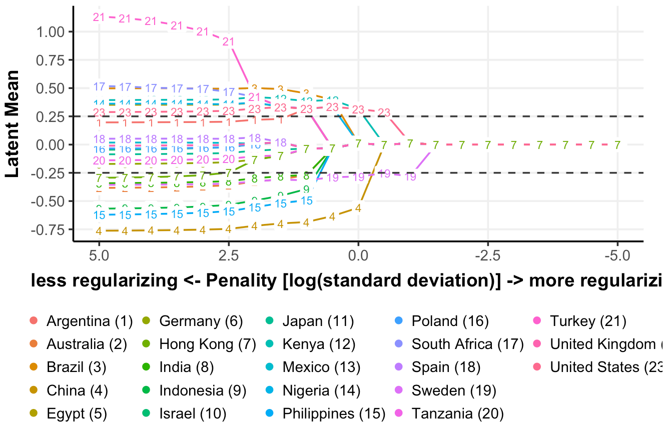


```
get_plot_discrimination(fit)
```

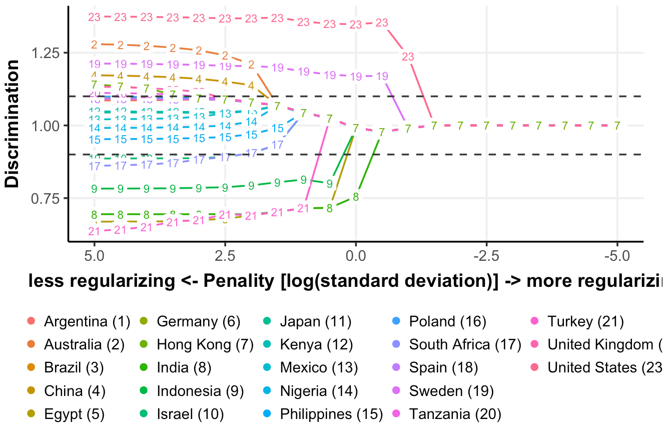


```
plot_iccs(fit, "icc")
```

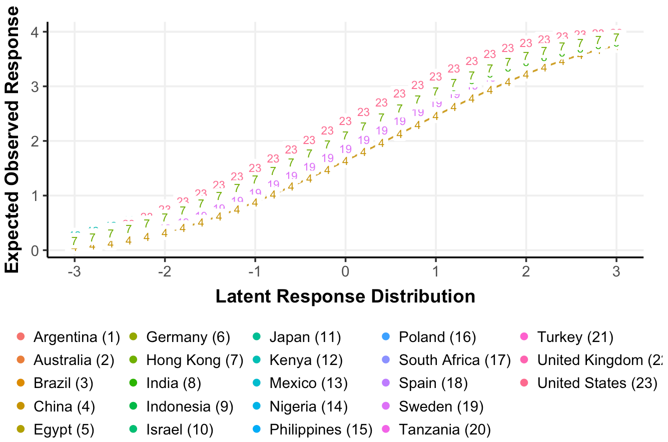


```
format_ft_hetop(fit, 2, cur.var)
```

Trust within country (TRUST_PEOPLE_Y2): Threshold Table.

threshold	est.unstd	se.unstd	est.unstd.lb	est.unstd.ub
c[1]	-2.00	0.06	-2.12	-1.88
c[2]	-0.66	0.04	-0.74	-0.59

Trust within country (TRUST_PEOPLE_Y2): Threshold Table.				
threshold	est.unstd	se.unstd	est.unstd.lb	est.unstd.ub
c[3]	0.44	0.03	0.38	0.50
c[4]	1.69	0.04	1.61	1.77

```
format_ft_hetop(fit, 1, cur.var)
```

Trust within country (TRUST_PEOPLE_Y2): HETOP summary table when penality is nu=0.

COUNTRY	mu.std	lambda.std	mu.unstd	mu.unstd.se	mu.unstd.lb	mu.unstd.ub	log.lambda.unstd	log.lambda.unstd.se	log.lambda.unstd.lb	log.lambda.unstd.ub	mu.nonzero	log.lambda.nonzero
Kenya	0.31	0.99	0.32	0.06	0.19	0.44	-0.01	0.05	-0.11	0.09	*	
United States	0.31	1.34	0.32	0.04	0.24	0.39	0.30	0.03	0.24	0.36	*	*
Brazil	0.01	0.99	0.01	0.11	-0.20	0.22	-0.01	0.08	-0.16	0.15		
Nigeria	0.01	0.99	0.01	0.11	-0.20	0.22	-0.01	0.08	-0.16	0.14		
Egypt	0.01	0.99	0.01	0.10	-0.19	0.21	-0.01	0.06	-0.13	0.11		
Argentina	0.01	0.99	0.01	0.10	-0.19	0.21	-0.01	0.08	-0.17	0.15		
South Africa	0.01	0.99	0.01	0.20	-0.38	0.41	-0.01	0.13	-0.27	0.26		
Turkey	0.01	0.99	0.01	0.50	-0.96	0.98	-0.01	0.26	-0.53	0.51		
Spain	0.01	0.99	0.01	0.10	-0.18	0.20	-0.01	0.08	-0.16	0.14		
Mexico	0.01	0.99	0.01	0.11	-0.20	0.22	-0.01	0.08	-0.17	0.16		
United Kingdom	0.01	0.99	0.01	0.09	-0.16	0.18	-0.01	0.07	-0.15	0.13		
Poland	0.01	0.99	0.01	0.07	-0.12	0.15	-0.01	0.05	-0.11	0.10		
Israel	0.01	0.99	0.01	0.10	-0.19	0.22	-0.01	0.07	-0.16	0.14		
Hong Kong	0.01	0.99	0.01	0.20	-0.38	0.40	-0.01	0.17	-0.34	0.32		
Tanzania	0.01	0.99	0.01	0.07	-0.13	0.15	-0.01	0.06	-0.12	0.11		
Japan	0.01	0.99	0.01	0.05	-0.09	0.11	-0.01	0.04	-0.08	0.07		
Germany	0.01	0.99	0.01	0.07	-0.13	0.16	-0.01	0.06	-0.12	0.11		
Australia	0.01	0.99	0.01	0.12	-0.22	0.24	-0.01	0.10	-0.21	0.19		
India	0.01	0.76	0.01	0.09	-0.16	0.19	-0.28	0.05	-0.39	-0.17		*
Indonesia	0.01	0.99	0.01	0.12	-0.22	0.24	-0.01	0.07	-0.15	0.14		
Philippines	0.01	0.99	0.01	0.13	-0.25	0.27	-0.01	0.09	-0.19	0.17		
Sweden	-0.28	1.17	-0.28	0.05	-0.38	-0.18	0.16	0.04	0.07	0.24	*	*
China	-0.55	0.99	-0.56	0.08	-0.72	-0.40	-0.01	0.07	-0.14	0.12	*	

Note. *significant mu/lambda identified from whether the 95% CI for the parameter contains 0.0. Due to rounding, sometimes the estimate may be '-0.00' [slightly below zero] or '0.00' [slightly above zero]. Standard errors estimated using the inverse of the observed information matrix and can sometimes be singular leading to no estimated standard error for some parameters.

5 Social Participation

5.1 Community participation

```
i <- i + 1
cur.var <- OUTCOME.LIST[i]
fit <- hetop_pml(
  data = df.raw |> filter(!is.na(ANNUAL_WEIGHT_C2))
  , var = cur.var
  , group = as.name("COUNTRY")
  , wgt = as.name("ANNUAL_WEIGHT_C2")
  , psu =  as.name("PSU")
  , strata = as.name("STRATA")
```

```r
  , pen = exp(seq(-5,5,0.5))
  , pen.type="alf"
)

get_plot_latent_mean(fit)
```

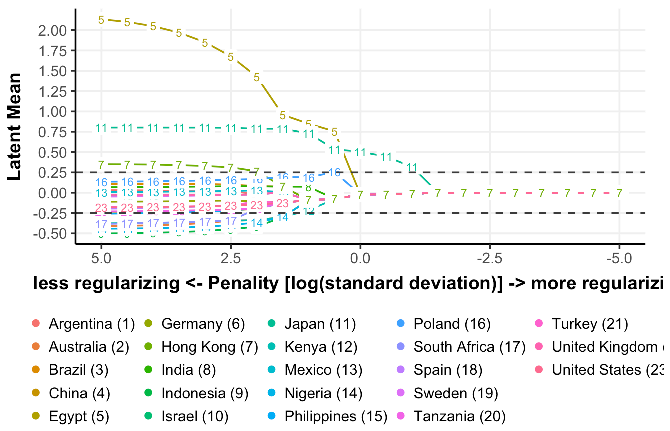


```r
get_plot_discrimination(fit)
```

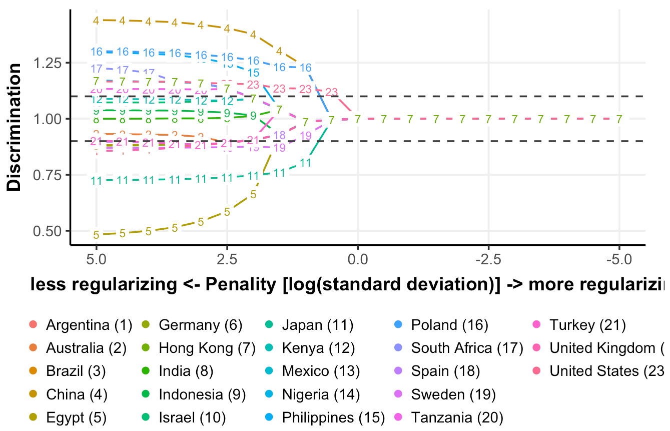


```r
plot_iccs(fit, "icc")
```

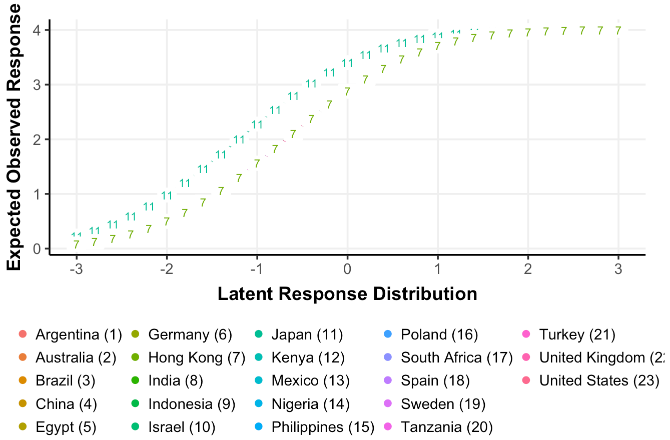


```r
format_ft_hetop(fit, 2, cur.var)
```

Weekly+ community participation (GROUP_NOT_REL_Y2): Threshold Table.				
threshold	est.unstd	se.unstd	est.unstd.lb	est.unstd.ub
c[1]	-1.43	0.05	-1.53	-1.33
c[2]	-0.93	0.04	-1.00	-0.86
c[3]	-0.52	0.03	-0.57	-0.47
c[4]	0.09	0.02	0.05	0.13

```
format_ft_hetop(fit, 1, cur.var)
```

Weekly+ community participation (GROUP_NOT_REL_Y2): HETOP summary table when penality is nu=0.

COUNTRY	mu.std	lambda.std	mu.unstd	mu.unstd.se	mu.unstd.lb	mu.unstd.ub	log.lambda.unstd	log.lambda.unstd.se	log.lambda.unstd.lb	log.lambda.unstd.ub	mu.nonzero	log.lambda.nonzero
Japan	0.50	1.00	0.50	0.06	0.38	0.63	-0.00	0.06	-0.11	0.11	*	
Poland	-0.02	1.00	-0.02	0.07	-0.15	0.11	0.00	0.10	-0.19	0.19		
Egypt	-0.02	1.00	-0.02				-0.00					
India	-0.02	1.00	-0.02	0.07	-0.15	0.11	0.00	0.09	-0.17	0.17		
Brazil	-0.02	1.00	-0.02	0.08	-0.18	0.13	-0.00	0.09	-0.18	0.18		
Mexico	-0.02	1.00	-0.02	0.11	-0.24	0.19	0.00	0.13	-0.25	0.25		
Hong Kong	-0.02	1.00	-0.02	0.29	-0.59	0.54	0.00	0.54	-1.06	1.06		
Israel	-0.02	1.00	-0.02	0.10	-0.23	0.18	0.00	0.11	-0.22	0.22		
Argentina	-0.02	1.00	-0.02	0.10	-0.21	0.17	-0.00	0.11	-0.21	0.21		
United Kingdom	-0.02	1.00	-0.02	0.09	-0.21	0.16	-0.00	0.09	-0.17	0.17		
Sweden	-0.02	1.00	-0.02	0.05	-0.13	0.08	-0.00	0.06	-0.11	0.11		
Turkey	-0.02	1.00	-0.02	0.27	-0.55	0.50	-0.00	0.27	-0.52	0.52		
South Africa	-0.02	1.00	-0.02	0.10	-0.22	0.17	0.00					
Philippines	-0.02	1.00	-0.02	0.10	-0.22	0.17	0.00					
China	-0.02	1.00	-0.02	0.09	-0.19	0.15	0.00	0.10	-0.19	0.19		
Germany	-0.02	1.00	-0.02	0.07	-0.17	0.12	-0.00	0.08	-0.16	0.16		
Spain	-0.02	1.00	-0.02	0.11	-0.24	0.19	-0.00	0.09	-0.19	0.19		
Tanzania	-0.02	1.00	-0.02	0.08	-0.18	0.14	0.00	0.08	-0.16	0.16		
Australia	-0.02	1.00	-0.02	0.14	-0.30	0.25	-0.00	0.11	-0.21	0.21		
Nigeria	-0.02	1.00	-0.02	0.13	-0.29	0.24	-0.00	0.12	-0.23	0.23		
United States	-0.02	1.00	-0.02	0.04	-0.09	0.05	0.00	0.05	-0.09	0.09		
Indonesia	-0.02	1.00	-0.02	0.15	-0.31	0.27	-0.00	0.11	-0.21	0.21		
Kenya	-0.02	1.00	-0.02	0.08	-0.17	0.13	-0.00	0.08	-0.17	0.17		

Note. *significant mu/lambda identified from whether the 95% CI for the parameter contains 0.0. Due to rounding, sometimes the estimate may be '-0.00' [slightly below zero] or '0.00' [slightly above zero]. Standard errors estimated using the inverse of the observed information matrix and can sometimes be singular leading to no estimated standard error for some parameters.

5.2 Religious attendance

```
i <- i + 1
cur.var <- OUTCOME.LIST[i]
fit <- hetop_pml(
  data = df.raw |> filter(!is.na(ANNUAL_WEIGHT_C2))
  , var = cur.var
  , group = as.name("COUNTRY")
  , wgt = as.name("ANNUAL_WEIGHT_C2")
  , psu =  as.name("PSU")
  , strata = as.name("STRATA")
```

```
  , pen = exp(seq(-5,5,0.5))
  , pen.type="alf"
)

get_plot_latent_mean(fit)
```

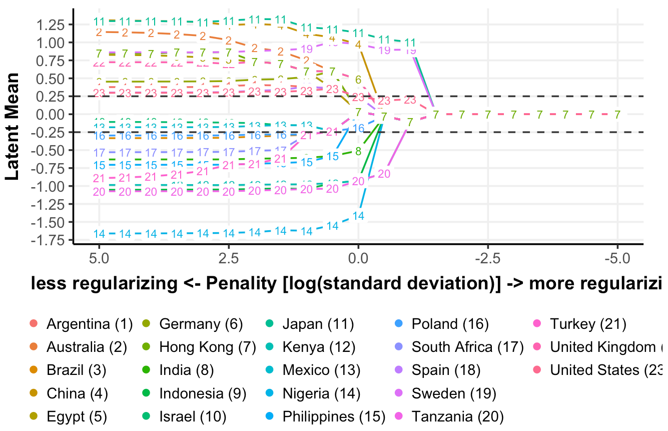


```
get_plot_discrimination(fit)
```

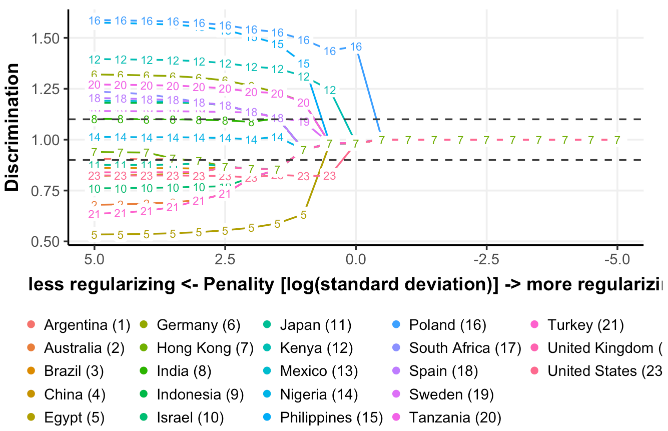


```
plot_iccs(fit, "icc")
```

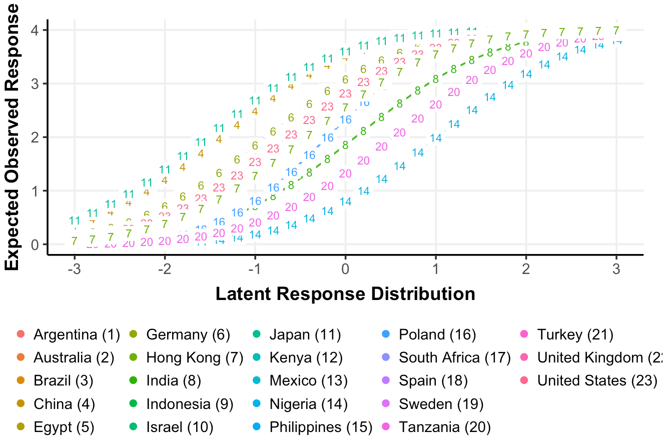


```
format_ft_hetop(fit, 2, cur.var)
```

Weekly+ religious attendance (ATTEND_SVCS_Y2): Threshold Table.				
threshold	est.unstd	se.unstd	est.unstd.lb	est.unstd.ub
c[1]	-1.30			
c[2]	-0.52			
c[3]	-0.22			
c[4]	0.42	0.03	0.35	0.48

```
format_ft_hetop(fit, 1, cur.var)
```

Weekly+ religious attendance (ATTEND_SVCS_Y2): HETOP summary table when penality is nu=0.

COUNTRY	mu.std	lambda.std	mu.unstd	mu.unstd.se	mu.unstd.lb	mu.unstd.ub	log.lambda.unstd	log.lambda.unstd.se	log.lambda.unstd.lb	log.lambda.unstd.ub	mu.nonzero	log.lambda.nonzero
Japan	0.96	0.99	1.14	0.09	0.95	1.32	-0.02	0.07	-0.15	0.12	*	
China	0.83	0.99	0.98	0.12	0.75	1.21	-0.02	0.11	-0.23	0.19	*	
Sweden	0.83	0.99	0.98	0.09	0.80	1.16	-0.02	0.08	-0.17	0.13	*	
Germany	0.41	0.99	0.49	0.08	0.33	0.64	-0.02	0.09	-0.20	0.17	*	
United Kingdom	0.41	0.99	0.49	0.10	0.30	0.67	-0.02	0.10	-0.21	0.17	*	
United States	0.21	0.98	0.25	0.04	0.17	0.32	-0.02	0.03	-0.07	0.03	*	
Spain	0.21	0.99	0.25	0.09	0.06	0.43	-0.02	0.11	-0.24	0.21	*	
Australia	0.03	0.99	0.03	0.13	-0.22	0.29	-0.02	0.19	-0.39	0.36		
Egypt	0.03	0.99	0.03	0.09	-0.14	0.21	-0.02	0.09	-0.19	0.16		
Argentina	0.03	0.99	0.03	0.09	-0.15	0.21	-0.02	0.11	-0.22	0.19		
Hong Kong	0.03	0.99	0.03				-0.02					
Israel	0.03	0.99	0.03	0.11	-0.18	0.25	-0.02	0.09	-0.20	0.16		
Mexico	0.03	0.99	0.03	0.11	-0.19	0.26	-0.02	0.11	-0.24	0.21		
Turkey	0.03	0.99	0.03	0.50	-0.94	1.00	-0.02	0.31	-0.63	0.60		
South Africa	0.03	0.99	0.03	0.30	-0.55	0.62	-0.02	0.25	-0.50	0.47		
Brazil	0.03	0.99	0.03	0.10	-0.16	0.23	-0.02	0.08	-0.17	0.13		
Philippines	0.03	0.99	0.03				-0.02					
Poland	-0.16	1.38	-0.19	0.03	-0.26	-0.12	0.38	0.06	0.27	0.49	*	*
India	-0.43	0.99	-0.51	0.05	-0.60	-0.41	-0.02	0.06	-0.14	0.11	*	
Indonesia	-0.79	0.99	-0.93	0.08	-1.08	-0.77	-0.02	0.11	-0.23	0.19	*	
Kenya	-0.79	0.99	-0.93	0.02	-0.96	-0.89	-0.02	0.07	-0.15	0.12	*	
Tanzania	-0.79	0.99	-0.93	0.04	-1.01	-0.84	-0.02	0.08	-0.17	0.13	*	
Nigeria	-1.20	0.99	-1.41	0.08	-1.56	-1.26	-0.02	0.11	-0.24	0.20	*	

Note. *significant mu/lambda identified from whether the 95% CI for the parameter contains 0.0. Due to rounding, sometimes the estimate may be '-0.00' [slightly below zero] or '0.00' [slightly above zero]. Standard errors estimated using the inverse of the observed information matrix and can sometimes be singular leading to no estimated standard error for some parameters.

6 Social Distress

6.1 Loneliness

```
i <- i + 1
cur.var <- OUTCOME.LIST[i]
fit <- hetop_pml(
  data = df.raw |> filter(!is.na(ANNUAL_WEIGHT_C2))
  , var = cur.var
  , group = as.name("COUNTRY")
```

```
  , wgt = as.name("ANNUAL_WEIGHT_C2")
  , psu =  as.name("PSU")
  , strata = as.name("STRATA")
  , pen = exp(seq(-5,5,0.5))
  , pen.type="alf"
)

get_plot_latent_mean(fit)
```

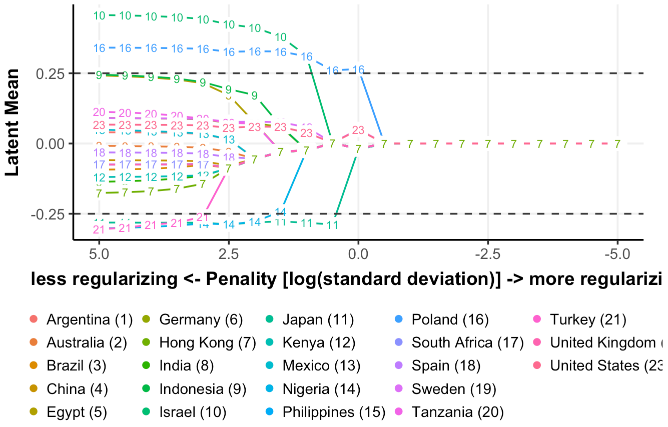


```
get_plot_discrimination(fit)
```

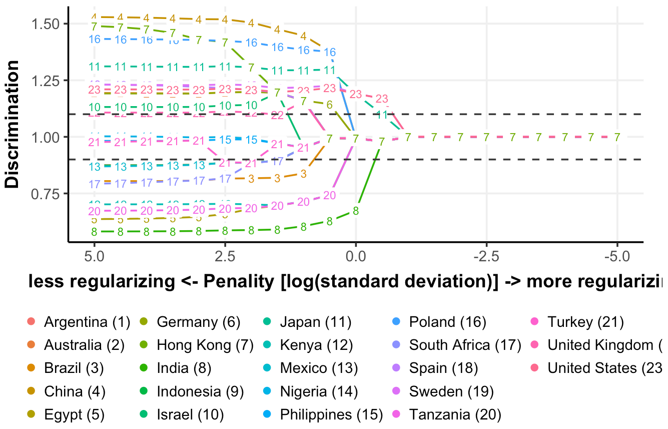


```
plot_iccs(fit, "icc")
```

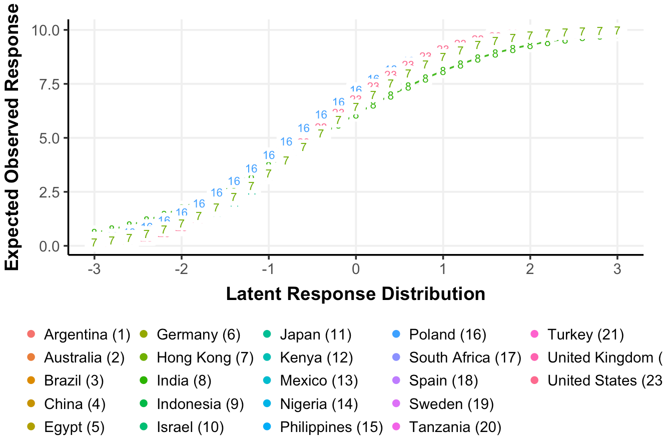


```
format_ft_hetop(fit, 2, cur.var)
```

Loneliness (LONELY_Y2): Threshold Table.				
**threshold**	**est.unstd**	**se.unstd**	**est.unstd.lb**	**est.unstd.ub**
c[1]	-1.46	0.04	-1.54	-1.39
c[2]	-1.33	0.03	-1.39	-1.26
c[3]	-1.12	0.03	-1.18	-1.06
c[4]	-0.88	0.03	-0.94	-0.83
c[5]	-0.68	0.03	-0.73	-0.63
c[6]	-0.37	0.02	-0.41	-0.32
c[7]	-0.18	0.02	-0.22	-0.13
c[8]	0.08	0.02	0.03	0.13
c[9]	0.43	0.03	0.38	0.49
c[10]	0.77	0.03	0.71	0.83

```
format_ft_hetop(fit, 1, cur.var)
```

Loneliness (LONELY_Y2): HETOP summary table when penality is nu=0.												
**COUNTRY**	**mu.std**	**lambda.std**	**mu.unstd**	**mu.unstd.se**	**mu.unstd.lb**	**mu.unstd.ub**	**log.lambda.unstd**	**log.lambda.unstd.se**	**log.lambda.unstd.lb**	**log.lambda.unstd.ub**	**mu.nonzero**	**log.lambda.nonzero**
Poland	0.26	0.99	0.27	0.06	0.14	0.39	-0.01	0.07	-0.15	0.14	*	
Sweden	0.05	1.19	0.05	0.04	-0.03	0.14	0.17	0.04	0.09	0.26		*
United States	0.05	1.19	0.05	0.03	-0.01	0.11	0.17	0.03	0.12	0.23		*
Israel	-0.02	0.99	-0.02	0.14	-0.29	0.25	-0.01	0.14	-0.27	0.26		
Germany	-0.02	0.99	-0.02	0.07	-0.15	0.12	-0.01	0.07	-0.14	0.12		
Indonesia	-0.02	0.99	-0.02	0.10	-0.21	0.18	-0.01	0.09	-0.18	0.16		
Egypt	-0.02	0.99	-0.02	0.09	-0.20	0.16	-0.01	0.07	-0.14	0.13		
Tanzania	-0.02	0.99	-0.02	0.07	-0.15	0.12	-0.01	0.05	-0.11	0.10		
Argentina	-0.02	0.99	-0.02	0.09	-0.20	0.16	-0.01	0.08	-0.16	0.15		
Mexico	-0.02	0.99	-0.02	0.10	-0.22	0.18	-0.01	0.09	-0.18	0.16		
Australia	-0.02	0.99	-0.02	0.10	-0.21	0.17	-0.01	0.09	-0.19	0.17		
Spain	-0.02	0.99	-0.02	0.09	-0.20	0.16	-0.01	0.09	-0.18	0.17		
China	-0.02	0.99	-0.02	0.07	-0.16	0.13	-0.01	0.08	-0.17	0.16		
South Africa	-0.02	0.99	-0.02	0.16	-0.33	0.29	-0.01	0.13	-0.25	0.24		
Hong Kong	-0.02	0.99	-0.02	0.19	-0.39	0.35	-0.01	0.21	-0.41	0.40		
United Kingdom	-0.02	0.99	-0.02	0.08	-0.18	0.14	-0.01	0.08	-0.16	0.14		
Philippines	-0.02	0.99	-0.02	0.10	-0.21	0.17	-0.01	0.09	-0.17	0.16		
Turkey	-0.02	0.99	-0.02	0.25	-0.50	0.46	-0.01	0.20	-0.40	0.39		
India	-0.02	0.68	-0.02	0.10	-0.21	0.17	-0.39	0.06	-0.52	-0.27		*
Brazil	-0.02	0.99	-0.02	0.08	-0.17	0.14	-0.01	0.06	-0.13	0.12		
Nigeria	-0.02	0.99	-0.02	0.10	-0.21	0.18	-0.01	0.08	-0.17	0.16		
Kenya	-0.02	0.99	-0.02	0.06	-0.14	0.10	-0.01	0.05	-0.10	0.08		
Japan	-0.02	1.19	-0.02	0.05	-0.11	0.07	0.17	0.04	0.09	0.26		*

Note. *significant mu/lambda identified from whether the 95% CI for the parameter contains 0.0. Due to rounding, sometimes the estimate may be '-0.00' [slightly below zero] or '0.00' [slightly above zero]. Standard errors estimated using the inverse of the observed information matrix and can sometimes be singular leading to no estimated standard error for some parameters.

6.2 Perceived discrimination

```
i <- i + 1
cur.var <- OUTCOME.LIST[i]
fit <- hetop_pml(
  data = df.raw |> filter(!is.na(ANNUAL_WEIGHT_C2))
  , var = cur.var
  , group = as.name("COUNTRY")
  , wgt = as.name("ANNUAL_WEIGHT_C2")
  , psu =  as.name("PSU")
  , strata = as.name("STRATA")
  , pen = exp(seq(-5,5,0.5))
  , pen.type="alf"
)

get_plot_latent_mean(fit)
```

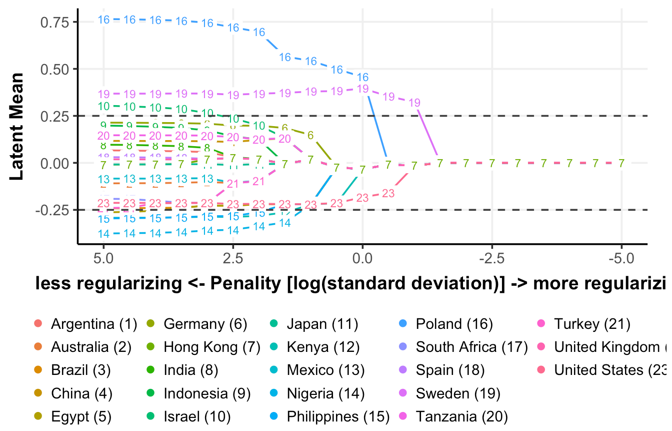


```
get_plot_discrimination(fit)
```

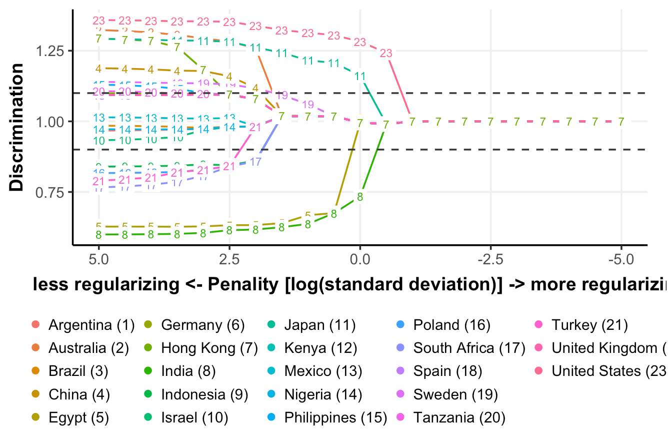


```
plot_iccs(fit, "icc")
```

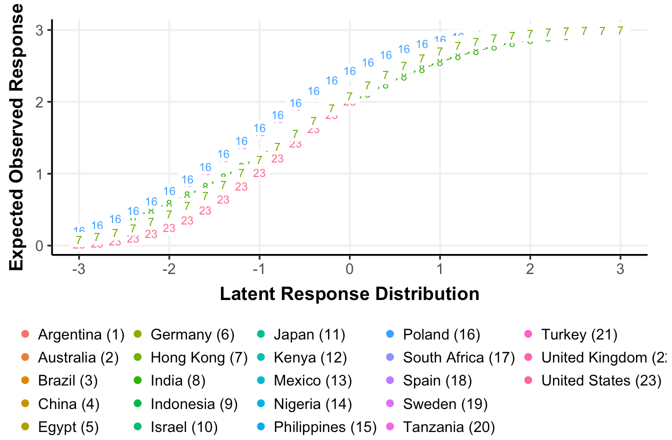

```r
format_ft_hetop(fit, 2, cur.var)
```

Perceived discrimination (DISCRIMINATED_Y2): Threshold Table.				
threshold	est.unstd	se.unstd	est.unstd.lb	est.unstd.ub
c[1]	-1.56	0.04	-1.63	-1.48
c[2]	-0.78	0.03	-0.83	-0.73
c[3]	0.29	0.03	0.24	0.34

```r
format_ft_hetop(fit, 1, cur.var)
```

Perceived discrimination (DISCRIMINATED_Y2): HETOP summary table when penality is nu=0.

COUNTRY	mu.std	lambda.std	mu.unstd	mu.unstd.se	mu.unstd.lb	mu.unstd.ub	log.lambda.unstd	log.lambda.unstd.se	log.lambda.unstd.lb	log.lambda.unstd.ub	mu.nonzero	log.lambda.nonzero
Poland	0.45	1.00	0.46	0.08	0.31	0.61	-0.00	0.07	-0.15	0.14	*	
Sweden	0.39	1.00	0.40	0.06	0.28	0.52	-0.00	0.06	-0.12	0.12	*	
Germany	-0.03	1.00	-0.03	0.07	-0.18	0.11	-0.00	0.08	-0.16	0.15		
Tanzania	-0.03	1.00	-0.03	0.07	-0.17	0.11	-0.00	0.08	-0.16	0.15		
China	-0.03	1.00	-0.03	0.08	-0.19	0.12	-0.00	0.09	-0.17	0.17		
Japan	-0.03	1.16	-0.03	0.04	-0.12	0.05	0.15	0.05	0.06	0.24		*
Israel	-0.03	1.00	-0.03	0.11	-0.24	0.18	-0.00	0.11	-0.22	0.21		
Indonesia	-0.03	1.00	-0.03	0.10	-0.23	0.17	-0.00	0.09	-0.19	0.18		
United Kingdom	-0.03	1.00	-0.03	0.09	-0.21	0.14	-0.00	0.09	-0.18	0.17		
Spain	-0.03	1.00	-0.03	0.10	-0.22	0.16	-0.00	0.10	-0.20	0.19		
Argentina	-0.03	1.00	-0.03	0.10	-0.22	0.16	-0.00	0.09	-0.19	0.18		
India	-0.03	0.74	-0.03	0.09	-0.21	0.15	-0.31	0.07	-0.45	-0.17		*
Hong Kong	-0.03	1.00	-0.03	0.19	-0.41	0.34	-0.00	0.21	-0.42	0.41		
Australia	-0.03	1.00	-0.03	0.11	-0.24	0.17	-0.00	0.11	-0.22	0.21		
Mexico	-0.03	1.00	-0.03	0.11	-0.26	0.19	-0.00	0.11	-0.21	0.20		
Turkey	-0.03	1.00	-0.03	0.26	-0.55	0.49	-0.00	0.21	-0.41	0.40		
South Africa	-0.03	1.00	-0.03	0.19	-0.40	0.33	-0.00	0.15	-0.29	0.28		
Philippines	-0.03	1.00	-0.03	0.12	-0.26	0.20	-0.00	0.11	-0.21	0.20		
Brazil	-0.03	1.00	-0.03	0.09	-0.21	0.15	-0.00	0.08	-0.16	0.15		
Egypt	-0.03	0.99	-0.03	0.12	-0.27	0.20	-0.01	0.09	-0.17	0.16		
Nigeria	-0.03	1.00	-0.03	0.12	-0.27	0.20	-0.00	0.10	-0.19	0.18		
Kenya	-0.03	1.00	-0.03	0.07	-0.18	0.11	-0.00	0.06	-0.13	0.12		
United States	-0.18	1.28	-0.18	0.03	-0.25	-0.12	0.25	0.03	0.18	0.31	*	*

Note. *significant mu/lambda identified from whether the 95% CI for the parameter contains 0.0. Due to rounding, sometimes the estimate may be '-0.00' [slightly below zero] or '0.00' [slightly above zero]. Standard errors estimated using the inverse of the observed information matrix and can sometimes be singular leading to no estimated standard error for some parameters.

7 Character & Prosocial Behavior

7.1 Orientation to promote good

```r
i <- i + 1
cur.var <- OUTCOME.LIST[i]
fit <- hetop_pml(
  data = df.raw |> filter(!is.na(ANNUAL_WEIGHT_C2))
  , var = cur.var
```

```
  , group = as.name("COUNTRY")
  , wgt = as.name("ANNUAL_WEIGHT_C2")
  , psu =  as.name("PSU")
  , strata = as.name("STRATA")
  , pen = exp(seq(-5,5,0.5))
  , pen.type="alf"
)

get_plot_latent_mean(fit)
```

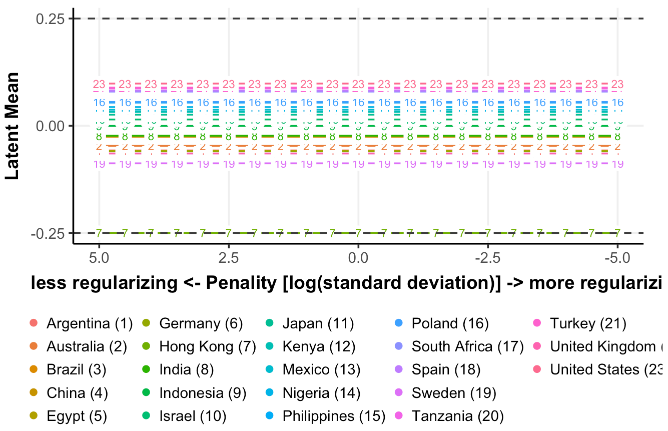


```
get_plot_discrimination(fit)
```

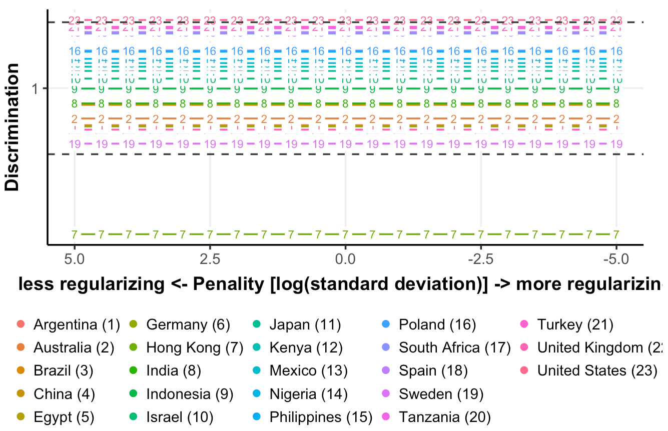


```
plot_iccs(fit, "icc")
```

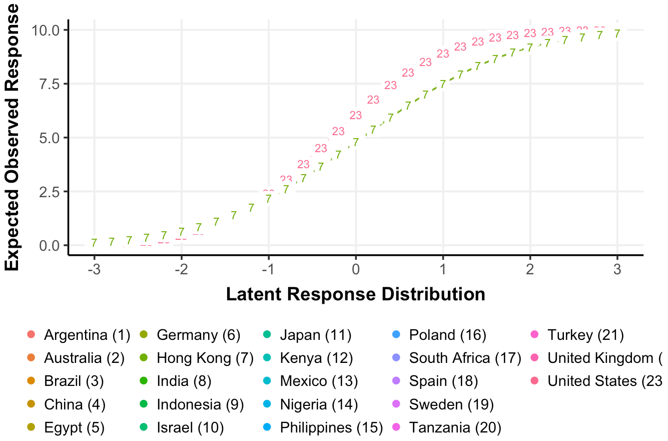


```
format_ft_hetop(fit, 2, cur.var)
```

Orientation to promote good (PROMOTE_GOOD_Y2): Threshold Table.				
threshold	est.unstd	se.unstd	est.unstd.lb	est.unstd.ub
c[1]	-0.97			
c[2]	-0.75			
c[3]	-0.47			
c[4]	-0.32			
c[5]	-0.24			
c[6]	-0.23			
c[7]	-0.04			
c[8]	0.20			
c[9]	0.30			
c[10]	0.74			

```
format_ft_hetop(fit, 1, cur.var)
```

Orientation to promote good (PROMOTE_GOOD_Y2): HETOP summary table when penality is nu=0.												
COUNTRY	mu.std	lambda.std	mu.unstd	mu.unstd.se	mu.unstd.lb	mu.unstd.ub	log.lambda.unstd	log.lambda.unstd.se	log.lambda.unstd.lb	log.lambda.unstd.ub	mu.nonzero	log.lambda.nonzero
Sweden			-0.09				-0.09					
United Kingdom			-0.06				-0.06					
Germany			-0.06				-0.06					
China			-0.06				-0.06					
Argentina			-0.06				-0.06					
Australia			-0.05				-0.05					
Brazil			-0.03				-0.03					
Egypt			-0.02				-0.02					
India			-0.02				-0.02					
Indonesia			-0.00				-0.00					
Israel			0.01				0.01					
Japan			0.03				0.03					
Kenya			0.03				0.03					
Mexico			0.04				0.04					
Nigeria			0.04				0.04					
Philippines			0.05				0.05					
Poland			0.06				0.06					
South Africa			0.08				0.08					
Spain			0.08				0.08					
Tanzania			0.09				0.09					
Turkey			0.09				0.09					
United States			0.10				0.10					
Hong Kong			-0.25				-0.25					

Orientation to promote good (PROMOTE_GOOD_Y2): HETOP summary table when penality is nu=0.

COUNTRY	mu.std	lambda.std	mu.unstd	mu.unstd.se	mu.unstd.lb	mu.unstd.ub	log.lambda.unstd	log.lambda.unstd.se	log.lambda.unstd.lb	log.lambda.unstd.ub	mu.nonzero	log.lambda.nonzero

Note. *significant mu/lambda identified from whether the 95% CI for the parameter contains 0.0. Due to rounding, sometimes the estimate may be '-0.00' [slightly below zero] or '0.00' [slightly above zero]. Standard errors estimated using the inverse of the observed information matrix and can sometimes be singular leading to no estimated standard error for some parameters.

7.2 Delayed gratification

```
i <- i + 1
cur.var <- OUTCOME.LIST[i]
fit <- hetop_pml(
  data = df.raw |> filter(!is.na(ANNUAL_WEIGHT_C2))
  , var = cur.var
  , group = as.name("COUNTRY")
  , wgt = as.name("ANNUAL_WEIGHT_C2")
  , psu =  as.name("PSU")
  , strata = as.name("STRATA")

  , pen = exp(seq(-5,5,0.5))
  , pen.type="alf"
)

get_plot_latent_mean(fit)
```

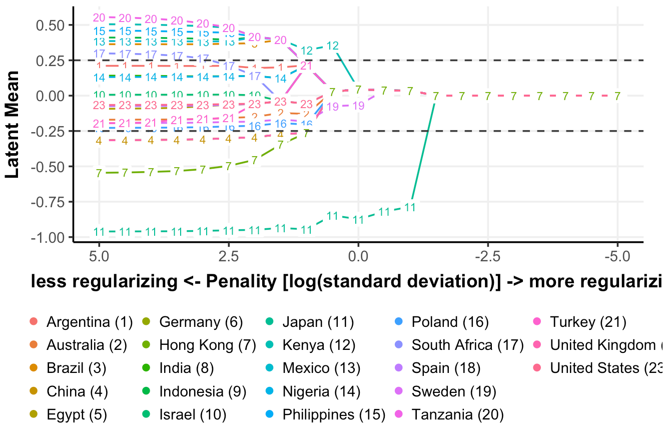


```
get_plot_discrimination(fit)
```

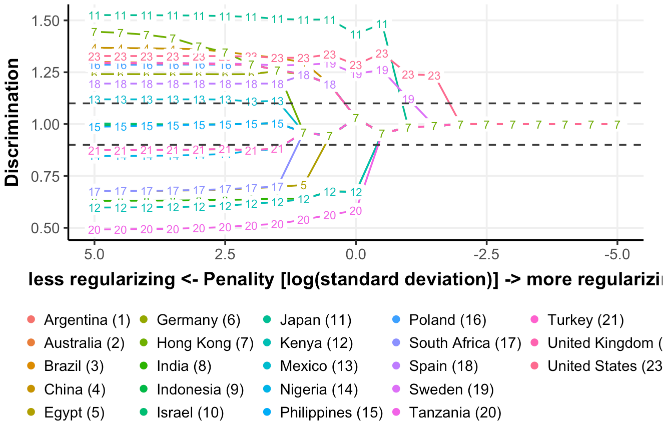


```
plot_iccs(fit, "icc")
```

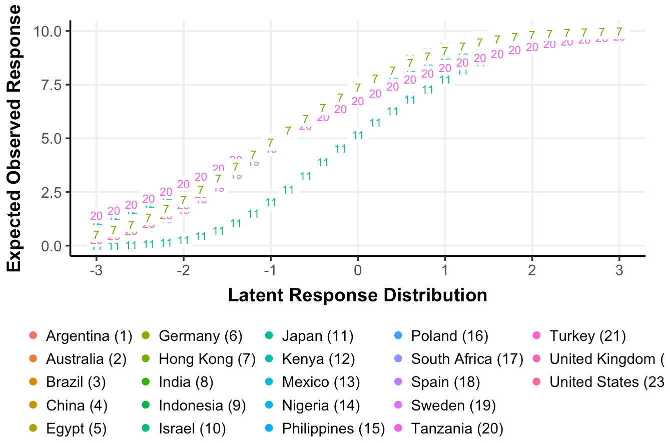


```
format_ft_hetop(fit, 2, cur.var)
```

Delayed gratification (GIVE_UP_Y2): Threshold Table.

threshold	est.unstd	se.unstd	est.unstd.lb	est.unstd.ub
c[1]	-1.99	0.05	-2.08	-1.90
c[2]	-1.87	0.04	-1.96	-1.79
c[3]	-1.71	0.04	-1.79	-1.63
c[4]	-1.48	0.04	-1.55	-1.41
c[5]	-1.25	0.03	-1.31	-1.18
c[6]	-0.78	0.03	-0.83	-0.72
c[7]	-0.47	0.03	-0.52	-0.42
c[8]	-0.07	0.03	-0.12	-0.01
c[9]	0.44	0.03	0.38	0.50
c[10]	0.85	0.04	0.78	0.92

```
format_ft_hetop(fit, 1, cur.var)
```

Delayed gratification (GIVE_UP_Y2): HETOP summary table when penality is nu=0.

COUNTRY	mu.std	lambda.std	mu.unstd	mu.unstd.se	mu.unstd.lb	mu.unstd.ub	log.lambda.unstd	log.lambda.unstd.se	log.lambda.unstd.lb	log.lambda.unstd.ub	mu.nonzero	log.lambda.nonzero
Brazil	0.04	1.03	0.05	0.08	-0.11	0.20	0.03	0.07	-0.10	0.16		
Kenya	0.04	0.69	0.05	0.09	-0.13	0.22	-0.40	0.06	-0.51	-0.28		*
Philippines	0.04	1.03	0.05	0.11	-0.17	0.27	0.03	0.10	-0.17	0.22		
Indonesia	0.04	1.03	0.05	0.11	-0.17	0.26	0.03	0.10	-0.16	0.22		
Mexico	0.04	1.03	0.05	0.12	-0.20	0.29	0.03	0.11	-0.20	0.25		
Tanzania	0.04	0.60	0.05	0.12	-0.19	0.28	-0.54	0.07	-0.67	-0.40		*
Argentina	0.04	1.03	0.05	0.09	-0.13	0.22	0.03	0.08	-0.13	0.19		
India	0.04	0.69	0.04	0.09	-0.14	0.23	-0.39	0.06	-0.51	-0.28		*
Nigeria	0.04	1.03	0.04	0.09	-0.12	0.21	0.03	0.07	-0.10	0.16		
South Africa	0.04	1.03	0.04	0.15	-0.25	0.34	0.03	0.11	-0.18	0.24		
Israel	0.04	1.03	0.04	0.10	-0.14	0.23	0.03	0.08	-0.13	0.18		
Spain	0.04	1.03	0.04	0.09	-0.13	0.22	0.03	0.08	-0.13	0.19		

Note. *significant mu/lambda identified from whether the 95% CI for the parameter contains 0.0. Due to rounding, sometimes the estimate may be '-0.00' [slightly below zero] or '0.00' [slightly above zero]. Standard errors estimated using the inverse of the observed information matrix and can sometimes be singular leading to no estimated standard error for some parameters.

COUNTRY	mu.std	lambda.std	mu.unstd	mu.unstd.se	mu.unstd.lb	mu.unstd.ub	log.lambda.unstd	log.lambda.unstd.se	log.lambda.unstd.lb	log.lambda.unstd.ub	mu.nonzero	log.lambda.nonzero
Turkey	0.04	1.03	0.04	0.22	-0.40	0.49	0.03	0.16	-0.28	0.34		
Australia	0.04	1.03	0.04	0.10	-0.14	0.23	0.03	0.09	-0.15	0.21		
Hong Kong	0.04	1.03	0.04	0.31	-0.55	0.64	0.03	0.25	-0.47	0.53		
Egypt	0.04	1.03	0.04	0.09	-0.14	0.23	0.03	0.06	-0.09	0.15		
United Kingdom	0.04	1.03	0.04	0.09	-0.14	0.23	0.03	0.08	-0.13	0.19		
United States	0.04	1.27	0.04	0.03	-0.02	0.11	0.25	0.03	0.20	0.31		*
Poland	0.04	1.03	0.04	0.07	-0.09	0.17	0.03	0.06	-0.09	0.15		
China	0.04	1.03	0.04	0.08	-0.12	0.21	0.03	0.08	-0.12	0.18		
Germany	0.04	1.03	0.04	0.08	-0.10	0.19	0.03	0.06	-0.10	0.16		
Sweden	-0.06	1.23	-0.07	0.04	-0.15	0.02	0.22	0.04	0.14	0.29		*
Japan	-0.82	1.40	-0.87	0.04	-0.95	-0.80	0.36	0.04	0.29	0.43	*	*

Delayed gratification (GIVE_UP_Y2): HETOP summary table when penality is nu=0.

Note. *significant mu/lambda identified from whether the 95% CI for the parameter contains 0.0. Due to rounding, sometimes the estimate may be '-0.00' [slightly below zero] or '0.00' [slightly above zero]. Standard errors estimated using the inverse of the observed information matrix and can sometimes be singular leading to no estimated standard error for some parameters.

7.3 Hope

```
i <- i + 1
cur.var <- OUTCOME.LIST[i]
fit <- hetop_pml(
  data = df.raw |> filter(!is.na(ANNUAL_WEIGHT_C2))
  , var = cur.var
  , group = as.name("COUNTRY")
  , wgt = as.name("ANNUAL_WEIGHT_C2")
  , psu =  as.name("PSU")
  , strata = as.name("STRATA")
  , pen = exp(seq(-5,5,0.5))
  , pen.type="alf"
)

get_plot_latent_mean(fit)
```

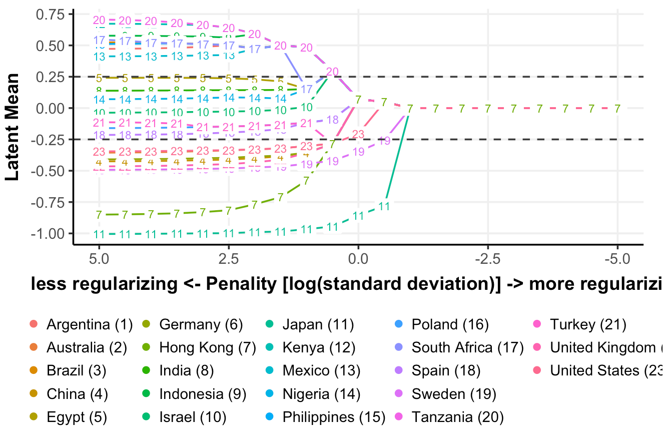


```
get_plot_discrimination(fit)
```

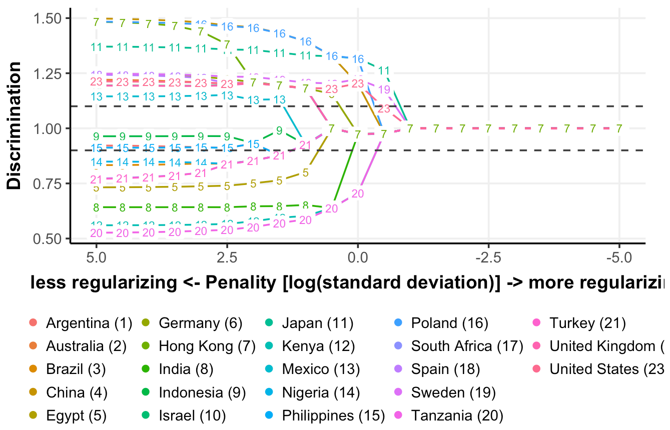


```
plot_iccs(fit, "icc")
```

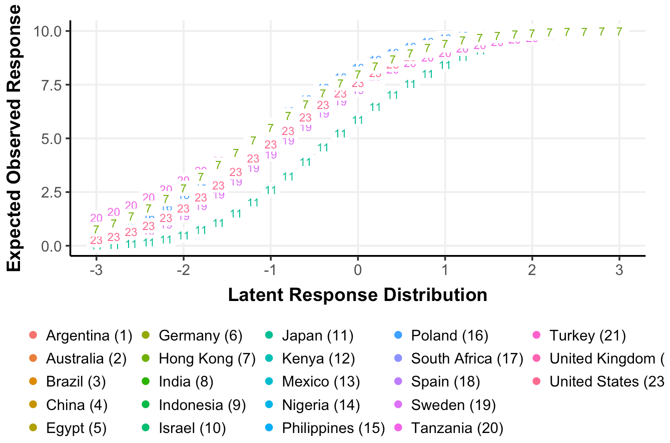


```
format_ft_hetop(fit, 2, cur.var)
```

Hope (HOPE_FUTURE_Y2): Threshold Table.

threshold	est.unstd	se.unstd	est.unstd.lb	est.unstd.ub
c[1]	-2.16	0.06	-2.27	-2.05
c[2]	-2.00	0.05	-2.11	-1.90
c[3]	-1.82	0.05	-1.91	-1.73
c[4]	-1.60	0.04	-1.68	-1.52
c[5]	-1.39	0.04	-1.47	-1.32
c[6]	-1.05	0.03	-1.11	-0.99
c[7]	-0.77	0.03	-0.82	-0.72
c[8]	-0.40	0.02	-0.44	-0.37
c[9]	0.07	0.01	0.05	0.09
c[10]	0.46			

```
format_ft_hetop(fit, 1, cur.var)
```

Hope (HOPE_FUTURE_Y2): HETOP summary table when penality is nu=0.

COUNTRY	mu.std	lambda.std	mu.unstd	mu.unstd.se	mu.unstd.lb	mu.unstd.ub	log.lambda.unstd	log.lambda.unstd.se	log.lambda.unstd.lb	log.lambda.unstd.ub	mu.nonzero	log.lambda.nonzero
Kenya	0.07	0.72	0.07	0.09	-0.10	0.24	-0.35	0.06	-0.47	-0.22	*	

Note. *significant mu/lambda identified from whether the 95% CI for the parameter contains 0.0. Due to rounding, sometimes the estimate may be '-0.00' [slightly below zero] or '0.00' [slightly above zero]. Standard errors estimated using the inverse of the observed information matrix and can sometimes be singular leading to no estimated standard error for some parameters.

Hope (HOPE_FUTURE_Y2): HETOP summary table when penality is nu=0.												
**COUNTRY**	**mu.std**	**lambda.std**	**mu.unstd**	**mu.unstd.se**	**mu.unstd.lb**	**mu.unstd.ub**	**log.lambda.unstd**	**log.lambda.unstd.se**	**log.lambda.unstd.lb**	**log.lambda.unstd.ub**	**mu.nonzero**	**log.lambda.nonzero**
Brazil	0.07	0.97	0.07	0.09	-0.10	0.24	-0.03	0.09	-0.19	0.14		
Indonesia	0.07	0.97	0.07	0.14	-0.19	0.34	-0.03	0.15	-0.32	0.27		
Argentina	0.07	0.97	0.07	0.11	-0.14	0.28	-0.03	0.11	-0.24	0.19		
Tanzania	0.07	0.72	0.07	0.10	-0.12	0.26	-0.35	0.07	-0.48	-0.21		*
Philippines	0.07	0.97	0.07	0.11	-0.15	0.30	-0.03	0.12	-0.26	0.21		
Mexico	0.07	0.97	0.07	0.14	-0.20	0.34	-0.03	0.16	-0.35	0.29		
Egypt	0.07	0.97	0.07	0.09	-0.11	0.25	-0.03	0.08	-0.18	0.12		
South Africa	0.07	0.97	0.07	0.17	-0.27	0.41	-0.03	0.16	-0.35	0.29		
Nigeria	0.07	0.97	0.07	0.09	-0.11	0.25	-0.03	0.08	-0.18	0.13		
Israel	0.07	0.98	0.07	0.10	-0.13	0.27	-0.03	0.11	-0.23	0.18		
Turkey	0.07	0.97	0.07	0.25	-0.41	0.55	-0.03	0.18	-0.38	0.33		
India	0.07	0.97	0.07	0.07	-0.06	0.20	-0.03	0.05	-0.12	0.07		
Spain	0.07	0.98	0.07	0.10	-0.13	0.27	-0.03	0.10	-0.22	0.17		
Hong Kong	0.07	0.97	0.07				-0.03					
Australia	0.07	0.98	0.07	0.12	-0.16	0.30	-0.03	0.11	-0.24	0.18		
Poland	0.07	1.30	0.07	0.05	-0.03	0.17	0.27	0.06	0.16	0.39		*
United Kingdom	0.07	0.98	0.07	0.11	-0.15	0.29	-0.03	0.09	-0.21	0.16		
Germany	0.07	0.98	0.07	0.09	-0.10	0.24	-0.03	0.08	-0.18	0.13		
China	0.07	1.19	0.07	0.08	-0.10	0.24	0.18	0.09	0.01	0.35		*
United States	-0.20	1.19	-0.21	0.03	-0.26	-0.15	0.19	0.03	0.13	0.24	*	*
Sweden	-0.33	1.21	-0.35	0.04	-0.42	-0.27	0.20	0.04	0.12	0.28	*	*
Japan	-0.82	1.30	-0.86	0.04	-0.93	-0.78	0.28	0.04	0.20	0.35	*	*

Note. *significant mu/lambda identified from whether the 95% CI for the parameter contains 0.0. Due to rounding, sometimes the estimate may be '-0.00' [slightly below zero] or '0.00' [slightly above zero]. Standard errors estimated using the inverse of the observed information matrix and can sometimes be singular leading to no estimated standard error for some parameters.

7.4 Gratitude

```
i <- i + 1
cur.var <- OUTCOME.LIST[i]
fit <- hetop_pml(
  data = df.raw |> filter(!is.na(ANNUAL_WEIGHT_C2))
  , var = cur.var
  , group = as.name("COUNTRY")
  , wgt = as.name("ANNUAL_WEIGHT_C2")
  , psu =  as.name("PSU")
  , strata = as.name("STRATA")
  , pen = exp(seq(-5,5,0.5))
  , pen.type="alf"
)

get_plot_latent_mean(fit)
```

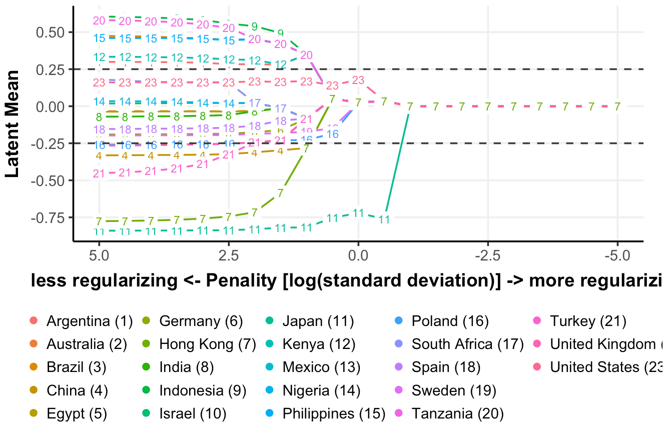


```
get_plot_discrimination(fit)
```

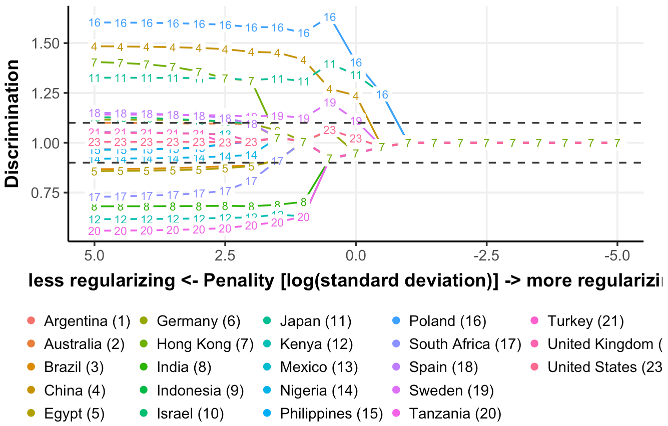


```
plot_iccs(fit, "icc")
```

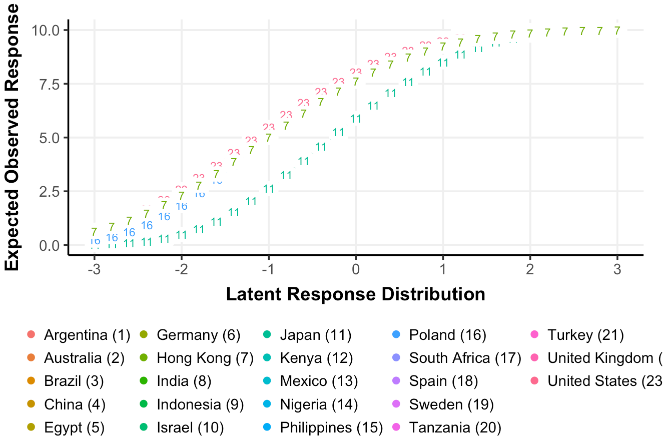


```
format_ft_hetop(fit, 2, cur.var)
```

Gratitude (GRATEFUL_Y2): Threshold Table.

threshold	est.unstd	se.unstd	est.unstd.lb	est.unstd.ub
c[1]	-2.05	0.05	-2.16	-1.94
c[2]	-1.89	0.05	-1.99	-1.80
c[3]	-1.70	0.04	-1.79	-1.62

Gratitude (GRATEFUL_Y2): Threshold Table.				
threshold	est.unstd	se.unstd	est.unstd.lb	est.unstd.ub
c[4]	-1.48	0.04	-1.55	-1.40
c[5]	-1.27	0.03	-1.33	-1.20
c[6]	-0.88	0.02	-0.92	-0.84
c[7]	-0.61	0.01	-0.62	-0.60
c[8]	-0.27			
c[9]	0.16			
c[10]	0.50			

```
format_ft_hetop(fit, 1, cur.var)
```

Gratitude (GRATEFUL_Y2): HETOP summary table when penality is nu=0.												
COUNTRY	mu.std	lambda.std	mu.unstd	mu.unstd.se	mu.unstd.lb	mu.unstd.ub	log.lambda.unstd	log.lambda.unstd.se	log.lambda.unstd.lb	log.lambda.unstd.ub	mu.nonzero	log.lambda.nonzero
United States	0.18	1.02	0.18				0.02	0.03	-0.03	0.07		
Brazil	0.03	0.95	0.03	0.09	-0.14	0.20	-0.05	0.08	-0.22	0.11		
Indonesia	0.03	0.95	0.03	0.16	-0.28	0.33	-0.05	0.16	-0.36	0.26		
Tanzania	0.03	0.95	0.03	0.07	-0.10	0.16	-0.06	0.05	-0.16	0.05		
Philippines	0.03	0.95	0.03	0.12	-0.21	0.26	-0.05	0.12	-0.29	0.18		
Mexico	0.03	0.95	0.03	0.14	-0.25	0.31	-0.05	0.15	-0.35	0.24		
Kenya	0.03	0.95	0.03	0.05	-0.08	0.13	-0.06	0.04	-0.14	0.03		
Argentina	0.03	0.95	0.03	0.10	-0.16	0.22	-0.05	0.10	-0.25	0.14		
Israel	0.03	0.95	0.03	0.10	-0.17	0.22	-0.05	0.10	-0.26	0.15		
Nigeria	0.03	0.95	0.03	0.09	-0.14	0.20	-0.05	0.08	-0.21	0.10		
Australia	0.03	0.95	0.03	0.10	-0.16	0.22	-0.05	0.10	-0.25	0.14		
South Africa	0.03	0.95	0.03	0.16	-0.29	0.35	-0.05	0.13	-0.31	0.21		
Egypt	0.03	0.95	0.03	0.09	-0.15	0.20	-0.05	0.08	-0.21	0.10		
Turkey	0.03	0.95	0.03	0.30	-0.57	0.62	-0.05	0.24	-0.52	0.42		
Spain	0.03	0.95	0.03	0.09	-0.15	0.21	-0.05	0.09	-0.24	0.13		
Hong Kong	0.03	0.95	0.03				-0.05					
Germany	0.02	0.95	0.03	0.06	-0.10	0.15	-0.05	0.07	-0.18	0.08		
United Kingdom	0.02	0.95	0.03	0.09	-0.14	0.19	-0.05	0.08	-0.21	0.10		
India	0.02	0.95	0.03	0.06	-0.09	0.14	-0.06	0.05	-0.15	0.04		
China	0.02	1.23	0.03	0.06	-0.09	0.14	0.21	0.07	0.07	0.36		*
Poland	0.02	1.39	0.02	0.03	-0.03	0.08	0.34	0.06	0.23	0.45		*
Sweden	0.02	1.11	0.02	0.02	-0.02	0.07	0.11	0.04	0.03	0.19		*
Japan	-0.70	1.33	-0.72	0.03	-0.78	-0.66	0.29	0.03	0.23	0.36	*	*

Note. *significant mu/lambda identified from whether the 95% CI for the parameter contains 0.0. Due to rounding, sometimes the estimate may be '-0.00' [slightly below zero] or '0.00' [slightly above zero]. Standard errors estimated using the inverse of the observed information matrix and can sometimes be singular leading to no estimated standard error for some parameters.

7.5 Showing love/care

```
i <- i + 1
cur.var <- OUTCOME.LIST[i]
fit <- hetop_pml(
  data = df.raw |> filter(!is.na(ANNUAL_WEIGHT_C2))
```

```r
  , var = cur.var
  , group = as.name("COUNTRY")
  , wgt = as.name("ANNUAL_WEIGHT_C2")
  , psu =  as.name("PSU")
  , strata = as.name("STRATA")
  , pen = exp(seq(-5,5,0.5))
  , pen.type="alf"
)

get_plot_latent_mean(fit)
```

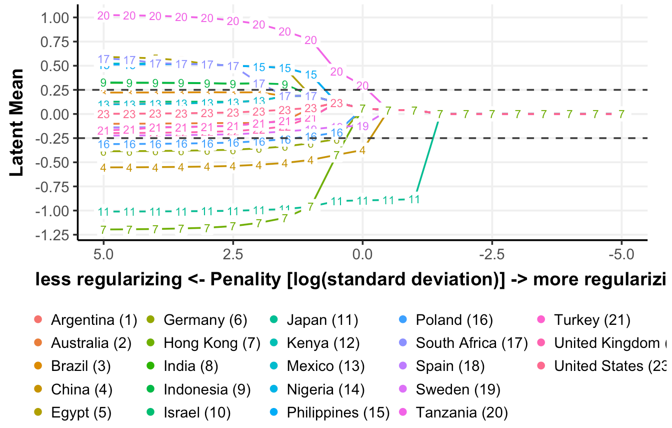


```r
get_plot_discrimination(fit)
```

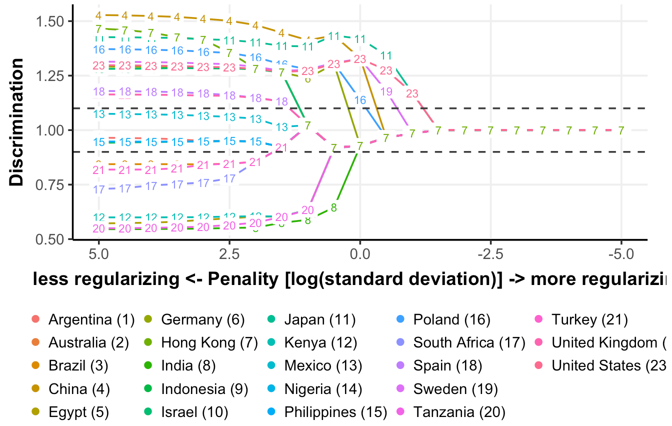


```r
plot_iccs(fit, "icc")
```

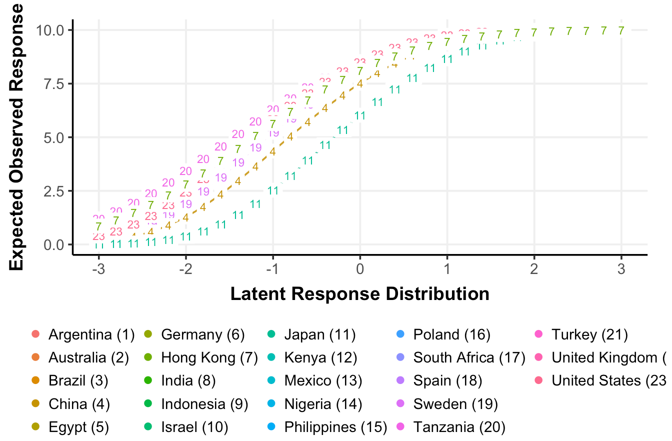


```r
format_ft_hetop(fit, 2, cur.var)
```

Showing love/care (SHOW_LOVE_Y2): Threshold Table.				
threshold	est.unstd	se.unstd	est.unstd.lb	est.unstd.ub
c[1]	-2.15	0.06	-2.26	-2.04
c[2]	-1.99	0.05	-2.09	-1.89
c[3]	-1.83	0.05	-1.92	-1.74
c[4]	-1.64	0.04	-1.72	-1.56
c[5]	-1.47	0.04	-1.54	-1.39
c[6]	-1.11	0.03	-1.17	-1.05
c[7]	-0.86	0.03	-0.91	-0.80
c[8]	-0.52	0.02	-0.57	-0.47
c[9]	-0.08	0.02	-0.13	-0.04
c[10]	0.31	0.02	0.26	0.35

```
format_ft_hetop(fit, 1, cur.var)
```

Showing love/care (SHOW_LOVE_Y2): HETOP summary table when penality is nu=0.												
COUNTRY	mu.std	lambda.std	mu.unstd	mu.unstd.se	mu.unstd.lb	mu.unstd.ub	log.lambda.unstd	log.lambda.unstd.se	log.lambda.unstd.lb	log.lambda.unstd.ub	mu.nonzero	log.lambda.nonzero
Tanzania	0.28	0.93	0.29	0.08	0.14	0.45	-0.07	0.06	-0.20	0.05	*	
Kenya	0.06	0.93	0.06	0.07	-0.07	0.19	-0.07	0.05	-0.18	0.03		
Philippines	0.06	0.93	0.06	0.12	-0.18	0.29	-0.07	0.14	-0.35	0.20		
Indonesia	0.06	0.93	0.06	0.11	-0.15	0.27	-0.07	0.11	-0.30	0.15		
Brazil	0.06	0.93	0.06	0.09	-0.11	0.23	-0.07	0.08	-0.23	0.08		
United States	0.06	1.31	0.06	0.03	-0.00	0.12	0.28	0.03	0.22	0.35		*
Egypt	0.06	0.93	0.06	0.10	-0.14	0.26	-0.07	0.08	-0.23	0.08		
Israel	0.06	0.93	0.06	0.11	-0.16	0.27	-0.07	0.13	-0.34	0.19		
Argentina	0.06	0.93	0.06	0.10	-0.14	0.26	-0.07	0.10	-0.27	0.12		
Mexico	0.06	0.93	0.06	0.11	-0.17	0.28	-0.07	0.12	-0.31	0.16		
South Africa	0.06	0.93	0.06	0.18	-0.29	0.41	-0.07	0.17	-0.40	0.25		
India	0.06	0.93	0.06	0.07	-0.09	0.20	-0.08	0.05	-0.18	0.03		
Australia	0.06	0.93	0.06	0.11	-0.16	0.27	-0.07	0.12	-0.31	0.16		
Turkey	0.06	0.93	0.06	0.28	-0.49	0.60	-0.07	0.20	-0.47	0.32		
Spain	0.06	0.93	0.06	0.11	-0.15	0.27	-0.07	0.10	-0.28	0.13		
Nigeria	0.06	0.93	0.06	0.11	-0.15	0.27	-0.07	0.09	-0.25	0.10		
United Kingdom	0.06	0.93	0.06	0.10	-0.14	0.26	-0.07	0.09	-0.26	0.11		
Hong Kong	0.06	0.93	0.06				-0.07					
Germany	0.05	0.93	0.06	0.10	-0.14	0.25	-0.07	0.09	-0.25	0.10		
Poland	0.05	1.13	0.06	0.07	-0.09	0.20	0.13	0.07	-0.01	0.27		
Sweden	-0.12	1.31	-0.12	0.04	-0.20	-0.04	0.28	0.04	0.20	0.37	*	*
China	-0.36	1.31	-0.37	0.06	-0.49	-0.26	0.28	0.07	0.15	0.42	*	*
Japan	-0.86	1.40	-0.89	0.04	-0.97	-0.82	0.35	0.04	0.28	0.43	*	*

Note. *significant mu/lambda identified from whether the 95% CI for the parameter contains 0.0. Due to rounding, sometimes the estimate may be '-0.00' [slightly below zero] or '0.00' [slightly above zero]. Standard errors estimated using the inverse of the observed information matrix and can sometimes be singular leading to no estimated standard error for some parameters.

7.6 Forgivingness

```r
i <- i + 1
cur.var <- OUTCOME.LIST[i]
fit <- hetop_pml(
  data = df.raw |> filter(!is.na(ANNUAL_WEIGHT_C2))
  , var = cur.var
  , group = as.name("COUNTRY")
  , wgt = as.name("ANNUAL_WEIGHT_C2")
  , psu =  as.name("PSU")
  , strata = as.name("STRATA")
  , pen = exp(seq(-5,5,0.5))
  , pen.type="alf"
)

get_plot_latent_mean(fit)
```

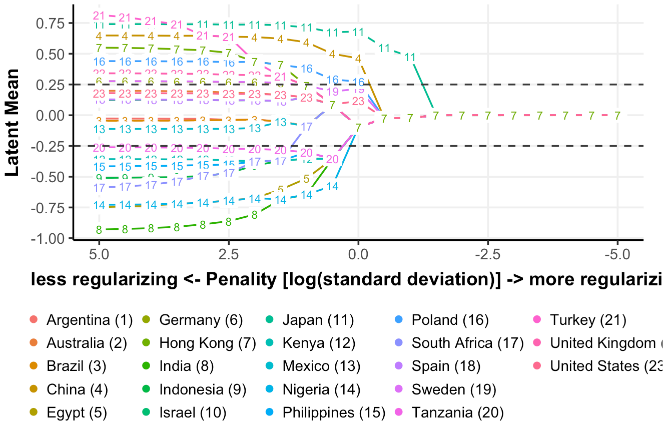


```r
get_plot_discrimination(fit)
```

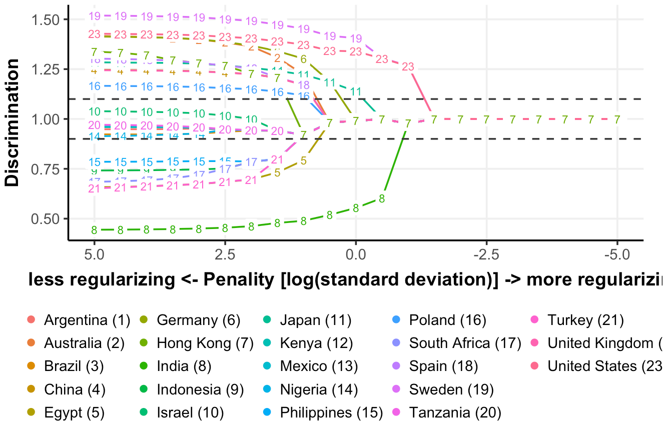


```r
plot_iccs(fit, "icc")
```

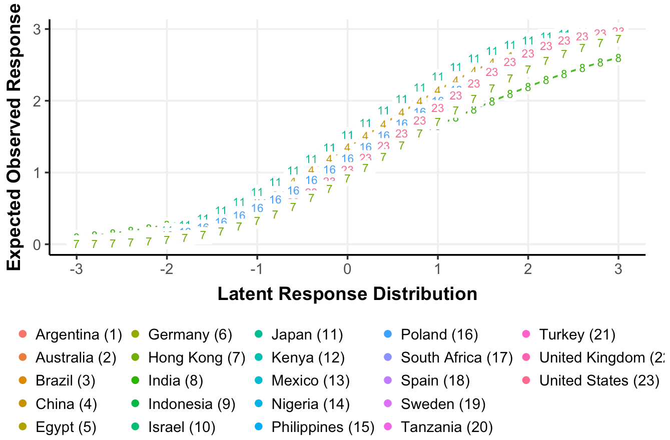

```r
format_ft_hetop(fit, 2, cur.var)
```

Forgivingness (FORGIVE_Y2): Threshold Table.				
threshold	est.unstd	se.unstd	est.unstd.lb	est.unstd.ub
c[1]	-0.53	0.04	-0.62	-0.44
c[2]	0.69	0.03	0.63	0.76
c[3]	1.73	0.04	1.65	1.81

```r
format_ft_hetop(fit, 1, cur.var)
```

Forgivingness (FORGIVE_Y2): HETOP summary table when penality is nu=0.

COUNTRY	mu.std	lambda.std	mu.unstd	mu.unstd.se	mu.unstd.lb	mu.unstd.ub	log.lambda.unstd	log.lambda.unstd.se	log.lambda.unstd.lb	log.lambda.unstd.ub	mu.nonzero	log.lambda.nonzero
Japan	0.65	1.13	0.68	0.05	0.59	0.78	0.13	0.04	0.04	0.21	*	*
China	0.44	0.99	0.46	0.08	0.30	0.63	-0.01	0.07	-0.15	0.14	*	
Poland	0.26	0.99	0.27	0.07	0.13	0.42	-0.01	0.06	-0.13	0.11	*	
Sweden	0.20	1.38	0.21	0.05	0.12	0.31	0.34	0.04	0.25	0.43	*	*
United States	0.11	1.32	0.12	0.04	0.03	0.20	0.29	0.03	0.23	0.36	*	*
Germany	-0.09	0.99	-0.10	0.09	-0.28	0.09	-0.01	0.08	-0.17	0.15		
United Kingdom	-0.09	0.99	-0.10	0.12	-0.33	0.14	-0.01	0.10	-0.20	0.18		
Australia	-0.09	0.99	-0.10	0.12	-0.33	0.14	-0.01	0.11	-0.23	0.21		
Turkey	-0.09	0.99	-0.10	0.61	-1.29	1.10	-0.01	0.34	-0.67	0.65		
Spain	-0.09	0.99	-0.10	0.11	-0.31	0.12	-0.01	0.10	-0.21	0.19		
Hong Kong	-0.09	0.99	-0.10	0.40	-0.89	0.69	-0.01	0.31	-0.62	0.60		
Israel	-0.09	0.99	-0.10	0.12	-0.33	0.14	-0.01	0.10	-0.20	0.18		
Brazil	-0.09	0.99	-0.10	0.09	-0.27	0.08	-0.01	0.07	-0.15	0.13		
Argentina	-0.09	0.99	-0.10	0.10	-0.30	0.11	-0.01	0.09	-0.18	0.16		
Mexico	-0.09	0.99	-0.10	0.11	-0.32	0.13	-0.01	0.10	-0.20	0.18		
South Africa	-0.09	0.99	-0.10	0.17	-0.43	0.24	-0.01	0.14	-0.29	0.27		
Philippines	-0.09	0.99	-0.10	0.11	-0.30	0.11	-0.01	0.09	-0.19	0.17		
Indonesia	-0.09	0.99	-0.10	0.11	-0.31	0.11	-0.01	0.09	-0.19	0.17		
Tanzania	-0.09	0.99	-0.10	0.08	-0.25	0.05	-0.01	0.07	-0.14	0.13		
India	-0.09	0.57	-0.10	0.12	-0.34	0.15	-0.59	0.08	-0.75	-0.43		*
Egypt	-0.09	0.99	-0.10	0.10	-0.30	0.11	-0.01	0.09	-0.18	0.16		
Nigeria	-0.09	0.99	-0.10	0.13	-0.35	0.15	-0.01	0.14	-0.28	0.26		
Kenya	-0.09	0.99	-0.10	0.07	-0.23	0.04	-0.01	0.06	-0.13	0.11		

Note. *significant mu/lambda identified from whether the 95% CI for the parameter contains 0.0. Due to rounding, sometimes the estimate may be '-0.00' [slightly below zero] or '0.00' [slightly above zero]. Standard errors estimated using the inverse of the observed information matrix and can sometimes be singular leading to no estimated standard error for some parameters.

7.7 Charitable giving

```r
i <- i + 1
cur.var <- OUTCOME.LIST[i]
fit <- hetop_pml(
  data = df.raw |> filter(!is.na(ANNUAL_WEIGHT_C2))
  , var = cur.var
  , group = as.name("COUNTRY")
  , wgt = as.name("ANNUAL_WEIGHT_C2")
  , psu =  as.name("PSU")
  , strata = as.name("STRATA")
  , pen = exp(seq(-5,5,0.5))
  , pen.type="alf"
```

```
)

get_plot_latent_mean(fit)
```

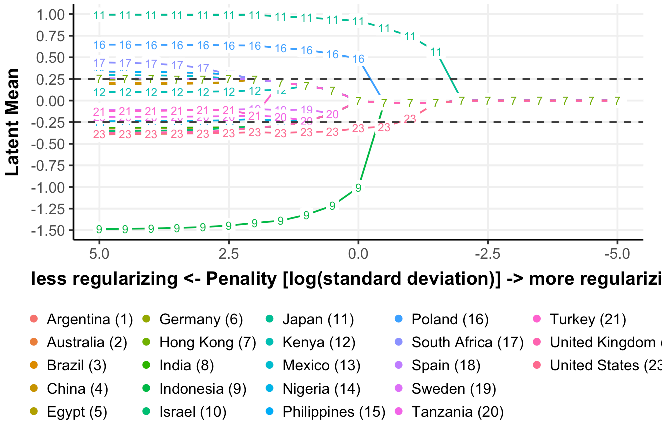


```
get_plot_discrimination(fit)
```

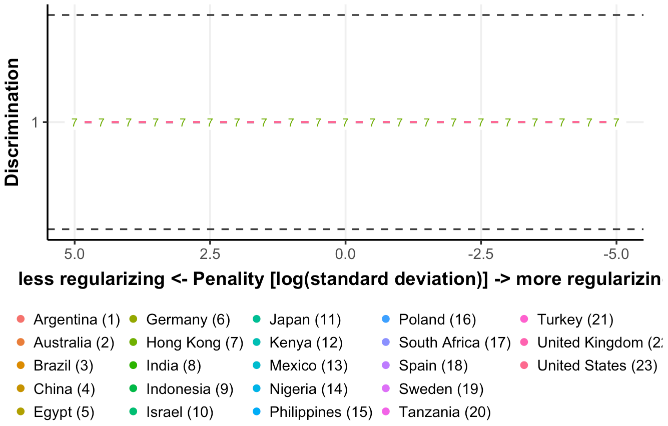


```
plot_iccs(fit, "icc")
```

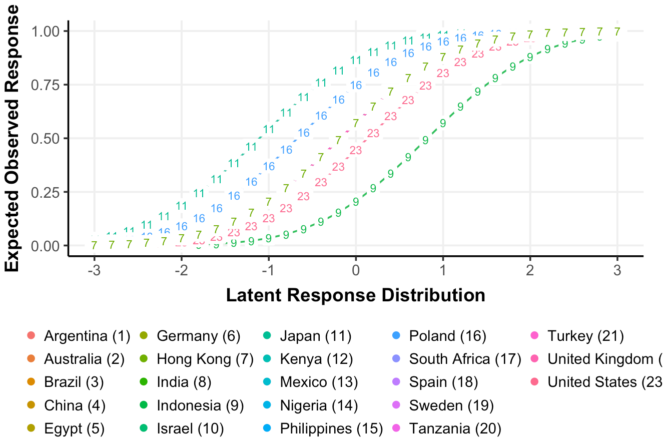


```
format_ft_hetop(fit, 2, cur.var)
```

Charitable giving (DONATED_Y2): Threshold Table.

threshold	est.unstd	se.unstd	est.unstd.lb	est.unstd.ub
c[1]	-0.18	0.03	-0.23	-0.13

```r
format_ft_hetop(fit, 1, cur.var)
```

Charitable giving (DONATED_Y2): HETOP summary table when penality is nu=0.												
**COUNTRY**	**mu.std**	**lambda.std**	**mu.unstd**	**mu.unstd.se**	**mu.unstd.lb**	**mu.unstd.ub**	**log.lambda.unstd**	**log.lambda.unstd.se**	**log.lambda.unstd.lb**	**log.lambda.unstd.ub**	**mu.nonzero**	**log.lambda.nonzero**
Japan	0.88	1.00	0.92	0.22	0.48	1.36	0.00	0.22	-0.44	0.44	*	
Poland	0.46	1.00	0.49	0.05	0.40	0.57	0.00				*	
Germany	-0.00	1.00	-0.00	0.09	-0.18	0.17	0.00	0.35	-0.70	0.70		
Argentina	-0.00	1.00	-0.00	0.11	-0.22	0.21	0.00	0.03	-0.06	0.06		
Kenya	-0.00	1.00	-0.00	0.07	-0.14	0.13	0.00					
China	-0.00	1.00	-0.00	0.09	-0.17	0.16	0.00					
Brazil	-0.00	1.00	-0.00	0.08	-0.17	0.16	0.00					
Philippines	-0.00	1.00	-0.00	0.09	-0.17	0.16	0.00					
Spain	-0.00	1.00	-0.00	0.09	-0.18	0.17	0.00					
Mexico	-0.00	1.00	-0.00	0.10	-0.19	0.18	0.00					
South Africa	-0.00	1.00	-0.00	0.12	-0.25	0.24	-0.00					
Hong Kong	-0.00	1.00	-0.00	0.19	-0.38	0.37	-0.00					
Turkey	-0.00	1.00	-0.00	0.25	-0.50	0.49	-0.00					
Nigeria	-0.00	1.00	-0.00	0.11	-0.21	0.20	-0.00					
Australia	-0.00	1.00	-0.00	0.11	-0.22	0.21	-0.00					
Israel	-0.00	1.00	-0.00	0.05	-0.11	0.10	-0.00					
Tanzania	-0.00	1.00	-0.00	0.08	-0.16	0.15	-0.00					
Egypt	-0.00	1.00	-0.00	0.09	-0.18	0.17	-0.00					
United Kingdom	-0.00	1.00	-0.00	0.05	-0.10	0.09	-0.00					
India	-0.00	1.00	-0.01				-0.00					
Sweden	-0.00	1.00	-0.01				-0.00					
United States	-0.31	1.00	-0.32	0.07	-0.46	-0.18	0.00	0.59	-1.16	1.16	*	
Indonesia	-0.96	1.00	-1.00	0.10	-1.20	-0.81	0.00				*	

Note. *significant mu/lambda identified from whether the 95% CI for the parameter contains 0.0. Due to rounding, sometimes the estimate may be '-0.00' [slightly below zero] or '0.00' [slightly above zero]. Standard errors estimated using the inverse of the observed information matrix and can sometimes be singular leading to no estimated standard error for some parameters.

7.8 Helping strangers

```r
i <- i + 1
cur.var <- OUTCOME.LIST[i]
fit <- hetop_pml(
  data = df.raw |> filter(!is.na(ANNUAL_WEIGHT_C2))
  , var = cur.var
  , group = as.name("COUNTRY")
  , wgt = as.name("ANNUAL_WEIGHT_C2")
  , psu =  as.name("PSU")
  , strata = as.name("STRATA")

  , pen = exp(seq(-5,5,0.5))
  , pen.type="alf"
)

get_plot_latent_mean(fit)
```

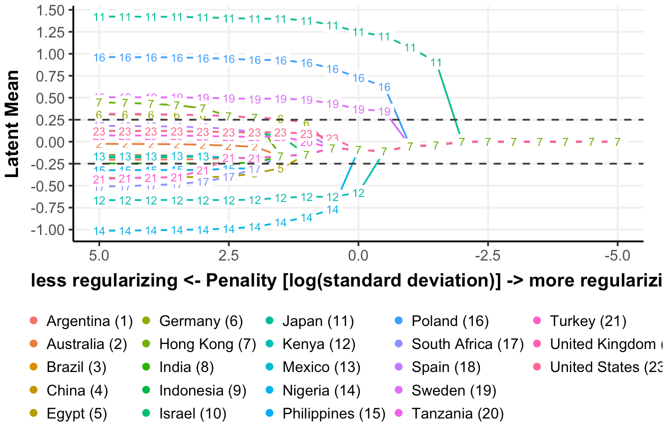


```
get_plot_discrimination(fit)
```

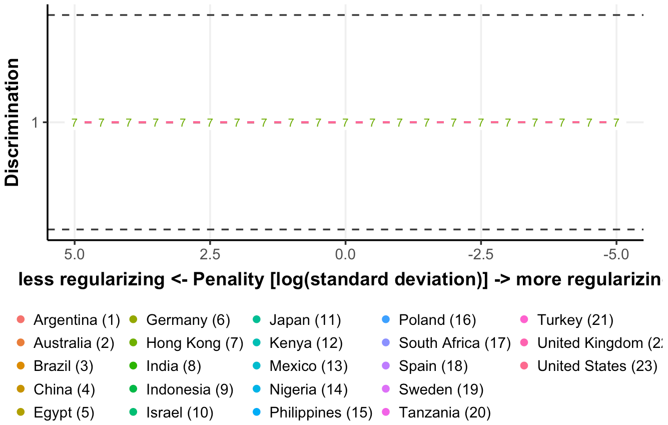


```
plot_iccs(fit, "icc")
```

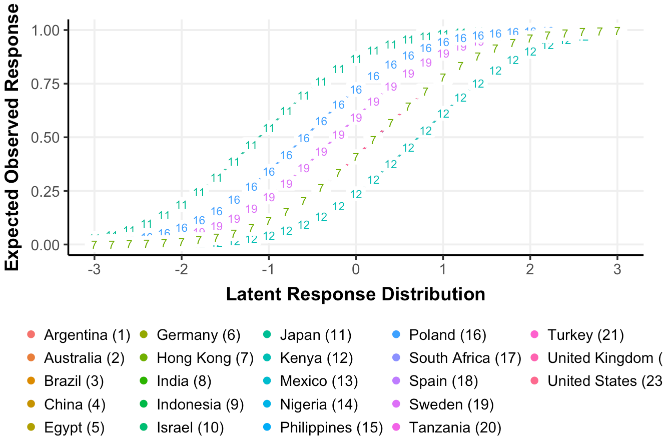


```
format_ft_hetop(fit, 2, cur.var)
```

Helping strangers (HELP_STRANGER_Y2): Threshold Table.

threshold	est.unstd	se.unstd	est.unstd.lb	est.unstd.ub
c[1]	0.14	0.02	0.10	0.18

```
format_ft_hetop(fit, 1, cur.var)
```

Helping strangers (HELP_STRANGER_Y2): HETOP summary table when penality is nu=0.

COUNTRY	mu.std	lambda.std	mu.unstd	mu.unstd.se	mu.unstd.lb	mu.unstd.ub	log.lambda.unstd	log.lambda.unstd.se	log.lambda.unstd.lb	log.lambda.unstd.ub	mu.nonzero	log.lambda.nonzero
Japan	1.18	1.00	1.25	0.20	0.87	1.63	0.00	0.18	-0.36	0.36	*	
Poland	0.69	1.00	0.73	0.01	0.70	0.76	0.00				*	
Sweden	0.35	1.00	0.37	0.13	0.12	0.62	0.00	0.63	-1.24	1.24	*	
United States	-0.09	1.00	-0.09				-0.00					
Germany	-0.09	1.00	-0.09				-0.00					
United Kingdom	-0.09	1.00	-0.09				-0.00					
Spain	-0.09	1.00	-0.09	0.08	-0.25	0.07	-0.00					
Tanzania	-0.09	1.00	-0.09	0.05	-0.19	0.00	-0.00					
Hong Kong	-0.09	1.00	-0.09				-0.00					
Israel	-0.09	1.00	-0.09	0.14	-0.36	0.17	-0.00	0.21	-0.40	0.40		
Australia	-0.09	1.00	-0.09	0.12	-0.33	0.14	-0.00	0.17	-0.33	0.33		
Turkey	-0.09	1.00	-0.09	0.22	-0.52	0.33	-0.00					
Mexico	-0.09	1.00	-0.09	0.09	-0.27	0.09	-0.00					
South Africa	-0.09	1.00	-0.09	0.14	-0.36	0.17	0.00					
Indonesia	-0.09	1.00	-0.09	0.12	-0.33	0.15	0.00					
Argentina	-0.09	1.00	-0.09	0.12	-0.33	0.14	0.00	1.42	-2.77	2.77		
Brazil	-0.09	1.00	-0.09	0.10	-0.28	0.09	0.00	0.52	-1.03	1.03		
China	-0.09	1.00	-0.09	0.12	-0.32	0.14	0.00	0.96	-1.88	1.88		
Philippines	-0.09	1.00	-0.09	0.12	-0.32	0.13	0.00					
Egypt	-0.09	1.00	-0.09	0.11	-0.31	0.12	0.00	0.05	-0.10	0.10		
India	-0.09	1.00	-0.09	0.08	-0.24	0.05	0.00					
Nigeria	-0.09	1.00	-0.09	0.06	-0.21	0.03	0.00					
Kenya	-0.55	1.00	-0.58	0.14	-0.86	-0.30	0.00	0.23	-0.44	0.44	*	

Note. *significant mu/lambda identified from whether the 95% CI for the parameter contains 0.0. Due to rounding, sometimes the estimate may be '-0.00' [slightly below zero] or '0.00' [slightly above zero]. Standard errors estimated using the inverse of the observed information matrix and can sometimes be singular leading to no estimated standard error for some parameters.

7.9 Volunteering

```r
i <- i + 1
cur.var <- OUTCOME.LIST[i]
fit <- hetop_pml(
  data = df.raw |> filter(!is.na(ANNUAL_WEIGHT_C2))
  , var = cur.var
  , group = as.name("COUNTRY")
  , wgt = as.name("ANNUAL_WEIGHT_C2")
  , psu =  as.name("PSU")
  , strata = as.name("STRATA")

  , pen = exp(seq(-5,5,0.5))
  , pen.type="alf"
)

get_plot_latent_mean(fit)
```

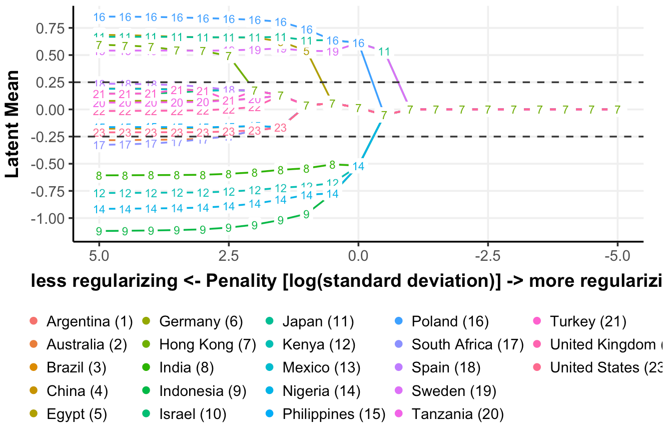


```
get_plot_discrimination(fit)
```

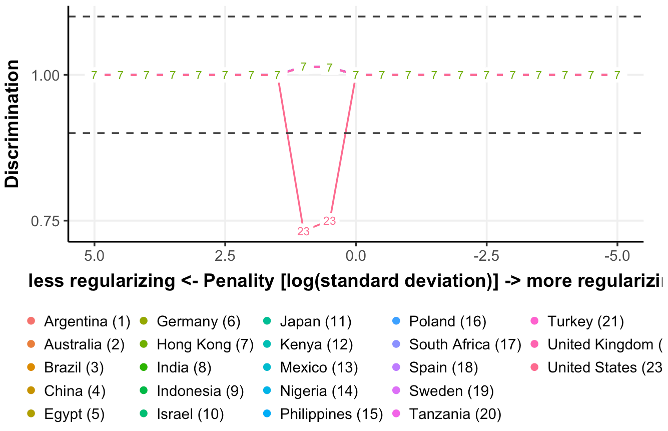


```
plot_iccs(fit, "icc")
```

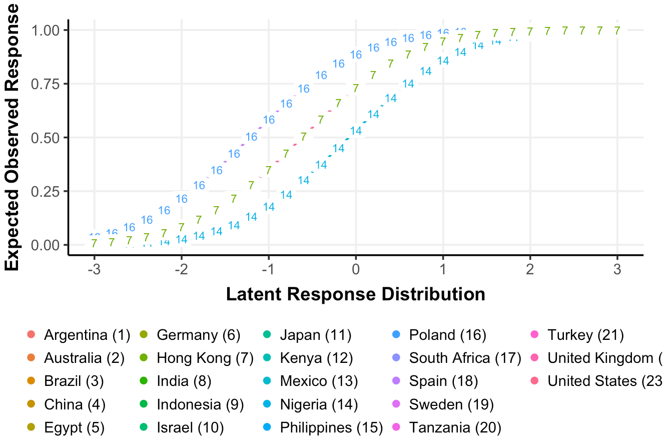


```
format_ft_hetop(fit, 2, cur.var)
```

Volunteering (VOLUNTEERED_Y2): Threshold Table.

threshold	est.unstd	se.unstd	est.unstd.lb	est.unstd.ub
c[1]	-0.60	0.04	-0.67	-0.53

```
format_ft_hetop(fit, 1, cur.var)
```

Volunteering (VOLUNTEERED_Y2): HETOP summary table when penality is nu=0.												
**COUNTRY**	**mu.std**	**lambda.std**	**mu.unstd**	**mu.unstd.se**	**mu.unstd.lb**	**mu.unstd.ub**	**log.lambda.unstd**	**log.lambda.unstd.se**	**log.lambda.unstd.lb**	**log.lambda.unstd.ub**	**mu.nonzero**	**log.lambda.nonzero**
Japan	0.59	1.00	0.61	0.20	0.23	1.00	0.00	0.18	-0.34	0.34	*	
Poland	0.59	1.00	0.61	0.22	0.18	1.05	0.00	0.22	-0.43	0.43	*	
Sweden	0.58	1.00	0.61	0.53	-0.43	1.66	0.00	0.46	-0.90	0.90		
Egypt	0.01	1.00	0.02	0.12	-0.23	0.26	0.00					
Brazil	0.01	1.00	0.02				0.00					
Spain	0.01	1.00	0.02	0.10	-0.18	0.21	0.00					
Germany	0.01	1.00	0.02				0.00					
Tanzania	0.01	1.00	0.02	0.15	-0.28	0.31	0.00	0.24	-0.46	0.46		
Argentina	0.01	1.00	0.02	0.09	-0.16	0.19	0.00					
Mexico	0.01	1.00	0.02	0.14	-0.25	0.28	0.00					
Israel	0.01	1.00	0.02	0.15	-0.27	0.30	0.00	0.16	-0.31	0.31		
Hong Kong	0.01	1.00	0.02	0.25	-0.47	0.50	0.00	0.70	-1.36	1.36		
United Kingdom	0.01	1.00	0.02	0.33	-0.64	0.67	-0.00	0.58	-1.14	1.14		
Turkey	0.01	1.00	0.01	0.28	-0.53	0.56	-0.00					
Philippines	0.01	1.00	0.01	0.09	-0.16	0.19	-0.00					
South Africa	0.01	1.00	0.01	0.15	-0.29	0.32	-0.00					
China	0.01	1.00	0.01				-0.00					
Australia	0.01	1.00	0.01				-0.00					
United States	0.01	1.00	0.01				-0.00					
India	-0.49	1.00	-0.52	0.08	-0.68	-0.36	-0.00				*	
Nigeria	-0.50	1.00	-0.52	0.10	-0.72	-0.32	-0.00				*	
Kenya	-0.50	1.00	-0.52	0.07	-0.66	-0.38	-0.00				*	
Indonesia	-0.50	1.00	-0.52				-0.00					

Note. *significant mu/lambda identified from whether the 95% CI for the parameter contains 0.0. Due to rounding, sometimes the estimate may be '-0.00' [slightly below zero] or '0.00' [slightly above zero]. Standard errors estimated using the inverse of the observed information matrix and can sometimes be singular leading to no estimated standard error for some parameters.

8 Physical Health & Health Behavior

8.1 Self-rated physical health

```
i <- i + 1
cur.var <- OUTCOME.LIST[i]
fit <- hetop_pml(
  data = df.raw |> filter(!is.na(ANNUAL_WEIGHT_C2))
  , var = cur.var
  , group = as.name("COUNTRY")
  , wgt = as.name("ANNUAL_WEIGHT_C2")
  , psu =  as.name("PSU")
  , strata = as.name("STRATA")

  , pen = exp(seq(-5,5,0.5))
  , pen.type="alf"
)

get_plot_latent_mean(fit)
```

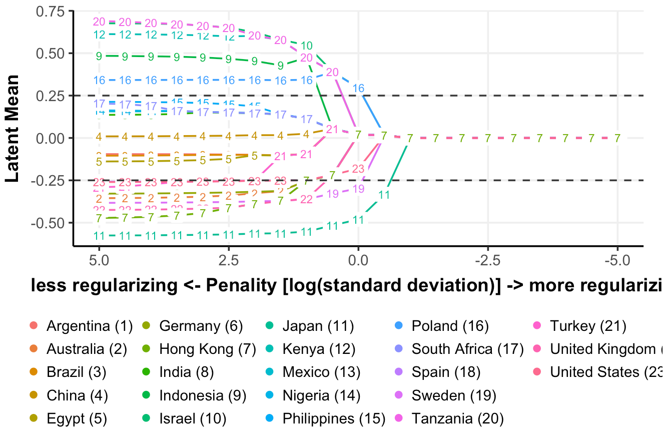


```
get_plot_discrimination(fit)
```

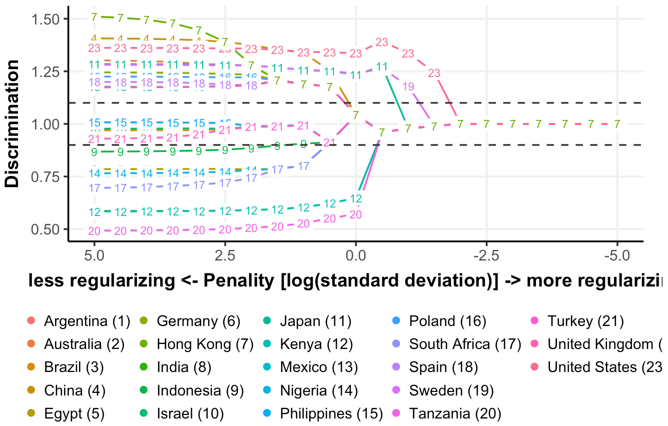


```
plot_iccs(fit, "icc")
```

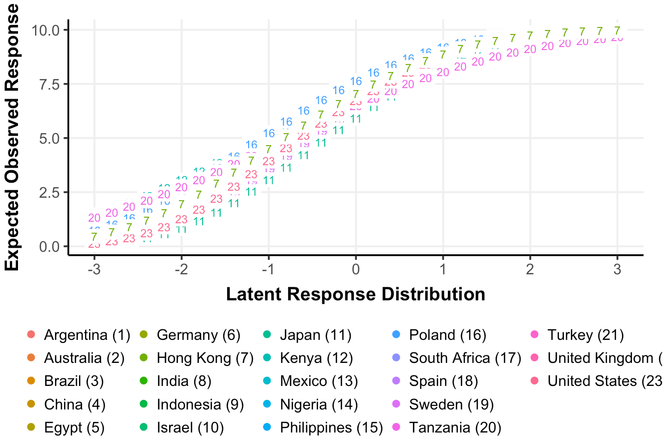


```
format_ft_hetop(fit, 2, cur.var)
```

Self-rated physical health (PHYSICAL_HLTH_Y2): Threshold Table.

threshold	est.unstd	se.unstd	est.unstd.lb	est.unstd.ub
c[1]	-2.07	0.05	-2.17	-1.97
c[2]	-1.89	0.04	-1.98	-1.81

Self-rated physical health (PHYSICAL_HLTH_Y2): Threshold Table.				
threshold	est.unstd	se.unstd	est.unstd.lb	est.unstd.ub
c[3]	-1.64	0.04	-1.72	-1.56
c[4]	-1.35	0.03	-1.42	-1.29
c[5]	-1.07	0.03	-1.13	-1.02
c[6]	-0.71	0.03	-0.76	-0.65
c[7]	-0.38	0.03	-0.43	-0.33
c[8]	0.06	0.03	0.01	0.12
c[9]	0.60	0.03	0.54	0.66
c[10]	1.04	0.04	0.97	1.11

```
format_ft_hetop(fit, 1, cur.var)
```

Self-rated physical health (PHYSICAL_HLTH_Y2): HETOP summary table when penality is nu=0.												
COUNTRY	mu.std	lambda.std	mu.unstd	mu.unstd.se	mu.unstd.lb	mu.unstd.ub	log.lambda.unstd	log.lambda.unstd.se	log.lambda.unstd.lb	log.lambda.unstd.ub	mu.nonzero	log.lambda.nonzero
Kenya	0.28	0.66	0.30	0.09	0.12	0.47	-0.43	0.06	-0.54	-0.32	*	*
Poland	0.28	1.04	0.30	0.06	0.17	0.42	0.04	0.06	-0.08	0.16	*	
Israel	0.02	1.04	0.02	0.17	-0.31	0.35	0.04	0.14	-0.23	0.31		
Indonesia	0.02	1.04	0.02	0.11	-0.19	0.23	0.04	0.08	-0.12	0.21		
Tanzania	0.02	0.59	0.02	0.12	-0.22	0.26	-0.56	0.07	-0.70	-0.42		*
Philippines	0.02	1.04	0.02	0.09	-0.16	0.21	0.04	0.08	-0.11	0.20		
Mexico	0.02	1.04	0.02	0.10	-0.18	0.22	0.04	0.09	-0.14	0.22		
Nigeria	0.02	1.04	0.02	0.09	-0.15	0.19	0.04	0.06	-0.08	0.16		
India	0.02	0.66	0.02	0.10	-0.17	0.21	-0.43	0.06	-0.55	-0.32		*
China	0.02	1.04	0.02	0.07	-0.12	0.16	0.04	0.07	-0.10	0.18		
South Africa	0.02	1.04	0.02	0.15	-0.27	0.31	0.04	0.10	-0.15	0.24		
Turkey	0.02	1.04	0.02	0.22	-0.41	0.46	0.04	0.16	-0.27	0.35		
Argentina	0.02	1.04	0.02	0.09	-0.15	0.19	0.04	0.08	-0.11	0.19		
Hong Kong	0.02	1.04	0.02	0.25	-0.48	0.52	0.04	0.23	-0.41	0.49		
Brazil	0.02	1.04	0.02	0.07	-0.13	0.17	0.04	0.06	-0.07	0.16		
Egypt	0.02	1.04	0.02	0.09	-0.16	0.20	0.04	0.06	-0.08	0.16		
Spain	0.02	1.04	0.02	0.09	-0.16	0.20	0.04	0.08	-0.11	0.20		
Australia	0.02	1.04	0.02	0.11	-0.19	0.23	0.04	0.09	-0.14	0.23		
United Kingdom	0.02	1.04	0.02	0.10	-0.17	0.21	0.04	0.08	-0.11	0.19		
Germany	0.02	1.04	0.02	0.07	-0.12	0.16	0.04	0.06	-0.08	0.16		
United States	-0.17	1.31	-0.18	0.03	-0.24	-0.12	0.29	0.03	0.24	0.34	*	*
Sweden	-0.28	1.22	-0.30	0.04	-0.38	-0.21	0.21	0.04	0.14	0.29	*	*
Japan	-0.45	1.22	-0.48	0.04	-0.56	-0.40	0.21	0.04	0.14	0.28	*	*

Note. *significant mu/lambda identified from whether the 95% CI for the parameter contains 0.0. Due to rounding, sometimes the estimate may be '-0.00' [slightly below zero] or '0.00' [slightly above zero]. Standard errors estimated using the inverse of the observed information matrix and can sometimes be singular leading to no estimated standard error for some parameters.

8.2 Health problems

```r
i <- i + 1
cur.var <- OUTCOME.LIST[i]
fit <- hetop_pml(
  data = df.raw |> filter(!is.na(ANNUAL_WEIGHT_C2))
  , var = cur.var
  , group = as.name("COUNTRY")
  , wgt = as.name("ANNUAL_WEIGHT_C2")
  , psu =  as.name("PSU")
  , strata = as.name("STRATA")

  , pen = exp(seq(-5,5,0.5))
  , pen.type="alf"
)

get_plot_latent_mean(fit)
```

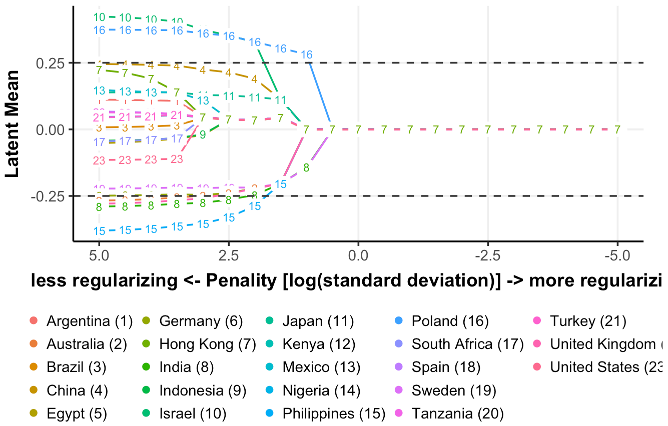


```r
get_plot_discrimination(fit)
```

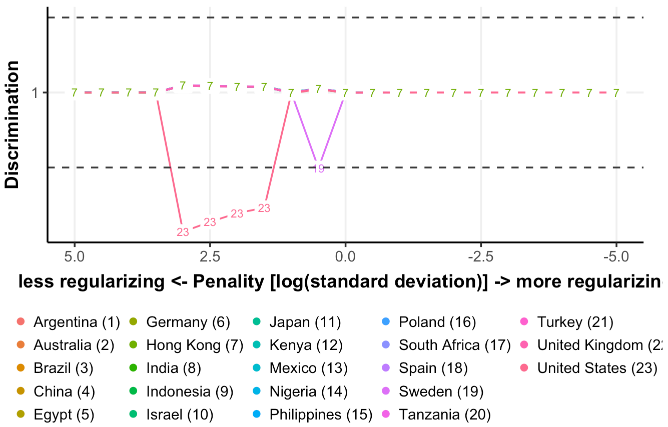


```r
plot_iccs(fit, "icc")
```

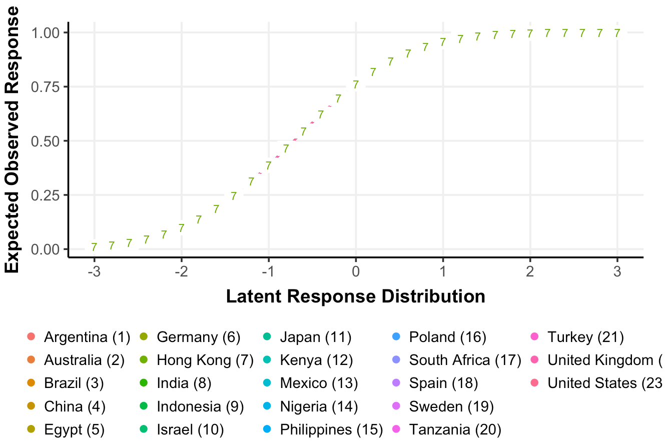

```r
format_ft_hetop(fit, 2, cur.var)
```

Health problems (HEALTH_PROB_Y2): Threshold Table.				
**threshold**	**est.unstd**	**se.unstd**	**est.unstd.lb**	**est.unstd.ub**
c[1]	-0.71	0.03	-0.77	-0.65

```r
format_ft_hetop(fit, 1, cur.var)
```

Health problems (HEALTH_PROB_Y2): HETOP summary table when penality is nu=0.

COUNTRY	mu.std	lambda.std	mu.unstd	mu.unstd.se	mu.unstd.lb	mu.unstd.ub	log.lambda.unstd	log.lambda.unstd.se	log.lambda.unstd.lb	log.lambda.unstd.ub	mu.nonzero	log.lambda.nonzero
Japan	0.00	1.00	0.00	0.13	-0.25	0.25	0.00	0.20	-0.39	0.39		
Poland	0.00	1.00	0.00	0.12	-0.24	0.24	0.00	0.24	-0.47	0.47		
China	0.00	1.00	0.00	0.15	-0.30	0.30	0.00	0.24	-0.48	0.48		
Israel	0.00	1.00	0.00	0.15	-0.30	0.30	0.00	0.33	-0.66	0.66		
Kenya	0.00	1.00	0.00	0.25	-0.50	0.50	0.00	0.34	-0.68	0.68		
Tanzania	0.00	1.00	0.00				0.00					
Argentina	0.00	1.00	0.00	0.06	-0.12	0.12	0.00					
Mexico	0.00	1.00	0.00	0.24	-0.47	0.47	0.00					
Spain	0.00	1.00	0.00	0.12	-0.24	0.24	0.00	0.07	-0.14	0.14		
Nigeria	0.00	1.00	0.00	0.15	-0.30	0.30	0.00	0.19	-0.37	0.37		
Brazil	0.00	1.00	0.00	0.15	-0.30	0.30	0.00	0.17	-0.34	0.34		
Hong Kong	0.00	1.00	0.00	0.44	-0.86	0.86	0.00	0.87	-1.70	1.70		
Turkey	0.00	1.00	0.00	0.11	-0.22	0.22	0.00	0.43	-0.83	0.83		
Indonesia	0.00	1.00	0.00	0.12	-0.24	0.24	0.00	0.23	-0.45	0.45		
South Africa	0.00	1.00	0.00				0.00	0.07	-0.14	0.14		
Egypt	0.00	1.00	0.00				0.00					
Australia	-0.00	1.00	-0.00				-0.00					
United Kingdom	-0.00	1.00	-0.00				-0.00					
Philippines	-0.00	1.00	-0.00				-0.00					
Germany	-0.00	1.00	-0.00				-0.00					
India	-0.00	1.00	-0.00				-0.00					
United States	-0.00	1.00	-0.00				-0.00					
Sweden	-0.00	1.00	-0.00				-0.00					

Note. *significant mu/lambda identified from whether the 95% CI for the parameter contains 0.0. Due to rounding, sometimes the estimate may be '-0.00' [slightly below zero] or '0.00' [slightly above zero]. Standard errors estimated using the inverse of the observed information matrix and can sometimes be singular leading to no estimated standard error for some parameters.

8.3 Pain in past 4 weeks

```r
i <- i + 1
cur.var <- OUTCOME.LIST[i]
fit <- hetop_pml(
  data = df.raw |> filter(!is.na(ANNUAL_WEIGHT_C2))
  , var = cur.var
  , group = as.name("COUNTRY")
  , wgt = as.name("ANNUAL_WEIGHT_C2")
  , psu =  as.name("PSU")
  , strata = as.name("STRATA")

  , pen = exp(seq(-5,5,0.5))
  , pen.type="alf"
)

get_plot_latent_mean(fit)
```

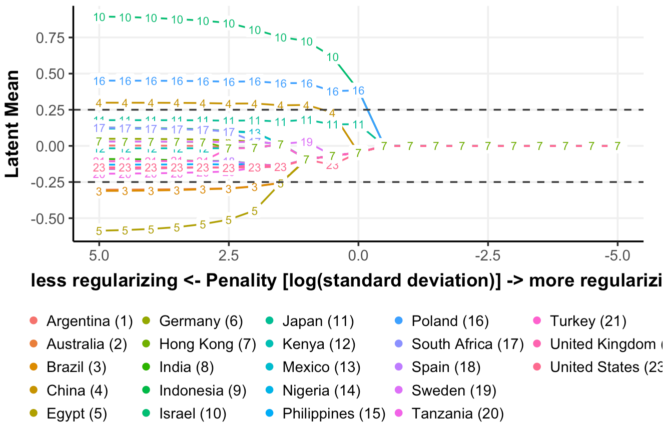


```
get_plot_discrimination(fit)
```

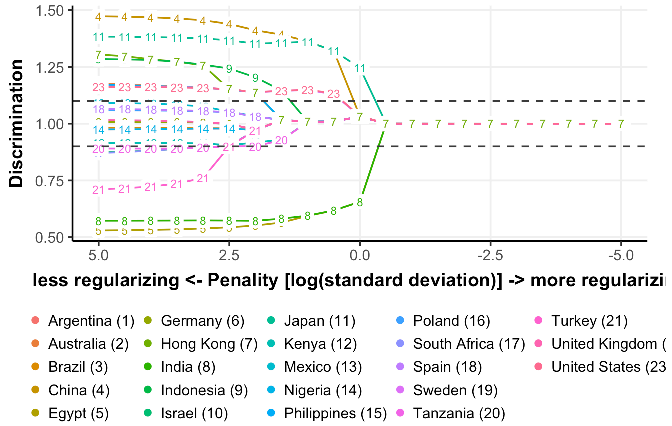


```
plot_iccs(fit, "icc")
```

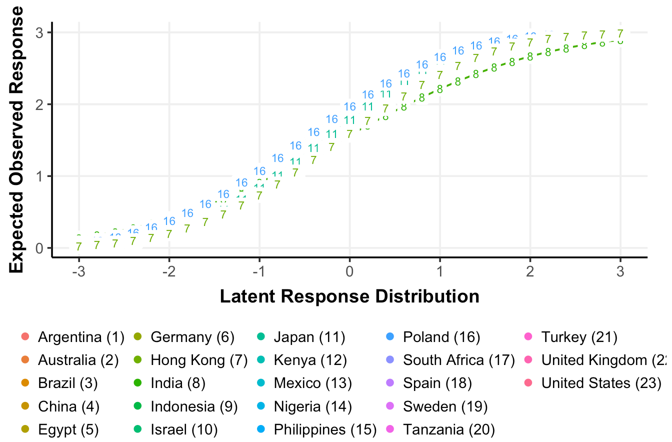


```
format_ft_hetop(fit, 2, cur.var)
```

Pain in past 4 weeks (BODILY_PAIN_Y2): Threshold Table.

threshold	est.unstd	se.unstd	est.unstd.lb	est.unstd.ub
c[1]	-1.14	0.03	-1.21	-1.08
c[2]	-0.10	0.02	-0.15	-0.06

Pain in past 4 weeks (BODILY_PAIN_Y2): Threshold Table.				
threshold	est.unstd	se.unstd	est.unstd.lb	est.unstd.ub
c[3]	0.79	0.03	0.74	0.85

```
format_ft_hetop(fit, 1, cur.var)
```

Pain in past 4 weeks (BODILY_PAIN_Y2): HETOP summary table when penality is nu=0.												
COUNTRY	mu.std	lambda.std	mu.unstd	mu.unstd.se	mu.unstd.lb	mu.unstd.ub	log.lambda.unstd	log.lambda.unstd.se	log.lambda.unstd.lb	log.lambda.unstd.ub	mu.nonzero	log.lambda.nonzero
Israel	0.37	1.03	0.39	0.11	0.17	0.61	0.03	0.12	-0.20	0.26	*	
Poland	0.37	1.03	0.38	0.06	0.26	0.51	0.03	0.06	-0.09	0.16	*	
Japan	0.15	1.24	0.15	0.04	0.07	0.23	0.22	0.04	0.13	0.30	*	*
Sweden	-0.04	1.03	-0.05	0.05	-0.14	0.05	0.03	0.05	-0.06	0.13		
China	-0.04	1.03	-0.05	0.09	-0.23	0.14	0.03	0.10	-0.16	0.23		
Germany	-0.04	1.03	-0.05	0.07	-0.18	0.09	0.03	0.06	-0.09	0.15		
Indonesia	-0.04	1.03	-0.05	0.10	-0.24	0.15	0.03	0.10	-0.17	0.23		
Mexico	-0.04	1.03	-0.05	0.10	-0.25	0.16	0.03	0.10	-0.16	0.23		
Kenya	-0.04	1.03	-0.05	0.06	-0.16	0.07	0.03	0.05	-0.07	0.13		
South Africa	-0.04	1.03	-0.05	0.16	-0.36	0.26	0.03	0.14	-0.24	0.30		
Argentina	-0.04	1.03	-0.05	0.09	-0.22	0.13	0.03	0.08	-0.13	0.19		
Hong Kong	-0.04	1.03	-0.05	0.18	-0.40	0.31	0.03	0.19	-0.34	0.40		
Turkey	-0.04	1.03	-0.05	0.22	-0.47	0.38	0.03	0.17	-0.31	0.37		
India	-0.04	0.66	-0.05	0.10	-0.24	0.15	-0.42	0.07	-0.56	-0.28		*
Spain	-0.04	1.03	-0.05	0.09	-0.22	0.13	0.03	0.08	-0.13	0.20		
Philippines	-0.04	1.03	-0.05	0.09	-0.23	0.14	0.03	0.09	-0.15	0.21		
Nigeria	-0.04	1.03	-0.05	0.09	-0.22	0.13	0.03	0.08	-0.12	0.19		
United Kingdom	-0.04	1.03	-0.05	0.08	-0.21	0.12	0.03	0.08	-0.12	0.18		
Egypt	-0.04	0.66	-0.05	0.16	-0.36	0.27	-0.42	0.11	-0.63	-0.21		*
Australia	-0.04	1.03	-0.05	0.10	-0.25	0.16	0.03	0.10	-0.16	0.23		
Tanzania	-0.04	1.03	-0.05	0.07	-0.18	0.09	0.03	0.06	-0.09	0.15		
Brazil	-0.05	1.03	-0.05	0.08	-0.21	0.12	0.03	0.07	-0.11	0.17		
United States	-0.05	1.03	-0.05	0.04	-0.12	0.02	0.03	0.03	-0.03	0.10		

Note. *significant mu/lambda identified from whether the 95% CI for the parameter contains 0.0. Due to rounding, sometimes the estimate may be '-0.00' [slightly below zero] or '0.00' [slightly above zero]. Standard errors estimated using the inverse of the observed information matrix and can sometimes be singular leading to no estimated standard error for some parameters.

8.4 Days exercise per week

```
i <- i + 1
cur.var <- OUTCOME.LIST[i]
fit <- hetop_pml(
  data = df.raw |> filter(!is.na(ANNUAL_WEIGHT_C2))
  , var = cur.var
  , group = as.name("COUNTRY")
  , wgt = as.name("ANNUAL_WEIGHT_C2")
  , psu =  as.name("PSU")
  , strata = as.name("STRATA")

  , pen = exp(seq(-5,5,0.5))
  , pen.type="alf"
)

get_plot_latent_mean(fit)
```

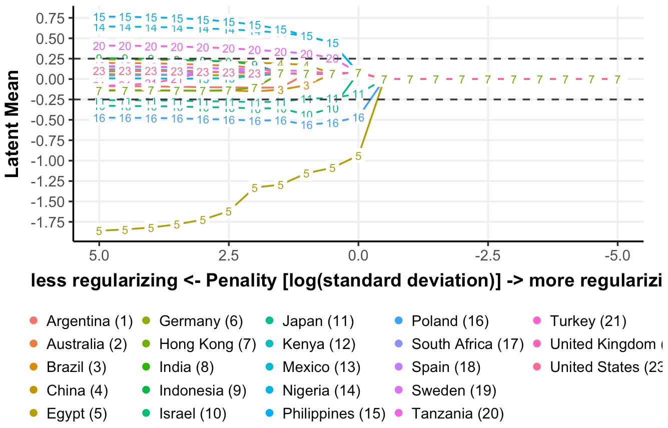


```
get_plot_discrimination(fit)
```

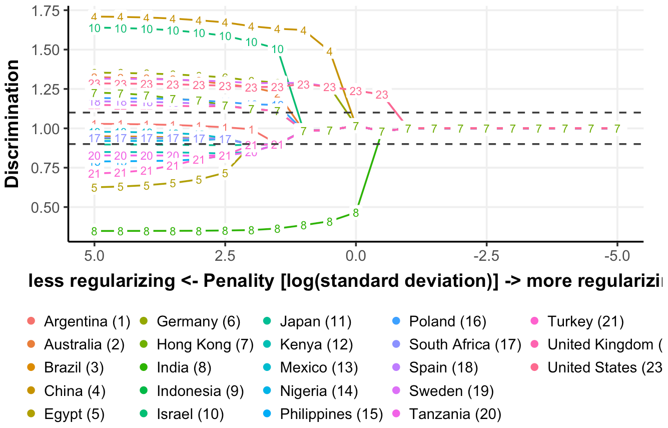


```
plot_iccs(fit, "icc")
```

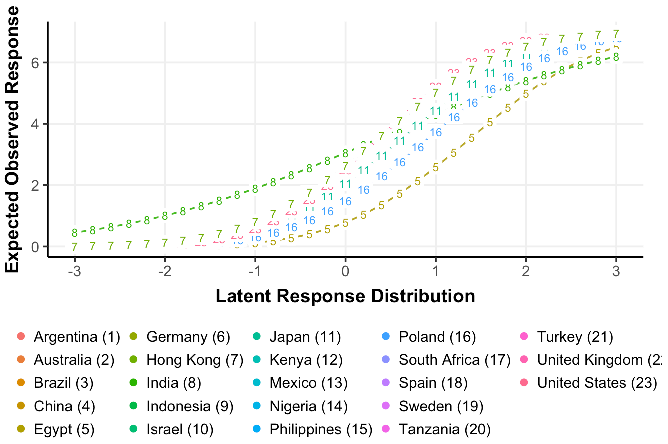


```
format_ft_hetop(fit, 2, cur.var)
```

Days exercise per week (DAYS_EXERCISE_Y2): Threshold Table.

threshold	est.unstd	se.unstd	est.unstd.lb	est.unstd.ub
c[1]	-0.41			
c[2]	-0.12			

Days exercise per week (DAYS_EXERCISE_Y2): Threshold Table.				
**threshold**	**est.unstd**	**se.unstd**	**est.unstd.lb**	**est.unstd.ub**
c[3]	0.20			
c[4]	0.54			
c[5]	0.73			
c[6]	0.97			
c[7]	1.11			

```
format_ft_hetop(fit, 1, cur.var)
```

Days exercise per week (DAYS_EXERCISE_Y2): HETOP summary table when penality is nu=0.

COUNTRY	mu.std	lambda.std	mu.unstd	mu.unstd.se	mu.unstd.lb	mu.unstd.ub	log.lambda.unstd	log.lambda.unstd.se	log.lambda.unstd.lb	log.lambda.unstd.ub	mu.nonzero	log.lambda.nonzero
Nigeria	0.07	1.02	0.08	0.13	-0.18	0.34	0.02					
Tanzania	0.07	1.02	0.08	0.06	-0.03	0.19	0.02					
Philippines	0.07	1.02	0.08	0.14	-0.20	0.36	0.02					
Sweden	0.07	1.22	0.08				0.21					
China	0.07	1.02	0.08	0.04	0.00	0.15	0.02	0.06	-0.09	0.13	*	
Indonesia	0.07	1.02	0.08	0.07	-0.06	0.22	0.02	0.05	-0.09	0.12		
Kenya	0.07	1.02	0.08				0.02	0.01	-0.01	0.04		
Australia	0.07	1.02	0.08	0.07	-0.06	0.22	0.02	0.07	-0.11	0.15		
Spain	0.07	1.02	0.08	0.06	-0.03	0.19	0.02	0.06	-0.10	0.14		
India	0.07	0.49	0.08	0.13	-0.19	0.34	-0.76	0.06	-0.88	-0.65		*
Turkey	0.07	1.02	0.08	0.20	-0.32	0.48	0.02	0.06	-0.11	0.14		
South Africa	0.07	1.02	0.08	0.14	-0.19	0.34	0.02	0.09	-0.15	0.19		
United Kingdom	0.07	1.02	0.08	0.02	0.04	0.11	0.02	0.05	-0.09	0.12	*	
Mexico	0.07	1.02	0.08	0.05	-0.03	0.19	0.02	0.07	-0.11	0.15		
Hong Kong	0.07	1.02	0.08	0.11	-0.13	0.29	0.02					
Germany	0.07	1.02	0.08				0.02	0.05	-0.07	0.11		
United States	0.07	1.22	0.08				0.21					
Argentina	0.07	1.02	0.08				0.02	0.06	-0.10	0.14		
Brazil	0.07	1.02	0.08				0.02	0.05	-0.07	0.11		
Israel	0.07	1.02	0.08				0.02					
Japan	-0.17	1.02	-0.18				0.02					
Poland	-0.43	1.02	-0.47	0.04	-0.54	-0.39	0.02	0.06	-0.09	0.13	*	
Egypt	-0.87	1.02	-0.94	0.12	-1.17	-0.70	0.02	0.09	-0.15	0.19	*	

Note. *significant mu/lambda identified from whether the 95% CI for the parameter contains 0.0. Due to rounding, sometimes the estimate may be '-0.00' [slightly below zero] or '0.00' [slightly above zero]. Standard errors estimated using the inverse of the observed information matrix and can sometimes be singular leading to no estimated standard error for some parameters.

9 Socioeconomic Outcomes

9.1 Financial security

```
i <- i + 1
cur.var <- OUTCOME.LIST[i]
fit <- hetop_pml(
```

```
  data = df.raw |> filter(!is.na(ANNUAL_WEIGHT_C2))
  , var = cur.var
  , group = as.name("COUNTRY")
  , wgt = as.name("ANNUAL_WEIGHT_C2")
  , psu =  as.name("PSU")
  , strata = as.name("STRATA")

  , pen = exp(seq(-5,5,0.5))
  , pen.type="alf"
)

get_plot_latent_mean(fit)
```

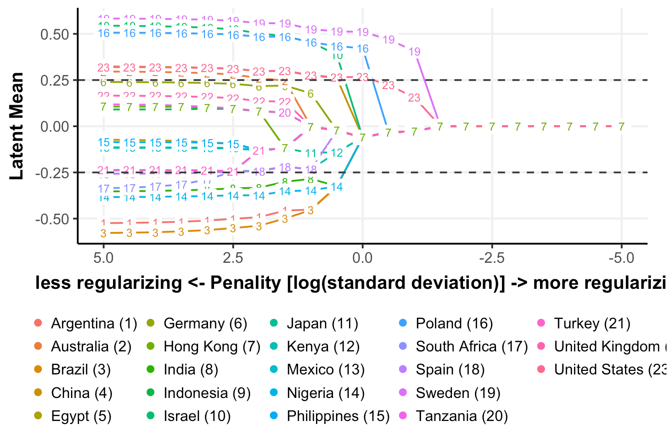


```
get_plot_discrimination(fit)
```

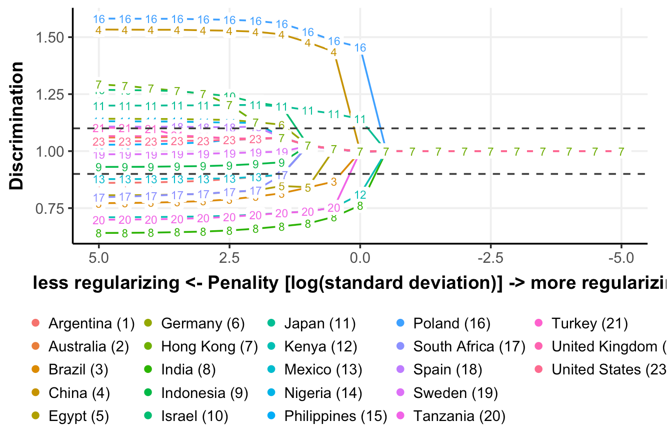


```
plot_iccs(fit, "icc")
```

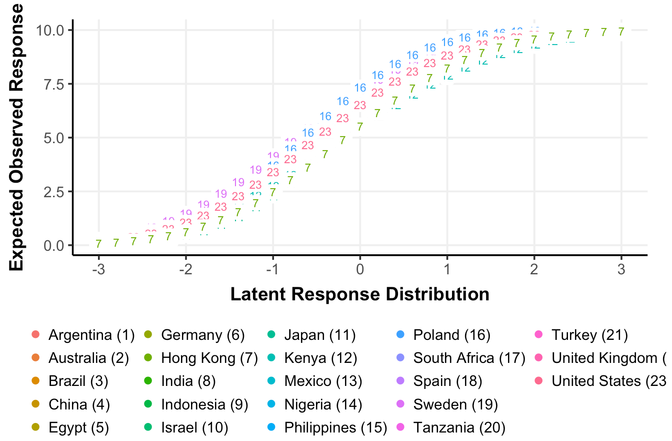


```
format_ft_hetop(fit, 2, cur.var)
```

Financial security (EXPENSES_Y2): Threshold Table.				
threshold	est.unstd	se.unstd	est.unstd.lb	est.unstd.ub
c[1]	-1.11	0.03	-1.18	-1.05
c[2]	-1.01	0.03	-1.07	-0.94
c[3]	-0.83	0.03	-0.89	-0.77
c[4]	-0.63	0.03	-0.69	-0.57
c[5]	-0.45	0.03	-0.50	-0.40
c[6]	-0.15	0.03	-0.20	-0.10
c[7]	0.05	0.03	-0.00	0.10
c[8]	0.33	0.03	0.28	0.38
c[9]	0.71	0.03	0.65	0.76
c[10]	1.05	0.03	0.99	1.12

```
format_ft_hetop(fit, 1, cur.var)
```

Financial security (EXPENSES_Y2): HETOP summary table when penality is nu=0.												
COUNTRY	mu.std	lambda.std	mu.unstd	mu.unstd.se	mu.unstd.lb	mu.unstd.ub	log.lambda.unstd	log.lambda.unstd.se	log.lambda.unstd.lb	log.lambda.unstd.ub	mu.nonzero	log.lambda.nonzero
Sweden	0.50	1.00	0.51	0.05	0.41	0.61	-0.00	0.05	-0.09	0.09	*	
Poland	0.41	1.44	0.42	0.05	0.33	0.52	0.38	0.06	0.27	0.48	*	*
United States	0.26	1.00	0.27	0.04	0.20	0.34	-0.00	0.03	-0.06	0.06	*	
China	-0.06	1.00	-0.06	0.10	-0.25	0.13	-0.00	0.10	-0.21	0.20		
Germany	-0.06	1.00	-0.06	0.07	-0.20	0.09	-0.00	0.07	-0.13	0.13		
Israel	-0.06	1.00	-0.06	0.21	-0.47	0.36	-0.00	0.19	-0.38	0.37		
Australia	-0.06	1.00	-0.06	0.11	-0.27	0.15	-0.00	0.10	-0.19	0.18		
United Kingdom	-0.06	1.00	-0.06	0.09	-0.23	0.11	-0.00	0.08	-0.15	0.15		
Tanzania	-0.06	1.00	-0.06	0.07	-0.20	0.08	-0.00	0.05	-0.11	0.10		
Indonesia	-0.06	1.00	-0.06	0.10	-0.25	0.13	-0.00	0.08	-0.17	0.16		
Hong Kong	-0.06	1.00	-0.06	0.18	-0.42	0.30	-0.00	0.19	-0.37	0.37		
Turkey	-0.06	1.00	-0.06	0.23	-0.50	0.38	-0.00	0.21	-0.42	0.42		
Egypt	-0.06	1.00	-0.06	0.09	-0.24	0.12	-0.00	0.08	-0.15	0.15		
Philippines	-0.06	1.00	-0.06	0.09	-0.25	0.13	-0.00	0.09	-0.17	0.17		
Mexico	-0.06	1.00	-0.06	0.10	-0.26	0.14	-0.00	0.09	-0.17	0.17		
South Africa	-0.06	1.00	-0.06	0.17	-0.38	0.26	-0.00	0.13	-0.27	0.26		
Spain	-0.06	1.00	-0.06	0.10	-0.25	0.13	-0.00	0.09	-0.18	0.17		
Kenya	-0.06	0.81	-0.06	0.07	-0.21	0.08	-0.21	0.06	-0.32	-0.10		*
India	-0.06	0.77	-0.06	0.09	-0.23	0.11	-0.27	0.06	-0.40	-0.15		*
Nigeria	-0.06	1.00	-0.06	0.11	-0.27	0.15	-0.00	0.10	-0.19	0.19		
Argentina	-0.06	1.00	-0.06	0.11	-0.27	0.15	-0.00	0.09	-0.18	0.17		
Japan	-0.06	1.14	-0.06	0.04	-0.14	0.02	0.13	0.04	0.05	0.21		*
Brazil	-0.06	1.00	-0.06	0.09	-0.24	0.12	-0.00	0.07	-0.15	0.14		

Note. *significant mu/lambda identified from whether the 95% CI for the parameter contains 0.0. Due to rounding, sometimes the estimate may be '-0.00' [slightly below zero] or '0.00' [slightly above zero]. Standard errors estimated using the inverse of the observed information matrix and can sometimes be singular leading to no estimated standard error for some parameters.

9.2 Material security

```r
i <- i + 1
cur.var <- OUTCOME.LIST[i]
fit <- hetop_pml(
  data = df.raw |> filter(!is.na(ANNUAL_WEIGHT_C2))
  , var = cur.var
  , group = as.name("COUNTRY")
  , wgt = as.name("ANNUAL_WEIGHT_C2")
  , psu =  as.name("PSU")
  , strata = as.name("STRATA")

  , pen = exp(seq(-5,5,0.5))
  , pen.type="alf"
)
get_plot_latent_mean(fit)
```

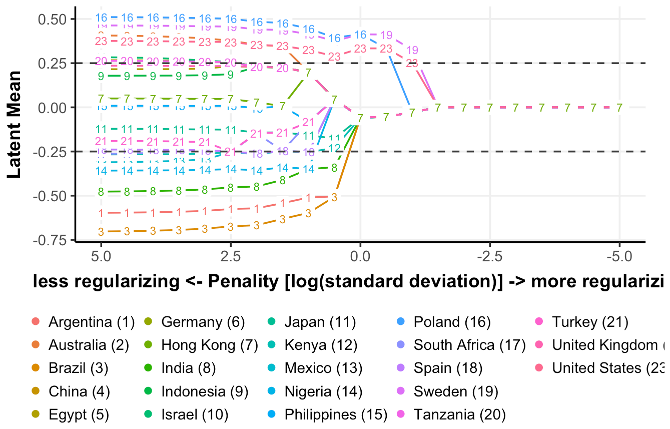


```r
get_plot_discrimination(fit)
```

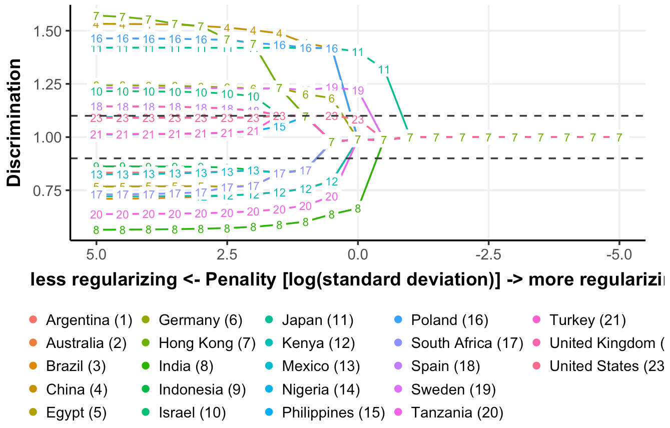


```r
plot_iccs(fit, "icc")
```

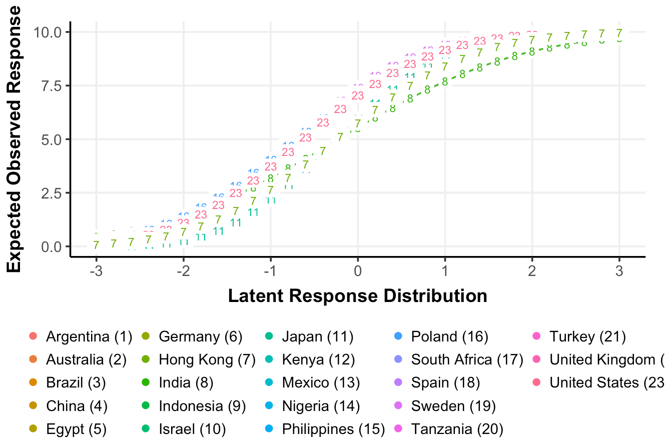

```r
format_ft_hetop(fit, 2, cur.var)
```

Material security (WORRY_SAFETY_Y2): Threshold Table.				
**threshold**	**est.unstd**	**se.unstd**	**est.unstd.lb**	**est.unstd.ub**
c[1]	-1.14	0.03	-1.21	-1.08
c[2]	-1.04	0.03	-1.11	-0.98
c[3]	-0.88	0.03	-0.94	-0.82
c[4]	-0.68	0.03	-0.74	-0.63
c[5]	-0.50	0.03	-0.56	-0.45
c[6]	-0.21	0.03	-0.26	-0.16
c[7]	-0.03	0.02	-0.08	0.02
c[8]	0.22	0.03	0.17	0.27
c[9]	0.57	0.03	0.52	0.63
c[10]	0.95	0.03	0.89	1.01

```r
format_ft_hetop(fit, 1, cur.var)
```

Material security (WORRY_SAFETY_Y2): HETOP summary table when penality is nu=0.

COUNTRY	mu.std	lambda.std	mu.unstd	mu.unstd.se	mu.unstd.lb	mu.unstd.ub	log.lambda.unstd	log.lambda.unstd.se	log.lambda.unstd.lb	log.lambda.unstd.ub	mu.nonzero	log.lambda.nonzero
Poland	0.40	0.99	0.41	0.06	0.29	0.54	-0.01	0.08	-0.16	0.14	*	
Sweden	0.40	1.21	0.41	0.04	0.33	0.50	0.20	0.04	0.11	0.29	*	*
United States	0.33	1.08	0.33	0.03	0.27	0.40	0.08	0.03	0.02	0.14	*	*
Germany	-0.06	0.99	-0.06	0.08	-0.21	0.09	-0.01	0.08	-0.16	0.14		
China	-0.06	0.99	-0.06	0.09	-0.24	0.12	-0.01	0.10	-0.21	0.19		
Tanzania	-0.06	0.99	-0.06	0.07	-0.20	0.08	-0.01	0.06	-0.12	0.10		
Australia	-0.06	0.99	-0.06	0.13	-0.30	0.19	-0.01	0.11	-0.23	0.21		
United Kingdom	-0.06	0.99	-0.06	0.09	-0.23	0.12	-0.01	0.09	-0.18	0.16		
Israel	-0.06	0.99	-0.06	0.11	-0.28	0.17	-0.01	0.11	-0.23	0.21		
Egypt	-0.06	0.99	-0.06	0.09	-0.24	0.13	-0.01	0.08	-0.16	0.14		
Indonesia	-0.06	0.99	-0.06	0.10	-0.25	0.14	-0.01	0.09	-0.18	0.16		
Philippines	-0.06	0.99	-0.06	0.10	-0.25	0.13	-0.01	0.09	-0.19	0.16		
Hong Kong	-0.06	0.99	-0.06	0.18	-0.41	0.30	-0.01	0.22	-0.44	0.42		
Turkey	-0.06	0.99	-0.06	0.22	-0.49	0.38	-0.01	0.21	-0.41	0.39		
South Africa	-0.06	0.99	-0.06	0.16	-0.38	0.26	-0.01	0.13	-0.27	0.24		
Mexico	-0.06	0.99	-0.06	0.11	-0.27	0.15	-0.01	0.09	-0.19	0.16		
Spain	-0.06	0.99	-0.06	0.10	-0.25	0.13	-0.01	0.09	-0.19	0.17		
Nigeria	-0.06	0.99	-0.06	0.10	-0.26	0.14	-0.01	0.09	-0.20	0.17		
India	-0.06	0.67	-0.06	0.10	-0.26	0.15	-0.41	0.07	-0.55	-0.27		*
Argentina	-0.06	0.99	-0.06	0.12	-0.29	0.17	-0.01	0.10	-0.20	0.18		
Kenya	-0.06	0.99	-0.06	0.06	-0.18	0.06	-0.01	0.05	-0.11	0.09		

Note. *significant mu/lambda identified from whether the 95% CI for the parameter contains 0.0. Due to rounding, sometimes the estimate may be '-0.00' [slightly below zero] or '0.00' [slightly above zero]. Standard errors estimated using the inverse of the observed information matrix and can sometimes be singular leading to no estimated standard error for some parameters.

Material security (WORRY_SAFETY_Y2): HETOP summary table when penality is nu=0.												
COUNTRY	mu.std	lambda.std	mu.unstd	mu.unstd.se	mu.unstd.lb	mu.unstd.ub	log.lambda.unstd	log.lambda.unstd.se	log.lambda.unstd.lb	log.lambda.unstd.ub	mu.nonzero	log.lambda.nonzero
Brazil	-0.06	0.99	-0.06	0.10	-0.26	0.14	-0.01	0.08	-0.17	0.14		
Japan	-0.06	1.39	-0.06	0.04	-0.13	0.01	0.34	0.04	0.26	0.41		*

Note. *significant mu/lambda identified from whether the 95% CI for the parameter contains 0.0. Due to rounding, sometimes the estimate may be '-0.00' [slightly below zero] or '0.00' [slightly above zero]. Standard errors estimated using the inverse of the observed information matrix and can sometimes be singular leading to no estimated standard error for some parameters.

9.3 Educational attainment

```r
i <- i + 1
cur.var <- OUTCOME.LIST[i]
fit <- hetop_pml(
  data = df.raw |> filter(!is.na(ANNUAL_WEIGHT_C2))
  , var = cur.var
  , group = as.name("COUNTRY")
  , wgt = as.name("ANNUAL_WEIGHT_C2")
  , psu =  as.name("PSU")
  , strata = as.name("STRATA")

  , pen = exp(seq(-5,5,0.5))
  , pen.type="alf"
)

get_plot_latent_mean(fit)
```

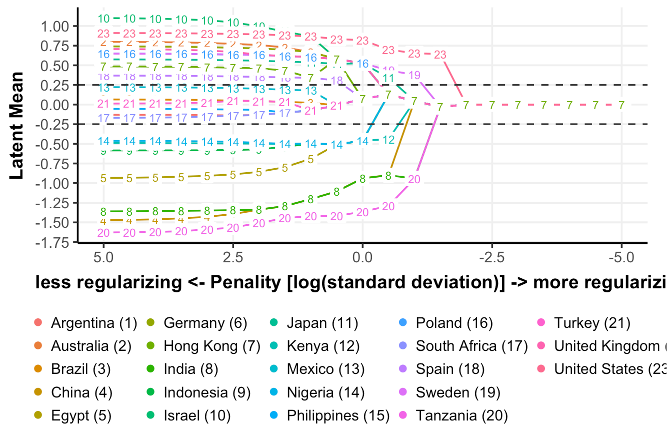


```r
get_plot_discrimination(fit)
```

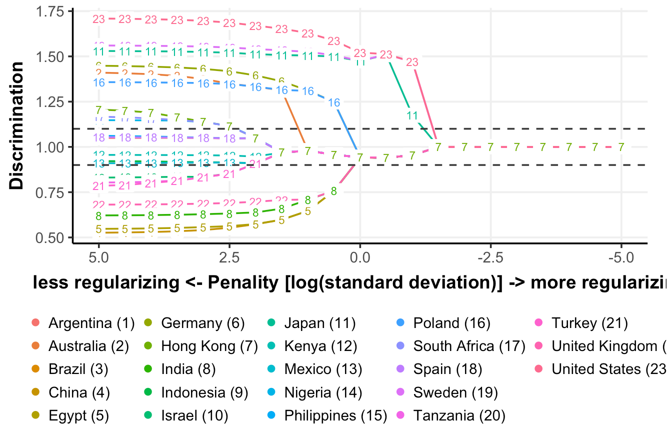


```r
plot_iccs(fit, "icc")
```

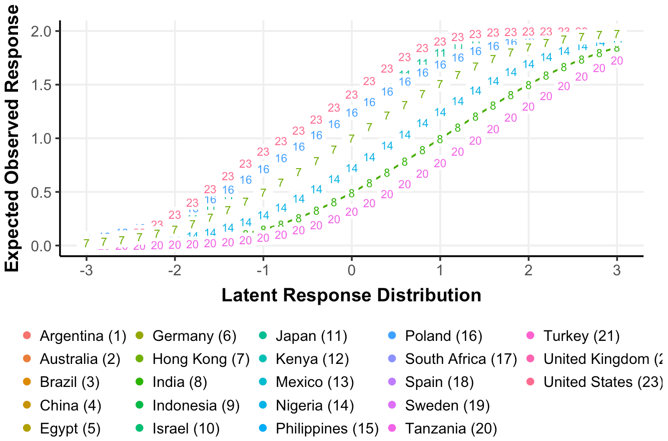


```
format_ft_hetop(fit, 2, cur.var)
```

Educational attainment (16+ years) (EDUCATION_3_Y2): Threshold Table.

threshold	est.unstd	se.unstd	est.unstd.lb	est.unstd.ub
c[1]	-0.82	0.03	-0.88	-0.75
c[2]	0.96	0.03	0.89	1.02

```
format_ft_hetop(fit, 1, cur.var)
```

Educational attainment (16+ years) (EDUCATION_3_Y2): HETOP summary table when penality is nu=0.

COUNTRY	mu.std	lambda.std	mu.unstd	mu.unstd.se	mu.unstd.lb	mu.unstd.ub	log.lambda.unstd	log.lambda.unstd.se	log.lambda.unstd.lb	log.lambda.unstd.ub	mu.nonzero	log.lambda.nonzero
United States	0.71	1.44	0.82	0.04	0.75	0.89	0.42	0.05	0.32	0.52	*	*
Sweden	0.45	1.40	0.53	0.05	0.44	0.62	0.39	0.06	0.28	0.50	*	*
Israel	0.45	0.95	0.53	0.13	0.27	0.78	-0.06	0.13	-0.31	0.19	*	
Germany	0.45	0.95	0.53	0.08	0.37	0.68	-0.06	0.09	-0.23	0.11	*	
Australia	0.45	0.95	0.53	0.12	0.30	0.75	-0.06	0.13	-0.31	0.19	*	
Poland	0.45	0.95	0.53	0.07	0.38	0.67	-0.06	0.08	-0.21	0.09	*	
United Kingdom	0.45	0.95	0.53	0.10	0.34	0.72	-0.06	0.08	-0.22	0.10	*	
Japan	0.45	1.40	0.52	0.04	0.44	0.61	0.39	0.05	0.29	0.49	*	*
Spain	0.06	0.95	0.07	0.11	-0.14	0.29	-0.06	0.10	-0.26	0.14		
Hong Kong	0.06	0.95	0.07	0.24	-0.39	0.54	-0.06	0.22	-0.49	0.37		
Mexico	0.06	0.95	0.07	0.12	-0.16	0.30	-0.06	0.10	-0.26	0.14		
Turkey	0.06	0.95	0.07	0.25	-0.42	0.56	-0.06	0.21	-0.46	0.34		
Brazil	0.06	0.95	0.07	0.09	-0.10	0.24	-0.06	0.08	-0.21	0.09		
South Africa	0.06	0.95	0.07	0.19	-0.31	0.45	-0.06	0.18	-0.41	0.29		
Philippines	0.06	0.95	0.07	0.11	-0.15	0.29	-0.06	0.10	-0.26	0.14		
Argentina	0.06	0.95	0.07	0.11	-0.15	0.29	-0.06	0.10	-0.26	0.14		
Nigeria	-0.40	0.95	-0.46	0.10	-0.66	-0.26	-0.06	0.11	-0.27	0.15	*	
Indonesia	-0.40	0.95	-0.46	0.11	-0.68	-0.24	-0.06	0.11	-0.27	0.15	*	

Note. *significant mu/lambda identified from whether the 95% CI for the parameter contains 0.0. Due to rounding, sometimes the estimate may be '-0.00' [slightly below zero] or '0.00' [slightly above zero]. Standard errors estimated using the inverse of the observed information matrix and can sometimes be singular leading to no estimated standard error for some parameters.

Educational attainment (16+ years) (EDUCATION_3_Y2): HETOP summary table when penality is nu=0.

COUNTRY	mu.std	lambda.std	mu.unstd	mu.unstd.se	mu.unstd.lb	mu.unstd.ub	log.lambda.unstd	log.lambda.unstd.se	log.lambda.unstd.lb	log.lambda.unstd.ub	mu.nonzero	log.lambda.nonzero
Kenya	-0.40	0.95	-0.46	0.07	-0.60	-0.32	-0.06	0.07	-0.19	0.07	*	
Egypt	-0.40	0.95	-0.46	0.11	-0.67	-0.25	-0.06	0.09	-0.23	0.11	*	
China	-0.81	0.95	-0.94	0.10	-1.13	-0.75	-0.06	0.08	-0.22	0.10	*	
India	-0.81	0.95	-0.94	0.08	-1.10	-0.78	-0.06	0.08	-0.21	0.09	*	
Tanzania	-1.17	0.95	-1.36	0.11	-1.57	-1.15	-0.06	0.12	-0.29	0.17	*	

Note. *significant mu/lambda identified from whether the 95% CI for the parameter contains 0.0. Due to rounding, sometimes the estimate may be '-0.00' [slightly below zero] or '0.00' [slightly above zero]. Standard errors estimated using the inverse of the observed information matrix and can sometimes be singular leading to no estimated standard error for some parameters.

9.4 Subjective financially status

```
i <- i + 1
cur.var <- OUTCOME.LIST[i]
fit <- hetop_pml(
  data = df.raw |> filter(!is.na(ANNUAL_WEIGHT_C2))
  , var = cur.var
  , group = as.name("COUNTRY")
  , wgt = as.name("ANNUAL_WEIGHT_C2")
  , psu =  as.name("PSU")
  , strata = as.name("STRATA")

  , pen = exp(seq(-5,5,0.5))
  , pen.type="alf"
)

get_plot_latent_mean(fit)
```

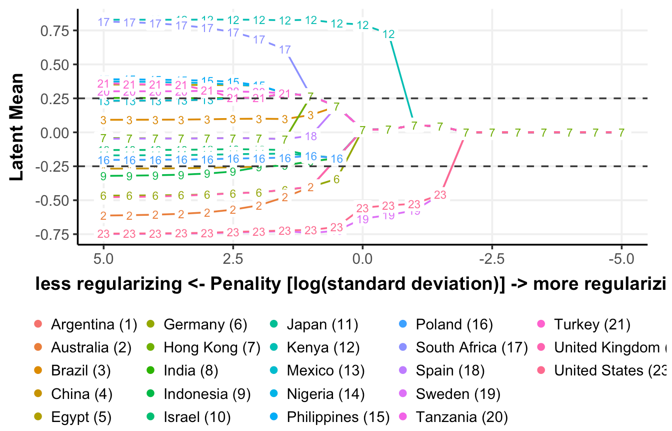


```
get_plot_discrimination(fit)
```

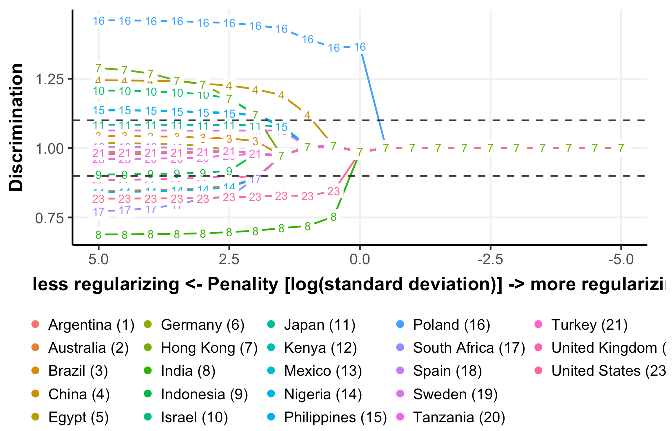


```
plot_iccs(fit, "icc")
```

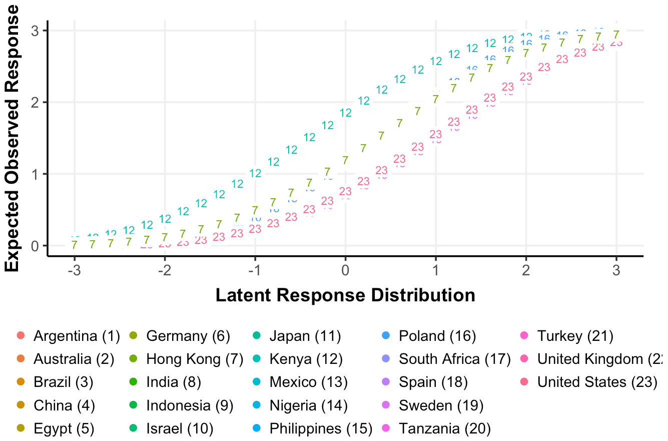


```
format_ft_hetop(fit, 2, cur.var)
```

Financially comfortable/getting by (INCOME_FEELINGS_Y2): Threshold Table.				
threshold	est.unstd	se.unstd	est.unstd.lb	est.unstd.ub
c[1]	-0.75	0.04	-0.82	-0.68
c[2]	0.49	0.03	0.44	0.55
c[3]	1.31	0.03	1.24	1.38

```
format_ft_hetop(fit, 1, cur.var)
```

Financially comfortable/getting by (INCOME_FEELINGS_Y2): HETOP summary table when penality is nu=0.												
COUNTRY	mu.std	lambda.std	mu.unstd	mu.unstd.se	mu.unstd.lb	mu.unstd.ub	log.lambda.unstd	log.lambda.unstd.se	log.lambda.unstd.lb	log.lambda.unstd.ub	mu.nonzero	log.lambda.nonzero
Kenya	0.77	0.99	0.79	0.06	0.67	0.92	-0.01	0.06	-0.14	0.11	*	
Tanzania	0.02	0.99	0.02	0.08	-0.14	0.19	-0.01	0.07	-0.15	0.12		
India	0.02	0.99	0.02	0.08	-0.13	0.17	-0.02	0.06	-0.13	0.10		
Nigeria	0.02	0.99	0.02	0.11	-0.20	0.24	-0.01	0.09	-0.19	0.16		
Egypt	0.02	0.99	0.02	0.12	-0.21	0.25	-0.01	0.10	-0.20	0.17		
Philippines	0.02	0.99	0.02	0.13	-0.24	0.28	-0.01	0.11	-0.24	0.21		
Argentina	0.02	0.99	0.02	0.11	-0.19	0.23	-0.01	0.09	-0.19	0.17		
South Africa	0.02	0.99	0.02	0.38	-0.73	0.77	-0.01	0.26	-0.53	0.50		
Mexico	0.02	0.99	0.02	0.12	-0.22	0.26	-0.01	0.10	-0.22	0.19		
Brazil	0.02	0.99	0.02	0.09	-0.15	0.19	-0.01	0.08	-0.16	0.13		
Turkey	0.02	0.99	0.02	0.28	-0.53	0.57	-0.01	0.23	-0.47	0.44		
Spain	0.02	0.99	0.02	0.10	-0.17	0.21	-0.01	0.09	-0.19	0.16		
Hong Kong	0.02	0.99	0.02	0.19	-0.35	0.39	-0.01	0.20	-0.41	0.38		
Israel	0.02	0.99	0.02	0.10	-0.18	0.22	-0.01	0.11	-0.22	0.19		
Indonesia	0.02	0.99	0.02	0.10	-0.18	0.22	-0.01	0.09	-0.20	0.17		
China	0.02	0.99	0.02	0.08	-0.14	0.18	-0.01	0.08	-0.18	0.15		
Australia	0.02	0.99	0.02	0.12	-0.22	0.26	-0.01	0.11	-0.23	0.20		
United Kingdom	0.02	0.99	0.02	0.10	-0.17	0.21	-0.01	0.09	-0.18	0.16		

Note. *significant mu/lambda identified from whether the 95% CI for the parameter contains 0.0. Due to rounding, sometimes the estimate may be '-0.00' [slightly below zero] or '0.00' [slightly above zero]. Standard errors estimated using the inverse of the observed information matrix and can sometimes be singular leading to no estimated standard error for some parameters.

Financially comfortable/getting by (INCOME_FEELINGS_Y2): HETOP summary table when penality is nu=0.

COUNTRY	mu.std	lambda.std	mu.unstd	mu.unstd.se	mu.unstd.lb	mu.unstd.ub	log.lambda.unstd	log.lambda.unstd.se	log.lambda.unstd.lb	log.lambda.unstd.ub	mu.nonzero	log.lambda.nonzero
Poland	0.02	1.35	0.02	0.05	-0.09	0.13	0.31	0.06	0.19	0.43		*
Japan	0.02	0.99	0.02	0.05	-0.08	0.12	-0.01	0.05	-0.10	0.08		
Germany	0.02	0.99	0.02	0.08	-0.14	0.18	-0.01	0.08	-0.16	0.13		
United States	-0.54	0.99	-0.55	0.05	-0.64	-0.47	-0.02	0.03	-0.08	0.05	*	
Sweden	-0.62	0.99	-0.64	0.06	-0.76	-0.52	-0.01	0.06	-0.13	0.10	*	

Note. *significant mu/lambda identified from whether the 95% CI for the parameter contains 0.0. Due to rounding, sometimes the estimate may be '-0.00' [slightly below zero] or '0.00' [slightly above zero]. Standard errors estimated using the inverse of the observed information matrix and can sometimes be singular leading to no estimated standard error for some parameters.
